# Metal-free alkene carbooxygenation following tandem intramolecular alkoxylation/Claisen rearrangement: stereocontrolled access to bridged [4.2.1] lactones†
†Electronic supplementary information (ESI) available. CCDC 1831051, 1831053, 1831054 and 1864037. For ESI and crystallographic data in CIF or other electronic format see DOI: 10.1039/c9sc00079h


**DOI:** 10.1039/c9sc00079h

**Published:** 2019-01-24

**Authors:** Long Li, Xin-Qi Zhu, Ying-Qi Zhang, Hao-Zhen Bu, Peng Yuan, Jinyu Chen, Jingyi Su, Xianming Deng, Long-Wu Ye

**Affiliations:** a State Key Laboratory of Physical Chemistry of Solid Surfaces , Key Laboratory for Chemical Biology of Fujian Province , College of Chemistry and Chemical Engineering , Xiamen University , Xiamen 361005 , China . Email: longwuye@xmu.edu.cn; b State Key Laboratory of Cellular Stress Biology , School of Life Sciences , Xiamen University , Xiamen , Fujian 361102 , China; c State Key Laboratory of Organometallic Chemistry , Shanghai Institute of Organic Chemistry , Chinese Academy of Sciences , Shanghai 200032 , China; d State Key Laboratory of Elemento-Organic Chemistry , Nankai University , Tianjin 300071 , China

## Abstract

Alkene carbooxygenation has attracted considerable attention over the past few decades as this approach provides an efficient access to various oxygen-containing molecules, especially the valuable O-heterocycles. However, examples of catalytic alkene carbooxygenation *via* a direct C–O cleavage are quite scarce, and the C–O cleavage in these cases is invariably initiated by transition metal-catalyzed oxidative addition. We report here a novel Brønsted acid-catalyzed intramolecular alkoxylation-initiated tandem sequence, which represents the first metal-free intramolecular alkoxylation/Claisen rearrangement. Significantly, an unprecedented Brønsted acid-catalyzed intramolecular alkene insertion into the C–O bond *via* a carbocation pathway was discovered. This method allows the stereocontrolled synthesis of valuable indole-fused bridged [4.2.1] lactones, providing ready access to biologically relevant scaffolds in a single synthetic step from an acyclic precursor. Moreover, such an asymmetric cascade cyclization has also been realized by employing a traceless chiral directing group. Control experiments favor the feasibility of a carbocation pathway for the process. In addition, biological tests showed that some of these newly synthesized indole-fused lactones exhibited their bioactivity as antitumor agents against different breast cancer cells, melanoma cells, and esophageal cancer cells.

## Abstract 2

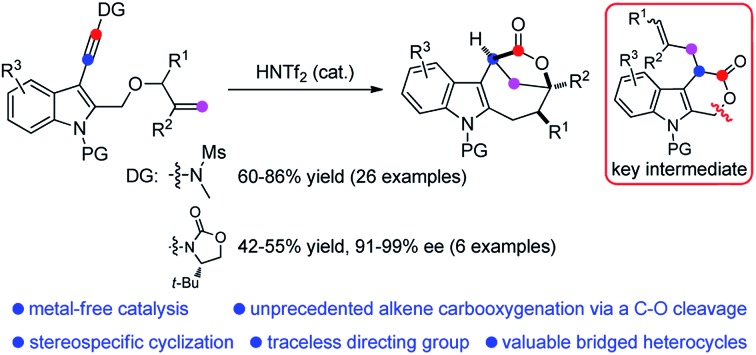
A metal-free intramolecular tandem sequence involving alkoxylation, Claisen rearrangement and lactone expansion has been achieved.

## Introduction

**Fig. 1:**
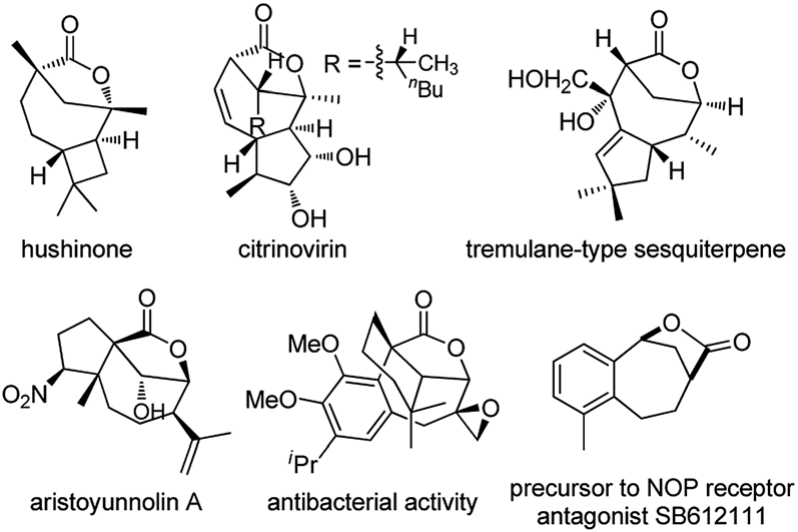
Selected bioactive molecules containing bridged [4.2.1] lactones.

Bridged [4.2.1] lactones are widely distributed heterocycles found in various natural products such as hushinone and citrinovirin, and bioactive compounds (Fig. 1).^1^ However, such bicyclic frameworks bearing both a medium ring and bridged unit are regarded as difficult skeletons to construct due to entropic effects and the ring strain factor,^2^ and very few methods have been reported to date.^1^,^3^ Hence, novel and stereocontrolled synthesis of bridged [4.2.1] lactone motifs allowing structurally diverse modification is in great demand in both organic and medicinal chemistry.

**Scheme 1:**
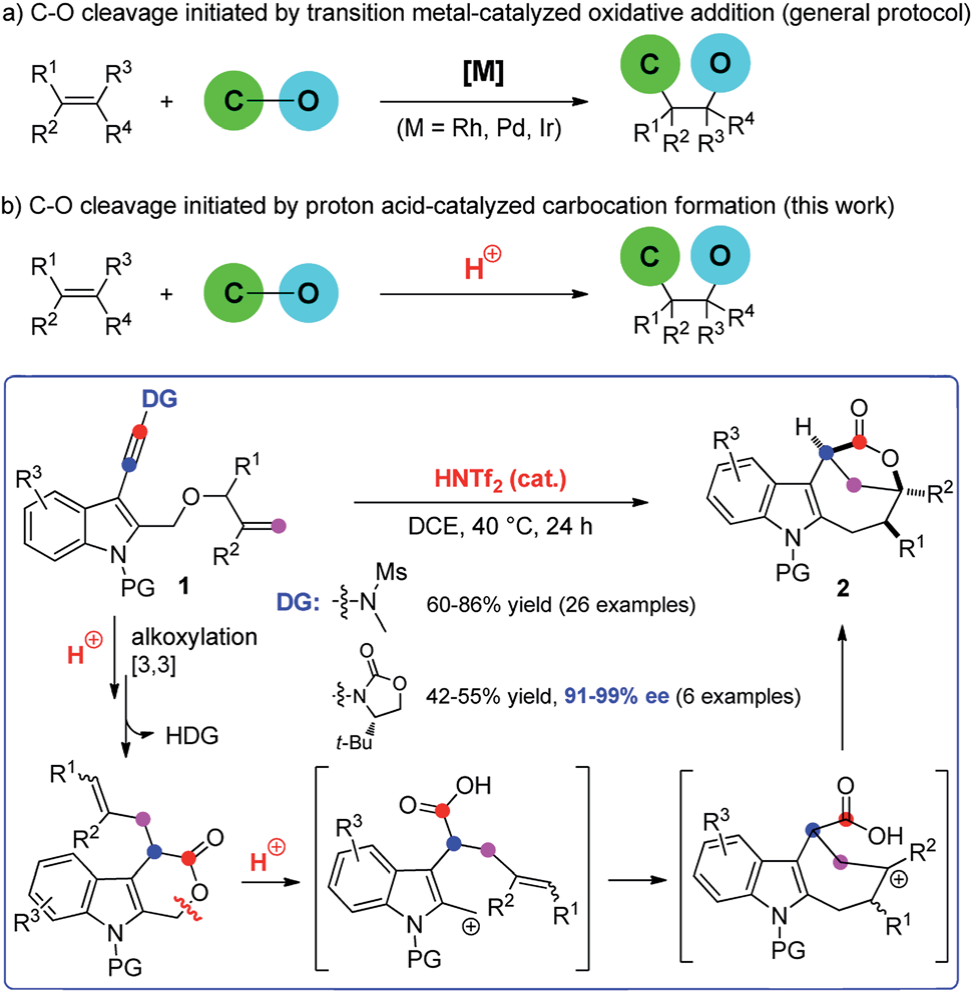
Catalytic alkene carbooxygenation *via* a C–O cleavage.

Difunctionalization of unactivated olefins in a single operation is one of the most valuable transformations in organic chemistry.^4^ Among them, alkene carbooxygenation is particularly attractive as this approach provides an efficient access to various oxygen-containing molecules, especially the valuable O-heterocycles, and various synthetic methods have been developed.^4^ However, examples of catalytic alkene carbooxygenation *via* a direct C–O cleavage are quite scarce, and the C–O cleavage in these cases is invariably initiated by transition metal-catalyzed oxidative addition (Scheme 1a).^5^,^6^ For example, Douglas *et al.* reported an elegant protocol for the rhodium-catalyzed intramolecular alkene oxyacylation reaction *via* an acyl C–O bond activation.6a,b In 2012, Nakao *et al.* disclosed an intramolecular oxycyanation of alkenes by palladium/BPh_3_ catalysis.6*c* Thus, the development of an alternative approach for the catalytic cleavage of the C–O bond and the addition reaction to the alkenes is highly desirable.

Because of their high bond-forming efficiency and atom economy, catalytic tandem intramolecular alkoxylation/Claisen rearrangements have received significant attention.^7^ In particular, such a cascade cyclization of alkynyl allyl ethers could lead to the formation of various valuable O-heterocycles, which is also established by Hashmi, Liu, Gagosz, Miyata, and others (Scheme 1a).^8^–^10^ While these achievements are impressive, almost all of these approaches rely on the use of noble metals such as gold and platinum as catalysts. In our recent study on the catalytic tandem reactions of ynamides for heterocycle synthesis,^11^,^12^ we realized the first metal-free intramolecular alkoxylation/Claisen rearrangement of indole-linked ynamide-allyl ethers (Scheme 1b). Interestingly, the resulting six-membered lactone intermediate further underwent an unprecedented Brønsted acid-catalyzed intramolecular carbooxygenation of olefins by C–O bond cleavage involving ring opening, carbocation rearrangement, and then ring closing. This Brønsted acid catalysis led to the highly efficient and stereocontrolled formation of valuable indole-fused bridged [4.2.1] lactones. Furthermore, such an asymmetric cascade cyclization was also realized by employing a traceless chiral directing group. The mechanistic rationale for this cascade reaction is strongly supported by a variety of control experiments. In this paper, we wish to report the results of our detailed investigations of this novel cascade cyclization, including the substrate scope, synthetic applications, biological tests and mechanistic studies.

## Results and discussion

Inspired by our previous work on the indolyl ynamide chemistry,12d–f we chose an indole-tethered ynamide **1a** as the model substrate for the initial study. As shown in Table 1, indole-fused lactone **2aa** was obtained in the presence of most non-noble metals (Table 1, entries 1–4), with Cu(OTf)_2_ giving the best yield of the desired **2aa** (Table 1, entry 4). Different from Hashmi's protocol,8*b**N*-methyl methanesulfonamide here not only serves as the directing group to achieve regioselective attack at the N-terminus of alkyne, but also can be removed spontaneously and regarded as a traceless directing group. Surprisingly, indole-fused bridged [4.2.1] lactone **2a** was detected as the main product by employing In(OTf)_3_ or Fe(OTf)_3_ as catalysts (Table 1, entries 5–6). Of note, typical gold catalysts, such as Ph_3_PAuNTf_2_ and IPrAuNTf_2_, were not effective in promoting this reaction and the decomposition of **1a** was observed in these cases (Table 1, entries 7–8). Various Brønsted acids were also evaluated, but typical organic acids (*e.g.*, TFA, MsOH, and TsOH) were not capable of catalyzing the reaction.^13^ Gratifyingly, HOTf and HNTf_2_ (Table 1, entries 9–10) could effectively catalyze this cascade cyclization,^14^ and the bridged lactone **2a** was obtained in 81% yield in the latter case (Table 1, entry 10). The reaction proved to be less efficient when it was performed at 60 °C (Table 1, entry 11) or in other solvents.^13^ The observed excellent efficiency with HNTf_2_ as the catalyst can be explained by its high Brønsted acidity combined with the low nucleophilicity of its counterion.^15^,^16^

**Table 1:** Optimization of reaction conditions^*a*^

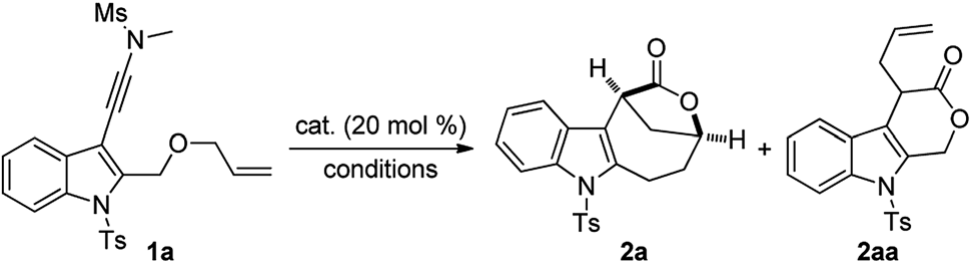					
Entry	Catalyst	Reaction conditions	Yield^*b*^ (%)		
			**2a**	**2aa**	**1a**
1	Y(OTf)_3_	DCE, 40 °C, 48 h	<1	20	75
2	Yb(OTf)_3_	DCE, 40 °C, 48 h	<1	50	30
3	Zn(OTf)_2_	DCE, 40 °C, 48 h	<1	70	25
4	Cu(OTf)_2_	DCE, 40 °C, 24 h	<1	84	<1
5	In(OTf)_3_	DCE, 40 °C, 48 h	63	15	<1
6	Fe(OTf)_3_	DCE, 40 °C, 48 h	74	<5	<1
7^*c*^	Ph_3_PAuNTf_2_	DCE, rt, 10 h	<1	<1	<1
8^*c*^	IPrAuNTf_2_	DCE, rt, 10 h	<1	<1	<1
9	HOTf	DCE, 40 °C, 24 h	72	<1	<1
**10**	**HNTf**_**2**_	**DCE, 40 °C, 24 h**	**81**	**<1**	**<1**
11	HNTf_2_	DCE, 60 °C, 18 h	67	<1	<1

^*a*^Reaction conditions: **1a** (0.1 mmol), catalyst (0.02 mmol), DCE (2 mL), 40–60 °C, in vials.

^*b*^Measured by ^1^H NMR using diethyl phthalate as the internal standard.

^*c*^5 mol% of catalyst was used.

With the optimal reaction conditions in hand (Table 1, entry 10), the scope of this novel tandem reaction was explored (Table 2). This metal-free cascade cyclization proceeded efficiently to furnish a series of indole-fused bridged [4.2.1] lactones in mostly good to excellent yields. For instance, various indolyl-substituted ynamides bearing both electron-donating and -withdrawing groups could be readily converted into the desired indole-fused bicyclic skeletons **2a–2p** with yields ranging from 60% to 86%. In particular, the functional groups, such as CF_3_, CN and CO_2_Me, were well tolerated under this Brønsted acid catalysis. Further investigation of N-protecting groups demonstrated that the Bs- and Ms-protected substrates **1q–1r** gave slightly improved yields. In addition, an ynamide with a methyl group (R^1^ = Me) was also a suitable substrate for this tandem reaction to afford the corresponding **2s** in 63% yield (dr: 4 : 1). This result clearly indicated that [3,3] rearrangement, but not [1,3] rearrangement,^17^ was presumably involved in this multiple cascade sequence.^13^ Furthermore, the reaction also occurred smoothly with various aryl- or methyl-substituted ynamides (R^2^ = aryl, Me), and the desired **2t–2z** containing a quaternary carbon center could be formed in 60–75% yields by employing 30 mol% of HNTf_2_ as the catalyst. Importantly, excellent diastereoselectivity (>20 : 1) was achieved in all cases except for the substrate **1s**. The molecular structures of **2a**, **2s** and **2u** were confirmed by X-ray diffraction.^18^ Thus, this metal-free protocol provides a highly convenient and practical route for the preparation of valuable bridged [4.2.1] lactones.

**Table 2:** Reaction scope for the construction of bridged [4.2.1] lactones **2**^*a*^

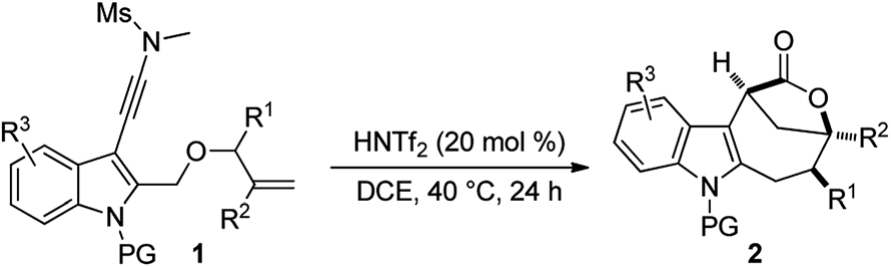
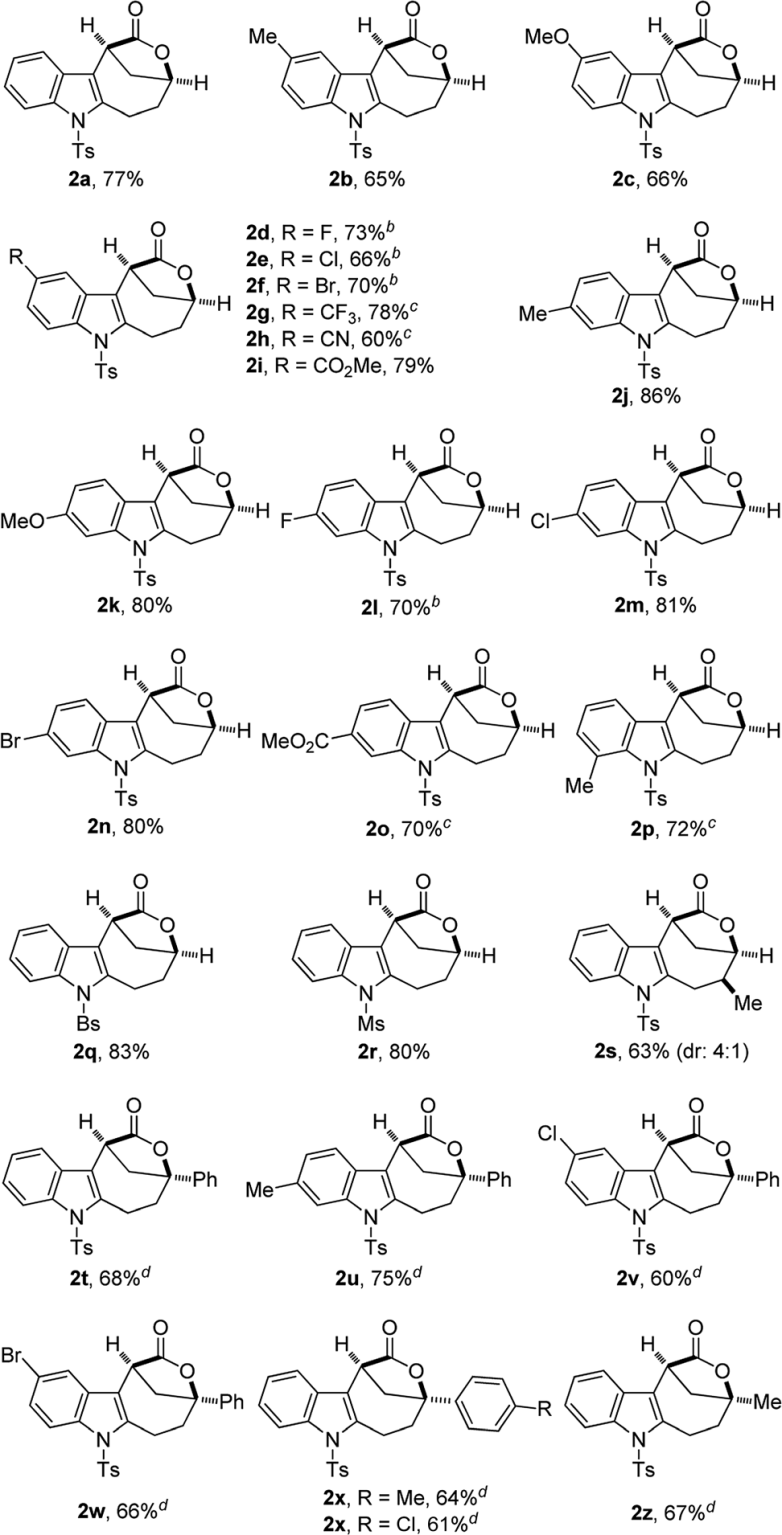

^*a*^Reaction conditions: **1** (0.2 mmol), HNTf_2_ (0.04 mmol), DCE (4 mL), 40 °C, in vials; yields are those for the isolated products.

^*b*^80 °C, 36 h.

^*c*^48 h.

^*d*^30 mol% of HNTf_2_ was used, 60 °C, 24 h.

In addition, this multiple cascade reaction was also applicable to other electron-rich aromatic ring-substituted ynamides such as benzofuran-, pyrrole-, and alkoxy arene-tethered ynamides **3a–3c**, delivering the desired bridged [4.2.1] lactones **4a–4c** in serviceable yields with excellent dr values (>20 : 1), as depicted in eqn (1)–(3). Of note, the use of HFIP as an additive led to a significantly improved yield in the case of **3c** (eqn (3)).^19^ Attempts to extend the reaction to non-terminal alkene-substituted ynamide **3d**, indole-linked ynamide-allyl amine **3e** and sulfide **3f** led to the formation of complicated mixtures.
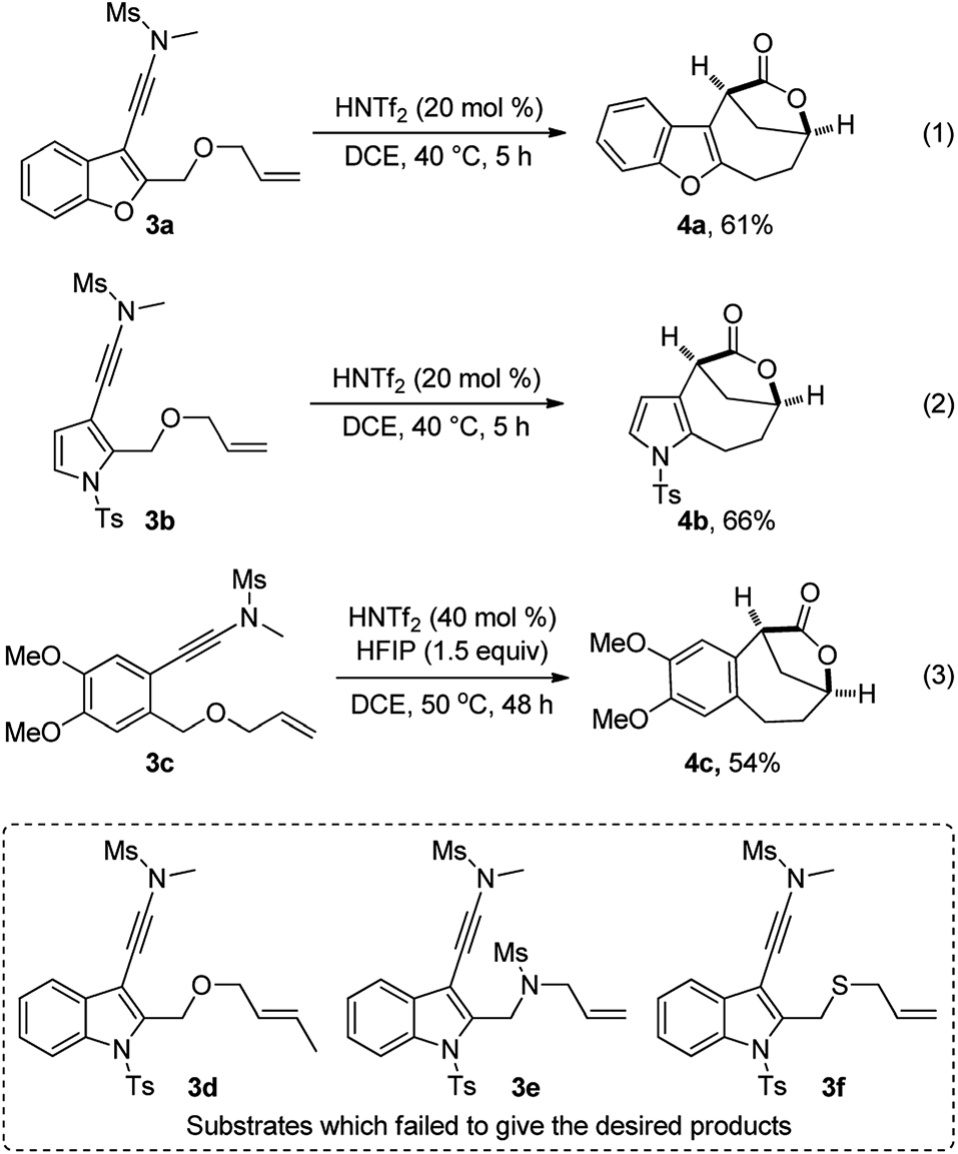


Although our attempts to employ various chiral Brønsted acids such as chiral phosphoric acids and chiral phosphoric amides to catalyze this cascade cyclization failed probably due to the fact that their acidity is not strong enough, the chiral bridged [4.2.1] lactones could be synthesized by employing a chiral oxazolidinone instead of *N*-methyl methanesulfonamide as the directing group.^20^ As shown in eqn (4), it was found that the *tert*-butyl substituted oxazolidinone-derived chiral ynamide **5d** gave the best enantioselectivity, and the desired chiral bridged [4.2.1] lactone **2a**-*ent* was formed in 55% yield with 95% ee in the presence of 30 mol% of HNTf_2_ as the catalyst and 1.5 equiv. of water as the additive. Thus, the chiral auxiliary can be regarded as a traceless directing group to introduce the chirality in a very facile manner. It should be mentioned that significant racemization was observed when prolonging the reaction time.^13^
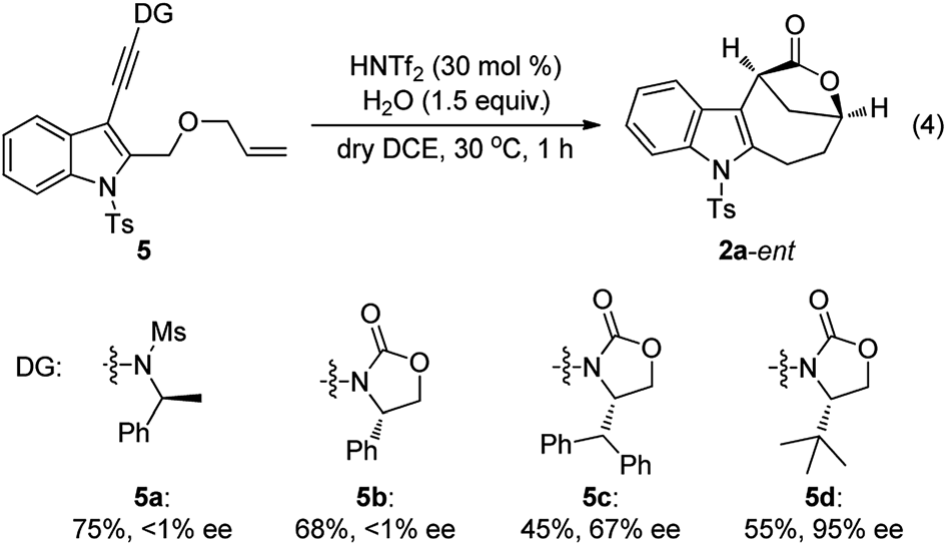


The scope of this asymmetric intramolecular alkoxylation-initiated tandem reaction was further examined by using *tert*-butyl substituted oxazolidinone-derived chiral ynamides. As depicted in Table 3, the reaction proceeded smoothly with various chiral ynamides **5**, allowing the facile synthesis of the corresponding enantioenriched bridged [4.2.1] lactones **2**-*ent* in serviceable yields with excellent ee values (91–99% ee) and excellent dr values (>20 : 1). Of note, (*R*)-*tert*-butyl substituted oxazolidinone-derived chiral ynamide **5d′** could also undergo smooth cascade cyclization to deliver the desired **2a**-*ent*′ with the opposite enantioselectivity.

**Table 3:** Reaction scope for the construction of chiral bridged [4.2.1] lactones **2**-*ent*^*a*^

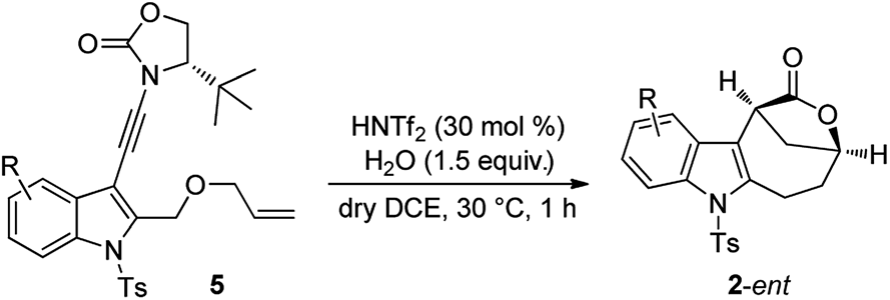
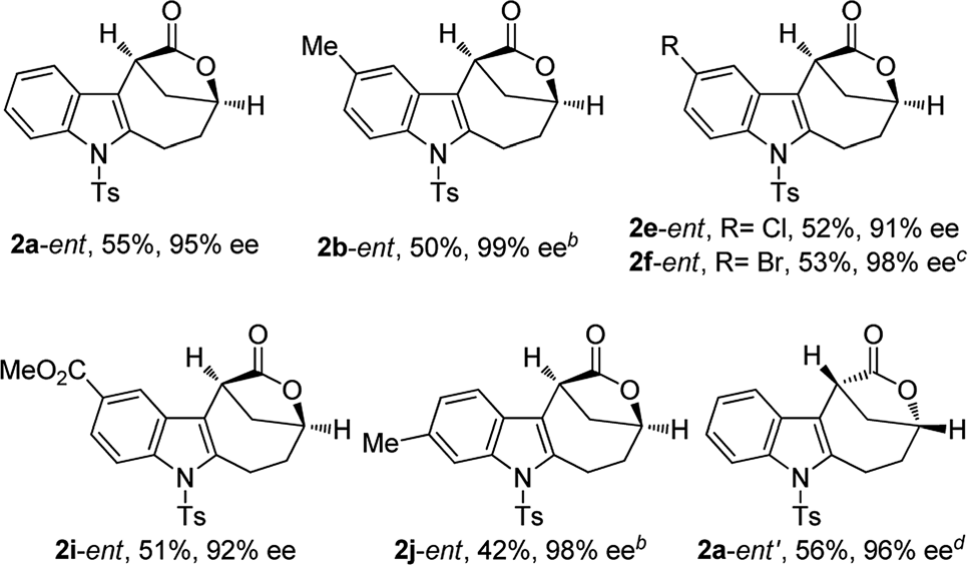

^*a*^Reaction conditions: **5** (0.2 mmol), HNTf_2_ (0.06 mmol), water (0.3 mmol), dry DCE (2 mL), 30 °C, in vials; yields are those for the isolated products; determined by HPLC analysis.

^*b*^40 °C, 20 min.

^*c*^30 min.

^*d*^Using (*R*)-configured ynamide **5d′** as the substrate.

**Scheme 2:**
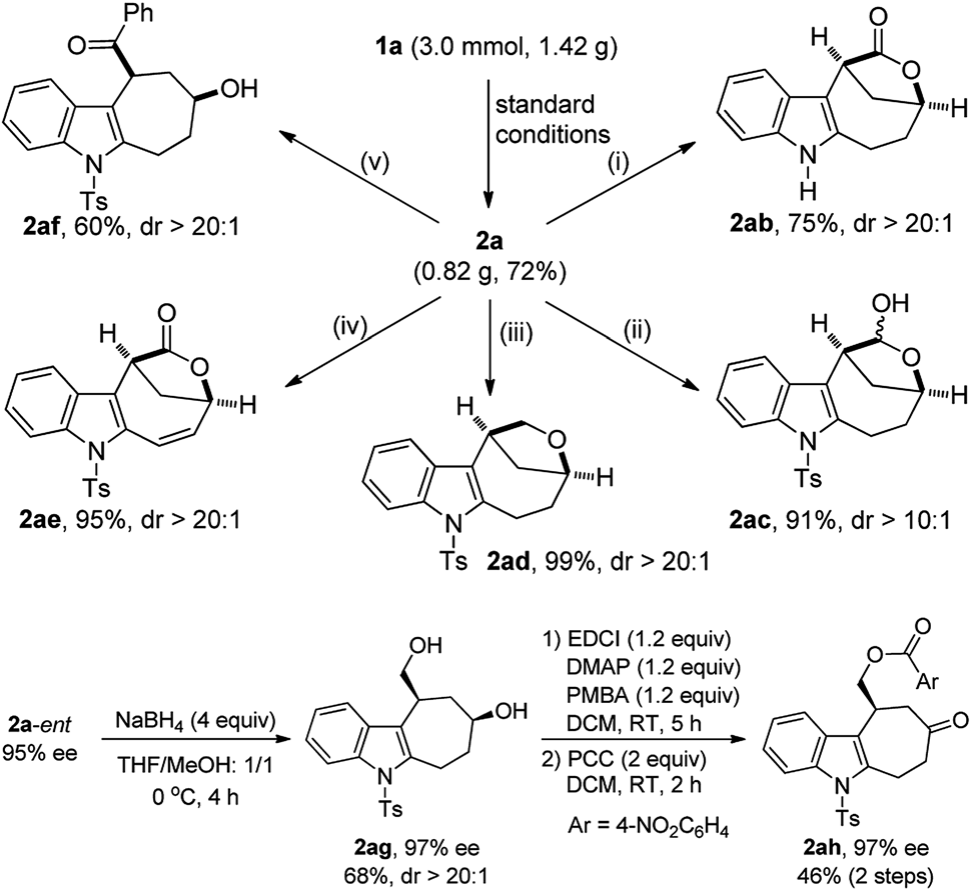
Gram scale reaction and synthetic applications. Reagents and conditions: (i) TBAF (4 equiv.), THF, 65 °C, 6 h; (ii) DIBAL-H (1.5 equiv.), THF, –78 °C, 6 h; (iii) InBr_3_ (0.5 equiv.), Et_3_SiH (2.5 equiv.), CHCl_3_, 60 °C, 10 h; (iv) DDQ (3 equiv.), DCE, 60 °C, 48 h; (v) PhLi (1.2 equiv.), THF, –40 °C, 2 h; –20 °C, 10 h.

The potential synthetic utility of this protocol was then demonstrated by the facile diversification of bridged lactone **2a**, and importantly, excellent diastereoselectivity was achieved in all cases (Scheme 2). For example, the Ts group in lactone **2a**, prepared on a gram scale in 72% yield, was easily removed by treatment with TBAF to afford the corresponding **2ab** in 75% yield. In addition, the lactone part of **2a** could be selectively reduced to furnish the hemiacetal **2ac** (91%, dr > 10 : 1) with DIBAL-H. By contrast, the use of Et_3_SiH led to the total reduction of the lactone to produce the desired **2ad** in almost quantitative yield. Interestingly, **2a** could also be oxidized with DDQ to deliver **2ae** in 95% yield. Moreover, the ring of lactone **2a** was readily opened by employing PhLi, leading to the stereocontrolled construction of cyclohepta[*b*]indole scaffold **2af**, frequently occurring in natural products and bioactive molecules.^21^ Finally, the chiral **2a**-*ent* could be further transformed into the indole-fused cycloheptanone **2ah** with the ee maintained, and its structure was confirmed by X-ray diffraction,^18^ which also determined the absolute configuration of **2a**-*ent*.^22^

Considering the bioactivity reported in the literature for bridged [4.2.1] lactone systems,^1^ we also tested the above synthesized indole-fused lactones for their biological activity as antitumor agents. The cytotoxic effects of these compounds were evaluated against a panel of cancer cells, including breast cancer cells MDA-MB-231 and MCF-7, melanoma cells A375, and esophageal cancer cells SK-GT-4 and KYSE-450 using cell viability assay.^13^ The results revealed that these compounds exhibited differential cytotoxicity and selectivity. Compounds **2k**, **2l**, and **2o** selectively inhibited the cell growth of A375 by more than 50% at a concentration of 20 μM, and compound **2af** inhibited the cell growth of MCF7 by around 70%. While compound **2ae** showed broad activity with a cell viability less than 50% against cancer cells MDA-MB-231, A375 and KYSE-450.

To understand the reaction mechanism, several control experiments were conducted. First, we performed deuterium labeling studies and found that no deuterium loss was observed, thus ruling out any possible reaction pathways involving a hydride shift (eqn (5)). Importantly, **2aa** was readily converted into **2a** in 95% yield in the presence of HNTf_2_ while no **2a** was formed without an acid catalyst,^13^ strongly supporting the notion that **2aa** is the key intermediate for this tandem reaction (eqn (6)). In addition, the formation of cyclohepta[*b*]indole **2ai** (dr > 20 : 1) was detected when **2aa** was treated with 10 equiv. of MeOH in the presence of HNTf_2_, which indicates that the cationic intermediate is presumably involved in such a tandem sequence (eqn (7)). Of note, **2a** could not be converted into **2ai** in the presence of HNTf_2_ and MeOH.^13^ Moreover, the cascade cyclization of ynamide **3g** or **3h** under the standard conditions only led to the formation of the corresponding **4ga** (42%) or **4ha** (85%), and no desired bridged lactone product was observed (eqn (8) and (9)). These results further suggest that the intramolecular alkene insertion into the C–O bond proceeds *via* a carbocation pathway, and the generation of a stable electron-rich benzylic carbocation is the key for this lactone expansion process. Finally, it was found that significant incorporation of ^18^O (>85%) into the product **2a** was observed in the presence of ^18^O-labelled water (10 equiv.), indicating that the oxygen atom on the carbonyl group of **2a** originates from water.^13^
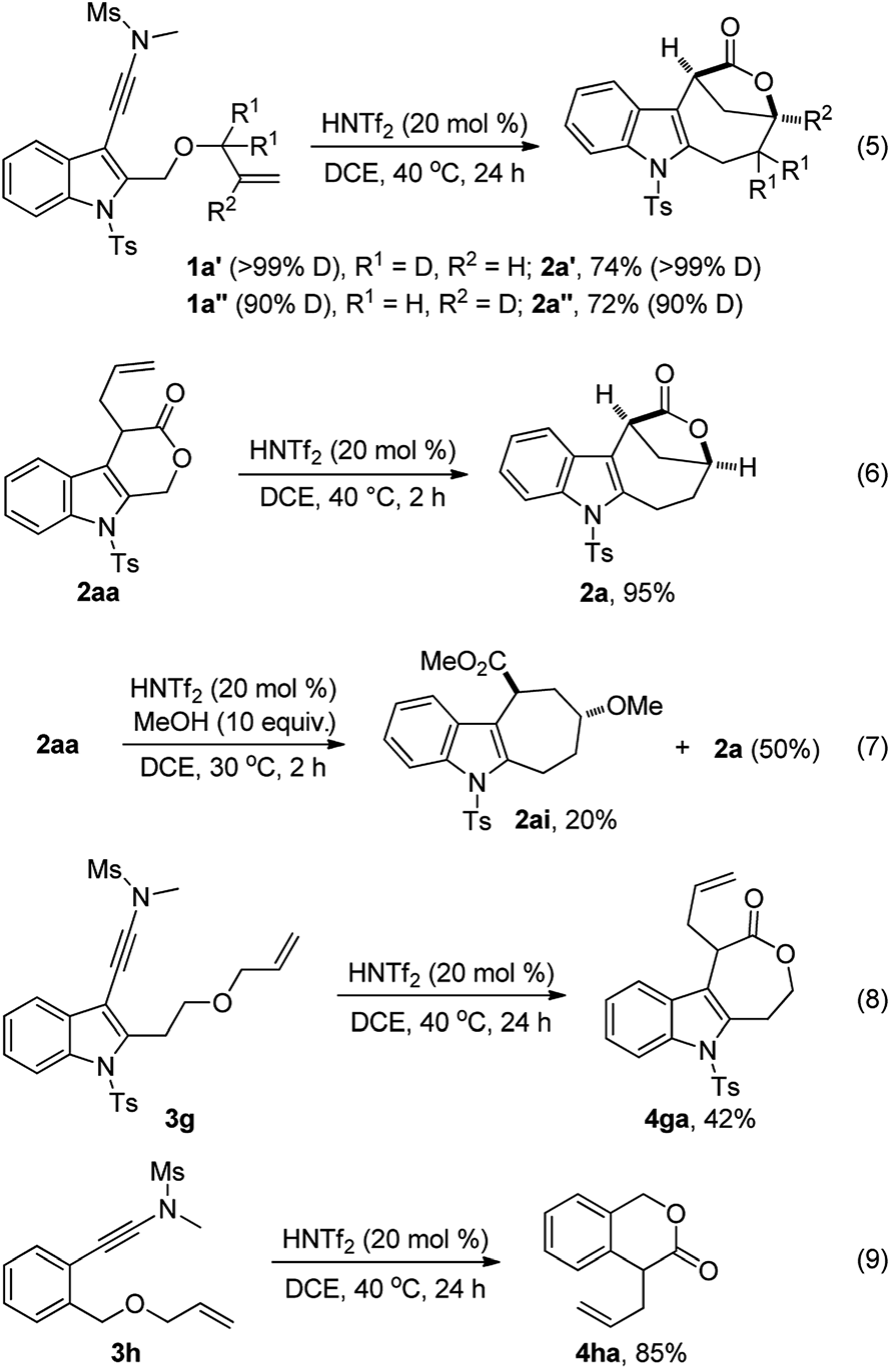


**Scheme 3:**
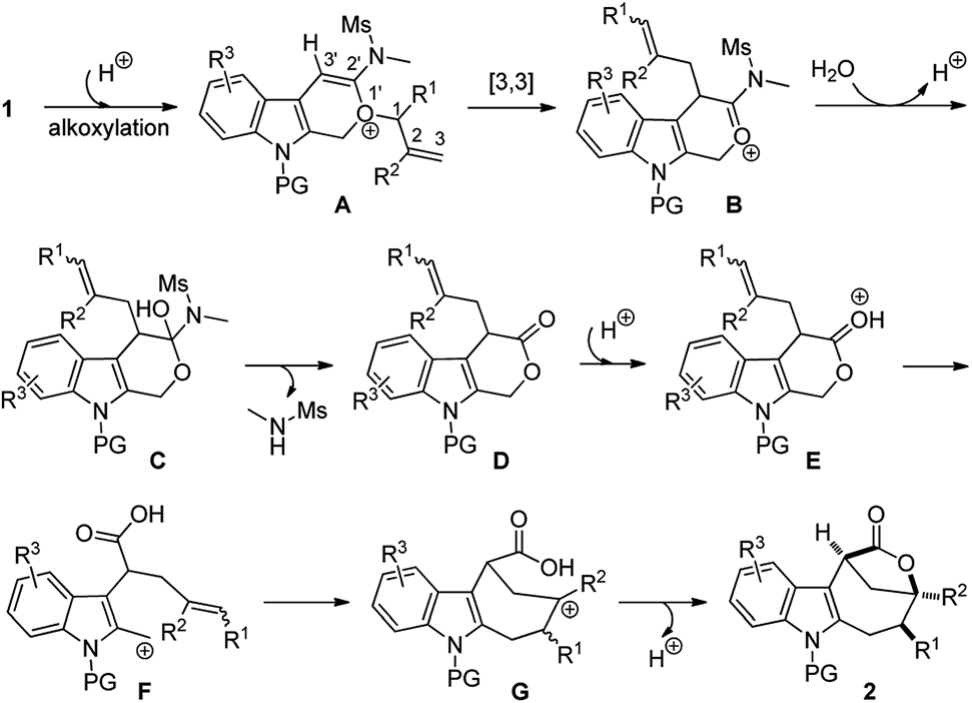
Plausible reaction mechanism.

Based on the above experimental observations, a plausible mechanism to rationalize the formation of indole-fused bridged [4.2.1] skeleton **2** is proposed (Scheme 3). Initially, the alkoxy group attacks the acid-activated ynamide **1** to form the Claisen rearrangement precursor **A***via* a keteniminium intermediate, which undergoes typical [3,3] rearrangement and subsequent trapping by trace water, delivering the indole-fused lactone **D**^23^ (that is, **2aa** in the case of substrate **1a**) along with the generation of sulfonamide. Brønsted acid further promotes the ring opening of lactone **D** to produce the carbocation intermediate **F***via* C–O bond cleavage. Finally, the carbocation of **F** is trapped by an intramolecular electron-rich alkenyl group to generate another carbocation intermediate **G**, which is further captured by the intramolecular carboxylic acid group to afford the final product **2**.^24^ Notably, the low nucleophilicity of Tf_2_N^–^ might be important to maintain the cationic nature and/or reactivity of certain intermediates involved.^15^

## Conclusions

In summary, we have developed a novel Brønsted acid-catalyzed intramolecular alkoxylation-initiated tandem sequence, which represents the first metal-free intramolecular alkoxylation/Claisen rearrangement to the best of our knowledge. Significantly, an unprecedented Brønsted acid-catalyzed intramolecular alkene insertion into the C–O bond *via* a carbocation pathway was discovered, which may serve as an alternative approach for alkene carbooxygenation *via* a direct C–O cleavage. This method enables efficient and stereocontrolled access to valuable indole-fused [4.2.1] lactones under mild reaction conditions, providing ready access to biologically relevant scaffolds in a single synthetic step from an acyclic precursor. Moreover, such an asymmetric cascade cyclization was also realized by employing a traceless chiral directing group. A mechanistic rationale for this novel tandem reaction is well supported by a variety of control experiments. In addition, our preliminary biological tests showed that some of these newly synthesized indole-fused lactones exhibited their bioactivity as antitumor agents against different breast cancer cells, melanoma cells, and esophageal cancer cells. Thus, we believe that this novel multiple cascade reaction will not only inspire chemists to design new rearrangement processes, but also encourage them to find their potential usefulness in organic and medicinal chemistry.

## Conflicts of interest

There are no conflicts to declare.

## Supplementary Material

Supplementary information

Crystal structure data
