# Supplementary material for: Metal-free alkene carbooxygenation following tandem intramolecular alkoxylation/Claisen rearrangement: stereocontrolled access to bridged [4.2.1] lactones
Source: Chem Sci. 2019 Jan 24;10(10):3123–9. doi: 10.1039/c9sc00079h (PMC6429610; doi:10.1039/c9sc00079h)
Supplement: Supplementary file 1 [file SC-010-C9SC00079H-s001.pdf]

Electronic Supplementary Material (ESI) for Chemical Science.
This journal is © The Royal Society of Chemistry 2019

Supporting Information

Metal-Free Alkene Carbooxygenation Following Tandem Intramolecular Alkoxylation/Claisen Rearrangement: Stereospecific Access to Bridged [4.2.1] Lactones

Long Li, Xin-Qi Zhu, Ying-Qi Zhang, Hao-Zhen Bu, Peng Yuan, Jinyu Chen, Jingyi Su, Xianming Deng and Long-Wu Ye*

State Key Laboratory of Physical Chemistry of Solid Surfaces and Key Laboratory for Chemical Biology of Fujian Province, College of Chemistry and Chemical Engineering, Xiamen University, Xiamen 361005, China

E-mail: longwuye@xmu.edu.cn

Content	Page Number
**General**	2
**More Reaction Condition, Scope and Mechanism Studies**	3
**Biological Tests**	6
**Preparation of Starting Materials**	7
**General Procedure: Brønsted Acid Catalysis**	36
**Crystal Data**	64
**HPLC Chromatograms**	68
**^{1}H and ^{13}C NMR Spectra**	77

General Information. Ethyl acetate (ACS grade), hexanes (ACS grade) and anhydrous 1,2-dichloroethane (ACS grade) were obtained commercially and used without further purification. Methylene chloride, tetrahydrofuran and diethyl ether were purified according to standard methods unless otherwise noted. Commercially available reagents were used without further purification. Ees are determined using HPLC on a chiral stationary phase. Reactions were monitored by thin layer chromatography (TLC) using silicycle pre-coated silica gel plates. Flash column chromatography was performed over silica gel (300-400 mesh). Infrared spectra were recorded on a Nicolet AVATER FTIR330 spectrometer as thin film and are reported in reciprocal centimeter (cm^{-1}). Mass spectra were recorded with Micromass QTOF2 Quadrupole/Time-of-Flight Tandem mass spectrometer using electron spray ionization.

^{1}H NMR spectra were recorded on a Bruker AV-400 spectrometer and a Bruker AV-500 spectrometer in chloroform-d_3. Chemical shifts are reported in ppm with the internal TMS signal at 0.0 ppm as a standard. The data is being reported as (s = singlet, d = doublet, t = triplet, m = multiplet or unresolved, brs = broad singlet, coupling constant(s) in Hz, integration).

^{13}C NMR spectra were recorded on a Bruker AV-400 spectrometer and a Bruker AV-500 spectrometer in chloroform-d_3. Chemical shifts are reported in ppm with the internal chloroform signal at 77.0 ppm as a standard.

More Reaction Condition, Scope and Mechanism Studies

1. Other reaction condition studies on the cascade cyclization of indolyl ynamide **1a**[a]

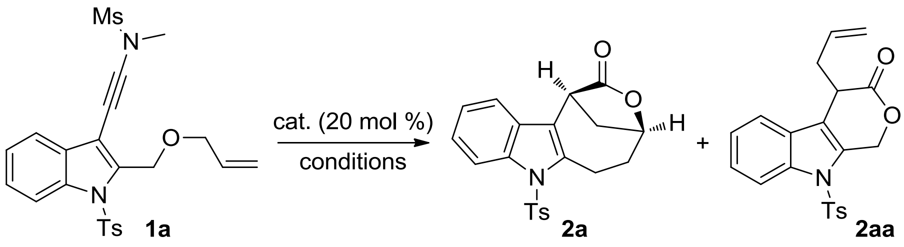

Entry	Catalyst	Reaction conditions	Yield[b] (%) 2a	2aa	1a
1	AgOTf	DCE, 40 °C, 48 h	<1	30	50
2	$AgNTf_2$	DCE, 40 °C, 48 h	<1	<1	>95
3	CF_3CO_2H	DCE, 40 °C, 48 h	<1	<1	<1
4	MsOH	DCE, 40 °C, 48 h	<1	<1	<1
5	TsOH	DCE, 40 °C, 48 h	<1	<1	<1
6	$HNTf_2$	toluene, 40 °C, 48 h	28	26	<1
7	$HNTf_2$	PhCl, 40 °C, 48 h	69	<1	<1
8	$HNTf_2$	THF, 40 °C, 48 h	<1	<1	<1
9	$HNTf_2$	CH_3CN, 40 °C, 48 h	44	12	<1
10[c]	$HNTf_2$	DCE, 40 °C, 24 h	80	<1	<1
11[d]	$HNTf_2$	DCE, 40 °C, 24 h	74	<1	<1

[a] Reaction conditions: **1a** (0.1 mmol), catalyst (20 mol %), solvent (2 mL), 40 °C, in vials. [b] Measured by ^1H NMR using diethyl phthalate as the internal standard. [c] The reaction was performed in a flame-dried vial with dry DCE as the solvent and 1 equiv of H_2O as an additive. [d] The reaction was performed in a flame-dried vial with dry DCE as the solvent and 2 equiv of H_2O as an additive.

2. Almost no desired product was formed by employing substrates with other PG groups such as Me, Boc, Ac and benzyl, as these kinds of substrates are too reactive and may undergo many side reactions such as hydration reaction, dimerization reaction and decomposition under the acidic conditions.

3. [3,3] rearrangement *vs* [1,3] rearrangement:

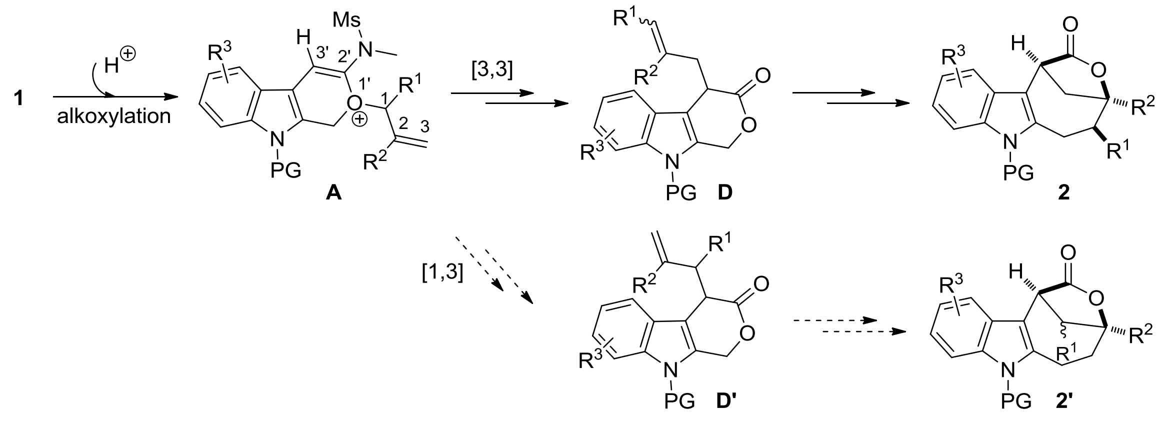

4. We tried various chiral Brønsted acids such as chiral phosphoric acids and chiral phosphoric amides (see as followed), but found that they failed to catalyze this cascade cyclization (most of substrates were recovered).

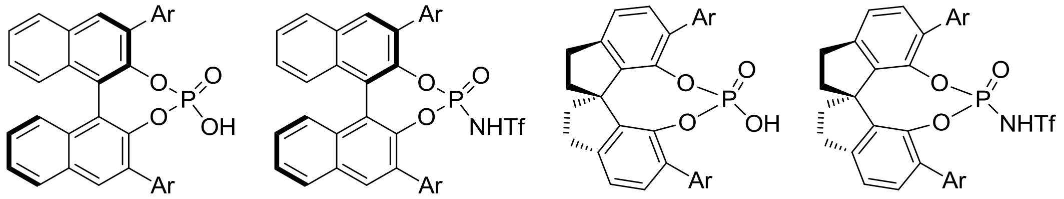

5. Notably, after the reaction was finished (1 h), significant epimerization was observed if prolonging the reaction time.

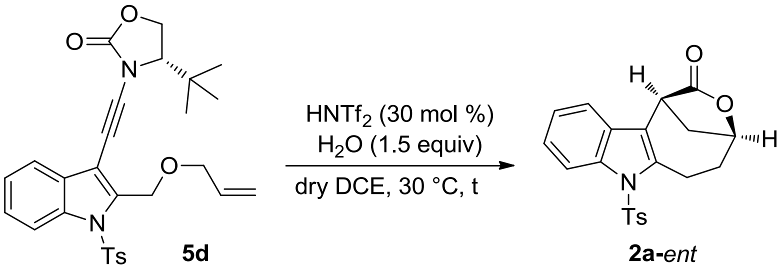

t/h	1	2	4	6	8	10	12	14
ee%	95	88	71	60	50	35	15	0

6. A possible molecular model of transition state to explain the chirality induction (**2a** as an example) has been proposed. Notably, the indole moiety has to occupy the axial

position but not the equatorial position as the indolyl group and oxygen are on the same side of the double bond. In addition, the second chiral center of product **2a** should be induced by the first chiral center (*cis* configuration).

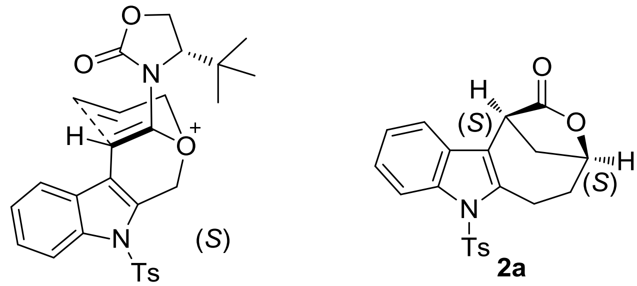

7. For the effects of other metal catalysts on catalyzing the convertion **2aa** into **2a**, see:

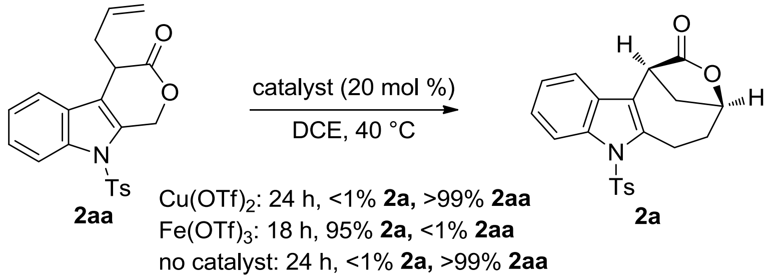

8. **2a** could not be converted into **2ai** in the presence of $HNTf_2$ and MeOH, and only **2a** was recoveried. This result indicated that **2ai** should come from **2aa** but not **2a**.

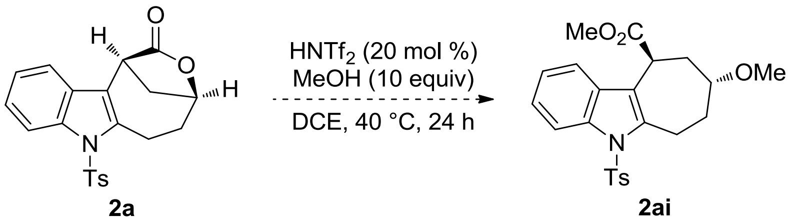

9. Significant incorporation of ^{18}O (>85%) into the product **2a** was observed in the presence of ^{18}O-labelled water (10 equiv), indicating that the oxygen atom on the carbonyl group of **2a** originates from water.

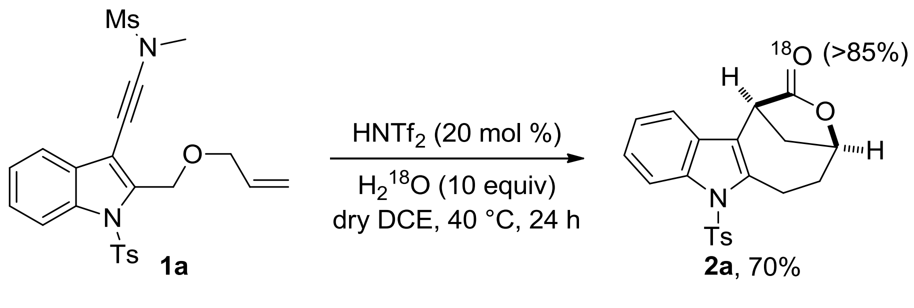

Cell Viability Assay.

The cytotoxic effects of the indole-fused lactone compounds on human cancer cells (MDA-MB-231, A375, MCF-7, SK-GT-4 and KYSE-450) were investigated using a commercially available proliferation assay kit (Promega, US). Briefly, the cells were plated in 96-well culture plates at an appropriate density in culture medium and allowed to attach overnight. After treatment of vehicle (0.1% DMSO as control) or test compounds for indicated times and concentrations, 20 μL of MTS reaction solution (3-(4, 5-dimethylthiazol-2-yl)-5-(3-carboxymethoxyphenyl)-2- (4-sulfophenyl)-2H-tetrazolium, inner salt; MTS (a) and 100 μg/mL phenazine methosulfate; PES) was added to each well. The absorbance values were read at 490 nm wavelength with a spectrophotometer (Varioskan Flash, Thermo, US) after 1 to 4 hours incubation. The cell viability was calculated as: cell survival = (ODcompd. - ODblank)/(ODcontrol - ODblank)*100%.

Table S1. The cytotoxic effects of the newly synthesized indole-fused lactone compounds against cancer cells

Cmpd ID	Cell viability at 20 μM (%)				
	MDA-MB-231	A375	MCF-7	SK-GT-4	KYSE-450
2a	99.49	82.26	105.05	94.43	92.88
2b	100.43	87.91	109.52	93.46	85.22
2c	88.17	85.78	126.55	90.16	84.49
2d	90.54	95.18	127.82	91.23	80.40
2e	94.13	86.68	89.27	93.19	87.92
2f	98.84	86.09	124.54	90.07	80.71
2g	95.33	78.72	95.25	91.62	75.82
2h	114.83	99.59	92.34	101.12	83.63
2i	105.53	109.08	99.99	96.41	82.66
2j	112.04	92.08	71.80	102.95	79.56
2k	56.71	40.50	98.57	72.27	76.90
2l	54.71	48.00	108.09	93.58	75.83
2m	86.96	87.76	121.30	93.12	97.55
2n	107.95	103.52	112.91	102.15	101.09
2o	56.27	45.57	66.78	91.57	88.43
2t	95.42	86.75	119.05	97.59	99.96
2z	93.06	94.49	100.37	96.98	91.59
2ab	91.08	102.28	113.21	95.11	84.87
2ae	41.59	33.41	70.16	64.00	37.01
2af	91.12	96.02	32.77	91.72	88.98
2ah	88.27	91.21	103.63	91.85	90.31

Results are average of two experiments.

Experimental Section

Representative synthetic procedures for the preparation of indole-tethered ynamides 1:[1-3]

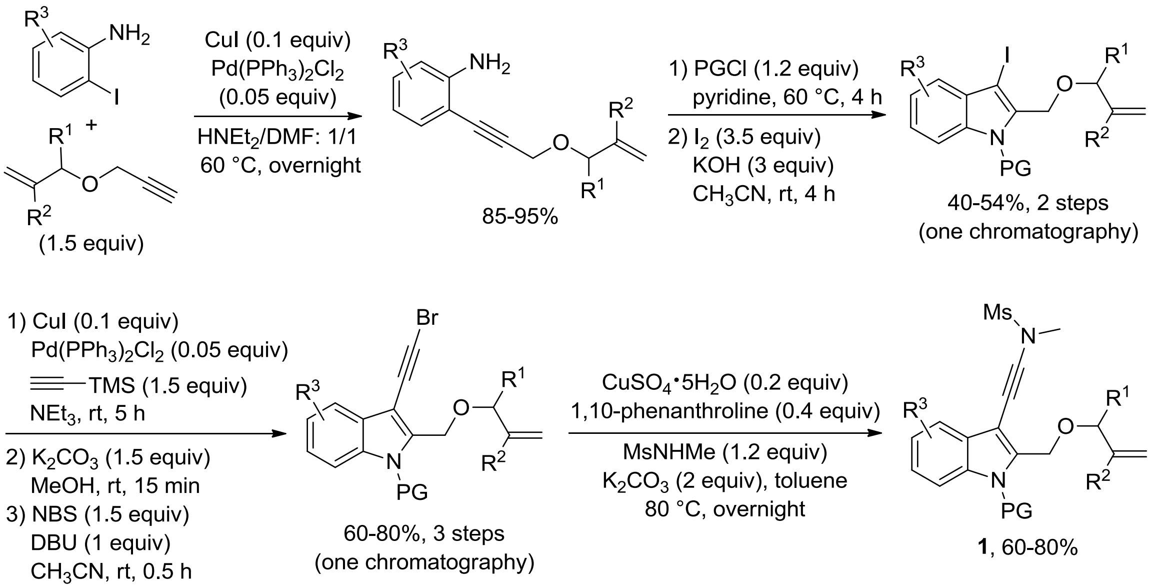

N-((2-((allyloxy)methyl)-1-tosyl-1*H*-indol-3-yl)ethynyl)-*N*-methylmethanesulfonamide (1a)

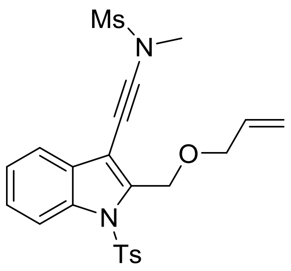

1a

Pale yellow solid (mp 110-111 °C). ^{1}H NMR (400 MHz, $CDCl_3$) δ 8.12 (d, *J* = 8.4 Hz, 1H), 7.94 (d, *J* = 8.4 Hz, 2H), 7.58 (d, *J* = 7.6 Hz, 1H), 7.44 – 7.32 (m, 1H), 7.32 – 7.24 (m, 1H), 7.18 (d, *J* = 8.0 Hz, 2H), 6.00 – 5.87 (m, 1H), 5.38 –5.27 (m, 1H), 5.21 (dd, *J* = 10.4, 1.2 Hz, 1H), 5.02 (s, 2H), 4.06 (dt, *J* = 5.6, 1.2 Hz, 2H), 3.33 (s, 3H), 3.14 (s, 3H), 2.33 (s, 3H); ^{13}C NMR (125 MHz, $CDCl_3$) δ 145.0, 139.3, 135.8, 135.7, 134.6, 129.6, 129.1, 127.4, 126.0, 123.8, 120.2, 117.2, 114.7, 107.5, 89.2, 71.2, 62.6, 60.7, 39.3, 37.0, 21.5; IR (neat): 2923, 2850, 2239(s), 1357(s), 1160, 1108, 576; HRESIMS Calcd for $[C_{23}H_{24}N_2NaO_5S_2]^+$ (M + Na^+) 495.1019, found 495.1008.

N-((2-((allyloxy)methyl)-5-methyl-1-tosyl-1H-indol-3-yl)ethynyl)-N-methylmethanesulfonamide (1b)

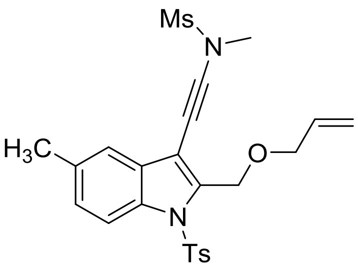

1b

Pale yellow solid (mp 115-116 °C). ^{1}H NMR (400 MHz, $CDCl_3$) δ 7.99 (d, *J* = 8.8 Hz, 1H), 7.91 (d, *J* = 8.0 Hz, 2H), 7.35 (s, 1H), 7.22 – 7.12 (m, 3H), 6.03 – 5.81 (m, 1H), 5.30 (d, *J* = 17.2 Hz, 1H), 5.19 (d, *J* = 10.4 Hz, 1H), 4.99 (s, 2H), 4.05 (d, *J* = 4.0 Hz, 2H), 3.34 (s, 3H), 3.14 (s, 3H), 2.42 (s, 3H), 2.32 (s, 3H); ^{13}C NMR (100 MHz, $CDCl_3$) δ 144.8, 139.4, 135.8, 134.6, 134.1, 133.6, 129.5, 129.3, 127.4, 127.3, 120.0, 117.1, 114.4, 107.3, 89.2, 71.1, 62.6, 60.9, 39.3, 37.0, 21.5, 21.2; IR (neat): 2924, 2850, 1774, 1612, 1491, 1362(s), 1147, 584; HRESIMS Calcd for $[C_{24}H_{26}N_2NaO_5S_2]^+$ (M + Na^+) 509.1175, found 509.1168.

N-((2-((allyloxy)methyl)-5-methoxy-1-tosyl-1H-indol-3-yl)ethynyl)-N-methylmethanesulfonamide (1c)

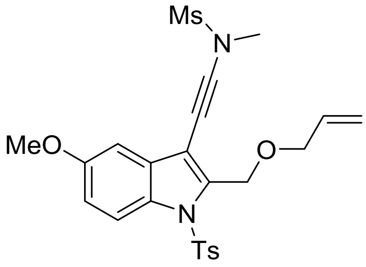

1c

Pale yellow oil. ^{1}H NMR (400 MHz, $CDCl_3$) δ 8.00 (d, *J* = 9.2 Hz, 1H), 7.88 (d, *J* = 8.4 Hz, 2H), 7.16 (d, *J* = 8.0 Hz, 2H), 7.00 (d, *J* = 2.4 Hz, 1H), 6.96 (dd, *J* = 9.2, 2.8 Hz, 1H), 6.00 – 5.85 (m, 1H), 5.36 – 5.26 (m, 1H), 5.24 – 5.16 (m, 1H), 4.97 (s, 2H), 4.05 (dt, *J* = 5.6, 1.6 Hz, 2H), 3.83 (s, 3H), 3.33 (s, 3H), 3.13 (s, 3H), 2.31 (s, 3H); ^{13}C NMR (100 MHz, $CDCl_3$) δ 156.8, 144.9, 139.6, 135.5, 134.5, 130.3, 130.2, 129.5, 127.2, 117.0,

115.6, 115.0, 107.5, 102.3, 89.4, 71.1, 62.5, 60.7, 55.6, 39.2, 36.9, 21.4; IR (neat): 2923, 2850, 2240(s), 1359(s), 1172, 1109, 999, 766; HRESIMS Calcd for $[C_{24}H_{26}N_2NaO_6S_2]^+$ (M + Na^+) 525.1124, found 525.1122.

N-((2-((allyloxy)methyl)-5-fluoro-1-tosyl-1*H*-indol-3-yl)ethynyl)-*N*-methylmethanesulfonamide (1d)

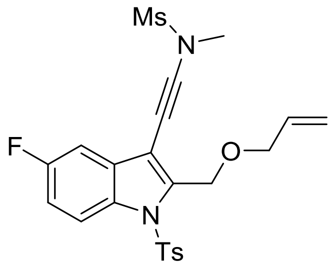

1d

Pale yellow solid (mp 115-116 °C). ^{1}H NMR (400 MHz, $CDCl_3$) δ 8.07 (dd, *J* = 9.2, 4.0 Hz, 1H), 7.90 (d, *J* = 8.4 Hz, 2H), 7.29 – 7.13 (m, 3H), 7.13 – 7.02 (m, 1H), 6.03 – 5.74 (m, 1H), 5.31 (dd, *J* = 17.6, 1.6 Hz, 1H), 5.21 (d, *J* = 10.4 Hz, 1H), 4.98 (s, 2H), 4.06 (d, *J* = 5.6 Hz, 2H), 3.33 (s, 3H), 3.13 (s, 3H), 2.32 (s, 3H); ^{13}C NMR (100 MHz, $CDCl_3$) δ 159.8 (d, *J* = 242.2 Hz), 145.2, 140.8, 135.3, 134.4, 132.0, 130.2 (d, *J* = 9.7 Hz), 129.6, 127.3, 117.2, 115.9 (d, *J* = 9.7 Hz), 113.9 (d, *J* = 25.3 Hz), 107.3 (d, *J* = 4.0 Hz), 105.7 (d, *J* = 24.3 Hz), 89.6, 71.2, 62.5, 60.0, 39.1, 37.0, 21.5; IR (neat): 2924, 2356(s), 1357(s), 1262, 1175, 760, 749; HRESIMS Calcd for $[C_{23}H_{23}FN_2NaO_5S_2]^+$ (M + Na^+) 513.0925, found 513.0909.

N-((2-((allyloxy)methyl)-5-chloro-1-tosyl-1*H*-indol-3-yl)ethynyl)-*N*-methylmethanesulfonamide (1e)

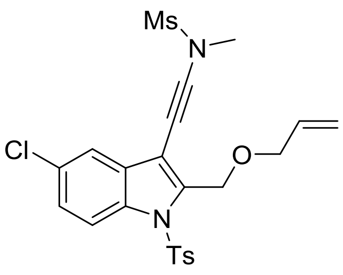

1e

Pale yellow oil. ^{1}H NMR (400 MHz, $CDCl_3$) δ 8.04 (d, *J* = 8.8 Hz, 1H), 7.90 (d, *J* = 8.4 Hz, 2H), 7.53 (d, *J* = 2.0 Hz, 1H), 7.36 – 7.28 (m, 1H), 7.19 (d, *J* = 8.4 Hz, 2H), 5.99 – 5.85 (m, 1H), 5.37 – 5.26 (m, 1H), 5.21 (dd, *J* = 10.4, 1.6 Hz, 1H), 4.98 (s, 2H), 4.05 (dt, *J* = 5.6, 1.2 Hz, 2H), 3.33 (s, 3H), 3.14 (s, 3H), 2.34 (s, 3H); ^{13}C NMR (100 MHz, $CDCl_3$) δ 145.3, 140.7, 135.4, 134.5, 134.2, 130.4, 129.8, 129.7, 127.4, 126.2, 119.8, 117.3, 115.9, 106.9, 89.6, 71.4, 62.5, 60.1, 39.3, 37.2, 21.6; IR (neat): 2926, 2238(s), 1360(s), 1265, 1175, 1111, 738; HRESIMS Calcd for $[C_{23}H_{23}ClN_2NaO_5S_2]^+$ (M + Na^+) 529.0629, found 529.0624.

_N_-((2-((allyloxy)methyl)-5-bromo-1-tosyl-1_H_-indol-3-yl)ethynyl)-_N_-methylmethanesulfonamide (1f)

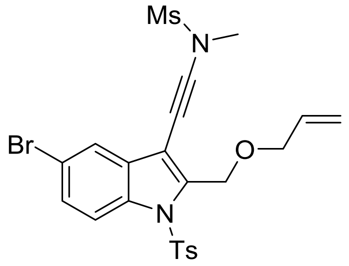

1f

Pale yellow solid (mp 130-131 °C). ^{1}H NMR (400 MHz, $CDCl_3$) δ 7.99 (d, *J* = 8.8 Hz, 1H), 7.90 (d, *J* = 8.4 Hz, 2H), 7.68 (d, *J* = 1.6 Hz, 1H), 7.44 (dd, *J* = 8.8, 2.0 Hz, 1H), 7.18 (d, *J* = 8.4 Hz, 2H), 6.03 – 5.78 (m, 1H), 5.31 (dd, *J* = 17.6, 1.6 Hz, 1H), 5.21 (d, *J* = 10.4 Hz, 1H), 4.98 (s, 2H), 4.05 (d, *J* = 5.2 Hz, 2H), 3.33 (s, 3H), 3.14 (s, 3H), 2.33 (s, 3H); ^{13}C NMR (100 MHz, $CDCl_3$) δ 145.3, 140.5, 135.3, 134.5, 134.4, 130.7, 129.7, 128.8, 127.3, 122.8, 117.3(4), 117.2(8), 116.1, 106.7, 89.5, 71.3, 62.4, 60.0, 39.2, 37.1, 21.5; IR (neat): 2927, 2240(s), 1444, 1360(s), 1175, 999, 688, 581; HRESIMS Calcd for $[C_{23}H_{23}BrN_2NaO_5S_2]^+$ (M + Na^+) 573.0124, found 573.0129.

_N_-((2-((allyloxy)methyl)-1-tosyl-5-(trifluoromethyl)-1_H_-indol-3-yl)ethynyl)-_N_-methylmethanesulfonamide (1g)

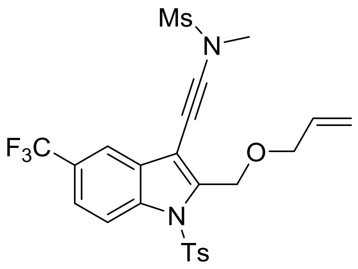

1g

Pale yellow solid (mp 150-151 ^{o}C). ^{1}H NMR (400 MHz, $CDCl_3$) δ 8.23 (d, *J* = 8.8 Hz, 1H), 7.95 (d, *J* = 8.4 Hz, 2H), 7.85 (s, 1H), 7.60 (d, *J* = 8.8 Hz, 1H), 7.21 (d, *J* = 8.4 Hz, 2H), 6.08 – 5.90 (m, 1H), 5.31 (dd, *J* = 17.2, 1.6 Hz, 1H), 5.22 (d, *J* = 10.4 Hz, 1H), 5.03 (s, 2H), 4.06 (d, *J* = 5.6 Hz, 2H), 3.35 (s, 3H), 3.15 (s, 3H), 2.35 (s, 3H); ^{13}C NMR (125 MHz, $CDCl_3$) δ 145.5, 141.2, 137.2, 135.2, 134.3, 129.8, 128.8, 127.4, 126.2 (q, *J* = 32.5 Hz), 124.3 (q, *J* = 270.0 Hz), 122.5 (q, *J* = 3.5 Hz), 117.8 (q, *J* = 4.0 Hz), 117.4, 115.0, 107.4, 89.8, 71.3, 62.4, 59.8, 39.2, 37.2, 21.5; IR (neat): 2927, 2241(s), 1361, 1320(s), 1170, 1117, 665, 592; HRESIMS Calcd for $[C_{24}H_{23}F_3N_2NaO_5S_2]^+$ (M + Na^+) 563.0893, found 563.0888.

_N_-((2-((allyloxy)methyl)-5-cyano-1-tosyl-1_H_-indol-3-yl)ethynyl)-_N_-methylmethanesulfonamide (1h)

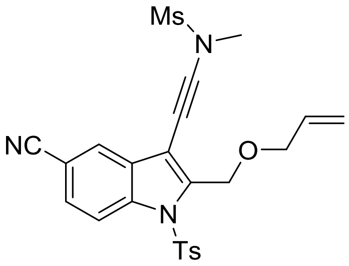

1h

Pale yellow oil. ^{1}H NMR (400 MHz, $CDCl_3$) δ 8.21 (dd, *J* = 8.8, 0.8 Hz, 1H), 8.02 – 7.82 (m, 3H), 7.60 (dd, *J* = 8.8, 1.6 Hz, 1H), 7.22 (d, *J* = 8.4 Hz, 2H), 5.98 – 5.87 (m, 1H), 5.36 – 5.27 (m, 1H), 5.26 – 5.17 (m, 1H), 5.01 (s, 2H), 4.06 (dt, *J* = 5.6, 1.2 Hz, 2H), 3.35 (s, 3H), 3.15 (s, 3H), 2.36 (s, 3H); ^{13}C NMR (100 MHz, $CDCl_3$) δ 145.7, 141.5, 137.4, 135.1, 134.2, 129.8, 129.2, 128.7, 127.5, 125.1, 118.9, 117.4, 115.5, 107.4, 107.0, 90.1, 71.4, 62.3, 59.4, 39.1, 37.3, 21.5; IR (neat): 2922, 2851, 2358, 2238(s), 1659, 1632,

1361(s), 1176, 737; HRESIMS Calcd for $[C_{24}H_{23}N_3NaO_5S_2]^+$ (M + Na^+) 520.0971, found 520.0972.

methyl 2-((allyloxy)methyl)-3-((*N*-methylmethylsulfonamido)ethynyl)-1-tosyl-1*H*-indole-5-carboxylate (1i)

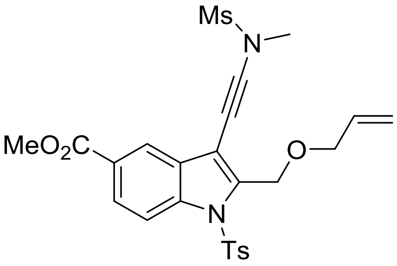

1i

Pale yellow oil. ^{1}H NMR (400 MHz, $CDCl_3$) δ 8.26 (d, *J* = 1.2 Hz, 1H), 8.16 (d, *J* = 8.8 Hz, 1H), 8.03 (dd, *J* = 8.8, 1.6 Hz, 1H), 7.93 (d, *J* = 8.4 Hz, 2H), 7.19 (d, *J* = 8.2 Hz, 2H), 5.85 – 5.98 (m, 1H), 5.36 – 5.27 (m, 1H), 5.20 (dd, *J* = 10.4, 1.2 Hz, 1H), 5.01 (s, 2H), 4.06 (dd, *J* = 4.4, 1.2 Hz, 2H), 3.93 (s, 3H), 3.35 (s, 3H), 3.15 (s, 3H), 2.33 (s, 3H); ^{13}C NMR (100 MHz, $CDCl_3$) δ 166.8, 145.3, 140.8, 138.2, 135.2, 134.3, 129.6, 128.8, 127.4, 126.9, 125.8, 122.3, 117.2, 114.4, 107.7, 89.6, 71.2, 62.4, 59.9, 52.1, 39.2, 37.0, 21.5; IR (neat): 2923, 2367, 2240(s), 1717(s), 1358(s), 1289, 1174, 758; HRESIMS Calcd for $[C_{25}H_{26}N_2NaO_7S_2]^+$ (M + Na^+) 553.1074, found 553.1075.

***N*-((2-((allyloxy)methyl)-6-methyl-1-tosyl-1*H*-indol-3-yl)ethynyl)-*N*-methylmethanesulfonamide (1j)**

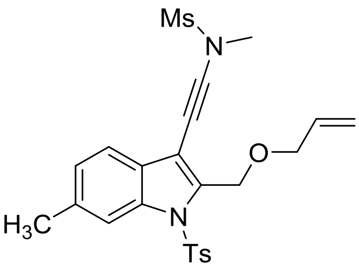

1j

Pale yellow oil. ^{1}H NMR (400 MHz, $CDCl_3$) δ 7.95 – 7.87 (m, 3H), 7.45 (d, *J* = 8.0 Hz, 1H), 7.18 (d, *J* = 8.0 Hz, 2H), 7.10 (dd, *J* = 8.0, 0.8 Hz, 1H), 5.99 – 5.84 (m, 1H), 5.36 – 5.26 (m, 1H), 5.24 – 5.15 (m, 1H), 4.99 (s, 2H), 4.03 (dt, *J* = 5.6, 1.2 Hz, 2H), 3.33 (s,

3H), 3.13 (s, 3H), 2.49 (s, 3H), 2.34 (s, 3H); ^{13}C NMR (100 MHz, $CDCl_3$) δ 144.8, 138.6, 136.2, 135.7, 134.5, 129.5, 127.2, 126.8, 125.3, 119.7, 117.0, 114.6, 107.5, 89.1, 71.0, 62.6, 60.8, 39.2, 36.9, 22.1, 21.5; IR (neat): 2927, 2241(s), 1361, 1326(s), 1176, 1117, 665, 592; HRESIMS Calcd for $[C_{24}H_{26}N_2NaO_5S_2]^+$ (M + Na^+) 509.1175, found 509.1173.

N-((2-((allyloxy)methyl)-6-methoxy-1-tosyl-1*H*-indol-3-yl)ethynyl)-*N*-methylmethanesulfonamide (1k)

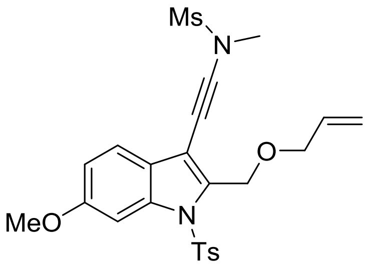

1k

Pale yellow oil. ^{1}H NMR (500 MHz, $CDCl_3$) δ 7.91 (d, *J* = 8.0 Hz, 2H), 7.65 (d, *J* = 2.0 Hz, 1H), 7.44 (d, *J* = 9.0 Hz, 1H), 7.18 (d, *J* = 8.0 Hz, 2H), 6.90 (dd, *J* = 8.5, 2.0Hz, 1H), 5.99 – 5.83 (m, 1H), 5.34 – 5.26 (m, 1H), 5.19 (dd, *J* = 5.5, 1.5 Hz, 1H), 4.96 (s, 2H), 4.04 (dt, *J* = 5.5, 1.5 Hz, 2H), 3.88 (s, 3H), 3.32 (s, 3H), 3.13 (s, 3H), 2.33 (s, 3H); ^{13}C NMR (125MHz, $CDCl_3$) δ 158.9, 144.9, 138.1, 136.9, 135.7, 134.6, 129.6, 127.3, 122.8, 120.7, 117.0, 113.0, 107.5, 99.0, 89.1, 71.0, 62.7, 60.9, 55.8, 39.2, 36.9, 21.5; IR (neat): 2927, 2240(s), 1445, 1360(s), 1176, 960, 582; HRESIMS Calcd for $[C_{24}H_{26}N_2NaO_6S_2]^+$ (M + Na^+) 525.1124, found 525.1118.

***N*-((2-((allyloxy)methyl)-6-fluoro-1-tosyl-1*H*-indol-3-yl)ethynyl)-*N*-methylmethanesulfonamide (1l)**

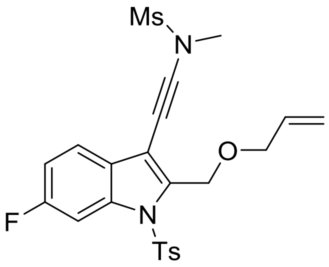

1l

Pale yellow oil. ^{1}H NMR (400 MHz, $CDCl_3$) δ 7.92 (d, *J* = 8.4 Hz, 2H), 7.84 (dd, *J* = 10.0, 2.0 Hz, 1H), 7.50 (dd, *J* = 8.4, 5.2 Hz, 1H), 7.19 (d, *J* = 8.4 Hz, 2H), 7.11 – 6.86 (m, 1H), 6.01 – 5.80 (m, 1H), 5.35 – 5.27 (m, 1H), 5.24 – 5.17 (m, 1H), 4.96 (s, 2H), 4.04 (dt, *J* = 5.6, 1.2 Hz, 2H), 3.32 (s, 3H), 3.13 (s, 3H), 2.32 (s, 3H); ^{13}C NMR (100 MHz, $CDCl_3$) δ 161.4 (d, *J* = 242.7 Hz), 145.2, 139.4 (d, *J* = 3.8 Hz), 135.8 (d, *J* = 12.7 Hz), 135.2, 134.4, 129.6, 127.3, 125.2, 121.1 (d, *J* = 10.0 Hz), 117.1, 112.2 (d, *J* = 24.4 Hz), 107.2, 102.0 (d, *J* = 29.3 Hz), 89.4, 71.0, 62.4, 60.2, 39.1, 36.9, 21.4; IR (neat): 2924, 2356(s), 1357(s), 1274, 1262, 1175, 764, 749; HRESIMS Calcd for $[C_{23}H_{23}FN_2NaO_5S_2]^+$ (M + Na^+) 513.0925, found 513.0923.

N-((2-((allyloxy)methyl)-6-chloro-1-tosyl-1*H*-indol-3-yl)ethynyl)-*N*-methylmethanesulfonamide (1m)

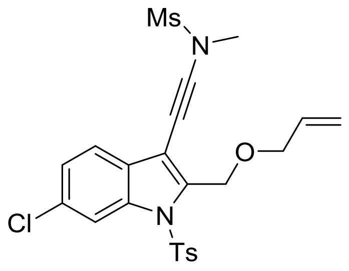

1m

Pale yellow solid (mp 110-111 °C). ^{1}H NMR (400 MHz, $CDCl_3$) δ 8.04 (d, *J* = 8.8 Hz, 1H), 7.90 (d, *J* = 8.4 Hz, 2H), 7.53 (d, *J* = 2.0 Hz, 1H), 7.31 (dd, *J* = 9.2, 2.0 Hz, 1H), 7.19 (d, *J* = 8.4 Hz, 2H), 6.02 – 5.83 (m, 1H), 5.31 (dd, *J* = 17.2, 1.2 Hz, 1H), 5.21 (dd, *J* = 10.4, 1.2 Hz, 1H), 4.98 (s, 2H), 4.05 (d, *J*= 5.6 Hz, 2H), 3.33 (s, 3H), 3.14 (s, 3H), 2.34 (s, 3H); ^{13}C NMR (100 MHz, $CDCl_3$) δ 145.3, 140.7, 135.4, 134.4, 134.1, 130.3, 129.8, 129.7, 127.3, 126.2, 119.8, 117.3, 115.8, 106.8, 89.5, 71.3, 62.5, 60.0, 39.2, 37.1, 21.5; IR (neat): 2926, 2238(s), 1445, 1360(s), 1265, 1175, 1111, 1000, 738, 583; HRESIMS Calcd for $[C_{23}H_{23}ClN_2NaO_5S_2]^+$ (M + Na^+) 529.0629, found 529.0629.

***N*-((2-((allyloxy)methyl)-6-bromo-1-tosyl-1*H*-indol-3-yl)ethynyl)-*N*-methylmethanesulfonamide (1n)**

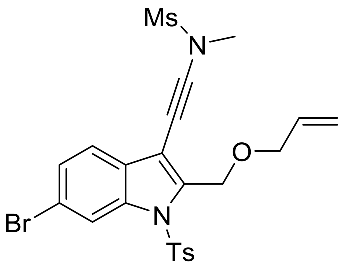

1n

Pale yellow oil. ^{1}H NMR (400 MHz, $CDCl_3$) δ 8.30 (s, 1H), 7.91 (d, *J* = 8.0 Hz, 2H), 7.46 – 7.35 (m, 2H), 7.21 (d, *J* = 8.0 Hz, 2H), 6.02 – 5.78 (m, 1H), 5.30 (d, *J* = 17.2 Hz, 1H), 5.20 (d, *J* = 10.4 Hz, 1H), 4.97 (s, 2H), 4.04 (d, *J* = 4.4 Hz, 2H), 3.33 (s, 3H), 3.13 (s, 3H), 2.35 (s, 3H); ^{13}C NMR (125 MHz, $CDCl_3$) δ 145.3, 139.7, 136.4, 135.4, 134.4, 129.7, 127.9, 127.4, 127.2, 121.4, 119.7, 117.7, 117.3, 107.3, 89.5, 71.3, 62.5, 60.2, 39.2, 37.1, 21.6; IR (neat): 2927, 2240(s), 1444, 1360(s), 1266, 1175, 1112, 999, 581; HRESIMS Calcd for $[C_{23}H_{23}BrN_2NaO_5S_2]^+$ (M + Na^+) 573.0124, found 573.0127.

methyl 2-((allyloxy)methyl)-3-((*N*-methylmethylsulfonamido)ethynyl)-1-tosyl-1*H*-indole-6-carboxylate (1o)

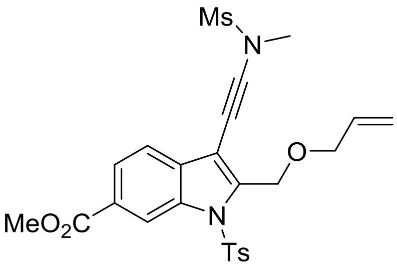

1o

Pale yellow oil. ^{1}H NMR (500 MHz, $CDCl_3$) δ 8.82 (s, 1H), 8.05 – 7.90 (m, 3H), 7.61 (d, *J* = 8.5 Hz, 1H), 7.20 (d, *J* = 8.0 Hz, 2H), 5.99 – 5.86 (m, 1H), 5.31 (dd, *J* = 18.5, 1.0 Hz, 1H), 5.21 (d, *J* = 10.5 Hz, 1H), 5.02 (s, 2H), 4.06 (d, *J* = 5.5 Hz, 2H), 3.97 (s, 3H), 3.34 (s, 3H), 3.14 (s, 3H), 2.33 (s, 3H);^{13}C NMR (125 MHz, $CDCl_3$) δ 167.0, 145.3, 142.0, 135.3, 135.2, 134.3, 132.6, 129.7, 127.7, 127.4, 124.8, 120.0, 117.3, 116.4, 107.2, 89.5, 71.3, 62.5, 60.1, 52.3, 39.2, 37.1, 21.5; IR (neat): 2923, 2240(s), 1717(s), 1358(s), 1174, 1161, 1089, 685; HRESIMS Calcd for $[C_{25}H_{26}N_2NaO_7S_2]^+$ (M + Na^+) 553.1074, found 553.1079.

N-((2-((allyloxy)methyl)-7-methyl-1-tosyl-1H-indol-3-yl)ethynyl)-N-methylmethanesulfonamide (1p)

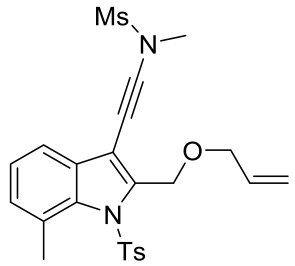

1p

Pale yellow oil. ^{1}H NMR (400 MHz, $CDCl_3$) δ 7.68 (d, J = 8.4 Hz, 2H), 7.39 (d, J = 7.2 Hz, 1H), 7.23 – 7.09 (m, 4H), 6.00 – 5.82 (m, 1H), 5.29 (dd, J = 17.2, 1.6 Hz, 1H), 5.17 (dd, J = 10.4, 0.8 Hz, 1H), 5.02 (s, 2H), 4.06 (d, J = 5.6 Hz, 2H), 3.32 (s, 3H), 3.13 (s, 3H), 2.51 (s, 3H), 2.35 (s, 3H); ^{13}C NMR (100 MHz, $CDCl_3$) δ 144.4, 142.0, 137.8, 136.1, 134.4, 131.7, 130.0, 129.3, 127.2, 126.6, 124.7, 117.9, 117.0, 110.3, 90.0, 71.3, 64.5, 60.7, 39.1, 36.9, 22.1, 21.4; IR (neat): 2930, 2245(s), 1440, 1368(s), 1260, 1182, 1122, 736, 585; HRESIMS Calcd for $[C_{24}H_{26}N_2NaO_5S_2]^+$ (M + Na^+) 509.1175, found 509.1173.

N-((2-((allyloxy)methyl)-1-((4-bromophenyl)sulfonyl)-1H-indol-3-yl)ethynyl)-N-methylmethanesulfonamide (1q)

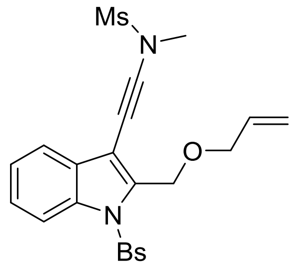

1q

Pale yellow oil. ^{1}H NMR (400 MHz, $CDCl_3$) δ 8.09 (d, J = 8.4 Hz, 1H), 7.94 (d, J = 8.8 Hz, 2H), 7.59 (d, J = 7.6 Hz, 1H), 7.52 (d, J = 8.8 Hz, 2H), 7.43 – 7.35 (m, 1H), 7.34 – 7.28 (m, 1H), 6.00 – 5.84 (m, 1H), 5.30 (dd, J = 17.6, 1.6 Hz, 1H), 5.22 (d, J = 10.4 Hz, 1H), 5.00 (s, 2H), 4.05 (d, J = 5.6 Hz, 2H), 3.34 (s, 3H), 3.14 (s, 3H); ^{13}C NMR (125 MHz, $CDCl_3$) δ 139.0, 137.4, 135.7, 134.4, 132.3, 129.2, 129.1, 128.9, 126.2, 124.2, 120.4, 117.3, 114.5, 108.2, 89.5, 71.2, 62.5, 60.5, 39.2, 37.0; IR (neat): 2923, 2239(s),

1357(s), 1170, 1160, 1108, 996, 576; HRESIMS Calcd for $[C_{22}H_{21}BrN_2NaO_5S_2]^+$ (M + Na^+) 558.9967, found 558.9974.

***N*-((2-((allyloxy)methyl)-1-(methylsulfonyl)-1*H*-indol-3-yl)ethynyl)-*N*-methylmethanesulfonamide (1r)**

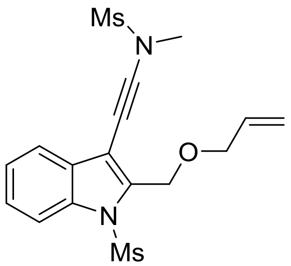

1r

Pale yellow oil. ^{1}H NMR (400 MHz, $CDCl_3$) δ 8.06 (d, *J* = 8.4 Hz, 1H), 7.65 (d, *J* = 7.2 Hz, 1H), 7.42 – 7.30 (m, 2H), 6.04 – 5.87 (m, 1H), 5.33 (dd, *J* = 17.2, 1.6 Hz, 1H), 5.21 (dd, *J* = 10.4, 1.2 Hz, 1H), 4.93 (s, 2H), 4.12 (d, *J* = 5.6 Hz, 2H), 3.36 (s, 3H), 3.30 (s, 3H), 3.16 (s, 3H); ^{13}C NMR (100 MHz, $CDCl_3$) δ 138.8, 136.0, 134.2, 128.8, 126.1, 123.9, 120.4, 117.6, 114.1, 106.6, 89.2, 71.5, 62.7, 60.4, 41.6, 39.2, 37.0; IR (neat): 32923, 2850, 2240(s), 1359(s), 1258, 1172, 1109, 999, 766; HRESIMS Calcd for $[C_{17}H_{20}N_2NaO_5S_2]^+$ (M + Na^+) 419.0706, found 419.0711.

***N*-((2-((but-3-en-2-yloxy)methyl)-1-tosyl-1*H*-indol-3-yl)ethynyl)-*N*-methylmethanesulfonamide (1s)**

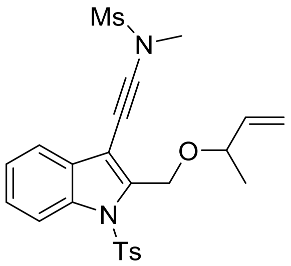

1s

Pale yellow oil. ^{1}H NMR (500 MHz, $CDCl_3$) δ 8.06 (d, *J* = 8.5 Hz, 1H), 7.96 (d, *J* = 8.5 Hz, 2H), 7.57 (d, *J* = 7.5 Hz, 1H), 7.35 – 7.30 (m, 1H), 7.29 – 7.23 (m, 1H), 7.18 (d, *J* = 8.0 Hz, 2H), 5.91 – 5.79 (m, 1H), 5.32 (d, *J* = 17.0 Hz, 1H), 5.19 (d, *J* = 10.5 Hz, 1H), 5.03 (d, *J* = 11.5 Hz, 1H), 4.84 (d, *J* = 11.5 Hz, 1H), 4.16 – 4.05 (m, 1H), 3.32 (s, 3H),

3.13 (s, 3H), 2.31 (s, 3H), 1.25 (d, *J* = 6.5 Hz, 3H); ^{13}C NMR (125 MHz, $CDCl_3$) δ 144.9, 140.0, 135.6(7), 135.6(5), 129.5, 129.1, 127.3, 125.8, 123.7, 120.1, 116.1, 114.5, 107.0, 89.1, 77.1, 60.8, 60.7, 39.2, 36.8, 21.5, 21.1; IR (neat): 2930, 2240(s), 1451, 1360(s), 1176, 1165, 997, 747, 578; HRESIMS Calcd for $[C_{24}H_{26}N_2NaO_5S_2]^+$ (M + Na^+) 509.1175, found 509.1168.

***N*-methyl-*N*-((2-(((2-phenylallyl)oxy)methyl)-1-tosyl-1*H*-indol-3-yl)ethynyl)methanesulfonamide (1t)**

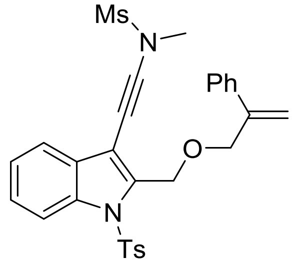

1t

Pale yellow oil. ^{1}H NMR (400 MHz, $CDCl_3$) δ 8.11 (d, *J* = 8.4 Hz, 1H), 7.89 (d, *J* = 8.4 Hz, 2H), 7.58 (d, *J* = 7.6 Hz, 1H), 7.53 – 7.45 (m, 2H), 7.40 – 7.22 (m, 5H), 7.11 (d, *J* = 8.4 Hz, 2H), 5.57 (s, 1H), 5.42 (d, *J* = 1.2 Hz, 1H), 5.09 (s, 2H), 4.45 (s, 2H), 3.23 (s, 3H), 3.02 (s, 3H), 2.30 (s, 3H); ^{13}C NMR (125 MHz, $CDCl_3$) δ 144.9, 143.9, 139.2, 138.8, 135.8, 135.5, 129.6, 129.1, 128.4, 127.8, 127.3, 126.0(4), 125.9(7), 123.8, 120.3, 114.7, 114.4, 107.7, 89.3, 72.1, 62.7, 60.7, 39.1, 36.8, 21.5; IR (neat): 2927, 2868, 2240(s), 1596, 1445, 1360(s), 1176, 577, 590; HRESIMS Calcd for $[C_{29}H_{28}N_2NaO_5S_2]^+$ (M + Na^+) 571.1332, found 571.1339.

***N*-methyl-*N*-((6-methyl-2-(((2-phenylallyl)oxy)methyl)-1-tosyl-1*H*-indol-3-yl)ethynyl)methanesulfonamide (1u)**

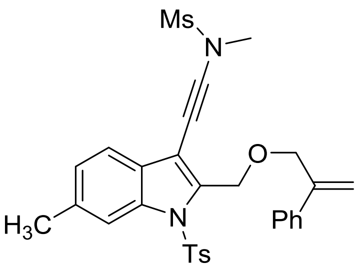

1u

Pale yellow oil. ^{1}H NMR (500 MHz, $CDCl_3$) δ 7.95 (s, 1H), 7.90 (d, *J* = 8.5 Hz, 2H), 7.52 – 7.45 (m, 3H), 7.35 – 7.29 (m, 2H), 7.29 – 7.24 (m, 1H), 7.14 – 7.08 (m, 3H), 5.59 (d, *J* = 0.5 Hz, 1H), 5.43 (d, *J* = 1.0 Hz, 1H), 5.08 (s, 2H), 4.46 (s, 2H), 3.23 (s, 3H), 3.03 (s, 3H), 2.50 (s, 3H), 2.30 (s, 3H); ^{13}C NMR (125 MHz, $CDCl_3$) δ 144.7, 143.8, 138.7, 138.5, 136.2, 135.6, 135.5, 129.5, 128.3, 127.7, 127.1, 126.8, 126.0, 125.3, 119.8, 114.6, 114.2, 107.6, 89.1, 71.9, 62.6, 60.7, 39.1, 36.7, 22.0, 21.4; IR (neat): 2923, 2239(s), 1446, 1360(s), 1175, 1111, 998, 579; HRESIMS Calcd for $[C_{30}H_{30}N_2NaO_5S_2]^+$ (M + Na^+) 585.1488, found 585.1491.

***N*-((5-chloro-2-(((2-phenylallyl)oxy)methyl)-1-tosyl-1*H*-indol-3-yl)ethynyl)-*N*-methylmethanesulfonamide (1v)**

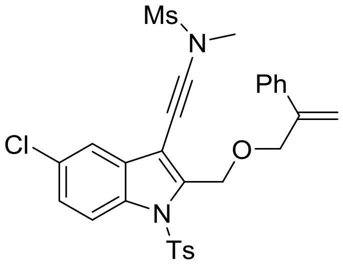

1v

Pale yellow oil. ^{1}H NMR (400 MHz, $CDCl_3$) δ 8.03 (d, *J* = 8.8 Hz, 1H), 7.86 (d, *J* = 8.4 Hz, 2H), 7.53 (d, *J* = 2.0 Hz, 1H), 7.47 (d, *J* = 6.8 Hz, 2H), 7.35 – 7.20 (m, 4H), 7.10 (d, *J* = 8.0 Hz, 2H), 5.57 (s, 1H), 5.41 (s, 1H), 5.05 (s, 2H), 4.45 (s, 2H), 3.23 (s, 3H), 3.02 (s, 3H), 2.29 (s, 3H); ^{13}C NMR (100 MHz, $CDCl_3$) δ 145.2, 143.7, 140.5, 138.6, 135.1, 134.0, 130.3, 129.7, 129.6, 128.3, 127.8, 127.3, 126.1, 126.0, 119.8, 115.8, 114.5, 107.0, 89.5, 72.2, 62.5, 59.9, 39.1, 36.9, 21.5; IR (neat): 2926, 2238(s), 1445, 1359(s), 1175, 1113, 999, 584; HRESIMS Calcd for $[C_{29}H_{27}ClN_2NaO_5S_2]^+$ (M + Na^+) 605.0942, found 605.0956.

***N*-((6-bromo-2-(((2-phenylallyl)oxy)methyl)-1-tosyl-1*H*-indol-3-yl)ethynyl)-*N*-methylmethanesulfonamide (1w)**

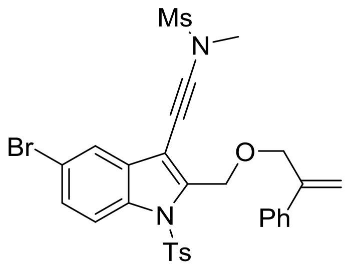

1w

Pale yellow solid (mp 120-121 °C). ^{1}H NMR (500 MHz, $CDCl_3$) δ 7.99 (d, *J* = 9.0 Hz, 1H), 7.87 (d, *J* = 8.5 Hz, 2H), 7.70 (d, *J* = 1.5 Hz, 1H), 7.52 – 7.46 (m, 2H), 7.45 (dd, *J* = 9.0, 2.0 Hz, 1H), 7.35 – 7.23 (m, 3H), 7.11 (d, *J* = 8.0 Hz, 2H), 5.59 (s, 1H), 5.42 (d, *J* = 1.5 Hz, 1H), 5.07 (s, 2H), 4.46 (s, 2H), 3.24 (s, 3H), 3.03 (s, 3H), 2.30 (s, 3H); ^{13}C NMR (125 MHz, $CDCl_3$) δ 145.2, 143.7, 140.4, 138.6, 135.2, 134.4, 130.8, 129.6, 128.8, 128.3, 127.8, 127.3, 126.0, 122.9, 117.3, 116.1, 114.5, 106.8, 89.6, 72.2, 62.5, 59.9, 39.1, 36.9, 21.5; IR (neat): 2927, 2240(s), 1596, 1445, 1360(s), 1176, 1113, 999, 667; HRESIMS Calcd for $[C_{29}H_{27}BrN_2NaO_5S_2]^+$ (M + Na^+) 649.0437, found 649.0451.

_N_-methyl-_N_-((2-(((2-(_p_-tolyl)allyl)oxy)methyl)-1-tosyl-1_H_-indol-3-yl)ethynyl)methanesulfonamide (1x)

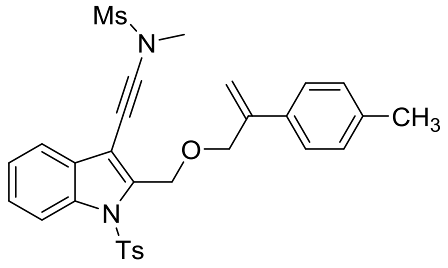

1x

Pale yellow oil. ^{1}H NMR (400 MHz, $CDCl_3$) δ 8.11 (d, *J* = 8.4 Hz, 1H), 7.89 (d, *J* = 8.4 Hz, 2H), 7.62 – 7.55 (m, 1H), 7.41 – 7.33 (m, 3H), 7.31 – 7.27 (m, 1H), 7.16 – 7.06 (m, 4H), 5.53 (s, 1H), 5.37 (d, *J* = 1.2 Hz, 1H), 5.08 (s, 2H), 4.43 (s, 2H), 3.23 (s, 3H), 3.03 (s, 3H), 2.32 (s, 3H), 2.30 (s, 3H); ^{13}C NMR (100 MHz, $CDCl_3$) δ 144.8, 143.7, 139.3, 137.5, 135.9, 135.8, 135.6, 129.5, 129.1, 129.0, 127.3, 125.9(3), 125.9(1), 123.8, 120.3, 114.7, 113.5, 107.6, 89.3, 72.1, 62.7, 60.7, 39.1, 36.8, 21.5, 21.1; IR (neat): 2923, 2240(s), 1446, 1365(s), 1170, 1111, 998, 580; HRESIMS Calcd for $[C_{30}H_{30}N_2NaO_5S_2]^+$ (M + Na^+) 585.1488, found 585.1491.

N-((2-(((2-(4-chlorophenyl)allyl)oxy)methyl)-1-tosyl-1H-indol-3-yl)ethynyl)-N-methylmethanesulfonamide (1y)

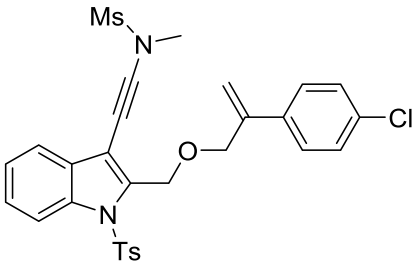

1y

Pale yellow oil. ^{1}H NMR (500 MHz, $CDCl_3$) δ 8.08 (d, *J* = 7.5 Hz, 1H), 7.84 (d, *J* = 7.0 Hz, 2H), 7.57 (d, *J* = 7.0 Hz, 1H), 7.48 – 7.16 (m, 6H), 7.07 (d, *J* = 7.0 Hz, 2H), 5.55 (s, 1H), 5.41 (s, 1H), 5.05 (s, 2H), 4.43 (s, 2H), 3.25 (s, 3H), 3.06 (s, 3H), 2.28 (s, 3H); ^{13}C NMR (125 MHz, $CDCl_3$) δ 144.9, 142.9, 139.1, 137.1, 135.8, 135.5, 133.5, 129.5, 129.0, 128.4, 127.5, 127.2, 126.0, 123.8, 120.2, 115.2, 114.7, 107.7, 89.4, 72.2, 62.7, 60.6, 39.1, 36.9, 21.5; IR (neat): 2932, 2250(s), 1443, 1370(s), 1260, 1182, 1122, 736, 575; HRESIMS Calcd for $[C_{29}H_{27}ClN_2NaO_5S_2]^+$ (M + Na^+) 605.0942, found 605.0956.

N-methyl-N-((2-(((2-methylallyl)oxy)methyl)-1-tosyl-1H-indol-3-yl)ethynyl)methanesulfonamide (1z)

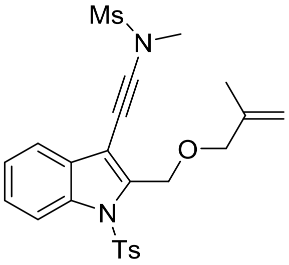

1z

Pale yellow oil. ^{1}H NMR (400 MHz, $CDCl_3$) δ 8.11 (d, *J* = 8.4 Hz, 1H), 7.93 (d, *J* = 8.4 Hz, 2H), 7.58 (dd, *J* = 7.6, 0.4 Hz, 1H), 7.39 – 7.33 (m, 1H), 7.31 – 7.27 (m, 1H), 7.17 (d, *J* = 8.0 Hz, 2H), 5.03 (d, *J* = 1.2 Hz, 1H), 5.00 (s, 2H), 4.93 (d, *J* = 0.4 Hz, 1H), 3.97 (s, 2H), 3.33 (s, 3H), 3.13 (s, 3H), 2.33 (s, 3H), 1.77 (s, 3H); ^{13}C NMR (100 MHz, $CDCl_3$) δ 144.9, 142.1, 139.5, 135.8, 135.7, 129.6, 129.2, 127.4, 125.9, 123.8, 120.2, 114.7, 112.1, 107.5, 89.2, 74.1, 62.6, 60.8, 39.2, 36.9, 21.5, 19.6; IR (neat): 2923, 2239(s), 1446,

1360(s), 1170, 1111, 1089, 998, 579; HRESIMS Calcd for $[C_{24}H_{26}N_2NaO_5S_2]^+$ (M + Na^+) 509.1175, found 509.1182.

N-((2-((allyloxy)methyl)benzofuran-3-yl)ethynyl)-*N*-methylmethanesulfonamide (3a)

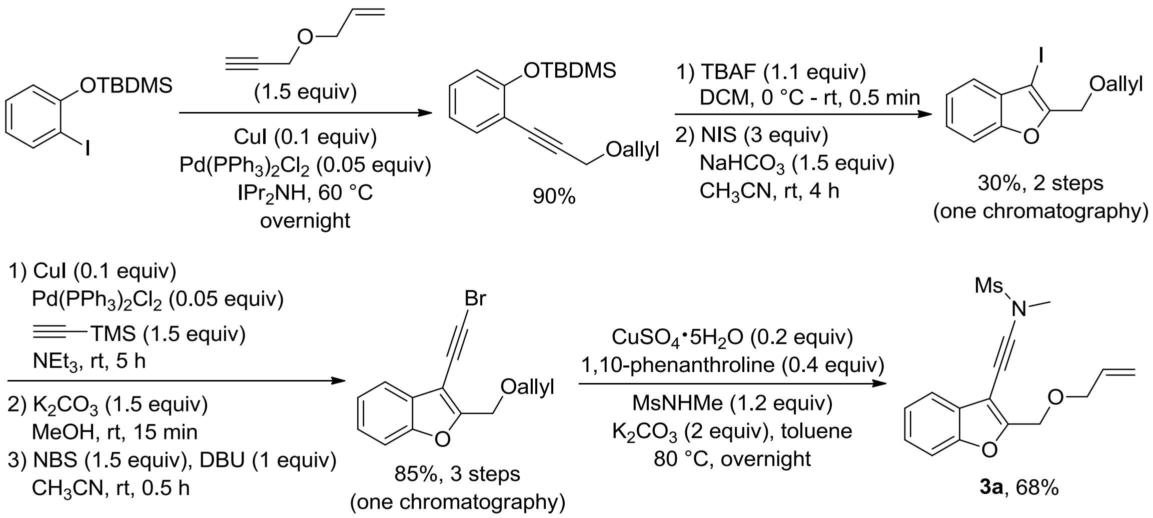

Compound **3a** was prepared according to the above known procedures.[1-3] Pale yellow oil. ^1H NMR (400 MHz, $CDCl_3$) δ 7.64 – 7.56 (m, 1H), 7.51 – 7.43 (m, 1H), 7.37 – 7.26 (m, 2H), 6.02 – 5.88 (m, 1H), 5.41 – 5.30 (m, 1H), 5.28 – 5.19 (m, 1H), 4.71 (s, 2H), 4.11 (dt, J = 5.6, 1.6 Hz, 2H), 3.35 (s, 3H), 3.16 (s, 3H); ^{13}C NMR (100 MHz, $CDCl_3$) δ 157.3, 154.3, 134.1, 128.2, 125.5, 123.3, 120.3, 117.9, 111.6, 102.8, 88.8, 71.5, 62.7, 59.2, 39.3, 36.9; IR (neat): 2922, 2851, 2239(s), 1469, 1356(s), 1158, 945, 747; HRESIMS Calcd for $[C_{16}H_{17}NNaO_4S]^+$ (M + Na^+) 342.0770, found 342.0770.

N-((2-((allyloxy)methyl)-1-tosyl-1*H*-pyrrol-3-yl)ethynyl)-*N*-methylmethanesulfonamide (3b)

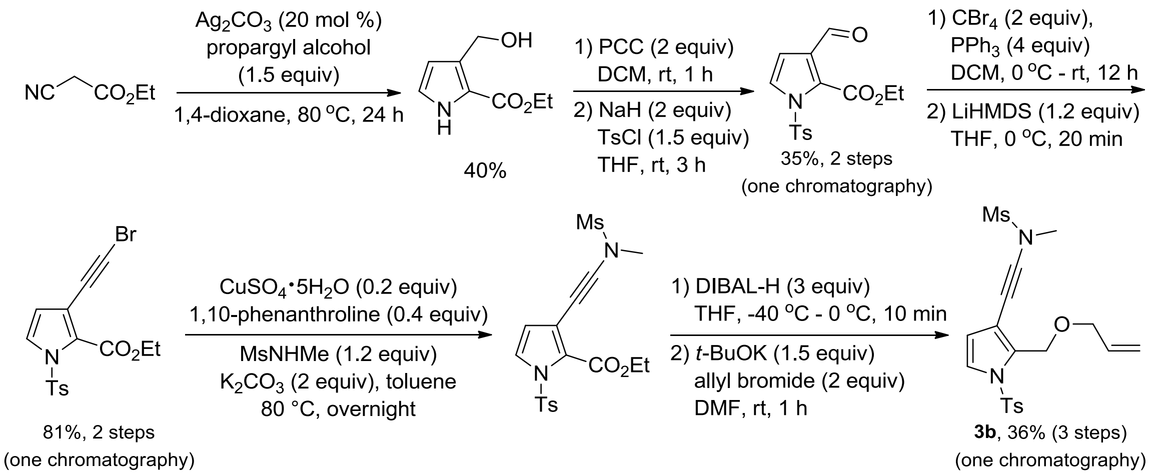

Compound **3b** was prepared according to the above known procedures.[1-4] Pale yellow oil. ^{1}H NMR (400 MHz, $CDCl_3$) δ 7.82 (d, *J* = 8.4 Hz, 2H), 7.32 – 7.22 (m, 3H), 6.27 (d, *J* = 3.2 Hz, 1H), 5.84 – 5.69 (m, 1H), 5.25 – 5.08 (m, 2H), 4.72 (s, 2H), 3.84 (dt, *J* = 5.6, 1.2 Hz, 2H), 3.24 (s, 3H), 3.06 (s, 3H), 2.39 (s, 3H); ^{13}C NMR (100 MHz, $CDCl_3$) δ 145.0, 135.9, 134.5, 134.3, 129.6, 127.6, 123.2, 116.9, 114.0, 111.5, 85.7, 70.6, 62.3, 61.1, 39.1, 36.8, 21.5; IR (neat): 2926, 2238(s), 1445, 1360(s), 1265, 1175, 1111, 1000, 738, 583; HRESIMS Calcd for $[C_{19}H_{22}N_2NaO_5S_2]^+$ (M + Na^+) 445.0862, found 445.0863.

***N*-((2-((allyloxy)methyl)-4,5-dimethoxyphenyl)ethynyl)-*N*-methylmethanesulfonamide (3c)**

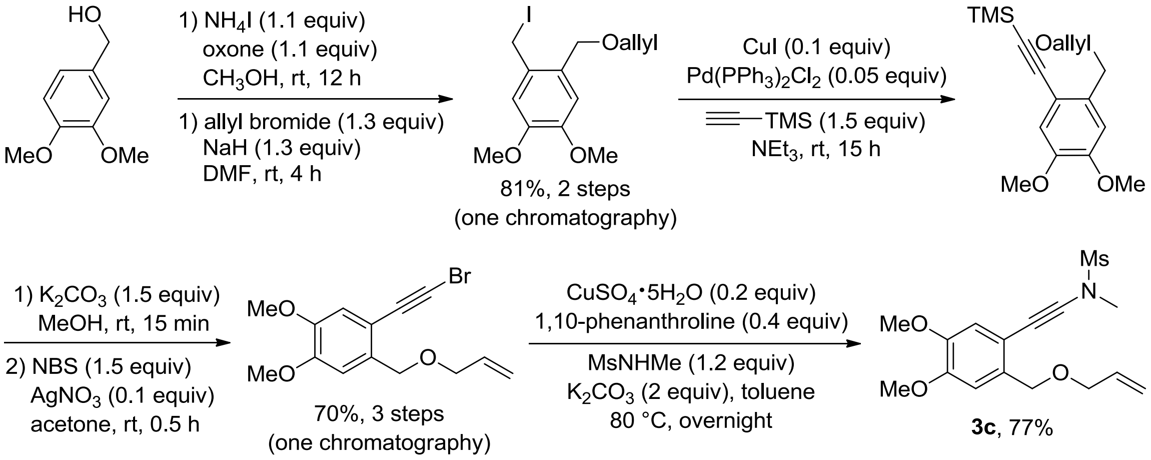

Compound **3c** was prepared according to the above known procedures.[1-3] Pale yellow solid (mp 100-101 ^{o}C). ^{1}H NMR (500 MHz, $CDCl_3$) δ 6.97 (s, 1H), 6.88 (s, 1H), 6.06 – 5.90 (m, 1H), 5.31 (dd, *J* = 17.5, 1.5 Hz, 1H), 5.20 (dd, *J* = 10.0, 1.0 Hz, 1H), 4.59 (s,

2H), 4.06 (t, *J* = 1.0 Hz, 2H), 3.90 (s, 3H), 3.86 (s, 3H), 3.29 (s, 3H), 3.12 (s, 3H); ^{13}C NMR (125 MHz, $CDCl_3$) δ 149.4, 148.0, 134.8, 133.6, 117.1, 114.4, 113.1, 111.1, 85.7, 71.3, 70.1, 67.3, 56.0, 55.9, 39.2, 36.7; IR (neat): 2927, 2240, 1596, 1445, 1360(s), 1176, 1088, 960, 590; HRESIMS Calcd for $[C_{16}H_{21}NNaO_5S]^+$ (M + Na^+) 362.1033, found 362.1035.

(*E*)-*N*-((2-((but-2-en-1-yloxy)methyl)-1-tosyl-1*H*-indol-3-yl)ethynyl)-*N*-methylmethanesulfonamide (3d)

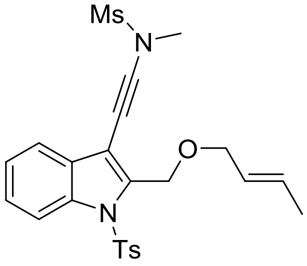

3d

Compound **3d** was prepared according to the general procedures for the synthesis of ynamides **1**.[1-3] Pale yellow oil. ^{1}H NMR (400 MHz, $CDCl_3$) δ 8.13 (d, *J* = 8.4 Hz, 1H), 7.93 (d, *J* = 8.4 Hz, 2H), 7.66 – 7.51 (m, 1H), 7.41 – 7.32 (m, 1H), 7.30 – 7.24 (m, 1H), 7.17 (d, *J* = 8.0 Hz, 2H), 5.84 – 5.65 (m, 1H), 5.65 – 5.49 (m, 1H), 4.98 (s, 2H), 3.98 (d, *J* = 6.4 Hz, 2H), 3.33 (s, 3H), 3.13 (s, 3H), 2.32 (s, 3H), 1.72 (dd, *J* = 6.4, 1.2 Hz, 3H); ^{13}C NMR (100 MHz, $CDCl_3$) δ 144.8, 139.4, 135.8, 135.7, 129.5(4), 129.4(6), 129.0, 127.3, 125.8, 123.7, 120.1, 114.6, 107.3, 89.2, 70.8, 62.3, 60.6, 39.2, 36.8, 21.4, 17.7; IR (neat): 2930, 2245(s), 1360, 1107, 1090, 748, 580; HRESIMS Calcd for $[C_{24}H_{26}N_2NaO_5S_2]^+$ (M + Na^+) 509.1175, found 509.1179.

***N*-allyl-*N*-((3-((*N*-methylmethylsulfonamido)ethynyl)-1-tosyl-1*H*-indol-2-yl)methyl)methanesulfonamide (3e)**

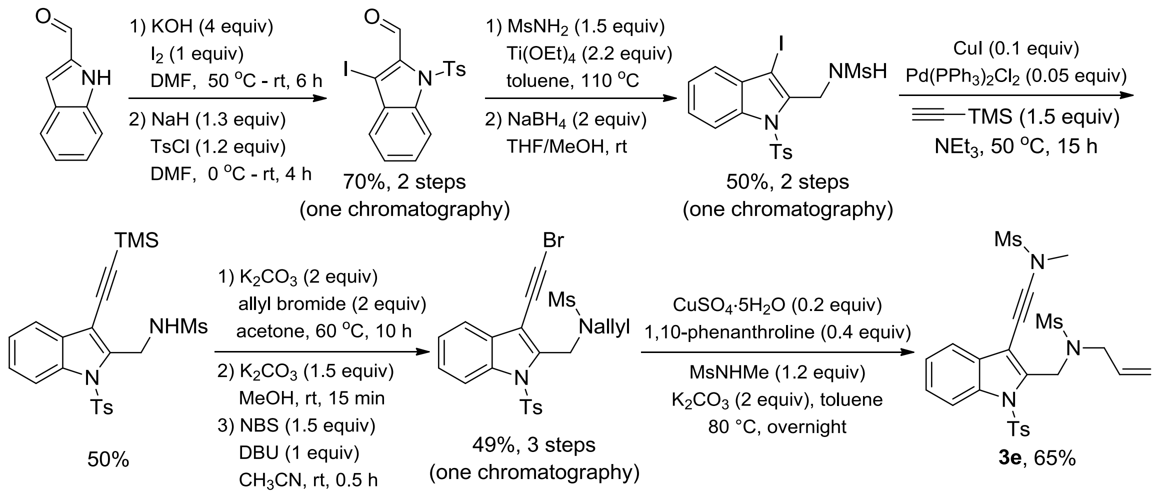

Compound **3e** was prepared according to the above known procedures.[1-3] Pale yellow oil. ^{1}H NMR (500 MHz, $CDCl_3$) δ 8.14 (d, *J* = 8.0 Hz, 1H), 7.62 (d, *J* = 8.0 Hz, 2H), 7.57 (d, *J* = 7.5 Hz, 1H), 7.43 – 7.34 (m, 1H), 7.33 – 7.27 (m, 1H), 7.19 (d, *J* = 8.0 Hz, 2H), 5.83 – 5.67 (m, 1H), 5.14 (d, *J* = 17.0 Hz, 1H), 5.07 (d, *J* = 10.0 Hz, 1H), 4.98 (s, 2H), 3.90 (d, *J* = 6.5 Hz, 2H), 3.35 (s, 3H), 3.16 (s, 3H), 2.96 (s, 3H), 2.33 (s, 3H); ^{13}C NMR (125 MHz, $CDCl_3$) δ 145.5, 136.9, 136.4, 134.8, 132.5, 130.1, 130.0, 126.5, 126.2, 124.5, 120.3, 118.9, 115.2, 109.2, 91.0, 60.2, 50.3, 44.0, 39.9, 39.2, 37.2, 21.5; IR (neat): 2926, 2235(s), 1440, 1363(s), 1265, 1175, 1111, 1000, 738, 585; HRESIMS Calcd for $[C_{24}H_{27}N_3NaO_6S_3]^+$ (M + Na^+) 572.0954, found 572.0956.

***N*-((2-((allylthio)methyl)-1-tosyl-1*H*-indol-3-yl)ethynyl)-*N*-methylmethanesulfonamide (3f)**

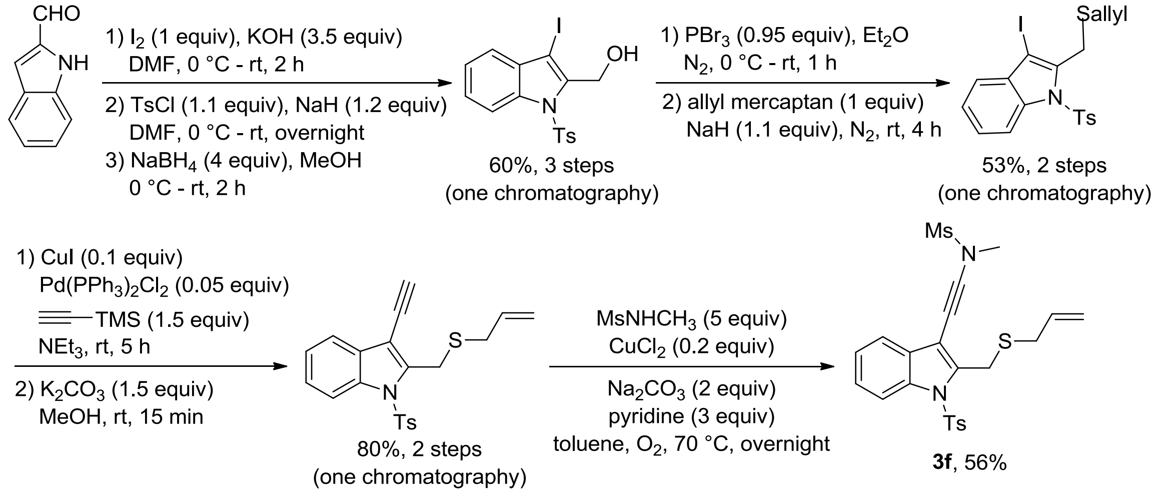

Compound **3f** was prepared according to the above known procedures.[1-3] ^{1}H NMR (500 MHz, $CDCl_3$) δ 8.04 (d, *J* = 8.5 Hz, 1H), 7.79 (d, *J* = 8.5 Hz, 2H), 7.53 (dd, *J* = 8.0, 1.0 Hz, 1H), 7.34 – 7.23 (m, 2H), 7.18 (d, *J* = 8.1 Hz, 2H), 5.96 – 5.78 (m, 1H), 5.30 – 5.20 (m, 1H), 5.16 – 5.04 (m, 1H), 4.28 (s, 2H), 3.33 (s, 3H), 3.29 (d, *J* = 7.0 Hz, 2H), 3.15 (s, 3H), 2.31 (s, 3H); ^{13}C NMR (125 MHz, $CDCl_3$) δ 145.1, 141.7, 135.8, 135.4, 134.2, 129.7, 129.4, 126.8, 125.4, 124.0, 119.7, 117.0, 114.7, 105.6, 89.6, 60.8, 39.2, 37.0, 35.3, 27.7, 21.4; IR (neat): 2930, 2250(s), 1365, 1127, 1090, 750, 590; HRESIMS Calcd for $[C_{23}H_{24}N_2NaO_4S_3]^+$ (M + Na^+) 511.0790, found 511.0796.

N-((2-(2-(allyloxy)ethyl)-1-tosyl-1*H*-indol-3-yl)ethynyl)-*N*-methylmethanesulfonamide (3g)

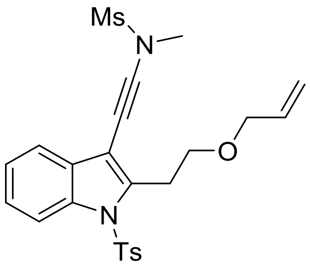

3g

Compound **3g** was prepared according to the general procedures for the synthesis of ynamides **1**.[1-3] Pale yellow oil. ^{1}H NMR (400 MHz, $CDCl_3$) δ 8.15 (d, *J* = 6.8 Hz, 1H), 7.62 (d, *J* = 6.8 Hz, 2H), 7.53 (dd, *J* = 6.0, 0.4 Hz, 1H), 7.36 – 7.21 (m, 2H), 7.14 (d, *J* = 6.4 Hz, 2H), 5.96 – 5.80 (m, 1H), 5.32 – 5.22 (m, 1H), 5.14 (dd, *J* = 8.0, 1.2 Hz, 1H), 4.01 (dt, *J* = 4.4, 0.8 Hz, 2H), 3.81 (t, *J* = 5.6 Hz, 2H), 3.49 (t, *J* = 5.6 Hz, 2H), 3.29 (s, 3H), 3.12 (s, 3H), 2.27 (s, 3H); ^{13}C NMR (125 MHz, $CDCl_3$) δ 145.1, 141.6, 136.0, 135.6, 134.8, 129.9(0), 129.8(6), 126.3, 125.1, 124.0, 119.5, 116.6, 114.8, 106.0, 89.0, 71.6, 69.4, 60.8, 39.3, 36.7, 28.8, 21.5; IR (neat): 2931, 2241(s), 1359, 1107, 1089, 748, 577; HRESIMS Calcd for $[C_{24}H_{26}N_2NaO_5S_2]^+$ (M + Na^+) 509.1175, found 509.1179.

***N*-((2-((allyloxy)methyl)phenyl)ethynyl)-*N*-methylmethanesulfonamide (3h)**

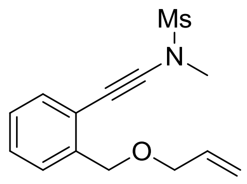

3h

Compound **3h** was prepared according to the general procedures for the synthesis of ynamides **1**.[1-3] Pale yellow oil. ^{1}H NMR (400 MHz, $CDCl_3$) δ 7.45 (d, 1H, *J* = 7.6 Hz), 7.41 – 7.36 (m, 1H), 7.32 – 7.26 (m, 1H), 7.25 – 7.19 (m, 1H), 6.02 – 5.91 (m, 1H), 5.35 – 5.27 (m, 1H), 5.24 – 5.17 (m, 1H), 4.63 (s, 2H), 4.09 – 4.06 (m, 2H), 3.29 (s, 3H), 3.11 (s, 3H); ^{13}C NMR (100 MHz, $CDCl_3$) δ 139.5, 134.7, 131.3, 127.9, 127.8, 127.2, 121.1, 116.9, 87.3, 71.3, 70.2, 67.4, 39.0, 36.7; IR (neat): 3016, 2931, 2856, 2235, 1360, 1163, 959, 779, 516; HRESIMS Calcd for $[C_{14}H_{17}NNaO_3S]^+$ (M + Na^+) 302.0821, found 302.0825.

(*S*)-*N*-((2-((allyloxy)methyl)-1-tosyl-1*H*-indol-3-yl)ethynyl)-*N*-(1-phenylethyl)methanesulfonamide (5a)

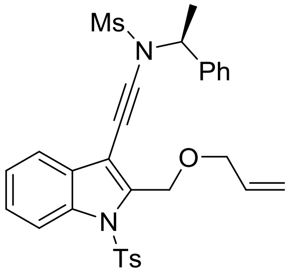

5a

Compound **5a** was prepared according to the general procedures for the synthesis of ynamides **1**.[1-3] Pale yellow oil. $[\alpha]_D^{20}$ = -246.7 ^{o}(c = 1.0, $CHCl_3$). ^{1}H NMR (400 MHz, $CDCl_3$) δ 8.14 (d, *J* = 8.4 Hz, 1H), 7.96 (d, *J* = 8.4 Hz, 2H), 7.56 – 7.47 (m, 3H), 7.44 – 7.33 (m, 4H), 7.33 – 7.25 (m, 1H), 7.19 (d, *J* = 8.0 Hz, 2H), 6.00 – 5.82 (m, 1H), 5.33 – 5.22 (m, 2H), 5.17 (dd, *J* = 10.8, 1.6 Hz, 1H), 5.00 (d, *J* = 0.8 Hz, 2H), 4.01 (dt, *J* = 5.6, 1.2 Hz, 2H), 2.81 (s, 3H), 2.34 (s, 3H), 1.80 (d, *J* = 7.2 Hz, 3H); ^{13}C NMR (125 MHz, $CDCl_3$) δ 144.9, 139.6, 139.3, 135.9, 135.8, 134.6, 129.8, 129.3, 128.8, 128.7, 127.4, 127.0, 125.9, 123.8, 120.2, 117.0, 114.7, 107.8, 85.8, 71.2, 64.9, 62.8, 59.1, 39.1, 21.5,

19.9; IR (neat): 2925, 2854, 2239(s), 1451, 1361, 1166, 1094, 577; HRESIMS Calcd for $[C_{30}H_{30}N_2NaO_5S_2]^+$ (M + Na^+) 585.1488, found 585.1491.

Representative synthetic procedures for the preparation of chiral ynamides 5b-5i:[3f]

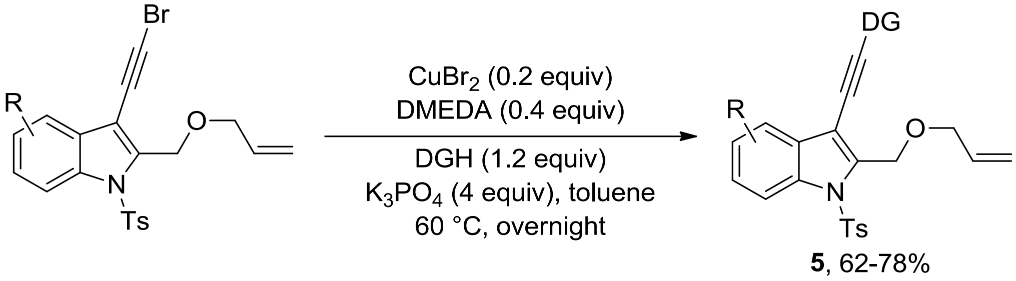

(*S*)-3-((2-((allyloxy)methyl)-1-tosyl-1*H*-indol-3-yl)ethynyl)-4-phenyloxazolidin-2-one (5b)

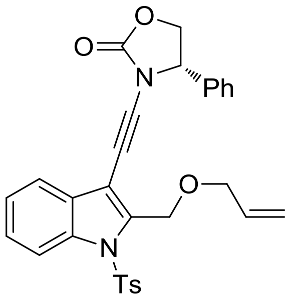

5b

Pale yellow oil. $[\alpha]_D^{20}$ = -41.5 °(c = 1.0, $CHCl_3$). ^{1}H NMR (400 MHz, $CDCl_3$) δ 8.06 (d, *J* = 8.4 Hz, 1H), 7.89 (d, *J* = 8.4 Hz, 2H), 7.58 – 7.37 (m, 5H), 7.36 – 7.22 (m, 2H), 7.21 – 7.08 (m, 3H), 5.93 – 5.66 (m, 1H), 5.33 – 5.07 (m, 3H), 4.94 – 4.70 (m, 3H), 4.34 (dd, *J* = 8.4, 7.6 Hz, 1H), 3.80 (d, *J* = 5.6 Hz, 2H), 2.31 (s, 3H); ^{13}C NMR (125 MHz, $CDCl_3$) δ 155.2, 144.9, 139.1, 135.9, 135.8, 135.7, 134.6, 129.7, 129.5, 129.4, 128.9, 127.4, 127.0, 125.8, 123.7, 120.3, 116.9, 114.5, 107.3, 84.3, 70.9, 70.8, 64.0, 62.5, 62.3, 21.5; IR (neat): 2920, 2257(s), 1779(s), 1374, 1176, 1087, 575; HRESIMS Calcd for $[C_{30}H_{26}N_2NaO_5S]^+$ (M + Na^+) 549.1455, found 549.1458.

(*S*)-3-((2-((allyloxy)methyl)-1-tosyl-1*H*-indol-3-yl)ethynyl)-4-benzhydryloxazolidin-2-one (5c)

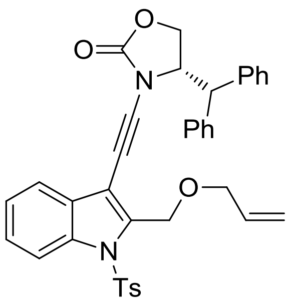

5c

Pale yellow solid (mp 140-141°C). $[\alpha]_D^{20}$ = -30.5 °(c = 1.0, $CHCl_3$). ^{1}H NMR (400 MHz, $CDCl_3$) δ 8.16 (d, *J* = 8.4 Hz, 1H), 7.97 (d, *J* = 8.4 Hz, 2H), 7.47 (d, *J* = 7.6 Hz, 1H), 7.42 – 7.22 (m, 11H), 7.18 (d, *J* = 8.4 Hz, 2H), 7.15 – 7.04 (m, 1H), 6.05 – 5.86 (m, 1H), 5.33 (d, *J* = 17.2 Hz, 1H), 5.21 (d, *J* = 10.4 Hz, 1H), 5.11 – 4.83 (m, 3H), 4.56 (d, *J* = 5.2 Hz, 2H), 4.35 – 4.21 (m, 1H), 4.05 (d, *J* = 5.2 Hz, 2H), 2.31 (s, 3H); ^{13}C NMR (100 MHz, $CDCl_3$) δ 155.4, 144.8, 139.2, 139.1, 138.4, 135.6, 135.5, 134.4, 129.4, 128.9, 128.7, 128.3, 128.1, 127.5, 127.3, 127.2, 125.7, 123.5, 120.4, 116.9, 114.3, 107.4, 84.6, 70.9, 66.5, 64.5, 62.4, 59.8, 53.2, 21.3; IR (neat): 2961, 2923, 2250(s), 1787(s), 1731, 1210, 1170, 580; HRESIMS Calcd for $[C_{37}H_{32}N_2NaO_5S]^+$ (M + Na^+) 639.1924, found 639.1925.

(*S*)-3-((2-((allyloxy)methyl)-1-tosyl-1H-indol-3-yl)ethynyl)-4-(*tert*-butyl)oxazolidin-2-one (5d)

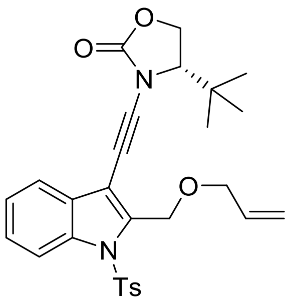

5d

Pale yellow solid (mp 135-136°C). $[\alpha]_D^{20}$ = -20.7 °(c = 1.0, $CHCl_3$). ^{1}H NMR (400 MHz, $CDCl_3$) δ 8.12 (d, *J* = 8.4 Hz, 1H), 7.94 (d, *J* = 8.4 Hz, 2H), 7.60 (d, *J* = 7.6 Hz, 1H), 7.42 – 7.32 (m, 1H), 7.31 – 7.23 (m, 1H), 7.17 (d, *J* = 8.4 Hz, 2H), 6.02 – 5.85 (m, 1H), 5.30 (dd, *J* = 17.2, 1.6 Hz, 1H), 5.19 (dd, *J* = 10.4, 1.2 Hz, 1H), 5.10 – 4.94 (m, 2H), 4.44 (t, *J* = 9.2 Hz, 1H), 4.27 (dd, *J* = 9.2, 5.2 Hz, 1H), 4.06 (d, *J* = 5.6 Hz, 2H), 3.88 (dd, *J* = 8.8, 5.2 Hz, 1H), 2.32 (s, 3H), 1.11 (s, 9H); ^{13}C NMR (100 MHz, $CDCl_3$) δ 156.3, 144.9,

139.2, 135.8, 135.7, 134.6, 129.5, 129.1, 127.4, 125.9, 123.8, 120.3, 117.0, 114.6, 107.7, 86.6, 71.2, 66.2, 65.5, 63.3, 62.6, 34.9, 25.3, 21.5; IR (neat): 2967, 2256(s), 1773(s), 1371, 1187, 1137, 662, 584; HRESIMS Calcd for $[C_{28}H_{30}N_2NaO_5S]^+$ (M + Na^+) 529.1768, found 529.1771.

(*S*)-3-((2-((allyloxy)methyl)-5-methyl-1-tosyl-1*H*-indol-3-yl)ethynyl)-4-(*tert*-butyl)oxazolidin-2-one (5e)

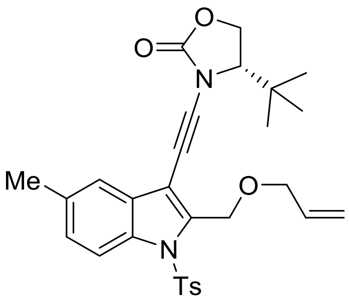

5e

Pale yellow solid (mp 142-143°C). $[\alpha]_D^{20}$ = -10.5 ^{o}(c = 1.0, $CHCl_3$). ^{1}H NMR (400 MHz, $CDCl_3$) δ 7.98 (d, *J* = 8.4 Hz, 1H), 7.91 (d, *J* = 8.4 Hz, 2H), 7.37 (s, 1H), 7.16 (d, *J* = 8.4 Hz, 3H), 6.00 – 5.83 (m, 1H), 5.29 (dd, *J* = 17.2, 1.6 Hz, 1H), 5.18 (d, *J* = 10.4 Hz, 1H), 5.00 (s, 2H), 4.44 (t, *J* = 9.2 Hz, 1H), 4.28 (dd, *J* = 9.2, 5.2 Hz, 1H), 4.05 (d, *J* = 5.6 Hz, 2H), 3.88 (dd, *J* = 8.8, 5.2 Hz, 1H), 2.41 (s, 3H), 2.32 (s, 3H), 1.11 (s, 9H); ^{13}C NMR (100 MHz, $CDCl_3$) δ 156.3, 144.8, 139.3, 135.8, 134.6, 134.1, 133.5, 129.5, 129.3, 127.3, 120.1, 117.0, 114.3, 107.5, 86.5, 71.1, 66.3, 65.5, 63.4, 62.6, 34.9, 25.3, 21.5, 21.1; IR (neat): 2961, 2923, 2254(s), 1777(s), 1731, 1217, 1176, 580; HRESIMS Calcd for $[C_{29}H_{32}N_2NaO_5S]^+$ (M + Na^+) 543.1924, found 543.1926.

(*S*)-3-((2-((allyloxy)methyl)-6-chloro-1-tosyl-1*H*-indol-3-yl)ethynyl)-4-(*tert*-butyl)oxazolidin-2-one (5f)

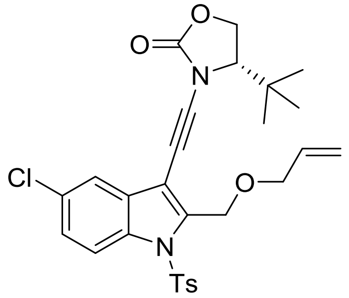

5f

Pale yellow solid (mp 150-151 °C). $[\alpha]_D^{20}$ = -30.5 °(c = 1.0, $CHCl_3$). ^{1}H NMR (400 MHz, $CDCl_3$) δ 8.14 (d, *J* = 1.6 Hz, 1H), 7.92 (d, *J* = 8.4 Hz, 2H), 7.50 (d, *J* = 8.4 Hz, 1H), 7.24 (dd, *J* = 8.4, 1.6 Hz, 1H), 7.20 (d, *J* = 8.4 Hz, 2H), 5.98 – 5.84 (m, 1H), 5.29 (dd, *J* = 17.6, 1.6 Hz, 1H), 5.19 (d, *J* = 10.4 Hz, 1H), 4.98 (d, *J* = 1.6 Hz, 2H), 4.44 (t, *J* = 8.8 Hz, 1H), 4.27 (dd, *J* = 9.2, 5.2 Hz, 1H), 4.04 (d, *J* = 5.6 Hz, 2H), 3.88 (dd, *J* = 8.8, 5.2 Hz, 1H), 2.34 (s, 3H), 1.10 (s, 9H); ^{13}C NMR (100 MHz, $CDCl_3$) δ 156.2, 145.2, 140.5, 135.4, 134.4, 134.1, 130.3, 129.7, 129.6, 127.3, 126.1, 119.9, 117.2, 115.8, 107.0, 87.0, 71.3, 66.2, 65.6, 62.6, 62.5, 34.9, 25.3, 21.5; IR (neat): 2967, 2261(s), 1174(s), 1371, 1179, 1137, 1071, 663, 585; HRESIMS Calcd for $[C_{28}H_{29}ClN_2NaO_5S]^+$ (M + Na^+) 563.1378, found 563.1375.

(*S*)-3-((2-((allyloxy)methyl)-5-bromo-1-tosyl-1*H*-indol-3-yl)ethynyl)-4-(*tert*-butyl)oxazolidin-2-one (5g)

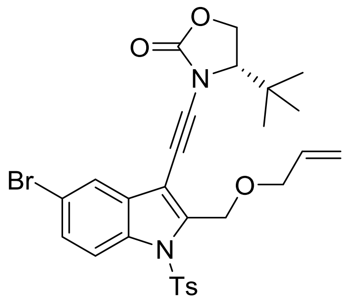

5g

Pale yellow solid (mp 170-171 °C). $[\alpha]_D^{20}$ = -10.5 °(c = 1.0, $CHCl_3$). ^{1}H NMR (400 MHz, $CDCl_3$) δ 7.98 (d, *J* = 8.8 Hz, 1H), 7.90 (d, *J* = 8.0 Hz, 2H), 7.69 (s, 1H), 7.42 (d, *J* = 8.8 Hz, 1H), 7.16 (d, *J* = 8.4 Hz, 2H), 5.98 – 5.83 (m, 1H), 5.29 (d, *J* = 17.2 Hz, 1H), 5.18 (d, *J* = 10.4 Hz, 1H), 4.98 (s, 2H), 4.43 (t, *J* = 9.2 Hz, 1H), 4.26 (dd, *J* = 9.2, 5.2 Hz, 1H), 4.05 (d, *J* = 5.6 Hz, 2H), 3.88 (dd, *J* = 8.8, 5.2 Hz, 1H), 2.29 (s, 3H), 1.08 (s, 9H); ^{13}C NMR (100 MHz, $CDCl_3$) δ 156.2, 145.2, 140.2, 135.2, 134.4, 134.3, 130.7, 129.5, 128.6, 127.2, 122.9, 117.2, 117.1, 116.0, 106.8, 87.0, 71.2, 66.1, 65.5, 62.4, 62.3, 34.8, 25.2, 21.4; IR (neat): 2963, 2257(s), 1771(s), 1445, 1372, 1247, 1178, 581; HRESIMS Calcd for $[C_{28}H_{29}BrN_2NaO_5S]^+$ (M + Na^+) 607.0873, found 607.0879.

methyl (*S)*-2-((allyloxy)methyl)-3-((4-(*tert*-butyl)-2-oxooxazolidin-3-yl)ethynyl)-1-tosyl-1*H*-indole-5-carboxylate (5h)

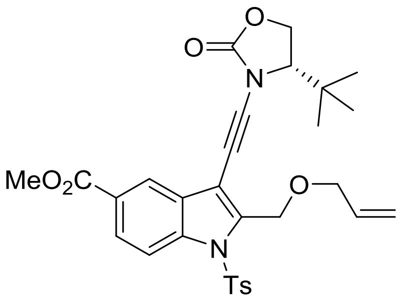

5h

Pale yellow solid (mp 150-151°C). $[\alpha]_D^{20}$ = -31.5 °(c = 1.0, $CHCl_3$). ^1H NMR (400 MHz, $CDCl_3$) δ 8.27 (d, *J* = 1.2 Hz, 1H), 8.14 (d, *J* = 8.8 Hz, 1H), 8.01 (dd, *J* = 8.8, 1.6 Hz, 1H), 7.92 (d, *J* = 8.4 Hz, 2H), 7.17 (d, *J* = 8.4 Hz, 2H), 5.99 – 5.80 (m, 1H), 5.29 (ddd, *J* = 17.2, 3.2, 1.6 Hz, 1H), 5.18 (dd, *J* = 10.4, 1.6 Hz, 1H), 5.08 – 4.93 (m, 2H), 4.45 (t, *J* = 9.0 Hz, 1H), 4.27 (dd, *J* = 9.2, 5.2 Hz, 1H), 4.05 (d, *J* = 5.6 Hz, 2H), 3.95 – 3.83 (m, 4H), 2.30 (s, 3H), 1.10 (s, 9H); $\delta^{13}C$ NMR (100 MHz, $CDCl_3$) δ 166.8, 156.2, 145.3, 140.6, 138.2, 135.2, 134.3, 129.6, 128.9, 127.4, 126.8, 125.8, 122.5, 117.2, 114.3, 107.9, 87.1, 71.3, 66.2, 65.6, 62.5, 62.4, 52.1, 34.9, 25.2, 21.4; IR (neat): 2951, 2933, 2255(s), 1790(s), 1780(s), 1731, 1218, 1175, 580; HRESIMS Calcd for $[C_{30}H_{32}N_2NaO_7S]^+$ (M + Na^+) 587.1822, found 587.1829.

(*S*)-3-((2-((allyloxy)methyl)-6-methyl-1-tosyl-1*H*-indol-3-yl)ethynyl)-4-(*tert*-butyl)oxazolidin-2-one (5i)

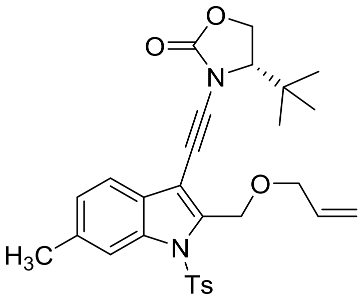

5i

Pale yellow solid (mp 130-131°C). $[\alpha]_D^{20}$ = -22.7 °(c = 1.0, $CHCl_3$). ^1H NMR (400 MHz, $CDCl_3$) δ 7.97 – 7.77 (m, 3H), 7.46 (d, *J* = 8.0 Hz, 1H), 7.18 (d, *J* = 8.4 Hz, 2H), 7.09 (d, *J* = 8.0 Hz, 1H), 6.01 – 5.81 (m, 1H), 5.29 (dd, *J* = 17.2, 1.6 Hz, 1H), 5.17 (dd, *J* = 10.4,

1.2 Hz, 1H), 5.08 – 4.88 (m, 2H), 4.43 (t, J = 9.2 Hz, 1H), 4.27 (dd, J = 9.2, 5.2 Hz, 1H), 4.04 (d, J = 5.6 Hz, 2H), 3.87 (dd, J = 8.8, 5.2 Hz, 1H), 2.48 (s, 3H), 2.32 (s, 3H), 1.11 (s, 9H); ^{13}C NMR (100 MHz, $CDCl_3$) δ 156.3, 144.8, 138.6, 136.2, 136.1, 135.8, 134.6, 129.5, 127.3, 126.8, 125.3, 119.9, 117.0, 114.7, 107.6, 86.5, 71.1, 66.2, 65.5, 63.4, 62.7, 34.9, 25.3, 22.1, 21.5; IR (neat): 2968, 2256(s), 1174(s), 1371, 1177, 662, 585; HRESIMS Calcd for $[C_{29}H_{32}N_2NaO_5S]^+$ (M + Na^+) 543.1924, found 543.1925.

***N*-((2-(((allyl-1,1-*d₂*)oxy)methyl)-1-tosyl-1*H*-indol-3-yl)ethynyl)-*N*-methylmethanesulfonamide (1a′)**

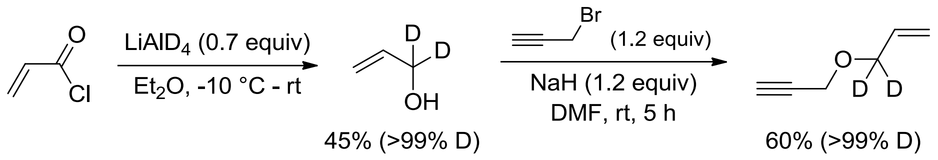

1,1-dideuterioallyl alcohol:[3b]

Under an argon atmosphere, $LiAlD_4$ (0.52 g, 12.5 mmol) and anhydrous ether (20 mL) were added into a 50 mL flame-dried flask fitted with magnetic stirrer bar at -10 ^{o}C. Then, a solution of acryloyl chloride (1.5 mL, 17.8 mmol) in ether was added dropwise over 10 min, The resulting mixture was warmed to rt slowly and stirred for 10 h. The mixture was cooled to -10 ^{o}C and H_2O (1.0 mL) was slowly added over a 5 min. period. After stirring for another 15 min, 15% aqueous NaOH solution (1.0 mL) and then H_2O (1.0 mL) were added. The resulting slurry was stirred for 1 h and then filtered. The filtrate was dried over Na_2SO_4. The solvent was removed carefuly on a rotary evaporator (atmospheric pressure, 37 ^{o}C) to afford a colorless liquid, which was used in the next step without further purification.

propargyl 1,1-dideuterioallyl ether:

NaH (18 mmol, 0.43 g) was added to a mixture of the 1,1-dideuterioallyl alcohol (15 mmol, 0.90 g) and DMF (15 mL) at rt. After stirring at rt for 1 h, propargyl bromide (18 mmol, 2.15 g) was added. The reaction mixture was stirred for another 5 h at rt then quenched with a saturated aqueous solution of NH_4Cl (30 mL). The resulting solution was extracted with EtOAc (2*30 mL) and washed with brine (2*30 mL). The combined organic layers were dried over anhydrous $MgSO_4$ and concentrated under reduced pressure, and the residue was purified by chromatography on silica gel (eluent:

hexanes/ethyl acetate) to afford the desired propargyl 1,1-dideuterioallyl ether in 60% yield (0.88 g).

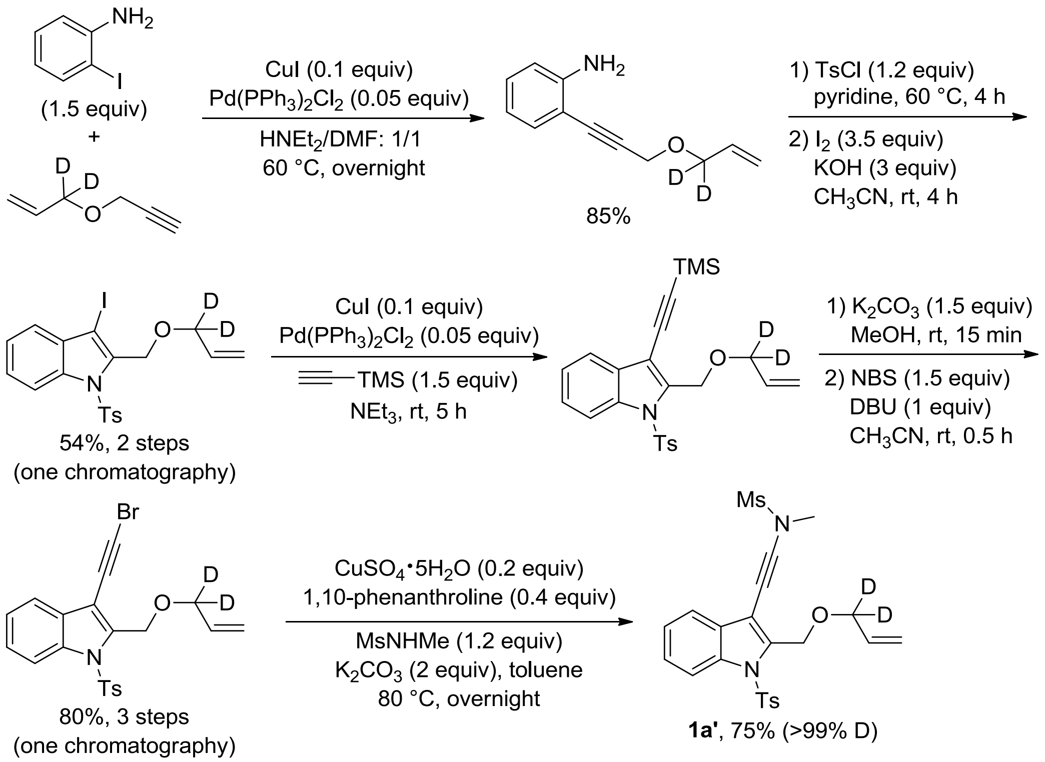

Compound **1a′** was prepared according to the above known procedures.[1-3] Pale yellow oil. ^{1}H NMR (400 MHz, $CDCl_3$) δ 8.12 (d, *J* = 8.4 Hz, 1H), 7.93 (d, *J* = 8.4 Hz, 2H), 7.58 (d, *J* = 7.6 Hz, 1H), 7.44 – 7.32 (m, 1H), 7.31 – 7.24 (m, 1H), 7.18 (d, *J* = 8.0 Hz, 2H), 5.93 (dd, *J* = 17.2, 10.4 Hz, 1H), 5.31 (dd, *J* = 17.2, 1.6 Hz, 1H), 5.21 (dd, *J* = 10.4, 1.6 Hz, 1H), 5.01 (s, 2H), 3.33 (s, 3H), 3.14 (s, 3H), 2.33 (s, 3H); IR (neat): 2927, 2240(s), 1450, 1358(s), 1176, 1109, 997, 764; HRESIMS Calcd for $[C_{23}H_{22}D_2N_2NaO_5S_2]^+$ (M + Na^+) 497.1144, found 497.1152.

***N*-((2-(((allyl-2-*d*)oxy)methyl)-1-tosyl-1*H*-indol-3-yl)ethynyl)-*N*-methylmethanesulfonamide (1a″)**

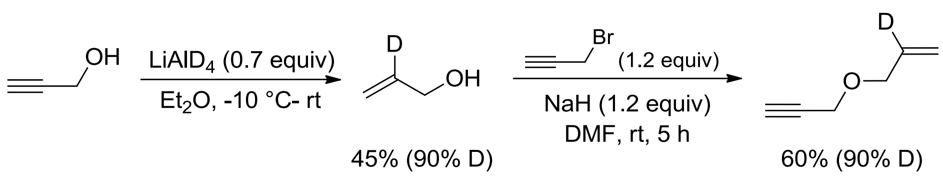

2-deuterioallyl alcohol:[3d]

Under an argon atmosphere, $LiAlD_4$ (0.52 g, 12.5 mmol) and anhydrous ether (20 mL) were added into a 50 mL flame-dried flask fitted with magnetic stirrer bar at -10 °C. Then,

a solution of allyl alcohol (1.0 g, 17.8 mmol) in ether was added dropwise over 10 min, The resulting mixture was warmed to rt slowly and stirred for 24 h. The mixture was cooled to -10 ^{o}C and H_2O (1.0 mL) was slowly added over a 5 min. period. After stirring for another 15 min, 15% aqueous NaOH solution (1.0 mL) and then H_2O (1.0 mL) were added. The resulting slurry was stirred for 1 h and then filtered. The filtrate was dried over Na_2SO_4. The solvent was removed carefuly on a rotary evaporator (atmospheric pressure, 37 ^{o}C) to afford a colorless liquid, which was used in the next step without further purification.

propargyl 2-deuterioallyl ether:

NaH (18 mmol, 0.43 g) was added to a mixture of the 2-deuterioallyl alcohol (15 mmol, 0.89 g) and DMF (15 mL) at rt. After stirring at rt for 1 h, propargyl bromide (18 mmol, 2.15 g) was added. The reaction mixture was stirred for another 5 h at rt then quenched with a saturated aqueous solution of NH_4Cl (30 mL). The resulting solution was extracted with EtOAc (2*30 mL) and washed with brine (2*30 mL). The combined organic layers were dried over anhydrous $MgSO_4$ and concentrated under reduced pressure, and the residue was purified by chromatography on silica gel (eluent: hexanes/ethyl acetate) to afford the desired propargyl 2-deuterioallyl ether in 60% yield (0.87 g).

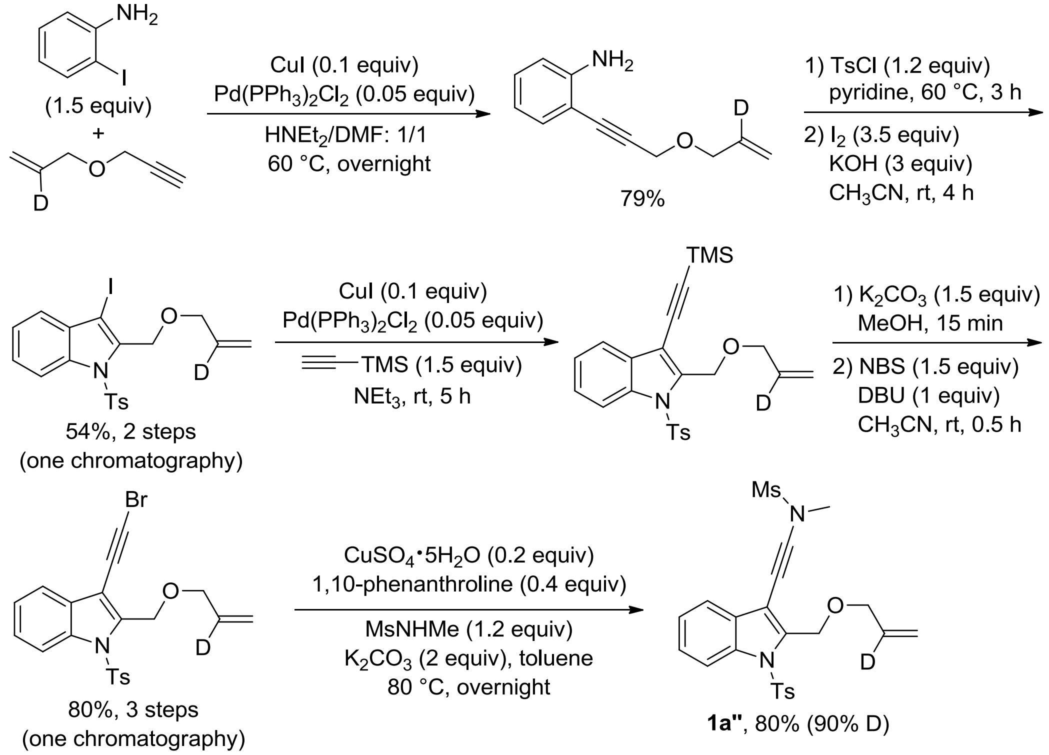

Compound **1a″** was prepared according to the above known procedures.[1-3] Pale yellow oil. ^{1}H NMR (400 MHz, $CDCl_3$) δ 8.13 (d, *J* = 8.4 Hz, 1H), 7.94 (d, *J* = 8.0 Hz, 2H), 7.59 (d, *J* = 7.6 Hz, 1H), 7.41 – 7.33 (m, 1H), 7.32 – 7.23 (m, 1H), 7.17 (d, *J* = 8.4 Hz, 2H), 6.00 – 5.87 (m, 0.1H), 5.31 (d, *J* = 1.6 Hz, 1H), 5.21 (s, 1H), 5.02 (s, 2H), 4.06 (s, 2H), 3.33 (s, 3H), 3.13 (s, 3H), 2.31 (s, 3H); IR (neat): 2920, 2230(s), 1359(s), 1275, 1260, 1176, 764, 749; HRESIMS Calcd for $[C_{23}H_{23}DN_2O_5S_2]^+$ (M + Na^+) 496.1082, found 496.1079.

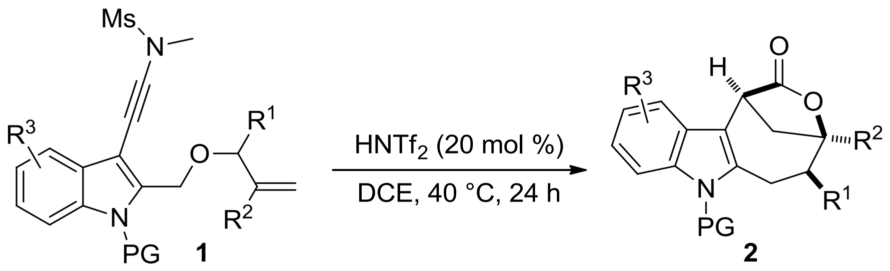

General procedure for the synthesis of indole-fused bridged [4.2.1] lactones 2:

$HNTf_2$ (0.04 mmol, 11.2 mg) was added to a mixture of the ynamide **1** (0.20 mmol) and DCE (4.0 mL) at room temperature. Then, the reaction mixture was stirred at 40 ℃ and the progress of the reaction was monitored by TLC. The reaction typically took 24 h. Upon completion, the mixture was concentrated and the residue was purified by chromatography on silica gel (eluent: hexanes/ethyl acetate) to afford the desired indole-fused bridged [4.2.1] lactone **2**.

7-tosyl-4,5,6,7-tetrahydro-1,4-methanooxocino[5,4-*b*]indol-2(1*H*)-one (2a)

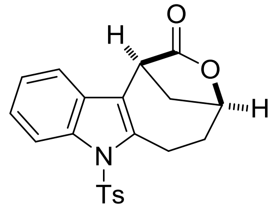

2a

Compound **2a** was prepared in 77% yield (58.7 mg) according to the general procedure (Table 2, entry 1). Pale yellow solid (mp 190-191 °C). ^{1}H NMR (400 MHz, $CDCl_3$) δ

8.23 (d, J = 8.0 Hz, 1H), 7.54 (d, J = 8.4 Hz, 2H), 7.49 – 7.39 (m, 1H), 7.40 – 7.23 (m, 2H), 7.17 (d, J = 8.4 Hz, 2H), 5.09 (t, J = 7.2 Hz, 1H), 3.94 (dt, J = 17.2, 4.4 Hz, 1H), 3.84 (d, J = 8.0 Hz, 1H), 2.96 – 2.80 (m, 1H), 2.76 – 2.61 (m, 1H), 2.47 – 2.34 (m, 1H), 2.31 (s, 3H), 1.87 (d, J = 12.4 Hz, 1H), 1.84 – 1.75 (m, 1H); ^{13}C NMR (125 MHz, $CDCl_3$) δ 175.5, 145.1, 136.7, 136.3, 135.8, 129.9, 128.5, 126.2, 124.9, 123.9, 121.4, 117.7, 115.4, 78.4, 37.0 35.9, 31.8, 21.6, 19.8; IR (neat): 2927, 1763(s), 1451, 1376, 1358, 1166, 1148, 974, 763; HRESIMS Calcd for $[C_{21}H_{19}NNaO_4S]^+$ (M + Na^+) 404.0927, found 404.0927.

4-allyl-9-tosyl-4,9-dihydropyrano[3,4-*b*]indol-3(1*H*)-one (2aa)

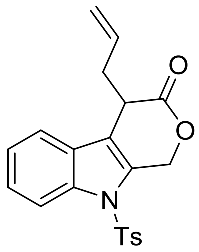

2aa

^{1}H NMR (400 MHz, $CDCl_3$) δ 8.11 (d, J = 8.0 Hz, 1H), 7.64 (d, J = 8.4 Hz, 2H), 7.47 – 7.34 (m, 2H), 7.33 – 7.17 (m, 3H), 5.85 – 5.64 (m, 2H), 5.55 – 5.41 (m, 1H), 4.96 – 4.81 (m, 2H), 4.08 – 3.96 (m, 1H), 2.93 – 2.80 (m, 1H), 2.75 – 2.63 (m, 1H), 2.35 (s, 3H); ^{13}C NMR (125 MHz, $CDCl_3$) δ 169.9, 145.6, 137.0, 134.7, 132.2, 130.1, 127.9, 127.3, 126.4, 125.5, 124.1, 119.5, 118.9, 116.3, 114.5, 67.0, 39.2, 36.7, 21.6; IR (neat): 2994, 1769(s), 1758, 1382, 1245, 1057, 913, 747; HRESIMS Calcd for $[C_{21}H_{19}NNaO_4S]^+$ (M + Na^+) 404.0927, found 404.0927.

10-methyl-7-tosyl-4,5,6,7-tetrahydro-1,4-methanooxocino[5,4-*b*]indol-2(1*H*)-one (2b)

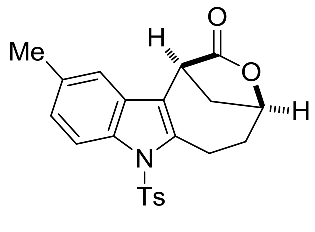

2b

Compound **2b** was prepared in 65% yield (51.4 mg) according to the general procedure (Table 2, entry 2). Pale yellow solid (mp 195-196 ^{o}C). ^{1}H NMR (400 MHz, $CDCl_3$) δ

8.10 (d, *J* = 8.4 Hz, 1H), 7.57 – 7.48 (m, 2H), 7.24 (s, 1H), 7.17 (d, *J* = 8.0 Hz, 2H), 7.13 (dd, *J* = 8.4, 1.2 Hz, 1H), 5.09 (t, *J* = 7.2 Hz, 1H), 3.93 (dt, *J* = 17.2, 4.4 Hz, 1H), 3.82 (d, *J* = 8.0 Hz, 1H), 2.90 – 2.80 (m, 1H), 2.78 – 2.63 (m, 1H), 2.42 (s, 3H), 2.41 – 2.36 (m, 1H), 2.33 (s, 3H), 1.87 (d, *J* = 12.4 Hz, 1H), 1.85 – 1.77 (m, 1H); ^{13}C NMR (100 MHz, $CDCl_3$) δ 175.5, 144.9, 136.8, 135.8, 134.6, 133.6, 129.9, 128.8, 126.2, 126.1, 121.4, 117.7, 115.2, 78.4, 36.9, 35.9, 31.8, 21.5, 21.2, 19.9; IR (neat): 2923, 2854, 1768(s), 1461, 1357, 1177, 1152, 1043, 744; HRESIMS Calcd for $[C_{22}H_{21}NNaO_4S]^+$ (M + Na^+) 418.1083, found 418.1080.

10-methoxy-7-tosyl-4,5,6,7-tetrahydro-1,4-methanooxocino[5,4-*b*]indol-2(1*H*)-one (2c)

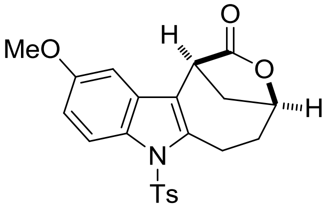

2c

Compound **2c** was prepared in 66% yield (54.3 mg) according to the general procedure (Table 2, entry 3). Pale yellow solid (mp 200-201 °C). ^{1}H NMR (400 MHz, $CDCl_3$) δ 8.11 (d, *J* = 9.2 Hz, 1H), 7.54 – 7.47 (m, 2H), 7.17 (d, *J* = 8.4 Hz, 2H), 6.92 (dd, *J* = 8.8, 2.4 Hz, 1H), 6.87 (d, *J* = 2.4 Hz, 1H), 5.10 (t, *J* = 7.2 Hz, 1H), 3.92 (dt, *J* = 17.2, 4.4 Hz, 1H), 3.83 (s, 3H), 3.78 (d, *J* = 8.0 Hz, 1H), 2.93 – 2.77 (m, 1H), 2.76 – 2.65 (m, 1H), 2.47 – 2.35 (m, 1H), 2.33 (s, 3H), 1.87 (d, *J* = 12.0 Hz, 1H), 1.85 – 1.77 (m, 1H); ^{13}C NMR (100 MHz, $CDCl_3$) δ 175.5, 156.9, 145.0, 137.5, 135.6, 130.9, 129.8, 129.6, 126.1, 121.7, 116.5, 113.6, 100.4, 78.4, 55.6, 36.9, 36.0, 31.7, 21.5, 19.9; IR (neat): 2923, 2852, 1773(s), 1475, 1458, 1356, 1150, 1087, 1042, 763, 748; HRESIMS Calcd for $[C_{22}H_{21}NNaO_5S]^+$ (M + Na^+) 434.1033, found 434.1034.

10-fluoro-7-tosyl-4,5,6,7-tetrahydro-1,4-methanooxocino[5,4-*b*]indol-2(1*H*)-one (2d)

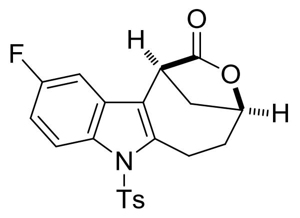

2d

Compound **2d** was prepared in 73% yield (58.3 mg) according to the general procedure (Table 2, entry 4). Pale yellow solid (mp 188-189 °C). ^{1}H NMR (400 MHz, $CDCl_3$) δ 8.26 – 8.13 (m, 1H), 7.53 (d, *J* = 8.4 Hz, 2H), 7.20 (d, *J* = 8.4 Hz, 2H), 7.11 (dd, *J* = 8.8, 2.8 Hz, 1H), 7.08 – 6.95 (m, 1H), 5.11 (t, *J* = 7.2 Hz, 1H), 3.93 (dt, *J* = 17.2, 4.0 Hz, 1H), 3.74 (d, *J* = 8.0 Hz, 1H), 2.99 – 2.79 (m, 1H), 2.77 – 2.63 (m, 1H), 2.48 – 2.37 (m, 1H), 2.35 (s, 3H), 1.89 (d, *J* = 12.4 Hz, 1H), 1.86 – 1.79 (m, 1H); ^{13}C NMR (125 MHz, $CDCl_3$) δ 175.2, 160.0 (d, *J* = 241.7 Hz), 145.4, 138.5, 135.5, 132.5, 130.0, 129.7 (d, *J* = 9.6 Hz), 126.1, 121.2 (d, *J* = 3.9 Hz), 116.7 (d, *J* = 9.1 Hz), 112.7 (d, *J* = 25.0 Hz), 103.5 (d, *J* = 24.4 Hz), 78.4, 36.8, 36.0, 31.6, 21.6, 20.0; IR (neat): 2922, 1773(s), 1458, 1376, 1358, 1265, 1176, 1150, 704; HRESIMS Calcd for $[C_{21}H_{18}FNNaO_4S]^+$ (M + Na^+) 422.0833, found 422.0836.

10-chloro-7-tosyl-4,5,6,7-tetrahydro-1,4-methanooxocino[5,4-*b*]indol-2(1*H*)-one (2e)

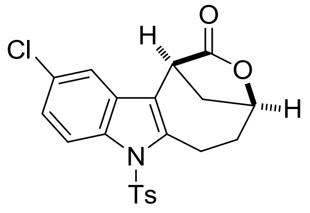

2e

Compound **2e** was prepared in 66% yield (54.7 mg) according to the general procedure (Table 2, entry 5). Pale yellow solid (mp 184-185 °C). ^{1}H NMR (400 MHz, $CDCl_3$) δ 8.16 (d, *J* = 8.8 Hz, 1H), 7.54 (d, *J* = 8.4 Hz, 2H), 7.44 (d, *J* = 2.0 Hz, 1H), 7.31 – 7.14 (m, 3H), 5.11 (t, *J* = 7.2 Hz, 1H), 3.93 (dt, *J* = 17.2, 4.4 Hz, 1H), 3.77 (d, *J* = 8.0 Hz, 1H), 2.92 – 2.78 (m, 1H), 2.77 – 2.67 (m, 1H), 2.46 – 2.36 (m, 1H), 2.35 (s, 3H), 1.88 (d, *J* = 12.4 Hz, 1H), 1.85 – 1.77 (m, 1H); ^{13}C NMR (100 MHz, $CDCl_3$) δ 175.1, 145.4, 138.3, 135.6, 134.6, 130.1, 129.8(0), 129.7(5), 126.2, 125.0, 120.7, 117.5, 116.5, 78.3, 36.8,

35.9, 31.6, 21.6, 19.9; IR (neat): 2923, 2852, 1769(s), 1450, 1377, 1355, 1159, 1043, 587; HRESIMS Calcd for $[C_{21}H_{18}ClNNaO_4S]^+$ (M + Na^+) 438.0537, found 438.0539.

10-bromo-7-tosyl-4,5,6,7-tetrahydro-1,4-methanooxocino[5,4-*b*]indol-2(1*H*)-one (2f)

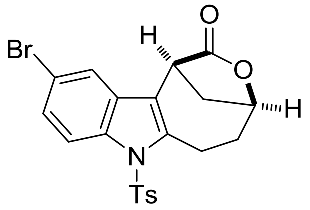

2f

Compound **2f** was prepared in 70% yield (67.5 mg) according to the general procedure (Table 2, entry 6). Pale yellow solid (mp 245-246 °C). ^{1}H NMR (400 MHz, $CDCl_3$) δ 8.11 (d, *J* = 8.8 Hz, 1H), 7.60 (d, *J* = 1.6 Hz, 1H), 7.54 (d, *J* = 8.4 Hz, 2H), 7.41 (dd, *J* = 8.8, 2.0 Hz, 1H), 7.21 (d, *J* = 8.0 Hz, 2H), 5.11 (t, *J* = 7.2 Hz, 1H), 3.93 (dt, *J* = 17.2, 4.4 Hz, 1H), 3.77 (d, *J* = 8.0 Hz, 1H), 2.92 – 2.77 (m, 1H), 2.76 – 2.66 (m, 1H), 2.46 – 2.37 (m, 1H), 2.35 (s, 3H), 1.87 (d, *J* = 12.4 Hz, 1H), 1.85 – 1.74 (m, 1H); ^{13}C NMR (100 MHz, $CDCl_3$) δ 175.1, 145.5, 138.1, 135.6, 135.0, 130.2, 130.1, 127.7, 126.2, 120.6, 120.5, 117.4, 116.8, 78.3, 36.9, 35.9, 31.6, 21.6, 19.9; IR (neat): 2292, 2850, 1768(s), 1450, 1375, 1355, 1157, 1088, 663, 584; HRESIMS Calcd for $[C_{21}H_{18}BrNNaO_4S]^+$ (M + Na^+) 482.0032, found 482.0039.

7-tosyl-10-(trifluoromethyl)-4,5,6,7-tetrahydro-1,4-methanooxocino[5,4-*b*]indol-2(1*H*)-one (2g)

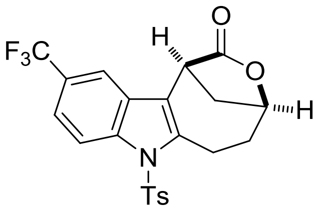

2g

Compound **2g** was prepared in 78% yield (70.0 mg) according to the general procedure (Table 2, entry 7). Pale yellow solid (mp 190-191 °C). ^{1}H NMR (400 MHz, $CDCl_3$) δ 8.36 (d, *J* = 8.8 Hz, 1H), 7.76 (s, 1H), 7.63 – 7.52 (m, 3H), 7.30 – 7.19 (m, 2H), 5.13 (t, *J* = 7.2 Hz, 1H), 3.96 (dt, *J* = 17.2, 4.4 Hz, 1H), 3.88 (d, *J* = 7.6 Hz, 1H), 2.94 – 2.82 (m,

1H), 2.81 – 2.68 (m, 1H), 2.50 – 2.39 (m, 1H), 2.36 (s, 3H), 1.91 (d, *J* = 12.4 Hz, 1H), 1.89 – 1.78 (m, 1H); ^{13}C NMR (212.5 MHz, $CDCl_3$) δ 175.1, 145.7, 138.6, 137.7, 135.5, 130.2, 128.1, 126.3 (q, *J* = 31.6 Hz), 126.2, 124.4 (q, *J* = 270.3 Hz), 121.5 (q, *J* = 3.2 Hz), 120.9, 115.6, 115.3 (q, *J* = 3.9 Hz), 78.4, 36.9, 35.8, 31.5, 21.6, 19.8; IR (neat): 2922, 2850, 1770(s), 1379, 1325, 1168, 1122, 579; HRESIMS Calcd for $[C_{22}H_{18}F_3NNaO_4S]^+$ (M + Na^+) 472.0801, found 472.0799.

2-oxo-7-tosyl-1,2,4,5,6,7-hexahydro-1,4-methanooxocino[5,4-*b*]indole-10-carbonitrile (2h)

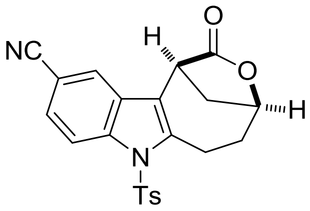

2h

Compound **2h** was prepared in 60% yield (48.7 mg) according to the general procedure (Table 2, entry 8). Pale yellow solid (mp 225-226 °C). ^{1}H NMR (400 MHz, $CDCl_3$) δ 8.35 (dd, *J* = 8.8, 0.8Hz, 1H), 7.82 (d, *J* = 1.2 Hz, 1H), 7.62 – 7.53 (m, 3H), 7.25 (d, *J* = 8.0 Hz, 2H), 5.13 (t, *J* = 7.2 Hz, 1H), 3.93 (dt, *J* = 17.2, 4.4 Hz, 1H), 3.83 (d, *J* = 7.6 Hz, 1H), 2.92 – 2.70 (m, 2H), 2.48 – 2.38 (m, 1H), 2.37 (s, 3H), 1.92 (d, *J* = 12.4 Hz, 1H), 1.91 – 1.80 (m, 1H); ^{13}C NMR (100 MHz, $CDCl_3$) δ 174.8, 146.0, 139.2, 137.9, 135.4, 130.3, 128.4, 127.7, 126.3, 122.6, 120.4, 119.0, 116.1, 107.4, 78.3, 36.7, 35.8, 31.5, 21.6, 19.9; IR (neat): 2924, 2226(s), 1769(s), 1459, 1380, 1357, 1173, 1153, 1087, 956, 668; HRESIMS Calcd for $[C_{22}H_{18}N_2NaO_4S]^+$ (M + Na^+) 429.0879, found 429.0884.

methyl 2-oxo-7-tosyl-1,2,4,5,6,7-hexahydro-1,4-methanooxocino[5,4-*b*]indole-10-carboxylate (2i)

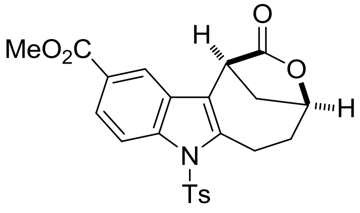

2i

Compound **2i** was prepared in 79% yield (69.4 mg) according to the general procedure (Table 2, entry 9). Pale yellow solid (mp 191-192 ^{o}C). ^{1}H NMR (400 MHz, $CDCl_3$) δ 8.28 (d, *J* = 8.8 Hz, 1H), 8.20 (d, *J* = 0.8 Hz, 1H), 8.00 (dd, *J* = 8.8, 1.2 Hz, 1H), 7.57 (d, *J* = 8.4 Hz, 2H), 7.20 (d, *J* = 8.0 Hz, 2H), 5.11 (t, *J* = 6.8 Hz, 1H), 4.04 – 3.83 (m, 5H), 2.92 – 2.80 (m, 1H), 2.79 – 2.68 (m, 1H), 2.49 – 2.27 (m, 4H), 1.90 (d, *J* = 12.4 Hz, 1H), 1.89 – 1.77 (m, 1H); ^{13}C NMR (100 MHz, $CDCl_3$) δ 175.1, 166.9, 145.5, 138.8, 138.1, 135.6, 130.1, 128.3, 126.2, 126.0, 125.8, 121.4, 119.9, 115.0, 78.3, 52.1, 36.9, 35.8, 31.6, 21.5, 19.8; IR (neat): 2924, 2851, 1773(s), 1718(s), 1274, 1160, 765; HRESIMS Calcd for $[C_{23}H_{21}NNaO_6S]^+$ (M + Na^+) 462.0982, found 462.0994.

9-methyl-7-tosyl-4,5,6,7-tetrahydro-1,4-methanooxocino[5,4-*b*]indol-2(1*H*)-one (2j)

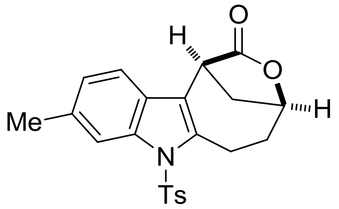

2j

Compound **2j** was prepared in 86% yield (68.0 mg) according to the general procedure (Table 2, entry 10). Pale yellow solid (mp 196-197 ^{o}C). ^{1}H NMR (400 MHz, $CDCl_3$) δ 8.05 (s, 1H), 7.53 (d, *J* = 8.4 Hz, 2H), 7.33 (d, *J* = 8.0 Hz, 1H), 7.18 (d, *J* = 8.0 Hz, 2H), 7.11 (d, *J* = 8.0 Hz, 1H), 5.09 (t, *J* = 7.2 Hz, 1H), 3.90 (dt, *J* = 17.2, 4.4 Hz, 1H), 3.81 (d, *J* = 7.6 Hz, 1H), 2.89 – 2.77 (m, 1H), 2.75 – 2.63 (m, 1H), 2.49 (s, 3H), 2.44 – 2.37 (m, 1H), 2.34 (s, 3H), 1.87 (d, *J* = 12.4 Hz, 1H), 1.86 – 1.76 (m, 1H); ^{13}C NMR (125 MHz, $CDCl_3$) δ 175.5, 145.0, 136.8, 135.9(3), 135.8(6), 135.0, 129.9, 126.3, 126.1, 125.3, 121.4, 117.3, 115.6, 78.4, 36.9, 35.9, 31.8, 22.0, 21.5, 19.8; IR (neat): 2923, 2854, 1768(s), 1461, 1357, 1177, 1152, 1043, 744; HRESIMS Calcd for $[C_{22}H_{21}NNaO_4S]^+$ (M + Na^+) 418.1083, found 418.1080.

9-methoxy-7-tosyl-4,5,6,7-tetrahydro-1,4-methanooxocino[5,4-*b*]indol-2(1*H*)-one (2k)

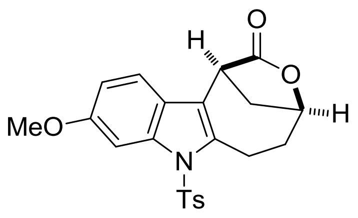

2k

Compound **2k** was prepared in 80% yield (65.8 mg) according to the general procedure (Table 2, entry 11). Pale yellow solid (mp 198-199 °C). ^{1}H NMR (500 MHz, $CDCl_3$) δ 7.81 (d, *J* = 2.0 Hz, 1H), 7.53 (d, *J* = 8.5 Hz, 2H), 7.32 (d, *J* = 8.5 Hz, 1H), 7.19 (d, *J* = 8.5 Hz, 2H), 6.92 (dd, *J* = 8.5, 2.0 Hz, 1H), 5.09 (t, *J* = 7.0 Hz, 1H), 3.94 – 3.81 (m, 4H), 3.78 (d, *J* = 8.0 Hz, 1H), 2.86 – 2.78 (m, 1H), 2.74 – 2.66 (m, 1H), 2.44 – 2.36 (m, 1H), 2.35 (m, 3H), 1.88 (d, *J* = 12.5 Hz, 1H), 1.84 – 1.76 (m, 1H); ^{13}C NMR (125 MHz, $CDCl_3$) δ 175.5, 158.1, 145.1, 137.4, 135.8, 135.2, 129.9, 126.1, 122.5, 121.4, 118.2, 112.8, 100.1, 78.5, 55.8, 36.9, 36.0, 31.8, 21.6, 19.9; IR (neat): 2924, 2852, 1774(s), 1612, 1491, 1362, 1270, 1042, 992, 584; HRESIMS Calcd for $[C_{22}H_{21}NaO_5S]^+$ (M + Na^+) 434.1033, found 434.1034.

9-fluoro-7-tosyl-4,5,6,7-tetrahydro-1,4-methanooxocino[5,4-*b*]indol-2(1*H*)-one (2l)

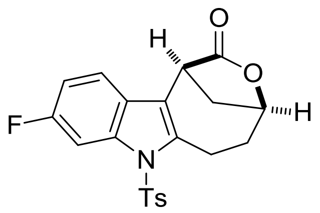

2l

Compound **2l** was prepared in 70% yield (55.9 mg) according to the general procedure (Table 2, entry 12). Pale yellow solid (mp 197-198 °C). ^{1}H NMR (400 MHz, $CDCl_3$) δ 7.99 (dd, *J* = 10.4, 2.0 Hz, 1H), 7.63 – 7.51 (m, 2H), 7.45 – 7.32 (m, 1H), 7.22 (d, *J* = 8.0 Hz, 2H), 7.12 – 6.99 (m, 1H), 5.11 (t, *J* = 7.2 Hz, 1H), 3.91 (dt, *J* = 17.2, 4.0 Hz, 1H), 3.80 (d, *J* = 7.6 Hz, 1H), 2.95 – 2.78 (m, 1H), 2.77 – 2.65 (m, 1H), 2.46 – 2.35 (m, 1H), 2.36 (s, 3H), 1.90 (d, *J* = 12.0 Hz, 1H), 1.89 – 1.78 (m, 1H); ^{13}C NMR (125 MHz, $CDCl_3$) δ 175.3, 161.0 (d, *J* = 241.4 Hz), 145.4, 136.8 (d, *J* = 4.1 Hz), 136.4 (d, *J* = 12.6 Hz), 135.6, 130.1, 126.2, 124.8, 120.8, 118.4 (d, *J* = 9.9 Hz), 112.1 (d, *J* = 24.4 Hz), 102.9 (d, *J* = 29.4 Hz), 78.4, 36.8, 36.0, 31.7, 21.6, 19.9; IR (neat): 2824, 1773(s), 1363, 1275,

1261, 993, 749; HRESIMS Calcd for $[C_{21}H_{18}FNNaO_4S]^+$ (M + Na^+) 422.0833, found 422.0836.

9-chloro-7-tosyl-4,5,6,7-tetrahydro-1,4-methanooxocino[5,4-*b*]indol-2(1*H*)-one (2m)

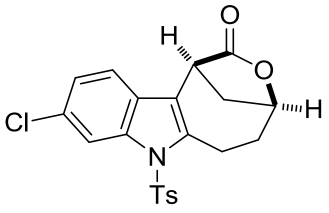

2m

Compound **2m** was prepared in 81% yield (67.2 mg) according to the general procedure (Table 2, entry 13). Pale yellow solid (mp 200-201 °C). ^1H NMR (400 MHz, $CDCl_3$) δ 8.16 (d, *J* = 8.8 Hz, 1H), 7.54 (d, *J* = 8.4 Hz, 2H), 7.44 (d, *J* = 2.0 Hz, 1H), 7.30 – 7.25 (m, 1H), 7.20 (d, *J* = 8.0 Hz, 2H), 5.11 (t, *J* = 7.2 Hz, 1H), 3.93 (dt, *J* = 17.2, 4.0 Hz, 1H), 3.77 (d, *J* = 8.0 Hz, 1H), 2.90 – 2.79 (m, 1H), 2.76 – 2.67 (m, 1H), 2.46 – 2.37 (m, 1H), 2.35 (m, 3H), 1.88 (d, *J* = 12.4 Hz, 1H), 1.87 – 1.77 (m, 1H); ^{13}C NMR (100 MHz, $CDCl_3$) δ 175.1, 145.5, 138.3, 135.6, 134.6, 130.1, 129.8(1), 129.7(5), 126.2, 125.0, 120.7, 117.5, 116.5, 78.3, 36.9, 35.9, 31.6, 21.6, 19.9; IR (neat): 2924, 1770(s), 1450, 1377, 1355, 1275, 1260, 764, 749; HRESIMS Calcd for $[C_{21}H_{18}ClNaO_4S]^+$ (M + Na^+) 438.0537, found 438.0539.

9-bromo-7-tosyl-4,5,6,7-tetrahydro-1,4-methanooxocino[5,4-*b*]indol-2(1*H*)-one (2n)

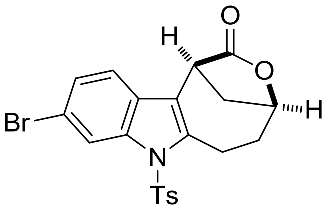

2n

Compound **2n** was prepared in 80% yield (73.4 mg) according to the general procedure (Table 2, entry 14). Pale yellow solid (mp 245-246 °C). ^1H NMR (400 MHz, $CDCl_3$) δ 8.44 (d, *J* = 1.2 Hz, 1H), 7.57 (d, *J* = 8.4 Hz, 2H), 7.40 (dd, *J* = 8.4, 1.6 Hz, 1H), 7.32 (d, *J* = 8.4 Hz, 1H), 7.23 (d, *J* = 8.0 Hz, 2H), 5.10 (t, *J* = 7.2 Hz, 1H), 3.89 (dt, *J* = 17.2, 4.4 Hz, 1H), 3.79 (d, *J* = 8.0 Hz, 1H), 2.87 – 2.67 (m, 2H), 2.46 – 2.37 (m, 1H), 2.36 (s, 3H),

1.89 (d, J = 12.4 Hz, 1H), 1.88 – 1.77 (m, 1H); ^{13}C NMR (100 MHz, $CDCl_3$) δ 175.2, 145.5, 137.2, 136.9, 135.7, 130.1, 127.3, 127.1, 126.2, 120.9, 118.8, 118.5, 118.3, 78.4, 36.8, 35.9, 31.6, 21.6, 19.9; IR (neat): 2921, 2848, 1768(s), 1450, 1370, 1355, 1155, 584; HRESIMS Calcd for $[C_{21}H_{18}BrNNaO_4S]^+$ (M + Na^+) 482.0032, found 482.0039.

methyl 2-oxo-7-tosyl-1,2,4,5,6,7-hexahydro-1,4-methanooxocino[5,4-*b*]indole-9-carboxylate (2o)

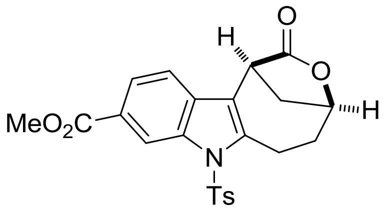

2o

Compound **2o** was prepared in 70% yield (61.5 mg) according to the general procedure (Table 2, entry 15). Pale yellow solid (mp 187-188 ^{o}C). ^{1}H NMR (400 MHz, $CDCl_3$) δ 8.93 (s, 1H), 7.97 (dd, J = 8.4, 1.2 Hz, 1H), 7.58 (d, J = 8.4 Hz, 2H), 7.49 (d, J = 8.4 Hz, 1H), 7.20 (d, J = 8.0 Hz, 2H), 5.11 (t, J = 7.2 Hz, 1H), 4.02 – 3.96 (m, 1H), 3.95 (s, 3H), 3.86 (d, J = 8.0 Hz, 1H), 2.93 – 2.80 (m, 1H), 2.78 – 2.68 (m, 1H), 2.48 – 2.37 (m, 1H), 2.33 (s, 3H), 1.90 (d, J = 12.4 Hz, 1H), 1.89 – 1.79 (m, 1H); ^{13}C NMR (100 MHz, $CDCl_3$) δ 175.2, 167.2, 145.5, 140.0, 135.6(4), 135.5(5), 131.9, 130.1, 126.6, 126.2, 125.0, 121.0, 117.4, 117.0, 78.3, 52.2, 36.8, 35.9, 31.5, 21.5, 20.0; IR (neat): 2952, 1777(s), 1716(s), 1435, 1423, 1166, 1090, 995, 736, 579; HRESIMS Calcd for $[C_{23}H_{21}NNaO_6S]^+$ (M + Na^+) 462.0982, found 462.0994.

8-methyl-7-tosyl-4,5,6,7-tetrahydro-1,4-methanooxocino[5,4-*b*]indol-2(1*H*)-one (2p)

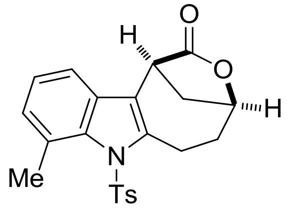

2p

Compound **2p** was prepared in 72% yield (56.9 mg) according to the general procedure (Table 2, entry 16). Pale yellow solid (mp 188-189 ^{o}C). ^{1}H NMR (400 MHz, $CDCl_3$) δ

7.23 – 7.17 (m, 1H), 7.17 – 7.09 (m, 4H), 7.07 (d, *J* = 8.0 Hz, 2H), 5.05 (t, *J* = 7.2 Hz, 1H), 3.63 – 3.48 (m, 2H), 2.95 – 2.81 (m, 1H), 2.70 (s, 3H), 2.68 – 2.54 (m, 1H), 2.40 – 2.33 (m, 1H), 2.32 (s, 3H), 1.76 – 1.67 (m, 1H), 1.65 (d, *J* = 12.4 Hz, 1H); ^{13}C NMR (100 MHz, $CDCl_3$) δ 174.8, 144.7, 140.9, 139.4, 133.2, 133.0, 130.2, 129.1, 128.9, 127.1, 126.3, 125.5, 115.4, 78.4, 36.6, 35.8, 31.5, 22.5, 21.6, 21.5; IR (neat): 2923, 2854, 1770(s), 1462, 1357, 1180, 1152, 1043, 754; HRESIMS Calcd for $[C_{22}H_{21}NNaO_4S]^+$ (M + Na^+) 418.1083, found 418.1080.

7-((4-bromophenyl)sulfonyl)-4,5,6,7-tetrahydro-1,4-methanooxocino[5,4-*b*]indol-2(1*H*)-one (2q)

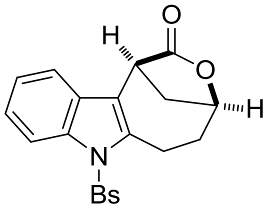

2q

Compound **2q** was prepared in 83% yield (73.8 mg) according to the general procedure (Table 2, entry 17). Pale yellow solid (mp 201-202 ^{o}C). ^{1}H NMR (400 MHz, $CDCl_3$) δ 8.19 (dd, *J* = 7.2, 1.2 Hz, 1H), 7.57 – 7.44 (m, 5H), 7.37 – 7.27 (m, 2H), 5.12 (t, *J* = 7.2 Hz, 1H), 3.92 (dt, *J* = 17.2, 4.4 Hz, 1H), 3.86 (d, *J* = 7.6 Hz, 1H), 2.97 – 2.83 (m, 1H), 2.80 – 2.67 (m, 1H), 2.51 – 2.37 (m, 1H), 1.90 (d, *J* = 12.4 Hz, 1H), 1.88 – 1.79 (m, 1H); ^{13}C NMR (100 MHz, $CDCl_3$) δ 175.3, 137.4, 136.6, 136.2, 132.6, 129.2, 128.7, 127.5, 125.2, 124.3, 122.3, 117.9, 115.4, 78.3, 36.8, 35.9, 31.7, 19.9; IR (neat): 2921, 2850, 1768(s), 1450, 1375, 1355, 1157, 1088, 584; HRESIMS Calcd for $[C_{20}H_{16}BrNNaO_4S]^+$ (M + Na^+) 467.9876, found 467.9880.

7-(methylsulfonyl)-4,5,6,7-tetrahydro-1,4-methanooxocino[5,4-*b*]indol-2(1*H*)-one (2r)

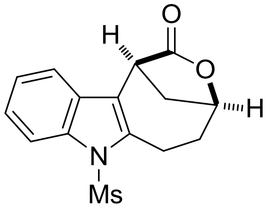

2r

Compound **2r** was prepared in 80% yield (48.8 mg) according to the general procedure (Table 2, entry 18). Pale yellow solid (mp 171-172 °C). ^{1}H NMR (400 MHz, $CDCl_3$) δ 8.08 – 7.99 (m, 1H), 7.60 – 7.51 (m, 1H), 7.38 – 7.29 (m, 2H), 5.16 (t, *J* = 7.2 Hz, 1H), 3.98 – 3.86 (m, 2H), 3.05 – 2.94 (m, 4H), 2.85 – 2.72 (m, 1H), 2.52 – 2.41 (m, 1H), 2.00 (d, *J* = 12.0 Hz, 1H), 1.99 – 1.89 (m, 1H); ^{13}C NMR (100 MHz, $CDCl_3$) δ 175.6, 136.6, 135.8, 128.4, 125.0, 124.0, 121.1, 117.9, 114.7, 78.5, 41.2, 36.8, 35.9, 31.7, 19.5; IR (neat): 2927, 2853, 1763(s), 1451, 1358, 1204, 1148, 974, 747; HRESIMS Calcd for $[C_{15}H_{15}NNaO_4S]^+$ (M + Na^+) 328.0614, found 328.0618.

5-methyl-7-tosyl-4,5,6,7-tetrahydro-1,4-methanooxocino[5,4-*b*]indol-2(1*H*)-one (2s)

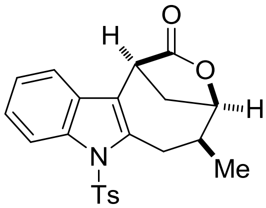

2s

Compound **2s** was prepared in 63% yield (49.8 mg; dr = 4:1) according to the general procedure (Table 2, entry 19). Pale yellow solid (mp 185-186 °C). ^{1}H NMR (500 MHz, $CDCl_3$) δ 8.23 (d, *J* = 8.0 Hz, 1H), 7.54 (d, *J* = 8.5 Hz, 2H), 7.44 (d, *J* = 7.5 Hz, 1H), 7.37 – 7.24 (m, 2H), 7.18 (d, *J* = 8.0 Hz, 2H), 4.80 (d, *J* = 8.0 Hz, 1H), 3.91 (dd, *J* = 17.0, 4.0 Hz, 1H), 3.81 (d, *J* = 7.5 Hz, 1H), 2.78 – 2.68 (m, 1H), 2.66 – 2.56 (m, 1H), 2.34 (s, 3H), 2.08 – 1.96 (m, 1H), 1.88 (d, *J* = 12.5 Hz, 1H), 1.25 (d, *J* = 7.0 Hz, 3H); ^{13}C NMR (125 MHz, $CDCl_3$) δ 175.6, 145.1, 136.2, 135.7, 135.4, 129.9, 128.4, 126.1, 124.9, 123.9, 121.9, 117.6, 115.5, 84.3, 37.9, 37.5, 35.4, 27.7, 21.5(4), 21.4(9); IR (neat): 2923, 2850, 1766(s), 1451, 1364, 1170, 1106, 1088, 747, 579; HRESIMS Calcd for $[C_{22}H_{21}NNaO_4S]^+$ (M + Na^+) 418.1083, found 418.1083.

4-phenyl-7-tosyl-4,5,6,7-tetrahydro-1,4-methanooxocino[5,4-*b*]indol-2(1*H*)-one (2t)

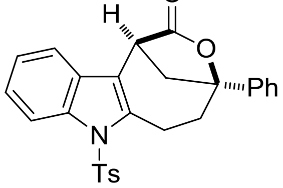

2t

Compound **2t** was prepared in 68% yield (62.2 mg) according to the general procedure (Table 2, entry 20). Pale yellow solid (mp 170-171 °C). ^{1}H NMR (400 MHz, $CDCl_3$) δ 8.27 (d, *J* = 8.0 Hz, 1H), 7.58 (d, *J* = 7.6 Hz, 2H), 7.48 (d, *J* = 7.2 Hz, 1H), 7.45 – 7.27 (m, 7H), 7.20 (d, *J* = 8.4 Hz, 2H), 4.19 – 3.93 (m, 2H), 3.05 – 2.86 (m, 1H), 2.80 – 2.65 (m, 1H), 2.50 – 2.30 (m, 5H), 2.25 – 2.13 (m, 1H); ^{13}C NMR (100 MHz, $CDCl_3$) δ 174.5, 145.2, 136.2, 135.8, 129.9, 128.8, 128.5, 127.8, 126.2, 124.9, 123.9, 123.5, 121.3, 117.6, 115.4, 88.3, 43.3, 39.7, 37.7, 21.5, 21.0; IR (neat): 2922, 1774(s), 1449, 1363, 1192, 1171, 1090, 748; HRESIMS Calcd for $[C_{27}H_{23}NNaO_4S]^+$ (M + Na^+) 480.1240, found 480.1244.

9-methyl-4-phenyl-7-tosyl-4,5,6,7-tetrahydro-1,4-methanooxocino[5,4-*b*]indol-2(1*H*)-one (2u)

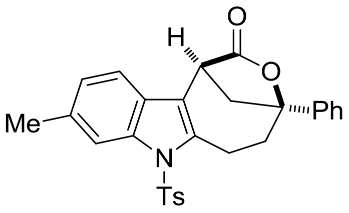

2u

Compound **2u** was prepared in 75% yield (70.7 mg) according to the general procedure (Table 2, entry 21). Pale yellow solid (mp 205-206 °C). ^{1}H NMR (500 MHz, $CDCl_3$) δ 8.09 (s, 1H), 7.57 (d, *J* = 8.5 Hz, 2H), 7.44 – 7.29 (m, 6H), 7.21 (d, *J* = 8.0 Hz, 2H), 7.13 (d, *J* = 8.0 Hz, 1H), 4.08 – 3.98 (m, 2H), 3.00 – 2.81 (m, 1H), 2.77 – 2.63 (m, 1H), 2.51 (s, 3H), 2.42 – 2.35 (m, 4H), 2.32 (d, *J* = 12.5 Hz, 1H), 2.20 – 2.11 (m, 1H); ^{13}C NMR (125 MHz, $CDCl_3$) δ 174.5, 145.2, 145.0, 136.7, 136.0, 135.4, 135.1, 129.9, 128.7, 127.7, 126.3, 126.1, 125.3, 123.5, 121.3, 117.2, 115.6, 88.3, 43.3, 39.8, 37.8, 22.0, 21.5, 21.0; IR (neat): 2921, 1775(s), 1448, 1363, 1171, 1148, 1090, 701, 582; HRESIMS Calcd for $[C_{28}H_{25}NNaO_4S]^+$ (M + Na^+) 494.1397, found 494.1400.

10-chloro-4-phenyl-7-tosyl-4,5,6,7-tetrahydro-1,4-methanooxocino[5,4-*b*]indol-2(1*H*)-one (2v)

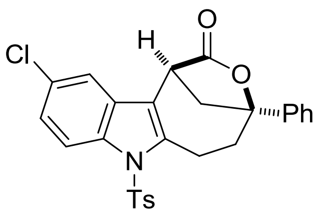

2v

Compound **2v** was prepared in 60% yield (58.9 mg) according to the general procedure (Table 2, entry 22). Pale yellow solid (mp 188-189 °C). ^{1}H NMR (500 MHz, $CDCl_3$) δ 8.19 (d, *J* = 9.0 Hz, 1H), 7.57 (d, *J* = 8.5 Hz, 2H), 7.46 (d, *J* = 2.0 Hz, 1H), 7.43 – 7.31 (m, 5H), 7.30 (dd, *J* = 9.0, 2.0 Hz, 1H), 7.23 (d, *J* = 8.0 Hz, 2H), 4.07 (dt, *J* = 17.0, 4.0 Hz, 1H), 3.99 (d, *J* = 8.0 Hz, 1H), 2.98 – 2.88 (m, 1H), 2.76 – 2.68 (m, 1H), 2.45 – 2.38 (m, 1H), 2.37 (s, 3H), 2.33 (d, *J* = 12.0 Hz, 1H), 2.23 – 2.13 (m, 1H); ^{13}C NMR (125 MHz, $CDCl_3$) δ 174.1, 145.5, 145.0, 137.8, 135.5, 134.5, 130.1, 129.8, 129.7, 128.8, 127.9, 126.2, 125.0, 123.4, 120.5, 117.5, 116.5, 88.3, 43.2, 39.6, 37.7, 21.6, 21.1; IR (neat): 2922, 1777(s), 1451, 1364, 1171, 1090, 574; HRESIMS Calcd for $[C_{27}H_{22}ClNNaO_4S]^+$ (M + Na^+) 514.0850, found 514.0853.

10-bromo-4-phenyl-7-tosyl-4,5,6,7-tetrahydro-1,4-methanooxocino[5,4 *b*]indol-2(1*H*)-one (2w)

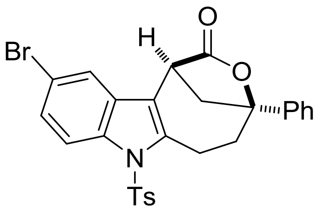

2w

Compound **2w** was prepared in 66% yield (70.6 mg) according to the general procedure (Table 2, entry 23). Pale yellow solid (mp 230-231 °C). ^{1}H NMR (500 MHz, $CDCl_3$) δ 8.14 (d, *J* = 9.0 Hz, 1H), 7.62 (d, *J* = 2.0 Hz, 1H), 7.57 (d, *J* = 8.5 Hz, 2H), 7.46 – 7.30 (m, 6H), 7.23 (d, *J* = 8.5 Hz, 2H), 4.07 (dt, *J* = 17.0, 4.0 Hz, 1H), 3.99 (d, *J* = 8.0 Hz, 1H), 2.97 – 2.87 (m, 1H), 2.75 –2.68 (m, 1H), 2.46 – 2.38 (m, 1H), 2.37 (s, 3H), 2.33 (d, *J* = 12.5 Hz, 1H), 2.22 – 2.14 (m, 1H); ^{13}C NMR (125 MHz, $CDCl_3$) δ 174.0, 145.5, 145.0, 137.7, 135.6, 135.0, 130.1, 128.8, 127.9, 127.7, 126.2, 123.4, 120.5, 120.4, 117.5, 116.9, 88.3, 43.3, 39.6, 37.7, 21.6, 21.1; IR (neat): 2291, 1777(s), 1450, 1378, 1359, 1192, 1168,

1090, 590; HRESIMS Calcd for $[C_{27}H_{22}BrNNaO_4S]^+$ (M + Na^+) 558.0345, found 558.0356.

4-(*p*-tolyl)-7-tosyl-4,5,6,7-tetrahydro-1,4-methanooxocino[5,4-*b*]indol-2(1*H*)-one (2x)

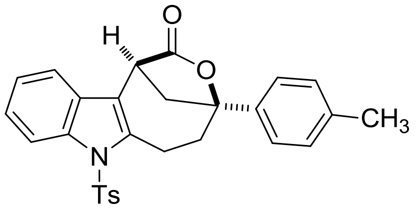

2x

Compound **2x** was prepared in 64% yield (60.3 mg) according to the general procedure (Table 2, entry 24). Pale yellow solid (mp 200-201 °C). ^1H NMR (400 MHz, $CDCl_3$) δ 8.27 (d, J = 7.6 Hz, 1H), 7.58 (d, J = 8.4 Hz, 2H), 7.53 – 7.44 (m, 1H), 7.40 – 7.12 (m, 8H), 4.16 – 3.98 (m, 2H), 3.03 – 2.87 (m, 1H), 2.78 – 2.64 (m, 1H), 2.49 – 2.27 (m, 8H), 2.22 – 2.07 (m, 1H); ^{13}C NMR (100 MHz, $CDCl_3$) δ 174.6, 145.1, 142.4, 137.5, 136.3, 135.8, 129.9, 129.4, 128.5, 126.2, 125.0, 123.9, 123.5, 121.4, 117.7, 115.5, 88.4, 43.4, 39.8, 37.8, 21.5, 21.0; IR (neat): 2921, 1770(s), 1450, 1365, 1171, 1150, 1090, 701, 575; HRESIMS Calcd for $[C_{28}H_{25}NNaO_4S]^+$ (M + Na^+) 494.1397, found 494.1400.

4-(4-chlorophenyl)-7-tosyl-4,5,6,7-tetrahydro-1,4-methanooxocino[5,4-*b*]indol-2(1*H*)-one (2y)

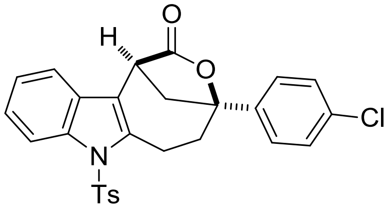

2y

Compound **2y** was prepared in 61% yield (59.9 mg) according to the general procedure (Table 2, entry 25). Pale yellow solid (mp 190-191 °C). ^1H NMR (400 MHz, $CDCl_3$) δ 8.26 (dd, J = 7.2, 0.4 Hz, 1H), 7.58 (d, J = 8.4 Hz, 2H), 7.51 – 7.44 (m, 1H), 7.40 – 7.28 (m, 6H), 7.20 (d, J = 8.0 Hz, 2H), 4.15 – 4.01 (m, 2H), 3.02 – 2.82 (m, 1H), 2.74 – 2.60 (m, 1H), 2.43 – 2.26 (m, 5H), 2.23 – 2.07 (m, 1H); ^{13}C NMR (100 MHz, $CDCl_3$) δ 174.2, 145.2, 143.6, 136.2, 136.1, 135.8, 133.7, 130.0, 129.0, 128.4, 126.2, 125.0, 123.9, 121.1,

117.6, 115.5, 87.8, 43.3, 39.6, 37.7, 21.6, 20.9; IR (neat): 2930, 1780(s), 1450, 1360, 1171, 1090, 585; HRESIMS Calcd for $[C_{27}H_{22}ClNNaO_4S]^+$ (M + Na^+) 514.0850, found 514.0853.

4-methyl-7-tosyl-4,5,6,7-tetrahydro-1,4-methanooxocino[5,4-*b*]indol-2(1*H*)-one (2z)

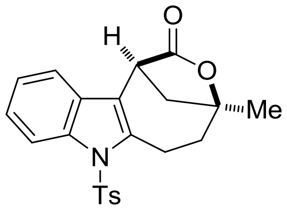

2z

Compound **2z** was prepared in 67% yield (52.9 mg) according to the general procedure (Table 2, entry 26). Pale yellow solid (mp 221-222 °C). ^{1}H NMR (400 MHz, $CDCl_3$) δ 8.26 (d, *J* = 7.6 Hz, 1H), 7.57 (d, *J* = 8.0 Hz, 2H), 7.47 (d, *J* = 7.2 Hz, 1H), 7.38 – 7.25 (m, 2H), 7.20 (d, *J* = 8.4 Hz, 2H), 4.06 – 3.93 (m, 2H), 2.90 – 2.78 (m, 1H), 2.50 – 2.40 (m, 1H), 2.35 (s, 3H), 2.30 – 2.22 (m, 1H), 1.93 (d, *J* = 12.4 Hz, 1H), 1.88 – 1.77 (m, 1H), 1.59 (s, 3H); ^{13}C NMR (125 MHz, $CDCl_3$) δ 175.0, 145.1, 136.3, 136.2, 135.7, 129.9, 128.5, 126.2, 124.8, 123.8, 121.3, 117.6, 115.4, 86.0, 43.1, 38.2, 38.1, 29.1, 21.6, 20.8; IR (neat): 2923, 2851, 1768(s), 1451, 1364, 1170, 1106, 1088, 748, 579; HRESIMS Calcd for $[C_{22}H_{21}NNaO_4S]^+$ (M + Na^+) 418.1083, found 418.1080.

1,4,5,6-tetrahydro-2*H*-1,4-methanooxocino[5,4-*b*]benzofuran-2-one (4a)

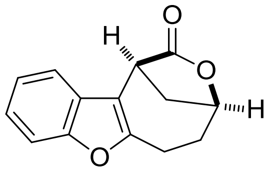

4a

Compound **4a** was prepared in 61% yield (27.8 mg) according to the general procedure. Pale yellow solid (mp 154-155 °C). ^{1}H NMR (400 MHz, $CDCl_3$) δ 7.56 – 7.47 (m, 1H), 7.44 – 7.36 (m, 1H), 7.32 – 7.17 (m, 2H), 5.15 (t, *J* = 7.2 Hz, 1H), 3.81 (d, *J* = 7.6 Hz, 1H), 3.18 – 2.98 (m, 2H), 2.87 – 2.76 (m, 1H), 2.51 – 2.40 (m, 1H), 2.03 (d, *J* = 12.4 Hz, 1H), 2.00 – 1.89 (m, 1H); ^{13}C NMR (100 MHz, $CDCl_3$) δ 175.5, 154.5, 153.4, 127.6, 123.9, 122.8, 118.1, 115.2, 110.9, 78.3, 37.2, 34.9, 30.0, 22.0; IR (neat): 2948, 1768(s),

1453, 1274, 1258, 1155, 1040, 749; HRESIMS Calcd for $[C_{14}H_{12}NaO_3]^+$ (M + Na^+) 251.0679, found 251.0679.

1-tosyl-4,7,8,9-tetrahydro-4,7-methanooxocino[5,4-*b*]pyrrol-5(1*H*)-one (4b)

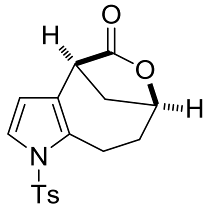

4b

Compound **4b** was prepared in 66% yield (43.7 mg) according to the general procedure. Pale yellow oil. ^{1}H NMR (400 MHz, $CDCl_3$) δ 7.61 (d, *J* = 8.4 Hz, 2H), 7.29 (d, *J* = 8.4 Hz, 2H), 7.24 (d, *J* = 3.2 Hz, 1H), 6.16 (d, *J* = 3.6 Hz, 1H), 5.01 (t, *J* = 7.2 Hz, 1H), 3.55 – 3.38 (m, 2H), 2.67 – 2.56 (m, 1H), 2.53 – 2.39 (m, 4H), 2.34 – 2.23 (m, 1H), 1.88 (d, *J* = 12.4 Hz, 1H), 1.79 – 1.66 (m, 1H); ^{13}C NMR (100 MHz, $CDCl_3$) δ 175.9, 145.3, 135.9, 130.2, 129.7, 126.8, 125.8, 121.3, 111.9, 78.4, 39.3, 37.4, 31.6, 21.6, 18.9; IR (neat):

2923, 2851, 1768(s), 1451, 1364, 1170, 1106, 1088, 748, 579; HRESIMS Calcd for $[C_{17}H_{17}NNaO_4S]^+$ (M + Na^+) 354.0770, found 354.0779.

8,9-dimethoxy-1,4,5,6-tetrahydro-2*H*-1,4-methanobenzo[*d*]oxocin-2-one (4c)

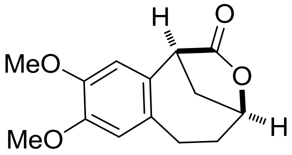

4c

Compound **4c** was prepared in 54% yield (26.8 mg) according to the general procedure. Pale yellow solid (mp 241-242 ^{o}C). ^{1}H NMR (400 MHz, $CDCl_3$) δ 6.69 (s, 1H), 6.68 (s, 1H), 5.06 (t, *J* = 7.2 Hz, 1H), 3.85 (s, 3H), 3.84 (s, 3H), 3.54 (d, *J* = 8.8 Hz, 1H), 3.18 – 3.06 (m, 1H), 2.78 – 2.60 (m, 2H), 2.40 – 2.29 (m, 1H), 1.92 (d, *J* = 12.4 Hz, 1H), 1.90–1.80 (m, 1H); ^{13}C NMR (100 MHz, $CDCl_3$) δ 177.3, 147.5, 147.2, 131.3, 131.1, 114.7, 113.5, 78.7, 56.0, 55.9, 46.9, 38.2, 33.4, 29.1; IR (neat): 2936, 1766(s), 1515, 1453, 1276,

1252, 1103, 749; HRESIMS Calcd for $[C_{14}H_{16}NaO_4]^+$ (M + Na^+) 271.0941, found 271.0943.

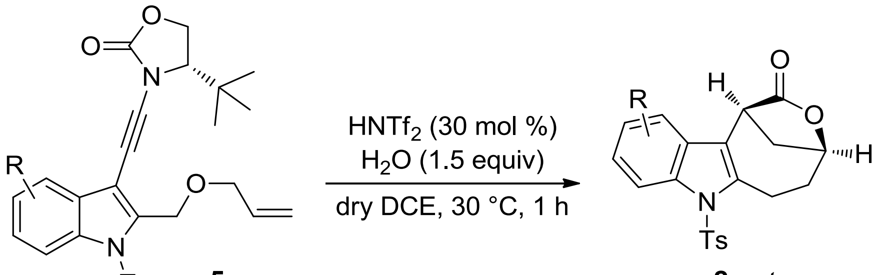

5 **2-*ent***

General procedure for the synthesis of chiral bridged [4.2.1] lactones 2-*ent*:

$HNTf_2$ (0.06 mmol, 16.8 mg) was added to a mixture of the chiral ynamide **5** (0.20 mmol), water (0.30 mmol, 5.4 uL) and dry DCE (2.0 mL) at room temperature. Then, the reaction mixture was stirred at 30 ℃ and the progress of the reaction was monitored by TLC. The reaction typically took 1 h. Upon completion, the mixture was concentrated and the residue was purified by chromatography on silica gel (eluent: hexanes/ethyl acetate) to afford the desired chiral bridged [4.2.1] lactone **2**-*ent*.

(1*S*,4*S*)-7-tosyl-4,5,6,7-tetrahydro-1,4-methanooxocino[5,4-*b*]indol-2(1*H*)-one (2a-*ent*)

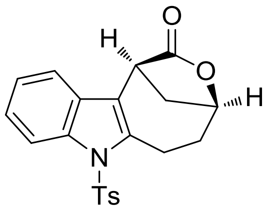

2a-*ent*

Compound **2a**-*ent* was prepared in 55% yield (41.9 mg) according to the general procedure (Table 3, emtry 1). 95% ee (determined by HPLC: Chiralcel OD-H Column, 10/90 *i*-PrOH/hexane, 1.0 mL/min, 200 nm; TR = 27.21 min (minor), 33.53 min (major)).

(1*S*,4*S*)-10-methyl-7-tosyl-4,5,6,7-tetrahydro-1,4-methanooxocino[5,4-*b*]indol-2(1*H*)-one (2b-*ent*)

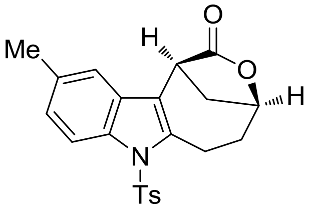

2b-*ent*

Compound **2b**-*ent* was prepared in 50% yield (35.9 mg) according to the general procedure (Table 3, emtry 2). 99% ee (determined by HPLC: Chiralcel OD-H Column, 15/85 *i*-PrOH/hexane, 1.3 mL/min, 200 nm; TR = 15.57 min (major), 21.21 min (minor)).

(1*S*,4*S*)-10-chloro-7-tosyl-4,5,6,7-tetrahydro-1,4-methanooxocino[5,4-*b*]indol-2(1*H*)-one (2e-*ent*)

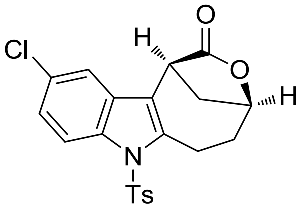

2e-*ent*

Compound **2c**-*ent* was prepared in 52% yield (43.2 mg) according to the general procedure (Table 3, emtry 3). 91% ee (determined by HPLC: Chiralcel OD-H Column, 5/95 *i*-PrOH/hexane, 1.3 mL/min, 200 nm; TR = 33.00 min (minor), 36.73 min (major)).

(1*S*,4*S*)-10-bromo-7-tosyl-4,5,6,7-tetrahydro-1,4-methanooxocino[5,4-*b*]indol-2(1*H*)-one (2f-*ent*)

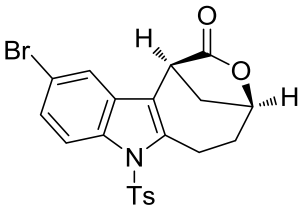

2f-*ent*

Compound **2f**-*ent* was prepared in 53% yield (48.6 mg) according to the general procedure (Table 3, emtry 4). 98% ee (determined by HPLC: Chiralcel OD-H Column, 5/95 *i*-PrOH/hexane, 1.3 mL/min, 200 nm; TR = 34.94 min (minor), 39.54 min (major)).

methyl (1*S*,4*S*)-2-oxo-7-tosyl-1,2,4,5,6,7-hexahydro-1,4-methanooxocino[5,4-*b*]indole-10-carboxylate (2i-*ent*)

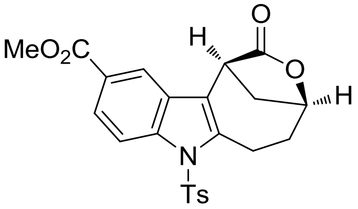

2i-*ent*

Compound **2i**-*ent* was prepared in 51% yield (44.8 mg) according to the general procedure (Table 3, emtry 5). $[\alpha]_D^{20}$ = -40.7 °(c = 1.0, $CHCl_3$). 92% ee (determined by HPLC: Chiralcel IA Column, 10/90 *i*-PrOH/hexane, 2.0 mL/min, 200 nm; TR = 17.49 min (minor), 21.05 min (major)).

(1*S*,4*S*)-9-methyl-7-tosyl-4,5,6,7-tetrahydro-1,4-methanooxocino[5,4-*b*]indol-2(1*H*)-one (2j-*ent*)

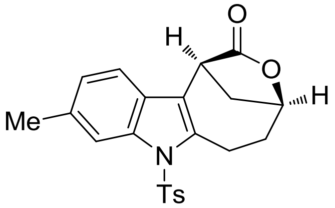

2j-*ent*

Compound **2j**-*ent* was prepared in 42% yield (33.2 mg) according to the general procedure (Table 3, emtry 6). 98% ee (determined by HPLC: Chiralcel OD-H Column, 15/85 *i*-PrOH/hexane, 1.3 mL/min, 200 nm; TR = 31.00 min (minor), 34.39 min (major)).

(1*R*,4*R*)-10-methyl-7-tosyl-4,5,6,7-tetrahydro-1,4-methanooxocino[5,4-*b*]indol-2(1*H*)-one (2a-*ent'*)

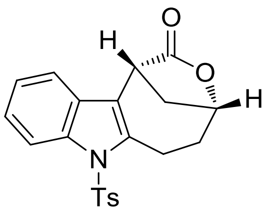

2a-*ent'*

Compound **2a**-*ent'* was prepared in 56% yield (42.7 mg) according to the general procedure (Table 3, emtry 7). $[\alpha]_D^{20}$ = +10.7 °(c = 1.0, $CHCl_3$). 96% ee (determined by HPLC: Chiralcel OD-H Column, 10/90 *i*-PrOH/hexane, 1.0 mL/min, 200 nm; TR = 25.17 min (major), 32.09 min (minor)).

4,5,6,7-tetrahydro-1,4-methanooxocino[5,4-*b*]indol-2(1*H*)-one (2ab)

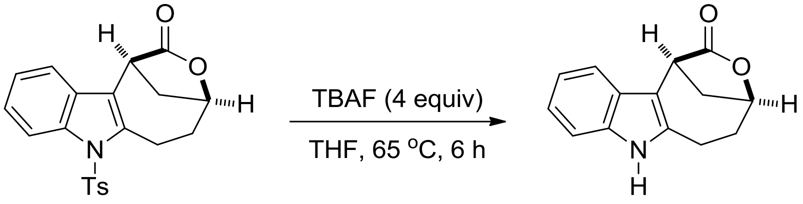

2a **2ab**, 75%

Compound **2ab** was prepared in 75% yield (34.1 mg) according to the known procedures (0.2 mmol scale).[5] Pale yellow solid (mp 143-144 °C). ^{1}H NMR (400 MHz, DMSO) δ 11.04 (s, 1H), 7.54 (d, *J* = 7.2 Hz, 1H), 7.31 – 7.25 (m, 1H), 7.12 – 6.67 (m, 2H), 5.14 (t, *J* = 7.2 Hz, 1H), 3.95 (d, *J* = 7.6 Hz, 1H), 3.00 – 2.69 (m, 3H), 2.39 – 2.26 (m, 1H), 1.99 – 1.91 (m, 1H), 1.89 (d, *J* = 12.4 Hz, 1H); ^{13}C NMR (125 MHz, DMSO) δ 177.7, 136.7, 135.1, 127.9, 121.6, 119.8, 118.1, 111.7, 111.6, 79.2, 38.1, 36.5, 31.7, 22.3; IR (neat):3270, 2923, 2851, 1760(s), 1515, 1455, 1360, 1172, 952, 742; HRESIMS Calcd for $[C_{14}H_{13}NNaO_2]^+$ (M + Na^+) 250.0838, found 250.0839.

7-tosyl-1,2,4,5,6,7-hexahydro-1,4-methanooxocino[5,4-*b*]indol-2-ol (2ac)

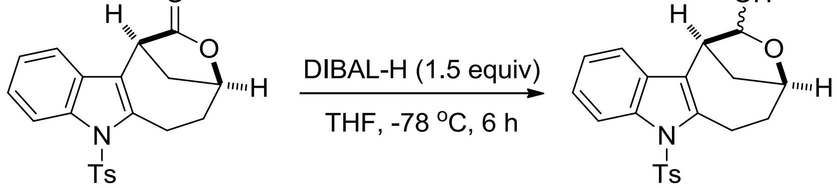

2a **2ac**, 91% (dr >10:1)

Compound **2ac** was prepared in 91% yield (69.7 mg; dr > 10/1) according to the known procedures (0.2 mmol scale).[6] Pale yellow oil. ^{1}H NMR (400 MHz, $CDCl_3$) δ 8.25 (dd, *J* = 7.2, 1.2 Hz, 1H), 7.54 (d, *J* = 8.0 Hz, 2H), 7.48 – 7.41 (m, 1H), 7.34 – 7.22 (m, 2H), 7.16 (d, *J* = 8.0 Hz, 2H), 5.24 (d, *J* = 2.8 Hz, 1H), 4.87 (t, *J* = 7.2 Hz, 1H), 3.62 (dt, *J* =

17.2, 4.4 Hz, 1H), 3.46 (d, J = 6.4 Hz, 1H), 3.12 (s, 1H), 3.05 – 2.90 (m, 1H), 2.72 – 2.59 (m, 1H), 2.33 (s, 3H), 2.23 – 2.09 (m, 1H), 1.67 (d, J = 12.0 Hz, 1H), 1.60 – 1.48 (m, 1H); ^{13}C NMR (100 MHz, $CDCl_3$) δ 144.7, 137.2, 136.5, 136.1, 129.7, 129.6, 126.2, 124.4, 123.6, 123.3, 117.7, 115.5, 102.9, 78.6, 41.3, 35.6, 32.2, 21.5, 20.9; IR (neat): 2930, 2851, 1750(s), 1450, 1250, 1170, 568; HRESIMS Calcd for $[C_{21}H_{21}NNaO_4S]^+$ (M + Na^+) 406.1083, found 406.1095.

7-tosyl-1,2,4,5,6,7-hexahydro-1,4-methanooxocino[5,4-*b*]indole (2ad)

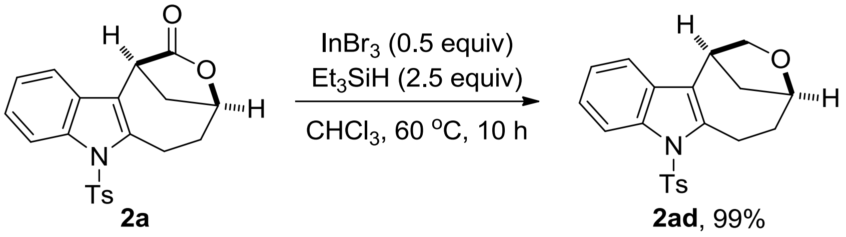

Compound **2ad** was prepared in 99% yield (72.7 mg) according to the known procedures (0.2 mmol scale).[7] Pale yellow solid (mp 150-151 °C). ^{1}H NMR (400 MHz, $CDCl_3$) δ 8.24 (dd, J = 7.2, 1.6 Hz, 1H), 7.54 (d, J = 8.4 Hz, 2H), 7.43 – 7.36 (m, 1H), 7.32 – 7.20 (m, 2H), 7.14 (d, J = 8.4 Hz, 2H), 4.69 (t, J = 7.6 Hz, 1H), 4.12 (dd, J = 7.6, 5.6 Hz, 1H), 3.86 (d, J = 7.6 Hz, 1H), 3.65 (dt, J = 16.8, 4.4 Hz, 1H), 3.56 (t, J = 6.0 Hz, 1H), 3.13 – 2.95 (m, 1H), 2.41 – 2.22 (m, 4H), 2.19 – 2.08 (m, 1H), 1.73 (d, J = 11.6 Hz, 1H), 1.58 – 1.43 (m, 1H); ^{13}C NMR (100 MHz, $CDCl_3$) δ 144.5, 137.0, 136.3, 136.0, 129.6, 129.5, 127.1, 126.1, 124.0, 123.4, 117.3, 115.5, 77.2, 75.6, 38.7, 34.0, 32.1, 21.4, 20.7; IR (neat): 2923, 1451, 1376, 1361, 1176, 1089, 1060, 748, 579; HRESIMS Calcd for $[C_{21}H_{21}NNaO_3S]^+$ (M + Na^+) 390.1134, found 390.1141.

7-tosyl-4,7-dihydro-1,4-methanooxocino[5,4-*b*]indol-2(1*H*)-one (2ae)

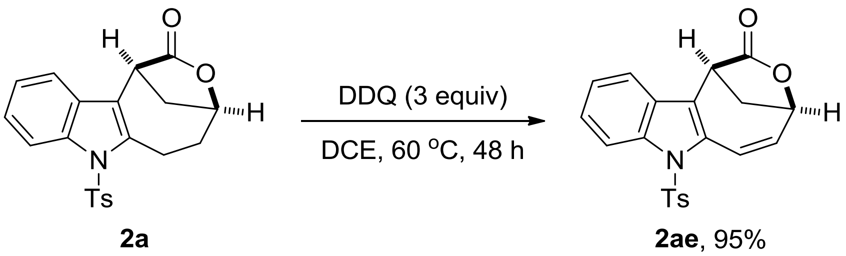

Compound **2ae** was prepared in 95% yield (72.0 mg) according to the known procedures (0.2 mmol scale).[8] Pale yellow solid (mp 187-188 °C). ^{1}H NMR (400 MHz, $CDCl_3$) δ 8.26 (d, *J* = 8.4 Hz, 1H), 7.73 (dd, *J* = 12.0, 0.8 Hz, 1H), 7.68 – 7.48 (m, 3H), 7.46 – 7.29 (m, 2H), 7.16 (d, *J* = 8.0 Hz, 2H), 6.45 – 6.22 (m, 1H), 5.09 (t, *J* = 7.2 Hz, 1H), 4.07 (d, *J* = 7.2 Hz, 1H), 2.85 – 2.67 (m, 1H), 2.31 (s, 3H), 2.14 – 1.97 (m, 1H); ^{13}C NMR (100 MHz, $CDCl_3$) δ 172.9, 145.2, 137.1, 135.0, 132.9, 129.8, 128.9, 128.5, 126.7, 126.3, 124.6, 123.3, 122.9, 119.0, 115.8, 73.7, 36.1, 27.0, 21.5; IR (neat): 2922, 1763(s), 1449, 1364, 1275, 1261, 1172, 764, 749; HRESIMS Calcd for $[C_{21}H_{17}NNaO_4S]^+$ (M + Na^+) 402.0770, found 402.0777.

(7-hydroxy-5-tosyl-5,6,7,8,9,10-hexahydrocyclohepta[*b*]indol-10-yl)(phenyl)methanone (2af)

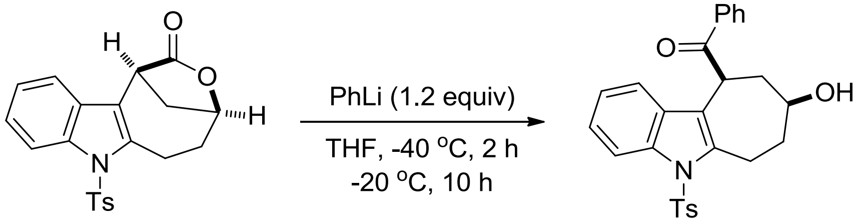

2a **2af**, 60%

PhLi (0.24 mmol, 1.2 qeuiv) was slowly added to the mixture of **2a** (0.20 mmol, 76.3 mg) in dry THF (4.0 mL) under N_2 at -40 ℃. The mixture was stired at -40 ℃ for 2 h, and then stired at -20 ℃ for amother 10 h. Upon completion, the mixture was quenched by the saturated ammonium chloride and extracted with EtOAc (3 × 10 mL). The combined extracts were dried with $MgSO_4$, and the solvent was removed under reduced pressure. The residue was purified by chromatography on silica gel (eluent: hexanes/ethyl acetate) to afford the desired **2af** in 60% yield (55.1 mg). Pale yellow oil. ^{1}H NMR (400 MHz, $CDCl_3$) δ 8.22 (d, *J* = 8.4 Hz, 1H), 8.08 – 7.93 (m, 2H), 7.65 – 7.54 (m, 3H), 7.51 – 7.41 (m, 2H), 7.26 – 7.13 (m, 5H), 5.11 (dd, *J* = 5.2, 4.0 Hz, 1H), 4.07 – 3.93 (m, 1H), 3.75 – 3.63 (m, 1H), 3.19 – 3.00 (m, 1H), 2.60 – 2.49 (m, 1H), 2.34 (s, 3H), 2.26 – 2.12 (m, 1H), 2.03 – 1.88 (m, 1H), 1.60 – 1.47 (m, 2H); ^{13}C NMR (125 MHz, $CDCl_3$) δ 199.0, 144.6, 141.0, 136.3, 136.1(2), 136.0(8), 133.3, 129.9, 129.8, 128.9, 128.3, 126.2, 124.2, 123.5, 119.9, 117.4, 115.6, 69.5, 40.4, 37.7, 35.6, 21.6, 21.3; IR (neat): 2924, 2853, 1774(s),

1612, 1491, 1362, 1170, 1042, 662, 584; HRESIMS Calcd for $[C_{27}H_{25}NNaO_4S]^+$ (M + Na^+) 482.1397, found 482.1396.

(8*S*,10*S*)-10-(hydroxymethyl)-5-tosyl-5,6,7,8,9,10-hexahydrocyclohepta[*b*]indol-8-ol (2ag)

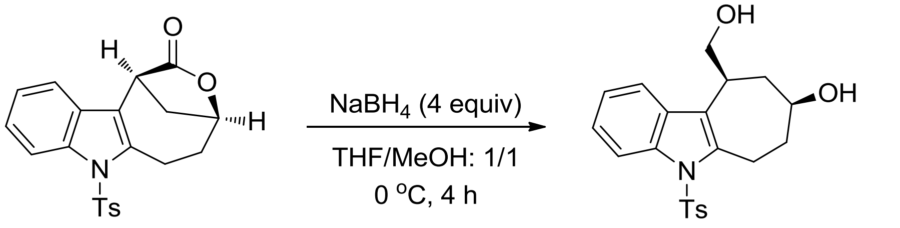

2a-*ent*, 95% ee **2ag**, 68%, 97% ee, dr > 20:1

Compound **2ag** was prepared in 68% yield (52.4 mg) according to the above procedure (0.2 mmol scale). $[\alpha]_D^{20}$ = -80.5 ° (c = 1.0, $CHCl_3$). 97% ee (determined by HPLC: Chiralcel AD-H Column, 5/95 *i*-PrOH/hexane, 2.0 mL/min, 200 nm; TR = 37.54 min (minor), 40.93 min (major)). Pale yellow oil. ^{1}H NMR (400 MHz, $CDCl_3$) δ 8.21 (d, *J* = 7.6 Hz, 1H), 7.56 (d, *J* = 8.4 Hz, 2H), 7.41 – 7.32 (m, 1H), 7.31 – 7.20 (m, 2H), 7.17 (d, *J* = 8.4 Hz, 2H), 4.34 – 4.20 (m, 1H), 3.82 (d, *J* = 6.4 Hz, 2H), 3.47 (s, 1H), 3.41 – 3.27 (m, 2H), 3.24 – 3.09 (m, 1H), 2.38 – 2.22 (m, 4H), 2.06 – 1.92 (m, 2H), 1.91 – 1.75 (m, 2H); ^{13}C NMR (100 MHz, $CDCl_3$) δ 144.6, 139.3, 136.5, 136.1, 130.3, 129.7, 126.2, 124.2, 123.6, 122.2, 117.7, 115.4, 69.9, 65.1, 36.0, 35.1, 34.0, 21.5, 21.0; IR (neat): 3300, 2933, 1530, 1218, 1175, 580; HRESIMS Calcd for $[C_{21}H_{23}NNaO_4S]^+$ (M + Na^+) 408.1240, found 408.1242.

(*S*)-(8-oxo-5-tosyl-5,6,7,8,9,10-hexahydrocyclohepta[*b*]indol-10-yl)methyl 4-nitrobenzoate (2ah)

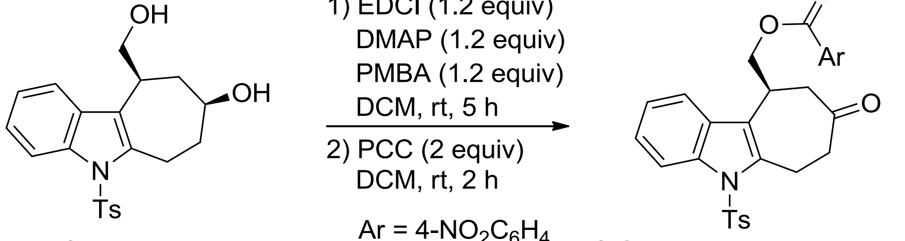

2ag, 97% ee **2ah**, 46%, 97% ee (2 steps)

Compound **2ah** was prepared in 46% yield (24.5 mg, 2 steps) according to the above procedure (0.1 mmol scale). $[\alpha]_D^{20}$ = -65.5 ° (c = 1.0, $CHCl_3$). 97% ee (determined by HPLC: Chiralcel OD-H Column, 20/80 *i*-PrOH/hexane, 2.0 mL/min, 200 nm; TR = 25.95 min (minor), 31.98 min (major)). Pale yellow solid (mp 201-202 °C). ^{1}H NMR (400 MHz, $CDCl_3$) δ 8.32 – 8.17 (m, 3H), 8.11 – 7.98 (m, 2H), 7.62 – 7.53 (m, 3H), 7.42 – 7.26 (m, 2H), 7.18 (d, *J* = 8.1 Hz, 2H), 4.67 (dd, *J* = 11.2, 5.6 Hz, 1H), 4.25 (dd, *J* = 11.2, 8.8 Hz, 1H), 3.87 – 3.78 (m, 1H), 3.69 – 3.57 (m, 1H), 3.52 – 3.40 (m, 1H), 3.12 – 2.94 (m, 2H), 2.81 – 2.69 (m, 1H), 2.68 – 2.56 (m, 1H), 2.33 (s, 3H); ^{13}C NMR (100 MHz, $CDCl_3$) δ 209.5, 164.4, 150.6, 145.1, 137.2, 136.5, 136.0, 135.0, 130.7, 129.9, 129.5, 126.2, 125.1, 123.9, 123.5, 119.1, 118.2, 115.5, 67.1, 43.2(4), 43.1(5), 32.3, 21.6, 21.5; IR (neat): 2933, 1750(s), 1738(s), 1520(s), 1325, 1215, 1120, 586; HRESIMS Calcd for $[C_{28}H_{24}N_2NaO_7S]^+$ (M + Na^+) 555.1196, found 555.1199.

methyl 7-methoxy-5-tosyl-5,6,7,8,9,10-hexahydrocyclohepta[*b*]indole-10-carboxylate (2ai)

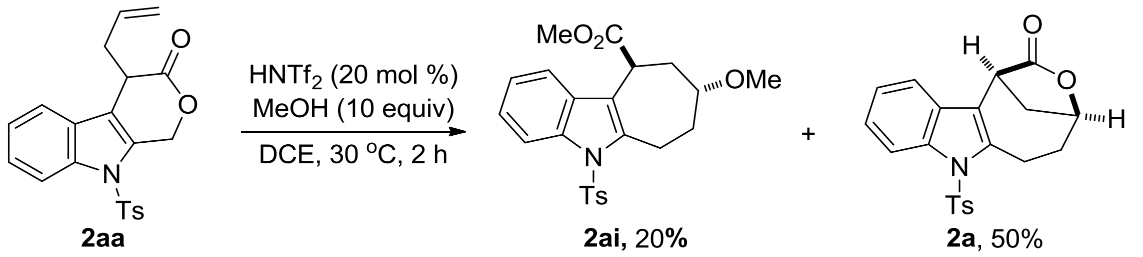

Compound **2ai** was prepared in 20% yield (17.1 mg; d.r. > 20:1) according to the general procedure. Pale yellow oil. ^{1}H NMR (400 MHz, $CDCl_3$) δ 8.22 (dd, *J* = 7.2, 0.8 Hz, 1H), 7.63 – 7.54 (m, 2H), 7.36 – 7.30 (m, 1H), 7.29 – 7.22 (m, 2H), 7.17 (d, *J* = 8.0 Hz, 2H), 4.10 (dd, *J* = 5.2, 3.2 Hz, 1H), 3.67 – 3.61 (m, 4H), 3.59 – 3.50 (m, 1H), 3.38 (s, 3H), 3.02 – 2.91 (m, 1H), 2.74 – 2.62 (m, 1H), 2.34 (s, 3H), 2.17 – 2.06 (m, 1H), 1.72 – 1.58 (m, 2H); ^{13}C NMR (125 MHz, $CDCl_3$) δ 172.8, 144.7, 139.7, 136.2, 130.2, 129.8, 126.3, 124.3, 123.6, 119.4, 117.7, 115.3, 78.8, 56.2, 52.1, 37.7, 34.4, 31.2, 21.6, 21.3; IR (neat): 2994, 1769(s), 1758, 1375, 1245, 1056, 913, 743; HRESIMS Calcd for $[C_{23}H_{25}NNaO_5S]^+$ (M + Na^+) 450.1346, found 548.0153.

7-tosyl-4,5,6,7-tetrahydro-1,4-methanooxocino[5,4-*b*]indol-2(1*H*)-one-5,5-d_2 (2a′)

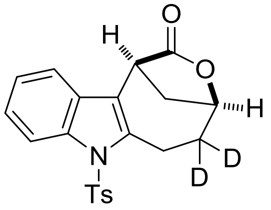

2a′

Compound **2a′** was prepared in 74% yield (56.7 mg) according to the general procedure. ^{1}H NMR (400 MHz, $CDCl_3$) δ 8.24 (d, *J* = 8.0 Hz, 1H), 7.55 (d, *J* = 8.4 Hz, 2H), 7.49 – 7.42 (m, 1H), 7.36 – 7.24 (m, 2H), 7.18 (d, *J* = 8.0 Hz, 2H), 5.09 (d, *J* = 8.0 Hz, 1H), 3.94 (d, *J* = 17.2 Hz, 1H), 3.85 (d, *J* = 8.0 Hz, 1H), 2.85 (d, *J* = 17.2 Hz, 1H), 2.78 – 2.65 (m, 1H), 2.33 (s, 3H), 1.88 (d, *J* = 12.4 Hz, 1H); IR (neat): 2922, 1785(s), 1449, 1363, 1274, 1261, 764, 749; HRESIMS Calcd for $[C_{21}H_{17}D_2NNaO_4S]^+$ (M + Na^+) 406.1053, found 406.1055.

7-tosyl-4,5,6,7-tetrahydro-1,4-methanooxocino[5,4-*b*]indol-2(1*H*)-one-4-*d* (2a″)

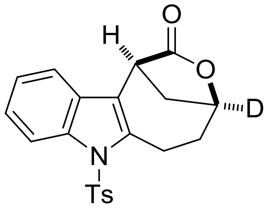

2a″

Compound **2a″** was prepared in 72% yield (55.0 mg) according to the general procedure. ^{1}H NMR (400 MHz, $CDCl_3$) δ 8.24 (d, *J* = 7.6 Hz, 1H), 7.55 (d, *J* = 8.4 Hz, 2H), 7.49 – 7.42 (m, 1H), 7.36 – 7.25 (m, 2H), 7.18 (d, *J* = 8.0 Hz, 2H), 5.11 (t, *J* = 7.2 Hz, 0.1H), 3.96 (dt, *J* = 17.2, 4.4 Hz, 1H), 3.85 (d, *J* = 8.0 Hz, 1H), 2.95 – 2.79 (m, 1H), 2.77 – 2.65 (m, 1H), 2.47 – 2.36 (m, 1H), 2.33 (s, 3H), 1.88 (d, *J* = 12.0 Hz, 1H), 1.85 – 1.76 (m, 1H); IR (neat): 2922, 2851, 1773(s), 1449, 1363, 1275, 1171, 763, 749; HRESIMS Calcd for $[C_{21}H_{18}DNNaO_4S]^+$ (M + Na^+) 405.0990, found 405.0996.

1-allyl-6-tosyl-1,4,5,6-tetrahydro-2*H*-oxepino[4,5-*b*]indol-2-one (4ga)

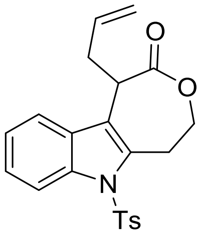

4ga

Compound **4ga** was prepared in 42% yield (33.2 mg) according to the general procedure. Pale yellow oil. ^{1}H NMR (400 MHz, $CDCl_3$) δ 8.21 (dd, *J* = 8.0, 0.8 Hz, 1H), 7.60 (d, *J* = 8.4, 2H), 7.40 – 7.31 (m, 2H), 7.29 (dd, *J* = 7.6, 1.2 Hz, 1H), 7.20 (d, *J* = 8.0, 2H), 6.00 – 5.80 (m, 1H), 5.24 – 5.07 (m, 2H), 4.85 (td, *J* = 12.8, 1.6 Hz, 1H), 4.53 (dt, *J* = 12.8, 4.0 Hz, 1H), 4.30 (dd, *J* = 9.6, 6.4 Hz, 1H), 3.66 (dd, *J* = 18.8, 2.0 Hz, 1H), 3.43 – 3.25 (m, 1H), 2.87 – 2.62 (m, 2H), 2.35 (s, 3H); ^{13}C NMR (100 MHz, $CDCl_3$) δ 171.9, 145.3, 136.4, 135.6, 133.8, 133.6, 130.1, 128.9, 126.3, 125.2, 123.8, 118.4, 118.1, 115.6, 114.8, 64.5, 45.2, 38.9, 29.3, 21.5; IR (neat): 2924, 1732(s), 1454, 1358, 1173, 1151, 1089, 575; HRESIMS Calcd for $[C_{22}H_{21}NNaO_4S]^+$ (M + Na^+) 418.1083, found 418.1083.

4-allylisochroman-3-one (4ha)

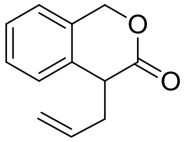

4ha

Compound **4ha** was prepared in 85% yield (32.0 mg) according to the general procedure. Pale yellow oil. ^{1}H NMR (400 MHz, $CDCl_3$) δ 7.38 – 7.20 (m, 4H), 5.93 – 5.81 (m, 1H), 5.41 (d, 1H, *J* = 14.0 Hz), 5.28 (d, 1H, *J* = 14.0 Hz), 5.17 – 5.09 (m, 2H), 3.70 (t, 1H, *J* = 6.8 Hz), 2.87 – 2.66 (m, 2H); ^{13}C NMR (100 MHz, $CDCl_3$) δ 172.2, 134.2, 134.0, 131.4, 128.6, 127.2, 126.6, 124.6, 118.1, 69.5, 45.3, 34.2; IR (neat): 3076, 2924, 1744 (s), 1641, 1489, 1462, 1385, 1234, 1146, 1047, 913, 747; HRESIMS Calcd for $[C_{12}H_{12}NaO_2]^+$ (M + Na^+) 211.0730, found 211.0732.

Reference:

1. a) J. Zheng, Z. Li, W. Wu, H. Jiang, *Org. Lett.* **2016**, *18*, 6232; b) A. Arcadi, G. Bianchi, F. Marinelli, *Synthesis* **2004**, *4*, 610.
2. a) C. Shu, L. Li, X.-Y. Xiao, Y.-F. Yu, Y.-F. Ping, J.-M. Zhou, L.-W. Ye, *Chem. Commun.* **2014**, *50*, 8689; b) F. Pan, S. Liu**,** C. Shu, R.-K. Lin**,** Y.-F. Yu, J.-M. Zhou, L.-W. Ye, *Chem. Commun.* **2014**, *50*, 10726; c) N. S. Kumar, E. M. Dullaghan, B. B. Finlay, H. Gong, N. E. Reiner, J. J. P. Selvam, L. M. Thorson, S. Campbell, N. Vitko, A. R. Richardson, R. Zoraghi, R. N. Young, *Bioorg. Med. Chem.* **2014**, *22*, 1708; d) M. Yu, Y. Xie, C. Xie, Y. Zhang, *Org. Lett.* **2012**, *14*, 2164; e) M. Garzon, P. W. Davies, *Org. Lett.* **2014**, *16*, 4850; f) Y. Pan, G.-W. Chen**,** C.-H. Shen, W. He, L.-W. Ye, *Org. Chem. Front.* **2016**, *3*, 491.
3. a) X. Wu, Z. Chen, Y.-B. Bai, V. M. Dong, *J. Am. Chem. Soc.* **2016**, *138*, 12013; b) L.-L. Mao, C.-C. Li, Q. Yang, M.-X. Cheng, S.-D. Yang, *Chem. Commun*, **2017**, *53*, 4473; c) S. Hirano, R. Tanaka, H. Urabe, F. Sato, *Org. Lett*. **2004**, *6*, 727; d) C. Gryparis, C. Efe, C. Raptis, I. N. Lykakis, M. Stratakis, *Org. Lett*. **2012**, *14*, 2956.
4. J. Liu, Z. Fang, Q. Zhang, Q. Liu, X. Bi, *Angew. Chem. Int. Ed.* **2013**, *52*, 6953.
5. C. Zheng, J. J. Chen, R. Fan, *Org. Lett.* **2014**, *16*, 816.
6. K. Miyashita, T. Tsunemi, T. Hosokawa, M. Ikejiri, T. Imanishi, *J. Org. Chem.* **2008**, *73*, 5360.
7. N. Sakai, T. Moriya, K. Fujii, T. Konakahara, *Synthesis* **2008**, *21*, 3533.
8. L. Li, X.-M. Chen, Z.-S. Wang, B. Zhou, X. Liu, X. Lu, L.-W. Ye, *ACS Catal.* **2017**, *7*, 4004.

7-tosyl-4,5,6,7-tetrahydro-1,4-methanooxocino[5,4-*b*]indol-2(1*H*)-one (2a). CCDC Number = 1831051

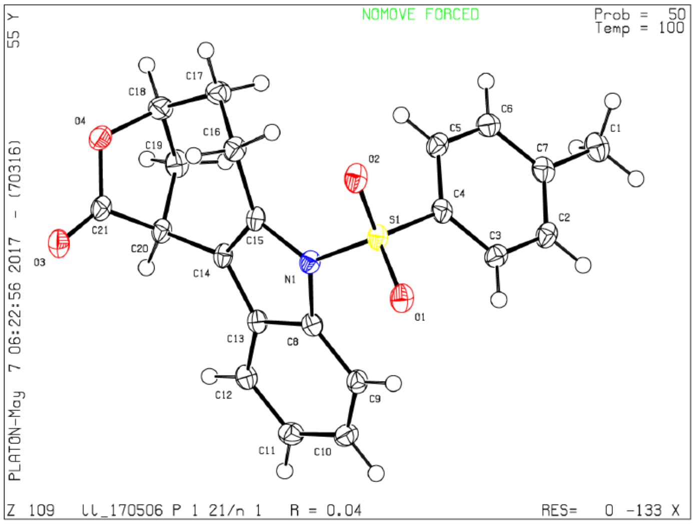

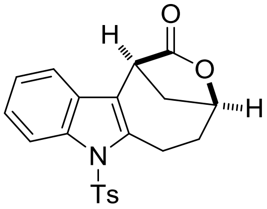

Bond precision: C-C = 0.0020 A Wavelength=1.54184

Cell:	a=9.07481(16)	b=15.0220(2)	c=12.7620(2)
	alpha=90	beta=99.1959(16)	gamma=90
Temperature:	100 K		

	Calculated	Reported
Volume	1717.38(5)	1717.38(5)
Space group	P 21/n	P 1 21/n 1
Hall group	-P 2yn	-P 2yn
Moiety formula	C21 H19 N O4 S	C21 H19 N O4 S
Sum formula	C21 H19 N O4 S	C21 H19 N O4 S
Mr	381.43	381.43
Dx,g cm-3	1.475	1.475
Z	4	4
Mu (mm-1)	1.924	1.924
F000	800.0	800.0
F000'	803.67	
h,k,lmax	10,17,15	10,17,15
Nref	3062	3057
Tmin,Tmax	0.794,0.825	0.567,1.000
Tmin'	0.749	

Correction method= # Reported T Limits: Tmin=0.567 Tmax=1.000
AbsCorr = MULTI-SCAN

Data completeness= 0.998 Theta(max)= 67.074

R(reflections)= 0.0396(2966) wR2(reflections)= 0.1068(3057)

S = 1.024 Npar= 245

5-methyl-7-tosyl-4,5,6,7-tetrahydro-1,4-methanooxocino[5,4-*b*]indol-2(1*H*)-one (2s).

CCDC Number = 1831053

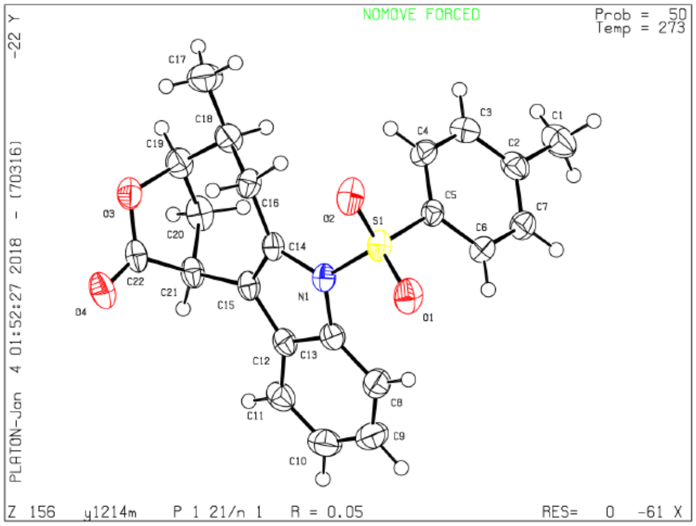

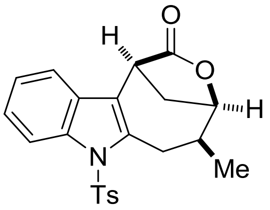

Bond precision:	C-C = 0.0030 A		Wavelength=0.71073
Cell:	a=13.780(3)	b=9.3801(19)	c=14.410(3)
	alpha=90	beta=92.893(4)	gamma=90
Temperature:	273 K		

	Calculated	Reported
Volume	1860.2(7)	1860.2(6)
Space group	P 21/n	P 1 21/n 1
Hall group	-P 2yn	-P 2yn
Moiety formula	C22 H21 N O4 S	C22 H21 N O4 S
Sum formula	C22 H21 N O4 S	C22 H21 N O4 S
Mr	395.46	395.46
Dx,g cm-3	1.412	1.412
Z	4	4
Mu (mm-1)	0.204	0.204
F000	832.0	832.0
F000'	832.88	
h,k,lmax	17,12,18	17,12,18
Nref	4265	4240
Tmin,Tmax	0.980,0.980	
Tmin'	0.980	

Correction method= Not given

Data completeness= 0.994 Theta(max)= 27.485

R(reflections)= 0.0530(3644) wR2(reflections)= 0.1365(4240)

S = 1.070 Npar= 255

9-methyl-4-phenyl-7-tosyl-4,5,6,7-tetrahydro-1,4-methanooxocino[5,4-*b*]indol-2(1*H*)-one (2u). CCDC Number = 1831054

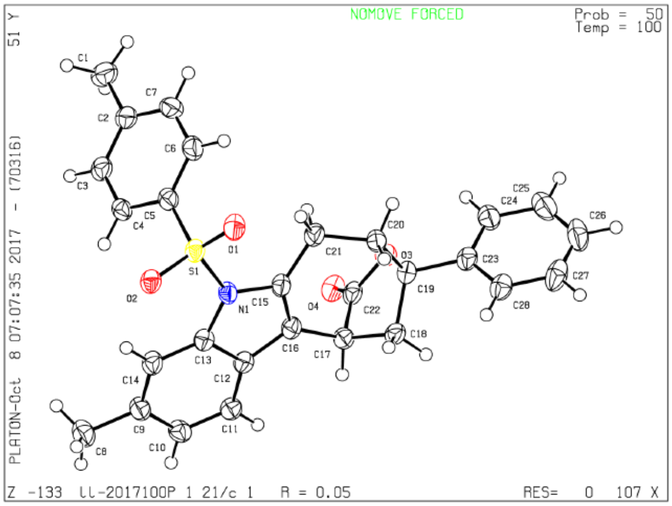

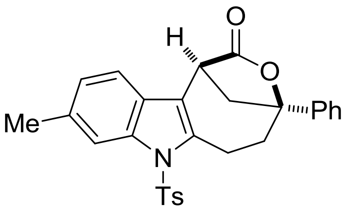

Bond precision:	C-C = 0.0041 A		Wavelength=1.54184
Cell:	a=12.3488(2)	b=9.82302(14)	c=19.2566(4)
	alpha=90	beta=108.549(2)	gamma=90
Temperature:	100 K		

	Calculated	Reported
Volume	2214.53(7)	2214.53(8)
Space group	P 21/c	P 1 21/c 1
Hall group	-P 2ybc	-P 2ybc
Moiety formula	C28 H25 N O4 S	C28 H25 N O4 S
Sum formula	C28 H25 N O4 S	C28 H25 N O4 S
Mr	471.55	471.55
Dx,g cm-3	1.414	1.414
Z	4	4
Mu (mm-1)	1.606	1.606
F000	992.0	992.0
F000'	996.15	
h,k,lmax	14,11,22	14,11,21
Nref	3907	3742
Tmin,Tmax	0.825,0.852	0.744,1.000
Tmin'	0.725	

Correction method= # Reported T Limits: Tmin=0.744 Tmax=1.000
AbsCorr = MULTI-SCAN

Data completeness= 0.958 Theta(max)= 66.583

R(reflections)= 0.0479(3502) wR2(reflections)= 0.1277(3742)

S = 1.156 Npar= 309

(*S*)-(8-oxo-5-tosyl-5,6,7,8,9,10-hexahydrocyclohepta[*b*]indol-10-yl)methyl 4-nitrobenzoate (2ah). CCDC Number = 1864037

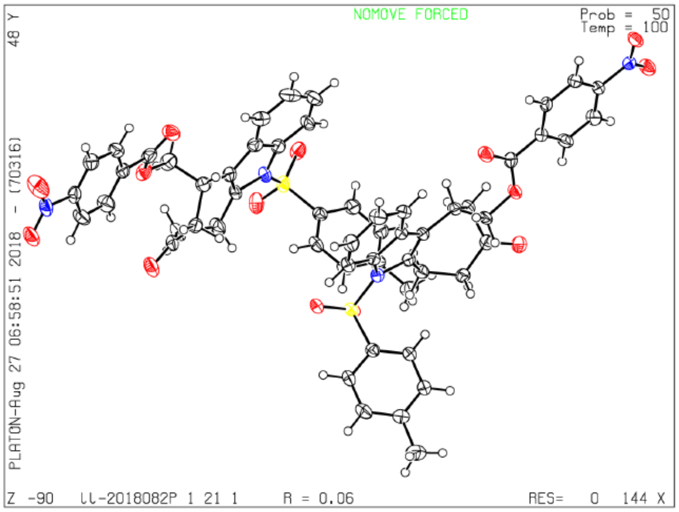

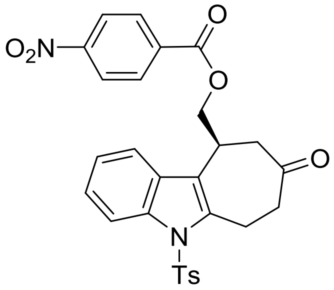

Bond precision: C-C = 0.0080 A Wavelength=1.54184

Cell:	a=9.68936(19)	b=16.6569(4)	c=15.2343(3)
	alpha=90	beta=94.933(2)	gamma=90
Temperature:	100 K		

	Calculated	Reported
Volume	2449.63(9)	2449.63(9)
Space group	P 21	P 1 21 1
Hall group	P 2yb	P 2yb
Moiety formula	C28 H24 N2 O7 S	C28 H24 N2 O7 S
Sum formula	C28 H24 N2 O7 S	C28 H24 N2 O7 S
Mr	532.55	532.55
Dx,g cm-3	1.444	1.444
Z	4	4
Mu (mm-1)	1.629	1.629
F000	1112.0	1112.0
F000’	1116.84	
h,k,lmax	11,19,18	11,19,18
Nref	8746[4539]	6188
Tmin,Tmax		0.532,1.000
Tmin’		

Correction method= # Reported T Limits: Tmin=0.532 Tmax=1.000
AbsCorr = MULTI-SCAN

Data completeness= 1.36/0.71 Theta(max)= 67.079

R(reflections)= 0.0570(5742) wR2(reflections)= 0.1593(6188)

S = 1.026 Npar= 687

Compound 2a-*ent*

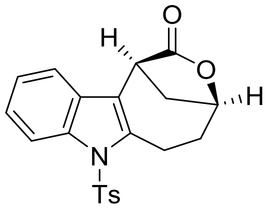

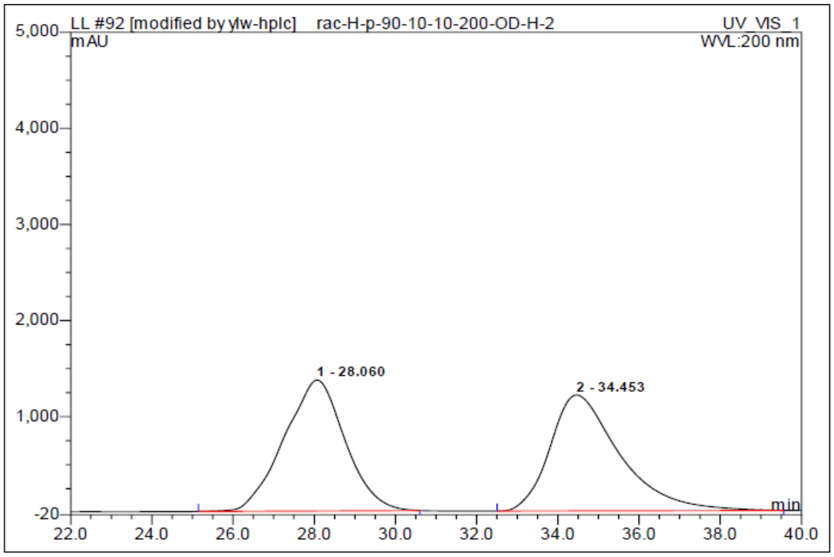

No.	Ret.Time min	Peak Name	Height mAU	Area mAU*min	Rel.Area %	Amount n.a.	Type
1	28.06	n.a.	1361.953	2376.246	49.84	n.a.	BMB*
2	34.45	n.a.	1204.479	2391.764	50.16	n.a.	BMB*
Total:			2566.432	4768.011	100.00	0.000	

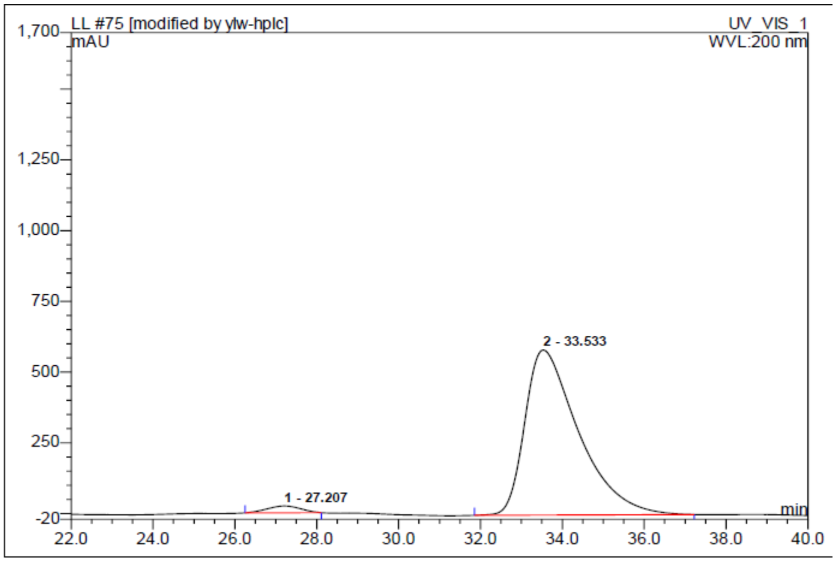

No.	Ret.Time min	Peak Name	Height mAU	Area mAU*min	Rel.Area %	Amount n.a.	Type
1	27.21	n.a.	23.780	22.429	2.46	n.a.	BMB*
2	33.53	n.a.	583.165	888.492	97.54	n.a.	BMB*
Total:			606.945	910.921	100.00	0.000	

Compound 2b-*ent*

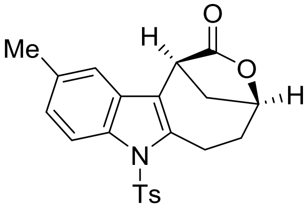

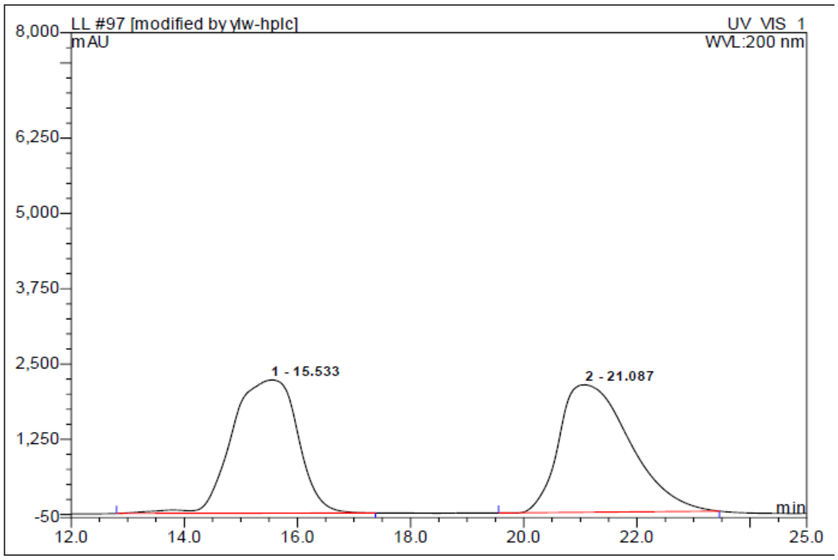

No.	Ret.Time min	Peak Name	Height mAU	Area mAU*min	Rel.Area %	Amount n.a.	Type
1	15.53	n.a.	2214.198	2969.358	49.14	n.a.	BMB*
2	21.09	n.a.	2117.625	3073.717	50.86	n.a.	BMB*
Total:			4331.823	6043.075	100.00	0.000	

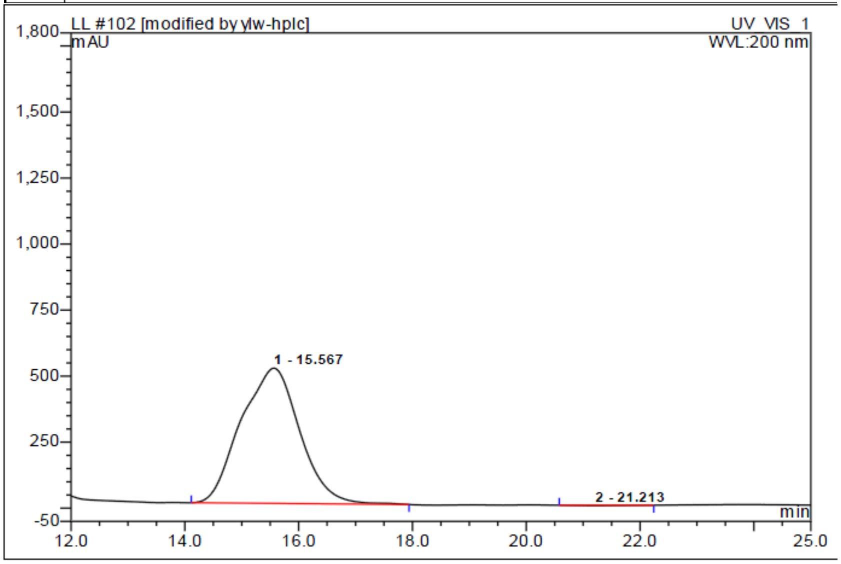

No.	Ret.Time min	Peak Name	Height mAU	Area mAU*min	Rel.Area %	Amount n.a.	Type
1	15.57	n.a.	511.996	613.674	99.75	n.a.	BMB*
2	21.21	n.a.	1.606	1.507	0.25	n.a.	BMB*
Total:			513.602	615.181	100.00	0.000	

Compound 2e-*ent*

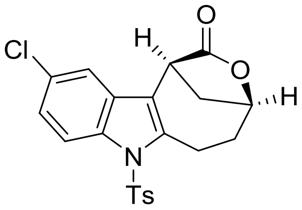

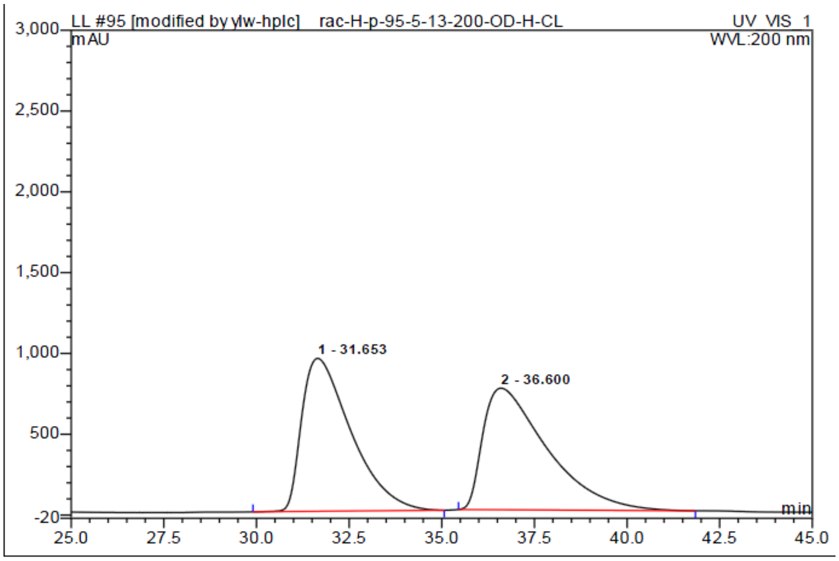

No.	Ret.Time min	Peak Name	Height mAU	Area mAU*min	Rel.Area %	Amount n.a.	Type
1	31.65	n.a.	945.708	1459.033	49.18	n.a.	BMB*
2	36.60	n.a.	751.863	1507.930	50.82	n.a.	BMB*
Total:			1697.571	2966.963	100.00	0.000	

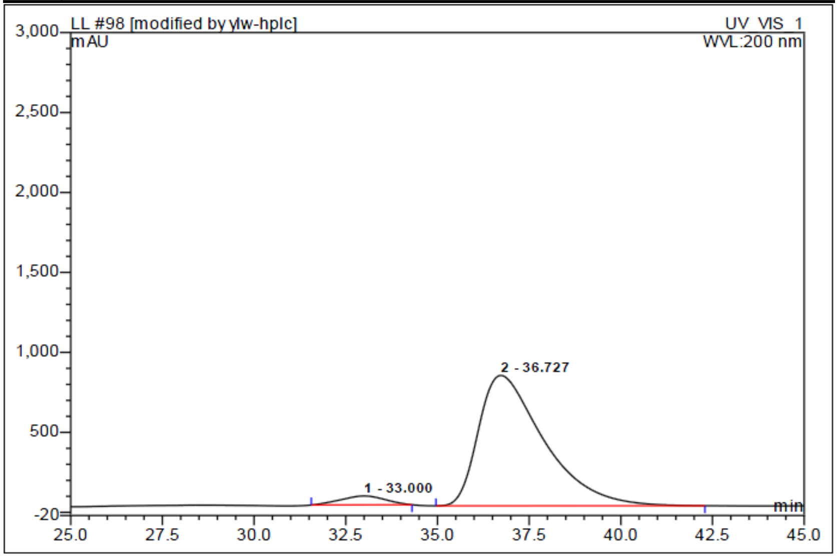

No.	Ret.Time min	Peak Name	Height mAU	Area mAU*min	Rel.Area %	Amount n.a.	Type
1	33.00	n.a.	55.668	78.538	4.45	n.a.	BMB*
2	36.73	n.a.	814.805	1686.126	95.55	n.a.	BMB*
Total:			870.473	1764.664	100.00	0.000	

Compound 2f-*ent*

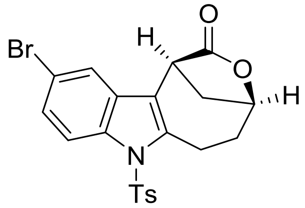

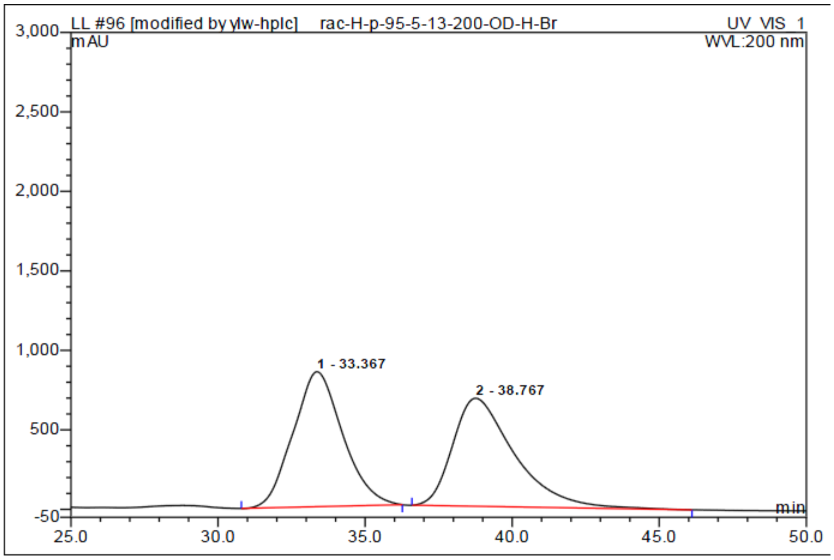

No.	Ret.Time min	Peak Name	Height mAU	Area mAU*min	Rel.Area %	Amount n.a.	Type
1	33.37	n.a.	848.367	1662.175	50.17	n.a.	BMB*
2	38.77	n.a.	678.954	1650.623	49.83	n.a.	BMB*
Total:			1527.320	3312.798	100.00	0.000	

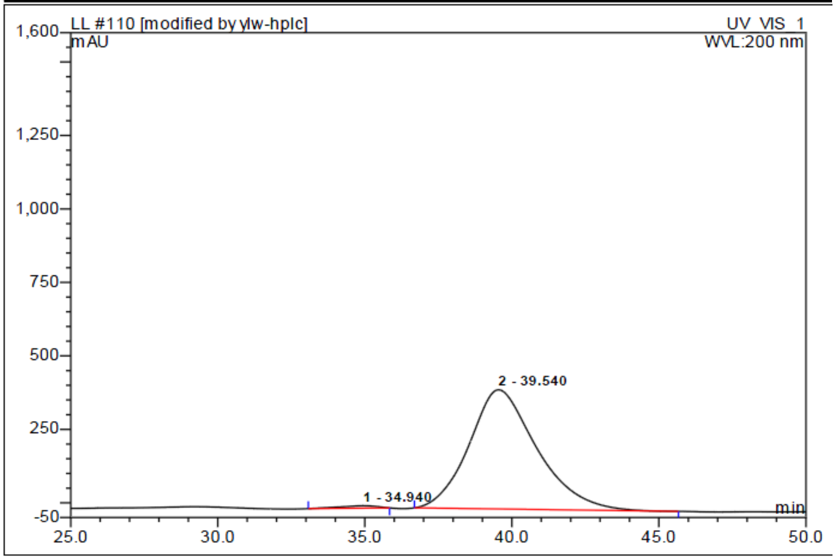

No.	Ret.Time min	Peak Name	Height mAU	Area mAU*min	Rel.Area %	Amount n.a.	Type
1	34.94	n.a.	8.311	12.311	1.12	n.a.	BMB*
2	39.54	n.a.	405.541	1086.257	98.88	n.a.	BMB*
Total:			413.852	1098.568	100.00	0.000	

Compound 2i-*ent*

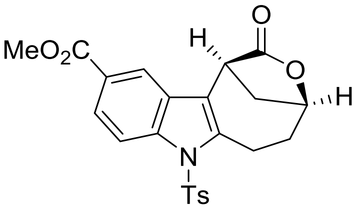

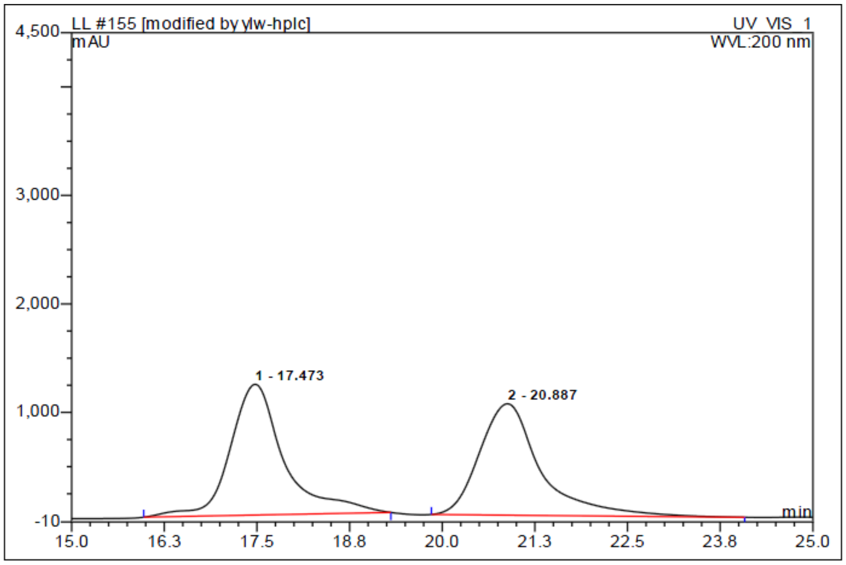

No.	Ret.Time min	Peak Name	Height mAU	Area mAU*min	Rel.Area %	Amount n.a.	Type
1	17.47	n.a.	1203.992	1018.388	50.96	n.a.	BMB*
2	20.89	n.a.	1025.920	979.895	49.04	n.a.	BMB*
Total:			2229.912	1998.283	100.00	0.000	

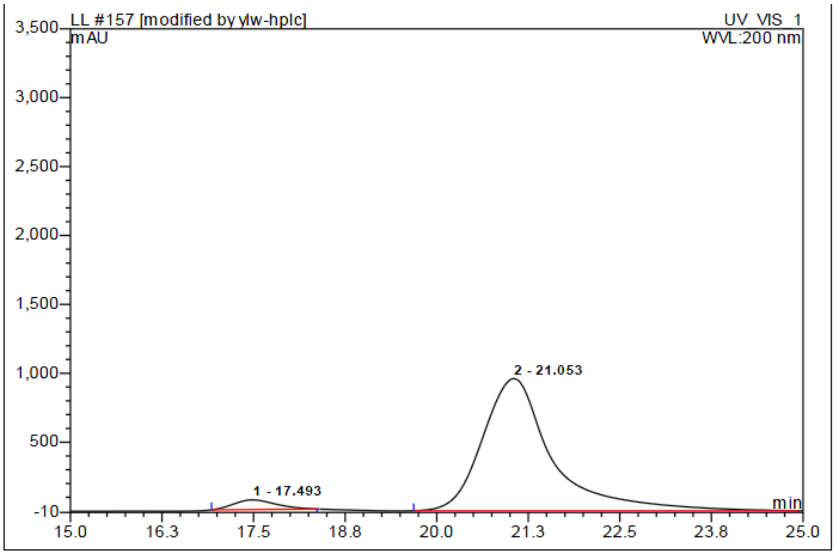

No.	Ret.Time min	Peak Name	Height mAU	Area mAU*min	Rel.Area %	Amount n.a.	Type
1	17.49	n.a.	69.773	46.415	4.22	n.a.	BMB*
2	21.05	n.a.	958.266	1053.332	95.78	n.a.	BMB*
Total:			1028.039	1099.746	100.00	0.000	

Compound 2j-*ent*

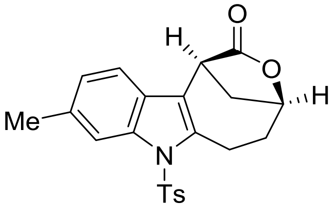

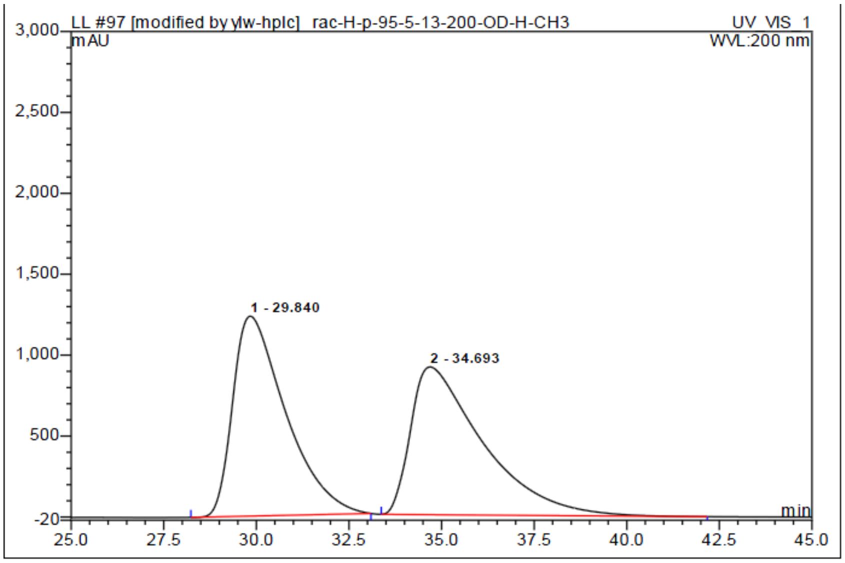

No.	Ret.Time min	Peak Name	Height mAU	Area mAU*min	Rel.Area %	Amount n.a.	Type
1	29.84	n.a.	1234.562	2044.940	49.83	n.a.	BMB*
2	34.69	n.a.	912.649	2058.774	50.17	n.a.	BMB*
Total:			2147.211	4103.713	100.00	0.000	

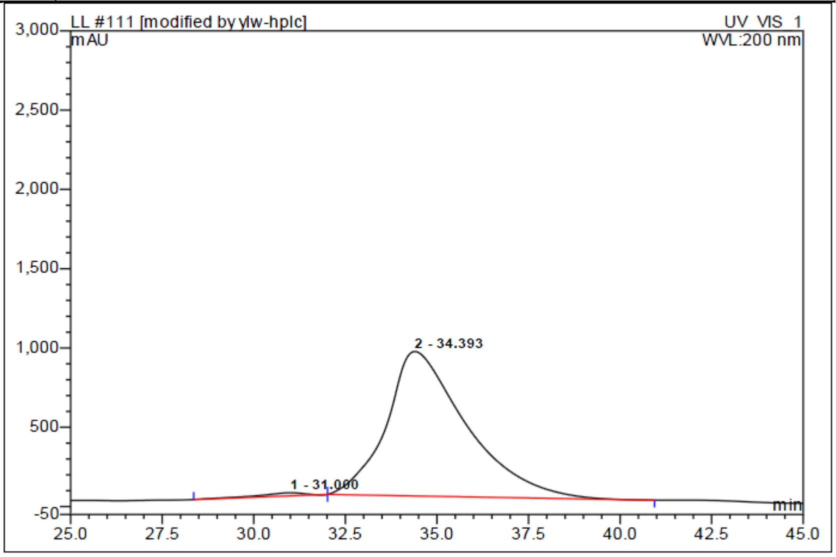

No.	Ret.Time min	Peak Name	Height mAU	Area mAU*min	Rel.Area %	Amount n.a.	Type
1	31.00	n.a.	19.113	27.169	1.16	n.a.	BMb*
2	34.39	n.a.	910.741	2305.936	98.84	n.a.	bMB*
Total:			929.855	2333.105	100.00	0.000	

Compound 2a-*ent'*

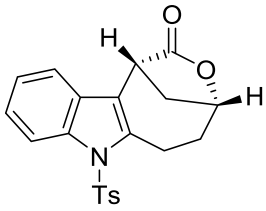

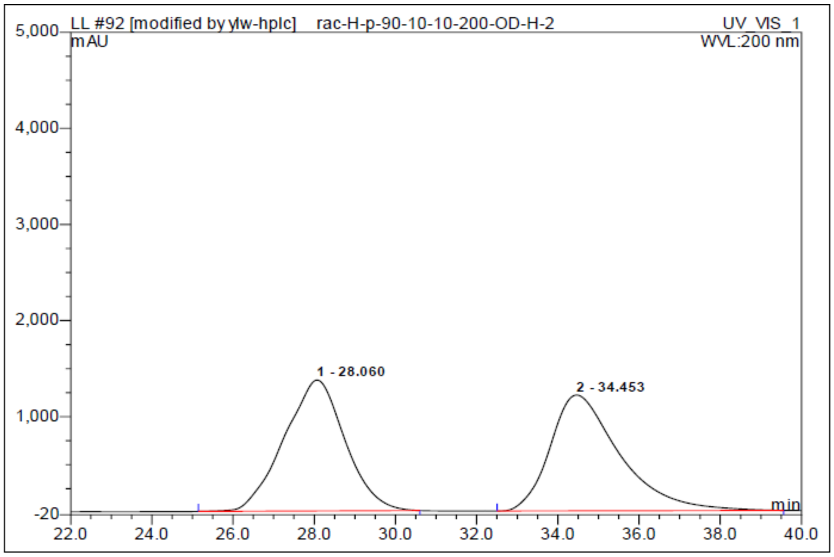

No.	Ret.Time min	Peak Name	Height mAU	Area mAU*min	Rel.Area %	Amount n.a.	Type
1	28.06	n.a.	1361.953	2376.246	49.84	n.a.	BMB*
2	34.45	n.a.	1204.479	2391.764	50.16	n.a.	BMB*
Total:			2566.432	4768.011	100.00	0.000	

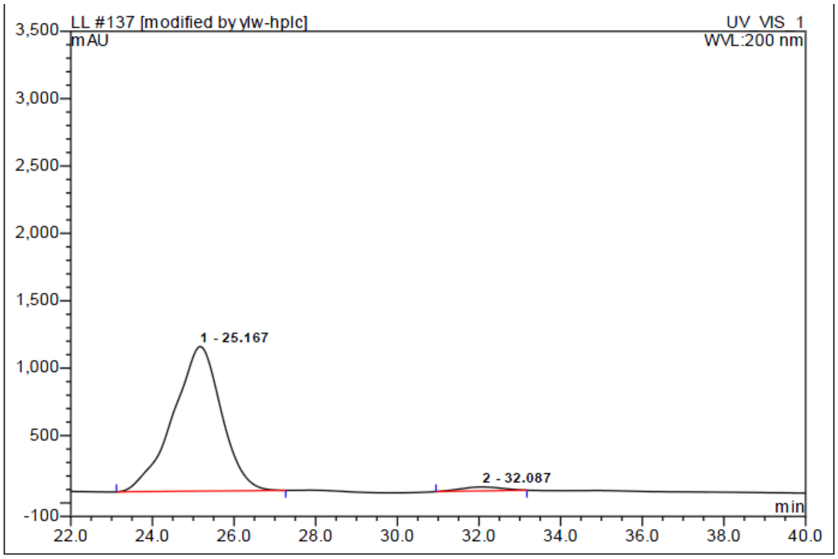

No.	Ret.Time min	Peak Name	Height mAU	Area mAU*min	Rel.Area %	Amount n.a.	Type
1	25.17	n.a.	1072.451	1471.426	97.73	n.a.	BMB*
2	32.09	n.a.	29.144	34.154	2.27	n.a.	BMB*
Total:			1101.595	1505.580	100.00	0.000	

Compound 2ag

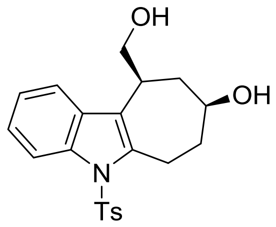

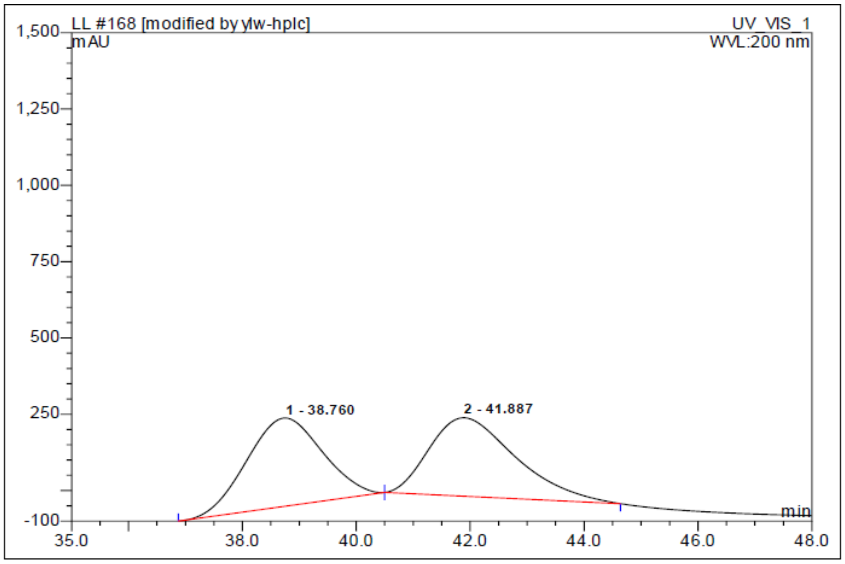

No.	Ret.Time min	Peak Name	Height mAU	Area mAU*min	Rel.Area %	Amount n.a.	Type
1	38.76	n.a.	288.746	444.091	49.57	n.a.	BMb*
2	41.89	n.a.	256.810	451.833	50.43	n.a.	bMB*
Total:			545.556	895.924	100.00	0.000	

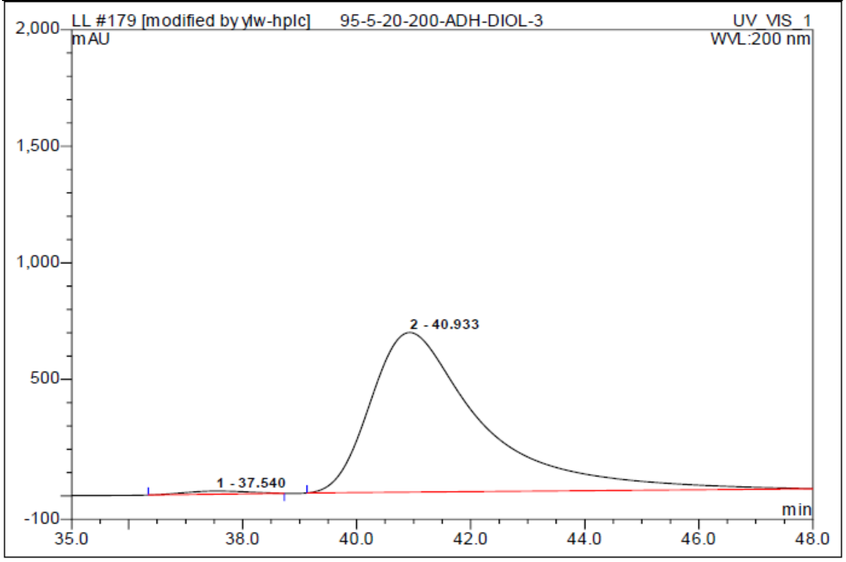

No.	Ret.Time min	Peak Name	Height mAU	Area mAU*min	Rel.Area %	Amount n.a.	Type
1	37.54	n.a.	13.595	17.382	1.11	n.a.	BMB*
2	40.93	n.a.	684.641	1545.118	98.89	n.a.	BMB*
Total:			698.237	1562.500	100.00	0.000	

Compound 2ah

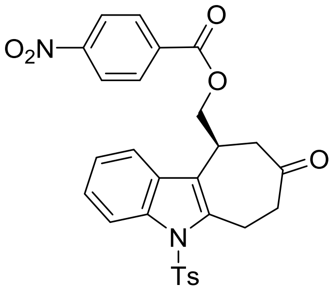

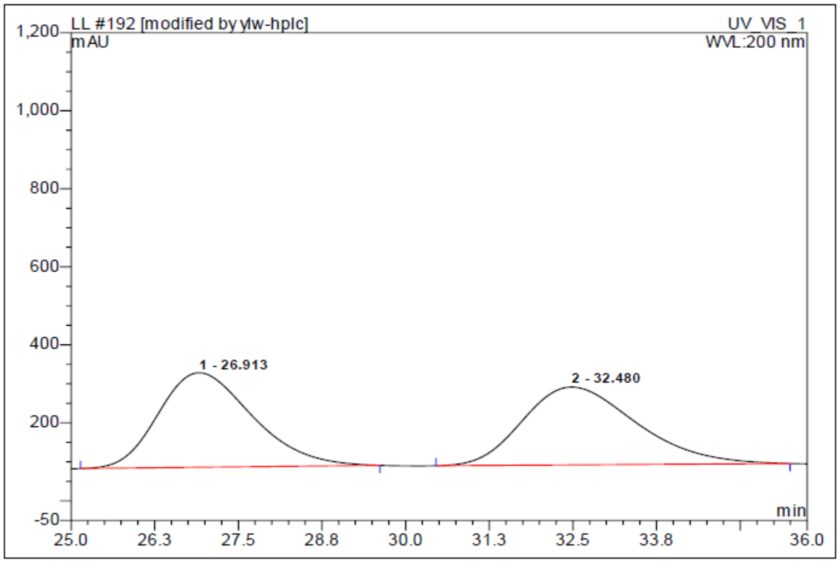

No.	Ret.Time min	Peak Name	Height mAU	Area mAU*min	Rel.Area %	Amount n.a.	Type
1	26.91	n.a.	242.256	394.451	50.08	n.a.	BMB*
2	32.48	n.a.	199.536	393.236	49.92	n.a.	BMB*
Total:			441.792	787.688	100.00	0.000	

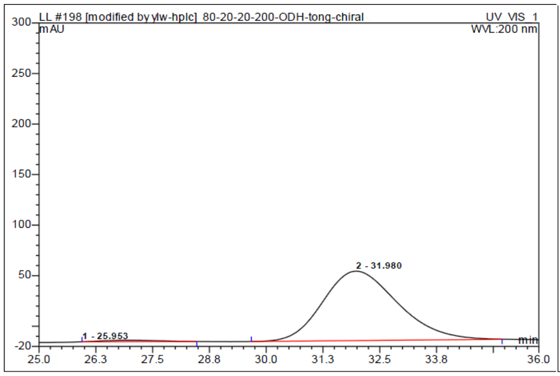

No.	Ret.Time min	Peak Name	Height mAU	Area mAU*min	Rel.Area %	Amount n.a.	Type
1	25.95	n.a.	0.040	2.070	1.56	n.a.	BMB*
2	31.98	n.a.	68.642	130.905	98.44	n.a.	BMB*
Total:			68.683	132.975	100.00	0.000	

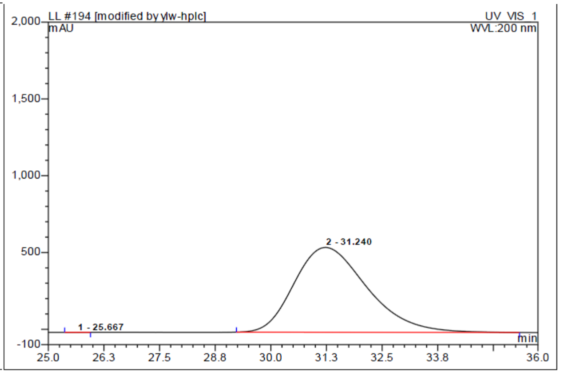

No.	Ret.Time min	Peak Name	Height mAU	Area mAU*min	Rel.Area %	Amount n.a.	Type
1	25.67	n.a.	0.087	0.017	0.00	n.a.	BMB*
2	31.24	n.a.	551.958	1050.901	100.00	n.a.	BMB*
Total:			552.044	1050.917	100.00	0.000	

after recrystallization

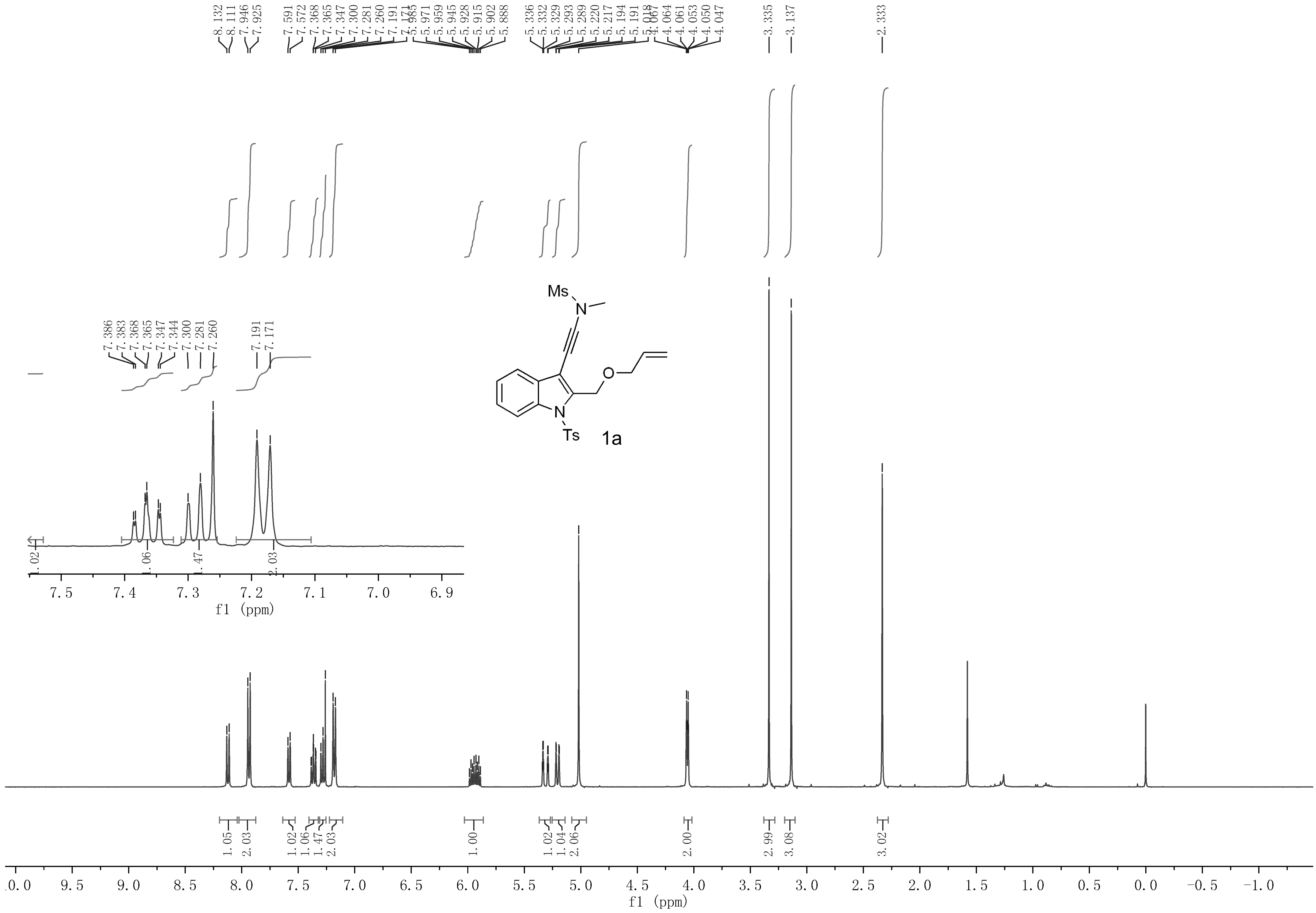

8.132 8.111 7.946 7.925 7.591 7.572 7.368 7.365 7.347 7.300 7.281 7.260 7.191 7.171 5.985 5.971 5.959 5.945 5.928 5.915 5.902 5.888
5.336 5.332 5.329 5.293 5.289 5.220 5.217 5.194 5.191 5.018 4.067 4.064 4.061 4.053 4.050 4.047
3.335 3.137 2.333
7.386 7.383 7.368 7.365 7.347 7.344 7.300 7.281 7.260 7.191 7.171
1.02 1.06 1.47 2.03
f1 (ppm)
Ms
N
O
N
Ts
1a
1.05 2.03 1.02 1.06 1.47 2.03 1.00 1.02 1.04 2.06 2.00 2.99 3.08 3.02
f1 (ppm)

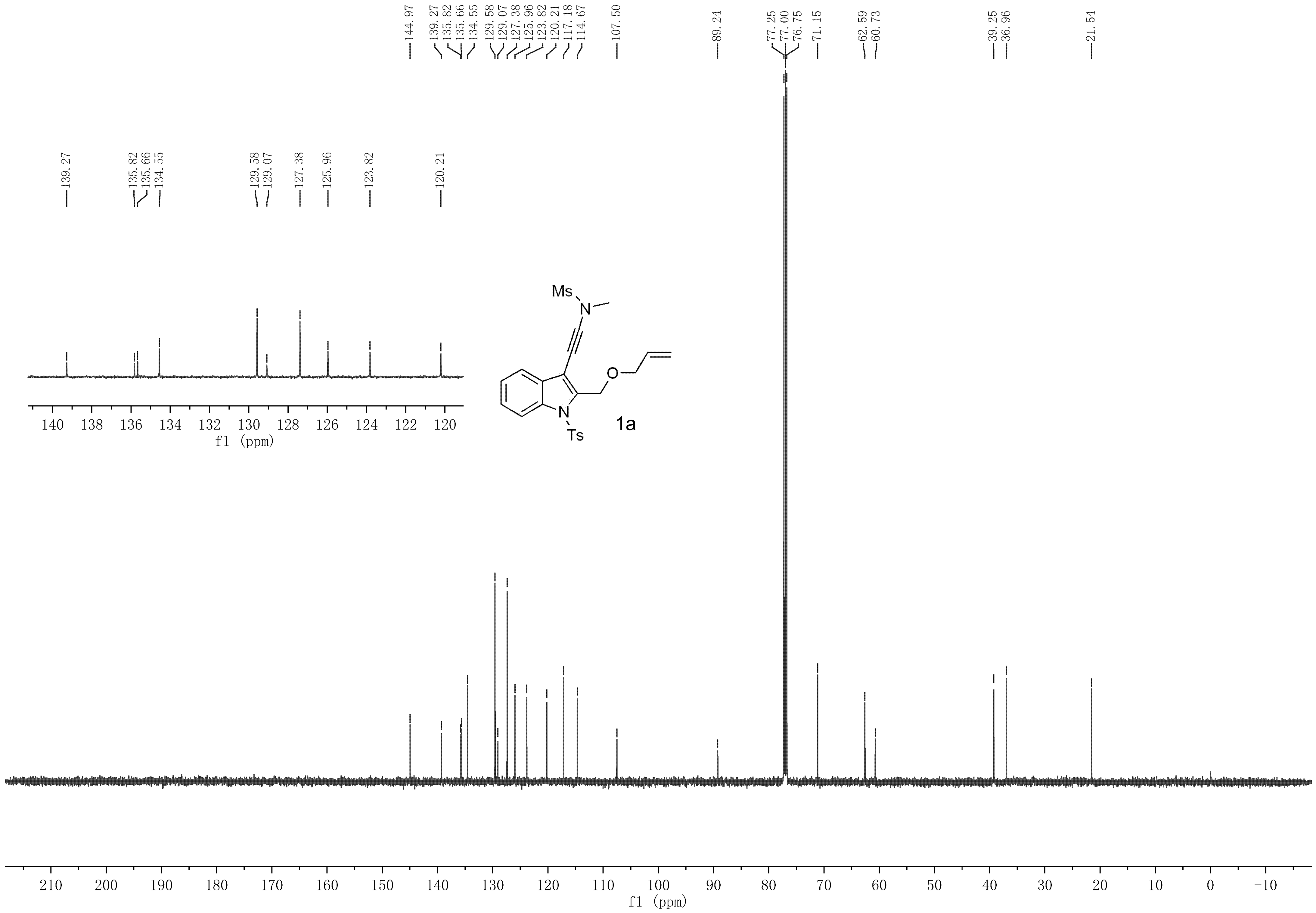
144.97
139.27
135.82
135.66
134.55
129.58
129.07
127.38
125.96
123.82
120.21
117.18
114.67
107.50
89.24
77.25
77.00
76.75
71.15
62.59
60.73
39.25
36.96
21.54
Ms
N
O
N
Ts
1a
140 138 136 134 132 130 128 126 124 122 120
f1 (ppm)
210 200 190 180 170 160 150 140 130 120 110 100 90 80 70 60 50 40 30 20 10 0 −10
f1 (ppm)

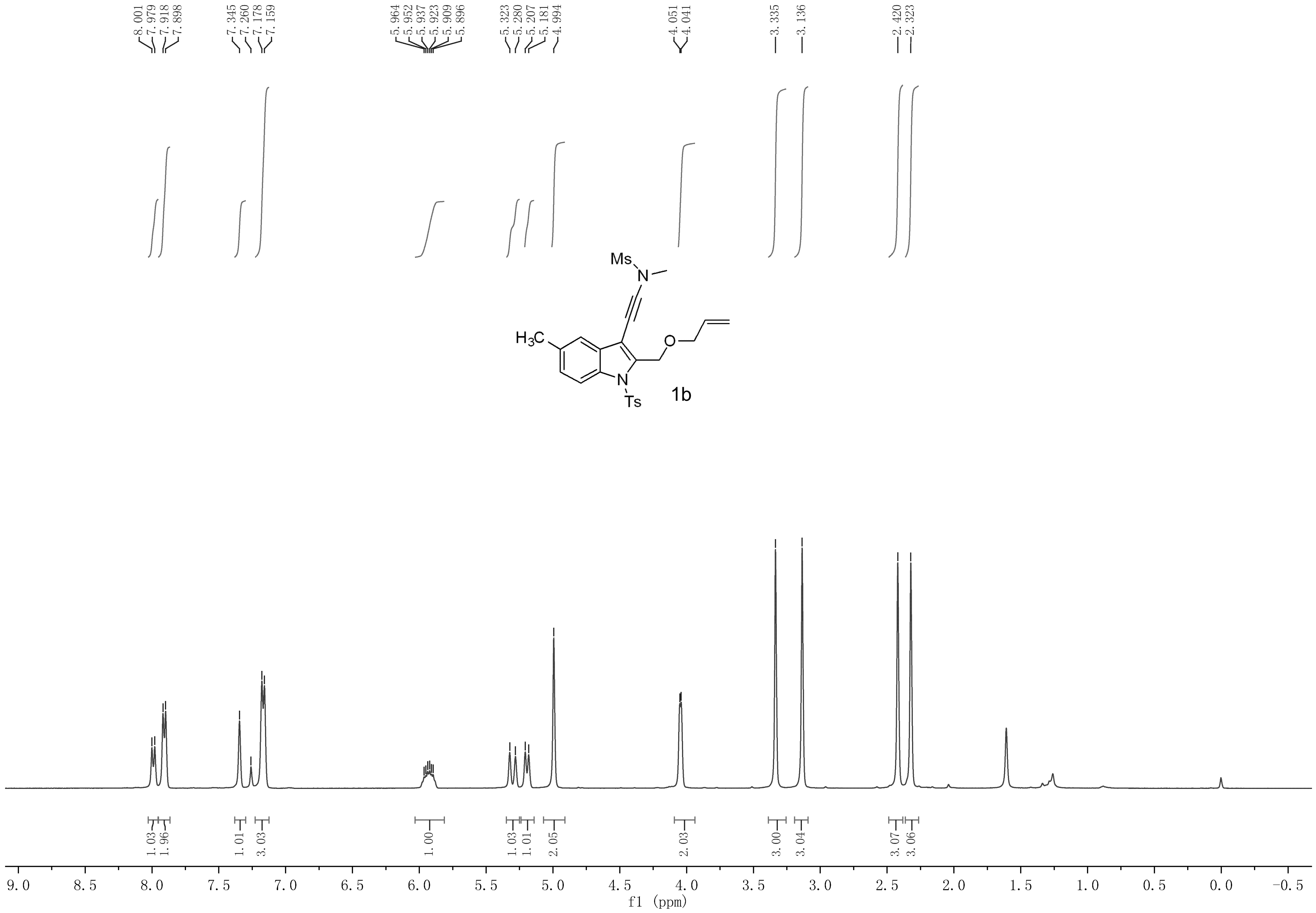
8.001
7.979
7.918
7.898
7.345
7.260
7.178
7.159
5.964
5.952
5.937
5.923
5.909
5.896
5.323
5.280
5.207
5.181
4.994
4.051
4.041
3.335
3.136
2.420
2.323
Ms
N
H3C
O
N
Ts
1b
1.03
1.96
1.01
3.03
1.00
1.03
1.01
2.05
2.03
3.00
3.04
3.07
3.06
9.0
8.5
8.0
7.5
7.0
6.5
6.0
5.5
5.0
4.5
4.0
3.5
3.0
2.5
2.0
1.5
1.0
0.5
0.0
-0.5
f1 (ppm)

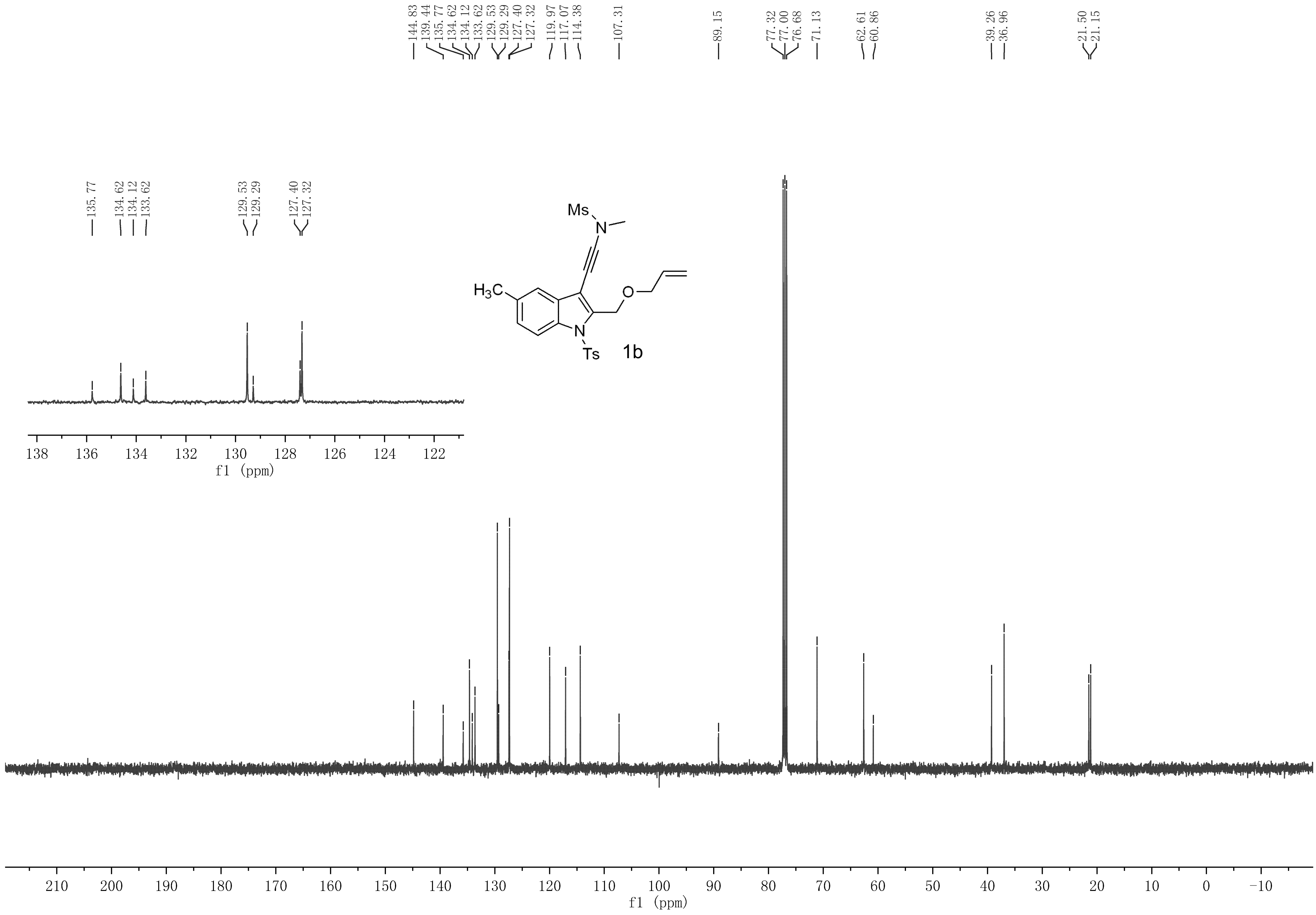
144.83
139.44
135.77
134.62
134.12
133.62
129.53
129.29
127.40
127.32
119.97
117.07
114.38
107.31
89.15
77.32
77.00
76.68
71.13
62.61
60.86
39.26
36.96
21.50
21.15
Ms
N
H3C
O
N
Ts
1b
138 136 134 132 130 128 126 124 122
f1 (ppm)
210 200 190 180 170 160 150 140 130 120 110 100 90 80 70 60 50 40 30 20 10 0 −10
f1 (ppm)

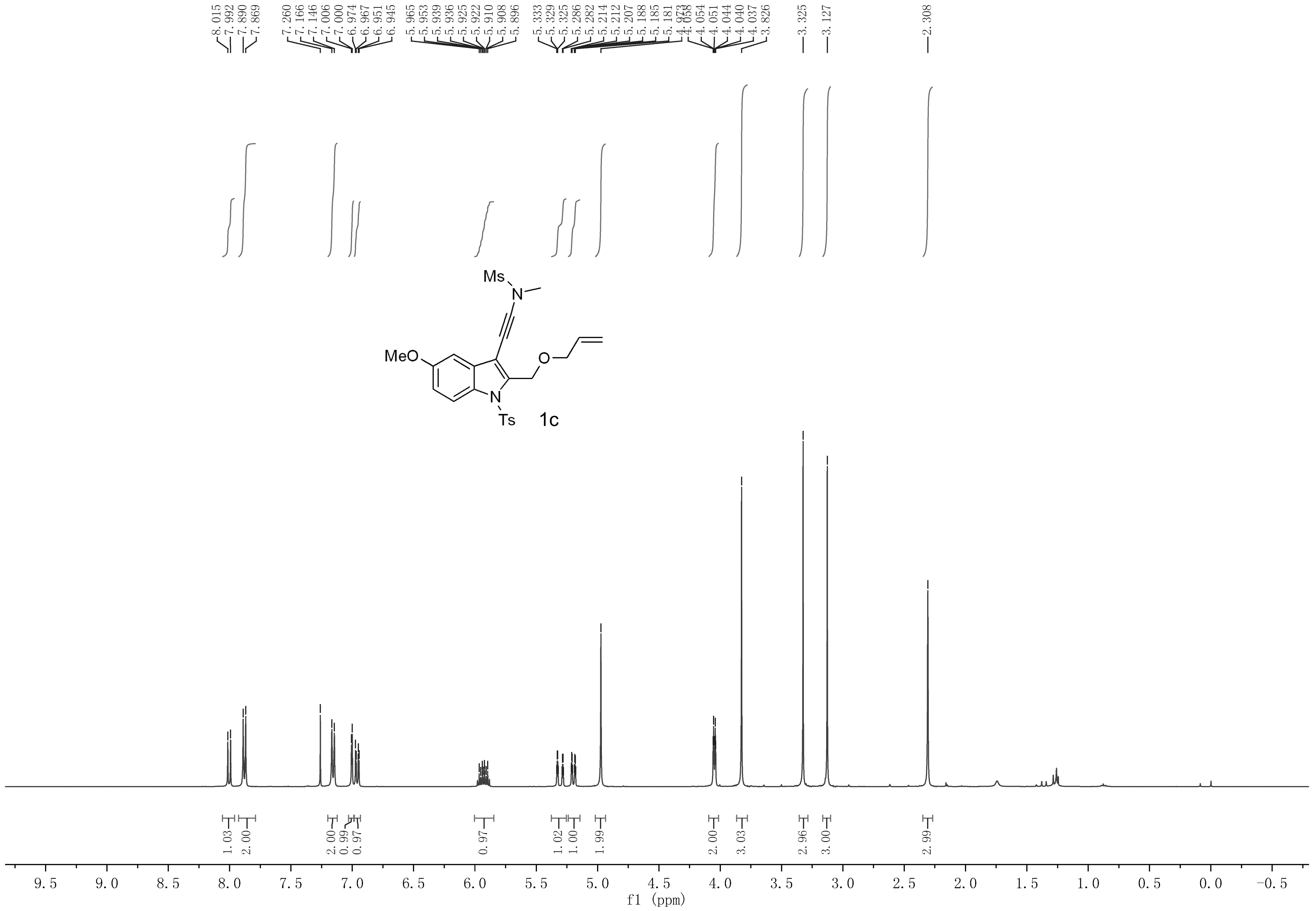
8.015
7.992
7.890
7.869
7.260
7.166
7.146
7.006
7.000
6.974
6.967
6.951
6.945
5.965
5.953
5.939
5.936
5.925
5.922
5.910
5.908
5.896
5.333
5.329
5.325
5.286
5.282
5.214
5.212
5.207
5.188
5.185
5.181
4.973
4.058
4.054
4.051
4.044
4.040
4.037
3.826
3.325
3.127
2.308
Ms
N
MeO
O
N
Ts
1c
1.03
2.00
2.00
0.99
0.97
0.97
1.02
1.00
1.99
2.00
3.03
2.96
3.00
2.99
9.5
9.0
8.5
8.0
7.5
7.0
6.5
6.0
5.5
5.0
4.5
4.0
3.5
3.0
2.5
2.0
1.5
1.0
0.5
0.0
-0.5
f1 (ppm)

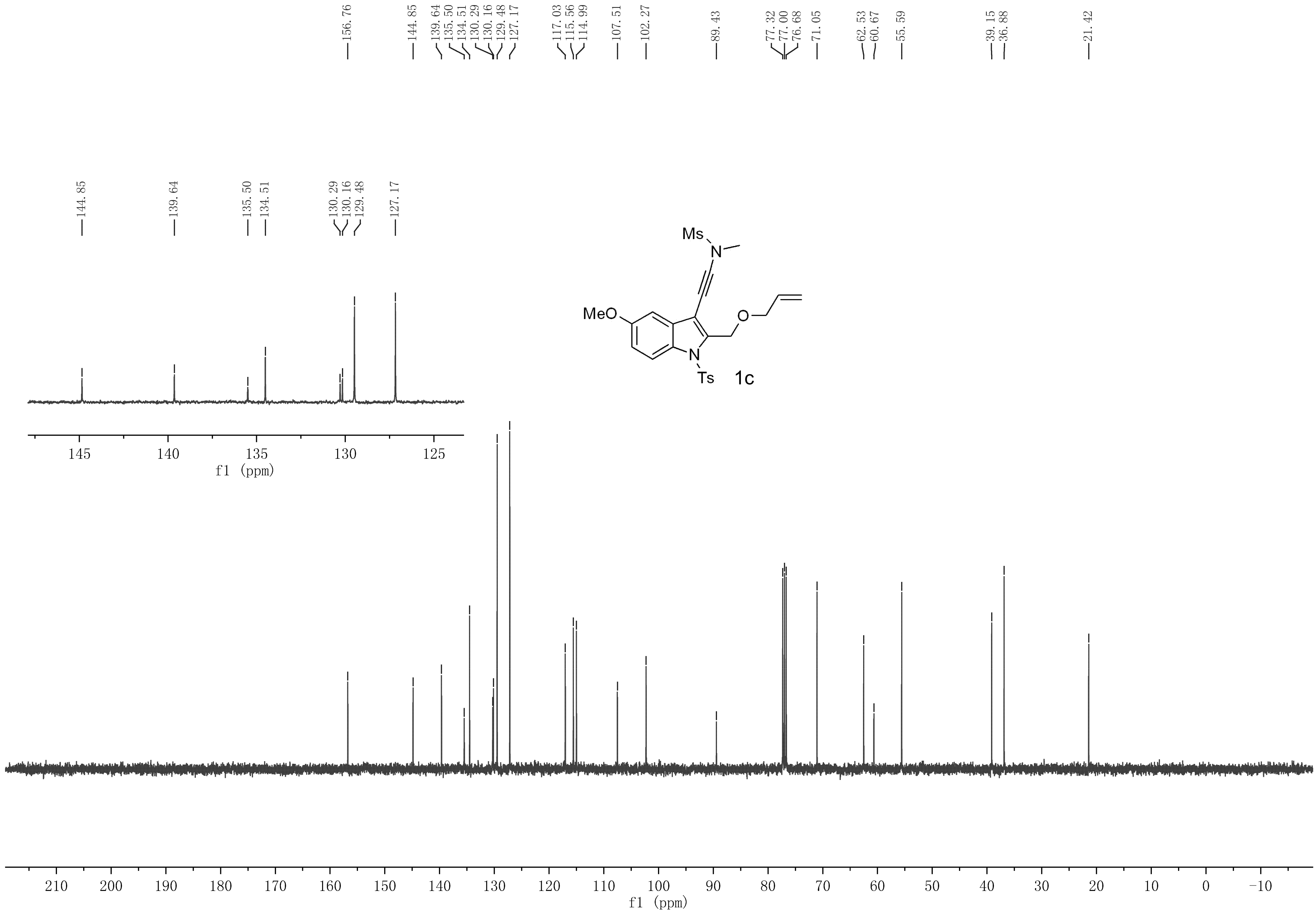

156.76
144.85
139.64
135.50
134.51
130.29
130.16
129.48
127.17
117.03
115.56
114.99
107.51
102.27
89.43
77.32
77.00
76.68
71.05
62.53
60.67
55.59
39.15
36.88
21.42
145 140 135 130 125
f1 (ppm)
Ms
N
MeO
O
N
Ts
1c
210 200 190 180 170 160 150 140 130 120 110 100 90 80 70 60 50 40 30 20 10 0 −10
f1 (ppm)

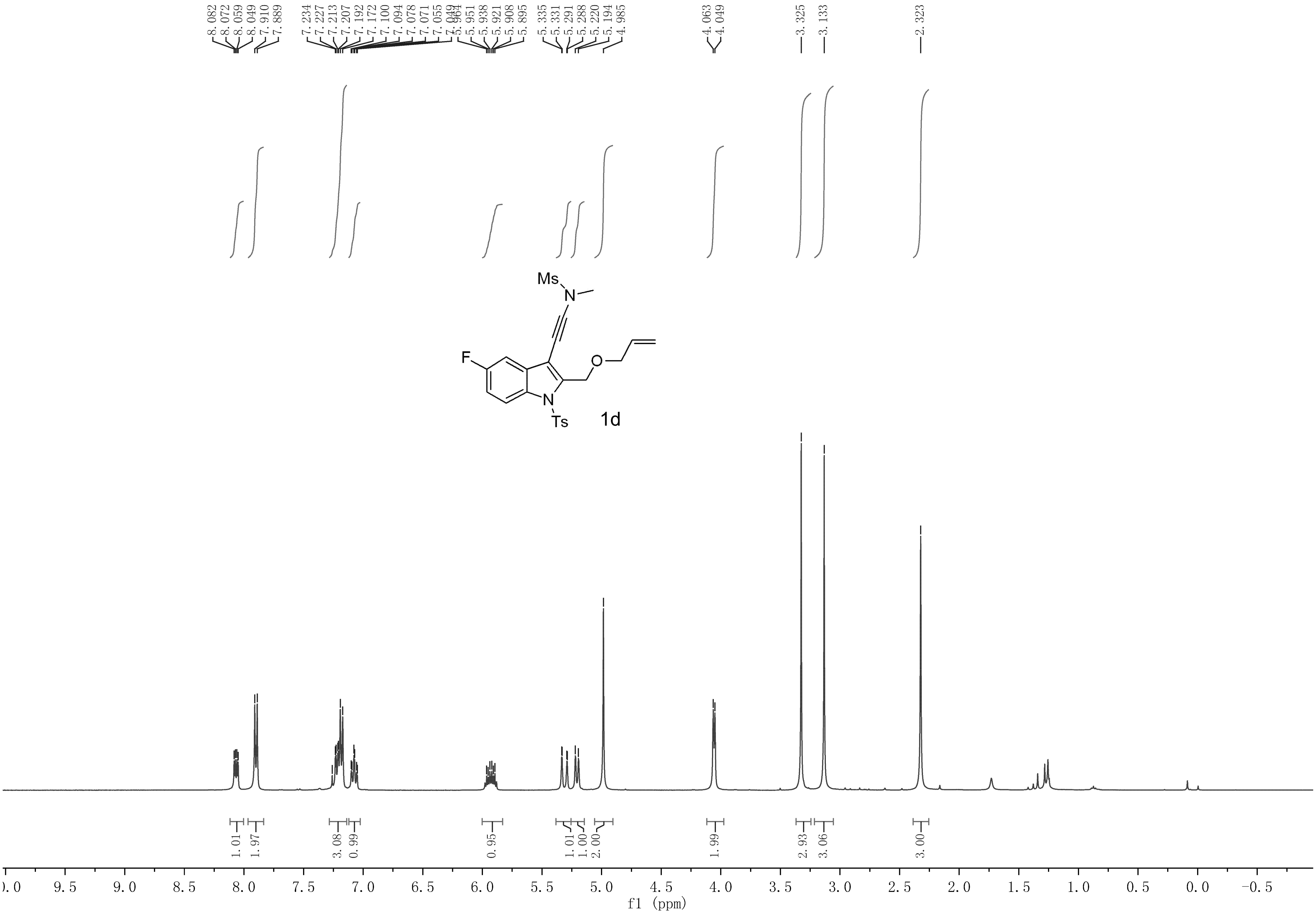
8.082
8.072
8.059
8.049
7.910
7.889
7.234
7.227
7.213
7.207
7.192
7.172
7.100
7.094
7.078
7.071
7.055
7.049
5.964
5.951
5.938
5.921
5.908
5.895
5.335
5.331
5.291
5.288
5.220
5.194
4.985
4.063
4.049
3.325
3.133
2.323
Ms
N
F
O
N
Ts
1d
1.01
1.97
3.08
0.99
0.95
1.01
1.00
2.00
1.99
2.93
3.06
3.00
).0 9.5 9.0 8.5 8.0 7.5 7.0 6.5 6.0 5.5 5.0 4.5 4.0 3.5 3.0 2.5 2.0 1.5 1.0 0.5 0.0 −0.5
f1 (ppm)

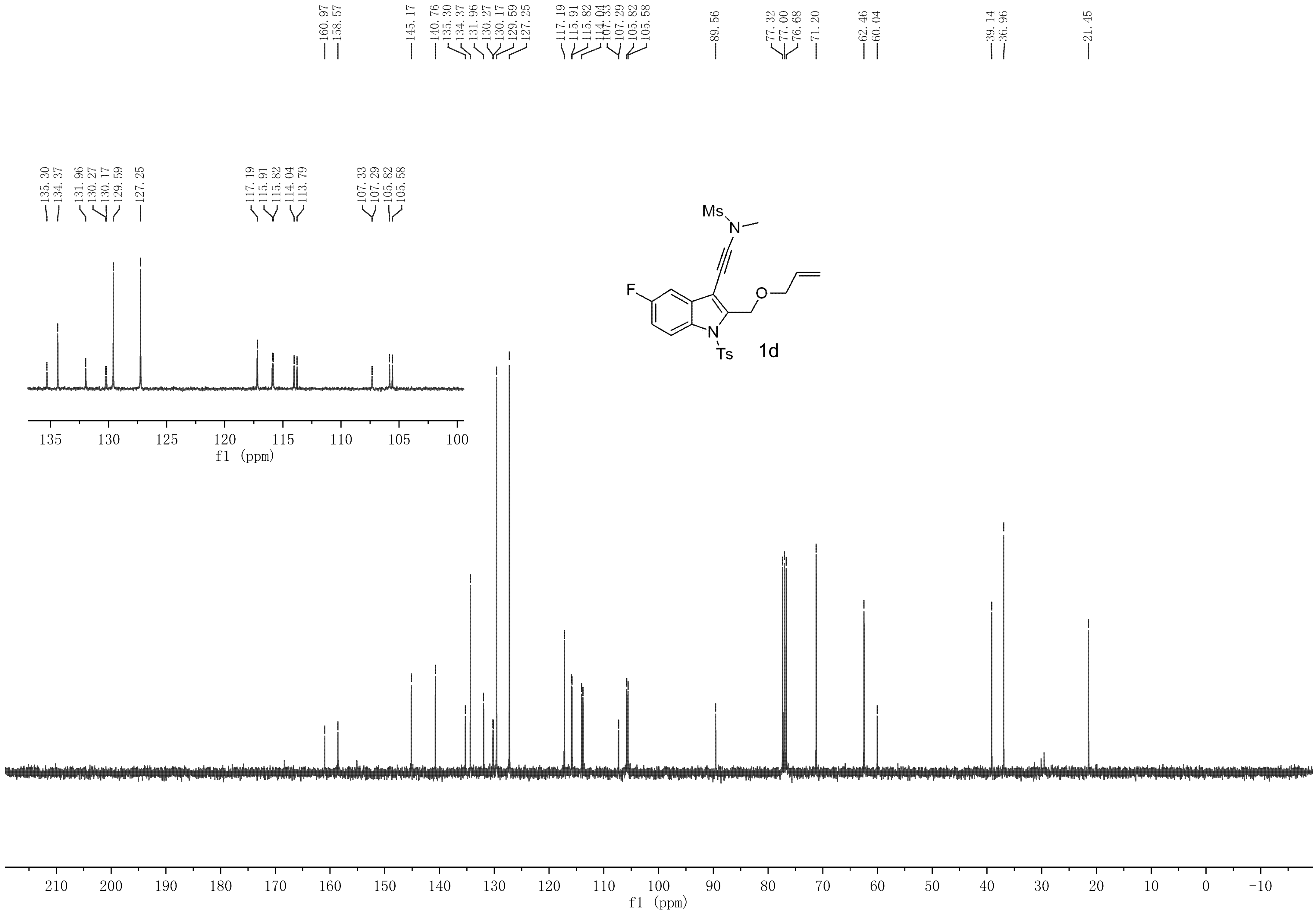
160.97
158.57
145.17
140.76
135.30
134.37
131.96
130.27
130.17
129.59
127.25
117.19
115.91
115.82
114.04
107.33
107.29
105.82
105.58
89.56
77.32
77.00
76.68
71.20
62.46
60.04
39.14
36.96
21.45
135.30
134.37
131.96
130.27
130.17
129.59
127.25
117.19
115.91
115.82
114.04
113.79
107.33
107.29
105.82
105.58
135 130 125 120 115 110 105 100
f1 (ppm)
Ms
N
F
O
N
Ts
1d
210 200 190 180 170 160 150 140 130 120 110 100 90 80 70 60 50 40 30 20 10 0 -10
f1 (ppm)

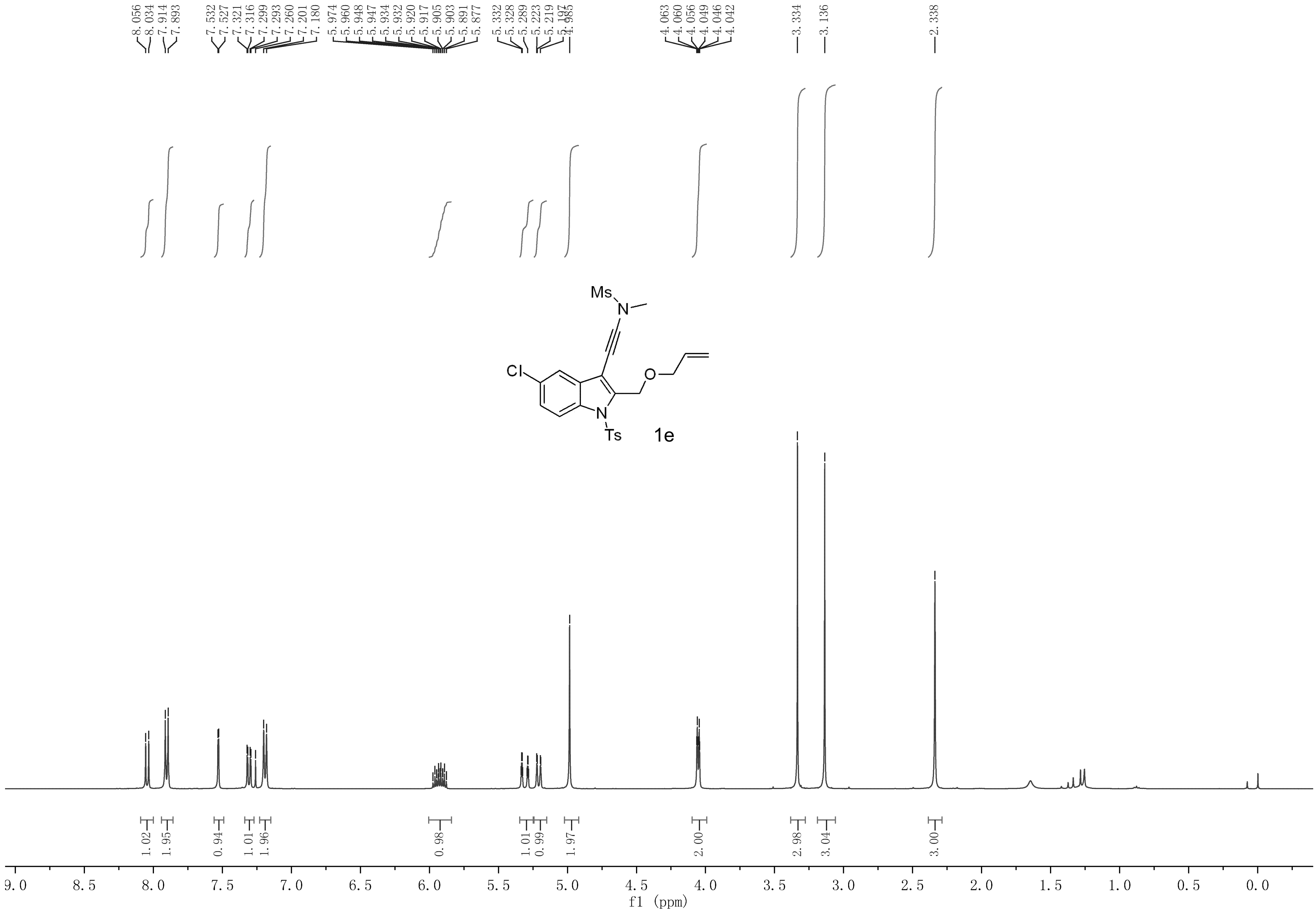
8.056
8.034
7.914
7.893
7.532
7.527
7.321
7.316
7.299
7.293
7.260
7.201
7.180
5.974
5.960
5.948
5.947
5.934
5.932
5.920
5.917
5.905
5.903
5.891
5.877
5.332
5.328
5.289
5.223
5.219
4.985
4.063
4.060
4.056
4.049
4.046
4.042
3.334
3.136
2.338
Ms
N
Cl
O
N
Ts
1e
1.02
1.95
0.94
1.01
1.96
0.98
1.01
0.99
1.97
2.00
2.98
3.04
3.00
9.0
8.5
8.0
7.5
7.0
6.5
6.0
5.5
5.0
4.5
4.0
3.5
3.0
2.5
2.0
1.5
1.0
0.5
0.0
f1 (ppm)

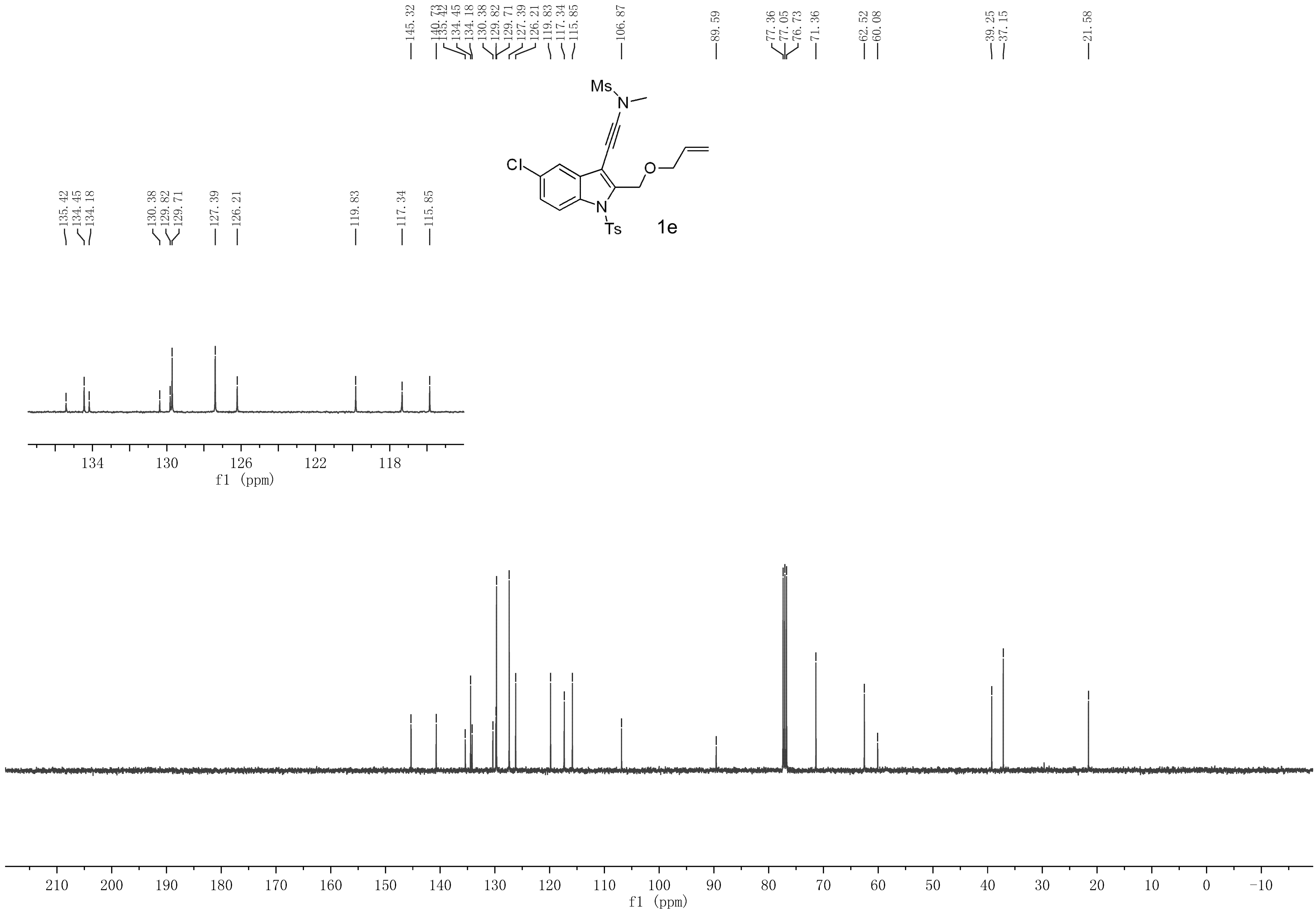
145.32
140.73
135.42
134.45
134.18
130.38
129.82
129.71
127.39
126.21
119.83
117.34
115.85
106.87
89.59
77.36
77.05
76.73
71.36
62.52
60.08
39.25
37.15
21.58
Ms
N
O
Cl
N
Ts
1e
134
130
126
122
118
f1 (ppm)
210
200
190
180
170
160
150
140
130
120
110
100
90
80
70
60
50
40
30
20
10
0
-10
f1 (ppm)

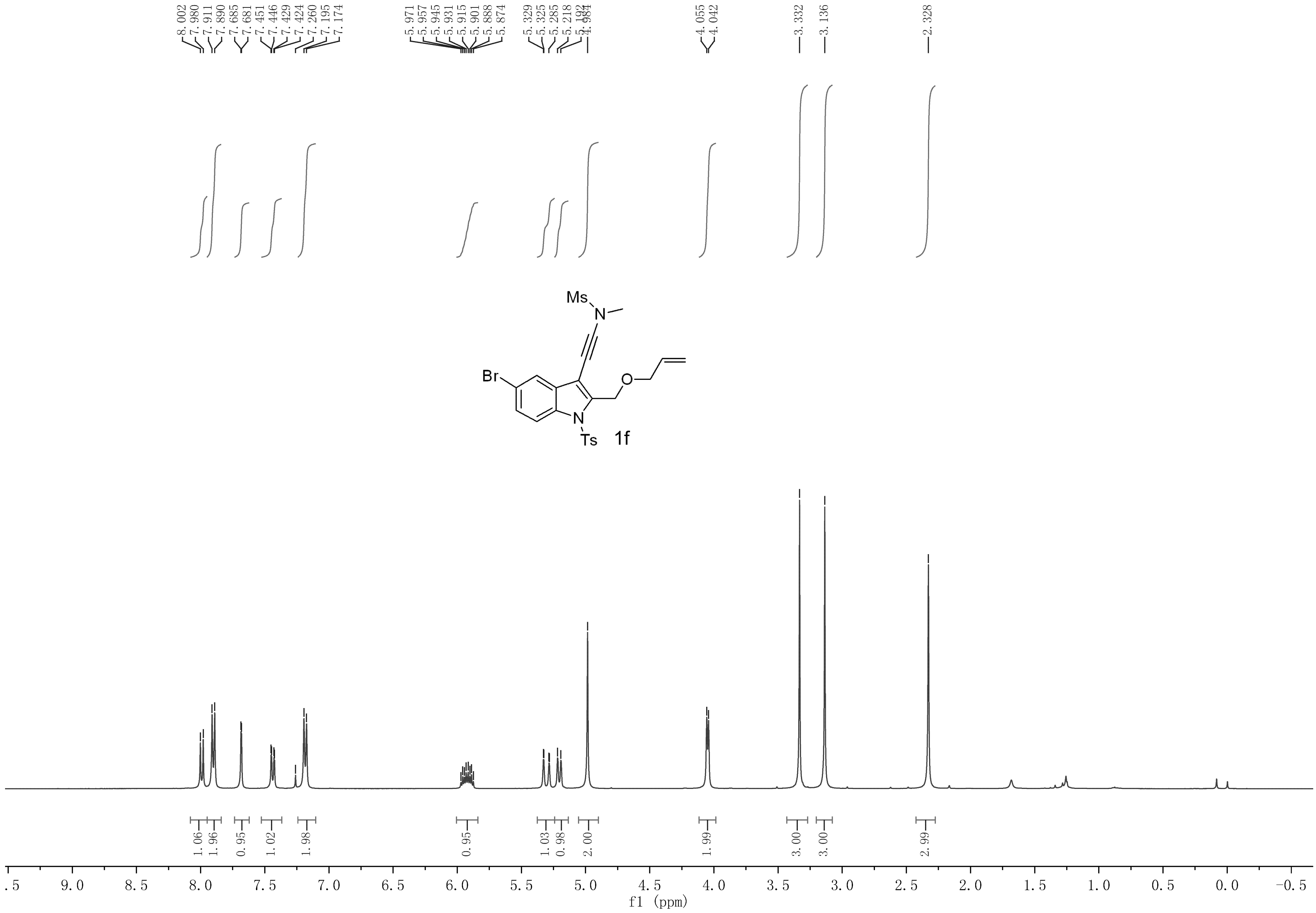
8.002
7.980
7.911
7.890
7.685
7.681
7.451
7.446
7.429
7.424
7.260
7.195
7.174
5.971
5.957
5.945
5.931
5.915
5.901
5.888
5.874
5.329
5.325
5.285
5.218
4.984
4.055
4.042
3.332
3.136
2.328
Ms
N
Br
O
N
Ts
1f
1.06
1.96
0.95
1.02
1.98
0.95
1.03
0.98
2.00
1.99
3.00
3.00
2.99
9.0
8.5
8.0
7.5
7.0
6.5
6.0
5.5
5.0
4.5
4.0
3.5
3.0
2.5
2.0
1.5
1.0
0.5
0.0
-0.5
f1 (ppm)

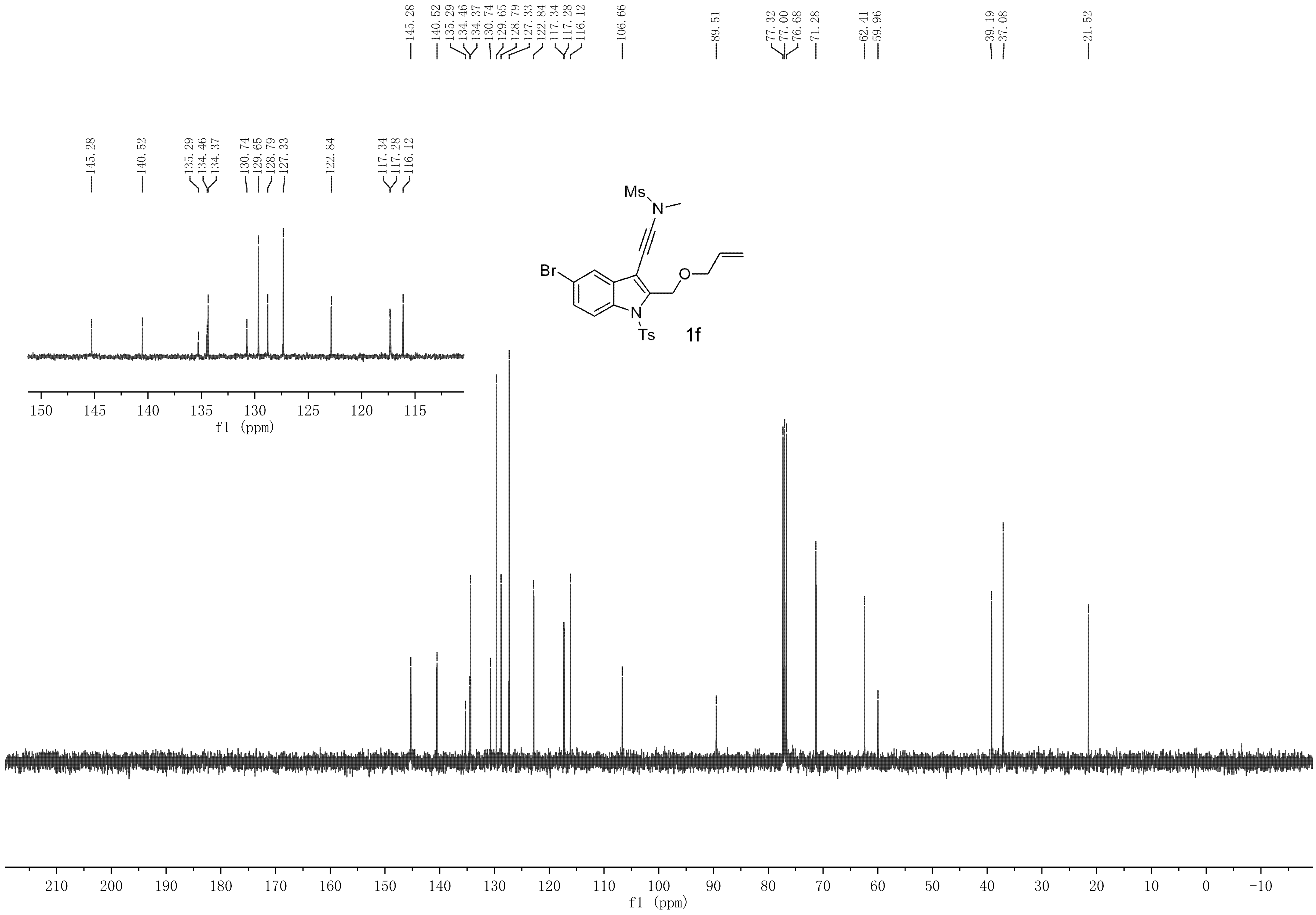
145.28
140.52
135.29
134.46
134.37
130.74
129.65
128.79
127.33
122.84
117.34
117.28
116.12
106.66
89.51
77.32
77.00
76.68
71.28
62.41
59.96
39.19
37.08
21.52
Ms
N
Br
O
N
Ts
1f
150 145 140 135 130 125 120 115
f1 (ppm)
210 200 190 180 170 160 150 140 130 120 110 100 90 80 70 60 50 40 30 20 10 0 −10
f1 (ppm)

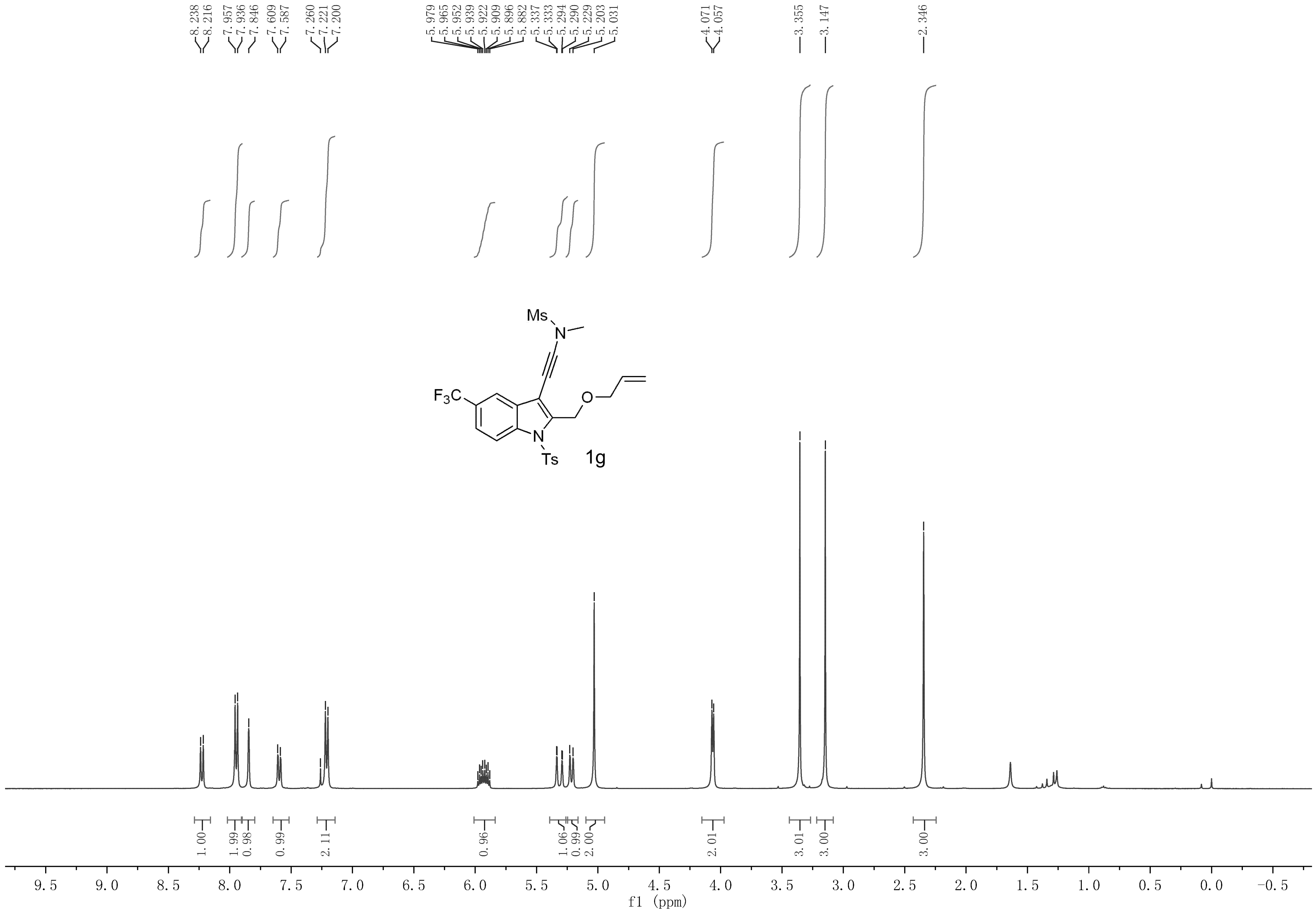

8.238
8.216
7.957
7.936
7.846
7.609
7.587
7.260
7.221
7.200
5.979
5.965
5.952
5.939
5.922
5.909
5.896
5.882
5.337
5.333
5.294
5.290
5.229
5.203
5.031
4.071
4.057
3.355
3.147
2.346
Ms
N
F3C
O
N
Ts
1g
1.00
1.99
0.98
0.99
2.11
0.96
1.06
0.99
2.00
2.01
3.01
3.00
3.00
9.5
9.0
8.5
8.0
7.5
7.0
6.5
6.0
5.5
5.0
4.5
4.0
3.5
3.0
2.5
2.0
1.5
1.0
0.5
0.0
-0.5
f1 (ppm)

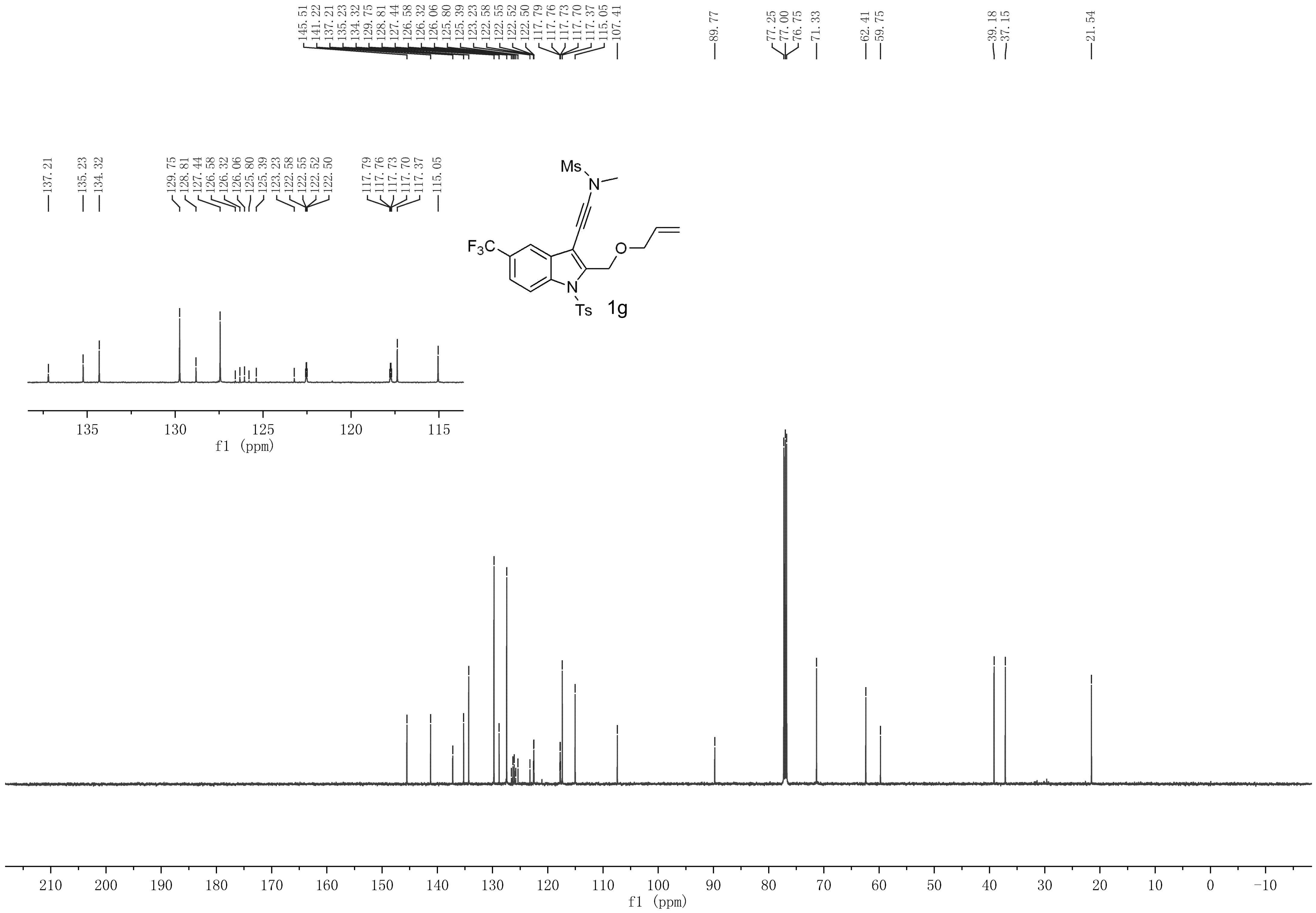
145.51
141.22
137.21
135.23
134.32
129.75
128.81
127.44
126.58
126.32
126.06
125.80
125.39
123.23
122.58
122.55
122.52
122.50
117.79
117.76
117.73
117.70
117.37
115.05
107.41
89.77
77.25
77.00
76.75
71.33
62.41
59.75
39.18
37.15
21.54
Ms
N
F3C
O
N
Ts
1g
f1 (ppm)

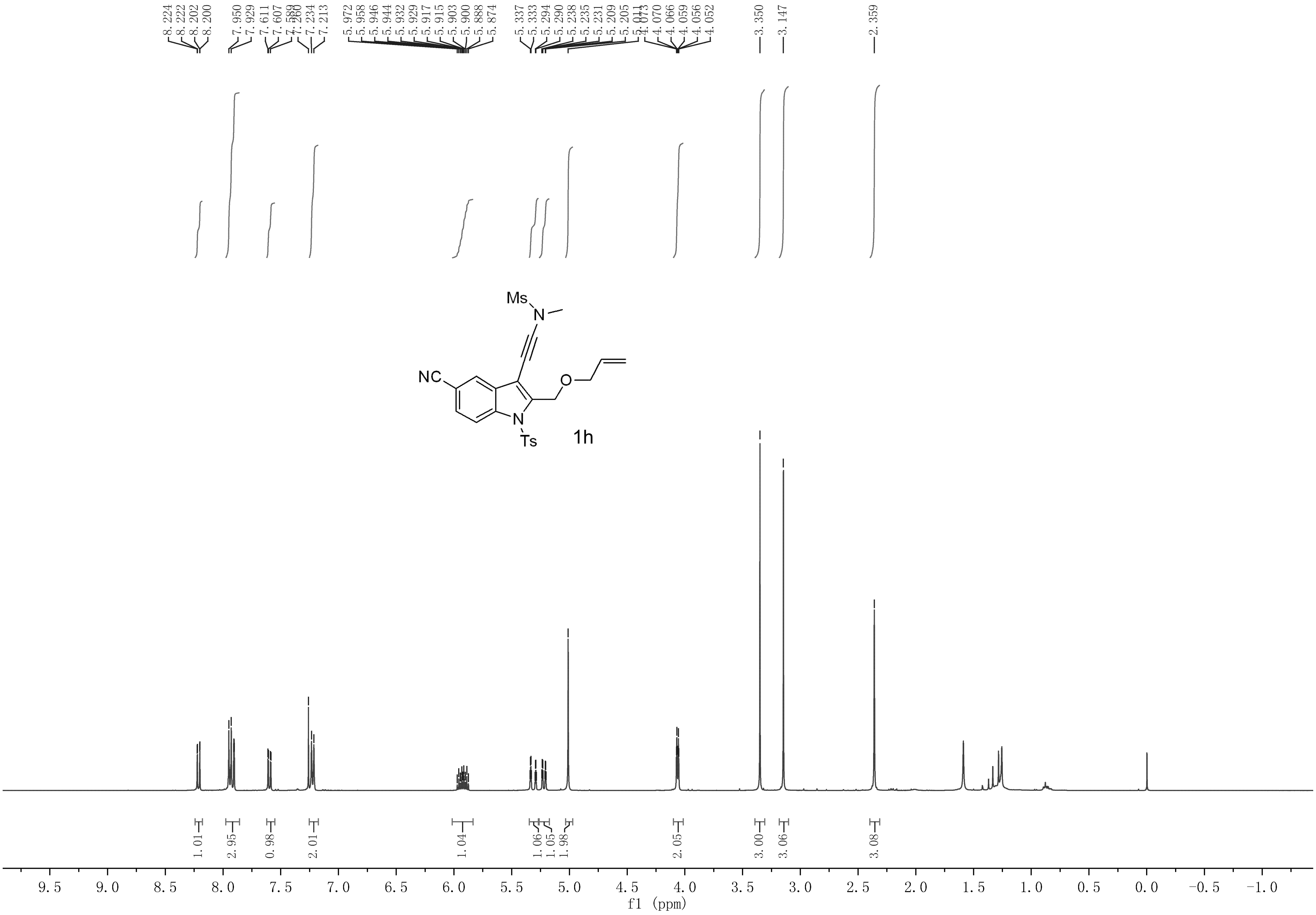
8.224
8.222
8.202
8.200
7.950
7.929
7.611
7.607
7.589
7.260
7.234
7.213
5.972
5.958
5.946
5.944
5.932
5.929
5.917
5.915
5.903
5.900
5.888
5.874
5.337
5.333
5.294
5.290
5.238
5.235
5.231
5.209
5.205
5.011
4.073
4.070
4.066
4.059
4.056
4.052
3.350
3.147
2.359
Ms
N
NC
O
N
Ts
1h
1.01
2.95
0.98
2.01
1.04
1.06
1.05
1.98
2.05
3.00
3.06
3.08
9.5
9.0
8.5
8.0
7.5
7.0
6.5
6.0
5.5
5.0
4.5
4.0
3.5
3.0
2.5
2.0
1.5
1.0
0.5
0.0
-0.5
-1.0
f1 (ppm)

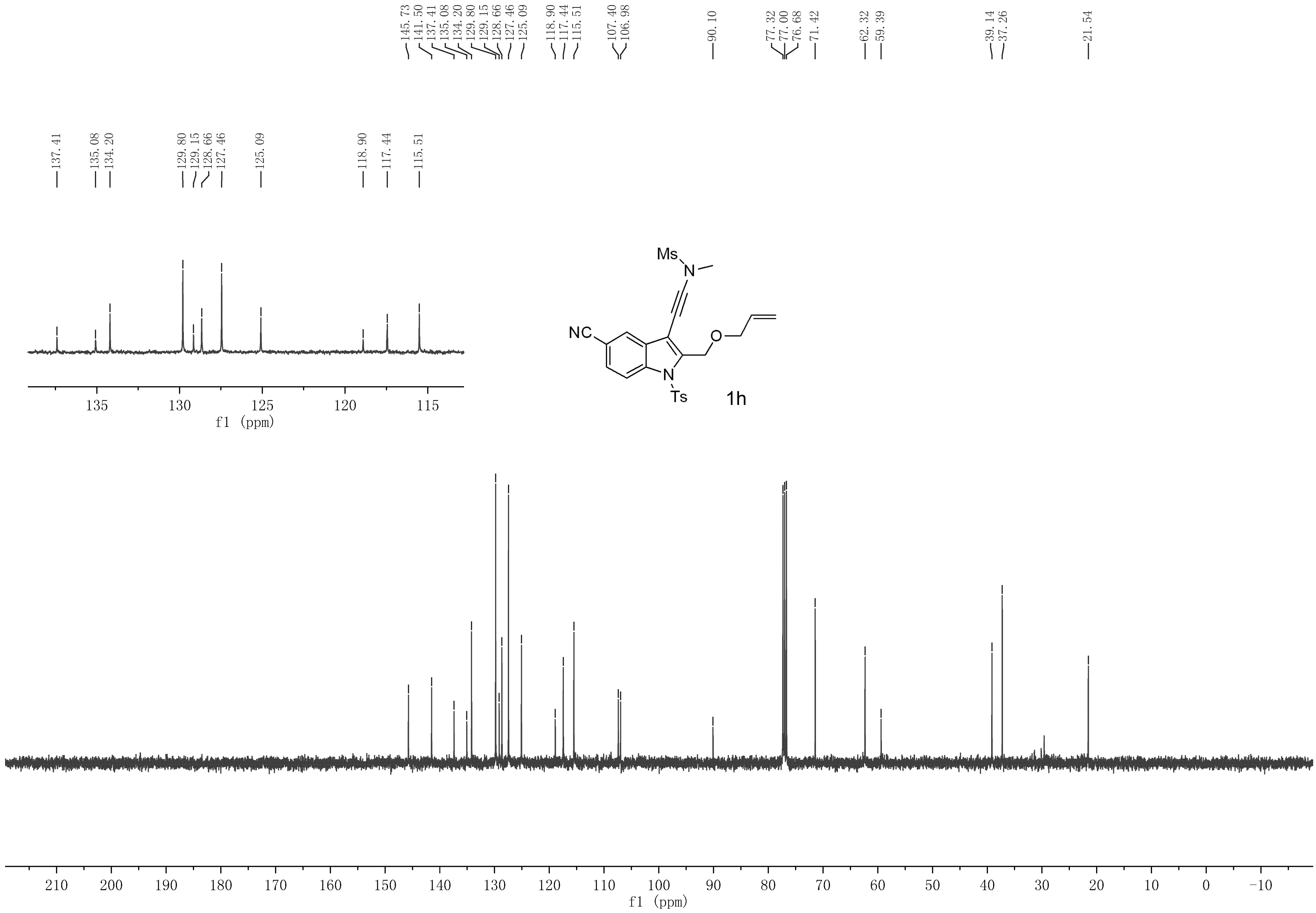
145.73
141.50
137.41
135.08
134.20
129.80
129.15
128.66
127.46
125.09
118.90
117.44
115.51
107.40
106.98
90.10
77.32
77.00
76.68
71.42
62.32
59.39
39.14
37.26
21.54
Ms
N
NC
O
N
Ts
1h
135
130
125
120
115
f1 (ppm)
210
200
190
180
170
160
150
140
130
120
110
100
90
80
70
60
50
40
30
20
10
0
−10
f1 (ppm)

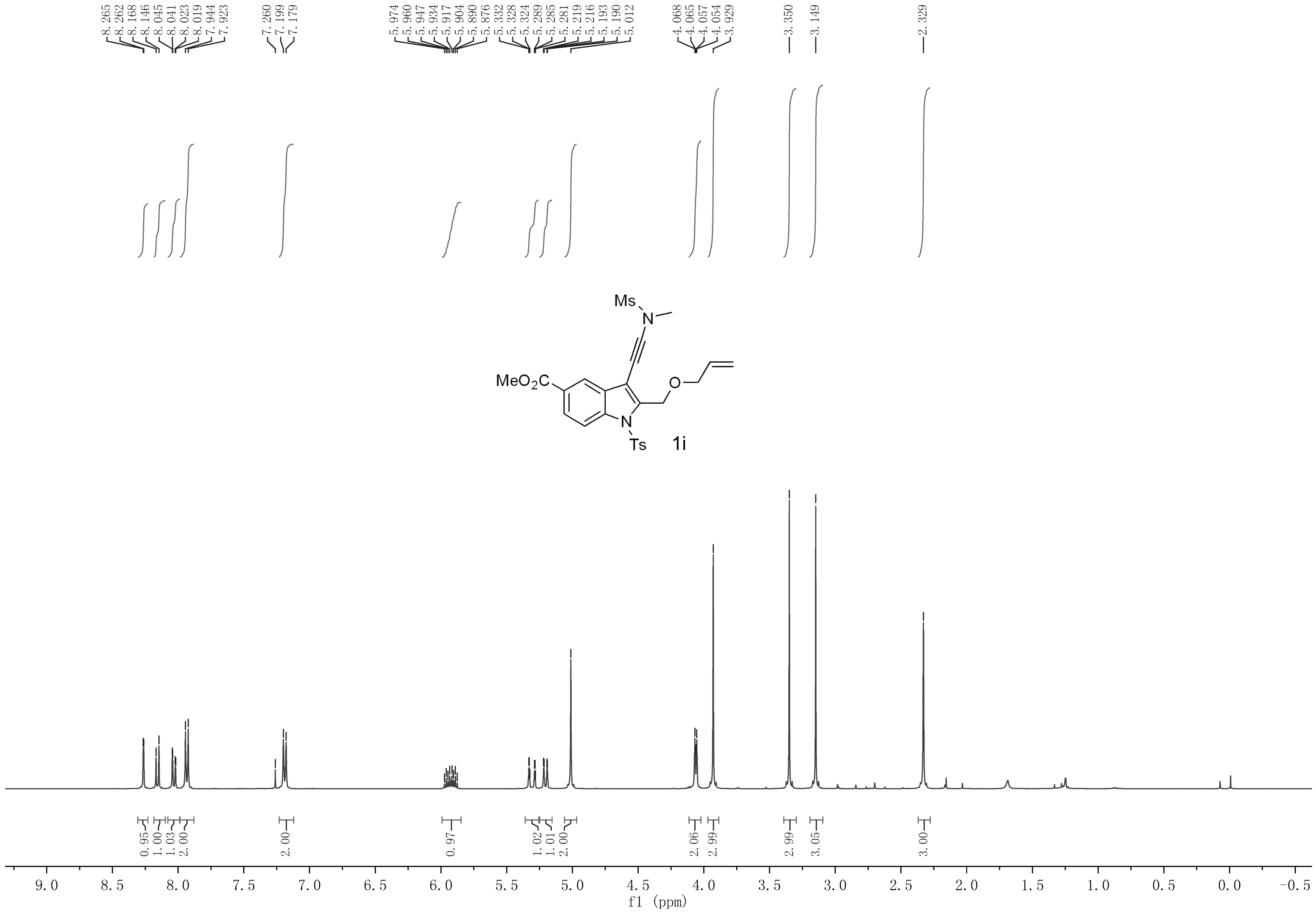

8.265
8.262
8.168
8.146
8.045
8.041
8.023
8.019
7.944
7.923
7.260
7.199
7.179
5.974
5.960
5.947
5.934
5.917
5.904
5.890
5.876
5.332
5.328
5.324
5.289
5.285
5.281
5.219
5.216
5.193
5.190
5.012
4.068
4.065
4.057
4.054
3.929
3.350
3.149
2.329
Ms
N
MeO2C
O
N
Ts
1i
0.95
1.00
1.03
2.00
2.00
0.97
1.02
1.01
2.00
2.06
2.99
2.99
3.05
3.00
9.0
8.5
8.0
7.5
7.0
6.5
6.0
5.5
5.0
4.5
4.0
3.5
3.0
2.5
2.0
1.5
1.0
0.5
0.0
-0.5
f1 (ppm)

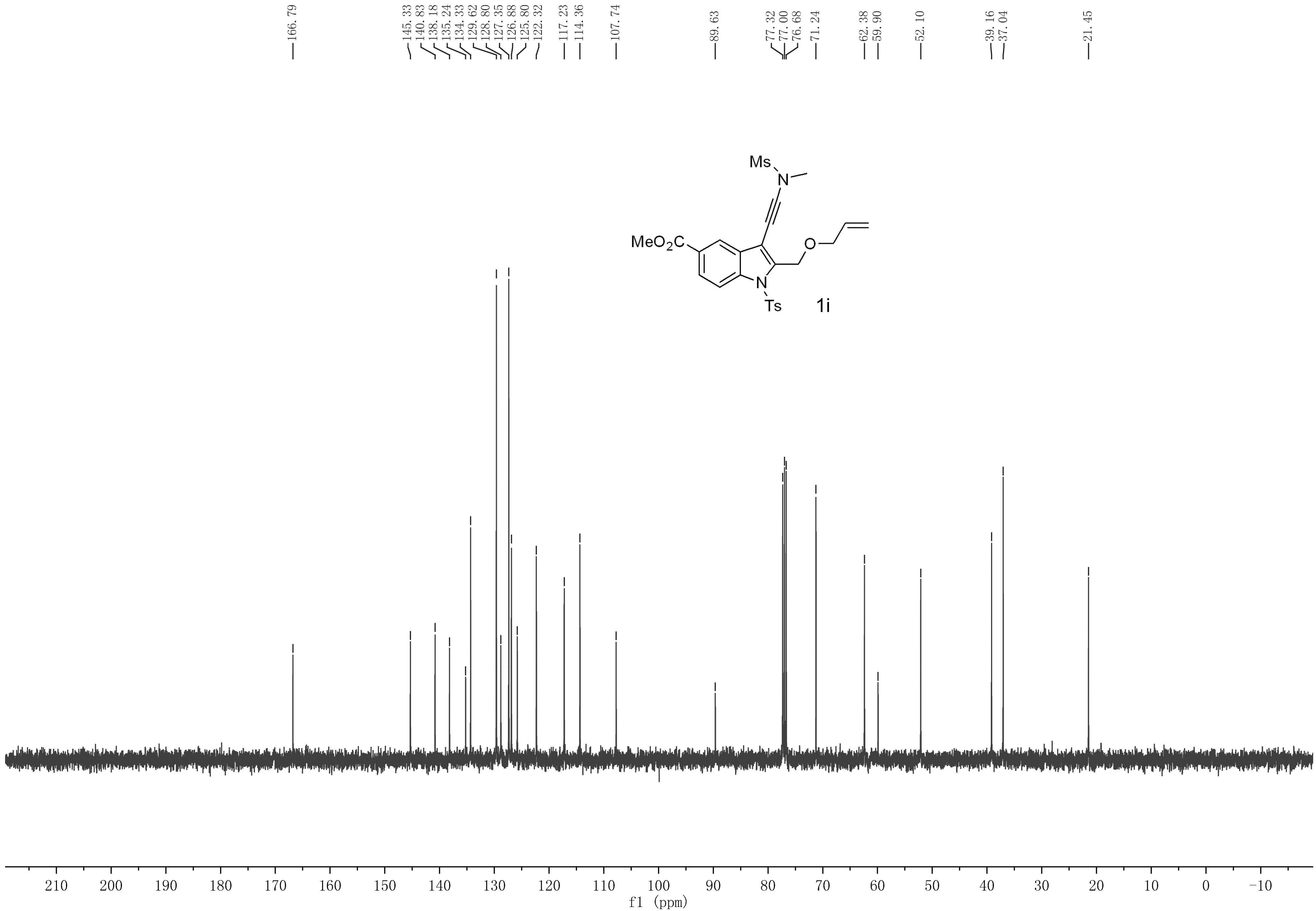

166.79
145.33
140.83
138.18
135.24
134.33
129.62
128.80
127.35
126.88
125.80
122.32
117.23
114.36
107.74
89.63
77.32
77.00
76.68
71.24
62.38
59.90
52.10
39.16
37.04
21.45
Ms
N
MeO2C
O
N
Ts
1i
210 200 190 180 170 160 150 140 130 120 110 100 90 80 70 60 50 40 30 20 10 0 −10
f1 (ppm)

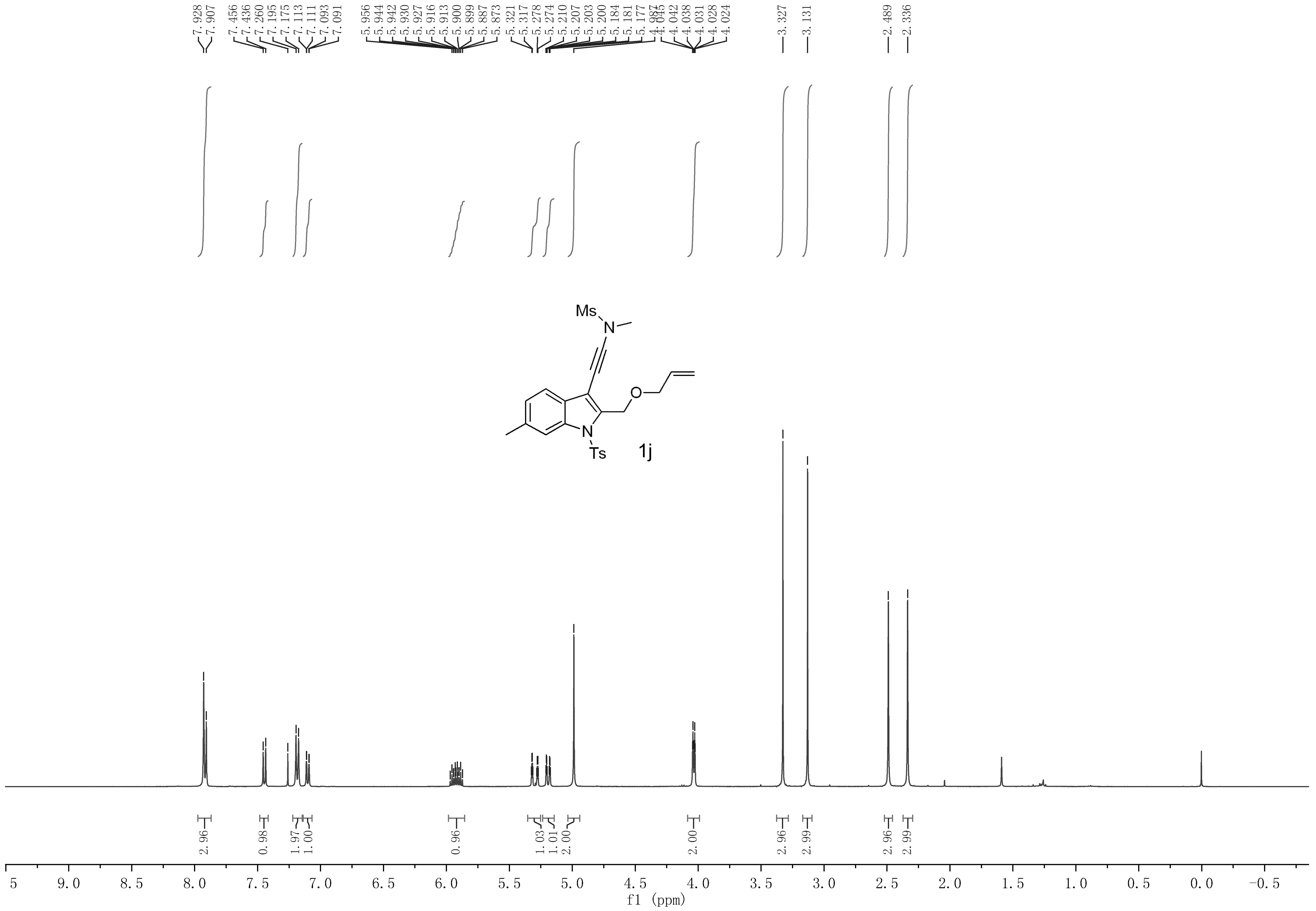
7.928
7.907
7.456
7.436
7.260
7.195
7.175
7.113
7.111
7.093
7.091
5.956
5.944
5.942
5.930
5.927
5.916
5.913
5.900
5.899
5.887
5.873
5.321
5.317
5.278
5.274
5.210
5.207
5.203
5.200
5.184
5.181
5.177
4.987
4.045
4.042
4.038
4.031
4.028
4.024
3.327
3.131
2.489
2.336
Ms
N
N
Ts
1j
2.96
0.98
1.97
1.00
0.96
1.03
1.01
2.00
2.00
2.96
2.99
2.96
2.99
f1 (ppm)

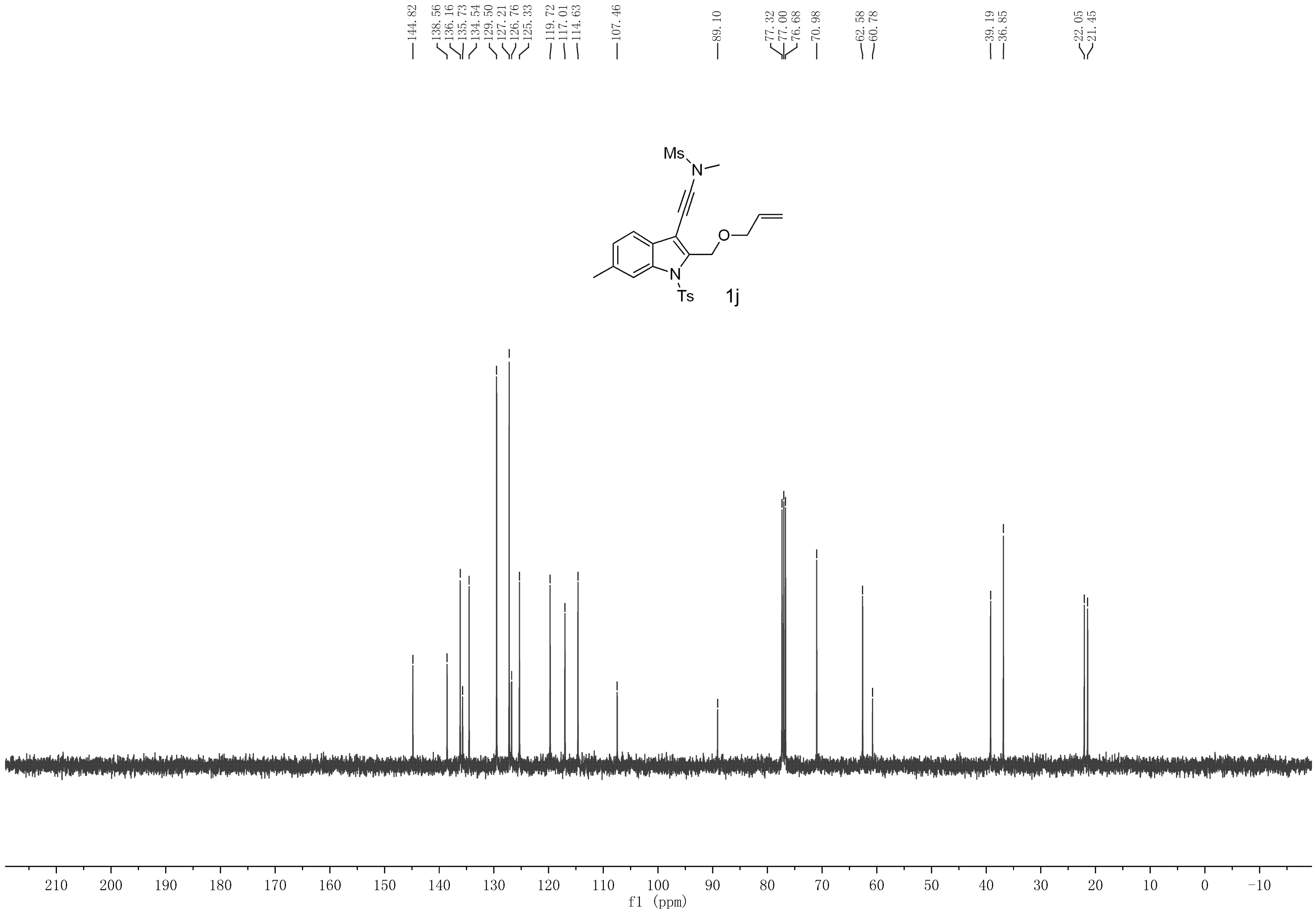
144.82
138.56
136.16
135.73
134.54
129.50
127.21
126.76
125.33
119.72
117.01
114.63
107.46
89.10
77.32
77.00
76.68
70.98
62.58
60.78
39.19
36.85
22.05
21.45
Ms
N
Ts
O
1j
210 200 190 180 170 160 150 140 130 120 110 100 90 80 70 60 50 40 30 20 10 0 −10
f1 (ppm)

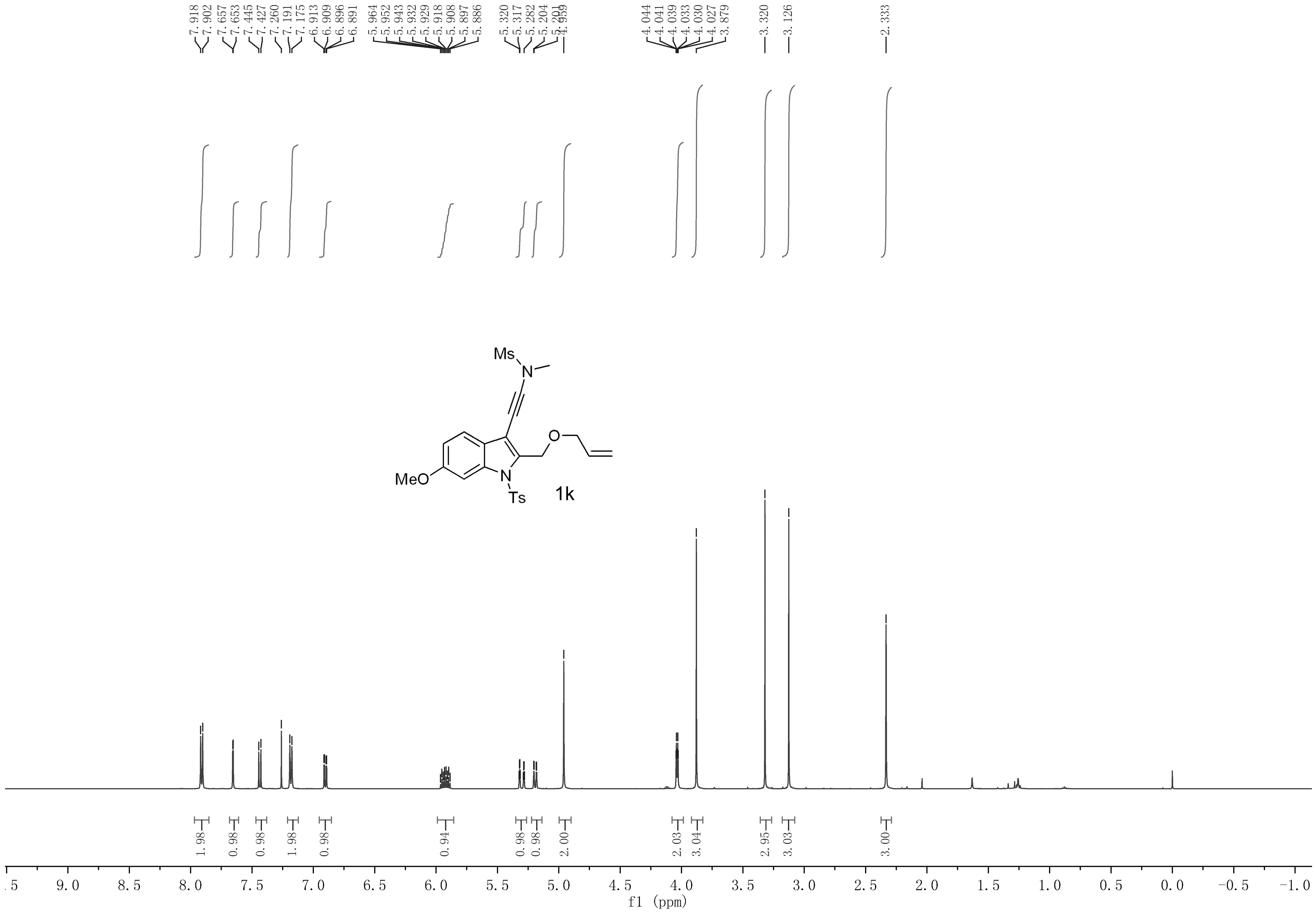
7.918
7.902
7.657
7.653
7.445
7.427
7.260
7.191
7.175
6.913
6.909
6.896
6.891
5.964
5.952
5.943
5.932
5.929
5.918
5.908
5.897
5.886
5.320
5.317
5.282
5.204
5.201
4.959
4.044
4.041
4.039
4.033
4.030
4.027
3.879
3.320
3.126
2.333
Ms
N
MeO
N
Ts
O
1k
1.98
0.98
0.98
1.98
0.98
0.94
0.98
0.98
2.00
2.03
3.04
2.95
3.03
3.00
9.0
8.5
8.0
7.5
7.0
6.5
6.0
5.5
5.0
4.5
4.0
3.5
3.0
2.5
2.0
1.5
1.0
0.5
0.0
-0.5
-1.0
f1 (ppm)

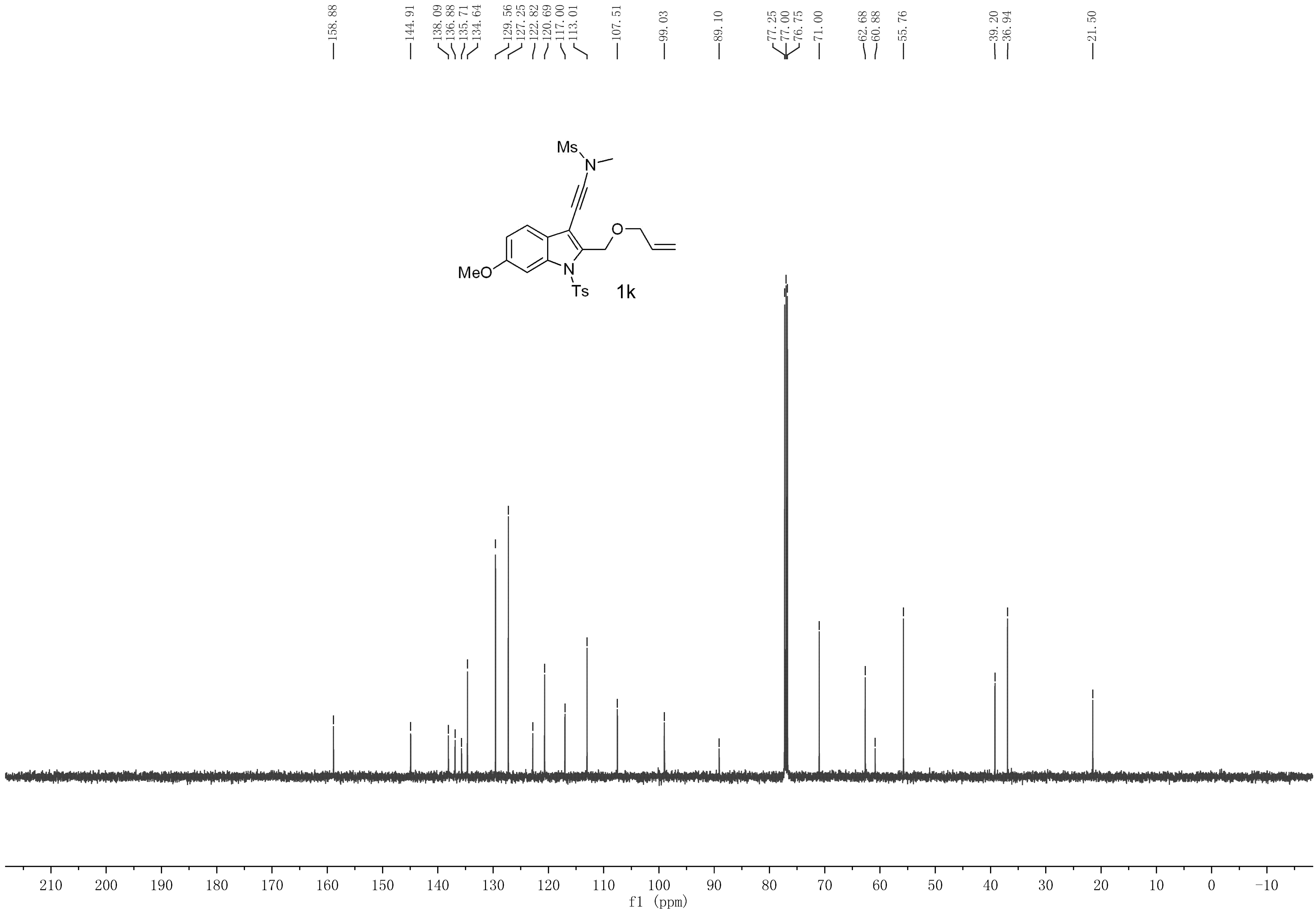
158.88
144.91
138.09
136.88
135.71
134.64
129.56
127.25
122.82
120.69
117.00
113.01
107.51
99.03
89.10
77.25
77.00
76.75
71.00
62.68
60.88
55.76
39.20
36.94
21.50
Ms
N
O
MeO
N
Ts
1k
210
200
190
180
170
160
150
140
130
120
110
100
90
80
70
60
50
40
30
20
10
0
-10
f1 (ppm)

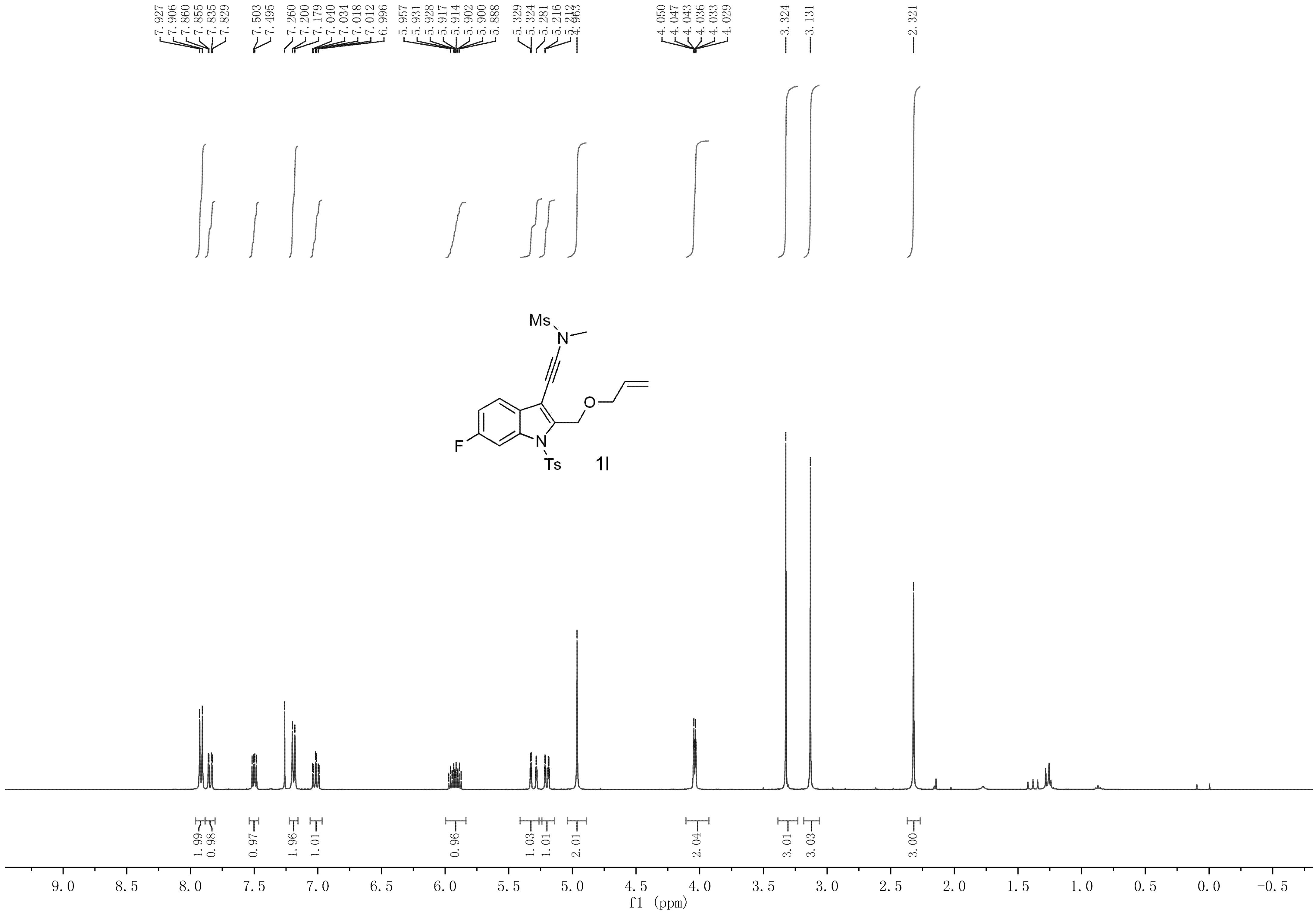
7.927
7.906
7.860
7.855
7.835
7.829
7.503
7.495
7.260
7.200
7.179
7.040
7.034
7.018
7.012
6.996
5.957
5.931
5.928
5.917
5.914
5.902
5.900
5.888
5.329
5.324
5.281
5.216
4.963
4.050
4.047
4.043
4.036
4.033
4.029
3.324
3.131
2.321
Ms
N
O
F
N
Ts
1l
1.99
0.98
0.97
1.96
1.01
0.96
1.03
1.01
2.01
2.04
3.01
3.03
3.00
9.0
8.5
8.0
7.5
7.0
6.5
6.0
5.5
5.0
4.5
4.0
3.5
3.0
2.5
2.0
1.5
1.0
0.5
0.0
−0.5
f1 (ppm)

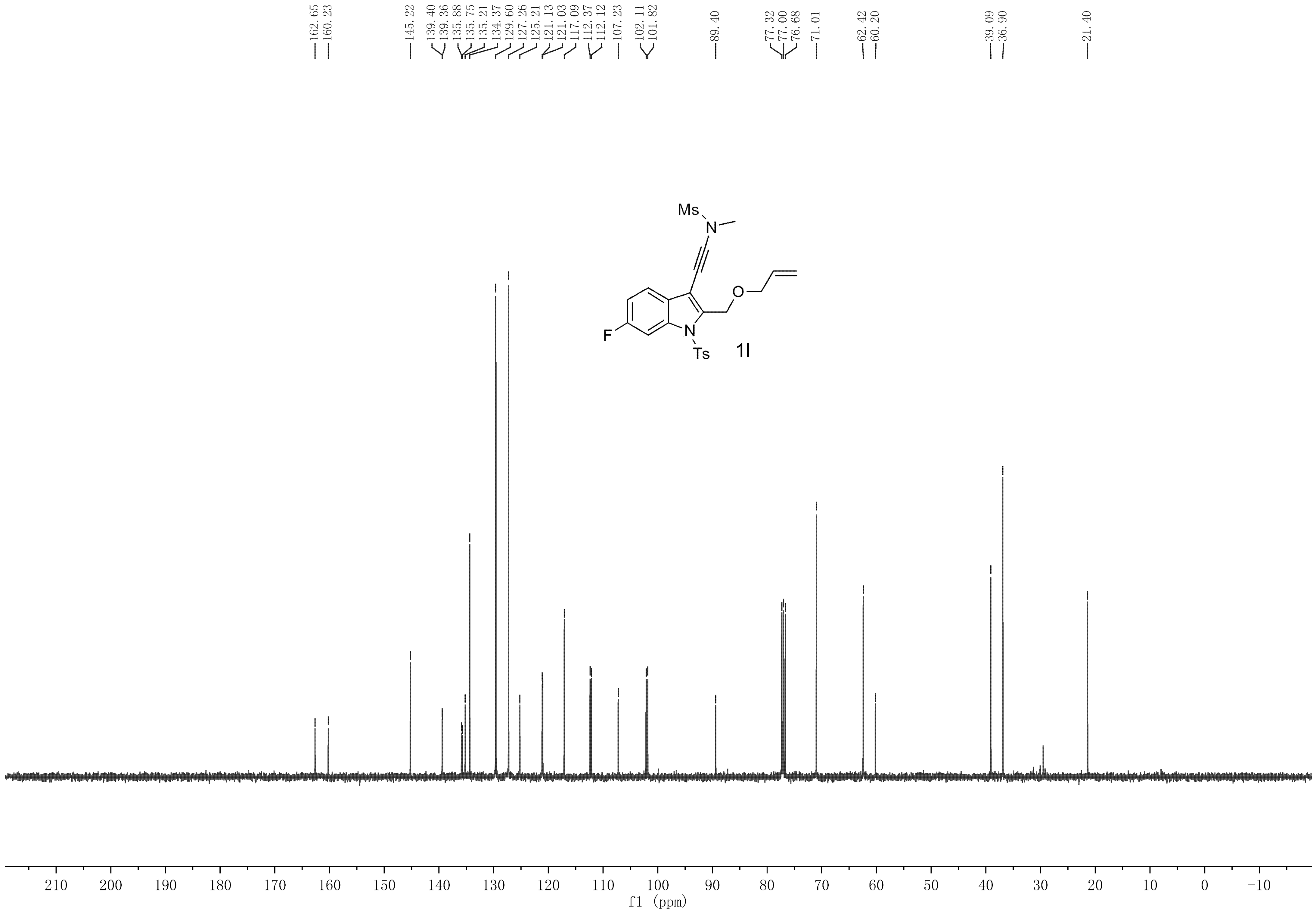
162.65
160.23
145.22
139.40
139.36
135.88
135.75
135.21
134.37
129.60
127.26
125.21
121.13
121.03
117.09
112.37
112.12
107.23
102.11
101.82
89.40
77.32
77.00
76.68
71.01
62.42
60.20
39.09
36.90
21.40
Ms
N
O
F
N
Ts
1l
210
200
190
180
170
160
150
140
130
120
110
100
90
80
70
60
50
40
30
20
10
0
−10
f1 (ppm)

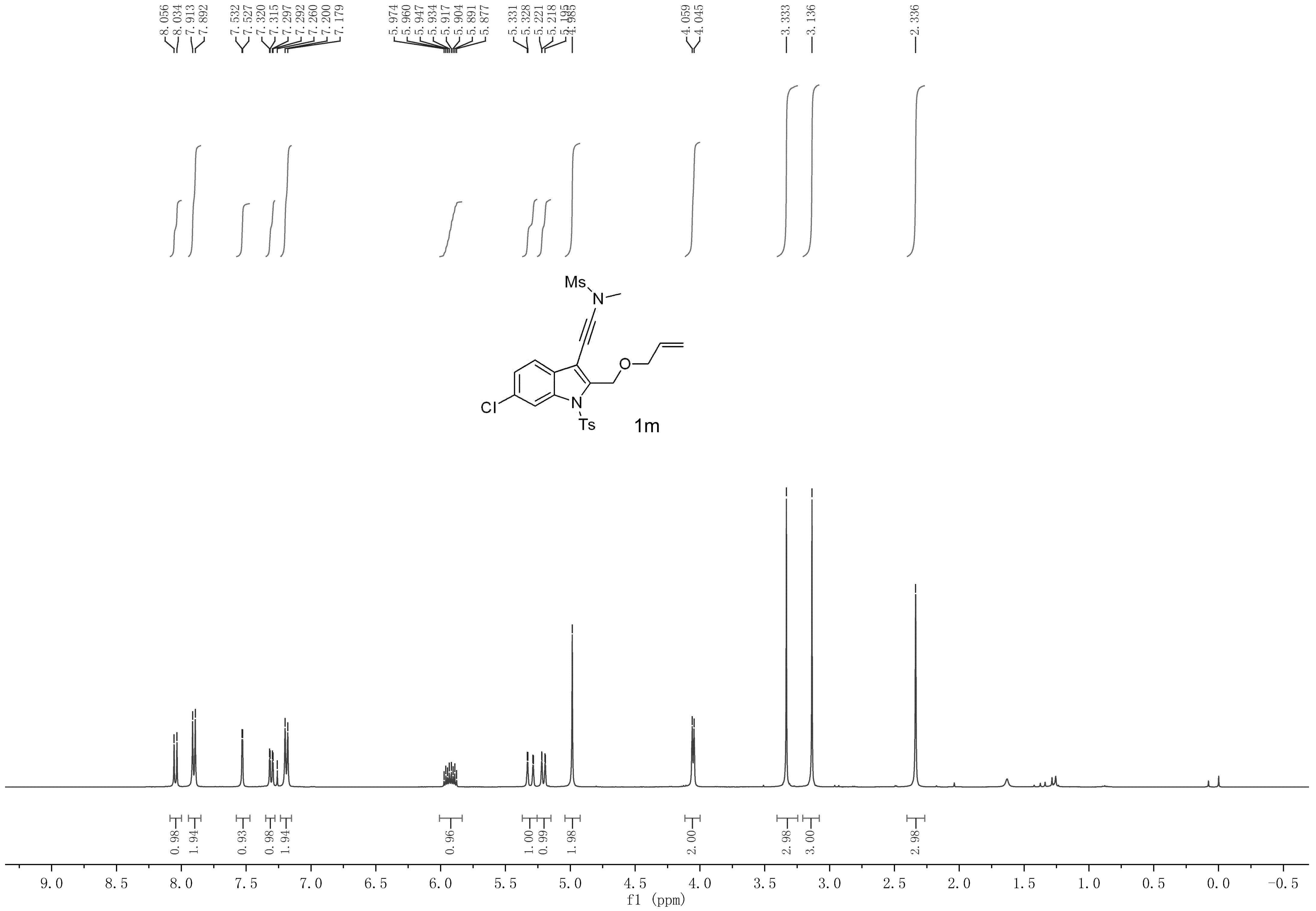
8.056
8.034
7.913
7.892
7.532
7.527
7.320
7.315
7.297
7.292
7.260
7.200
7.179
5.974
5.960
5.947
5.934
5.917
5.904
5.891
5.877
5.331
5.328
5.221
5.218
5.195
4.985
4.059
4.045
3.333
3.136
2.336
Ms
N
O
Cl
N
Ts
1m
0.98
1.94
0.93
0.98
1.94
0.96
1.00
0.99
1.98
2.00
2.98
3.00
2.98
9.0 8.5 8.0 7.5 7.0 6.5 6.0 5.5 5.0 4.5 4.0 3.5 3.0 2.5 2.0 1.5 1.0 0.5 0.0 -0.5
f1 (ppm)

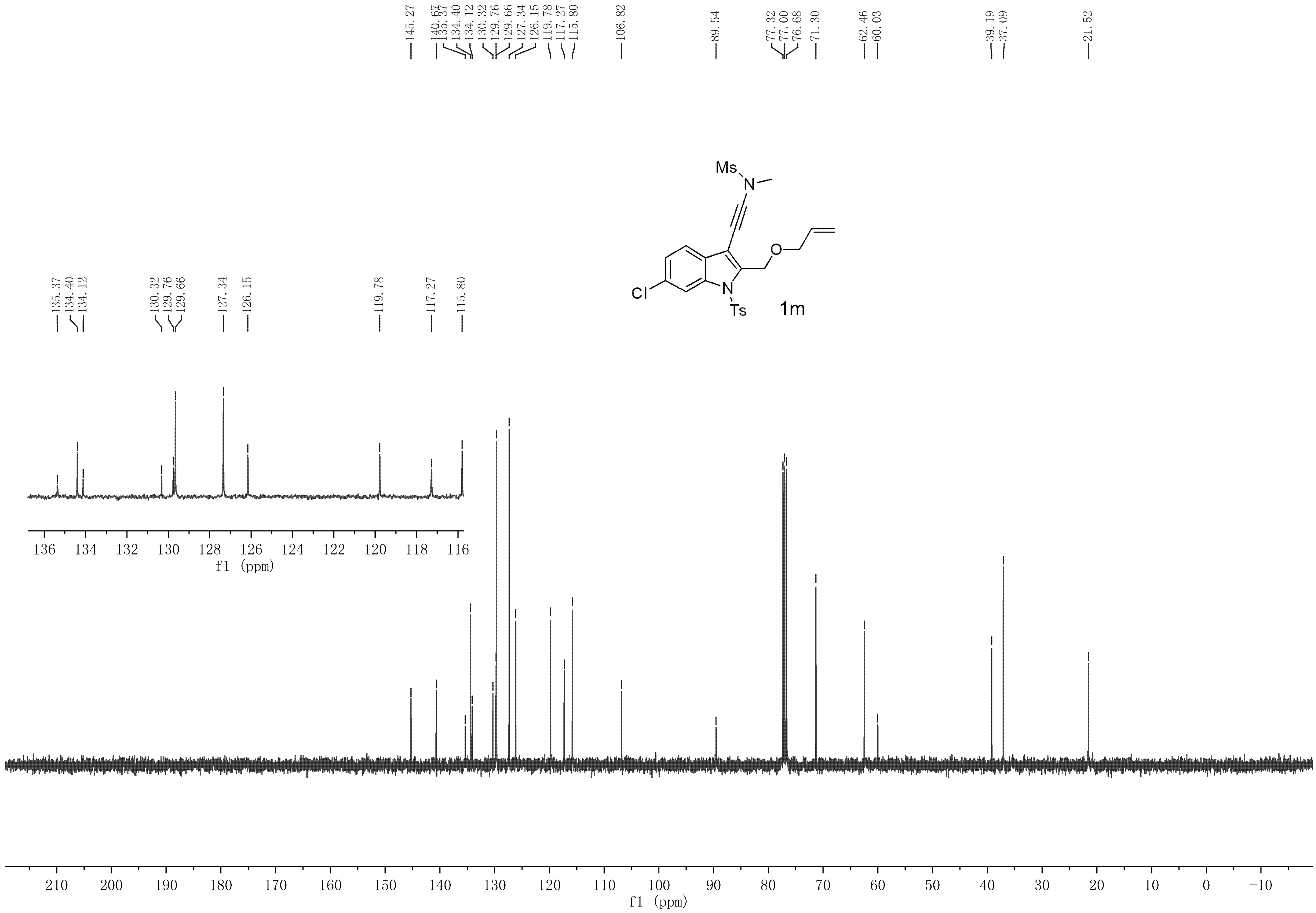
145.27
140.67
135.37
134.40
134.12
130.32
129.76
129.66
127.34
126.15
119.78
117.27
115.80
106.82
89.54
77.32
77.00
76.68
71.30
62.46
60.03
39.19
37.09
21.52
Ms
N
O
Cl
N
Ts
1m
f1 (ppm)
f1 (ppm)

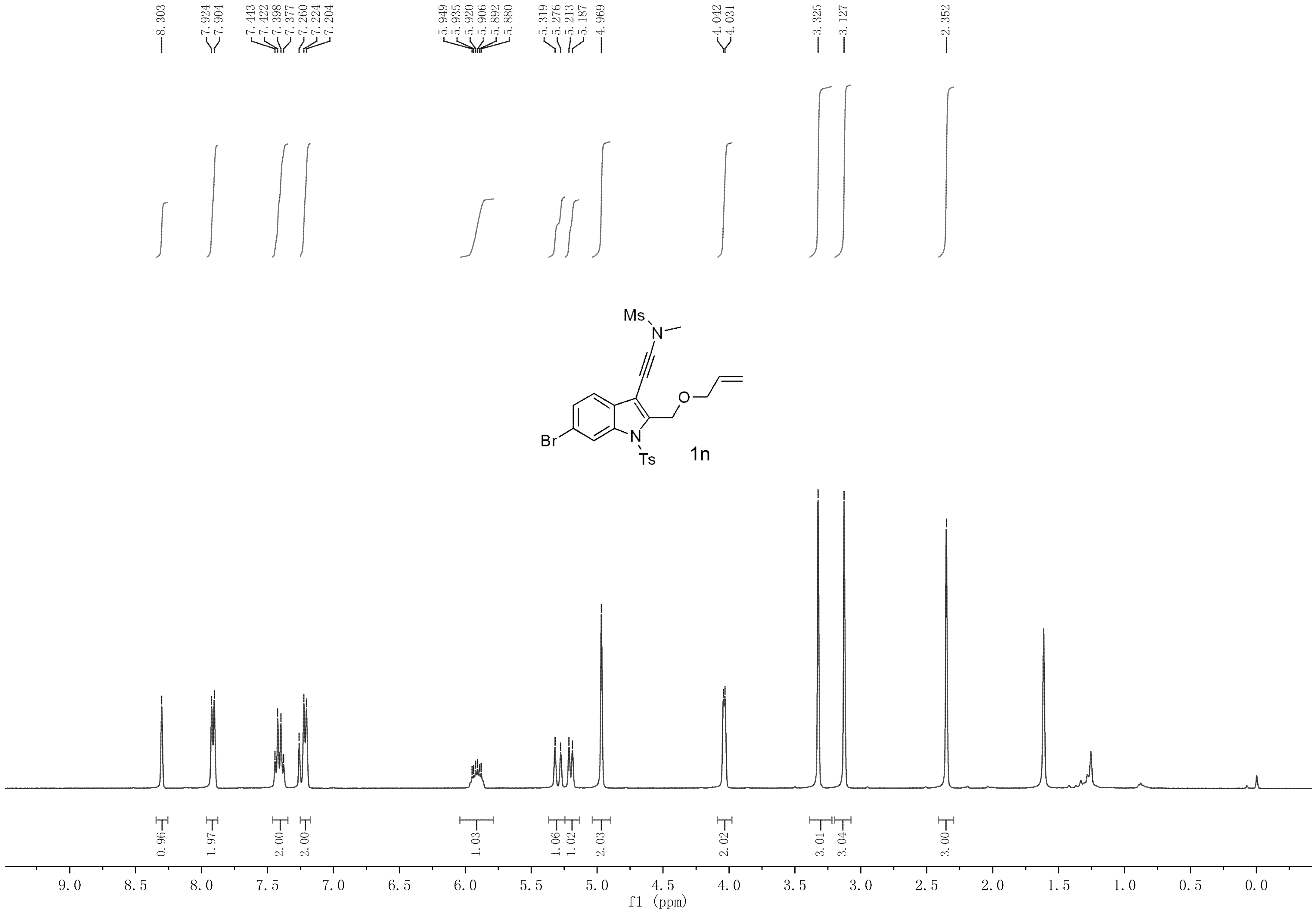
8.303
7.924
7.904
7.443
7.422
7.398
7.377
7.260
7.224
7.204
5.949
5.935
5.920
5.906
5.892
5.880
5.319
5.276
5.213
5.187
4.969
4.042
4.031
3.325
3.127
2.352
Ms
N
O
Br
N
Ts
1n
0.96
1.97
2.00
2.00
1.03
1.06
1.02
2.03
2.02
3.01
3.04
3.00
9.0 8.5 8.0 7.5 7.0 6.5 6.0 5.5 5.0 4.5 4.0 3.5 3.0 2.5 2.0 1.5 1.0 0.5 0.0
f1 (ppm)

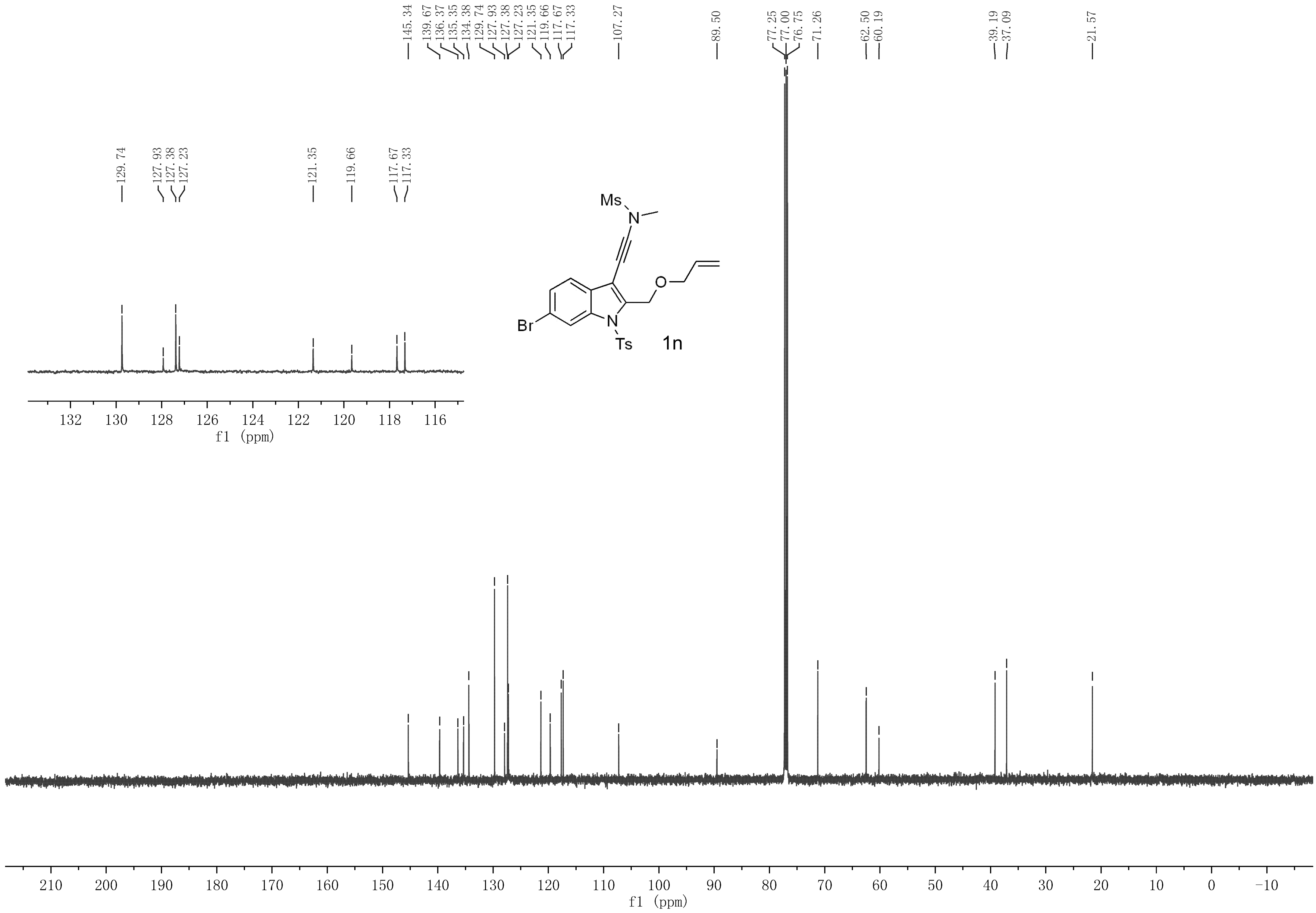
145.34
139.67
136.37
135.35
134.38
129.74
127.93
127.38
127.23
121.35
119.66
117.67
117.33
107.27
89.50
77.25
77.00
76.75
71.26
62.50
60.19
39.19
37.09
21.57
Ms
N
O
Br
N
Ts
1n
132 130 128 126 124 122 120 118 116
f1 (ppm)
210 200 190 180 170 160 150 140 130 120 110 100 90 80 70 60 50 40 30 20 10 0 −10
f1 (ppm)

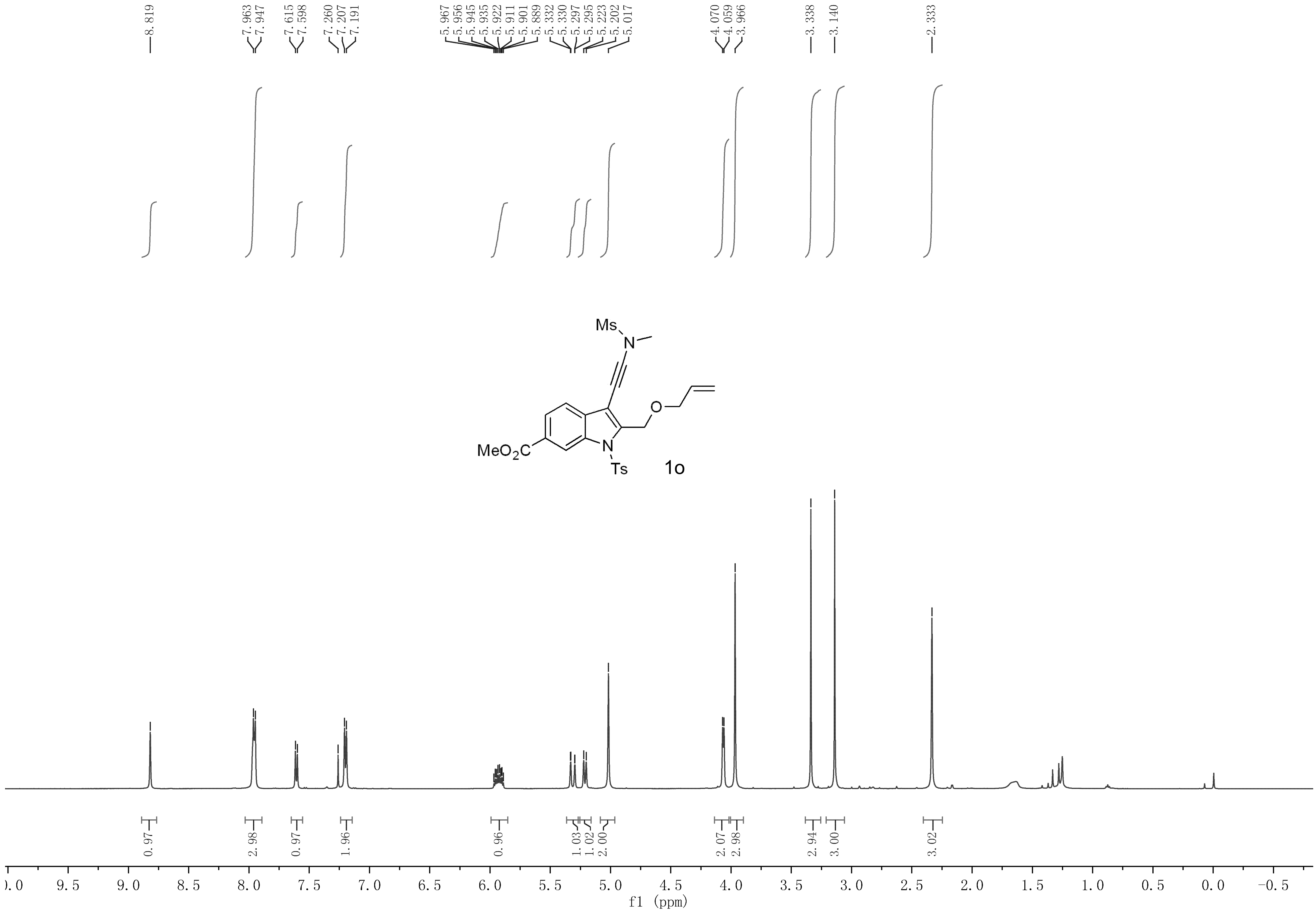

8.819
7.963
7.947
7.615
7.598
7.260
7.207
7.191
5.967
5.956
5.945
5.935
5.922
5.911
5.901
5.889
5.332
5.330
5.297
5.295
5.223
5.202
5.017
4.070
4.059
3.966
3.338
3.140
2.333
Ms
N
MeO2C
N
Ts
O
1o
0.97
2.98
0.97
1.96
0.96
1.03
1.02
2.00
2.07
2.98
2.94
3.00
3.02
9.5
9.0
8.5
8.0
7.5
7.0
6.5
6.0
5.5
5.0
4.5
4.0
3.5
3.0
2.5
2.0
1.5
1.0
0.5
0.0
−0.5
f1 (ppm)

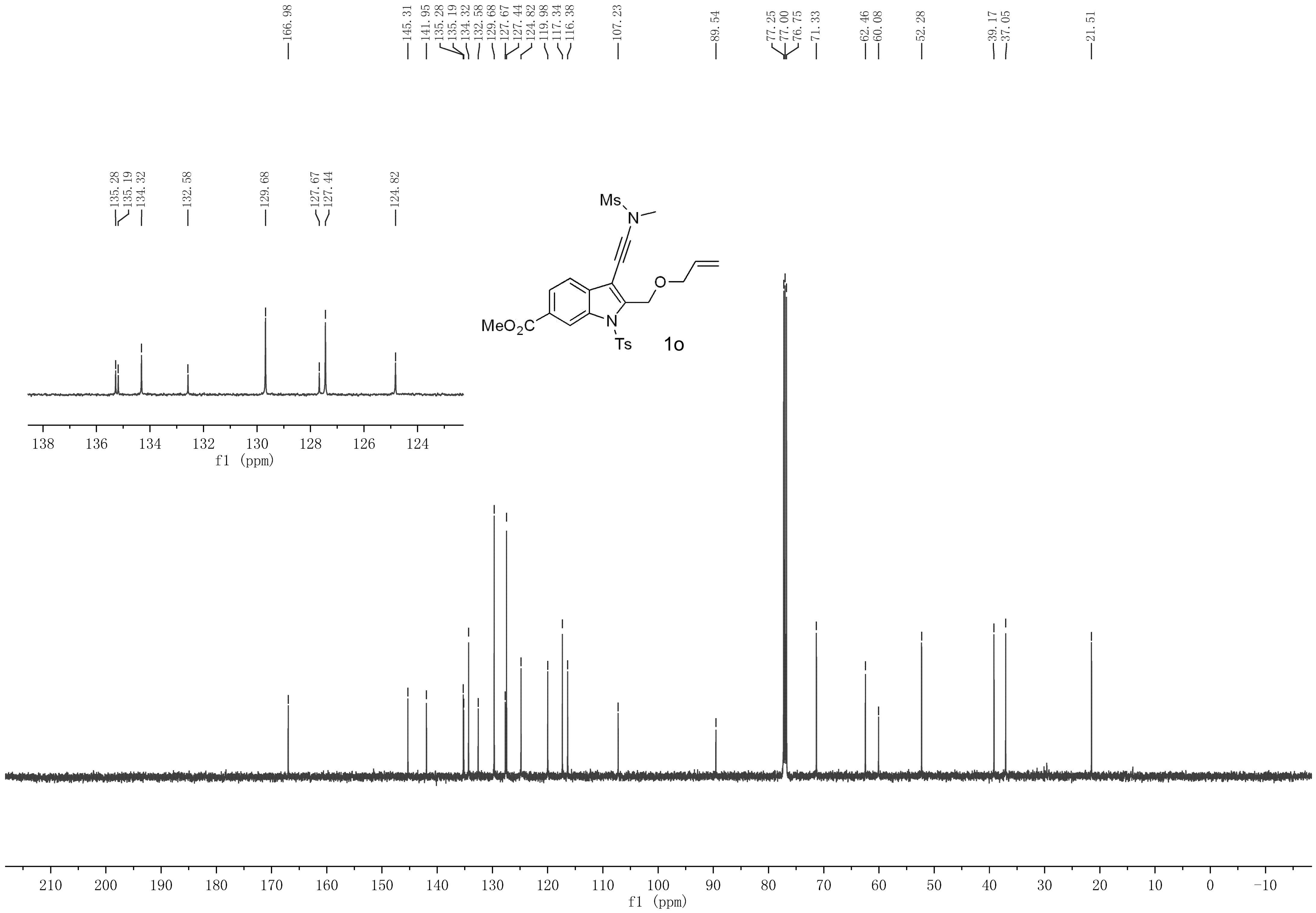
166.98
145.31
141.95
135.28
135.19
134.32
132.58
129.68
127.67
127.44
124.82
119.98
117.34
116.38
107.23
89.54
77.25
77.00
76.75
71.33
62.46
60.08
52.28
39.17
37.05
21.51
Ms
N
MeO2C
N
Ts
O
1o
138 136 134 132 130 128 126 124
f1 (ppm)
210 200 190 180 170 160 150 140 130 120 110 100 90 80 70 60 50 40 30 20 10 0 -10
f1 (ppm)

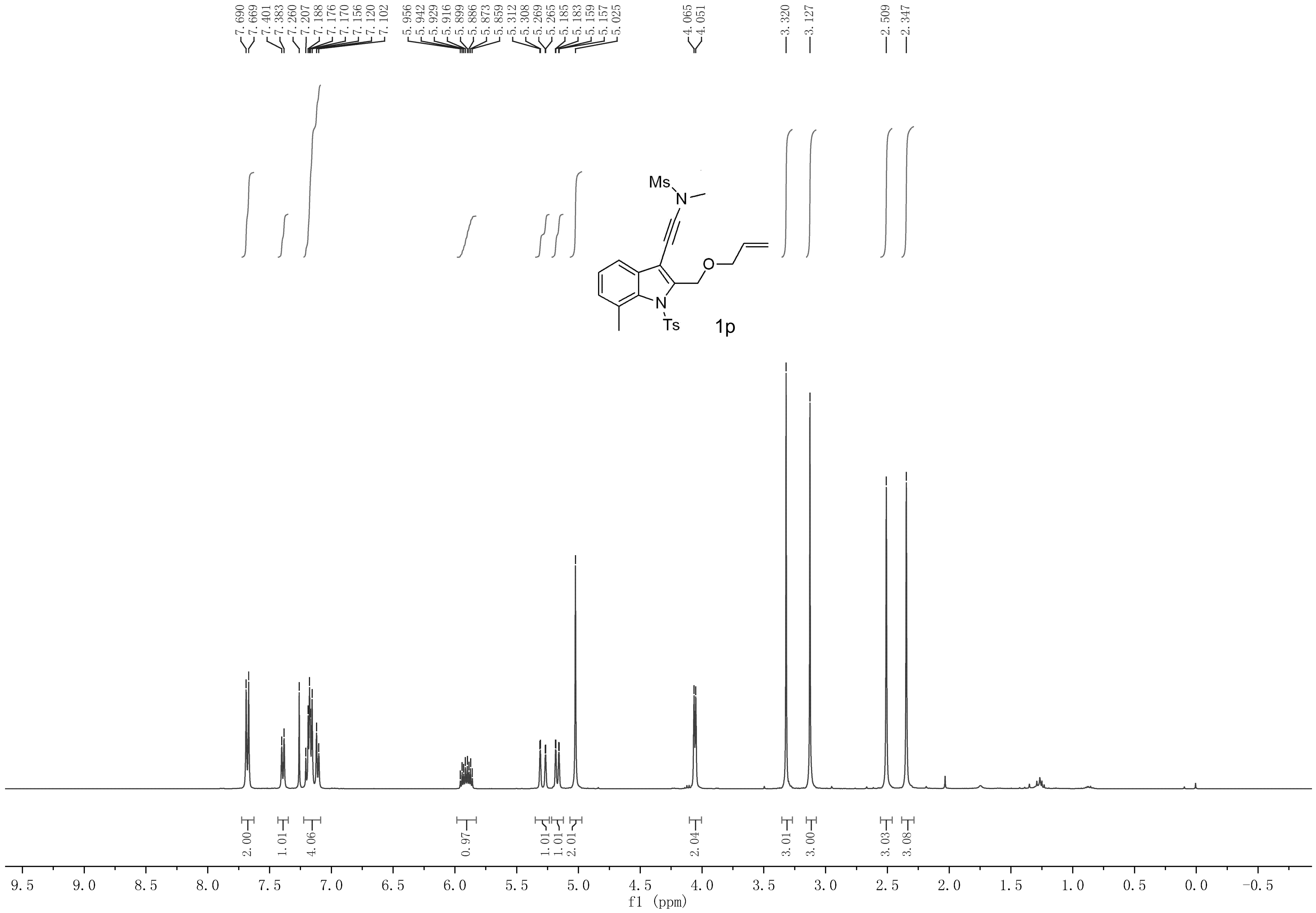
7.690
7.669
7.401
7.383
7.260
7.207
7.188
7.176
7.170
7.156
7.120
7.102
5.956
5.942
5.929
5.916
5.899
5.886
5.873
5.859
5.312
5.308
5.269
5.265
5.185
5.183
5.159
5.157
5.025
4.065
4.051
3.320
3.127
2.509
2.347
Ms
N
O
N
Ts
1p
2.00
1.01
4.06
0.97
1.01
1.01
2.01
2.04
3.01
3.00
3.03
3.08
9.5 9.0 8.5 8.0 7.5 7.0 6.5 6.0 5.5 5.0 4.5 4.0 3.5 3.0 2.5 2.0 1.5 1.0 0.5 0.0 -0.5
f1 (ppm)

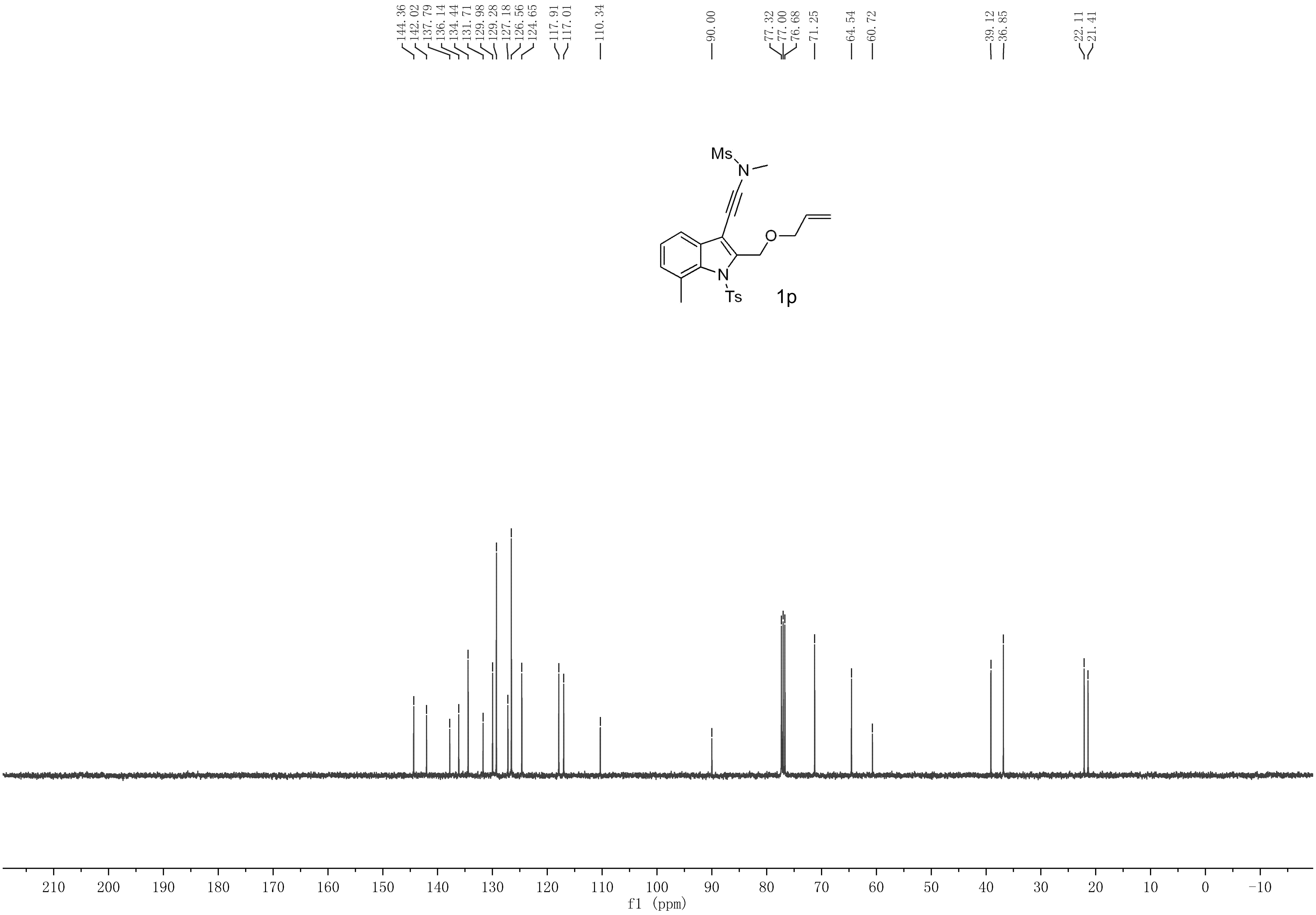
144.36
142.02
137.79
136.14
134.44
131.71
129.98
129.28
127.18
126.56
124.65
117.91
117.01
110.34
90.00
77.32
77.00
76.68
71.25
64.54
60.72
39.12
36.85
22.11
21.41
Ms
N
O
N
Ts
1p
210 200 190 180 170 160 150 140 130 120 110 100 90 80 70 60 50 40 30 20 10 0 −10
f1 (ppm)

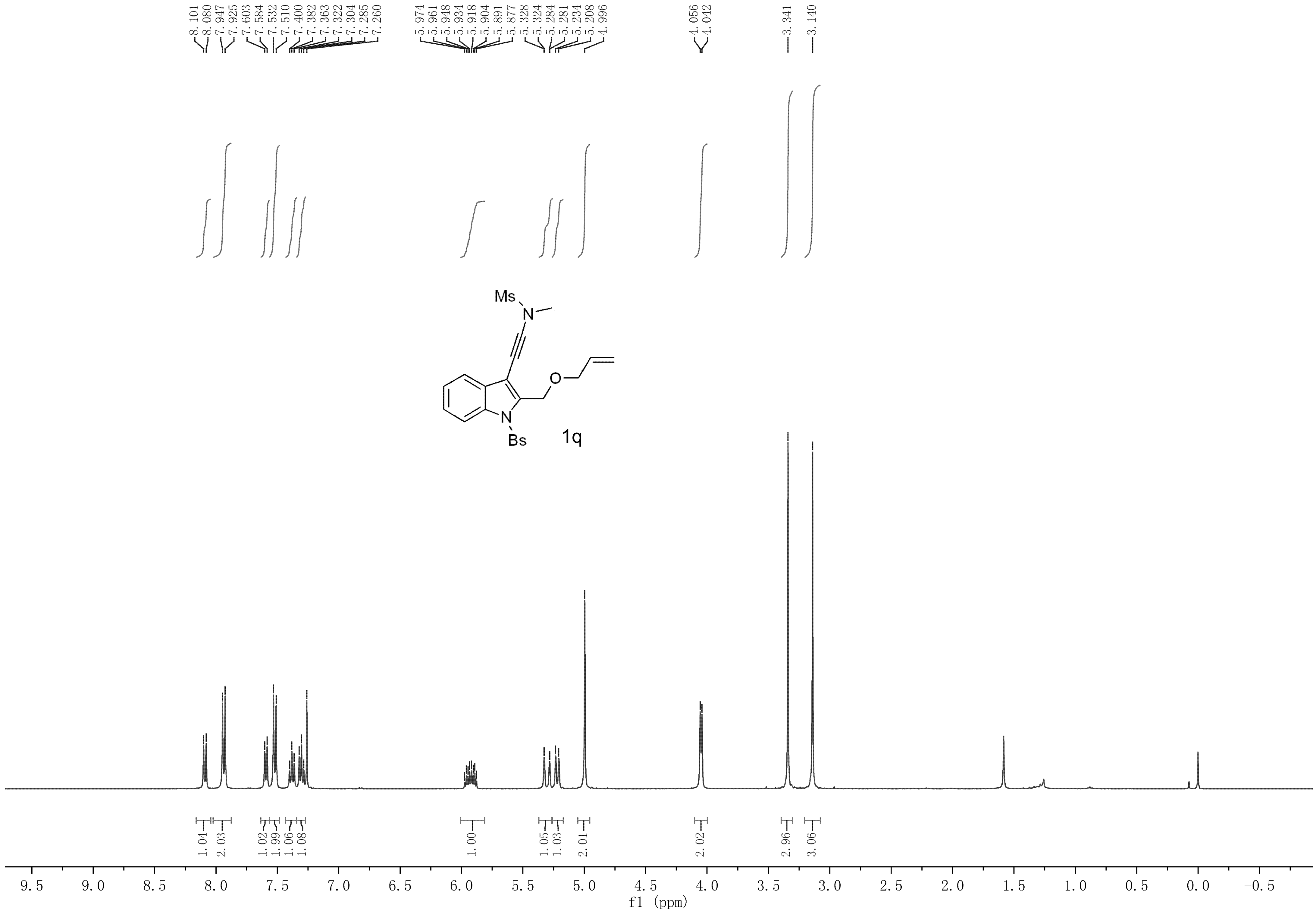

8.101
8.080
7.947
7.925
7.603
7.584
7.532
7.510
7.400
7.382
7.363
7.322
7.304
7.285
7.260
5.974
5.961
5.948
5.934
5.918
5.904
5.891
5.877
5.328
5.324
5.284
5.281
5.234
5.208
4.996
4.056
4.042
3.341
3.140
Ms
N
O
N
Bs
1q
1.04
2.03
1.02
1.99
1.06
1.08
1.00
1.05
1.03
2.01
2.02
2.96
3.06
9.5
9.0
8.5
8.0
7.5
7.0
6.5
6.0
5.5
5.0
4.5
4.0
3.5
3.0
2.5
2.0
1.5
1.0
0.5
0.0
-0.5
f1 (ppm)

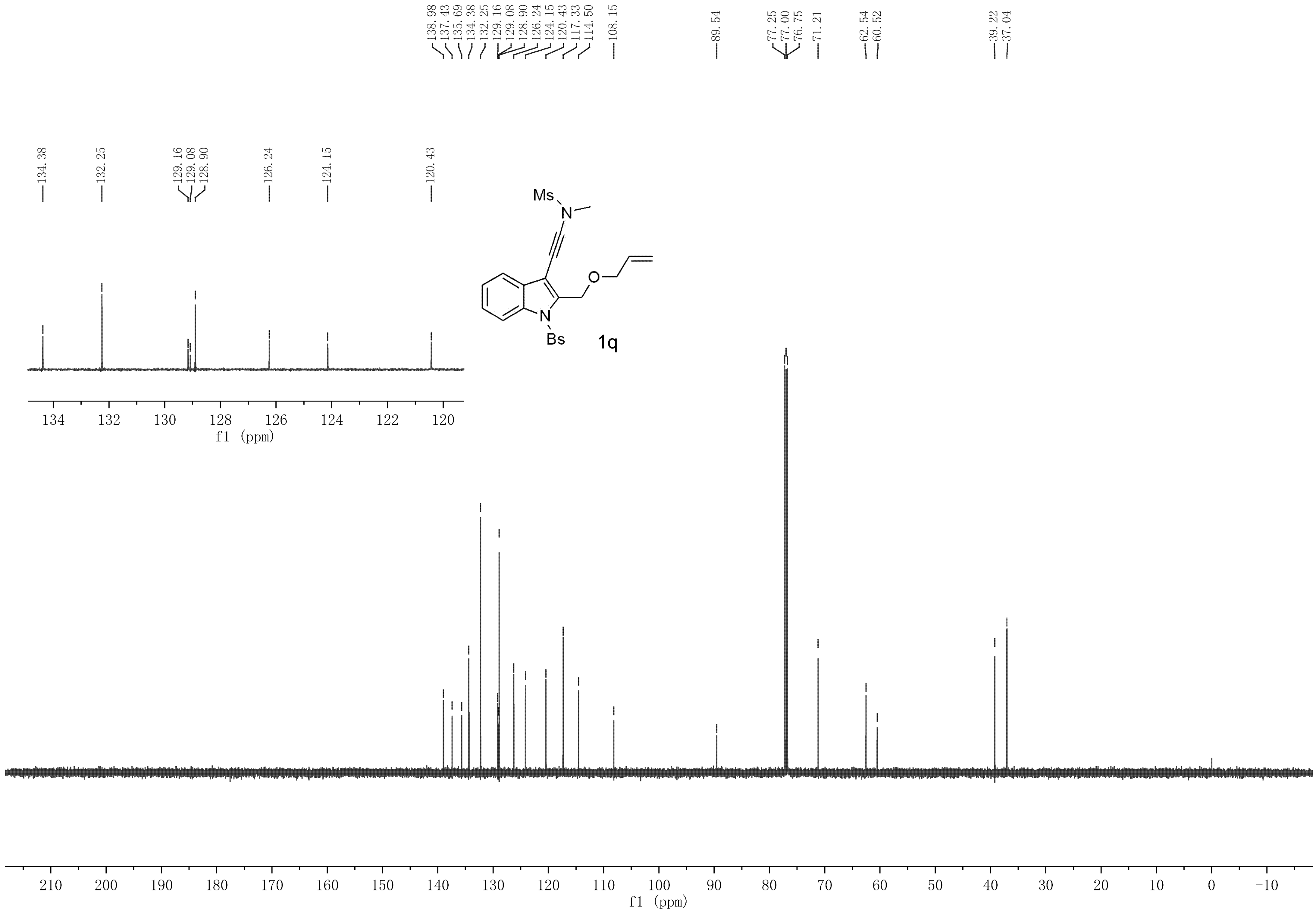
138.98
137.43
135.69
134.38
132.25
129.16
129.08
128.90
126.24
124.15
120.43
117.33
114.50
108.15
89.54
77.25
77.00
76.75
71.21
62.54
60.52
39.22
37.04
134.38
132.25
129.16
129.08
128.90
126.24
124.15
120.43
Ms
N
O
N
Bs
1q
134 132 130 128 126 124 122 120
f1 (ppm)
210 200 190 180 170 160 150 140 130 120 110 100 90 80 70 60 50 40 30 20 10 0 −10
f1 (ppm)

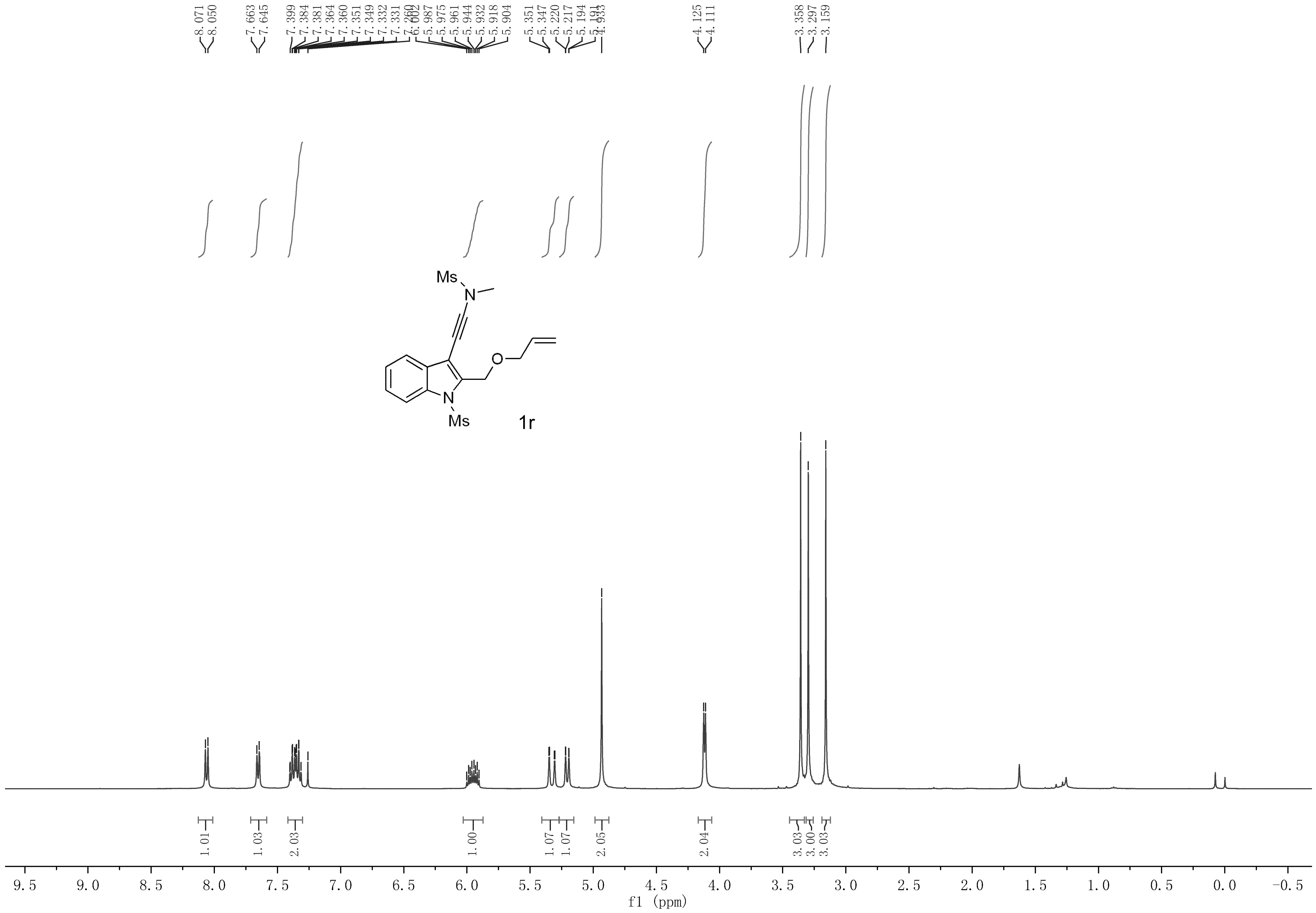
Ms
N
O
N
Ms
1r
f1 (ppm)

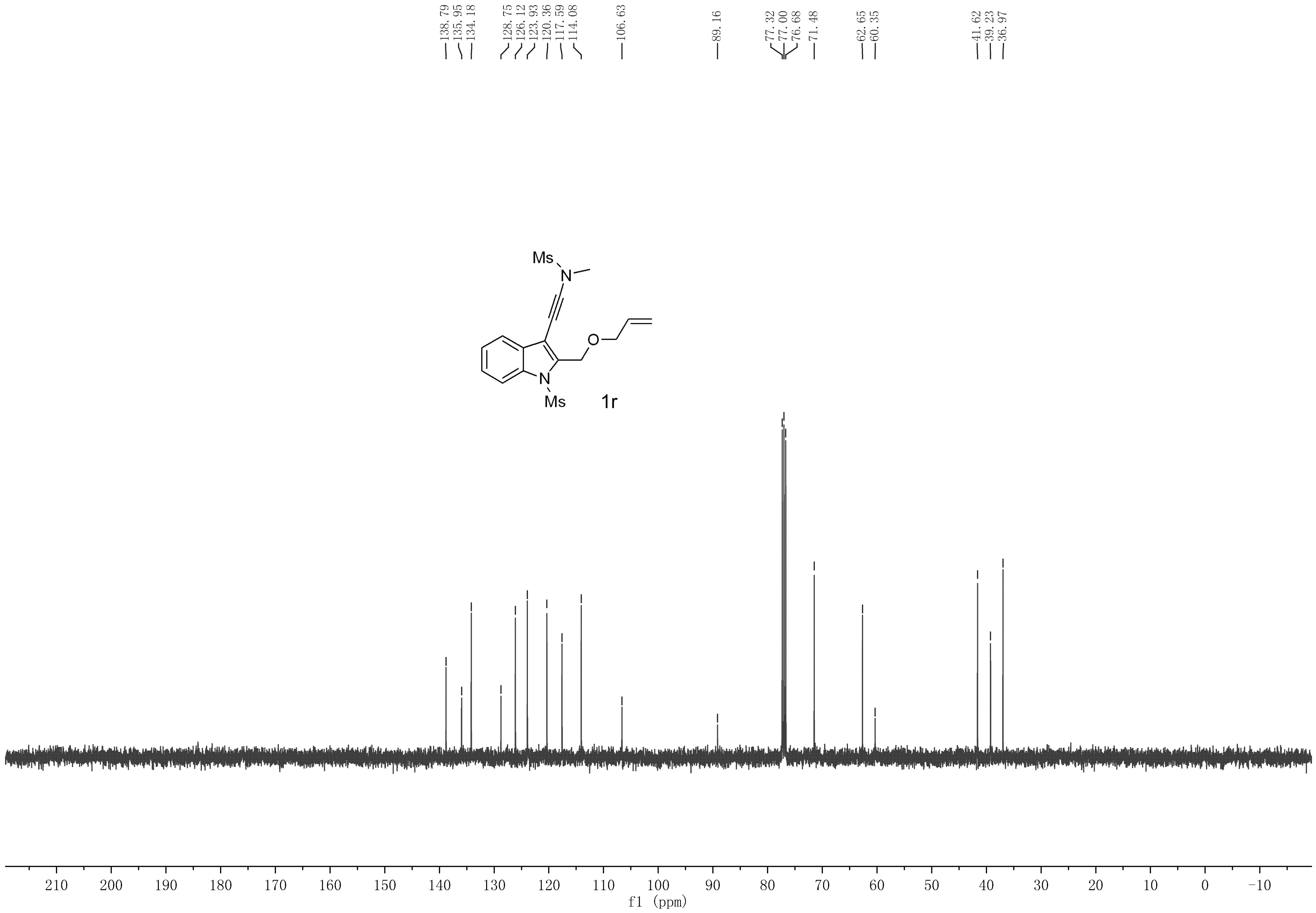
138.79
135.95
134.18
128.75
126.12
123.93
120.36
117.59
114.08
106.63
89.16
77.32
77.00
76.68
71.48
62.65
60.35
41.62
39.23
36.97
Ms
N
O
N
Ms
1r
210 200 190 180 170 160 150 140 130 120 110 100 90 80 70 60 50 40 30 20 10 0 −10
f1 (ppm)

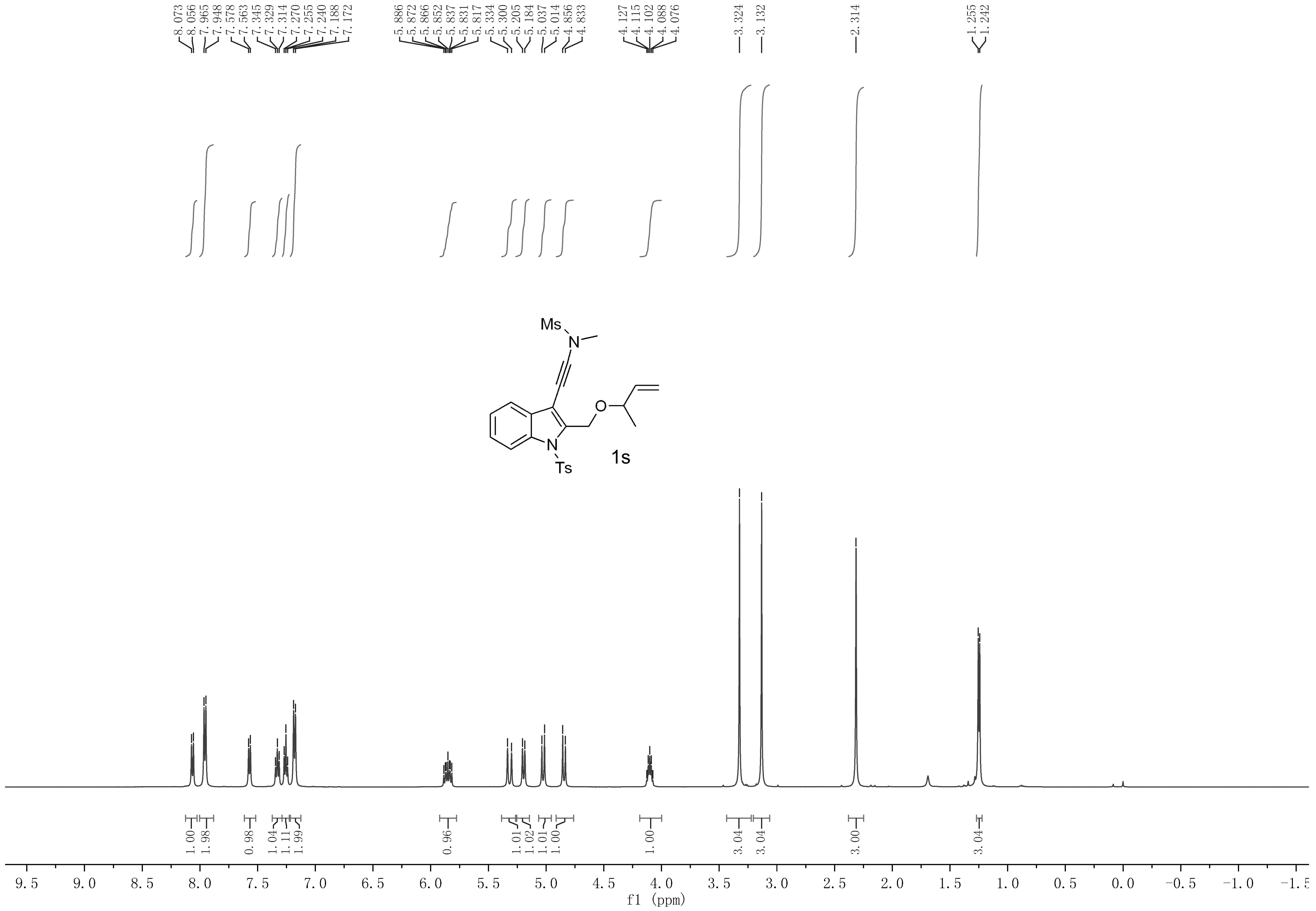
8.073
8.056
7.965
7.948
7.578
7.563
7.345
7.329
7.314
7.270
7.255
7.240
7.188
7.172
5.886
5.872
5.866
5.852
5.837
5.831
5.817
5.334
5.300
5.205
5.184
5.037
5.014
4.856
4.833
4.127
4.115
4.102
4.088
4.076
3.324
3.132
2.314
1.255
1.242
Ms
N
O
N
Ts
1s
1.00
1.98
0.98
1.04
1.11
1.99
0.96
1.01
1.02
1.01
1.00
1.00
3.04
3.04
3.00
3.04
9.5
9.0
8.5
8.0
7.5
7.0
6.5
6.0
5.5
5.0
4.5
4.0
3.5
3.0
2.5
2.0
1.5
1.0
0.5
0.0
−0.5
−1.0
f1 (ppm)

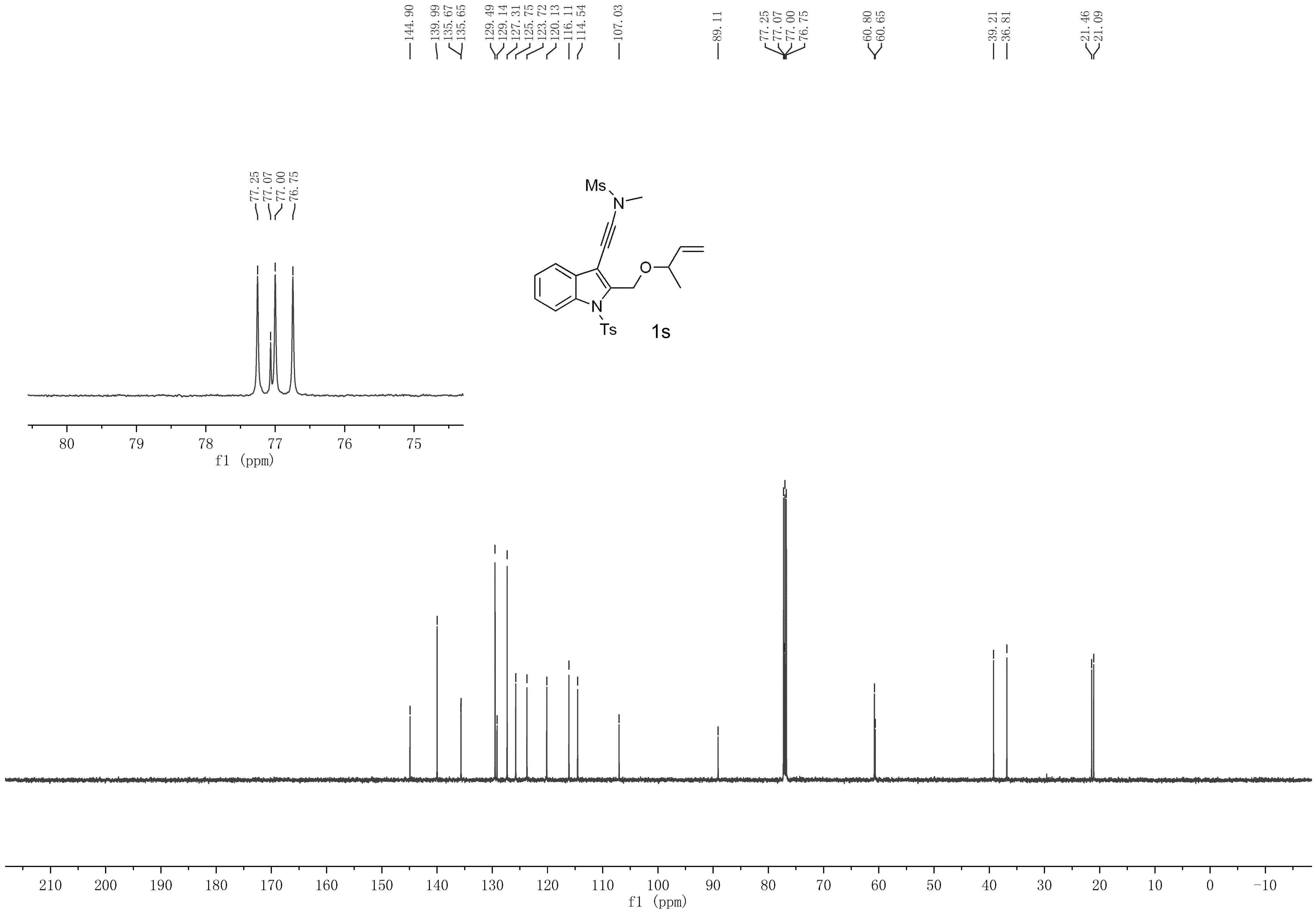
144.90
139.99
135.67
135.65
129.49
129.14
127.31
125.75
123.72
120.13
116.11
114.54
107.03
89.11
77.25
77.07
77.00
76.75
60.80
60.65
39.21
36.81
21.46
21.09
77.25
77.07
77.00
76.75
80 79 78 77 76 75
f1 (ppm)
Ms
N
O
N
Ts
1s
210 200 190 180 170 160 150 140 130 120 110 100 90 80 70 60 50 40 30 20 10 0 −10
f1 (ppm)

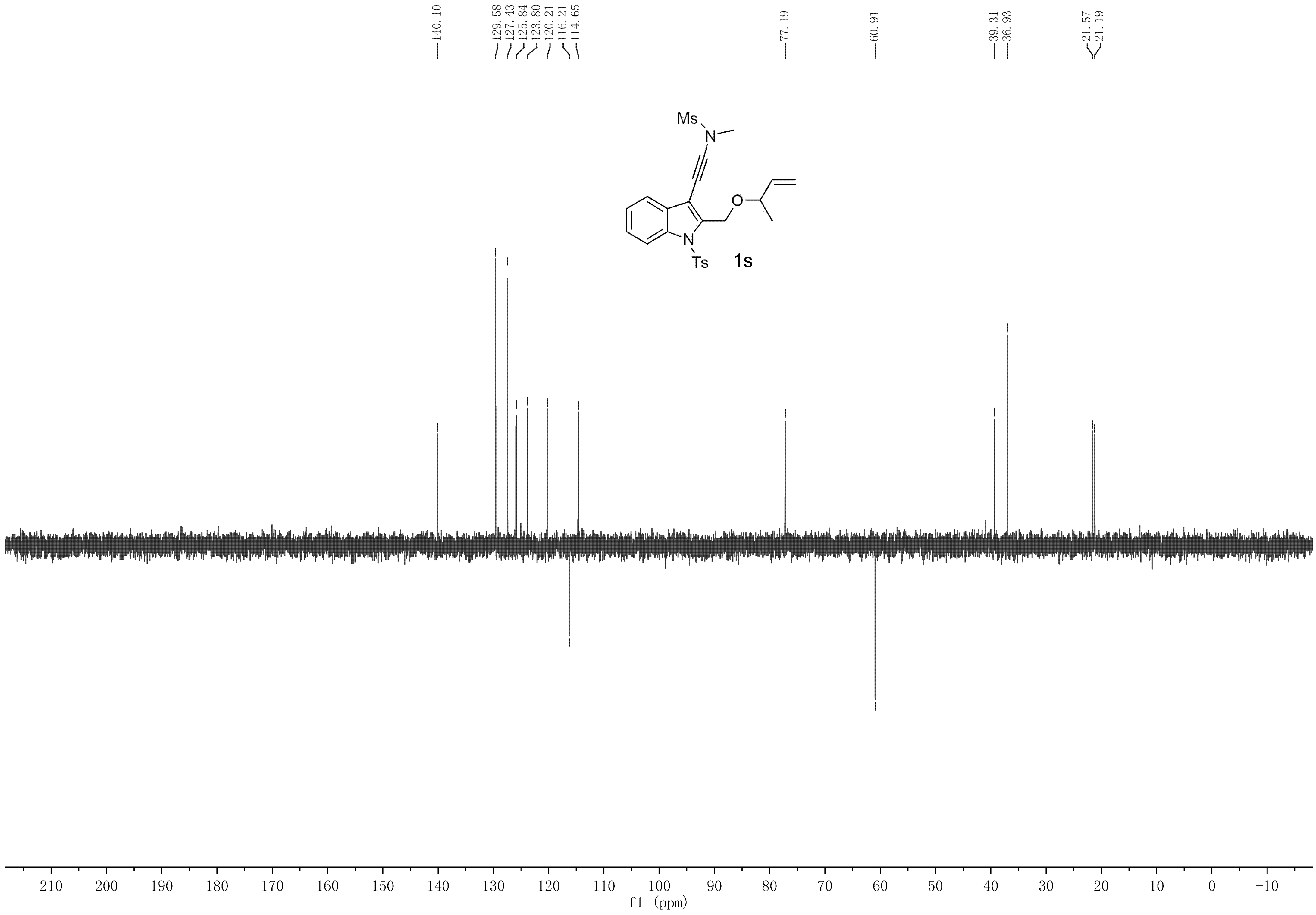
140.10
129.58
127.43
125.84
123.80
120.21
116.21
114.65
77.19
60.91
39.31
36.93
21.57
21.19
Ms
N
O
N
Ts
1s
210 200 190 180 170 160 150 140 130 120 110 100 90 80 70 60 50 40 30 20 10 0 -10
f1 (ppm)

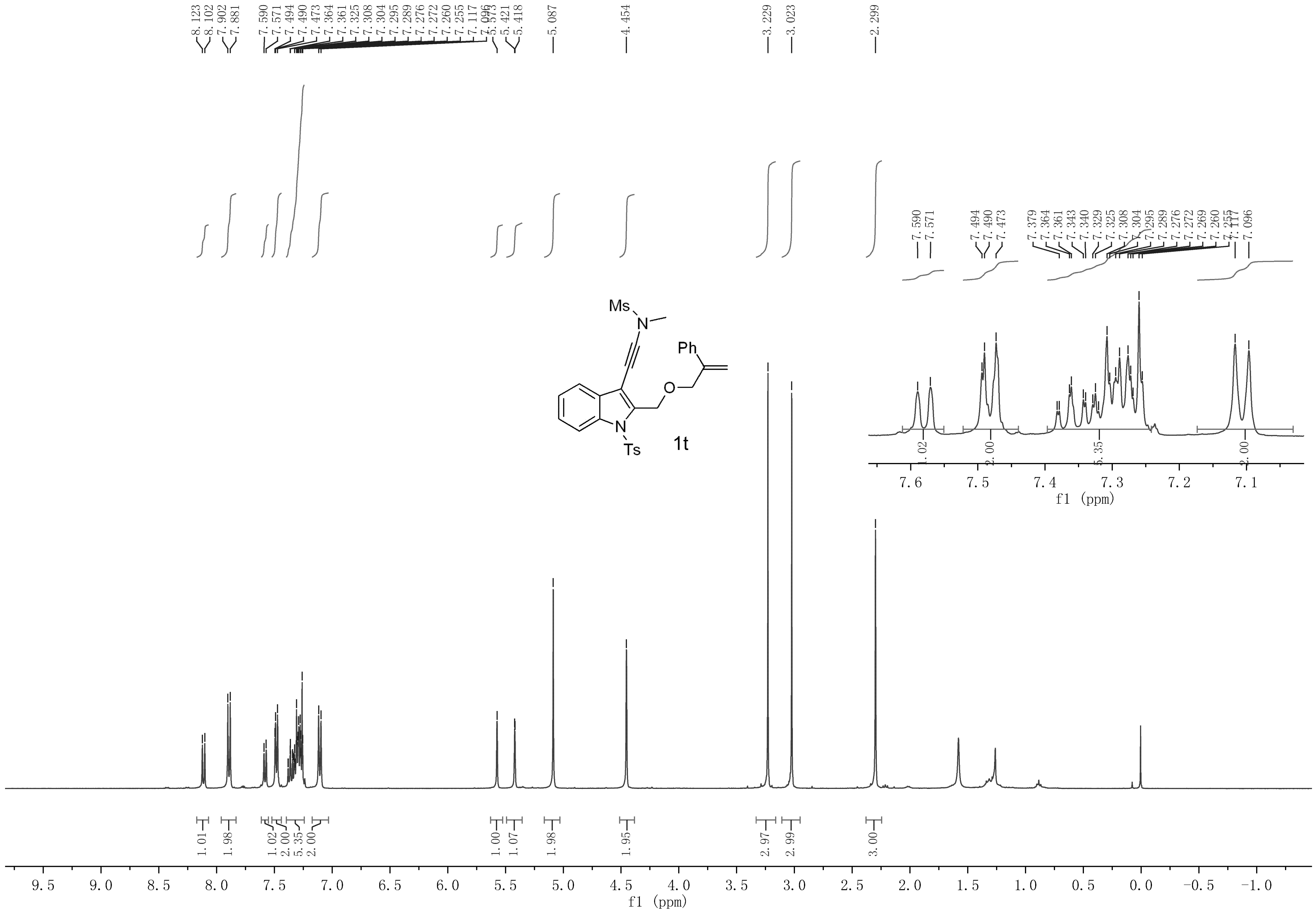
8.123
8.102
7.902
7.881
7.590
7.571
7.494
7.490
7.473
7.364
7.361
7.325
7.308
7.304
7.295
7.289
7.276
7.272
7.260
7.255
7.117
7.096
5.573
5.421
5.418
5.087
4.454
3.229
3.023
2.299
Ms
N
Ph
O
N
Ts
1t
7.590
7.571
7.494
7.490
7.473
7.379
7.364
7.361
7.343
7.340
7.329
7.325
7.308
7.304
7.295
7.289
7.276
7.272
7.269
7.260
7.255
7.117
7.096
1.02
2.00
5.35
2.00
7.6 7.5 7.4 7.3 7.2 7.1
f1 (ppm)
1.01
1.98
1.02
2.00
5.35
2.00
1.00
1.07
1.98
1.95
2.97
2.99
3.00
9.5 9.0 8.5 8.0 7.5 7.0 6.5 6.0 5.5 5.0 4.5 4.0 3.5 3.0 2.5 2.0 1.5 1.0 0.5 0.0 -0.5 -1.0
f1 (ppm)

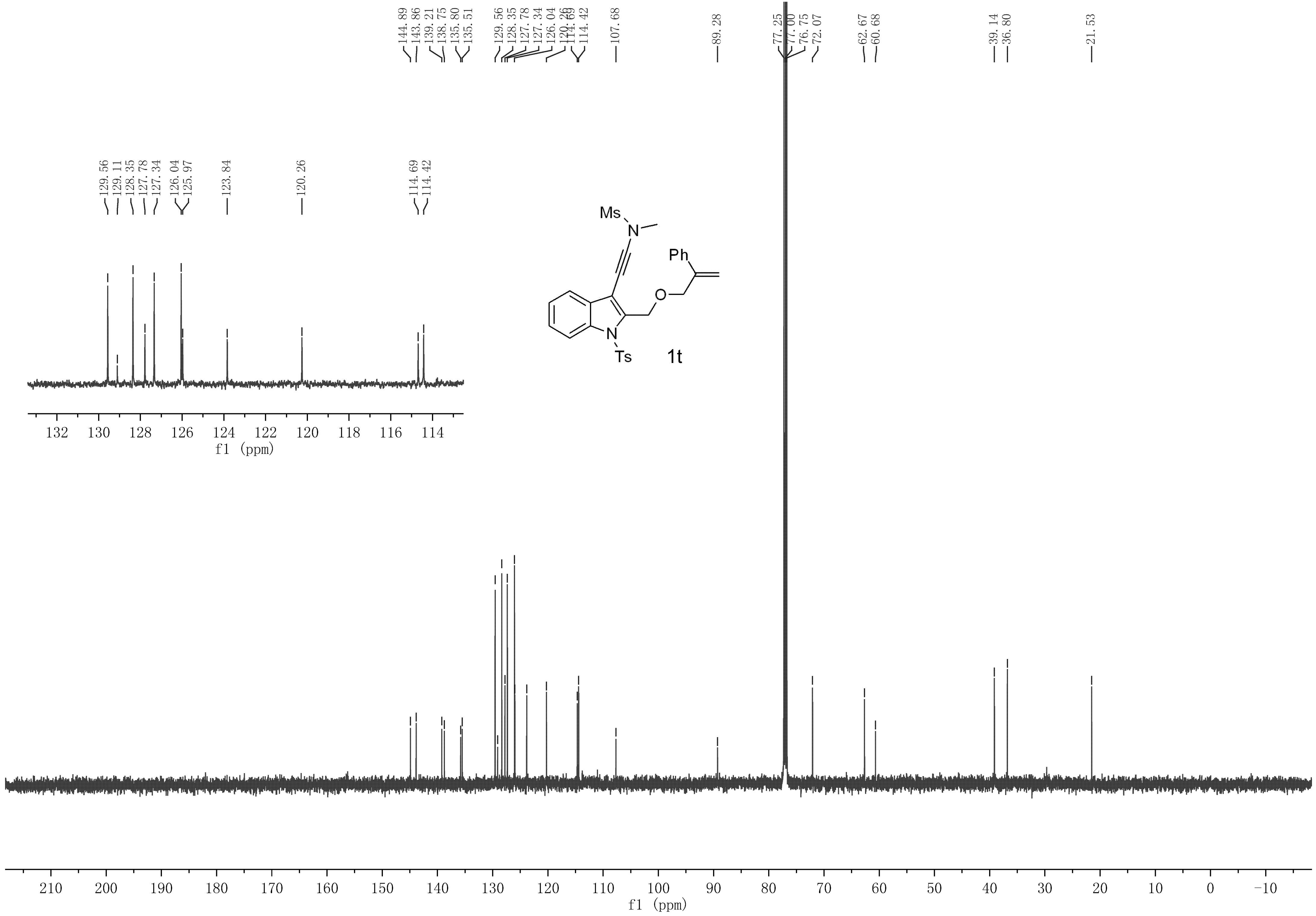

144.89
143.86
139.21
138.75
135.80
135.51
129.56
128.35
127.78
127.34
126.04
120.26
114.69
114.42
107.68
89.28
77.25
77.00
76.75
72.07
62.67
60.68
39.14
36.80
21.53
129.56
129.11
128.35
127.78
127.34
126.04
125.97
123.84
120.26
114.69
114.42
Ms
N
Ph
O
N
Ts
1t
f1 (ppm)
f1 (ppm)

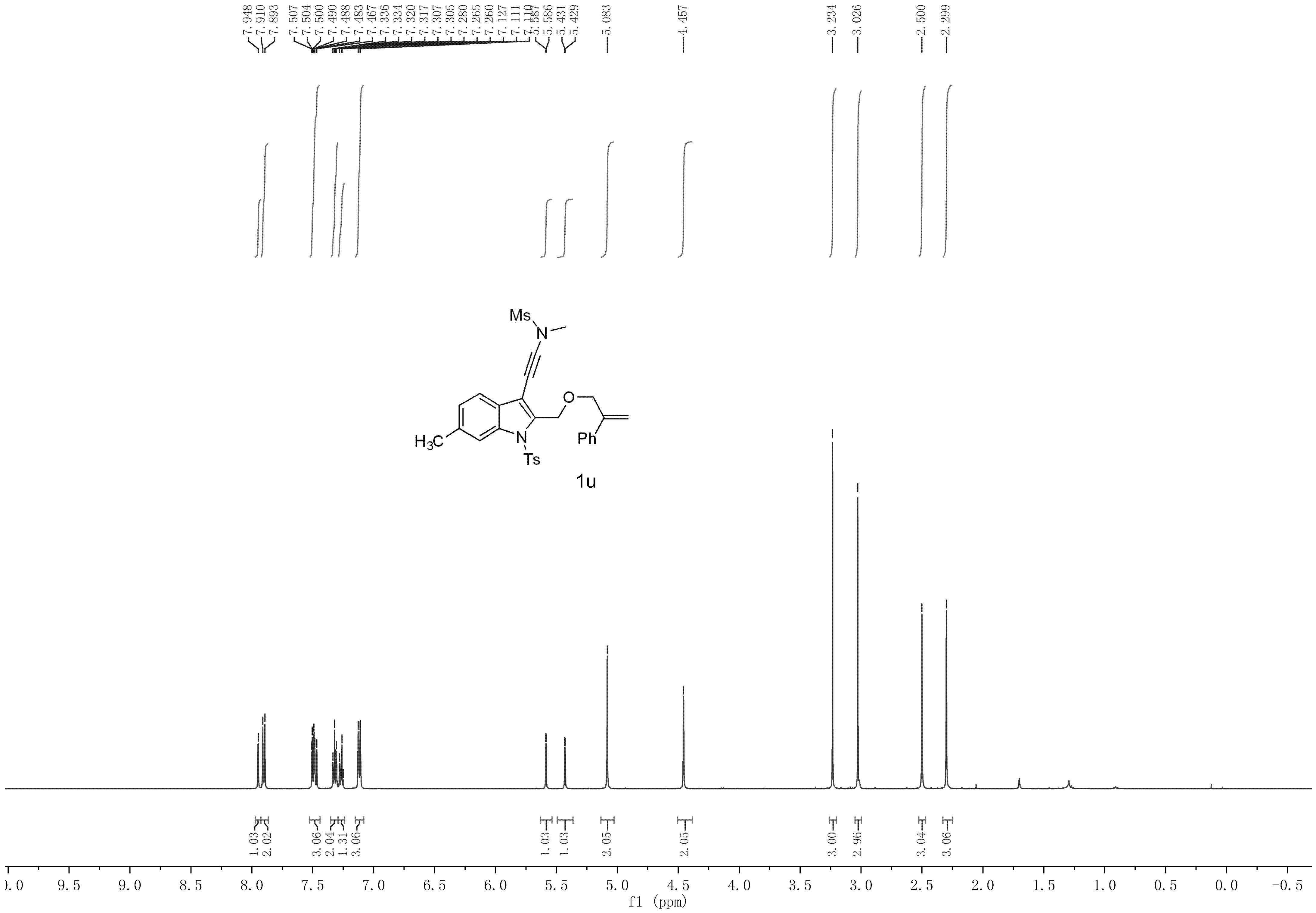
7.948
7.910
7.893
7.507
7.504
7.500
7.490
7.488
7.483
7.467
7.336
7.334
7.320
7.317
7.307
7.305
7.280
7.265
7.260
7.127
7.111
7.110
5.587
5.586
5.431
5.429
5.083
4.457
3.234
3.026
2.500
2.299
Ms
N
H3C
N
Ts
O
Ph
1u
1.03
2.02
3.06
2.04
1.31
3.06
1.03
1.03
2.05
2.05
3.00
2.96
3.04
3.06
f1 (ppm)

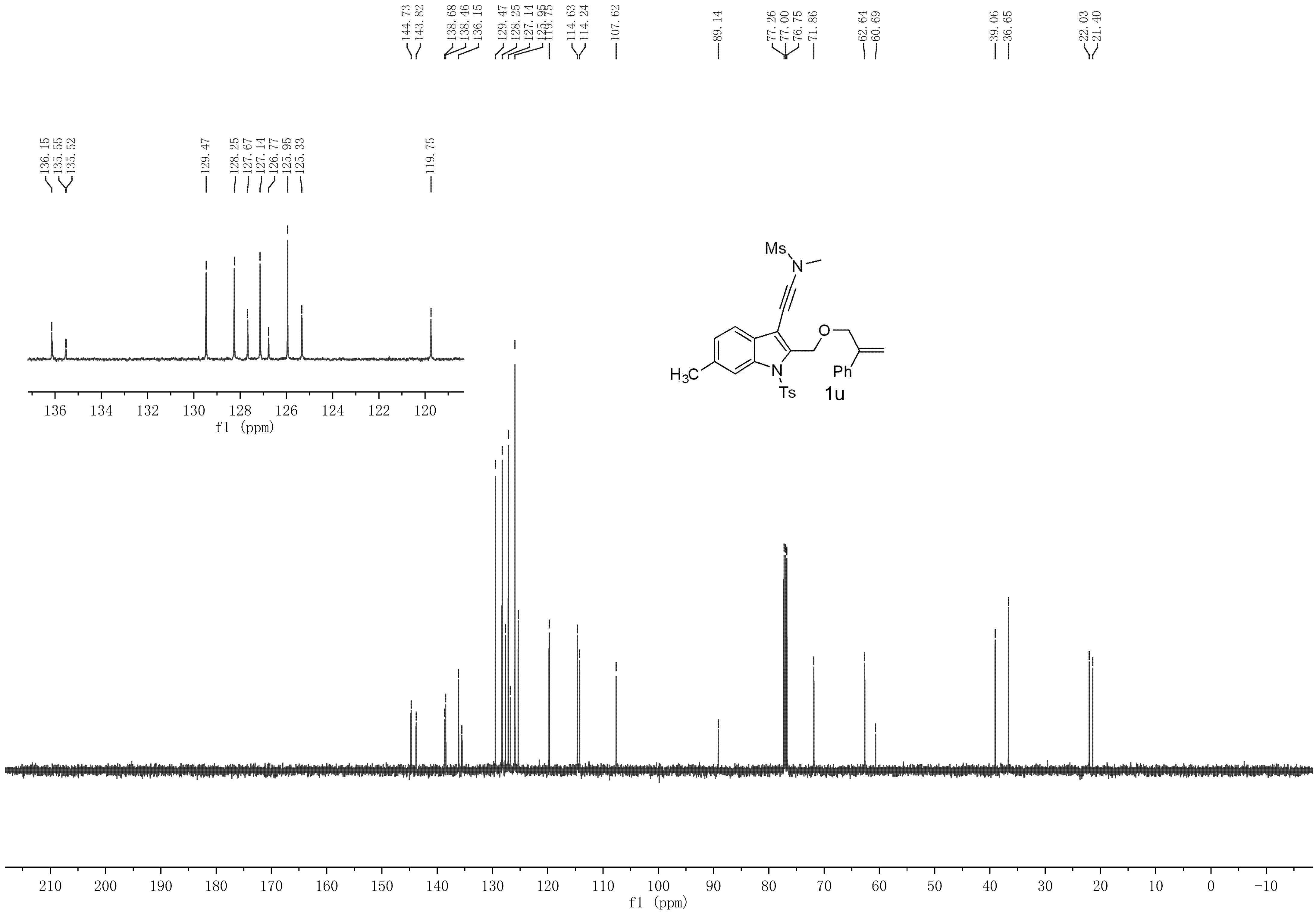
144.73
143.82
138.68
138.46
136.15
129.47
128.25
127.14
125.95
119.75
114.63
114.24
107.62
89.14
77.26
77.00
76.75
71.86
62.64
60.69
39.06
36.65
22.03
21.40
136.15
135.55
135.52
129.47
128.25
127.67
127.14
126.77
125.95
125.33
119.75
136 134 132 130 128 126 124 122 120
f1 (ppm)
Ms
N
O
Ph
H3C
N
Ts
1u
210 200 190 180 170 160 150 140 130 120 110 100 90 80 70 60 50 40 30 20 10 0 −10
f1 (ppm)

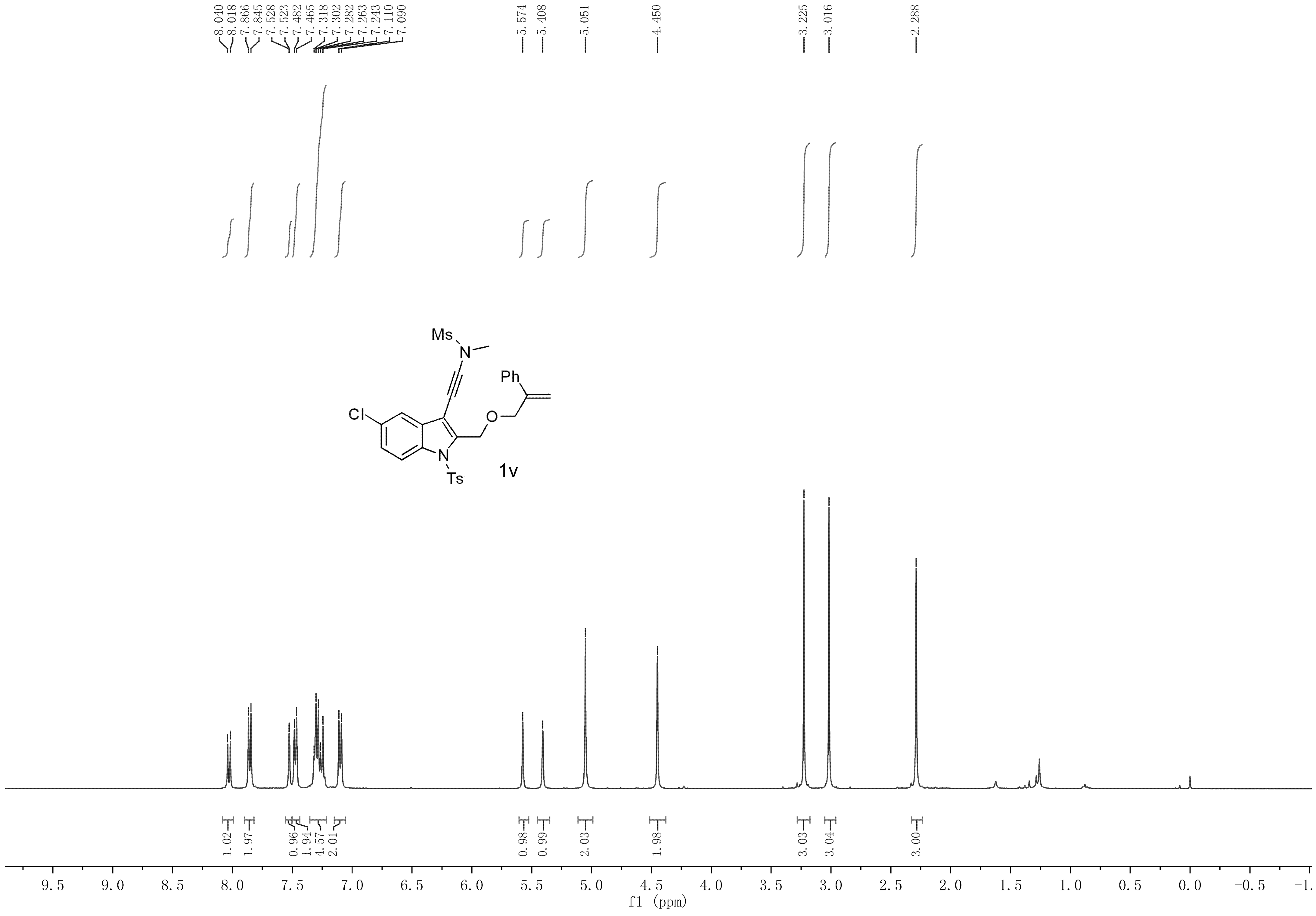
8.040
8.018
7.866
7.845
7.528
7.523
7.482
7.465
7.318
7.302
7.282
7.263
7.243
7.110
7.090
5.574
5.408
5.051
4.450
3.225
3.016
2.288
Ms
N
Ph
O
Cl
N
Ts
1v
1.02
1.97
0.96
1.94
4.57
2.01
0.98
0.99
2.03
1.98
3.03
3.04
3.00
9.5
9.0
8.5
8.0
7.5
7.0
6.5
6.0
5.5
5.0
4.5
4.0
3.5
3.0
2.5
2.0
1.5
1.0
0.5
0.0
-0.5
-1.
f1 (ppm)

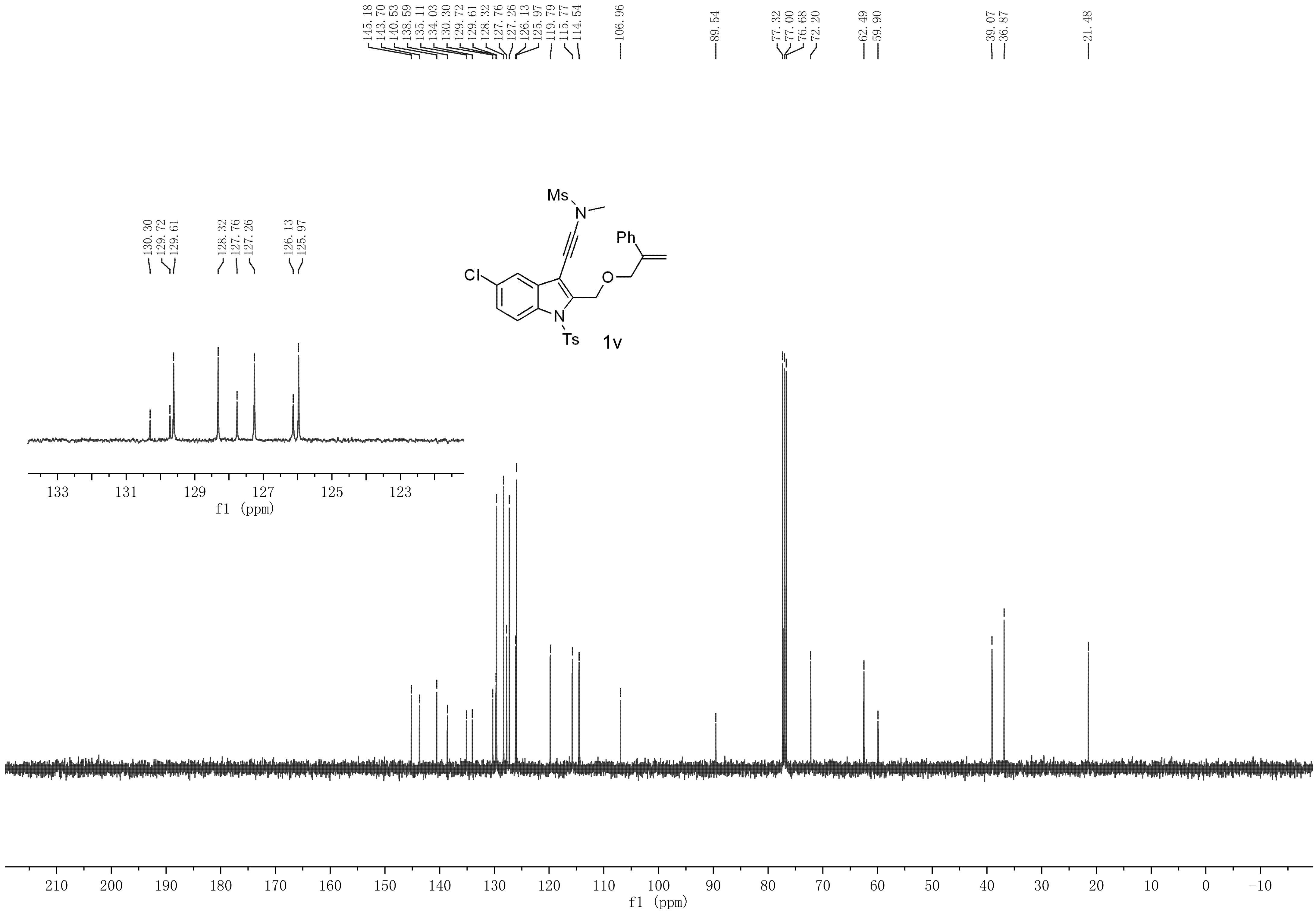
145.18
143.70
140.53
138.59
135.11
134.03
130.30
129.72
129.61
128.32
127.76
127.26
126.13
125.97
119.79
115.77
114.54
106.96
89.54
77.32
77.00
76.68
72.20
62.49
59.90
39.07
36.87
21.48
Ms
N
Ph
Cl
O
N
Ts
1v
f1 (ppm)
f1 (ppm)

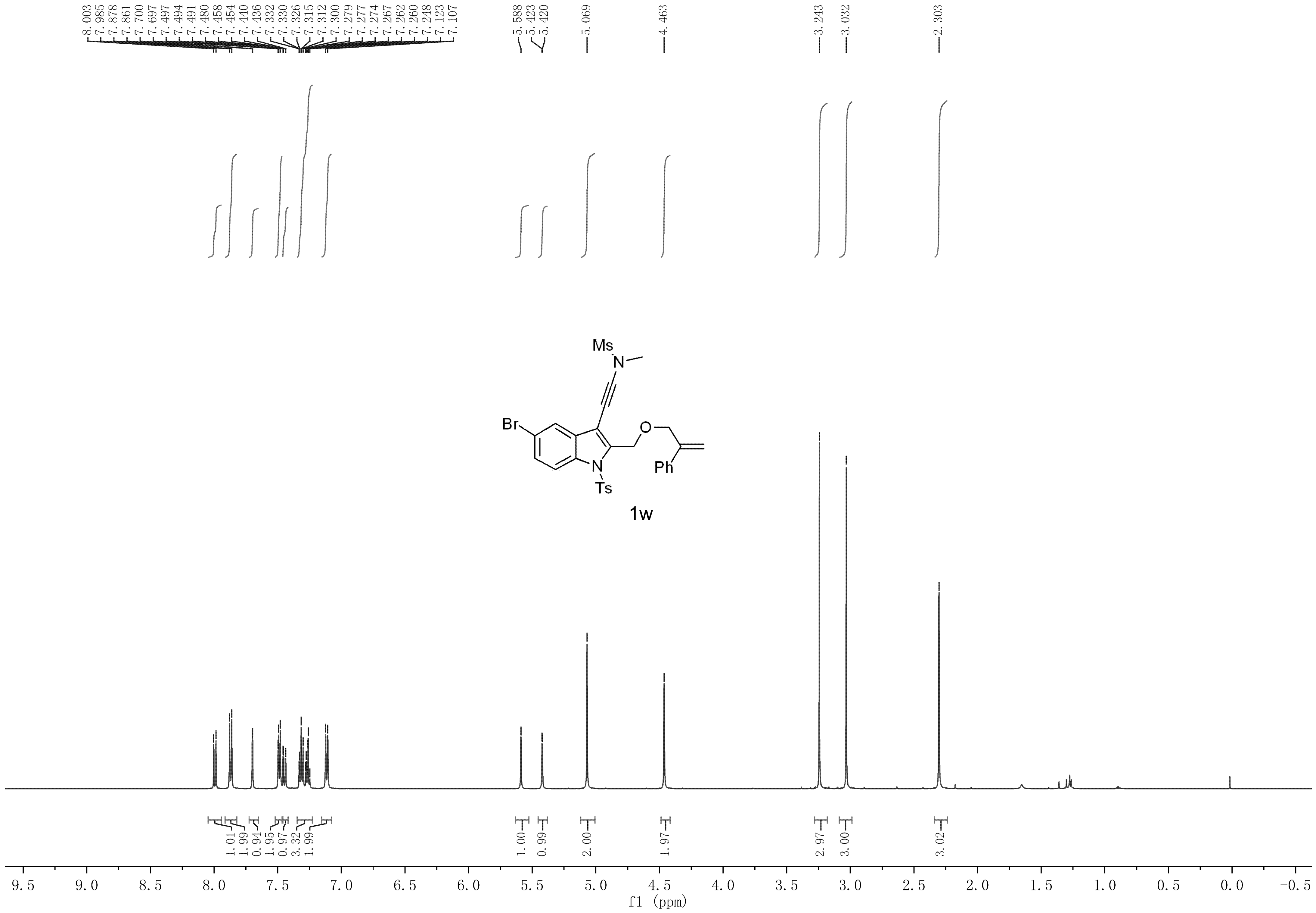

8.003 7.985 7.878 7.861 7.700 7.697 7.497 7.494 7.491 7.480 7.458 7.454 7.440 7.436 7.332 7.330 7.326 7.315 7.312 7.300 7.279 7.277 7.274 7.267 7.262 7.260 7.248 7.123 7.107
5.588 5.423 5.420
5.069
4.463
3.243
3.032
2.303
Ms
N
Br
O
N
Ts
Ph
1w
1.01 1.99 0.94 1.95 0.97 3.32 1.99
1.00 0.99 2.00 1.97 2.97 3.00 3.02
9.5 9.0 8.5 8.0 7.5 7.0 6.5 6.0 5.5 5.0 4.5 4.0 3.5 3.0 2.5 2.0 1.5 1.0 0.5 0.0 −0.5
f1 (ppm)

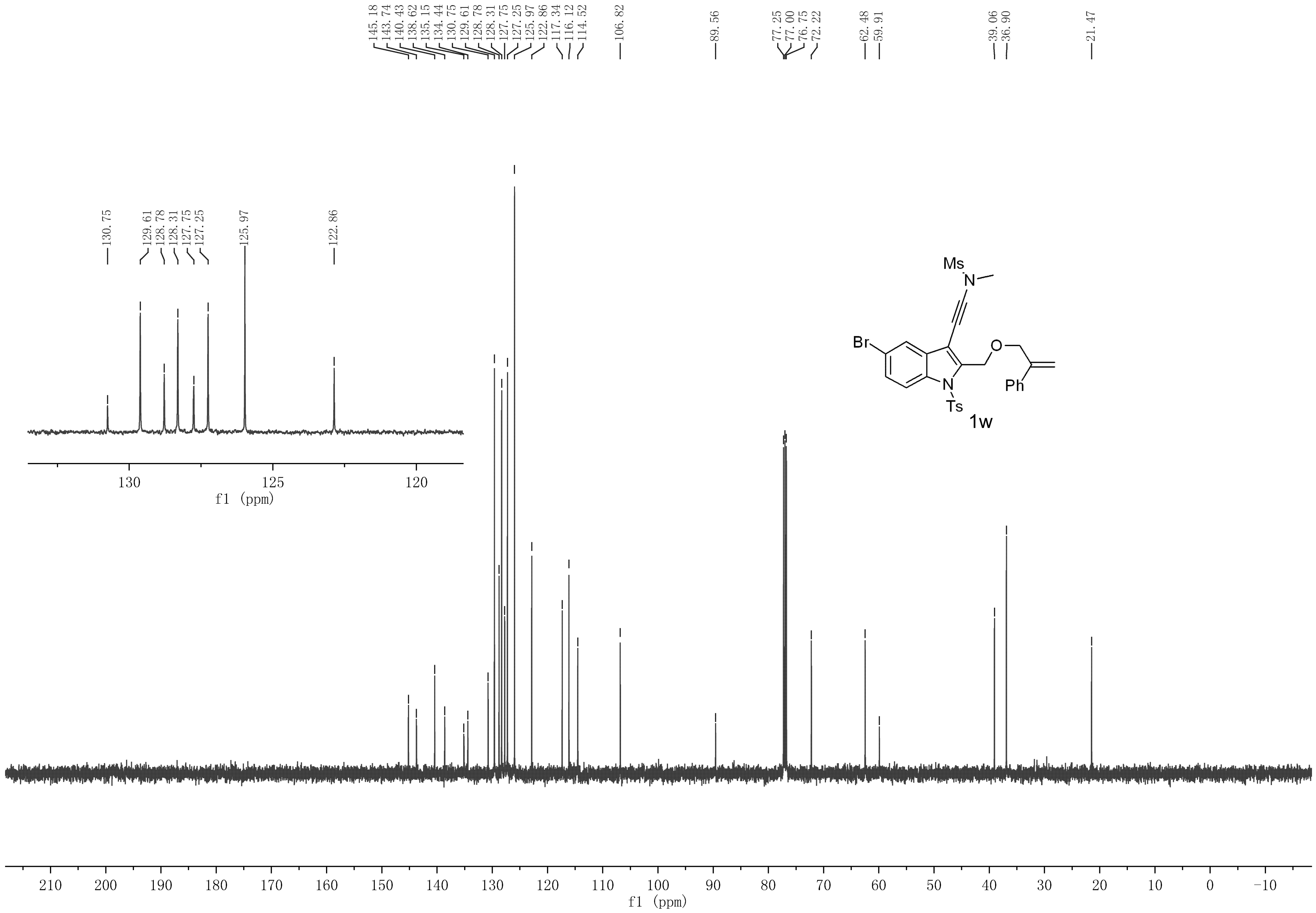
145.18
143.74
140.43
138.62
135.15
134.44
130.75
129.61
128.78
128.31
127.75
127.25
125.97
122.86
117.34
116.12
114.52
106.82
89.56
77.25
77.00
76.75
72.22
62.48
59.91
39.06
36.90
21.47
130.75
129.61
128.78
128.31
127.75
127.25
125.97
122.86
130
125
120
f1 (ppm)
Ms
N
Br
O
N
Ph
Ts
1w
210
200
190
180
170
160
150
140
130
120
110
100
90
80
70
60
50
40
30
20
10
0
-10
f1 (ppm)

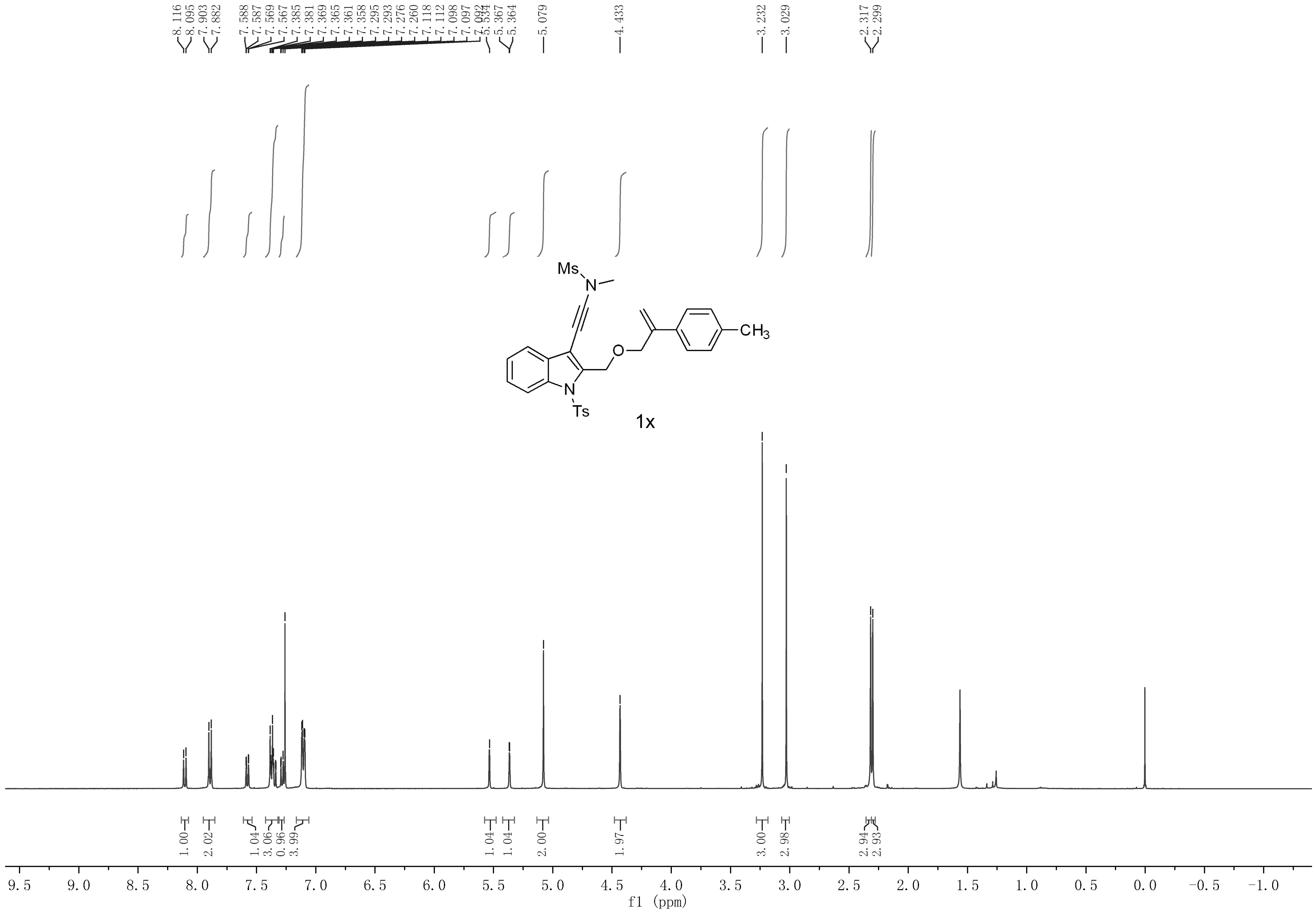
8.116
8.095
7.903
7.882
7.588
7.587
7.569
7.567
7.385
7.381
7.369
7.365
7.361
7.358
7.295
7.293
7.276
7.260
7.118
7.112
7.098
7.097
5.367
5.364
5.079
4.433
3.232
3.029
2.317
2.299
Ms
N
O
CH_3
N
Ts
1x
1.00
2.02
1.04
3.06
0.96
3.99
1.04
1.04
2.00
1.97
3.00
2.98
2.94
2.93
9.5
9.0
8.5
8.0
7.5
7.0
6.5
6.0
5.5
5.0
4.5
4.0
3.5
3.0
2.5
2.0
1.5
1.0
0.5
0.0
-0.5
-1.0
f1 (ppm)

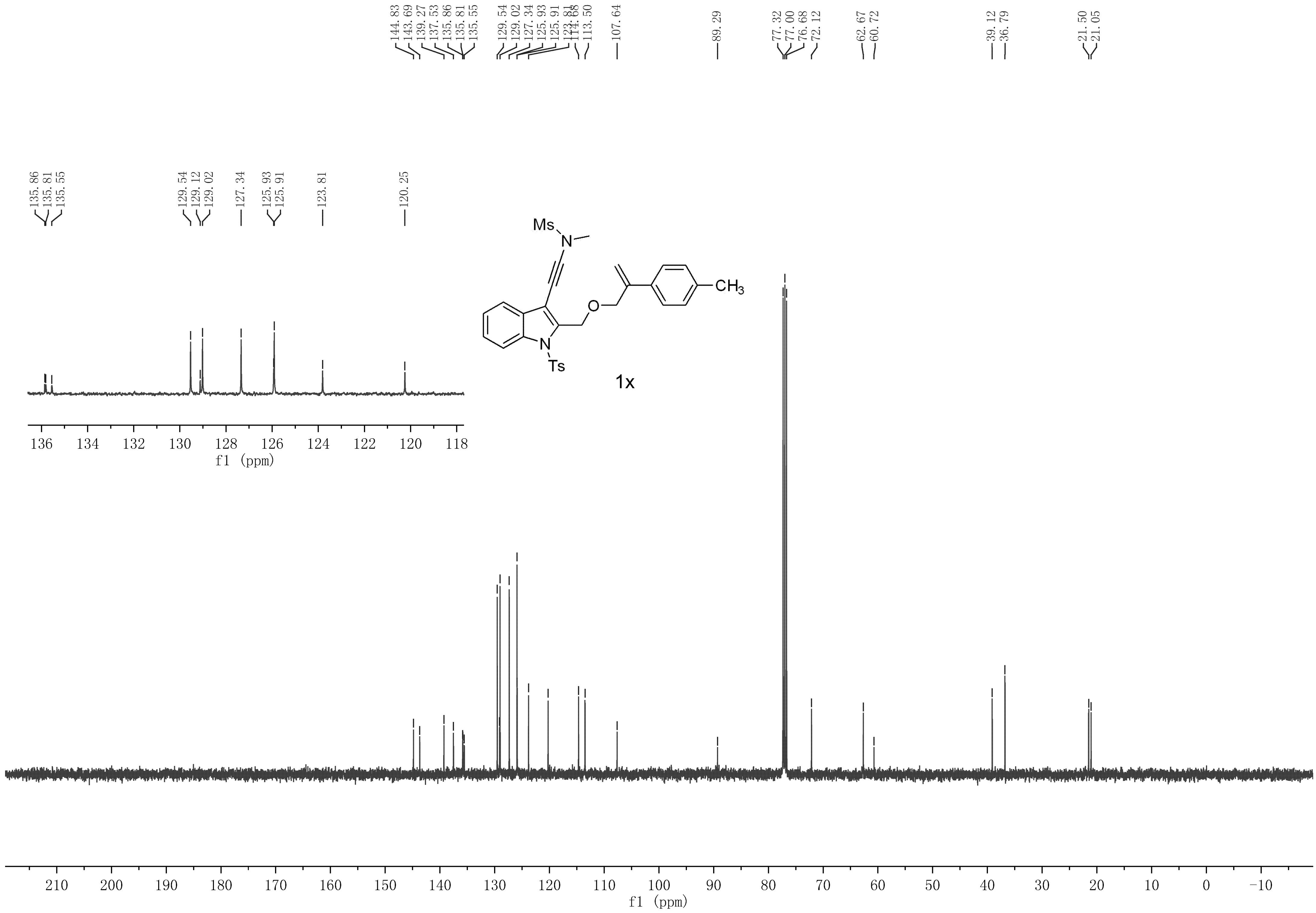
144.83
143.69
139.27
137.53
135.86
135.81
135.55
129.54
129.02
127.34
125.93
125.91
123.81
113.50
107.64
89.29
77.32
77.00
76.68
72.12
62.67
60.72
39.12
36.79
21.50
21.05
135.86
135.81
135.55
129.54
129.12
129.02
127.34
125.93
125.91
123.81
120.25
136 134 132 130 128 126 124 122 120 118
f1 (ppm)
Ms
N
O
CH_3
N
Ts
1x
210 200 190 180 170 160 150 140 130 120 110 100 90 80 70 60 50 40 30 20 10 0 -10
f1 (ppm)

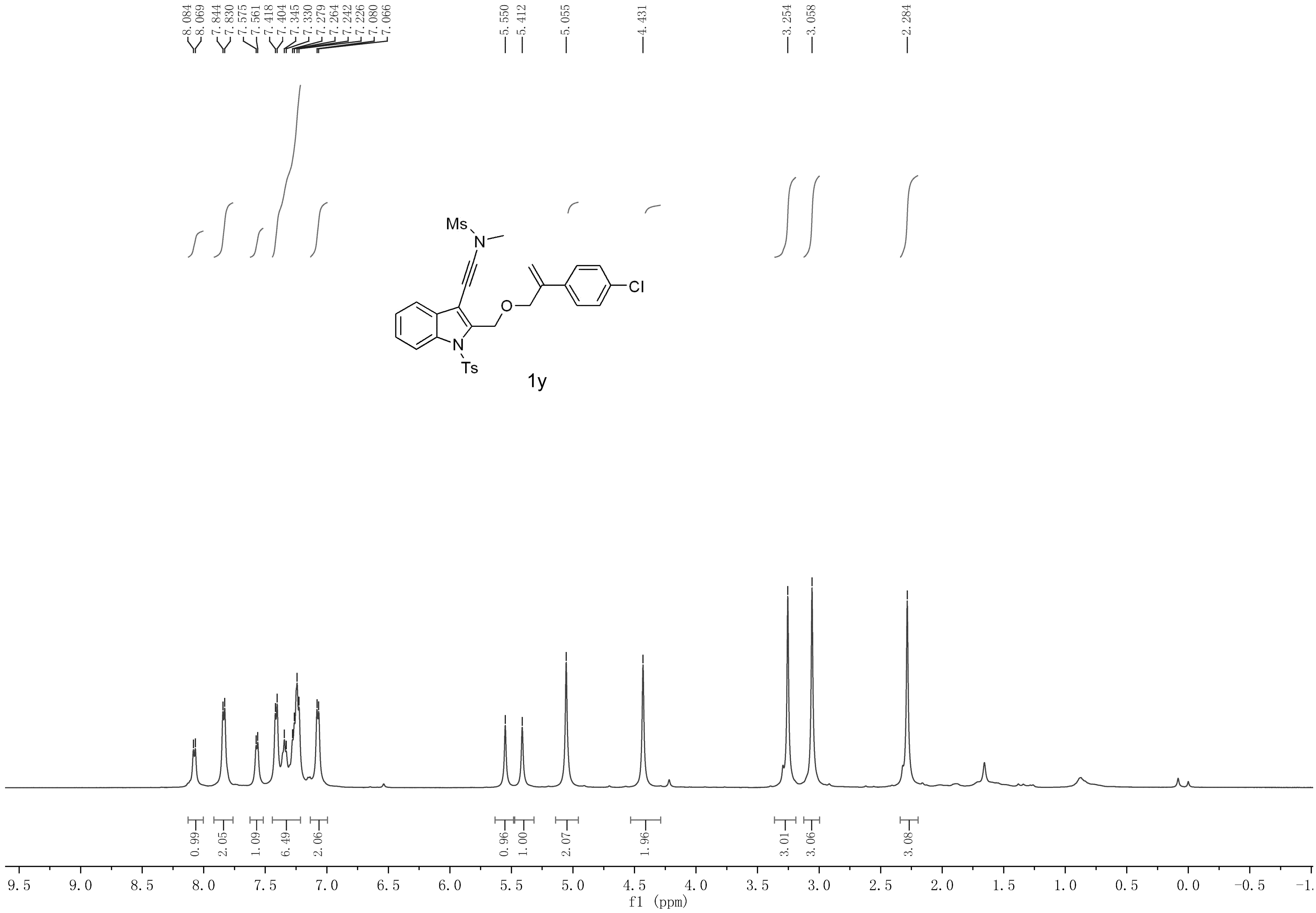
8.084
8.069
7.844
7.830
7.575
7.561
7.418
7.404
7.345
7.330
7.279
7.264
7.242
7.226
7.080
7.066
5.550
5.412
5.055
4.431
3.254
3.058
2.284
Ms
N
O
Cl
N
Ts
1y
0.99
2.05
1.09
6.49
2.06
0.96
1.00
2.07
1.96
3.01
3.06
3.08
9.5 9.0 8.5 8.0 7.5 7.0 6.5 6.0 5.5 5.0 4.5 4.0 3.5 3.0 2.5 2.0 1.5 1.0 0.5 0.0 -0.5 -1.
f1 (ppm)

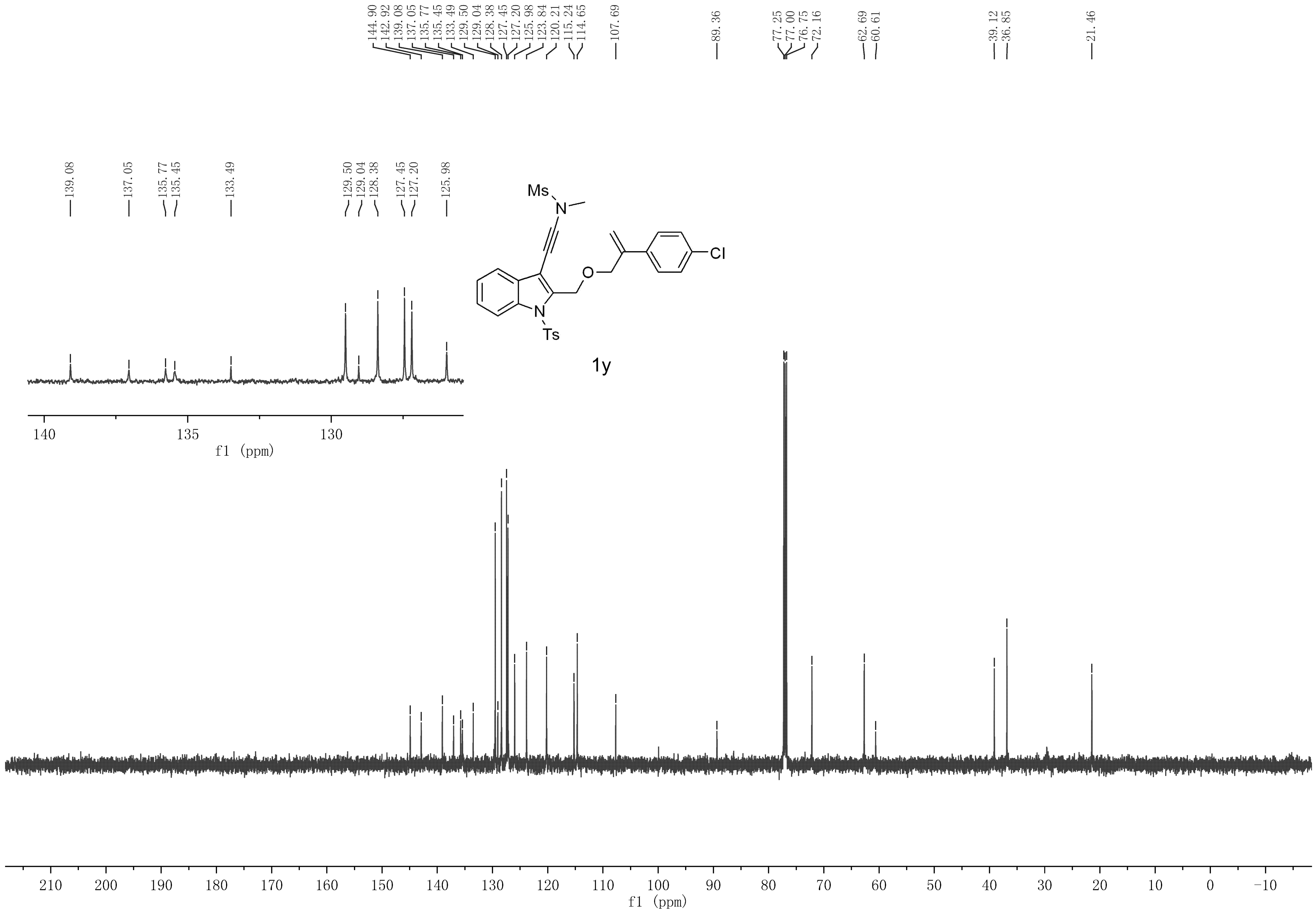

144.90
142.92
139.08
137.05
135.77
135.45
133.49
129.50
129.04
128.38
127.45
127.20
125.98
123.84
120.21
115.24
114.65
107.69
89.36
77.25
77.00
76.75
72.16
62.69
60.61
39.12
36.85
21.46
Ms
N
Cl
O
N
Ts
1y
140
135
130
f1 (ppm)
210 200 190 180 170 160 150 140 130 120 110 100 90 80 70 60 50 40 30 20 10 0 -10
f1 (ppm)

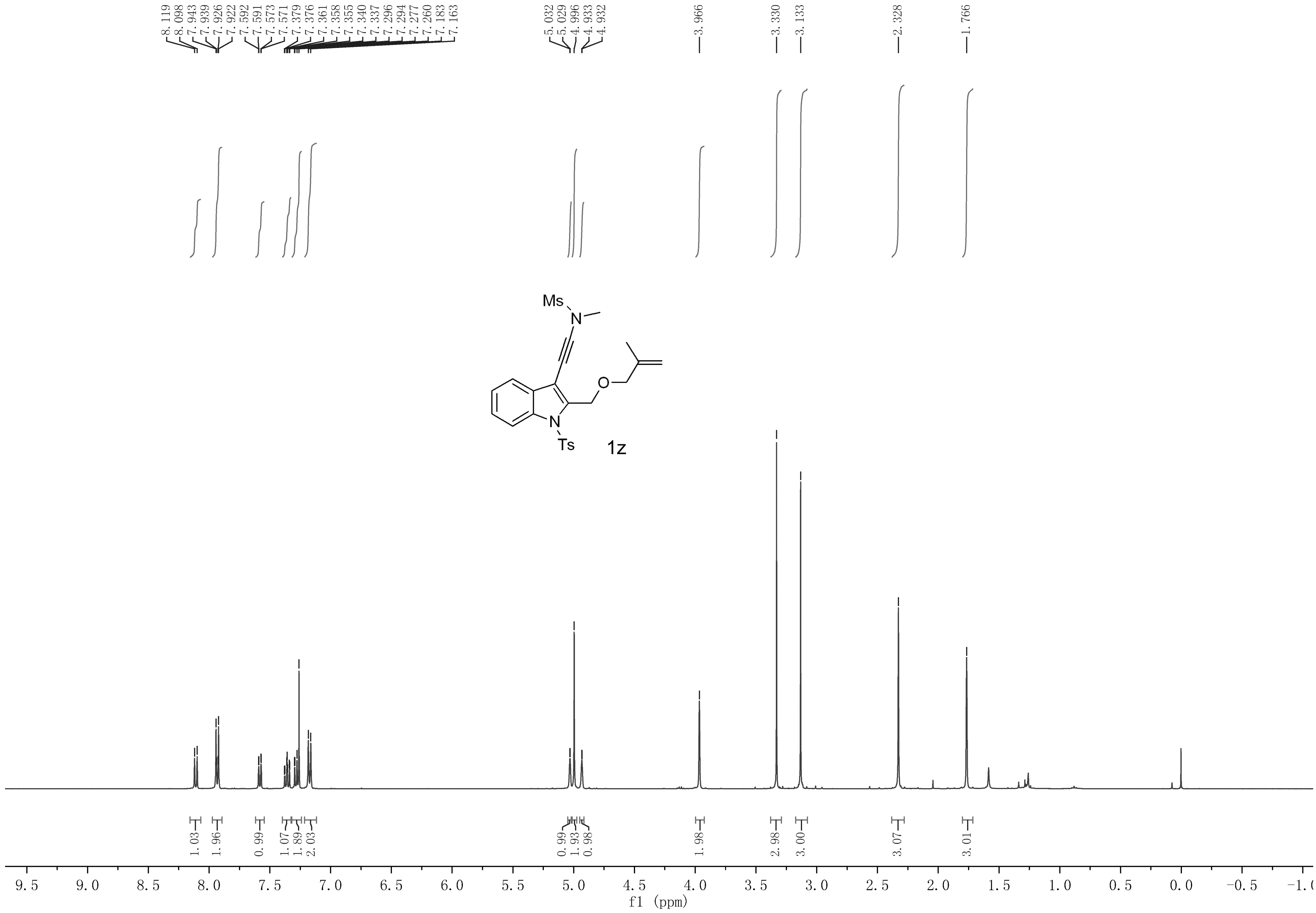
8.119 8.098 7.943 7.939 7.926 7.922 7.592 7.591 7.573 7.571 7.379 7.376 7.361 7.358 7.355 7.340 7.337 7.296 7.294 7.277 7.260 7.183 7.163
5.032 5.029 4.996 4.933 4.932
3.966
3.330
3.133
2.328
1.766
Ms
N
O
N
Ts
1z
1.03 1.96 0.99 1.07 1.89 2.03
0.99 1.93 0.98
1.98
2.98
3.00
3.07
3.01
9.5 9.0 8.5 8.0 7.5 7.0 6.5 6.0 5.5 5.0 4.5 4.0 3.5 3.0 2.5 2.0 1.5 1.0 0.5 0.0 −0.5
f1 (ppm)

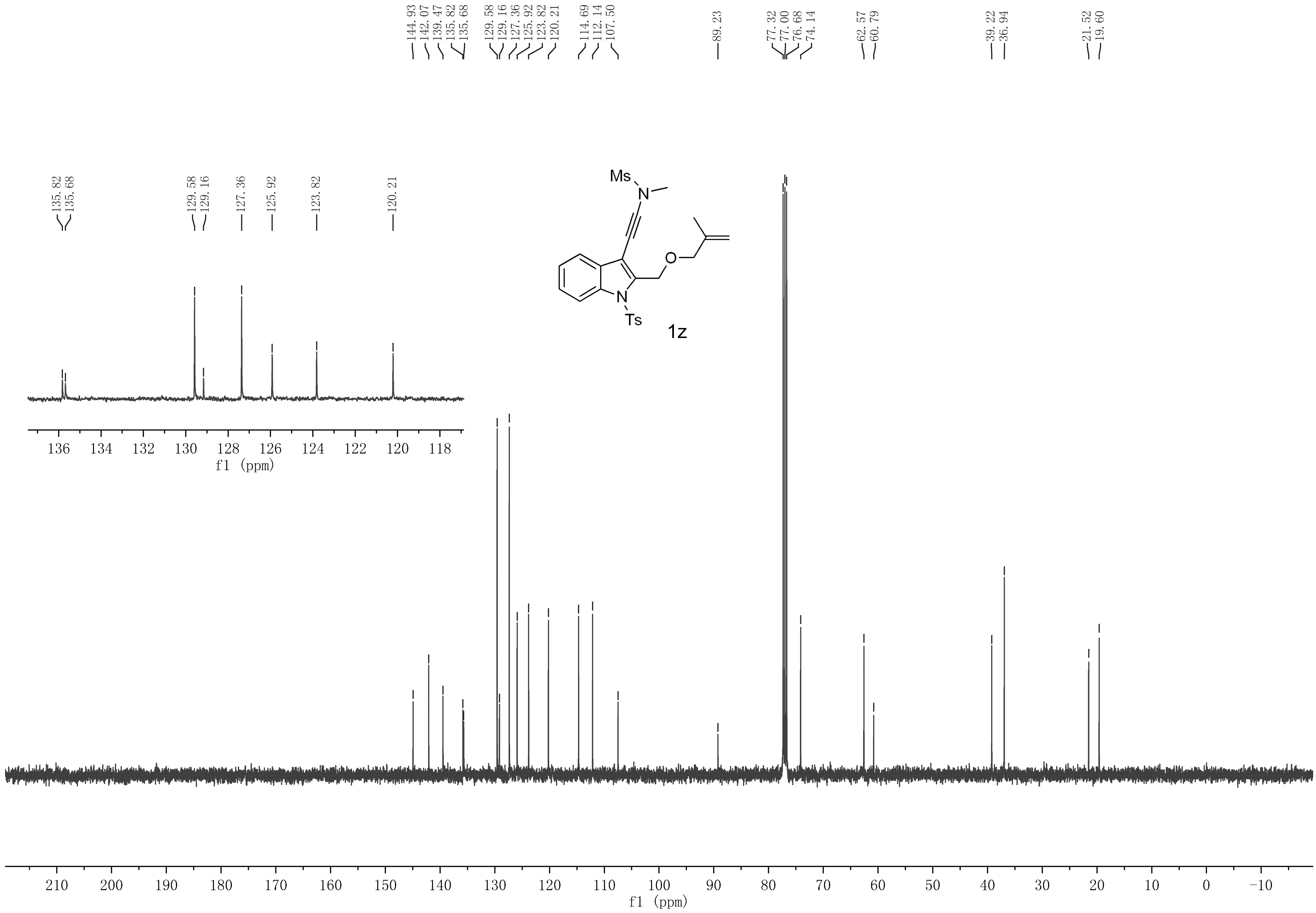
144.93
142.07
139.47
135.82
135.68
129.58
129.16
127.36
125.92
123.82
120.21
114.69
112.14
107.50
89.23
77.32
77.00
76.68
74.14
62.57
60.79
39.22
36.94
21.52
19.60
Ms
N
O
N
Ts
1z
136 134 132 130 128 126 124 122 120 118
f1 (ppm)
210 200 190 180 170 160 150 140 130 120 110 100 90 80 70 60 50 40 30 20 10 0 −10
f1 (ppm)

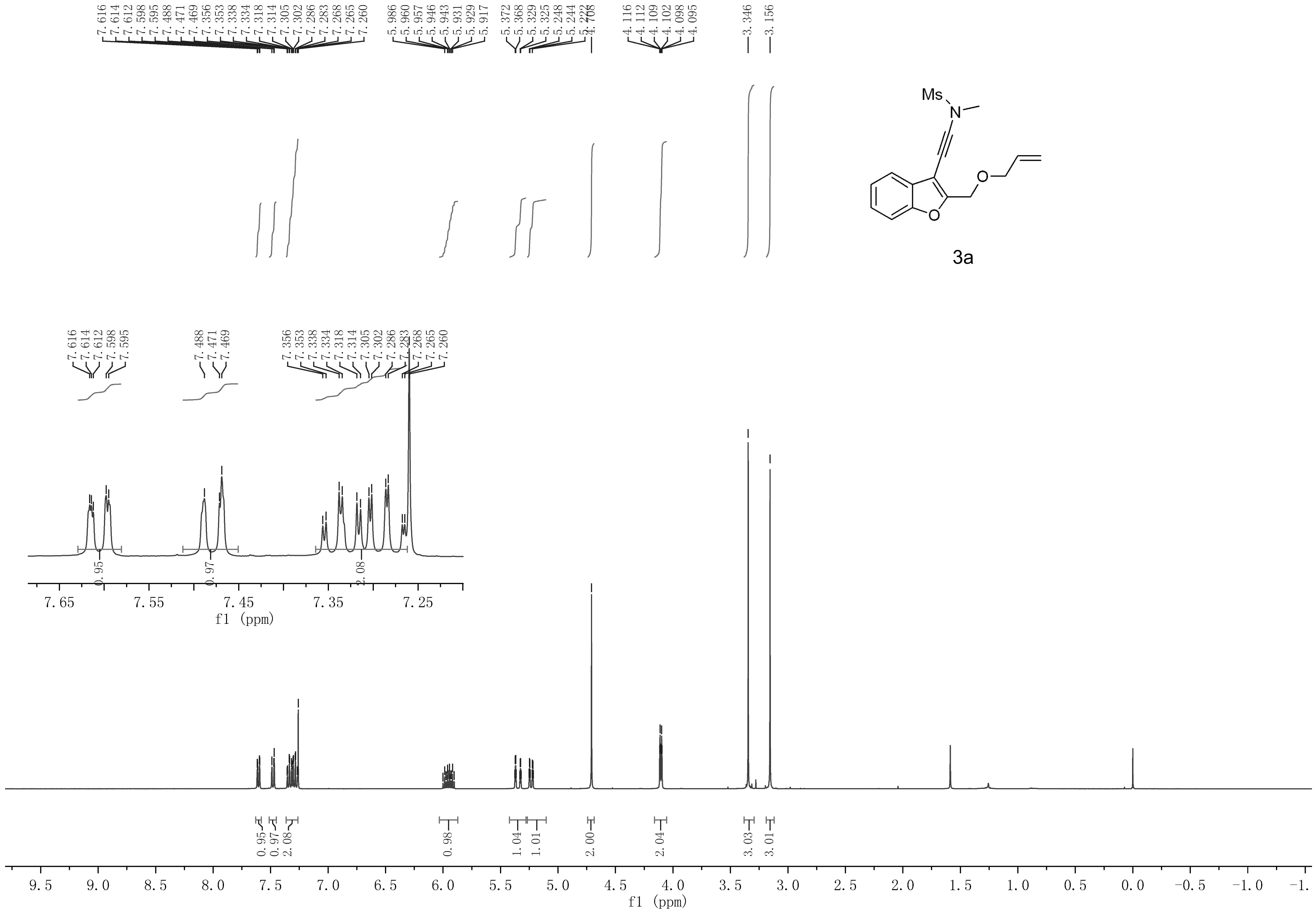

7.616
7.614
7.612
7.598
7.595
7.488
7.471
7.469
7.356
7.353
7.338
7.334
7.318
7.314
7.305
7.302
7.286
7.283
7.268
7.265
7.260
5.986
5.960
5.957
5.946
5.943
5.931
5.929
5.917
5.372
5.368
5.329
5.325
5.248
5.244
4.708
4.116
4.112
4.109
4.102
4.098
4.095
3.346
3.156
Ms
N
O
3a
0.95
0.97
2.08
7.65
7.55
7.45
7.35
7.25
f1 (ppm)
0.98
1.04
1.01
2.00
2.04
3.03
3.01
9.5
9.0
8.5
8.0
7.5
7.0
6.5
6.0
5.5
5.0
4.5
4.0
3.5
3.0
2.5
2.0
1.5
1.0
0.5
0.0
-0.5
-1.0
f1 (ppm)

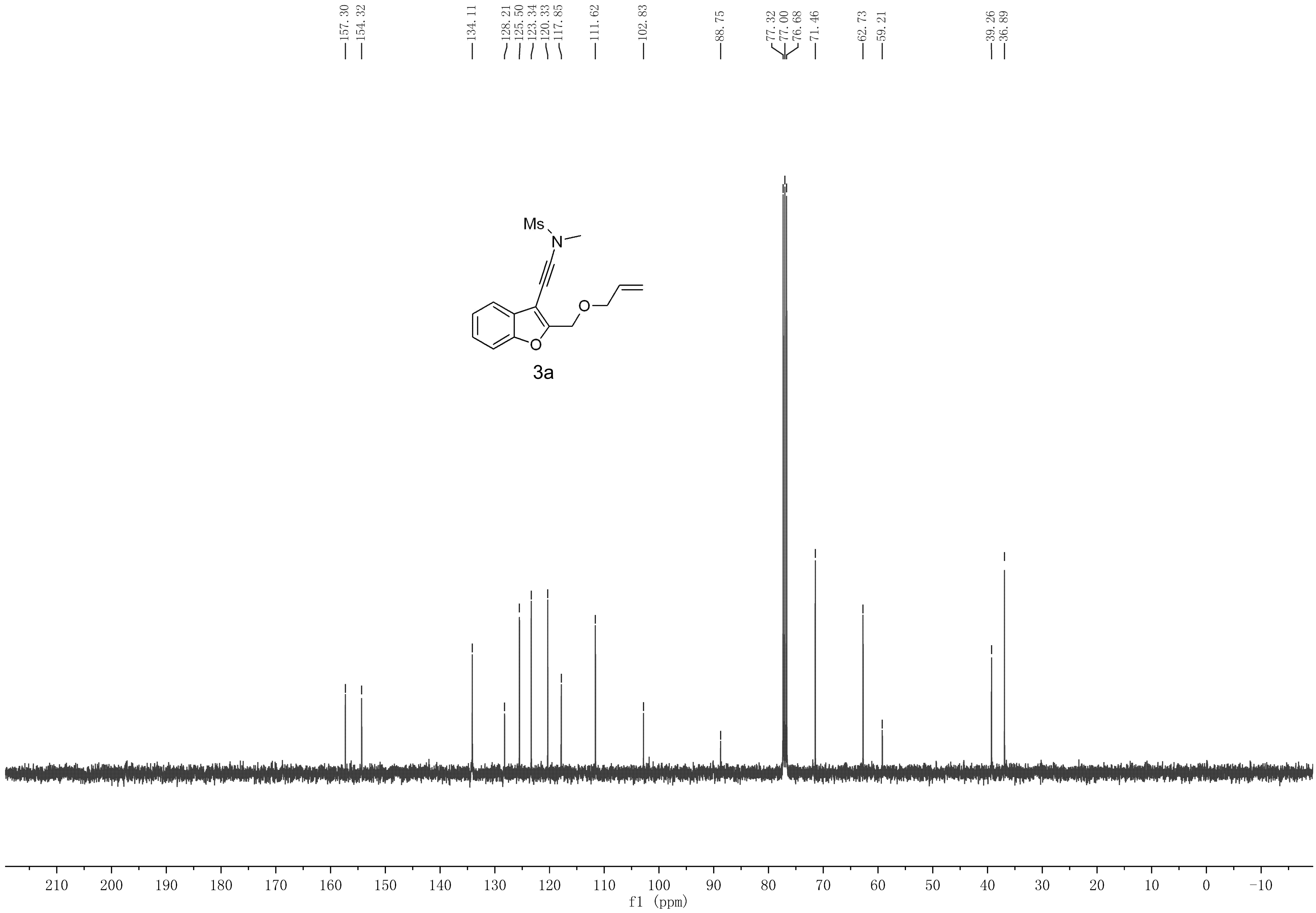
157.30
154.32
134.11
128.21
125.50
123.34
120.33
117.85
111.62
102.83
88.75
77.32
77.00
76.68
71.46
62.73
59.21
39.26
36.89
Ms
N
O
O
3a
210 200 190 180 170 160 150 140 130 120 110 100 90 80 70 60 50 40 30 20 10 0 −10
f1 (ppm)

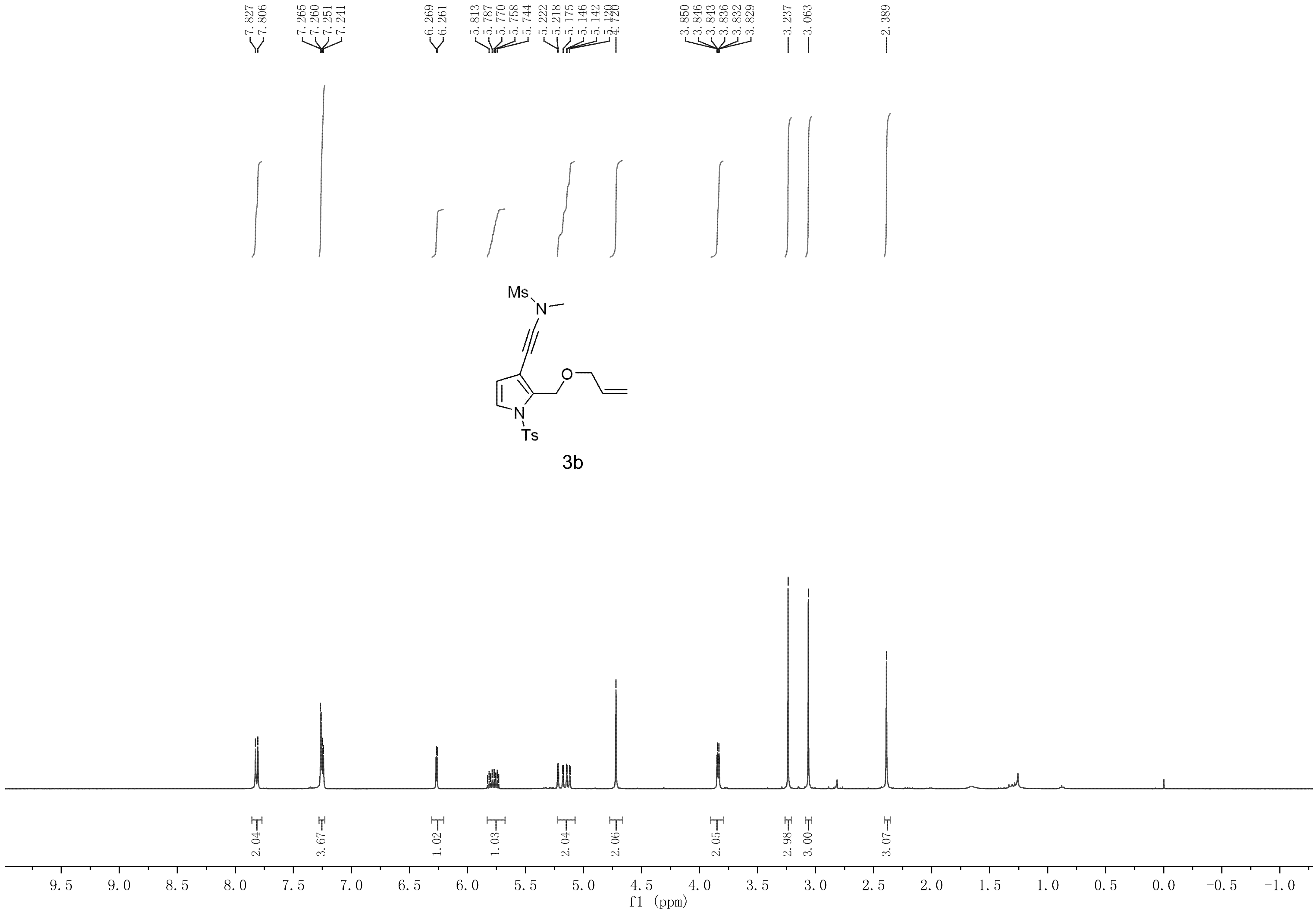
7.827
7.806
7.265
7.260
7.251
7.241
6.269
6.261
5.813
5.787
5.770
5.758
5.744
5.222
5.218
5.175
5.146
5.142
5.120
4.720
3.850
3.846
3.843
3.836
3.832
3.829
3.237
3.063
2.389
Ms
N
O
N
Ts
3b
2.04
3.67
1.02
1.03
2.04
2.06
2.05
2.98
3.00
3.07
9.5
9.0
8.5
8.0
7.5
7.0
6.5
6.0
5.5
5.0
4.5
4.0
3.5
3.0
2.5
2.0
1.5
1.0
0.5
0.0
-0.5
-1.0
f1 (ppm)

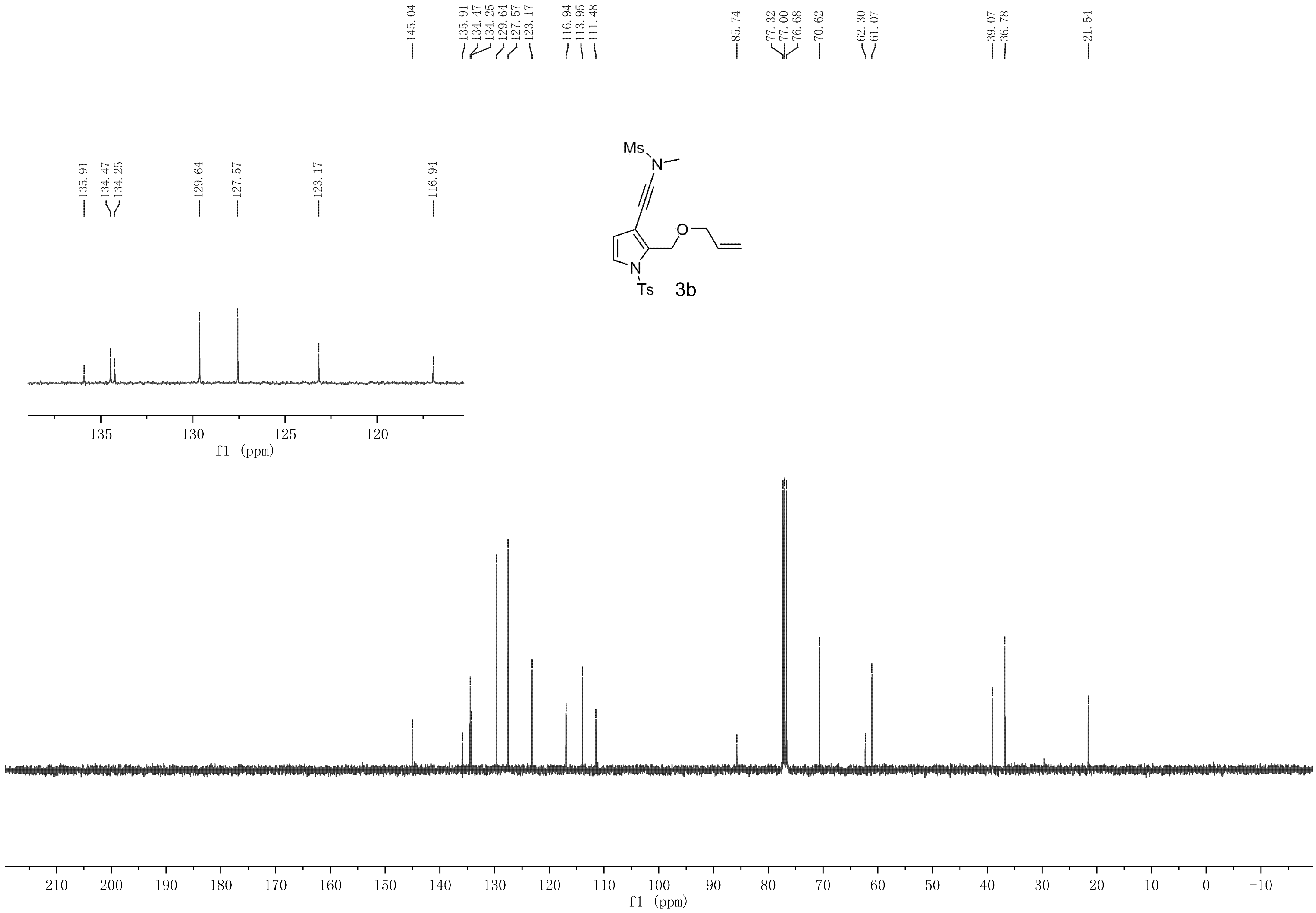
145.04
135.91
134.47
134.25
129.64
127.57
123.17
116.94
113.95
111.48
85.74
77.32
77.00
76.68
70.62
62.30
61.07
39.07
36.78
21.54
Ms
N
O
N
Ts
3b
135.91
134.47
134.25
129.64
127.57
123.17
116.94
135
130
125
120
f1 (ppm)
210
200
190
180
170
160
150
140
130
120
110
100
90
80
70
60
50
40
30
20
10
0
−10
f1 (ppm)

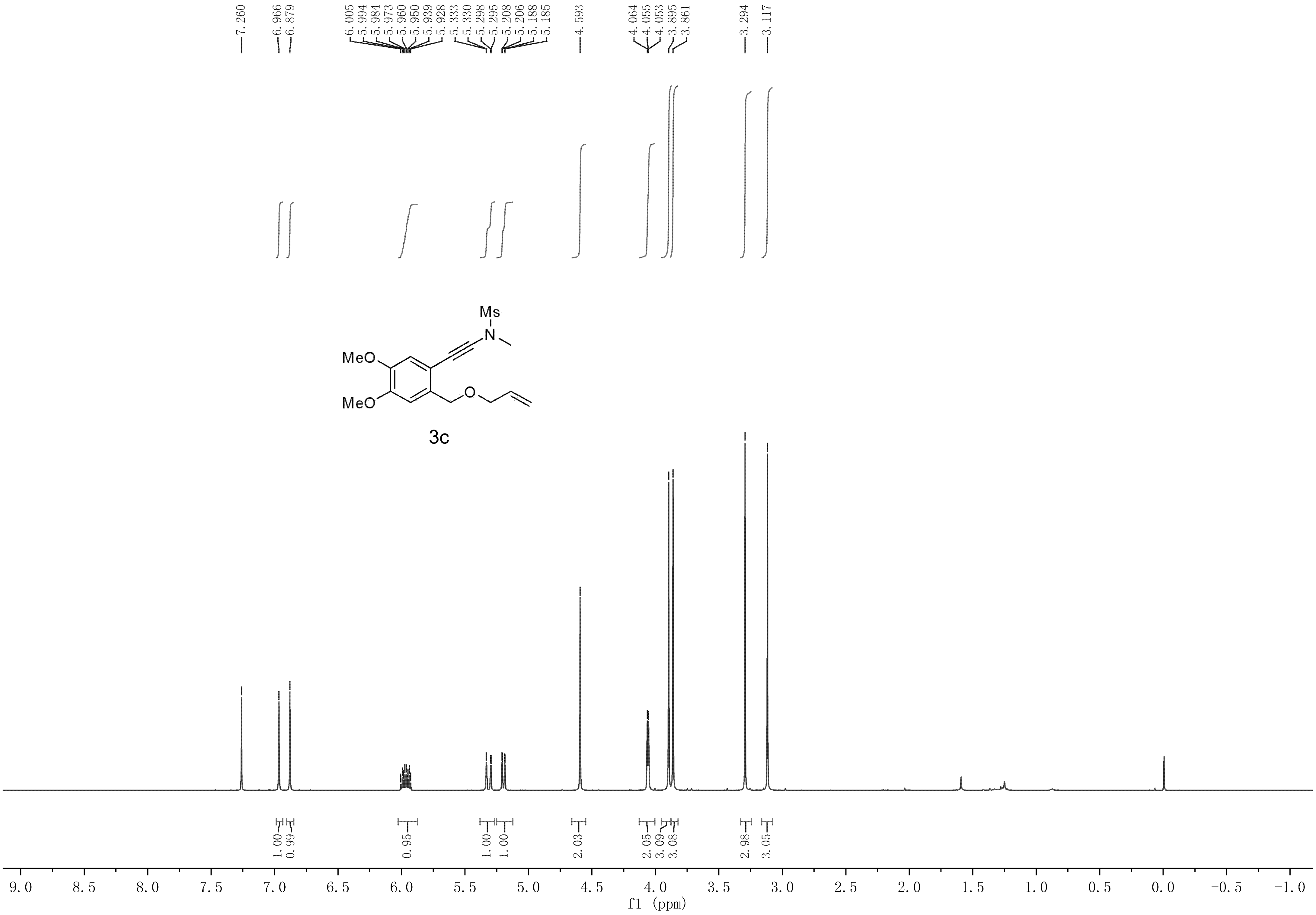

7.260
6.966
6.879
6.005
5.994
5.984
5.973
5.960
5.950
5.939
5.928
5.333
5.330
5.298
5.295
5.208
5.206
5.188
5.185
4.593
4.064
4.055
4.053
3.895
3.861
3.294
3.117
Ms
N
MeO
MeO
O
3c
1.00
0.99
0.95
1.00
1.00
2.03
2.05
3.09
3.08
2.98
3.05
9.0
8.5
8.0
7.5
7.0
6.5
6.0
5.5
5.0
4.5
4.0
3.5
3.0
2.5
2.0
1.5
1.0
0.5
0.0
-0.5
-1.0
f1 (ppm)

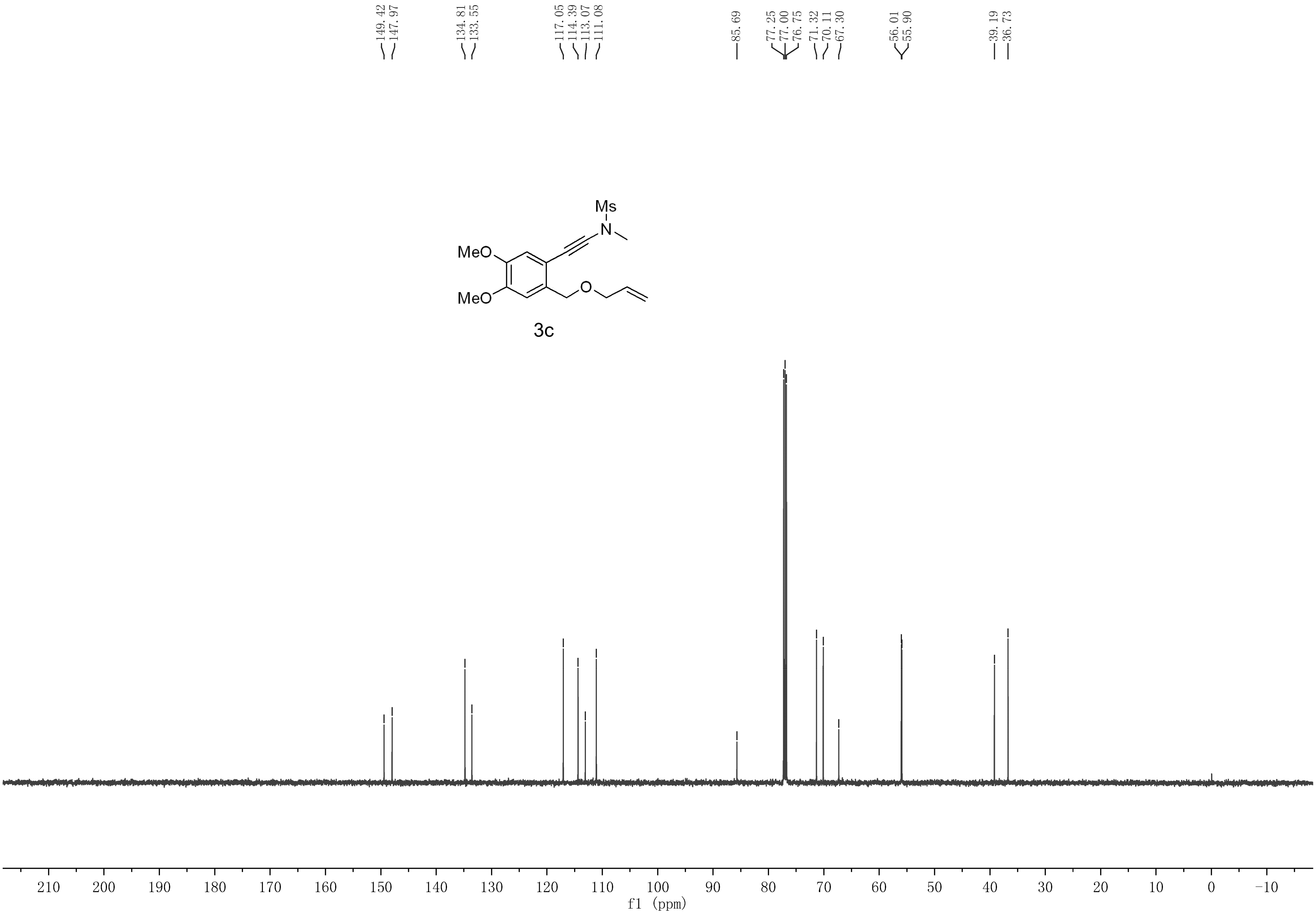
149.42
147.97
134.81
133.55
117.05
114.39
113.07
111.08
85.69
77.25
77.00
76.75
71.32
70.11
67.30
56.01
55.90
39.19
36.73
Ms
N
MeO
MeO
O
3c
210 200 190 180 170 160 150 140 130 120 110 100 90 80 70 60 50 40 30 20 10 0 −10
f1 (ppm)

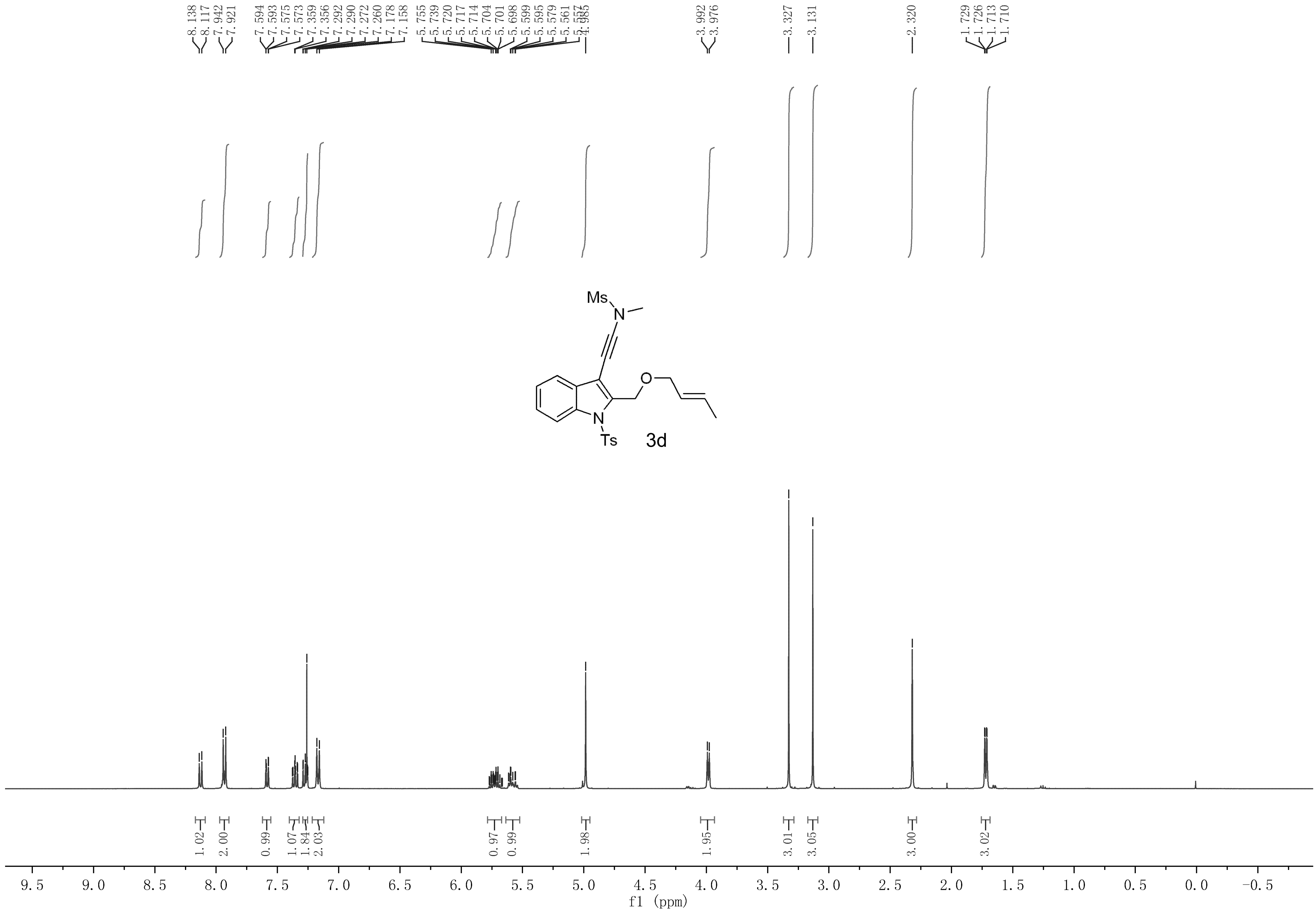
8.138
8.117
7.942
7.921
7.594
7.593
7.575
7.573
7.359
7.356
7.292
7.290
7.272
7.260
7.178
7.158
5.755
5.739
5.720
5.717
5.714
5.704
5.701
5.698
5.599
5.595
5.579
5.561
4.985
3.992
3.976
3.327
3.131
2.320
1.729
1.726
1.713
1.710
Ms
N
O
N
Ts
3d
1.02
2.00
0.99
1.07
1.84
2.03
0.97
0.99
1.98
1.95
3.01
3.05
3.00
3.02
9.5
9.0
8.5
8.0
7.5
7.0
6.5
6.0
5.5
5.0
4.5
4.0
3.5
3.0
2.5
2.0
1.5
1.0
0.5
0.0
-0.5
f1 (ppm)

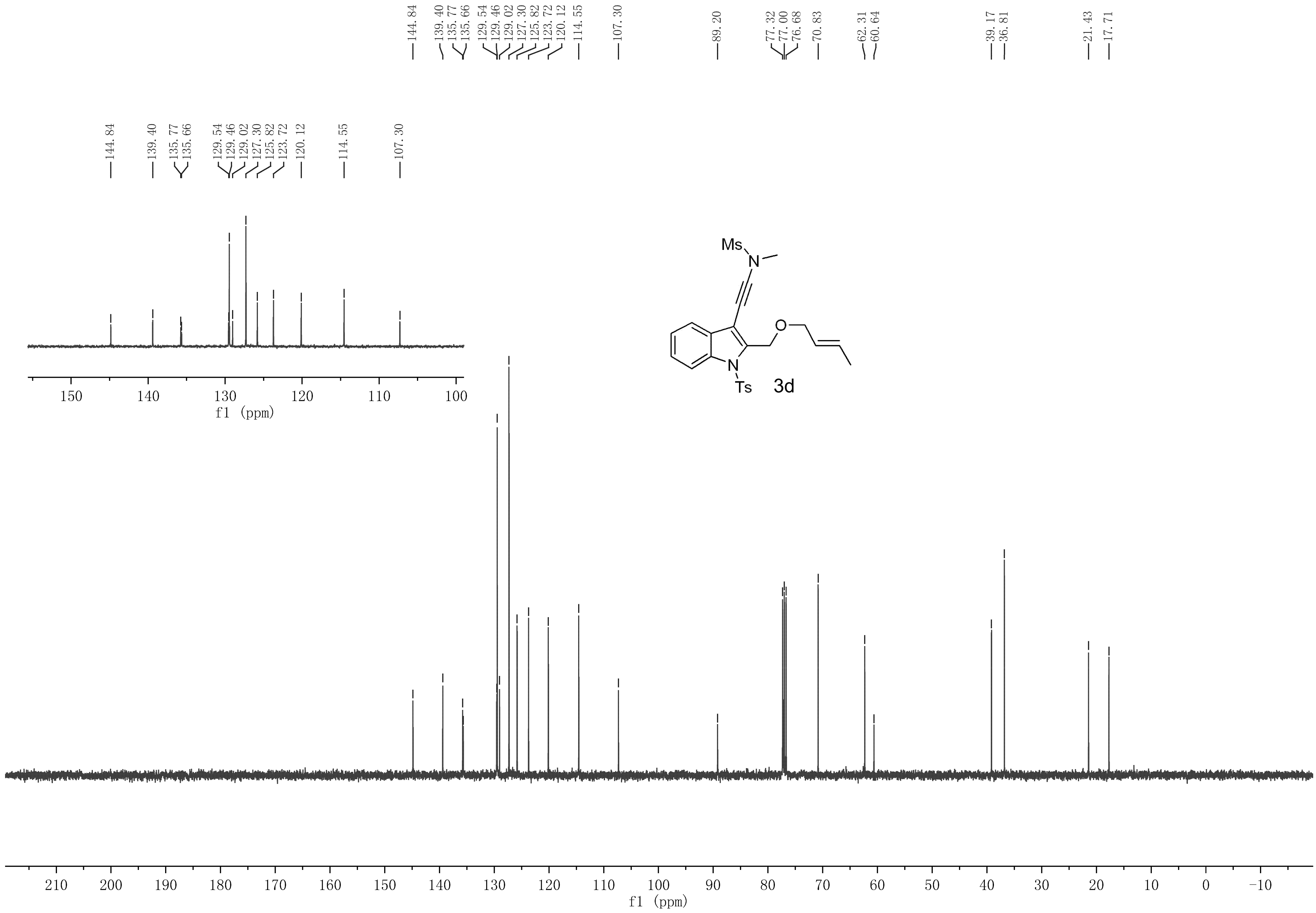
144.84
139.40
135.77
135.66
129.54
129.46
129.02
127.30
125.82
123.72
120.12
114.55
107.30
89.20
77.32
77.00
76.68
70.83
62.31
60.64
39.17
36.81
21.43
17.71
Ms
N
O
N
Ts
3d
150 140 130 120 110 100
f1 (ppm)
210 200 190 180 170 160 150 140 130 120 110 100 90 80 70 60 50 40 30 20 10 0 −10
f1 (ppm)

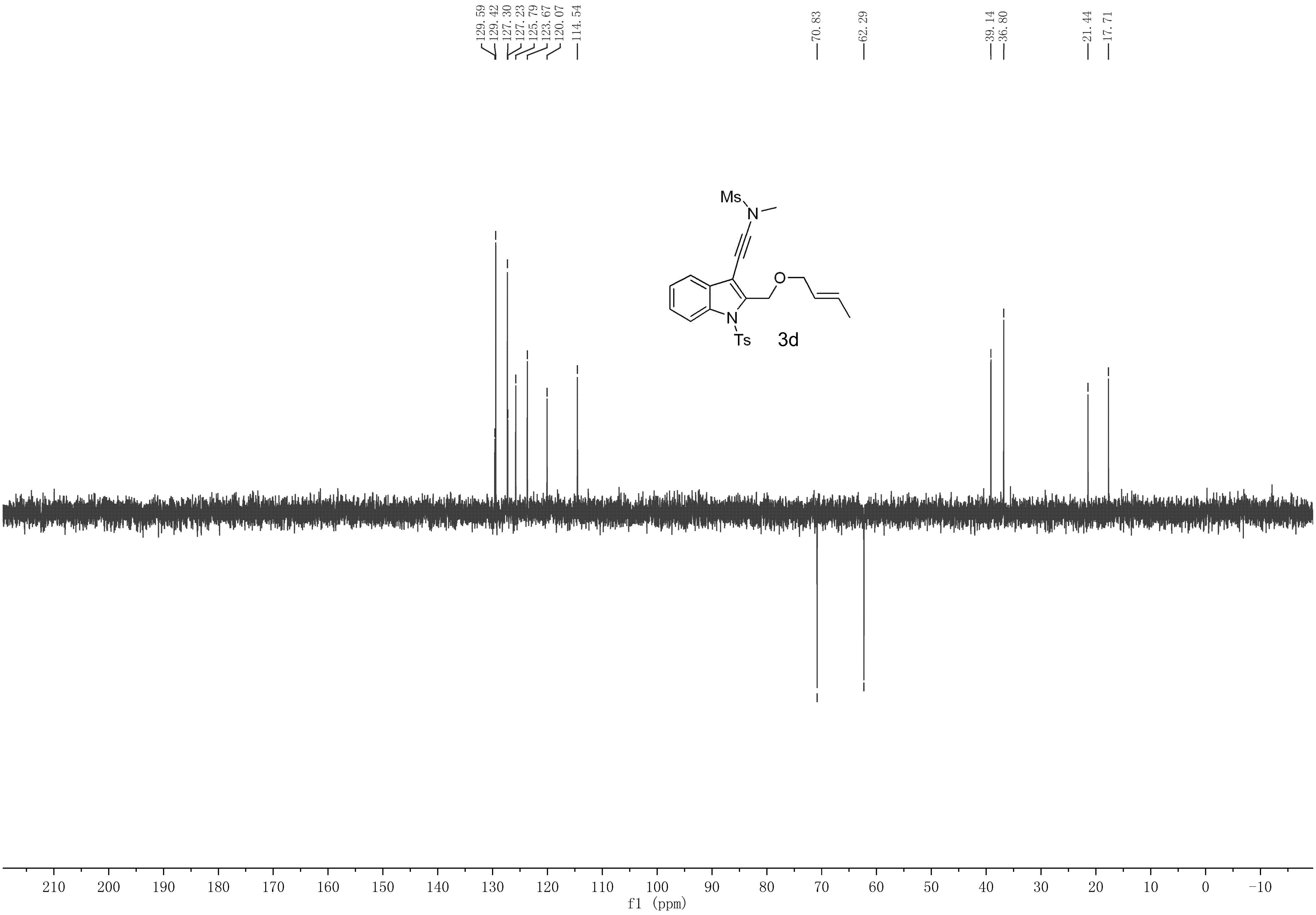
129.59
129.42
127.30
127.23
125.79
123.67
120.07
114.54
70.83
62.29
39.14
36.80
21.44
17.71
Ms
N
O
N
Ts
3d
210 200 190 180 170 160 150 140 130 120 110 100 90 80 70 60 50 40 30 20 10 0 −10
f1 (ppm)

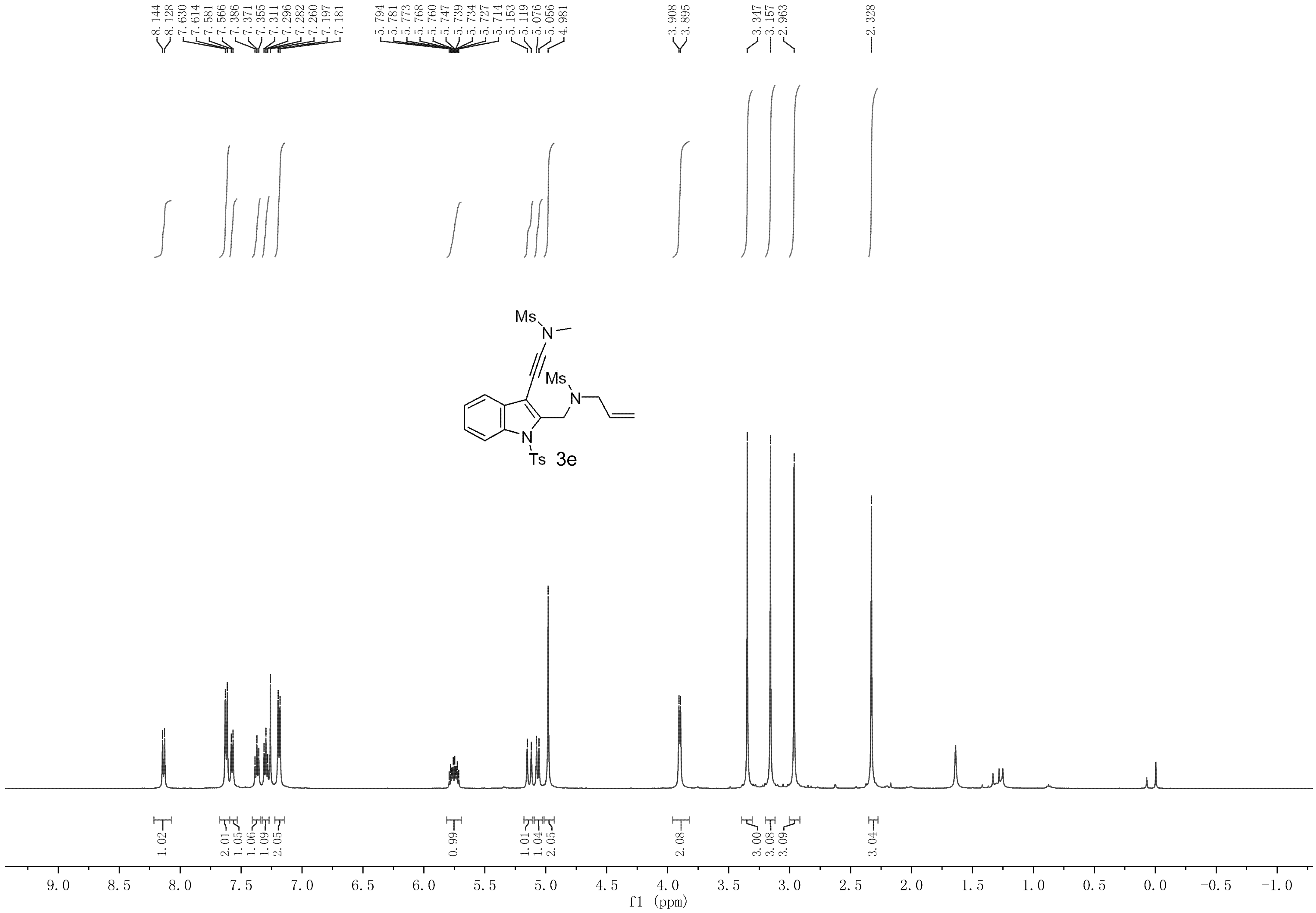
8.144
8.128
7.630
7.614
7.581
7.566
7.386
7.371
7.355
7.311
7.296
7.282
7.260
7.197
7.181
5.794
5.781
5.773
5.768
5.760
5.747
5.739
5.734
5.727
5.714
5.153
5.119
5.076
5.056
4.981
3.908
3.895
3.347
3.157
2.963
2.328
Ms
N
Ms
N
N
Ts
3e
1.02
2.01
1.05
1.06
1.09
2.05
0.99
1.01
1.04
2.05
2.08
3.00
3.08
3.09
3.04
9.0
8.5
8.0
7.5
7.0
6.5
6.0
5.5
5.0
4.5
4.0
3.5
3.0
2.5
2.0
1.5
1.0
0.5
0.0
−0.5
−1.0
f1 (ppm)

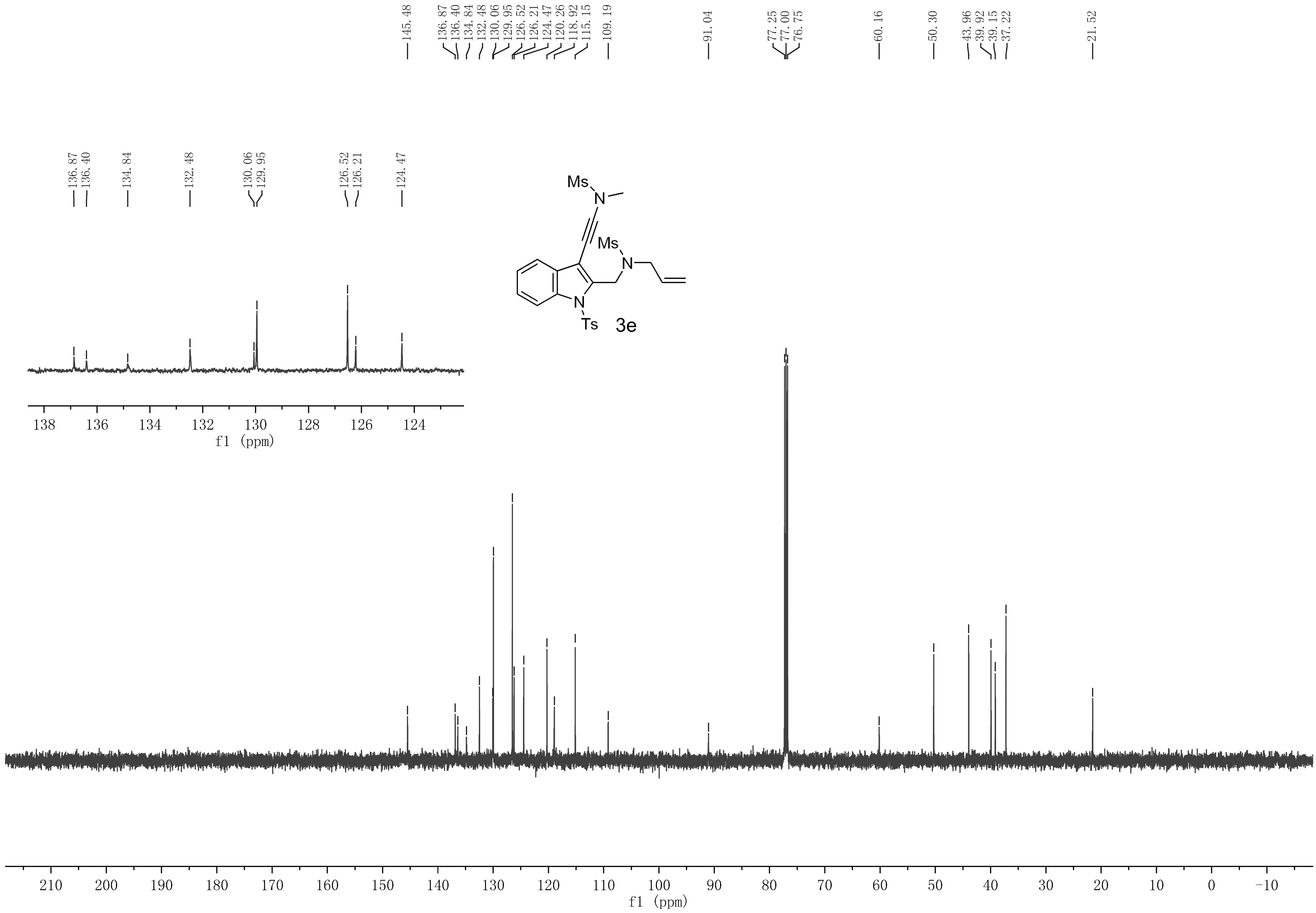
145.48
136.87
136.40
134.84
132.48
130.06
129.95
126.52
126.21
124.47
120.26
118.92
115.15
109.19
91.04
77.25
77.00
76.75
60.16
50.30
43.96
39.92
39.15
37.22
21.52
Ms
N
Ms
N
N
Ts
3e
138 136 134 132 130 128 126 124
f1 (ppm)
210 200 190 180 170 160 150 140 130 120 110 100 90 80 70 60 50 40 30 20 10 0 −10
f1 (ppm)

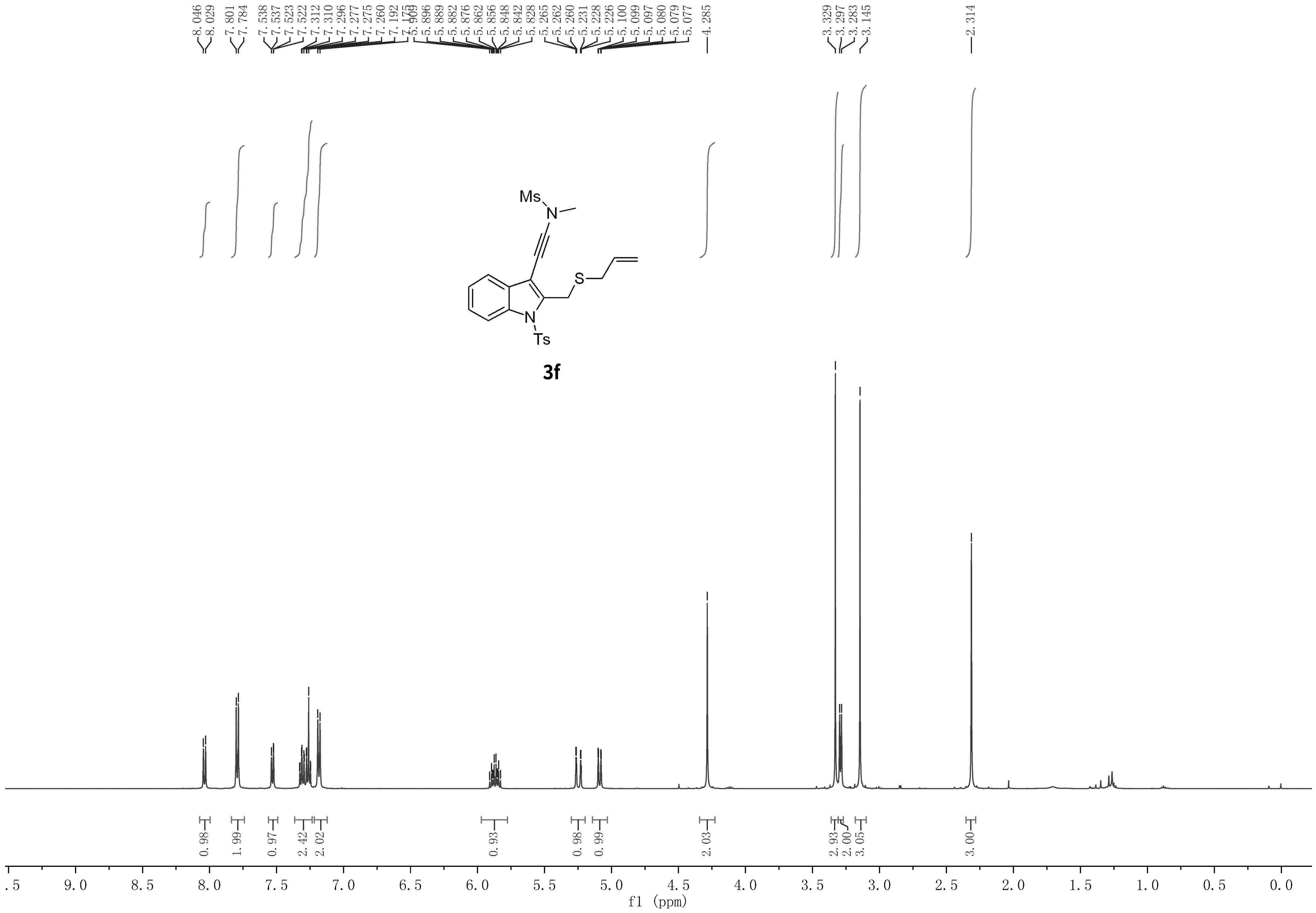
8.046
8.029
7.801
7.784
7.538
7.537
7.523
7.522
7.312
7.310
7.296
7.277
7.275
7.260
7.192
7.175
5.909
5.896
5.889
5.882
5.876
5.862
5.856
5.848
5.842
5.828
5.265
5.262
5.260
5.231
5.228
5.226
5.100
5.099
5.097
5.080
5.079
5.077
4.285
3.329
3.297
3.283
3.145
2.314
Ms
N
S
N
Ts
3f
0.98
1.99
0.97
2.42
2.02
0.93
0.98
0.99
2.03
2.93
2.00
3.05
3.00
.5
9.0
8.5
8.0
7.5
7.0
6.5
6.0
5.5
5.0
4.5
4.0
3.5
3.0
2.5
2.0
1.5
1.0
0.5
0.0
f1 (ppm)

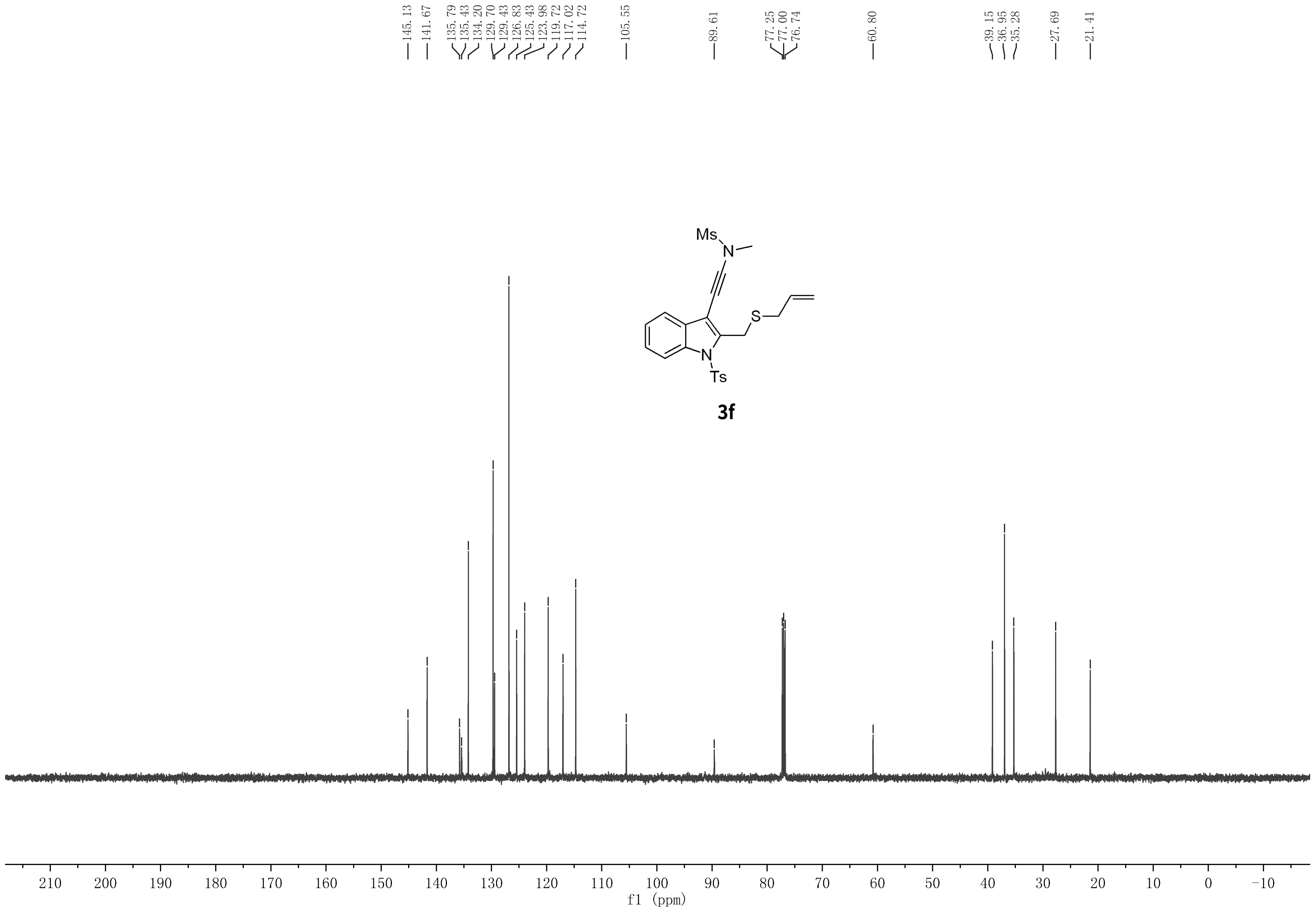
145.13
141.67
135.79
135.43
134.20
129.70
129.43
126.83
125.43
123.98
119.72
117.02
114.72
105.55
89.61
77.25
77.00
76.74
60.80
39.15
36.95
35.28
27.69
21.41
Ms
N
S
N
Ts
3f
210 200 190 180 170 160 150 140 130 120 110 100 90 80 70 60 50 40 30 20 10 0 -10
f1 (ppm)

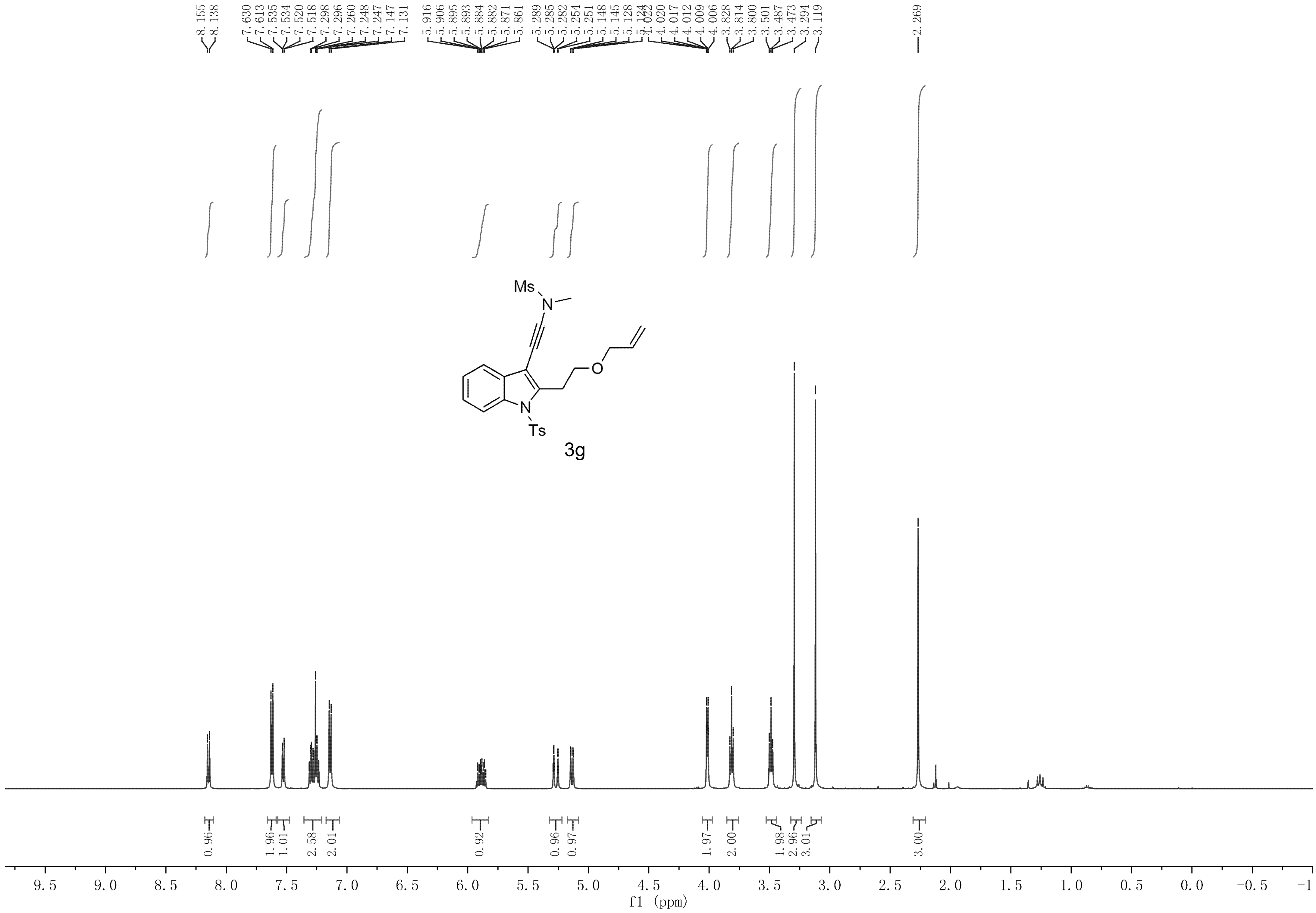
8.155
8.138
7.630
7.613
7.535
7.534
7.520
7.518
7.298
7.296
7.260
7.248
7.247
7.147
7.131
5.916
5.906
5.895
5.893
5.884
5.882
5.871
5.861
5.289
5.285
5.282
5.254
5.251
5.148
5.145
5.128
5.124
4.022
4.020
4.017
4.012
4.009
4.006
3.828
3.814
3.800
3.501
3.487
3.473
3.294
3.119
2.269
Ms
N
O
N
Ts
3g
0.96
1.96
1.01
2.58
2.01
0.92
0.96
0.97
1.97
2.00
1.98
2.96
3.01
3.00
f1 (ppm)

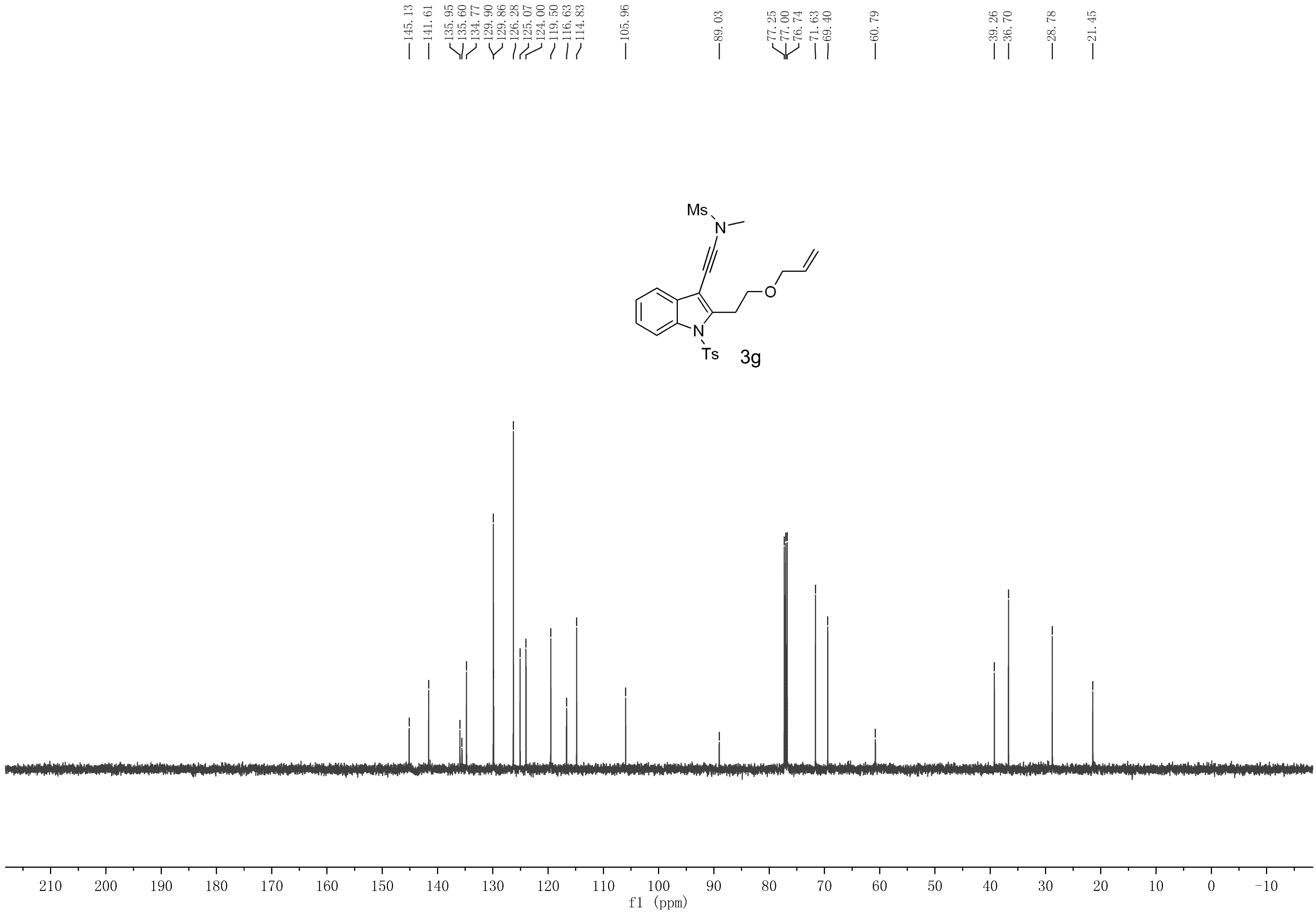
145.13
141.61
135.95
135.60
134.77
129.90
129.86
126.28
125.07
124.00
119.50
116.63
114.83
105.96
89.03
77.25
77.00
76.74
71.63
69.40
60.79
39.26
36.70
28.78
21.45
Ms
N
O
N
Ts
3g
210 200 190 180 170 160 150 140 130 120 110 100 90 80 70 60 50 40 30 20 10 0 -10
f1 (ppm)

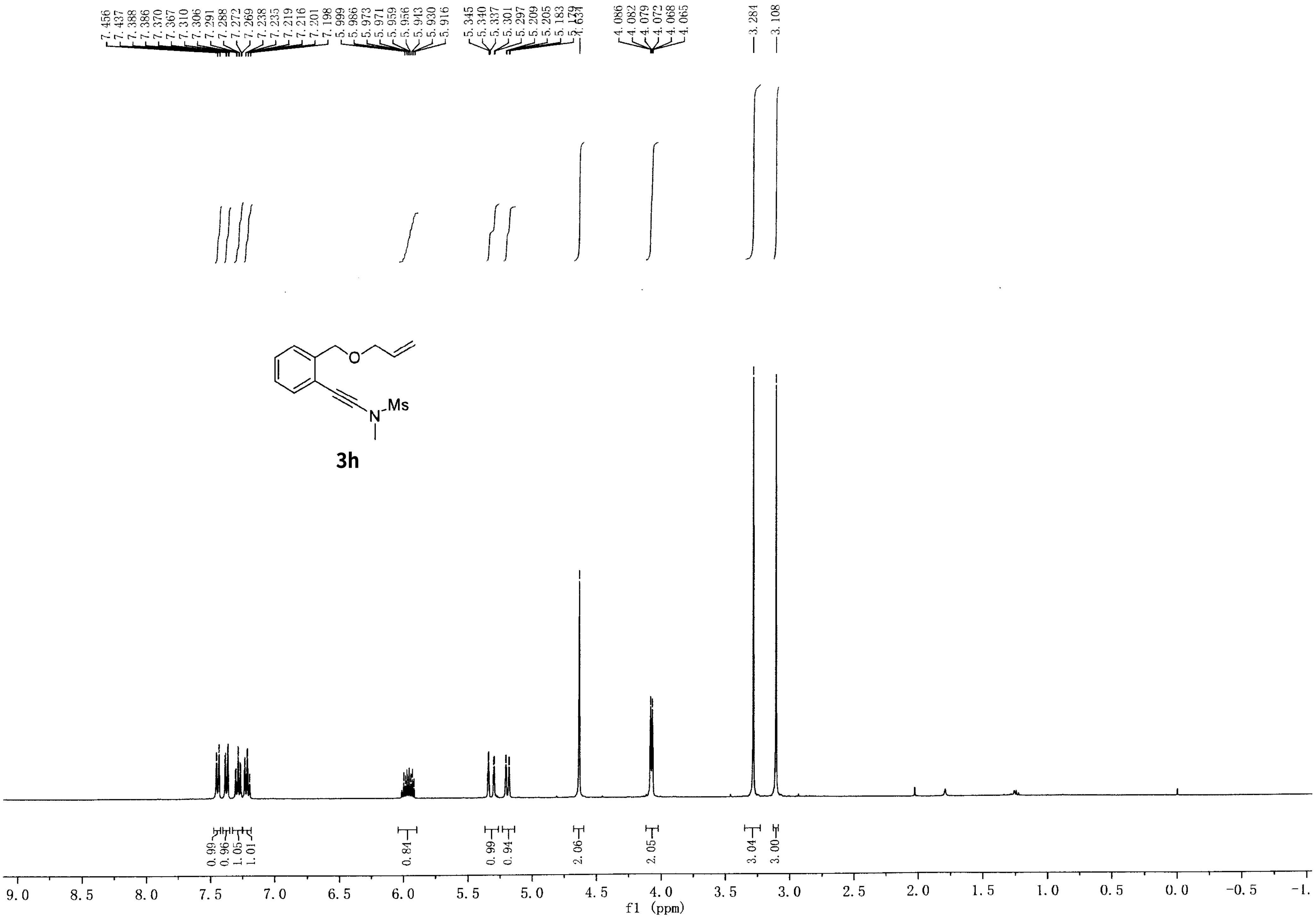
7.456
7.437
7.388
7.386
7.370
7.367
7.310
7.306
7.291
7.288
7.272
7.269
7.238
7.235
7.219
7.216
7.201
7.198
5.999
5.986
5.973
5.971
5.959
5.956
5.943
5.930
5.916
5.345
5.340
5.337
5.301
5.297
5.209
5.205
5.183
5.179
4.631
4.086
4.082
4.079
4.072
4.068
4.065
3.284
3.108
O
N
Ms
3h
0.99
0.96
1.05
1.01
0.84
0.99
0.94
2.06
2.05
3.04
3.00
9.0
8.5
8.0
7.5
7.0
6.5
6.0
5.5
5.0
4.5
4.0
3.5
3.0
2.5
2.0
1.5
1.0
0.5
0.0
-0.5
-1.
f1 (ppm)

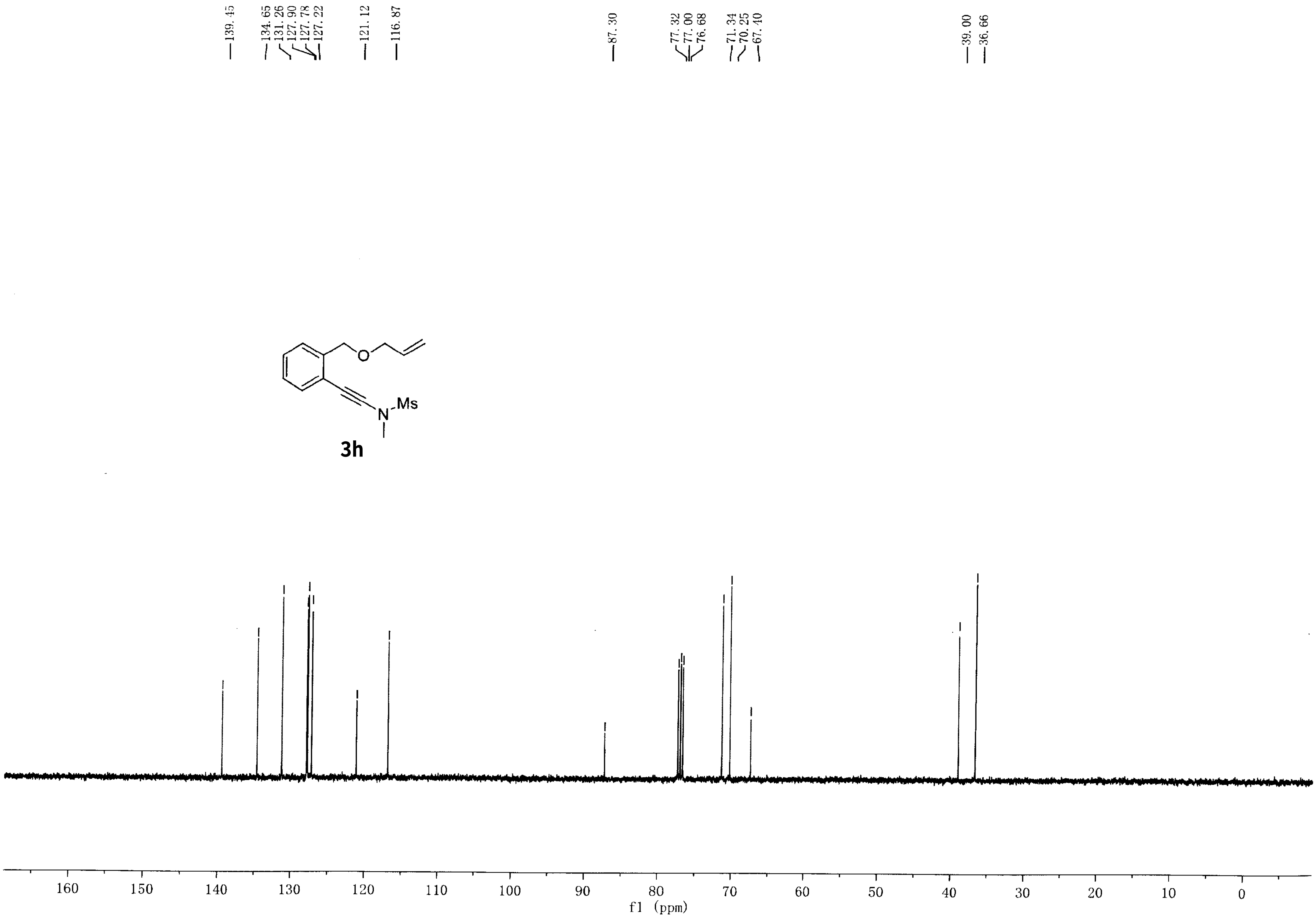
139.45
134.65
131.26
127.90
127.78
127.22
121.12
116.87
87.30
77.32
77.00
76.68
71.34
70.25
67.40
39.00
36.66
N
Ms
160
150
140
130
120
110
100
90
80
70
60
50
40
30
20
10
0
f1 (ppm)

3h

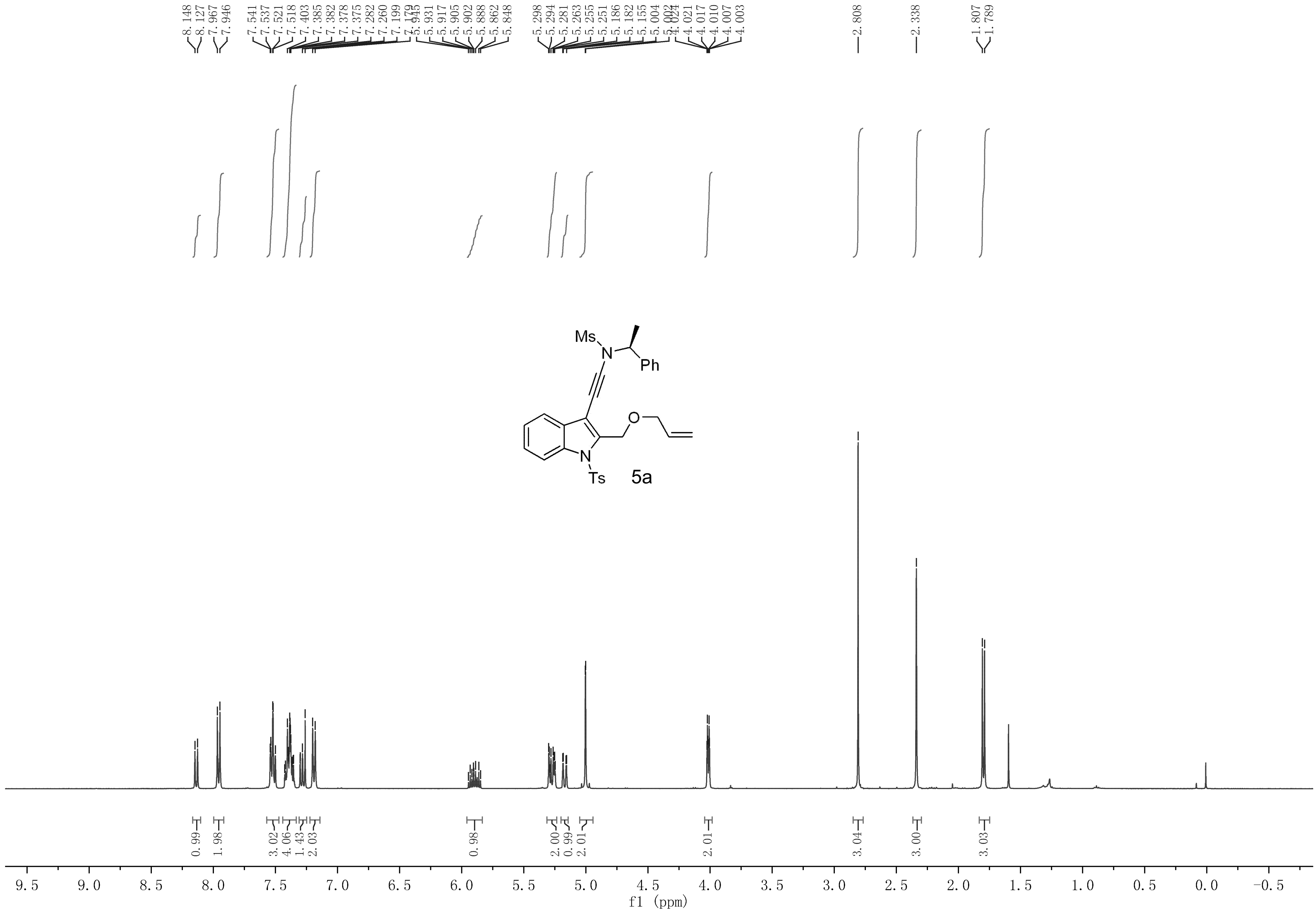
8.148
8.127
7.967
7.946
7.541
7.537
7.521
7.518
7.403
7.385
7.382
7.378
7.375
7.282
7.260
7.199
5.931
5.917
5.905
5.902
5.888
5.862
5.848
5.298
5.294
5.281
5.263
5.255
5.251
5.186
5.182
5.155
5.004
4.021
4.017
4.010
4.007
4.003
2.808
2.338
1.807
1.789
Ms
N
Ph
O
N
Ts
5a
0.99
1.98
3.02
4.06
1.43
2.03
0.98
2.00
0.99
2.01
2.01
3.04
3.00
3.03
9.5 9.0 8.5 8.0 7.5 7.0 6.5 6.0 5.5 5.0 4.5 4.0 3.5 3.0 2.5 2.0 1.5 1.0 0.5 0.0 -0.5
f1 (ppm)

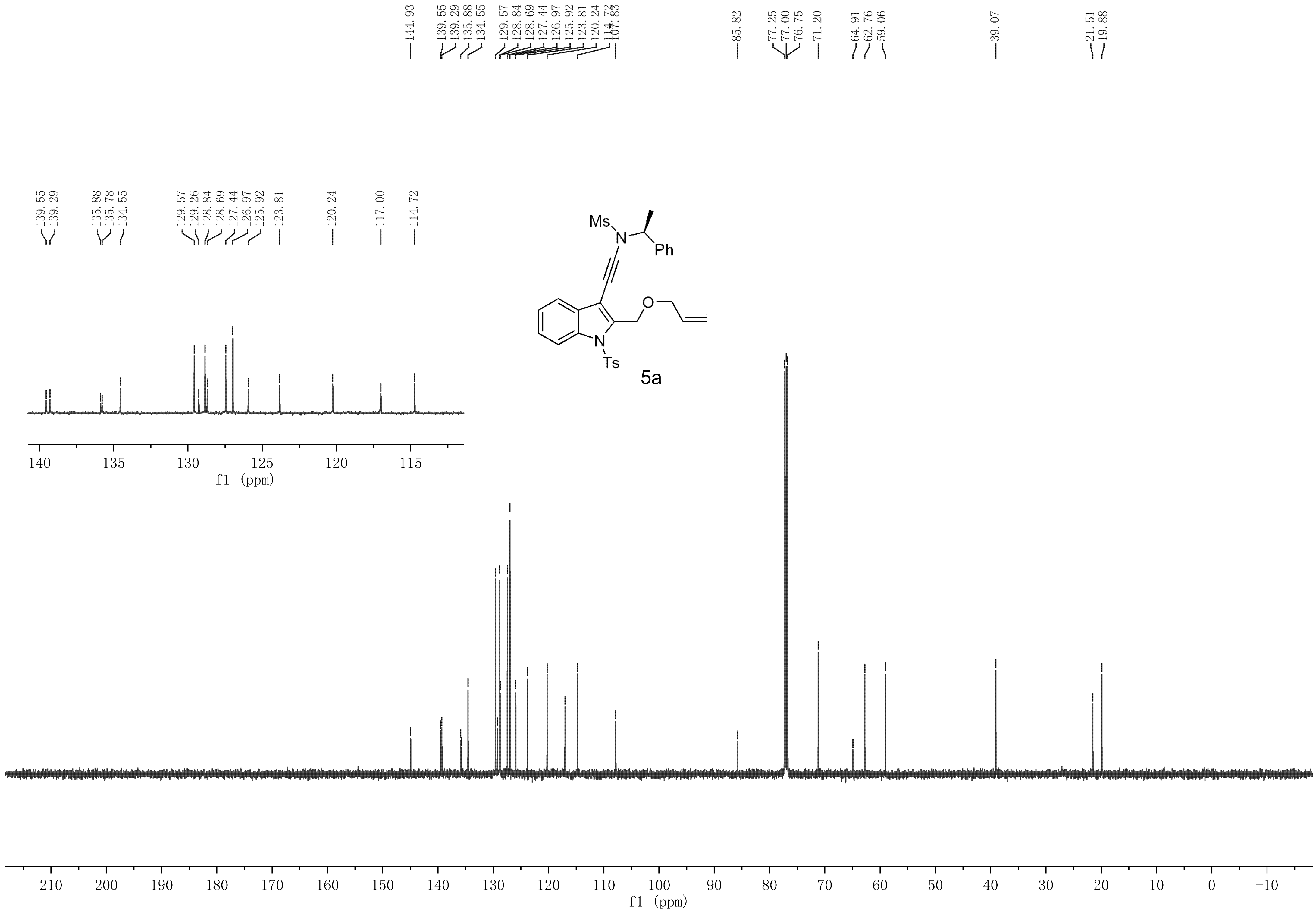
144.93
139.55
139.29
135.88
134.55
129.57
128.84
128.69
127.44
126.97
125.92
123.81
120.24
114.72
107.83
85.82
77.25
77.00
76.75
71.20
64.91
62.76
59.06
39.07
21.51
19.88
139.55
139.29
135.88
135.78
134.55
129.57
129.26
128.84
128.69
127.44
126.97
125.92
123.81
120.24
117.00
114.72
Ms
N
Ph
O
N
Ts
5a
140 135 130 125 120 115
f1 (ppm)
210 200 190 180 170 160 150 140 130 120 110 100 90 80 70 60 50 40 30 20 10 0 -10
f1 (ppm)

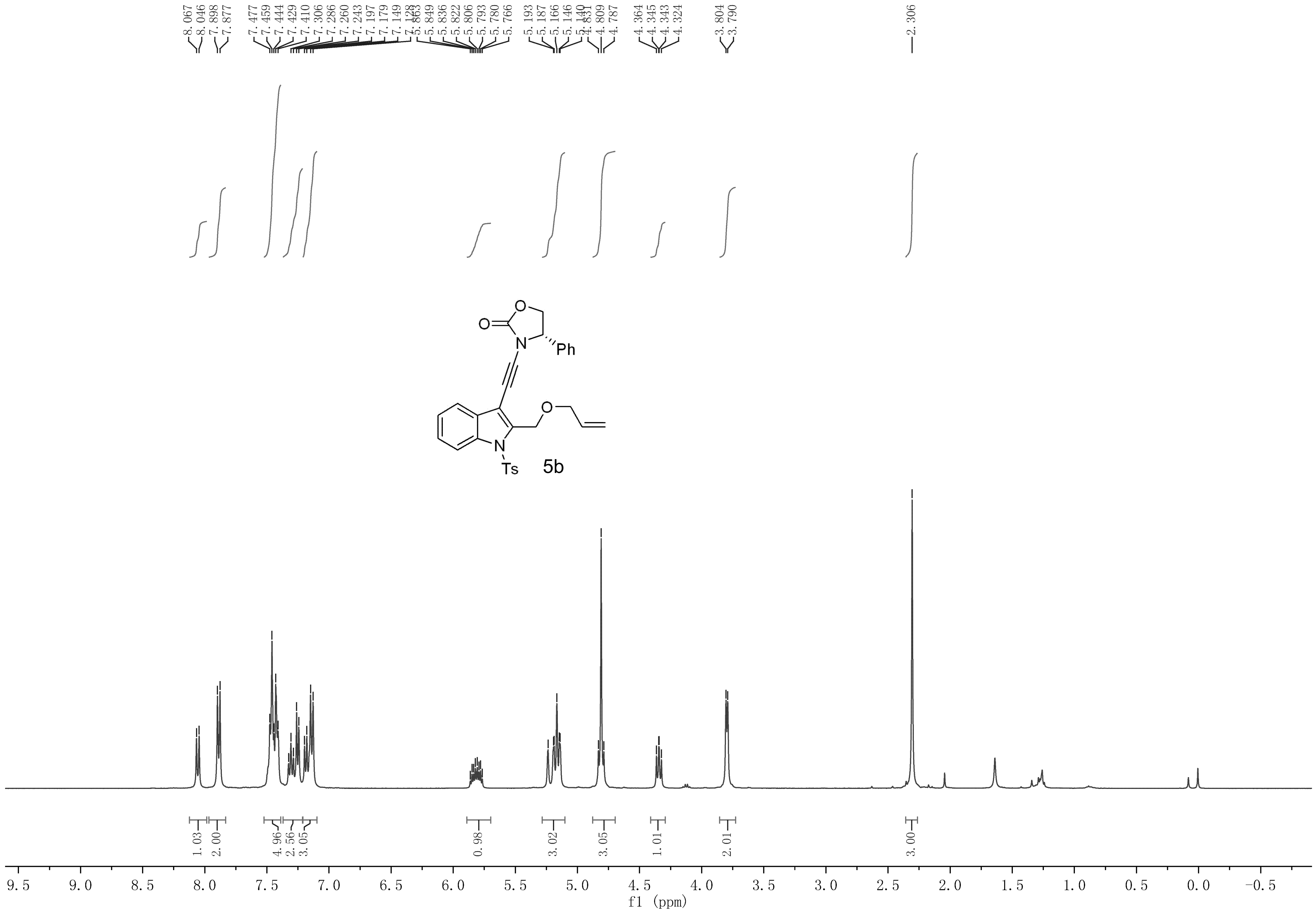
8.067
8.046
7.898
7.877
7.477
7.459
7.444
7.429
7.410
7.306
7.286
7.260
7.243
7.197
7.179
7.149
7.128
5.863
5.849
5.836
5.822
5.806
5.793
5.780
5.766
5.193
5.187
5.166
5.146
5.140
4.831
4.809
4.787
4.364
4.345
4.343
4.324
3.804
3.790
2.306
O
O
N
Ph
O
N
Ts
5b
1.03
2.00
4.96
2.56
3.05
0.98
3.02
3.05
1.01
2.01
3.00
9.5 9.0 8.5 8.0 7.5 7.0 6.5 6.0 5.5 5.0 4.5 4.0 3.5 3.0 2.5 2.0 1.5 1.0 0.5 0.0 -0.5
f1 (ppm)

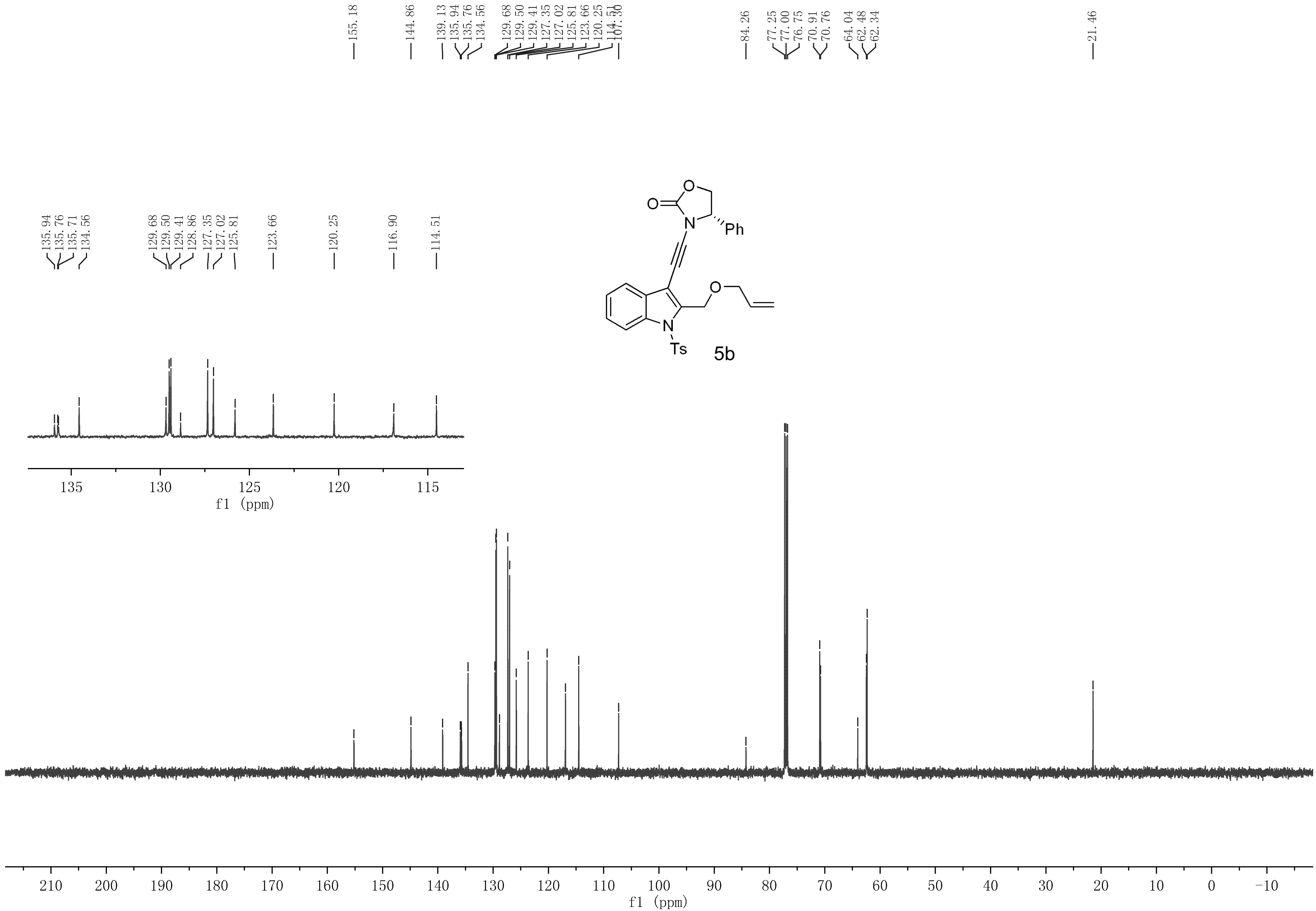
155.18
144.86
139.13
135.94
135.76
134.56
129.68
129.50
129.41
127.35
127.02
125.81
123.66
120.25
114.51
107.30
84.26
77.25
77.00
76.75
70.91
70.76
64.04
62.48
62.34
21.46
135.94
135.76
135.71
134.56
129.68
129.50
129.41
128.86
127.35
127.02
125.81
123.66
120.25
116.90
114.51
f1 (ppm)
O
N
Ph
Ts
5b
f1 (ppm)

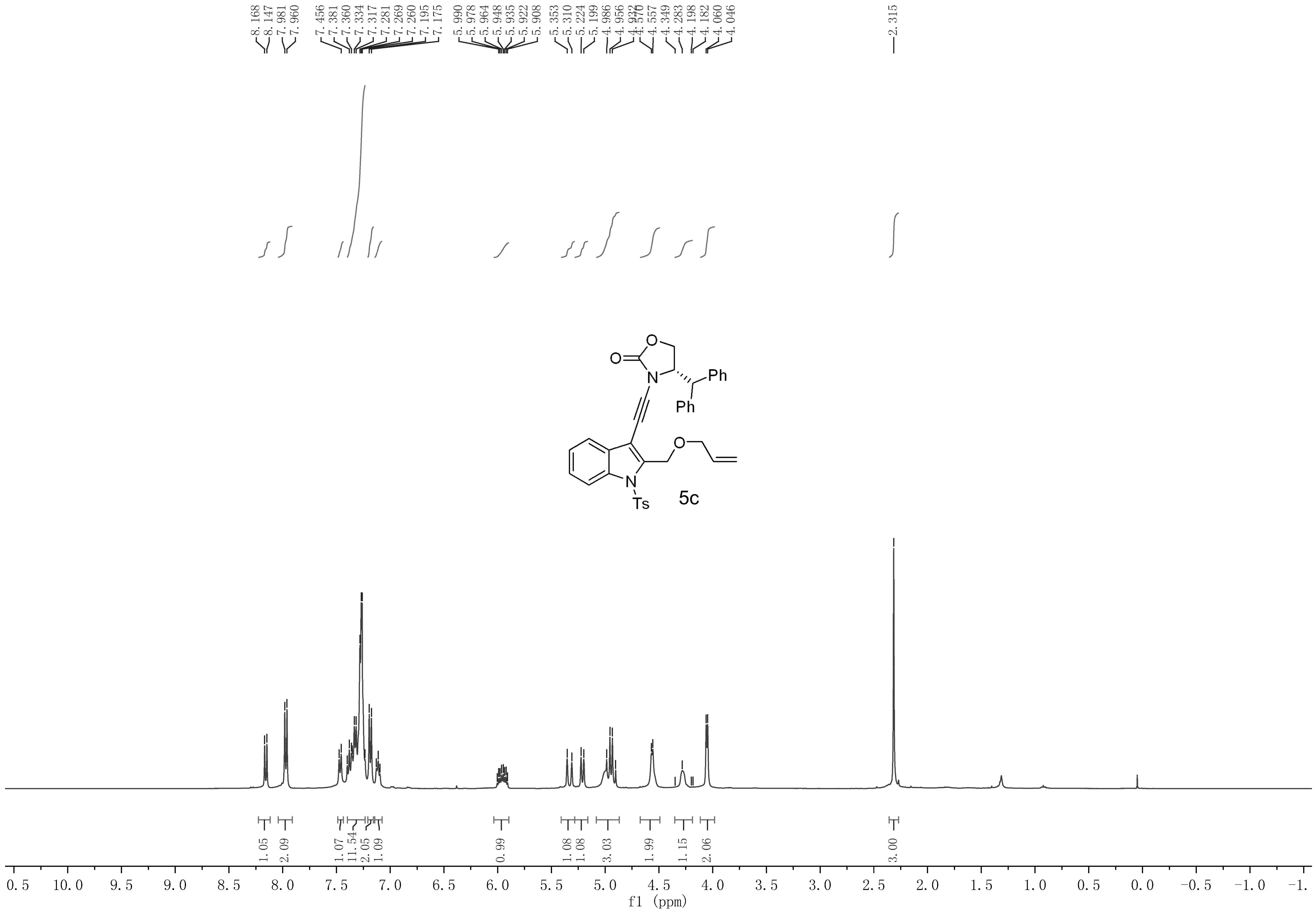
8.168
8.147
7.981
7.960
7.456
7.381
7.360
7.334
7.317
7.281
7.269
7.260
7.195
7.175
5.990
5.978
5.964
5.948
5.935
5.922
5.908
5.353
5.310
5.224
5.199
4.986
4.956
4.932
4.570
4.557
4.349
4.283
4.198
4.182
4.060
4.046
2.315
O
O
N
Ph
Ph
O
N
Ts
5c
1.05
2.09
1.07
11.54
2.05
1.09
0.99
1.08
1.08
3.03
1.99
1.15
2.06
3.00
f1 (ppm)

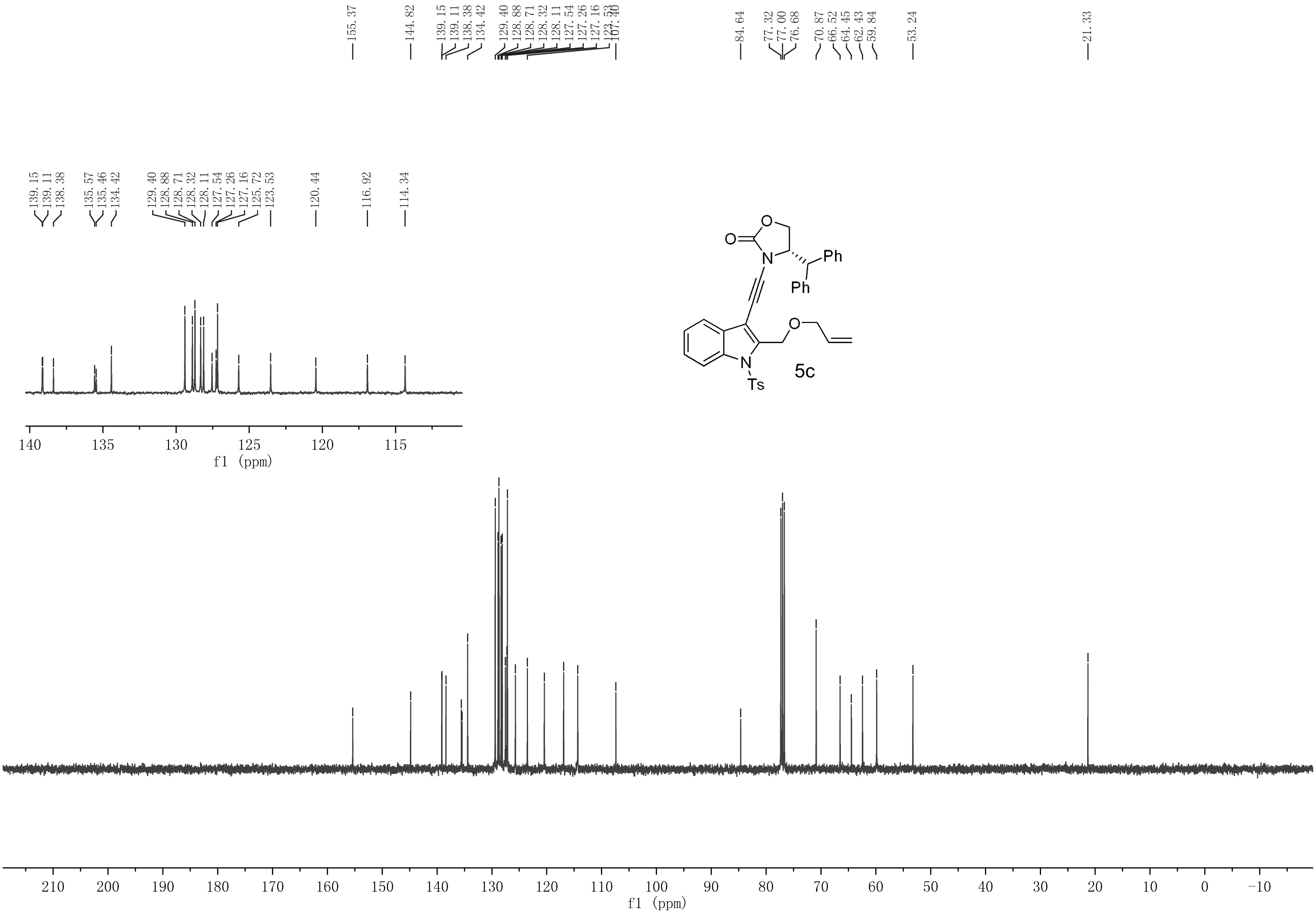
155.37
144.82
139.15
139.11
138.38
134.42
129.40
128.88
128.71
128.32
128.11
127.54
127.26
127.16
123.53
107.40
84.64
77.32
77.00
76.68
70.87
66.52
64.45
62.43
59.84
53.24
21.33
139.15
139.11
138.38
135.57
135.46
134.42
129.40
128.88
128.71
128.32
128.11
127.54
127.26
127.16
125.72
123.53
120.44
116.92
114.34
140 135 130 125 120 115
f1 (ppm)
210 200 190 180 170 160 150 140 130 120 110 100 90 80 70 60 50 40 30 20 10 0 −10
f1 (ppm)
O
O
N
Ph
Ph
O
N
Ts

5c

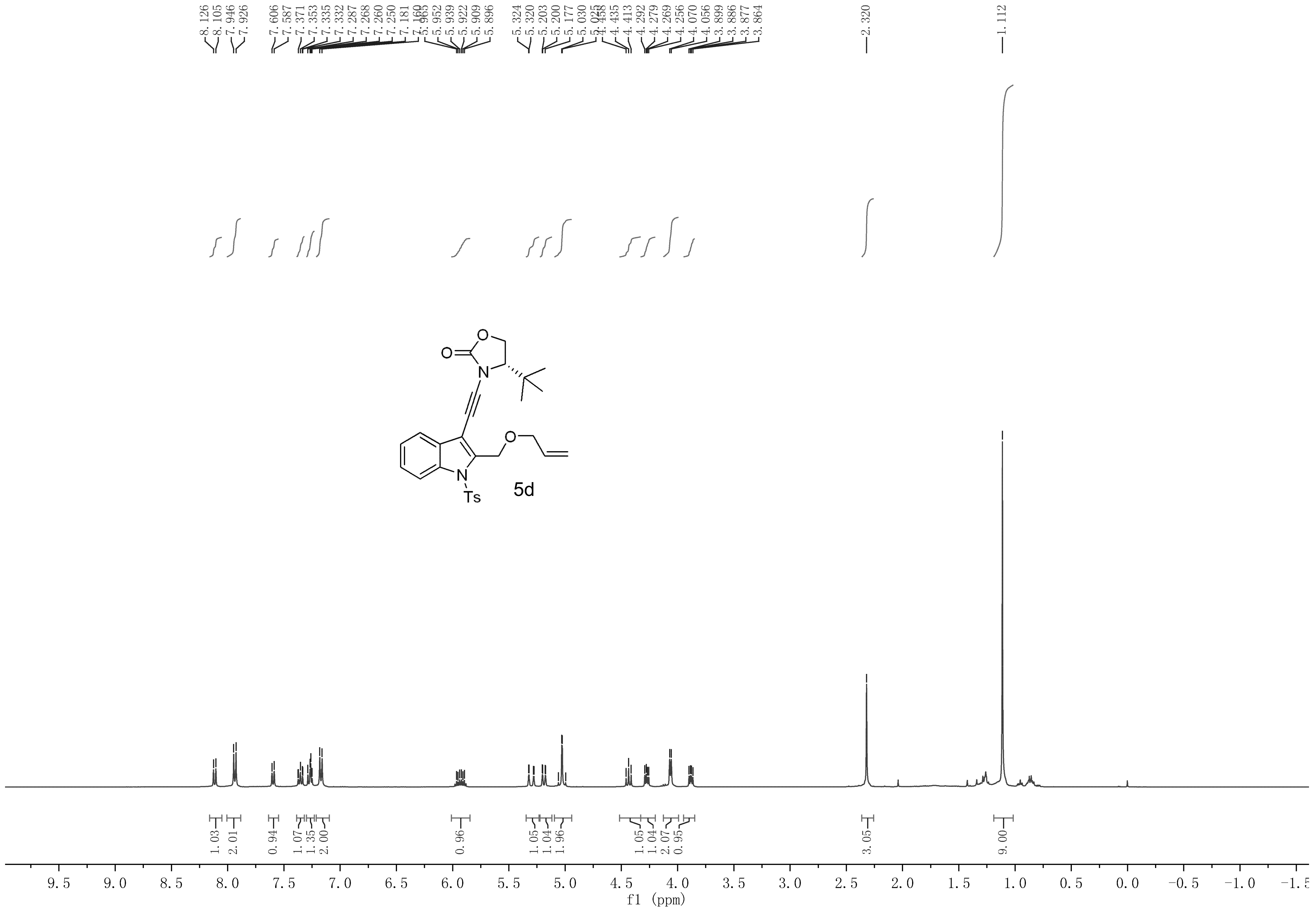
8.126
8.105
7.946
7.926
7.606
7.587
7.371
7.353
7.335
7.332
7.287
7.268
7.260
7.250
7.181
7.160
5.965
5.952
5.939
5.922
5.909
5.896
5.324
5.320
5.203
5.200
5.177
5.030
5.025
4.458
4.435
4.413
4.292
4.279
4.269
4.256
4.070
4.056
3.899
3.886
3.877
3.864
2.320
1.112
O
O
N
N
O
Ts
5d
1.03
2.01
0.94
1.07
1.35
2.00
0.96
1.05
1.04
1.96
1.05
1.04
2.07
0.95
3.05
9.00
f1 (ppm)

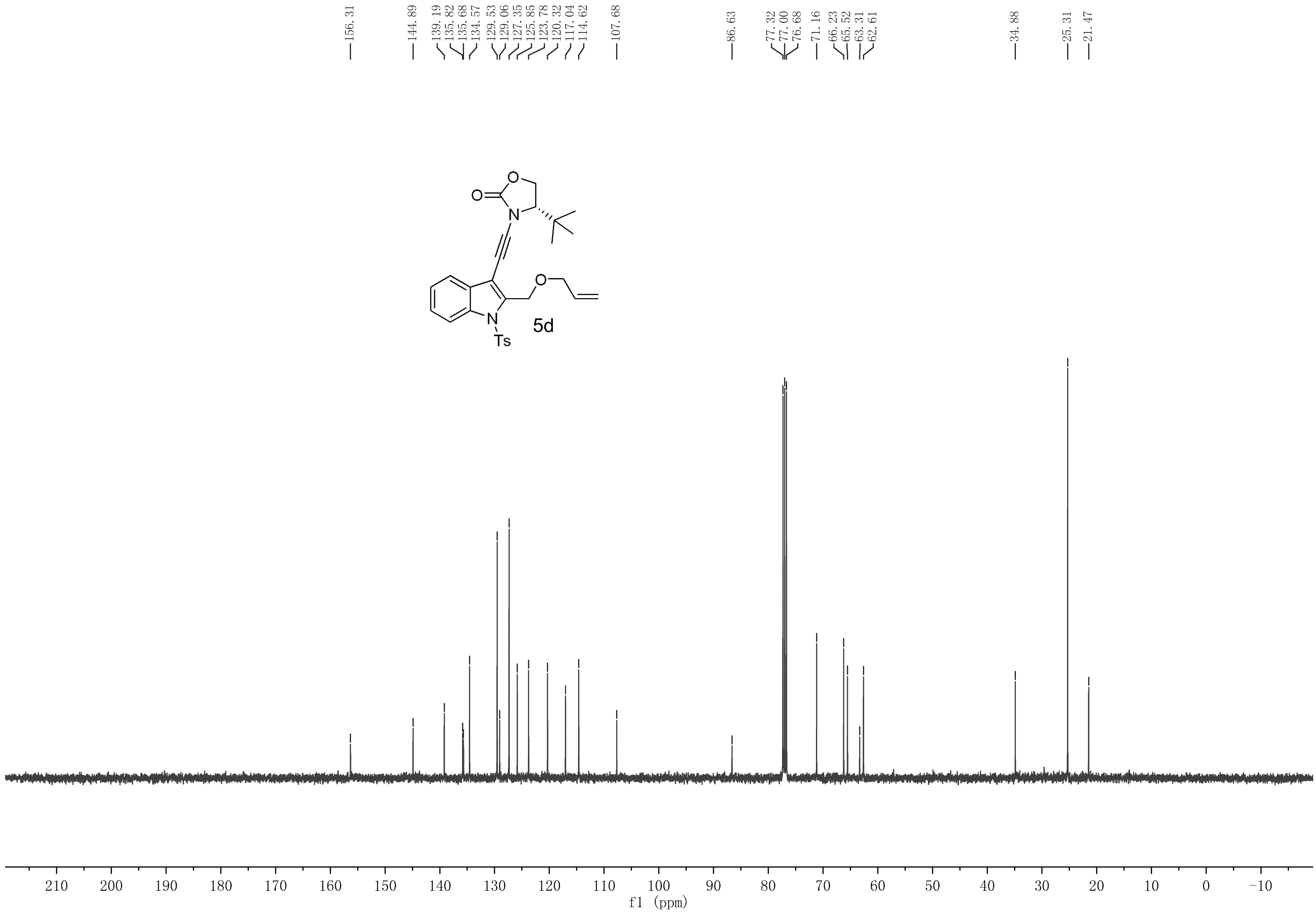
156.31
144.89
139.19
135.82
135.68
134.57
129.53
129.06
127.35
125.85
123.78
120.32
117.04
114.62
107.68
86.63
77.32
77.00
76.68
71.16
66.23
65.52
63.31
62.61
34.88
25.31
21.47
O
O
N
N
Ts
5d
210 200 190 180 170 160 150 140 130 120 110 100 90 80 70 60 50 40 30 20 10 0 −10
f1 (ppm)

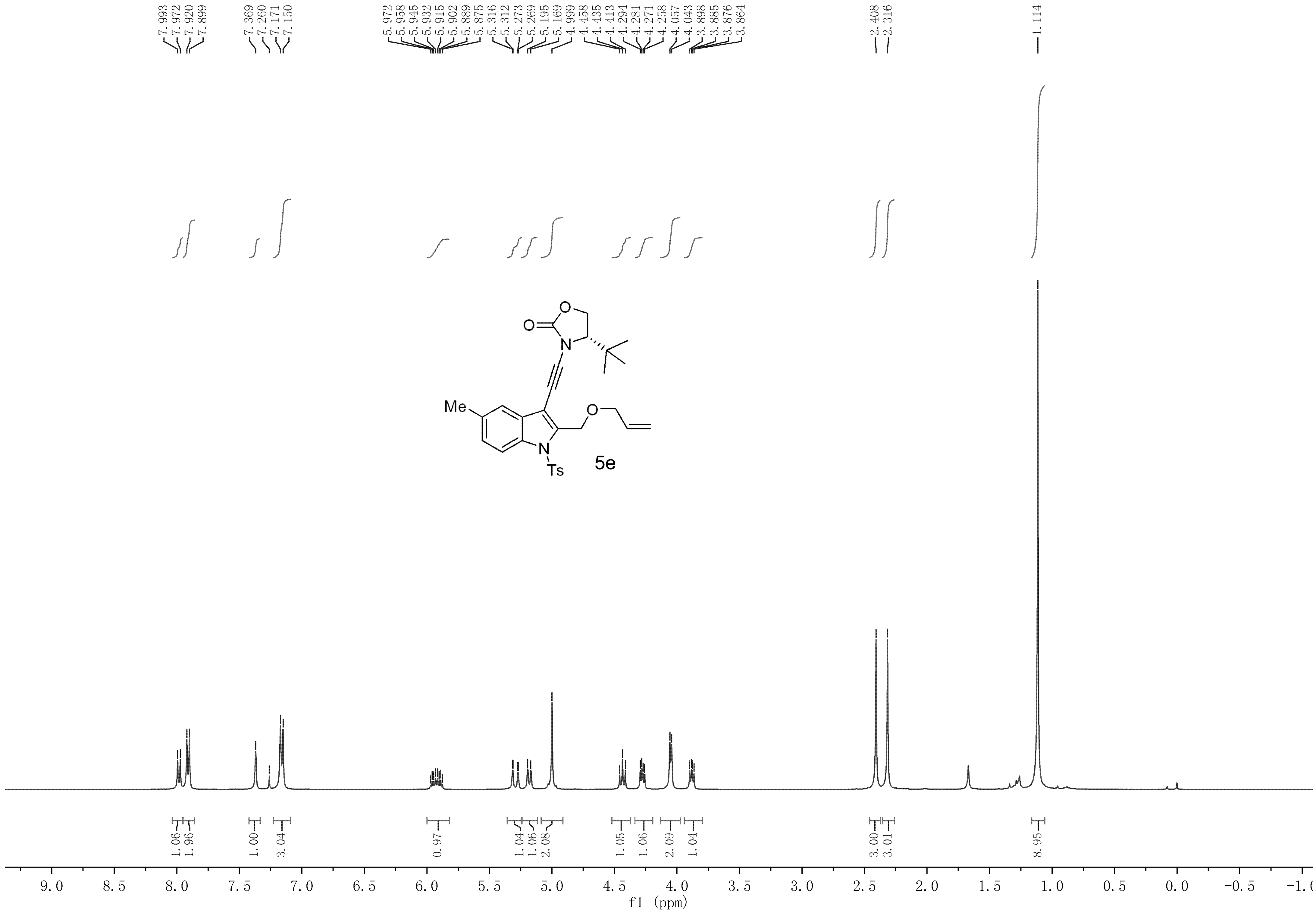
7.993
7.972
7.920
7.899
7.369
7.260
7.171
7.150
5.972
5.958
5.945
5.932
5.915
5.902
5.889
5.875
5.316
5.312
5.273
5.269
5.195
5.169
4.999
4.458
4.435
4.413
4.294
4.281
4.271
4.258
4.057
4.043
3.898
3.885
3.876
3.864
2.408
2.316
1.114
O
O
N
Me
O
N
Ts
5e
1.06
1.96
1.00
3.04
0.97
1.04
1.06
2.08
1.05
1.06
2.09
1.04
3.00
3.01
8.95
9.0
8.5
8.0
7.5
7.0
6.5
6.0
5.5
5.0
4.5
4.0
3.5
3.0
2.5
2.0
1.5
1.0
0.5
0.0
-0.5
f1 (ppm)

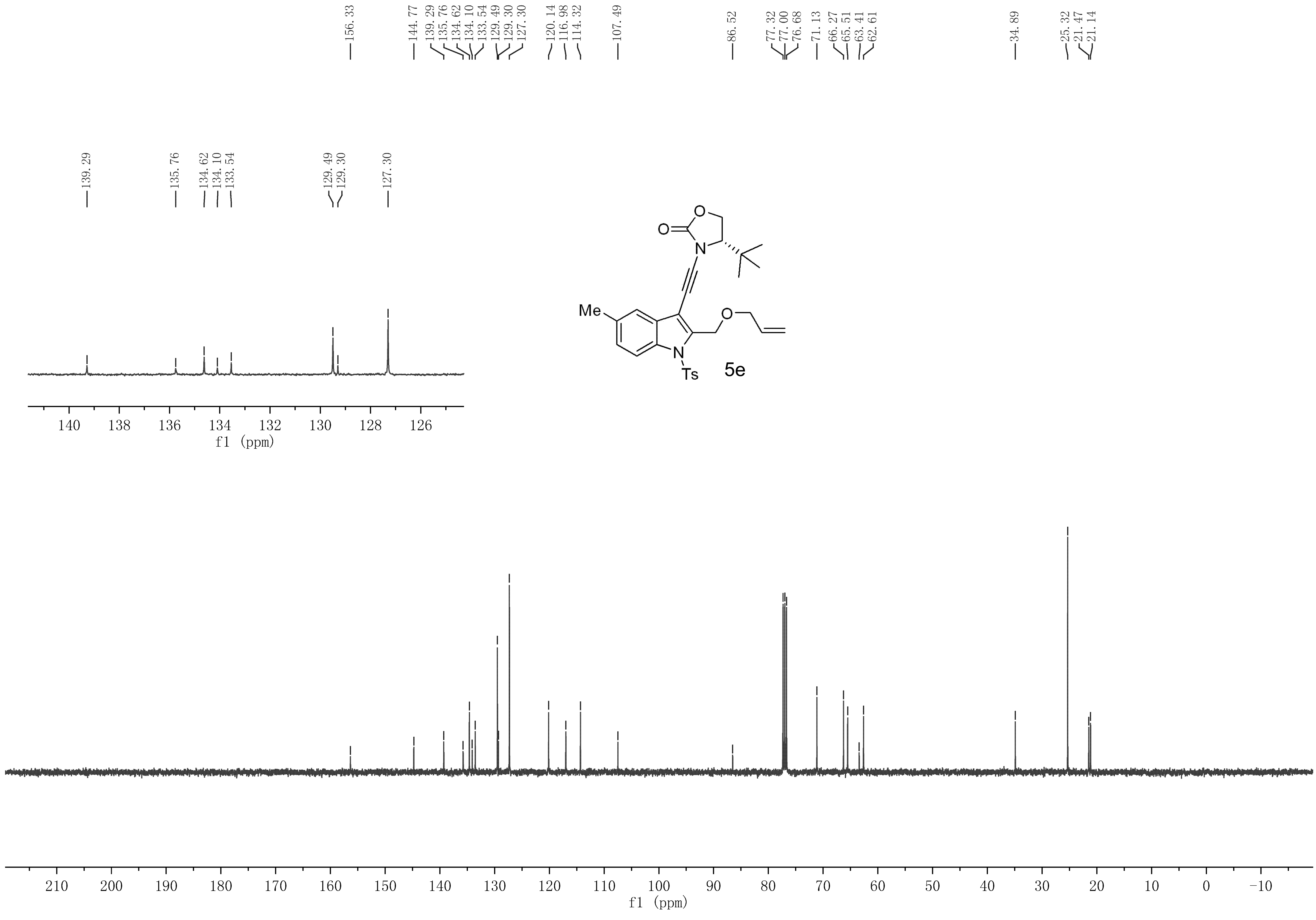

156.33
144.77
139.29
135.76
134.62
134.10
133.54
129.49
129.30
127.30
120.14
116.98
114.32
107.49
86.52
77.32
77.00
76.68
71.13
66.27
65.51
63.41
62.61
34.89
25.32
21.47
21.14
139.29
135.76
134.62
134.10
133.54
129.49
129.30
127.30
140 138 136 134 132 130 128 126
f1 (ppm)
O
O
N
Me
O
N
Ts
5e
210 200 190 180 170 160 150 140 130 120 110 100 90 80 70 60 50 40 30 20 10 0 -10
f1 (ppm)

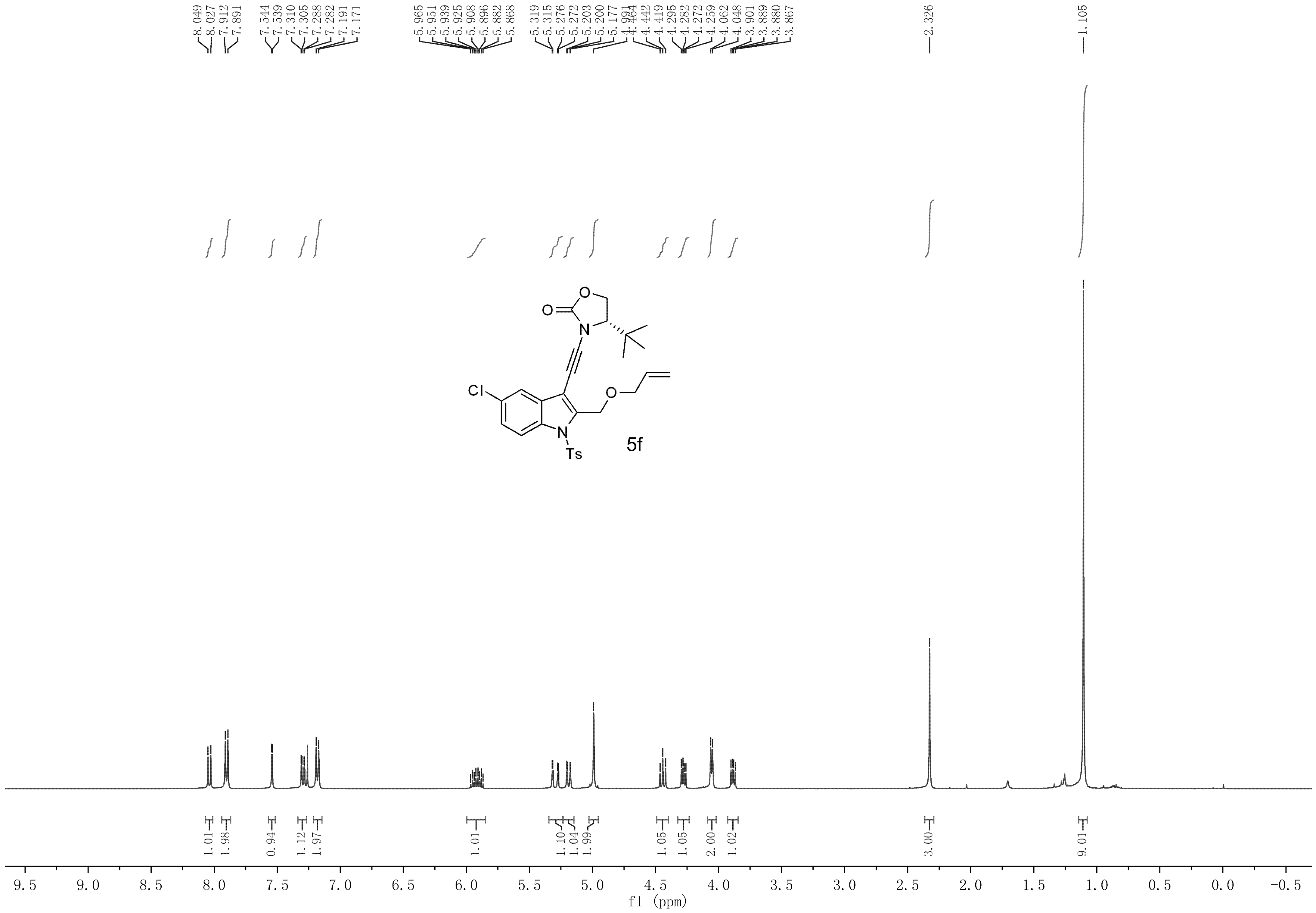

8.049
8.027
7.912
7.891
7.544
7.539
7.310
7.305
7.288
7.282
7.191
7.171
5.965
5.951
5.939
5.925
5.908
5.896
5.882
5.868
5.319
5.315
5.276
5.272
5.203
5.200
5.177
4.991
4.464
4.442
4.419
4.295
4.282
4.272
4.259
4.062
4.048
3.901
3.889
3.880
3.867
2.326
1.105
O
O
N
Cl
O
N
Ts
5f
1.01
1.98
0.94
1.12
1.97
1.01
1.10
1.04
1.99
1.05
1.05
2.00
1.02
3.00
9.01
9.5
9.0
8.5
8.0
7.5
7.0
6.5
6.0
5.5
5.0
4.5
4.0
3.5
3.0
2.5
2.0
1.5
1.0
0.5
0.0
-0.5
f1 (ppm)

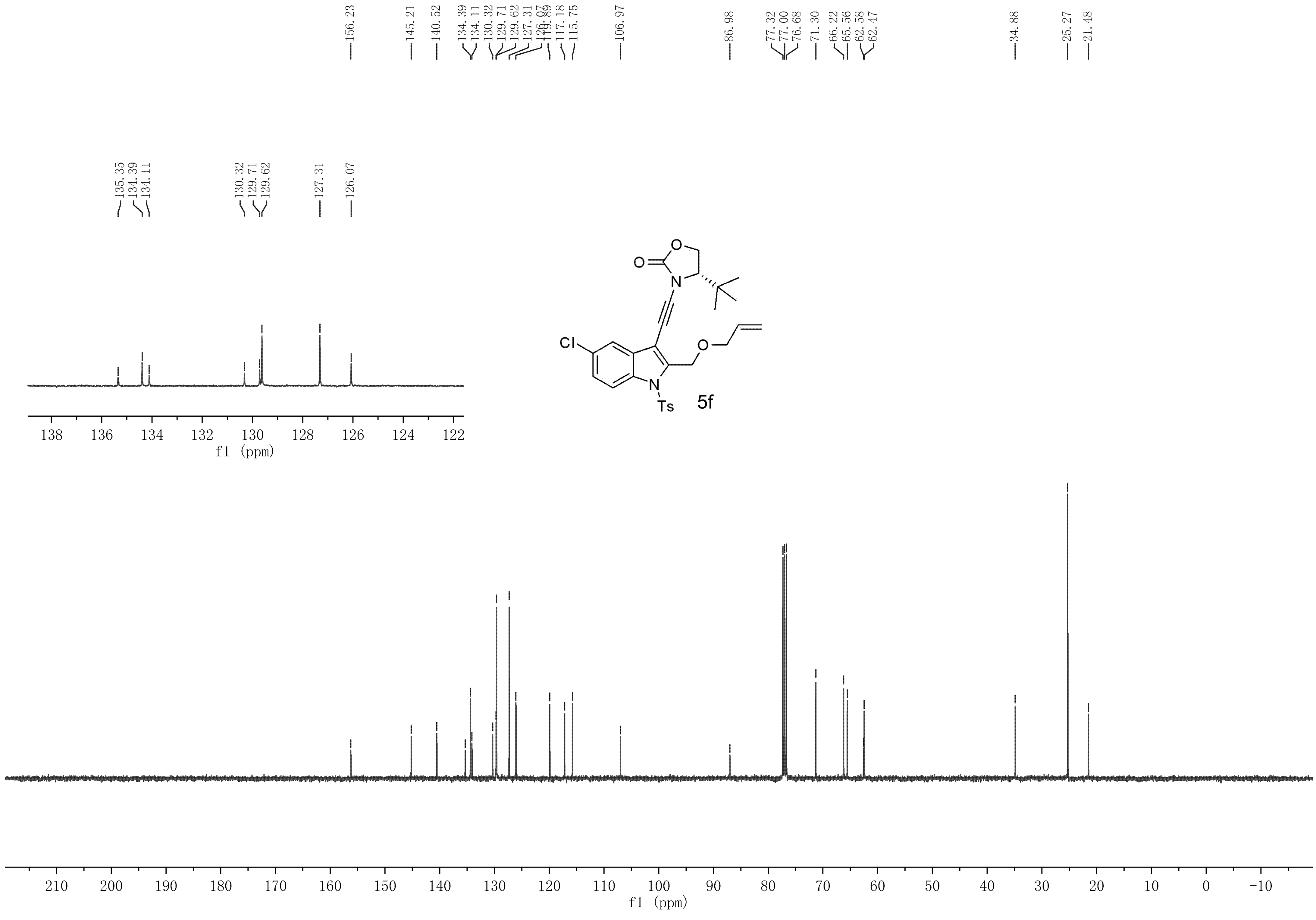
156.23
145.21
140.52
134.39
134.11
130.32
129.71
129.62
127.31
126.07
119.89
117.18
115.75
106.97
86.98
77.32
77.00
76.68
71.30
66.22
65.56
62.58
62.47
34.88
25.27
21.48
135.35
134.39
134.11
130.32
129.71
129.62
127.31
126.07
138
136
134
132
130
128
126
124
122
f1 (ppm)
O
N
Cl
Ts
5f
210
200
190
180
170
160
150
140
130
120
110
100
90
80
70
60
50
40
30
20
10
0
−10
f1 (ppm)

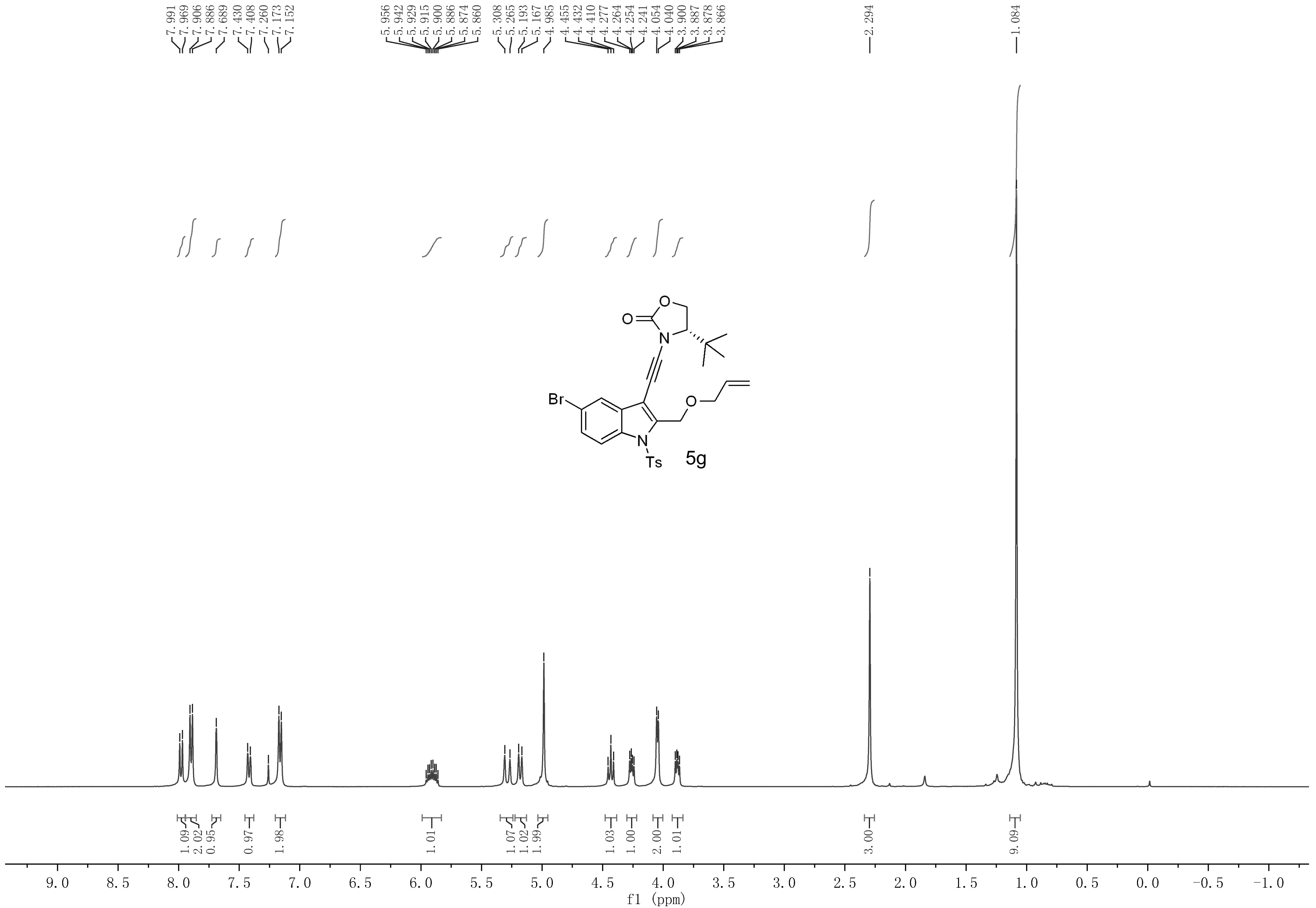
7.991
7.969
7.906
7.886
7.689
7.430
7.408
7.260
7.173
7.152
5.956
5.942
5.929
5.915
5.900
5.886
5.874
5.860
5.308
5.265
5.193
5.167
4.985
4.455
4.432
4.410
4.277
4.264
4.254
4.241
4.054
4.040
3.900
3.887
3.878
3.866
2.294
1.084
O
O
N
Br
O
N
Ts
5g
1.09
2.02
0.95
0.97
1.98
1.01
1.07
1.02
1.99
1.03
1.00
2.00
1.01
3.00
9.09
9.0
8.5
8.0
7.5
7.0
6.5
6.0
5.5
5.0
4.5
4.0
3.5
3.0
2.5
2.0
1.5
1.0
0.5
0.0
-0.5
-1.0
f1 (ppm)

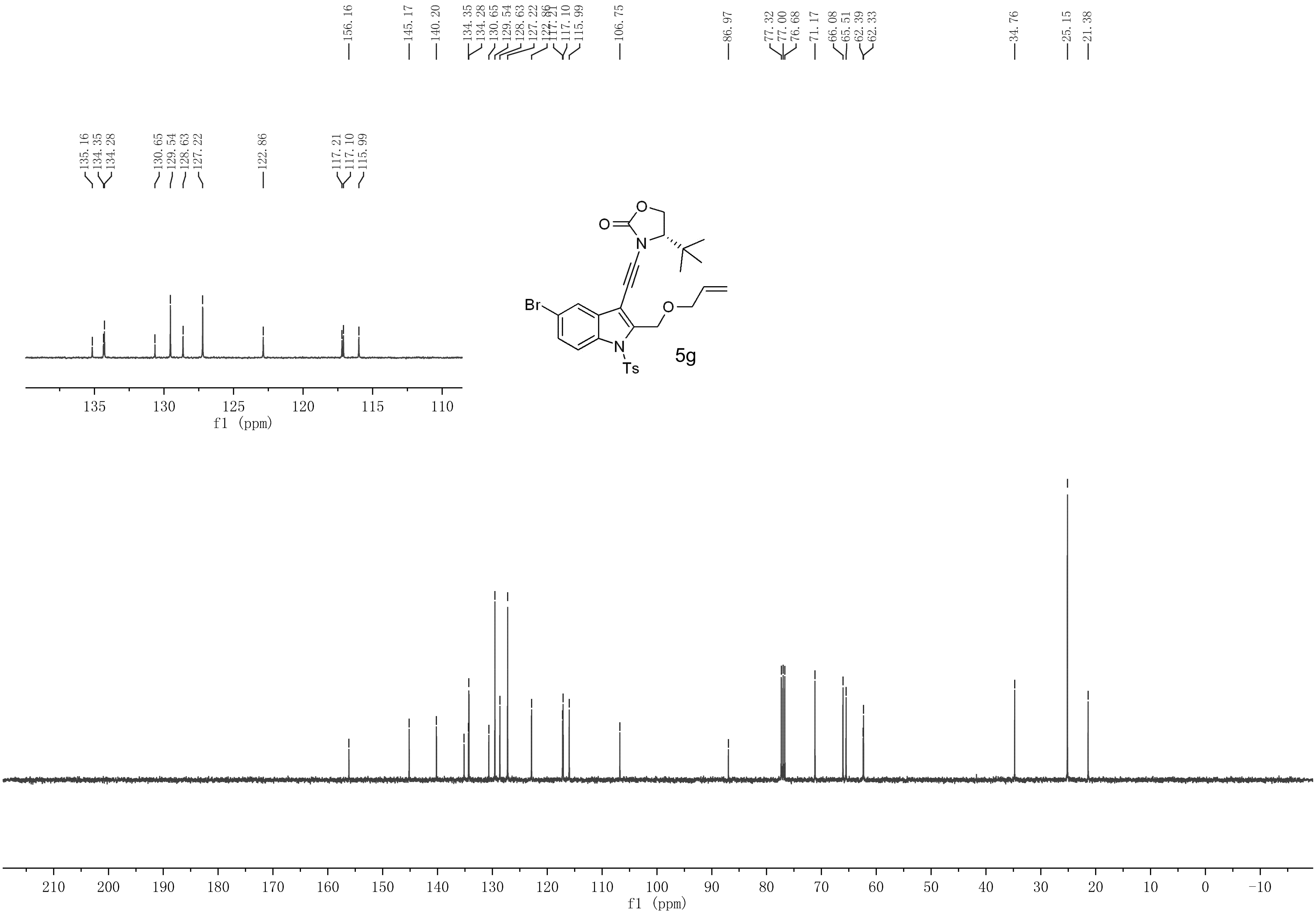
156.16
145.17
140.20
134.35
134.28
130.65
129.54
128.63
127.22
122.86
117.21
117.10
115.99
106.75
86.97
77.32
77.00
76.68
71.17
66.08
65.51
62.39
62.33
34.76
25.15
21.38
135.16
134.35
134.28
130.65
129.54
128.63
127.22
122.86
117.21
117.10
115.99
135 130 125 120 115 110
f1 (ppm)
O
O
N
Br
O
N
Ts
5g
210 200 190 180 170 160 150 140 130 120 110 100 90 80 70 60 50 40 30 20 10 0 −10
f1 (ppm)

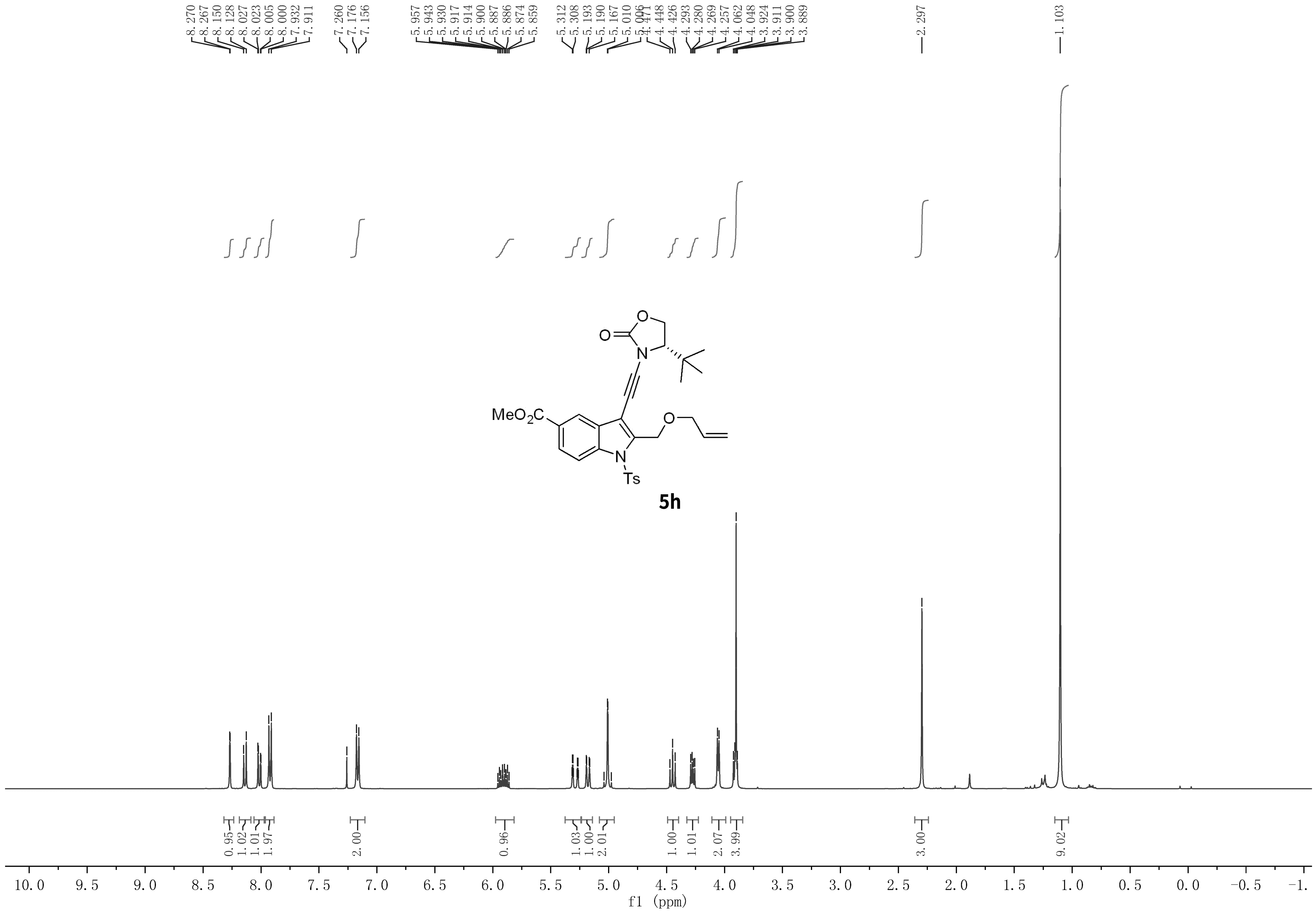

8.270
8.267
8.150
8.128
8.027
8.023
8.005
8.000
7.932
7.911
7.260
7.176
7.156
5.957
5.943
5.930
5.917
5.914
5.900
5.887
5.886
5.874
5.859
5.312
5.308
5.193
5.190
5.167
5.010
5.006
4.471
4.448
4.426
4.293
4.280
4.269
4.257
4.062
4.048
3.924
3.911
3.900
3.889
2.297
1.103
MeO2C
O
N
O
O
N
Ts
0.95
1.02
1.01
1.97
2.00
0.96
1.03
1.00
2.01
1.00
1.01
2.07
3.99
3.00
9.02
f1 (ppm)

5h

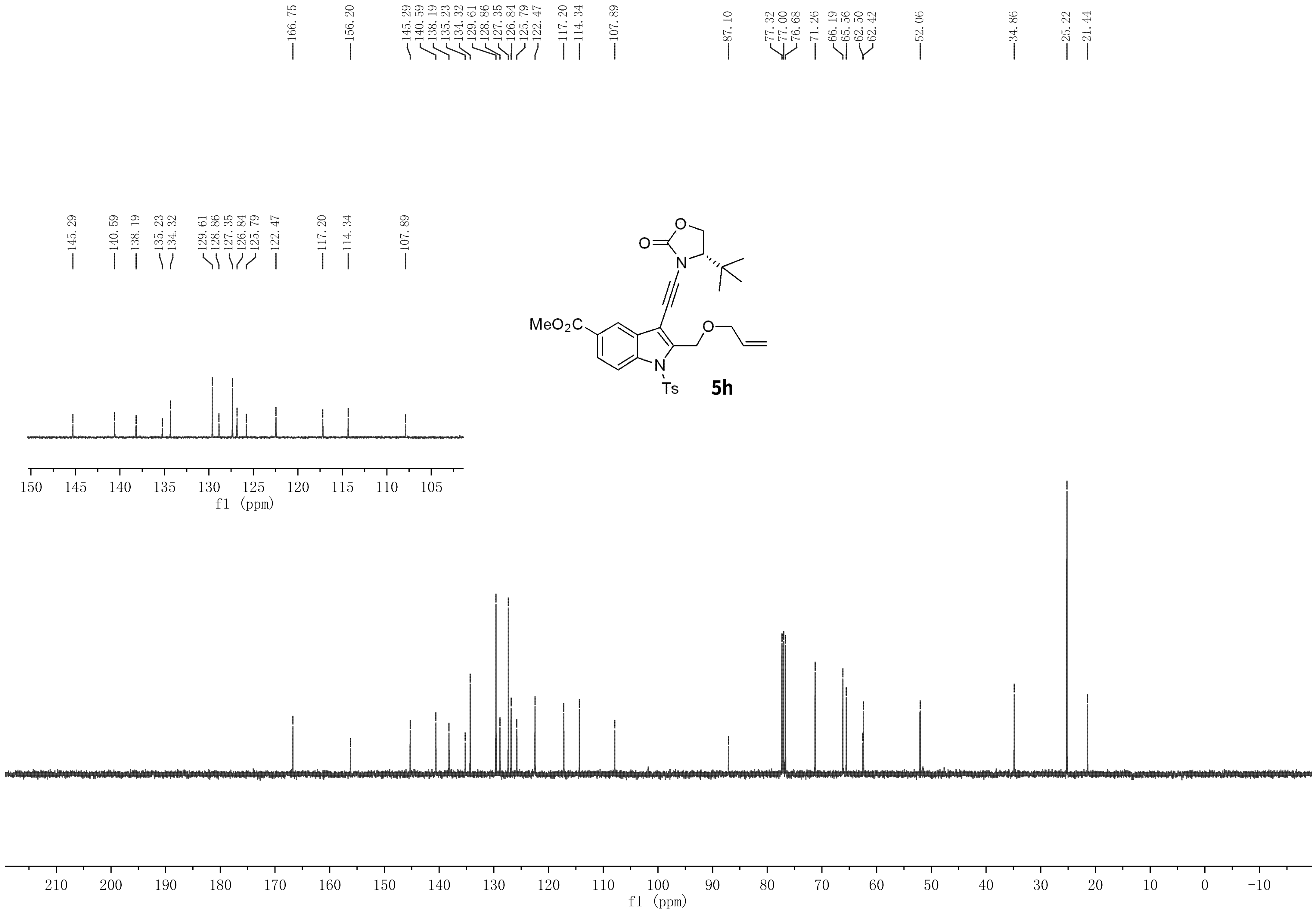

166.75
156.20
145.29
140.59
138.19
135.23
134.32
129.61
128.86
127.35
126.84
125.79
122.47
117.20
114.34
107.89
87.10
77.32
77.00
76.68
71.26
66.19
65.56
62.50
62.42
52.06
34.86
25.22
21.44
MeO2C
O
N
Ts
5h
f1 (ppm)

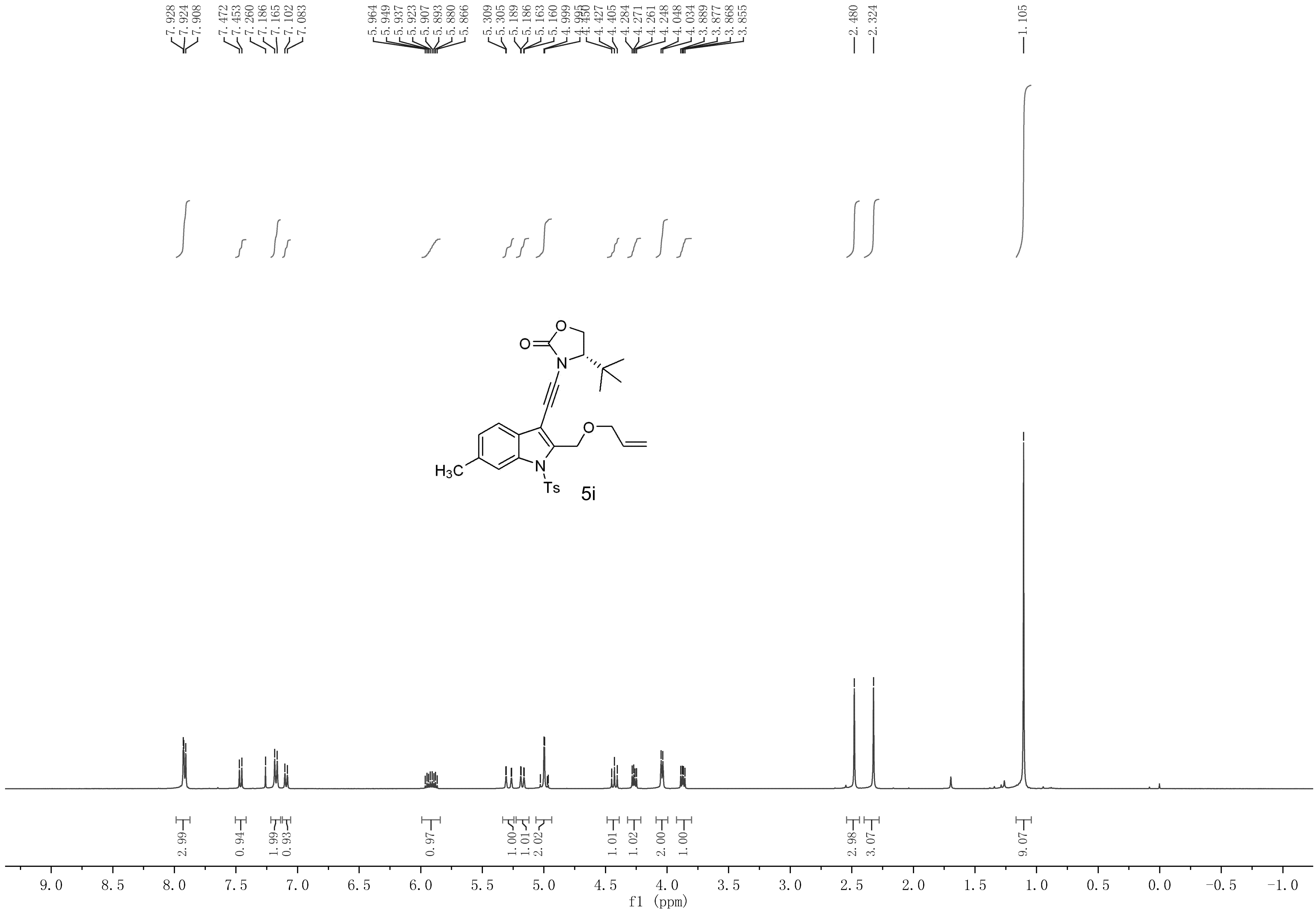
7.928
7.924
7.908
7.472
7.453
7.260
7.186
7.165
7.102
7.083
5.964
5.949
5.937
5.923
5.907
5.893
5.880
5.866
5.309
5.305
5.189
5.186
5.163
5.160
4.999
4.995
4.450
4.427
4.405
4.284
4.271
4.261
4.248
4.048
4.034
3.889
3.877
3.868
3.855
2.480
2.324
1.105
O
N
H3C
N
Ts
5i
2.99
0.94
1.99
0.93
0.97
1.00
1.01
2.02
1.01
1.02
2.00
1.00
2.98
3.07
9.07
9.0
8.5
8.0
7.5
7.0
6.5
6.0
5.5
5.0
4.5
4.0
3.5
3.0
2.5
2.0
1.5
1.0
0.5
0.0
-0.5
-1.0
f1 (ppm)

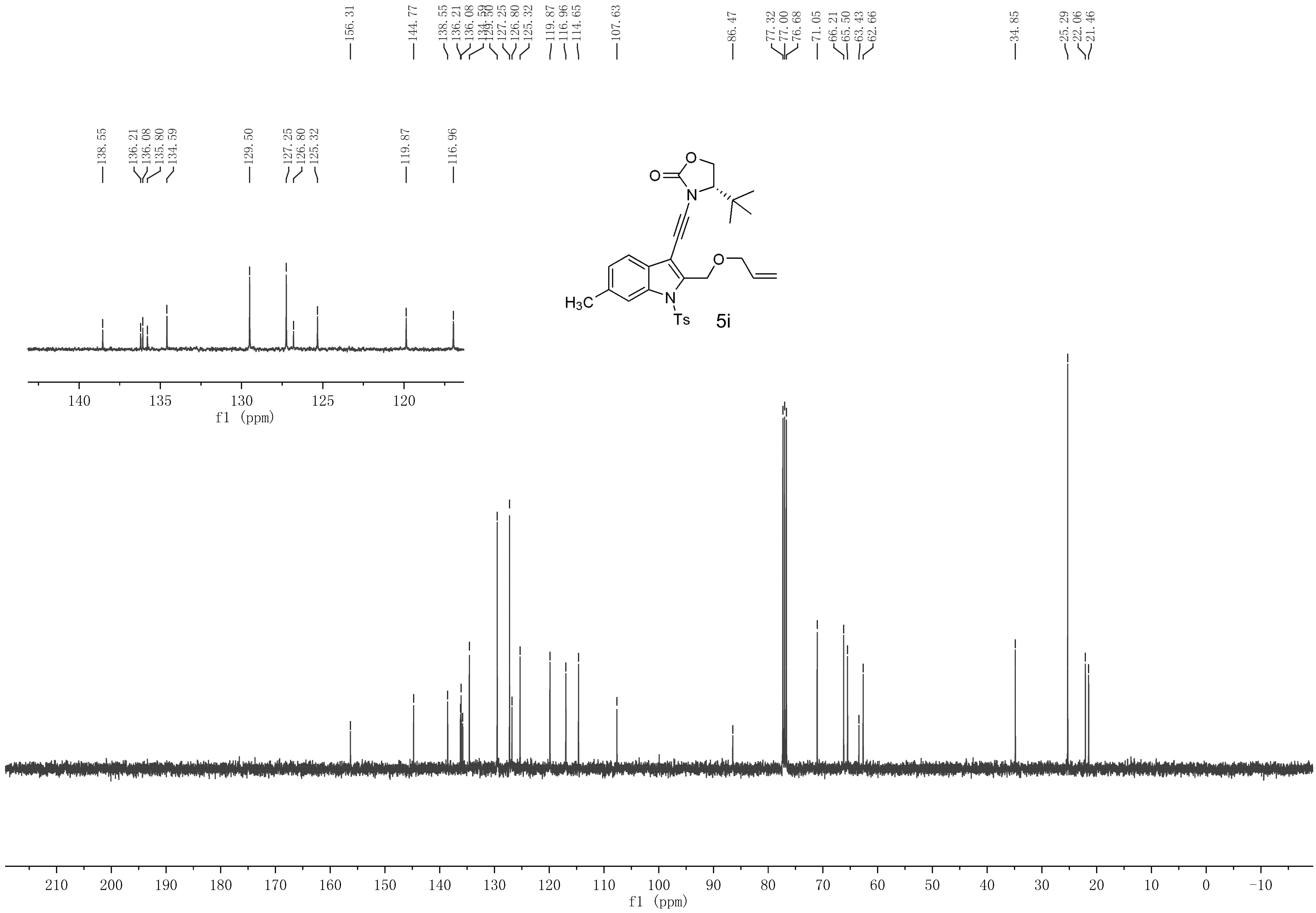
156.31
144.77
138.55
136.21
136.08
134.59
129.50
127.25
126.80
125.32
119.87
116.96
114.65
107.63
86.47
77.32
77.00
76.68
71.05
66.21
65.50
63.43
62.66
34.85
25.29
22.06
21.46
138.55
136.21
136.08
135.80
134.59
129.50
127.25
126.80
125.32
119.87
116.96
140 135 130 125 120
f1 (ppm)
O
O
N
O
H3C
N
Ts
5i
210 200 190 180 170 160 150 140 130 120 110 100 90 80 70 60 50 40 30 20 10 0 −10
f1 (ppm)

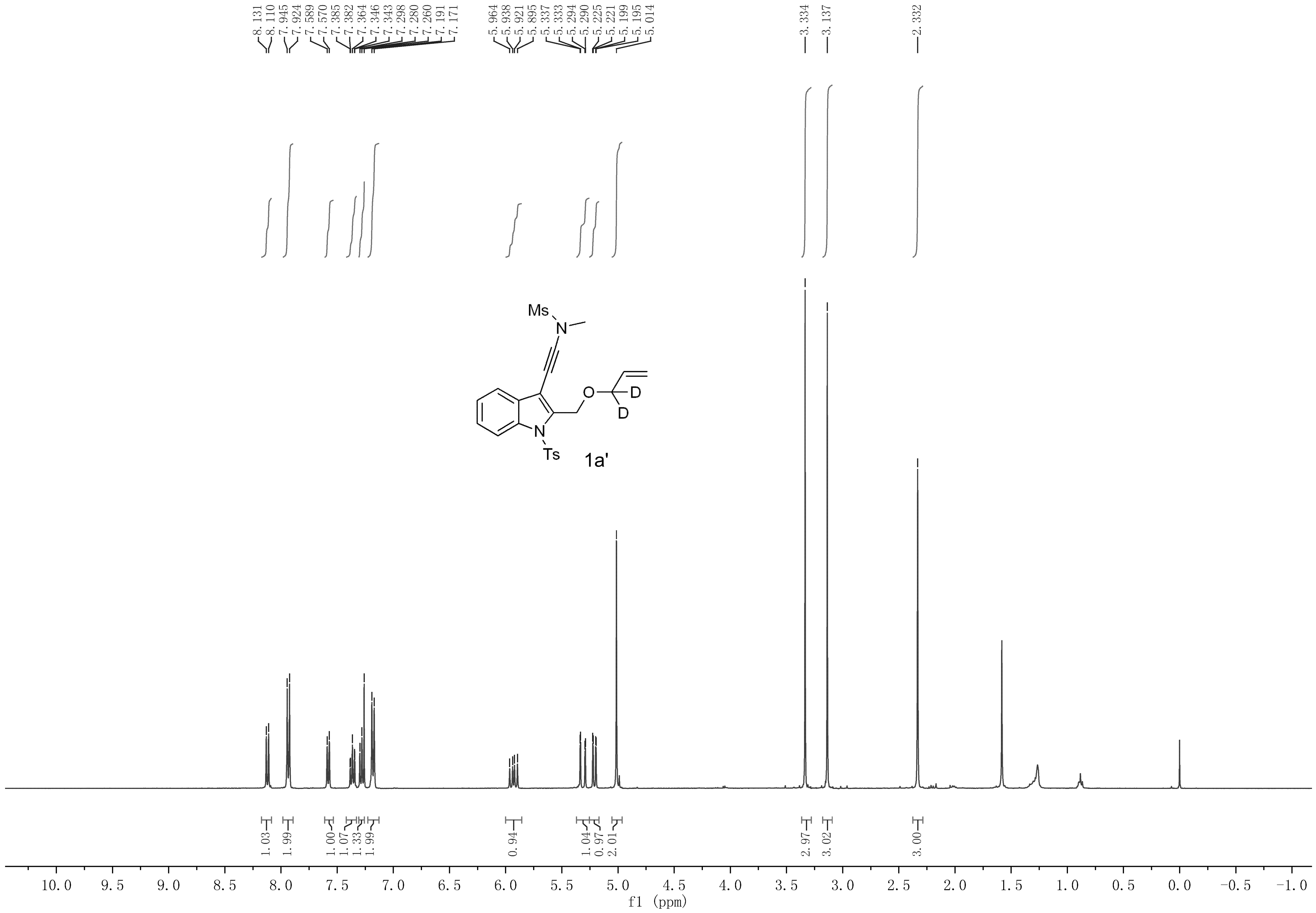
8.131
8.110
7.945
7.924
7.589
7.570
7.385
7.382
7.364
7.346
7.343
7.298
7.280
7.260
7.191
7.171
5.964
5.938
5.921
5.895
5.337
5.333
5.294
5.290
5.225
5.221
5.199
5.195
5.014
3.334
3.137
2.332
Ms
N
O
D
D
N
Ts
1a'
1.03
1.99
1.00
1.07
1.33
1.99
0.94
1.04
0.97
2.01
2.97
3.02
3.00
10.0 9.5 9.0 8.5 8.0 7.5 7.0 6.5 6.0 5.5 5.0 4.5 4.0 3.5 3.0 2.5 2.0 1.5 1.0 0.5 0.0 -0.5 -1.0
f1 (ppm)

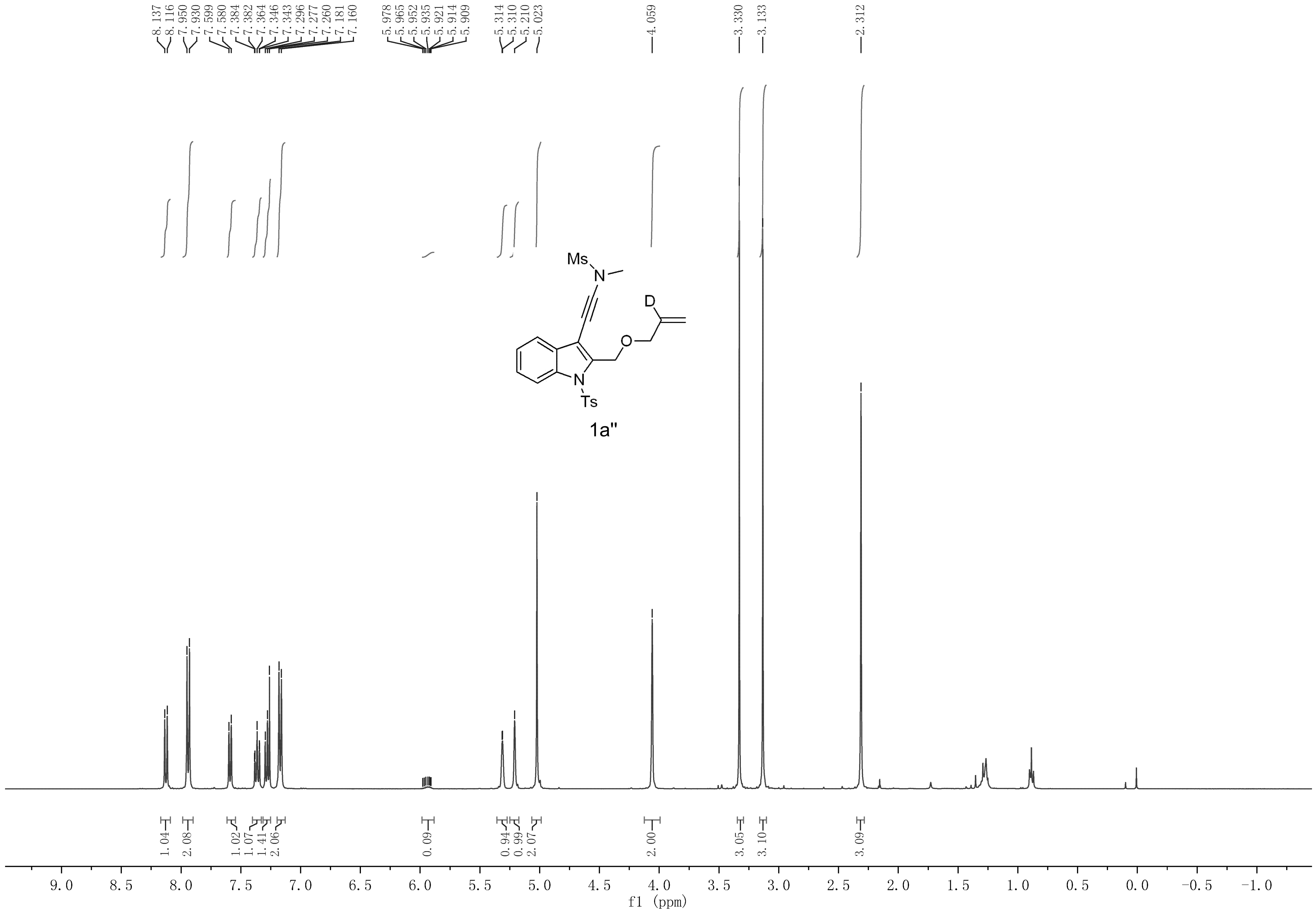
8.137
8.116
7.950
7.930
7.599
7.580
7.384
7.382
7.364
7.346
7.343
7.296
7.277
7.260
7.181
7.160
5.978
5.965
5.952
5.935
5.921
5.914
5.909
5.314
5.310
5.210
5.023
4.059
3.330
3.133
2.312
Ms
N
D
O
N
Ts
1a''
1.04
2.08
1.02
1.07
1.41
2.06
0.09
0.94
0.99
2.07
2.00
3.05
3.10
3.09
9.0
8.5
8.0
7.5
7.0
6.5
6.0
5.5
5.0
4.5
4.0
3.5
3.0
2.5
2.0
1.5
1.0
0.5
0.0
-0.5
-1.0
f1 (ppm)

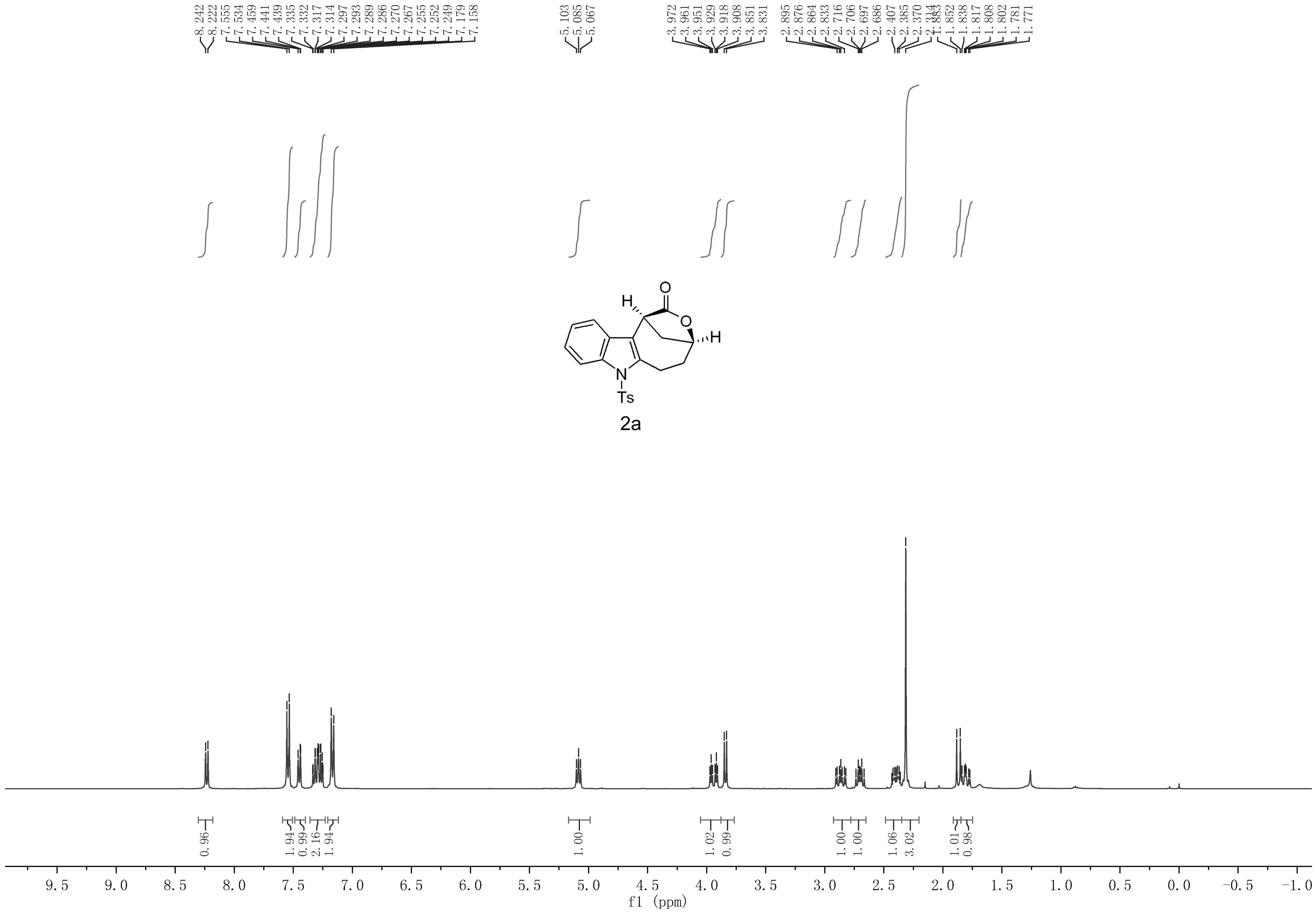
8.242
8.222
7.555
7.534
7.459
7.441
7.439
7.335
7.332
7.317
7.314
7.297
7.293
7.289
7.286
7.270
7.267
7.255
7.252
7.249
7.179
7.158
5.103
5.085
5.067
3.972
3.961
3.951
3.929
3.918
3.908
3.851
3.831
2.895
2.876
2.864
2.833
2.716
2.706
2.697
2.686
2.407
2.385
2.370
2.314
1.883
1.852
1.838
1.817
1.808
1.802
1.781
1.771
O
H
H
N
Ts
2a
0.96
1.94
0.99
2.16
1.94
1.00
1.02
0.99
1.00
1.00
1.06
3.02
1.01
0.98
9.5
9.0
8.5
8.0
7.5
7.0
6.5
6.0
5.5
5.0
4.5
4.0
3.5
3.0
2.5
2.0
1.5
1.0
0.5
0.0
−0.5
−1.0
f1 (ppm)

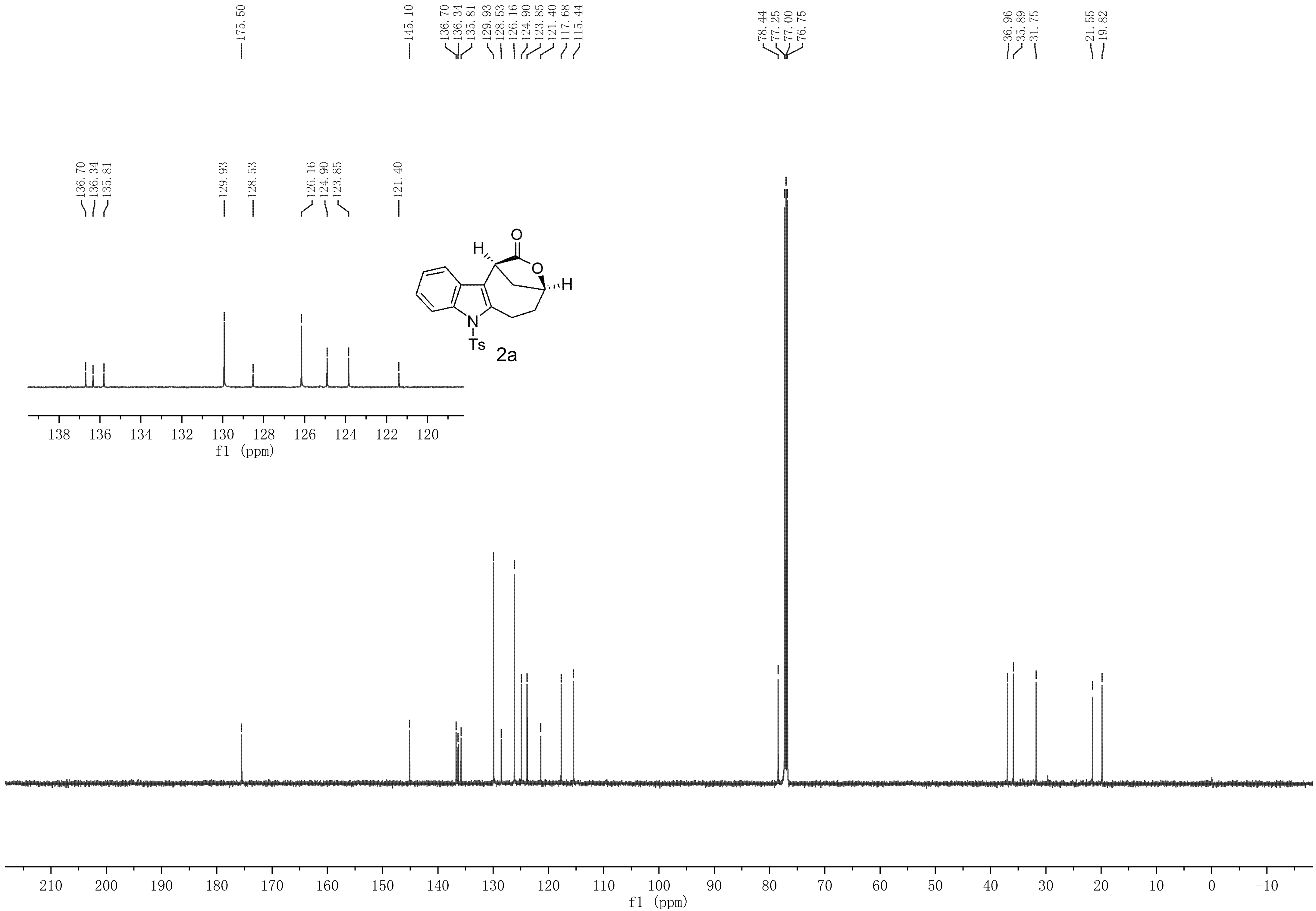
175.50
145.10
136.70
136.34
135.81
129.93
128.53
126.16
124.90
123.85
121.40
117.68
115.44
78.44
77.25
77.00
76.75
36.96
35.89
31.75
21.55
19.82
H
O
O
H
N
Ts
2a
138 136 134 132 130 128 126 124 122 120
f1 (ppm)
210 200 190 180 170 160 150 140 130 120 110 100 90 80 70 60 50 40 30 20 10 0 -10
f1 (ppm)

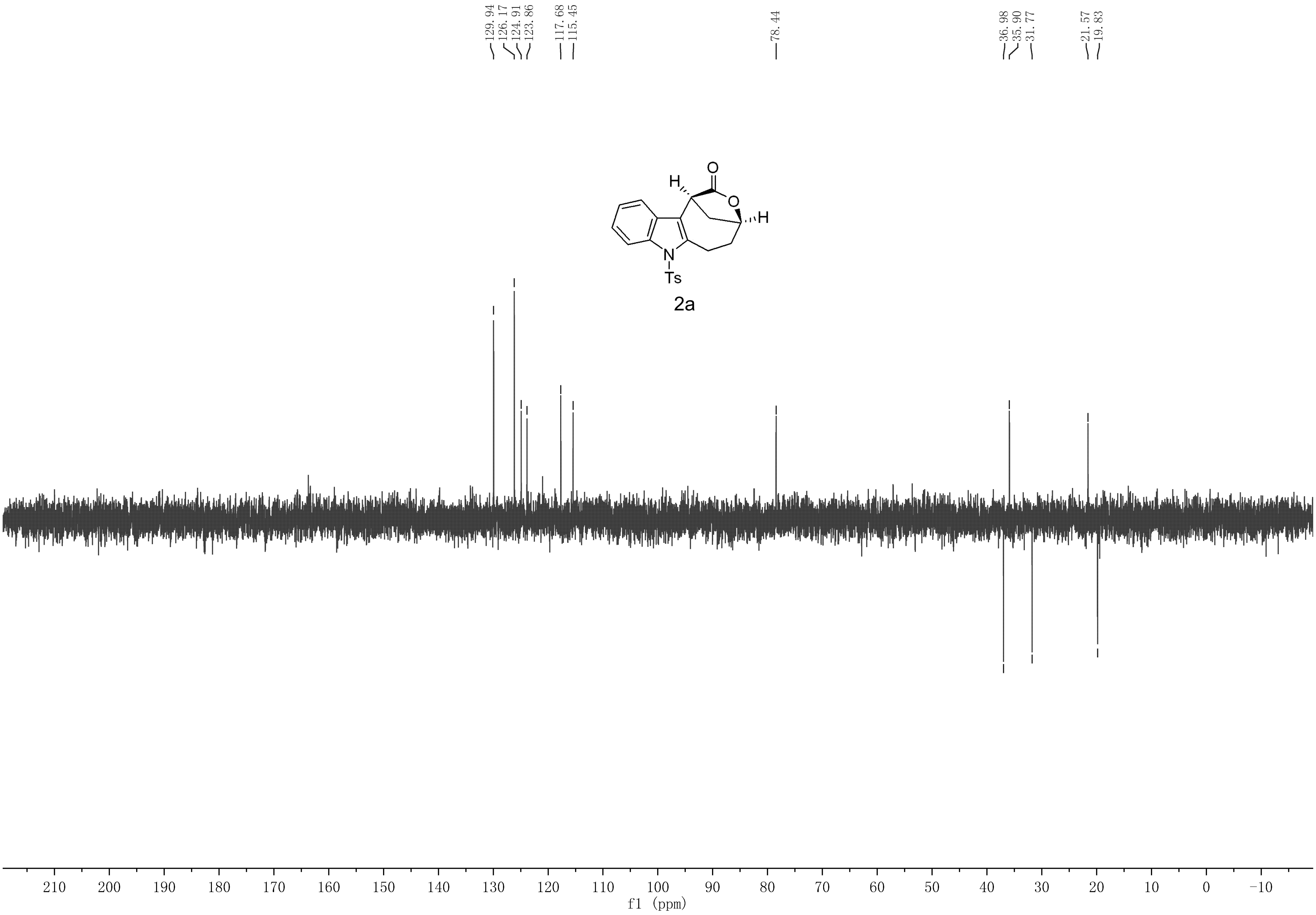

129.94
126.17
124.91
123.86
117.68
115.45
78.44
36.98
35.90
31.77
21.57
19.83
H
O
O
H
N
Ts
210
200
190
180
170
160
150
140
130
120
110
100
90
80
70
60
50
40
30
20
10
0
−10
f1 (ppm)

2a

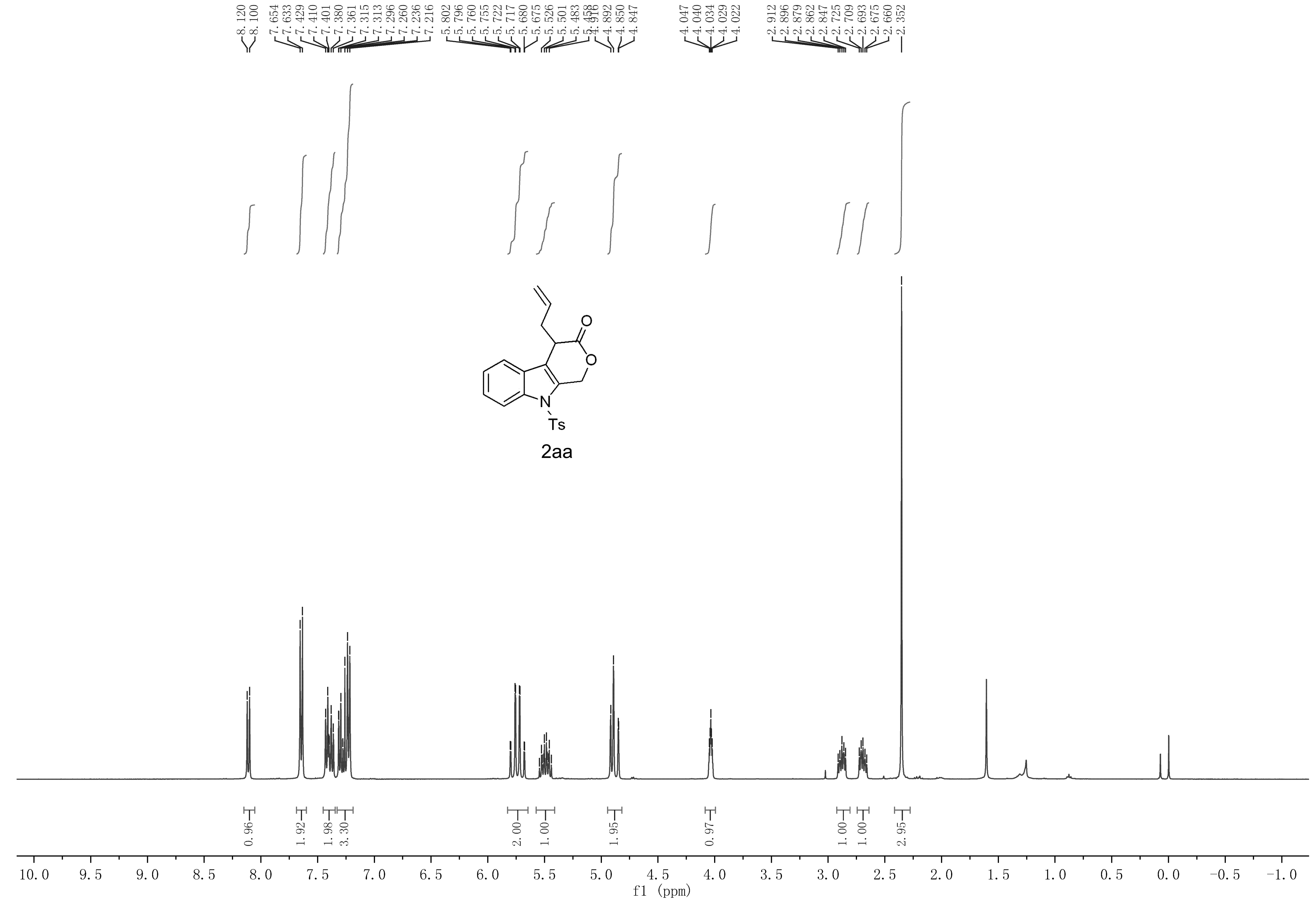
8.120
8.100
7.654
7.633
7.429
7.410
7.401
7.380
7.361
7.315
7.313
7.296
7.260
7.236
7.216
5.802
5.796
5.760
5.755
5.722
5.717
5.680
5.675
5.526
5.501
5.483
5.458
4.916
4.892
4.850
4.847
4.047
4.040
4.034
4.029
4.022
2.912
2.896
2.879
2.862
2.847
2.725
2.709
2.693
2.675
2.660
2.352
O
O
N
Ts
2aa
0.96
1.92
1.98
3.30
2.00
1.00
1.95
0.97
1.00
1.00
2.95
10.0 9.5 9.0 8.5 8.0 7.5 7.0 6.5 6.0 5.5 5.0 4.5 4.0 3.5 3.0 2.5 2.0 1.5 1.0 0.5 0.0 -0.5 -1.0
f1 (ppm)

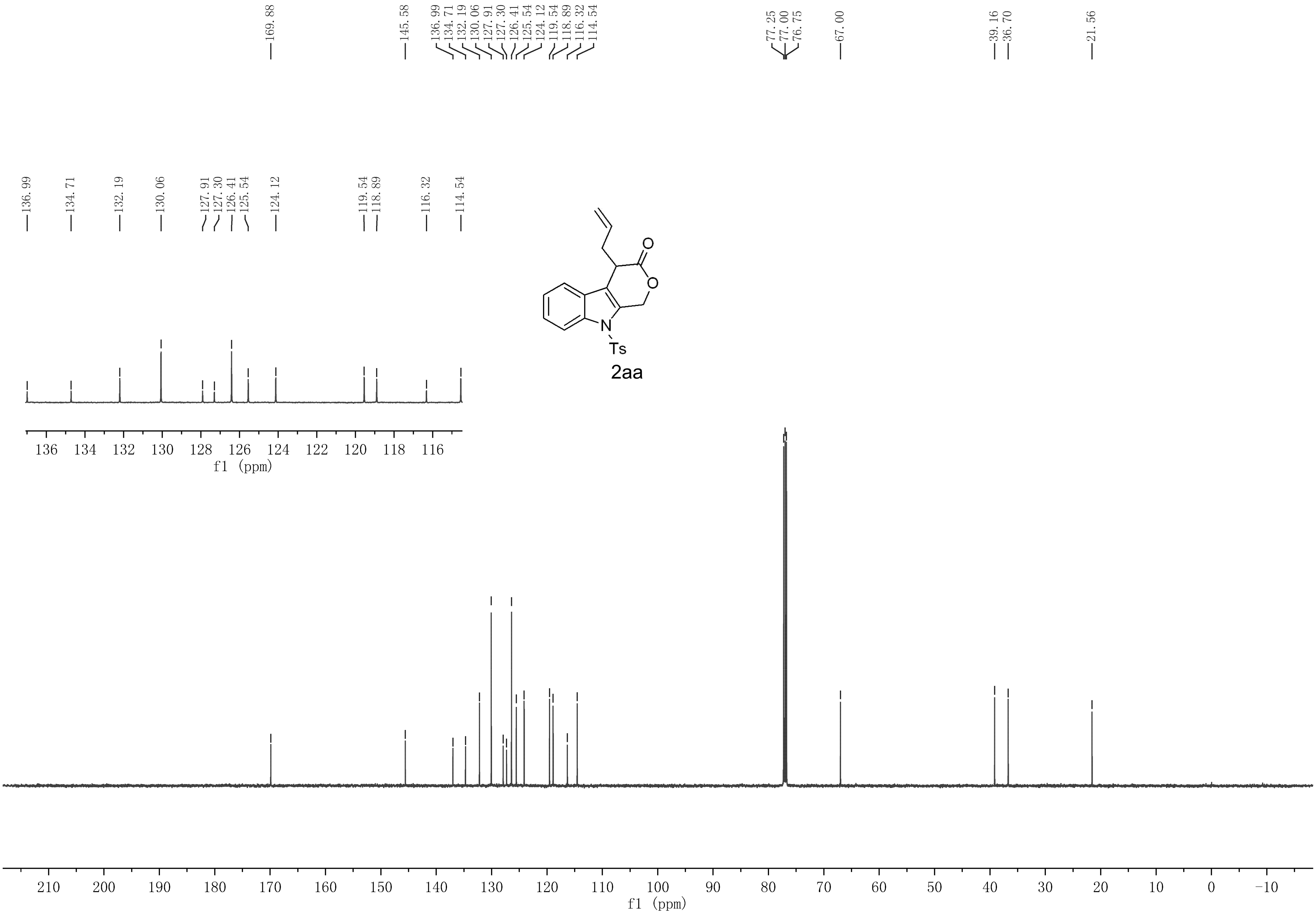
169.88
145.58
136.99
134.71
132.19
130.06
127.91
127.30
126.41
125.54
124.12
119.54
118.89
116.32
114.54
77.25
77.00
76.75
67.00
39.16
36.70
21.56
O
O
N
Ts
2aa
f1 (ppm)
f1 (ppm)

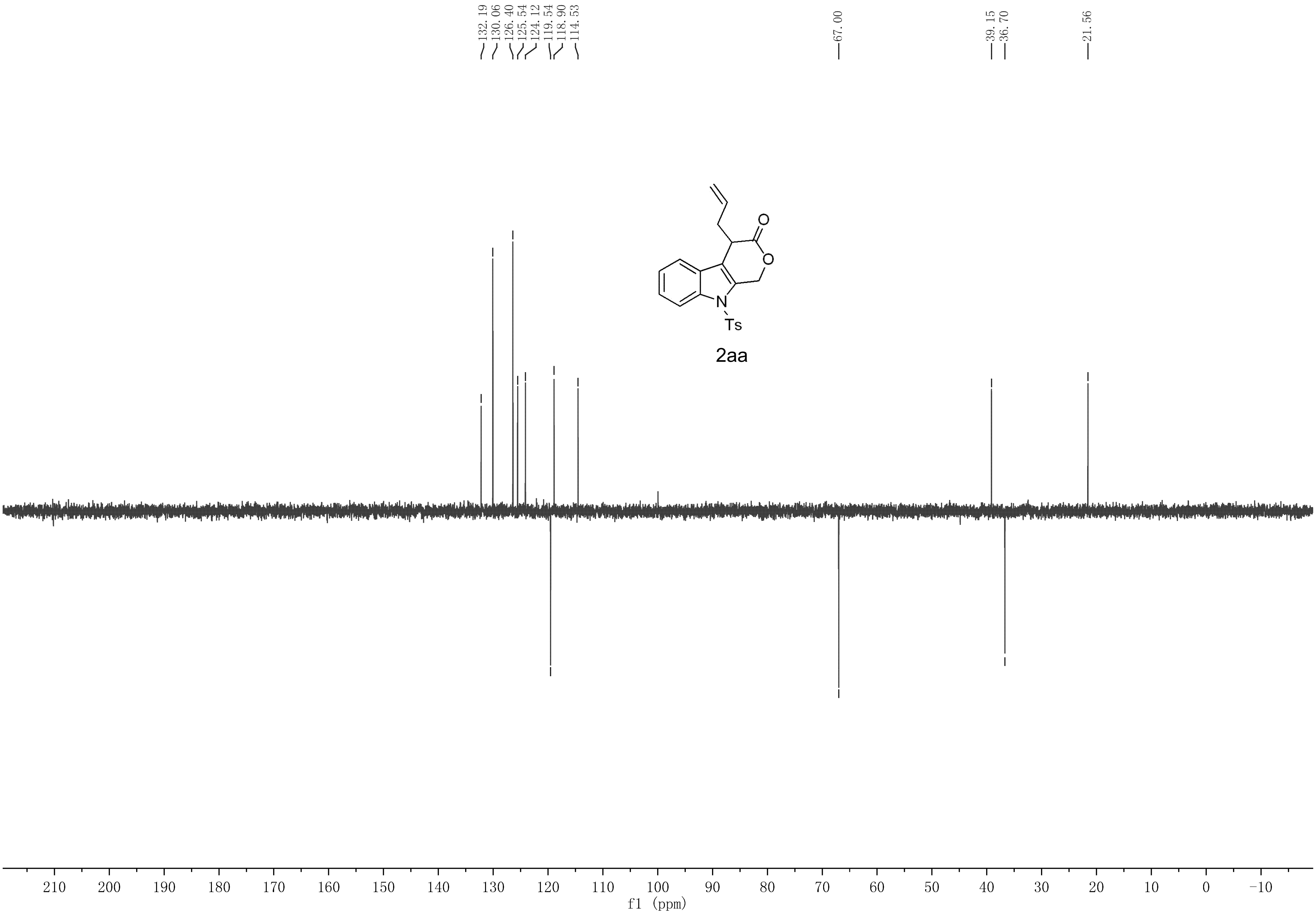
132.19
130.06
126.40
125.54
124.12
119.54
118.90
114.53
67.00
39.15
36.70
21.56
210
200
190
180
170
160
150
140
130
120
110
100
90
80
70
60
50
40
30
20
10
0
−10
f1 (ppm)
O
O
N
Ts

2aa

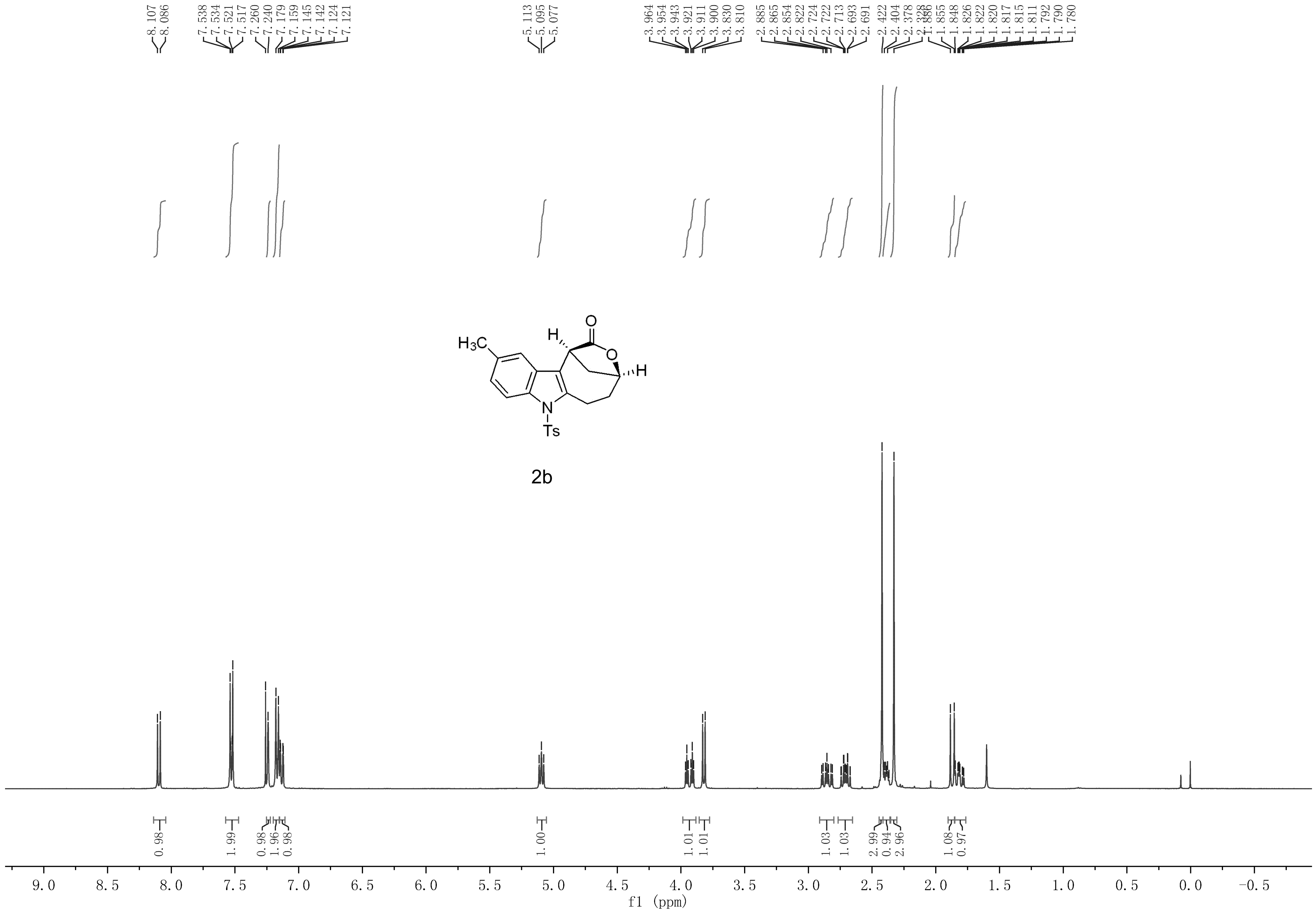
8.107
8.086
7.538
7.534
7.521
7.517
7.260
7.240
7.179
7.159
7.145
7.142
7.124
7.121
5.113
5.095
5.077
3.964
3.954
3.943
3.921
3.911
3.900
3.830
3.810
2.885
2.865
2.854
2.822
2.724
2.722
2.713
2.693
2.691
2.422
2.404
2.378
2.328
1.855
1.848
1.826
1.822
1.820
1.817
1.815
1.811
1.792
1.790
1.780
H3C
H
O
O
H
N
Ts
0.98
1.99
0.98
1.96
0.98
1.00
1.01
1.01
1.03
1.03
2.99
0.94
2.96
1.08
0.97
9.0 8.5 8.0 7.5 7.0 6.5 6.0 5.5 5.0 4.5 4.0 3.5 3.0 2.5 2.0 1.5 1.0 0.5 0.0 -0.5
f1 (ppm)

2b

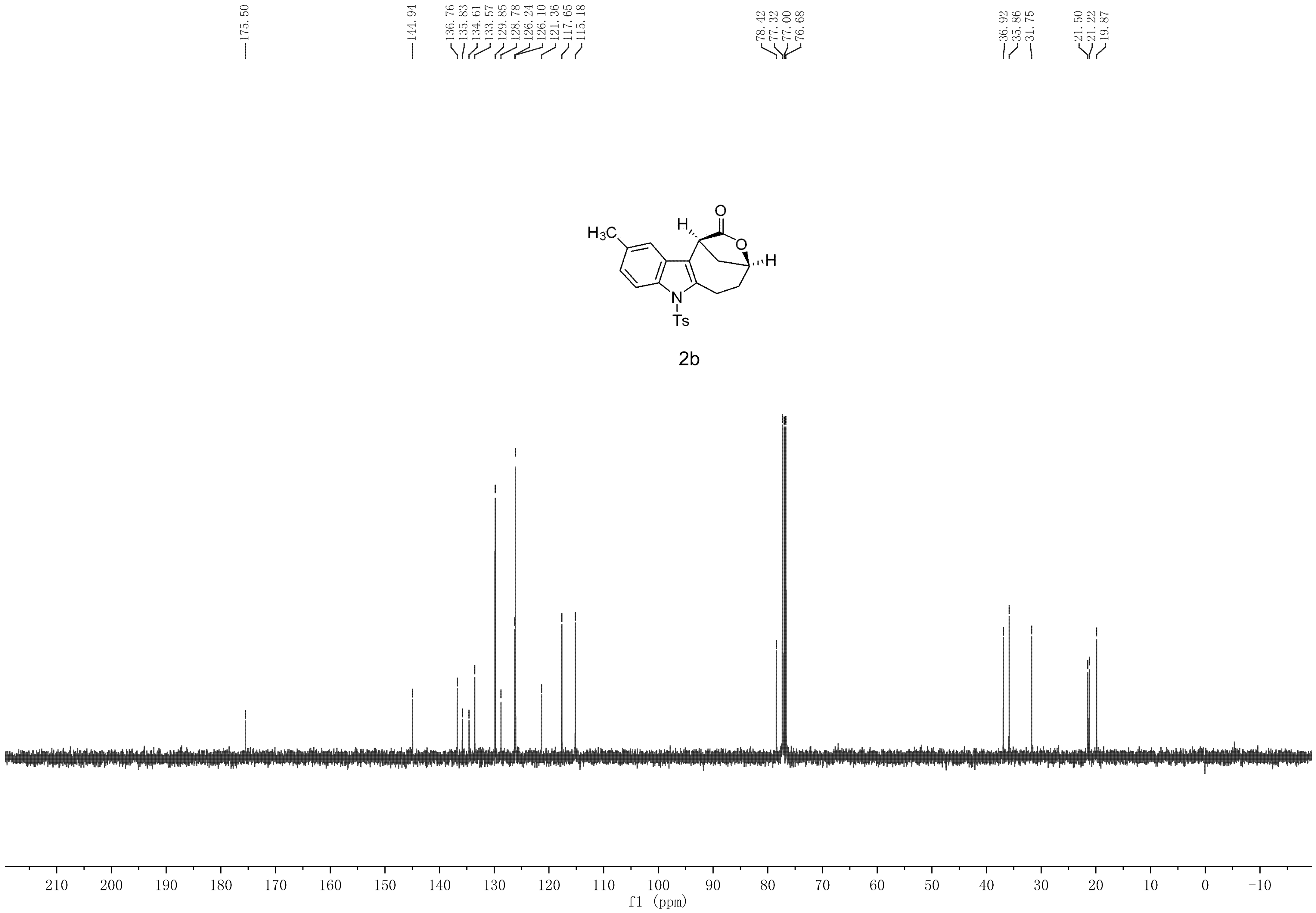
175.50
144.94
136.76
135.83
134.61
133.57
129.85
128.78
126.24
126.10
121.36
117.65
115.18
78.42
77.32
77.00
76.68
36.92
35.86
31.75
21.50
21.22
19.87
H3C
H
O
O
H
N
Ts
2b
210 200 190 180 170 160 150 140 130 120 110 100 90 80 70 60 50 40 30 20 10 0 −10
f1 (ppm)

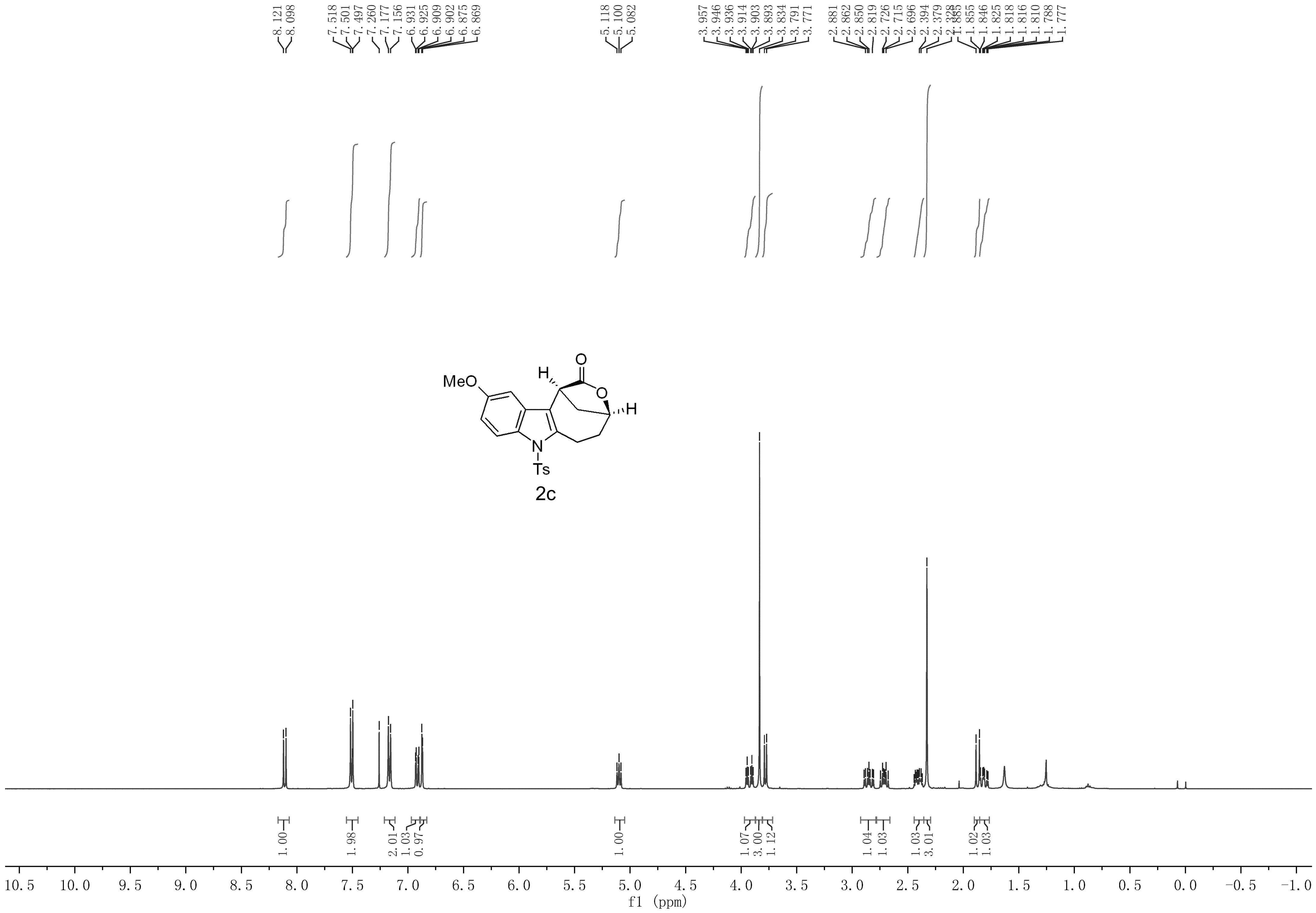

8.121
8.098
7.518
7.501
7.497
7.260
7.177
7.156
6.931
6.925
6.909
6.902
6.875
6.869
5.118
5.100
5.082
3.957
3.946
3.936
3.914
3.903
3.893
3.834
3.791
3.771
2.881
2.862
2.850
2.819
2.726
2.715
2.696
2.394
2.379
1.885
1.855
1.846
1.825
1.818
1.816
1.810
1.788
1.777
MeO
O
H
N
Ts
2c
1.00
1.98
2.01
1.03
0.97
1.00
1.07
3.00
1.12
1.04
1.03
1.03
3.01
1.02
1.03
10.5
10.0
9.5
9.0
8.5
8.0
7.5
7.0
6.5
6.0
5.5
5.0
4.5
4.0
3.5
3.0
2.5
2.0
1.5
1.0
0.5
0.0
-0.5
-1.0
f1 (ppm)

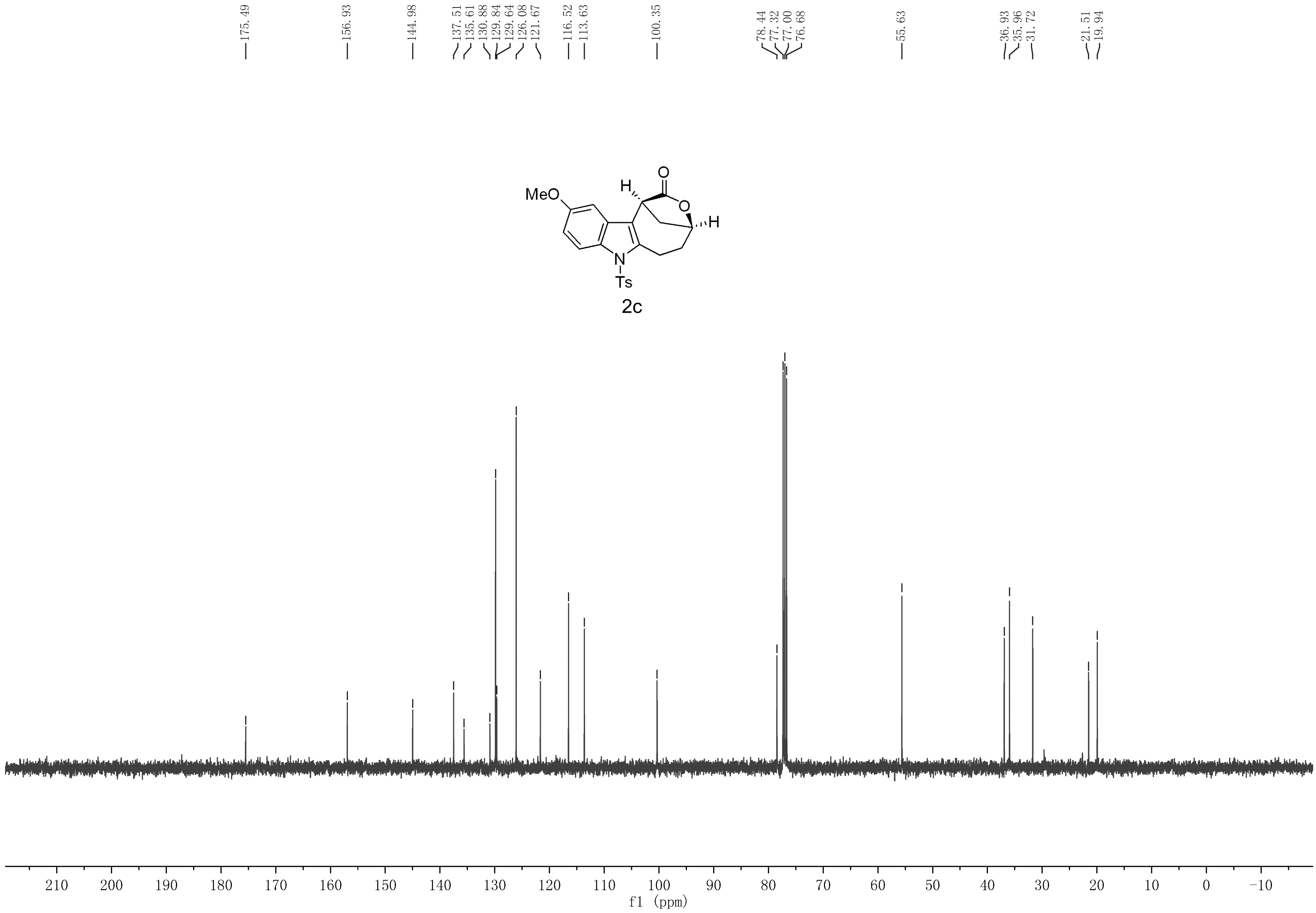
175.49
156.93
144.98
137.51
135.61
130.88
129.84
129.64
126.08
121.67
116.52
113.63
100.35
78.44
77.32
77.00
76.68
55.63
36.93
35.96
31.72
21.51
19.94
MeO
H
O
O
H
N
Ts
2c
210 200 190 180 170 160 150 140 130 120 110 100 90 80 70 60 50 40 30 20 10 0 −10
f1 (ppm)

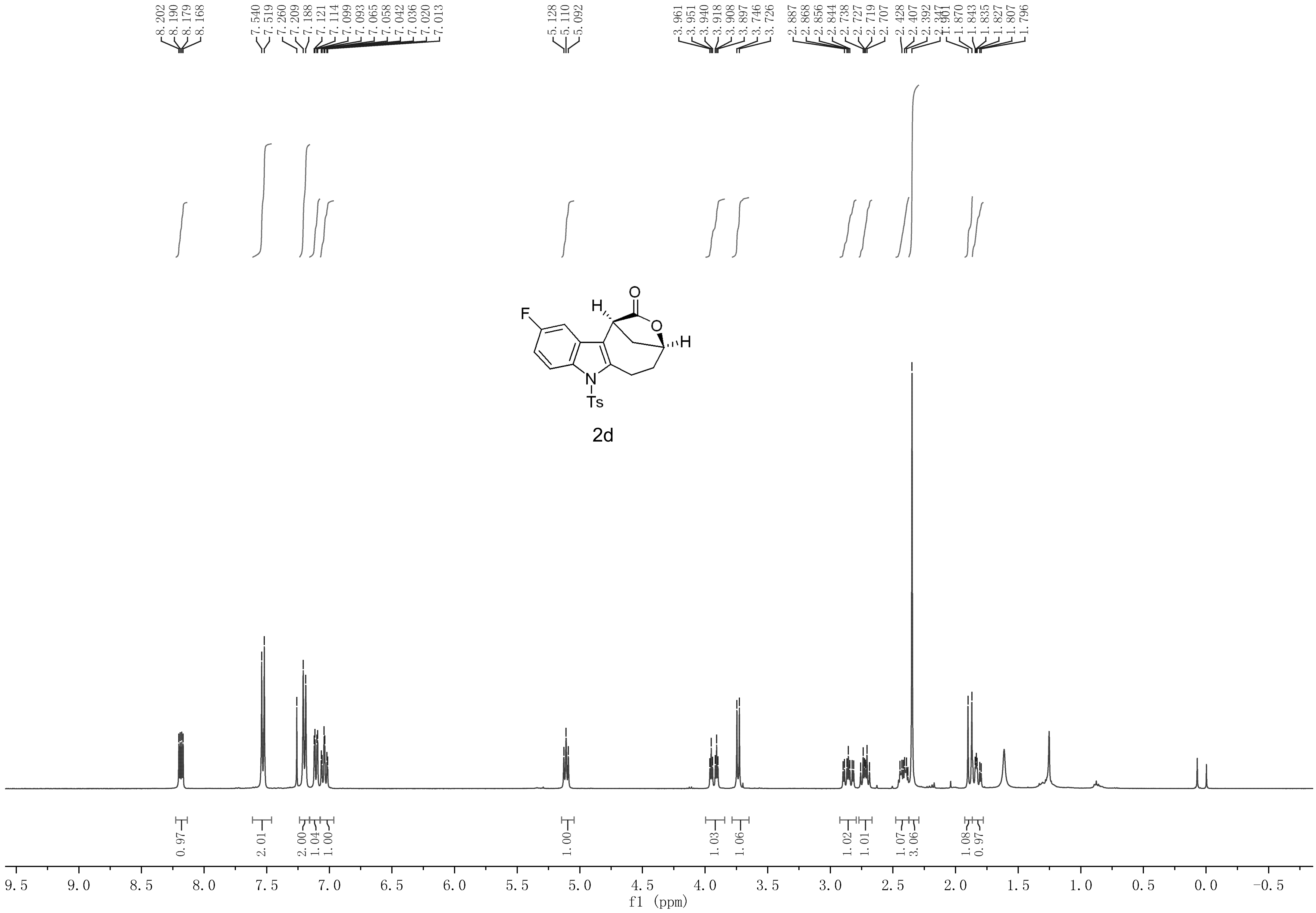

8.202
8.190
8.179
8.168
7.540
7.519
7.260
7.209
7.188
7.121
7.114
7.099
7.093
7.065
7.058
7.042
7.036
7.020
7.013
5.128
5.110
5.092
3.961
3.951
3.940
3.918
3.908
3.897
3.746
3.726
2.887
2.868
2.856
2.844
2.738
2.727
2.719
2.707
2.428
2.407
2.392
2.347
1.901
1.870
1.843
1.835
1.827
1.807
1.796
F
H
O
H
N
Ts
0.97
2.01
2.00
1.04
1.00
1.00
1.03
1.06
1.02
1.01
1.07
3.06
1.08
0.97
9.5
9.0
8.5
8.0
7.5
7.0
6.5
6.0
5.5
5.0
4.5
4.0
3.5
3.0
2.5
2.0
1.5
1.0
0.5
0.0
-0.5
f1 (ppm)

2d

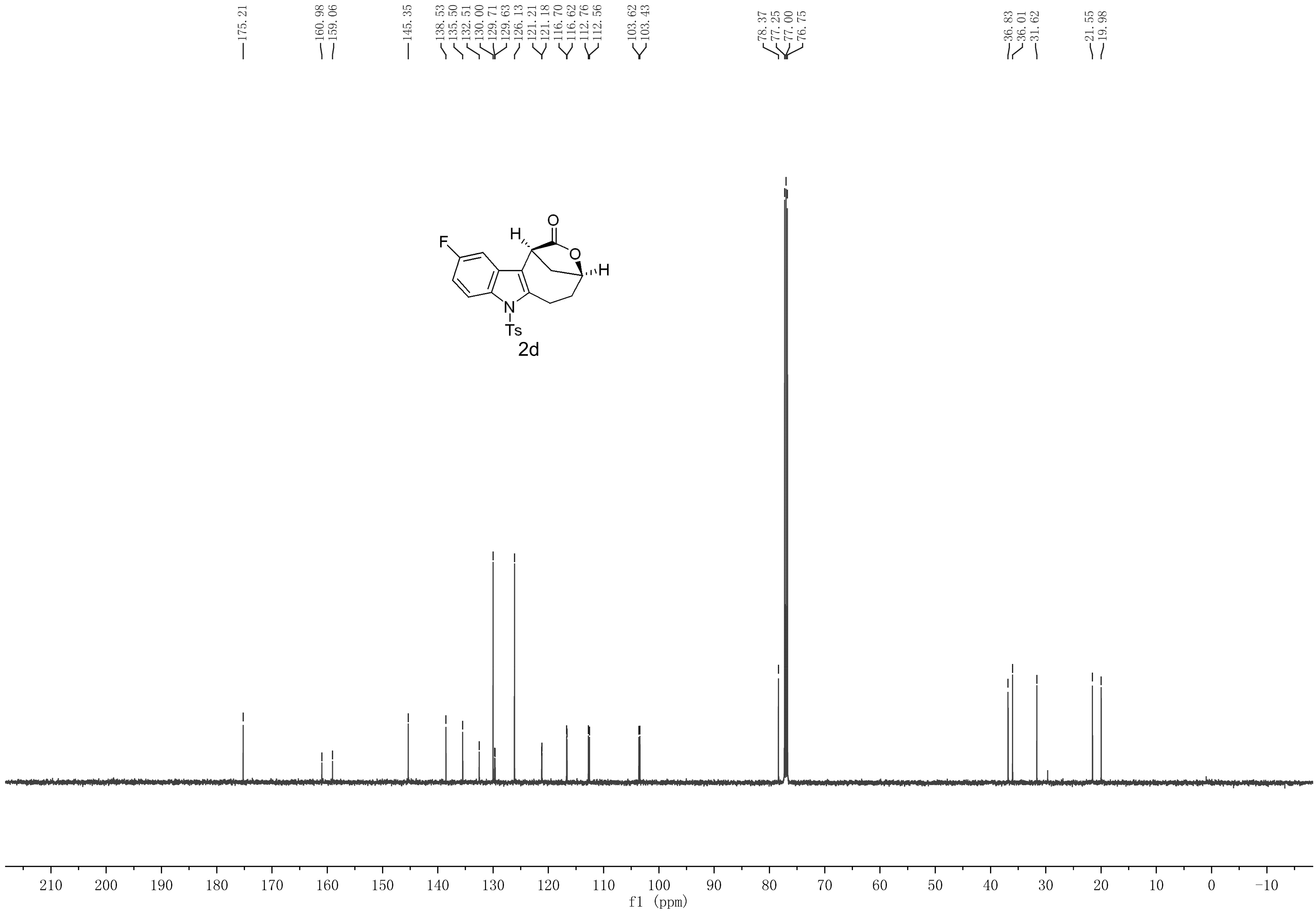
175.21
160.98
159.06
145.35
138.53
135.50
132.51
130.00
129.71
129.63
126.13
121.21
121.18
116.70
116.62
112.76
112.56
103.62
103.43
78.37
77.25
77.00
76.75
36.83
36.01
31.62
21.55
19.98
F
H
O
O
H
N
Ts
2d
210
200
190
180
170
160
150
140
130
120
110
100
90
80
70
60
50
40
30
20
10
0
−10
f1 (ppm)

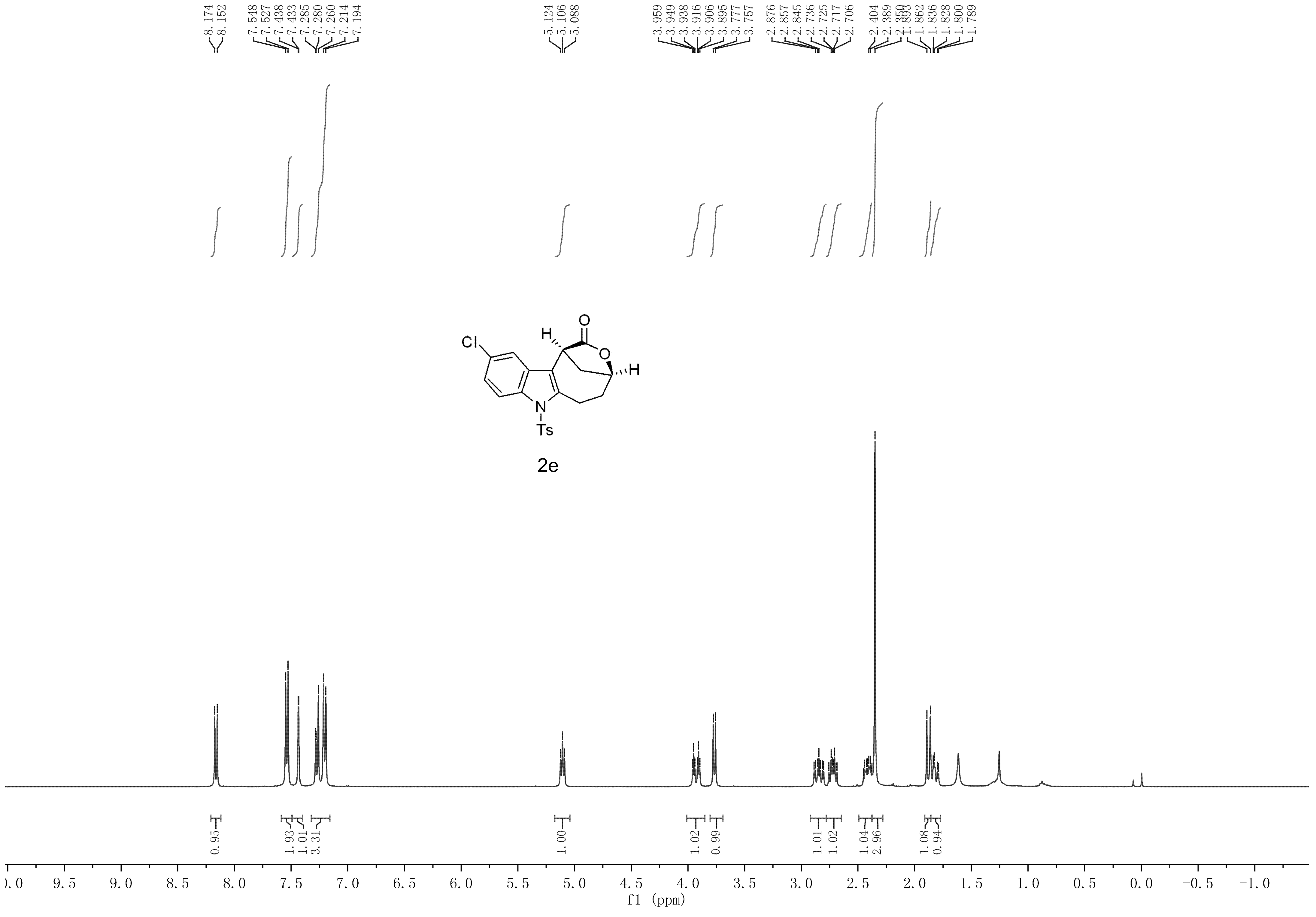
8.174
8.152
7.548
7.527
7.438
7.433
7.285
7.280
7.260
7.214
7.194
5.124
5.106
5.088
3.959
3.949
3.938
3.916
3.906
3.895
3.777
3.757
2.876
2.857
2.845
2.736
2.725
2.717
2.706
2.404
2.389
2.350
1.893
1.862
1.836
1.828
1.800
1.789
Cl
H
O
O
H
N
Ts
2e
0.95
1.93
1.01
3.31
1.00
1.02
0.99
1.01
1.02
1.04
2.96
1.08
0.94
9.5
9.0
8.5
8.0
7.5
7.0
6.5
6.0
5.5
5.0
4.5
4.0
3.5
3.0
2.5
2.0
1.5
1.0
0.5
0.0
-0.5
-1.0
f1 (ppm)

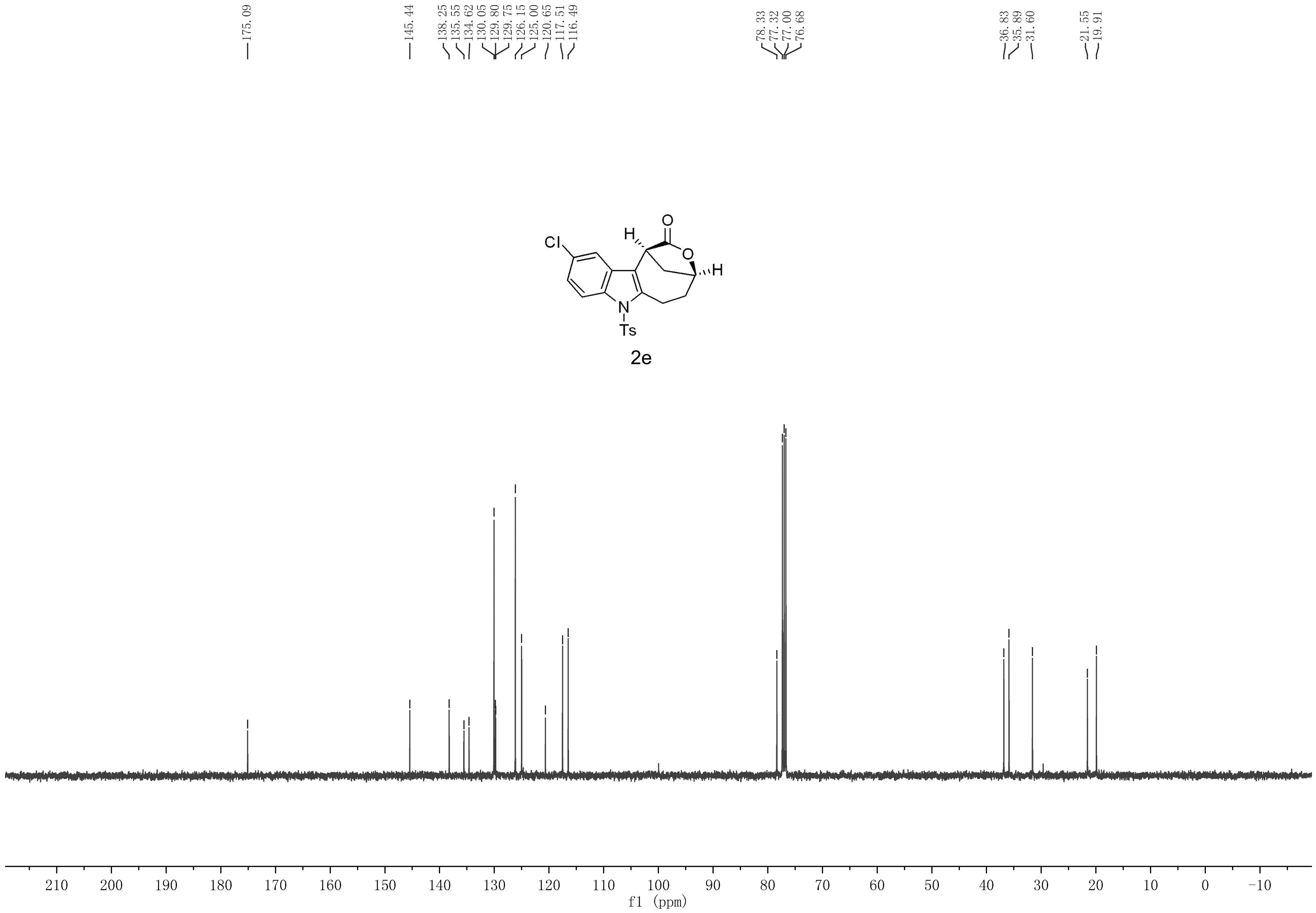
175.09
145.44
138.25
135.55
134.62
130.05
129.80
129.75
126.15
125.00
120.65
117.51
116.49
78.33
77.32
77.00
76.68
36.83
35.89
31.60
21.55
19.91
Cl
H
O
O
H
N
Ts
2e
210 200 190 180 170 160 150 140 130 120 110 100 90 80 70 60 50 40 30 20 10 0 −10
f1 (ppm)

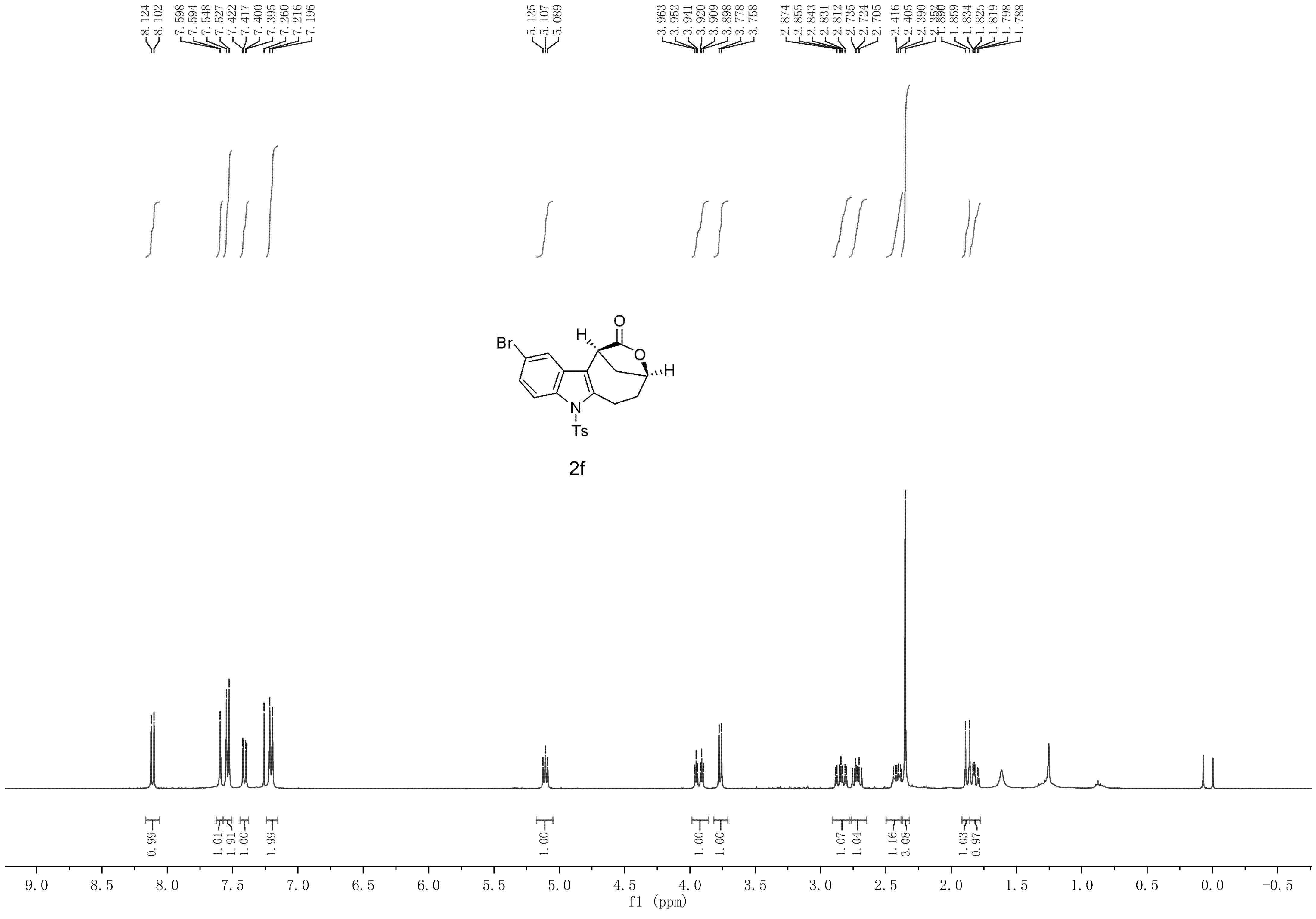
8.124
8.102
7.598
7.594
7.548
7.527
7.422
7.417
7.400
7.395
7.260
7.216
7.196
5.125
5.107
5.089
3.963
3.952
3.941
3.920
3.909
3.898
3.778
3.758
2.874
2.855
2.843
2.831
2.812
2.735
2.724
2.705
2.416
2.405
2.390
2.352
1.890
1.859
1.834
1.825
1.819
1.798
1.788
Br
H
O
O
H
N
Ts
2f
0.99
1.01
1.91
1.00
1.99
1.00
1.00
1.00
1.07
1.04
1.16
3.08
1.03
0.97
9.0 8.5 8.0 7.5 7.0 6.5 6.0 5.5 5.0 4.5 4.0 3.5 3.0 2.5 2.0 1.5 1.0 0.5 0.0 -0.5
f1 (ppm)

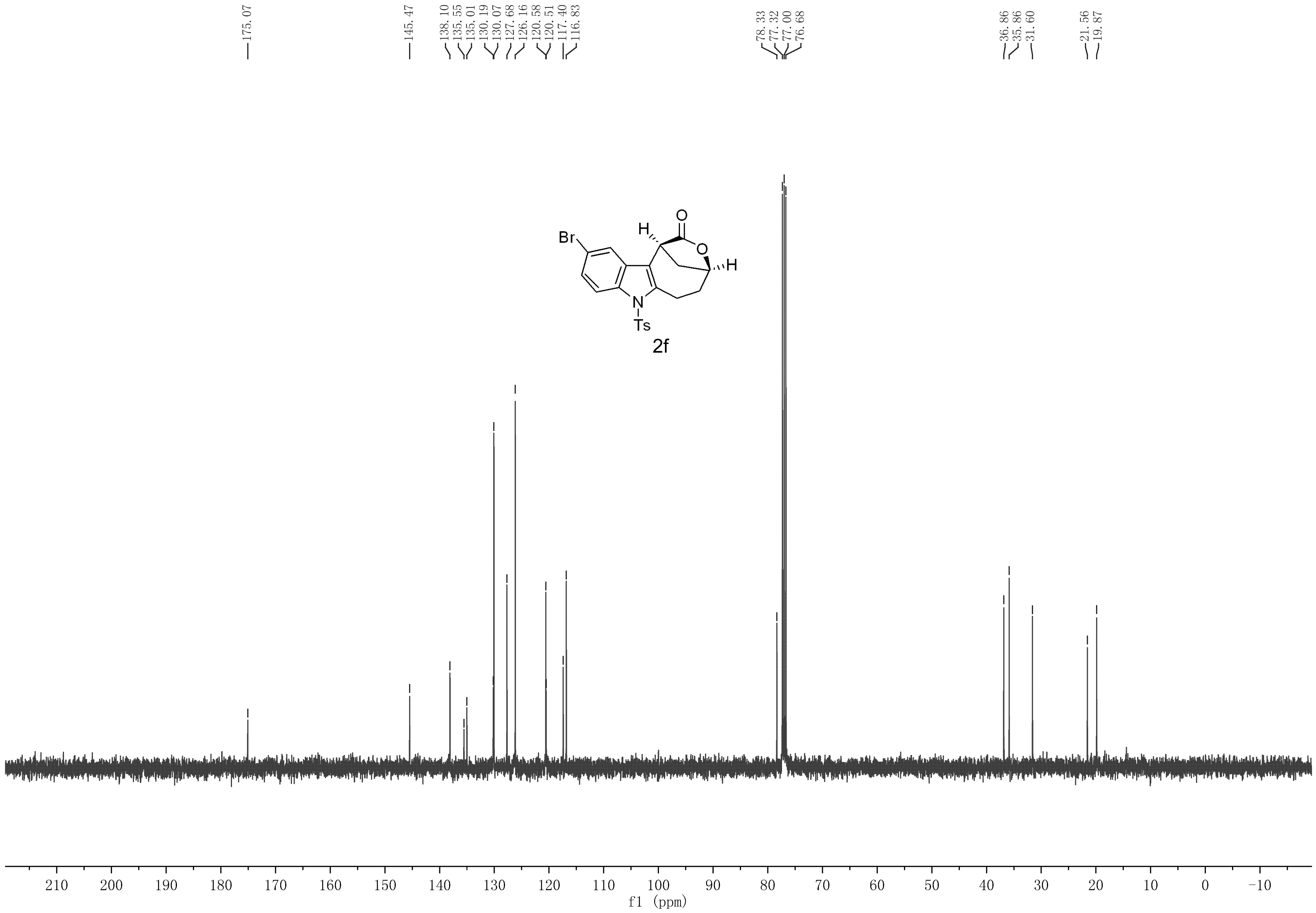

175.07
145.47
138.10
135.55
135.01
130.19
130.07
127.68
126.16
120.58
120.51
117.40
116.83
78.33
77.32
77.00
76.68
36.86
35.86
31.60
21.56
19.87
Br
H
O
O
H
N
Ts
2f
210 200 190 180 170 160 150 140 130 120 110 100 90 80 70 60 50 40 30 20 10 0 −10
f1 (ppm)

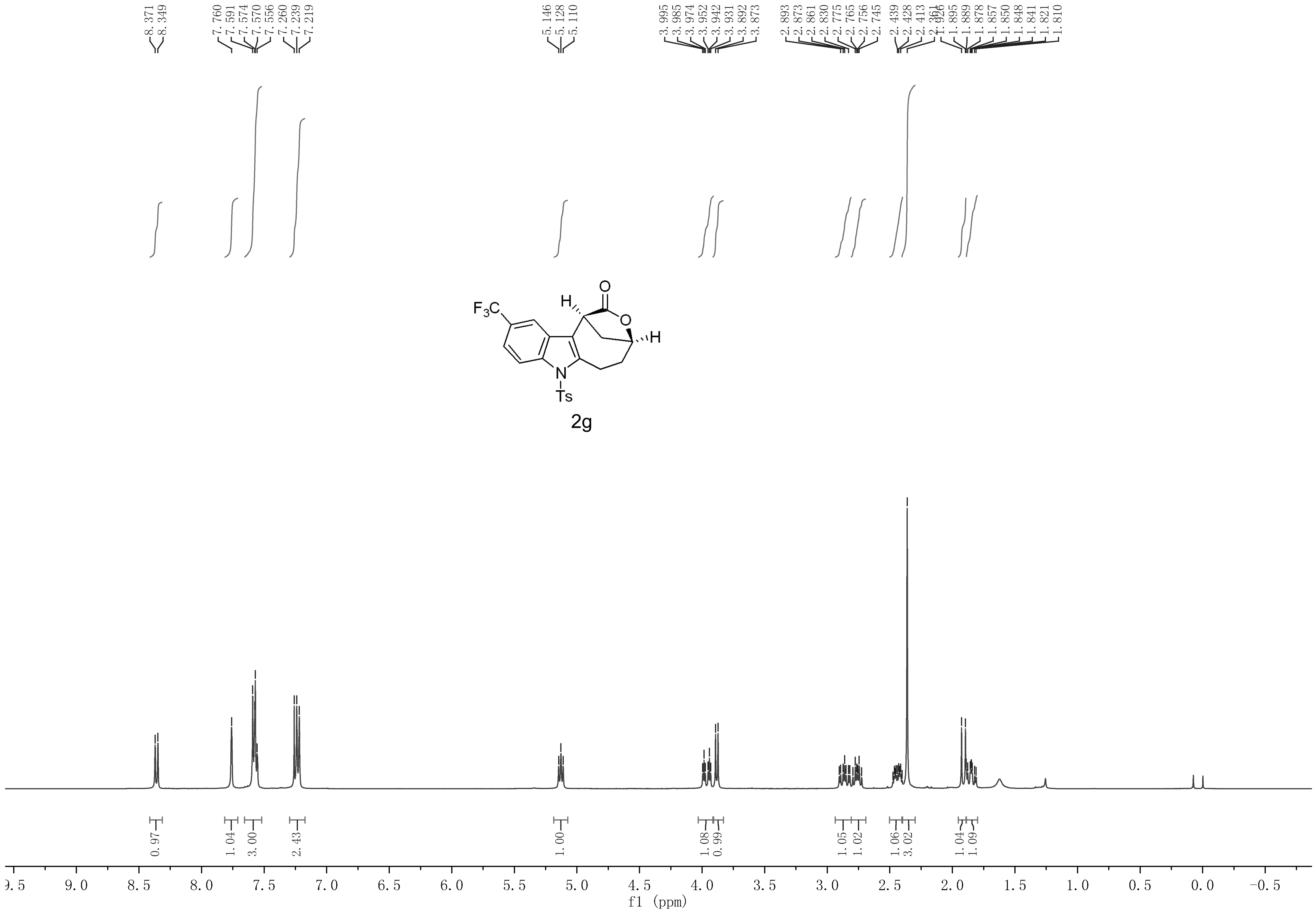

8.371
8.349
7.760
7.591
7.574
7.570
7.556
7.260
7.239
7.219
5.146
5.128
5.110
3.995
3.985
3.974
3.952
3.942
3.931
3.892
3.873
2.893
2.873
2.861
2.830
2.775
2.765
2.756
2.745
2.439
2.428
2.413
2.361
1.926
1.895
1.889
1.878
1.857
1.850
1.848
1.841
1.821
1.810
F3C
H
O
O
H
N
Ts
2g
0.97
1.04
3.00
2.43
1.00
1.08
0.99
1.05
1.02
1.06
3.02
1.04
1.09
9.0
8.5
8.0
7.5
7.0
6.5
6.0
5.5
5.0
4.5
4.0
3.5
3.0
2.5
2.0
1.5
1.0
0.5
0.0
-0.5
f1 (ppm)

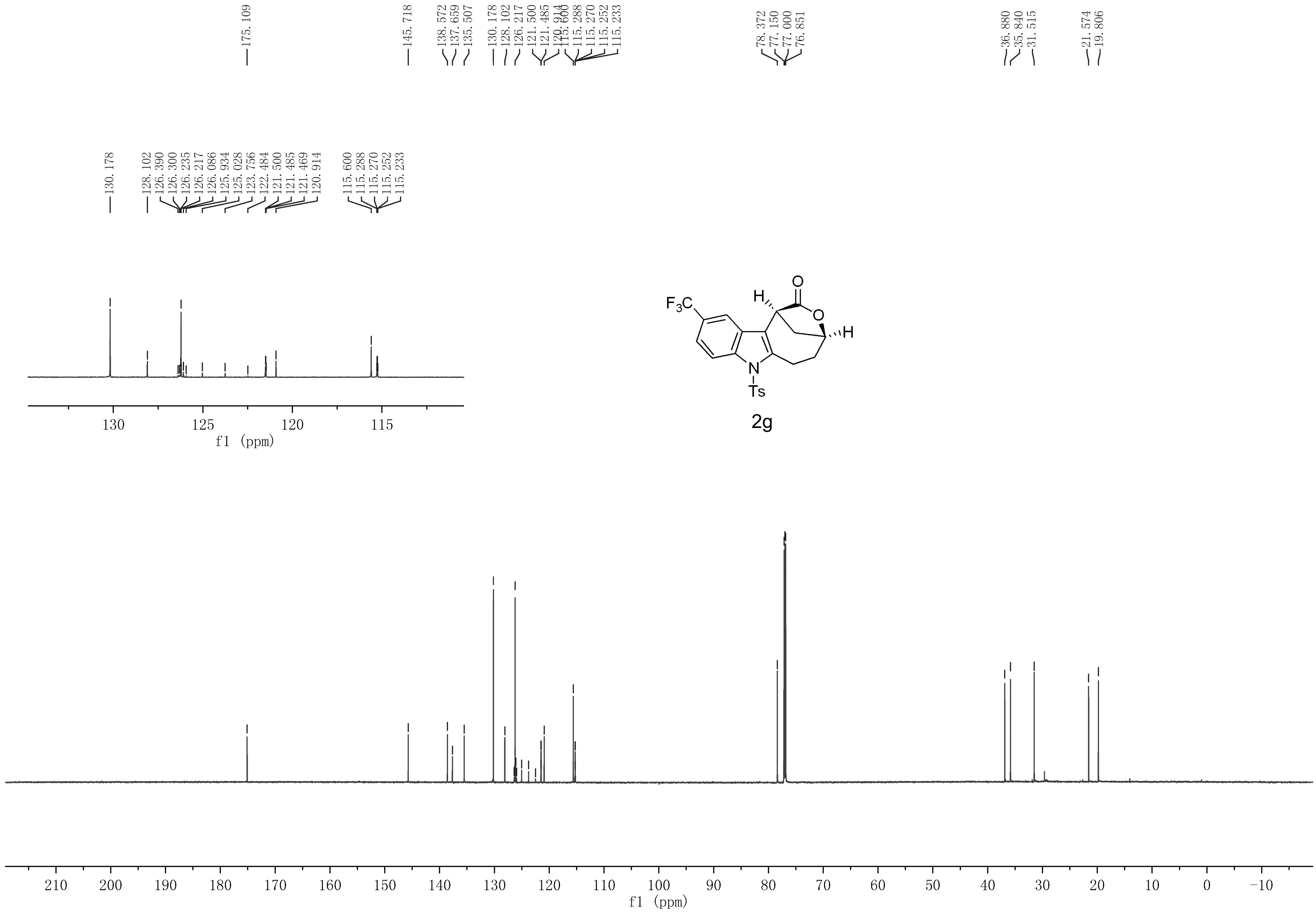
175.109
145.718
138.572
137.659
135.507
130.178
128.102
126.217
121.500
121.485
120.914
115.600
115.288
115.270
115.252
115.233
78.372
77.150
77.000
76.851
36.880
35.840
31.515
21.574
19.806
130.178
128.102
126.390
126.300
126.235
126.217
126.086
125.934
125.028
123.756
122.484
121.500
121.485
121.469
120.914
115.600
115.288
115.270
115.252
115.233
130 125 120 115
f1 (ppm)
F_3C
H
O
O
H
N
Ts
2g
210 200 190 180 170 160 150 140 130 120 110 100 90 80 70 60 50 40 30 20 10 0 −10
f1 (ppm)

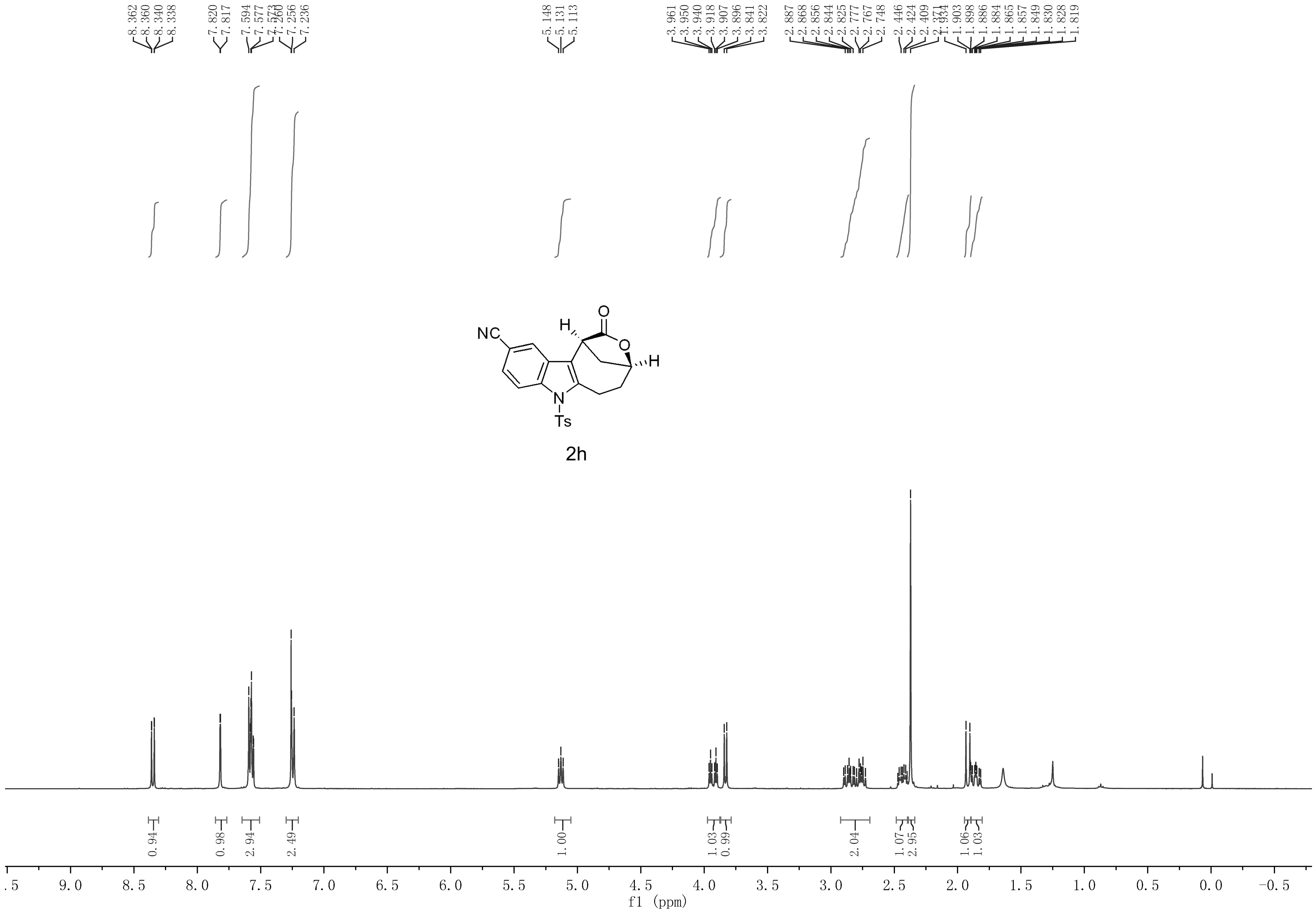
8.362
8.360
8.340
8.338
7.820
7.817
7.594
7.577
7.573
7.260
7.256
7.236
5.148
5.131
5.113
3.961
3.950
3.940
3.918
3.907
3.896
3.841
3.822
2.887
2.868
2.856
2.844
2.825
2.777
2.767
2.748
2.446
2.424
2.409
2.371
1.934
1.903
1.898
1.886
1.884
1.865
1.857
1.849
1.830
1.828
1.819
NC
H
O
O
H
N
Ts
2h
0.94
0.98
2.94
2.49
1.00
1.03
0.99
2.04
1.07
2.95
1.06
1.03
.5
9.0
8.5
8.0
7.5
7.0
6.5
6.0
5.5
5.0
4.5
4.0
3.5
3.0
2.5
2.0
1.5
1.0
0.5
0.0
-0.5
f1 (ppm)

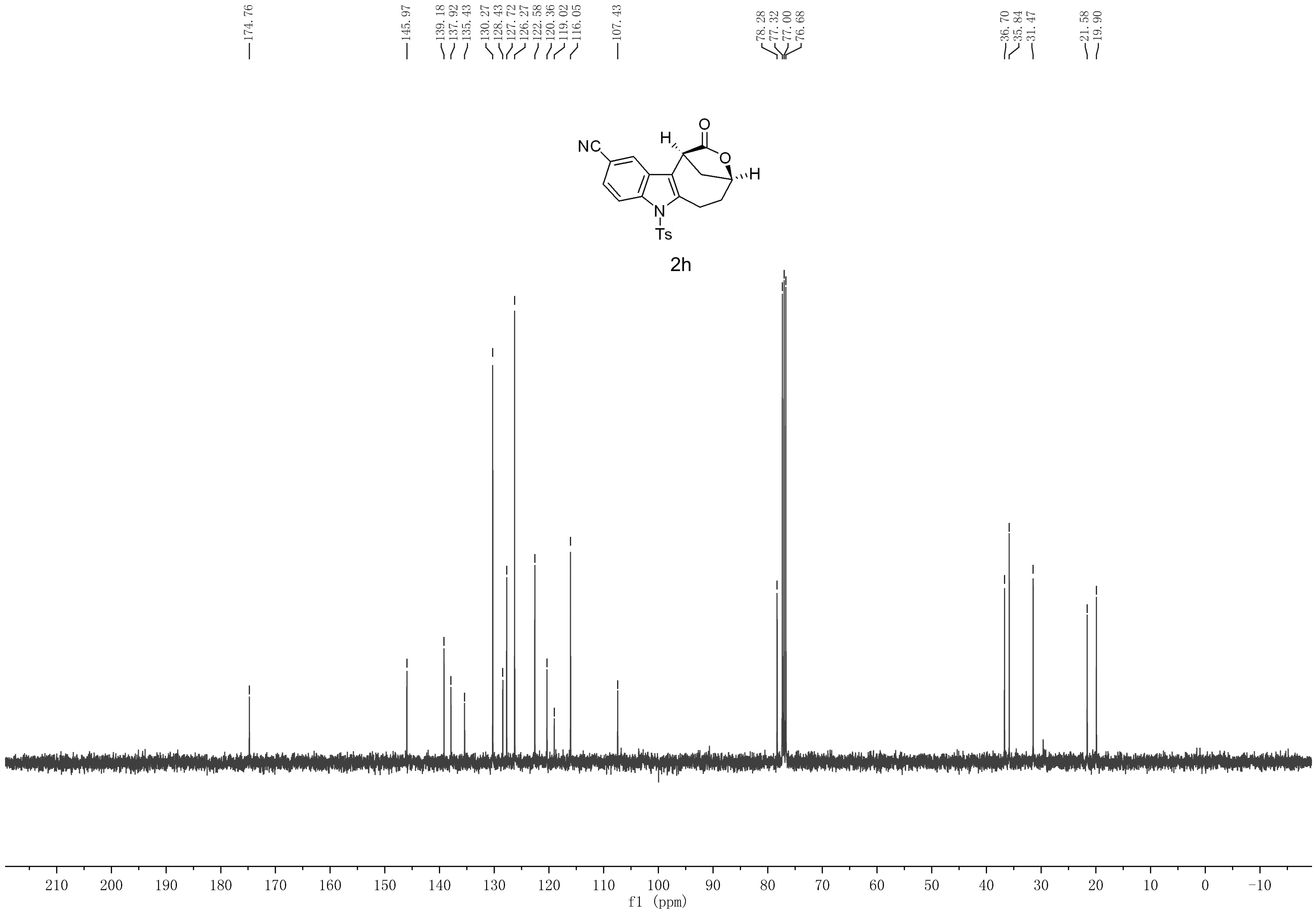
174.76
145.97
139.18
137.92
135.43
130.27
128.43
127.72
126.27
122.58
120.36
119.02
116.05
107.43
78.28
77.32
77.00
76.68
36.70
35.84
31.47
21.58
19.90
NC
H
O
O
H
N
Ts
2h
210 200 190 180 170 160 150 140 130 120 110 100 90 80 70 60 50 40 30 20 10 0 −10
f1 (ppm)

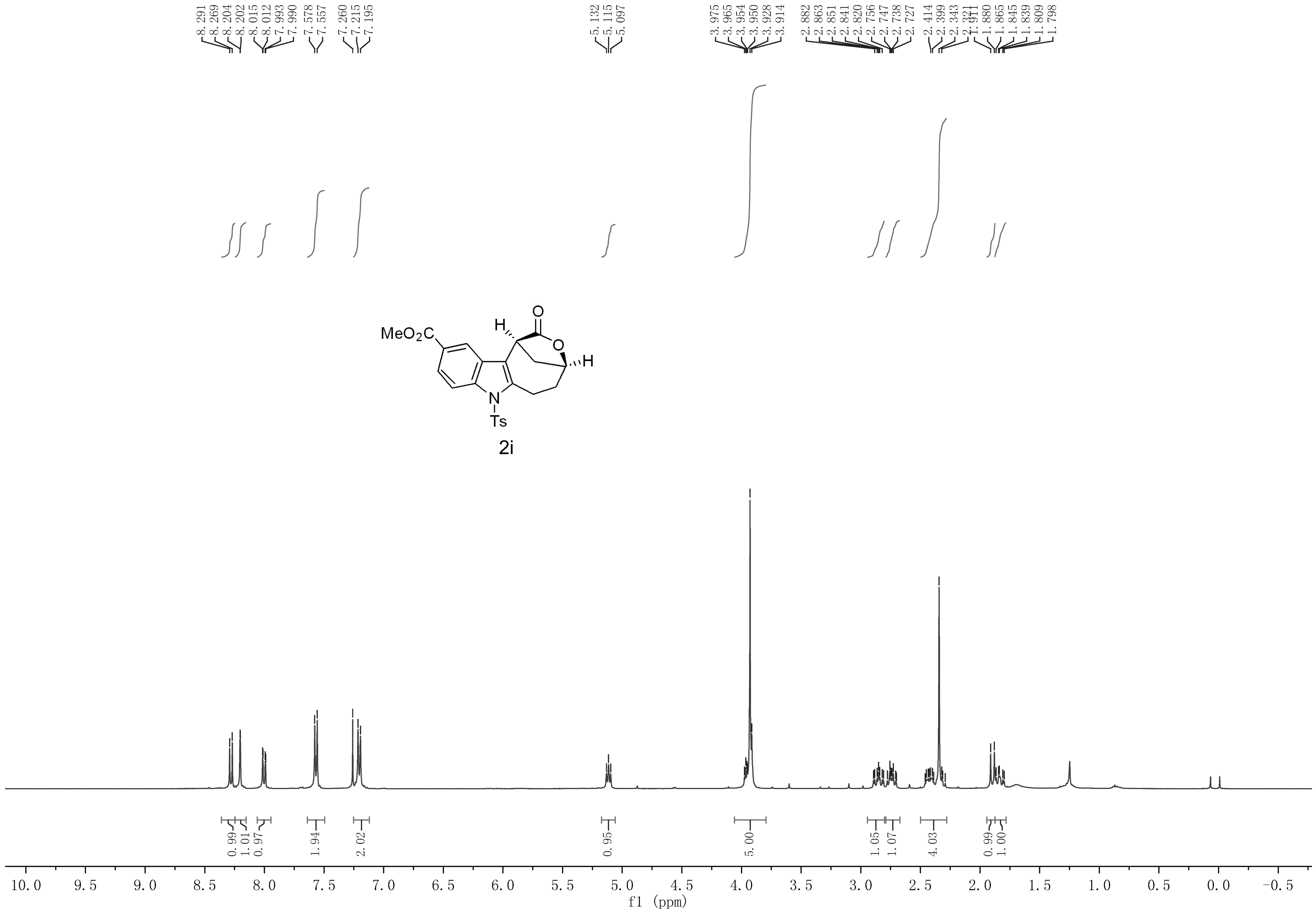
8.291
8.269
8.204
8.202
8.015
8.012
7.993
7.990
7.578
7.557
7.260
7.215
7.195
5.132
5.115
5.097
3.975
3.965
3.954
3.950
3.928
3.914
2.882
2.863
2.851
2.841
2.820
2.756
2.747
2.738
2.727
2.414
2.399
2.343
2.321
1.911
1.880
1.865
1.845
1.839
1.809
1.798
MeO2C
H
O
O
H
N
Ts
2i
0.99
1.01
0.97
1.94
2.02
0.95
5.00
1.05
1.07
4.03
0.99
1.00
10.0
9.5
9.0
8.5
8.0
7.5
7.0
6.5
6.0
5.5
5.0
4.5
4.0
3.5
3.0
2.5
2.0
1.5
1.0
0.5
0.0
−0.5
f1 (ppm)

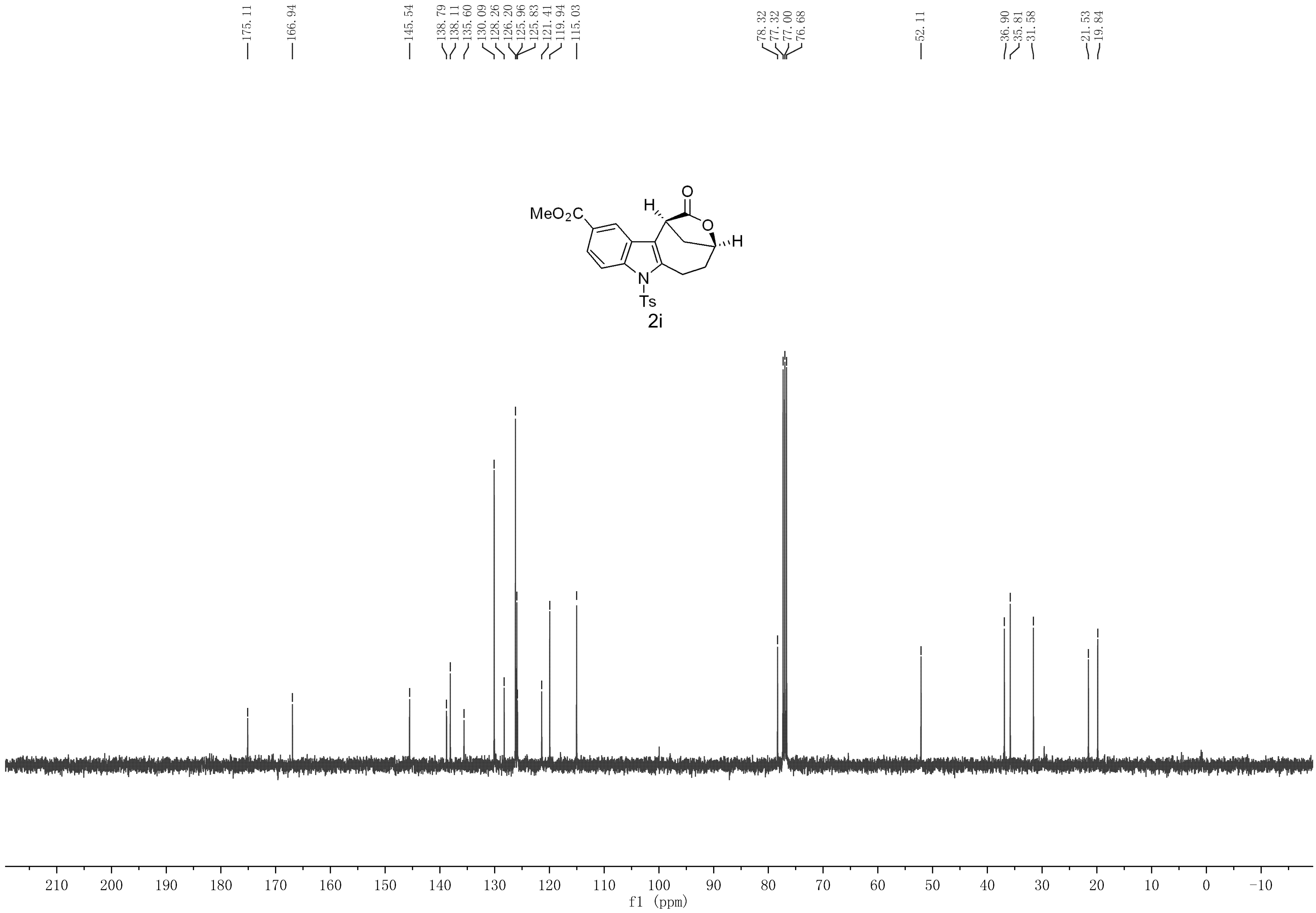
175.11
166.94
145.54
138.79
138.11
135.60
130.09
128.26
126.20
125.96
125.83
121.41
119.94
115.03
78.32
77.32
77.00
76.68
52.11
36.90
35.81
31.58
21.53
19.84
MeO2C
H
O
O
H
N
Ts
2i
210 200 190 180 170 160 150 140 130 120 110 100 90 80 70 60 50 40 30 20 10 0 −10
f1 (ppm)

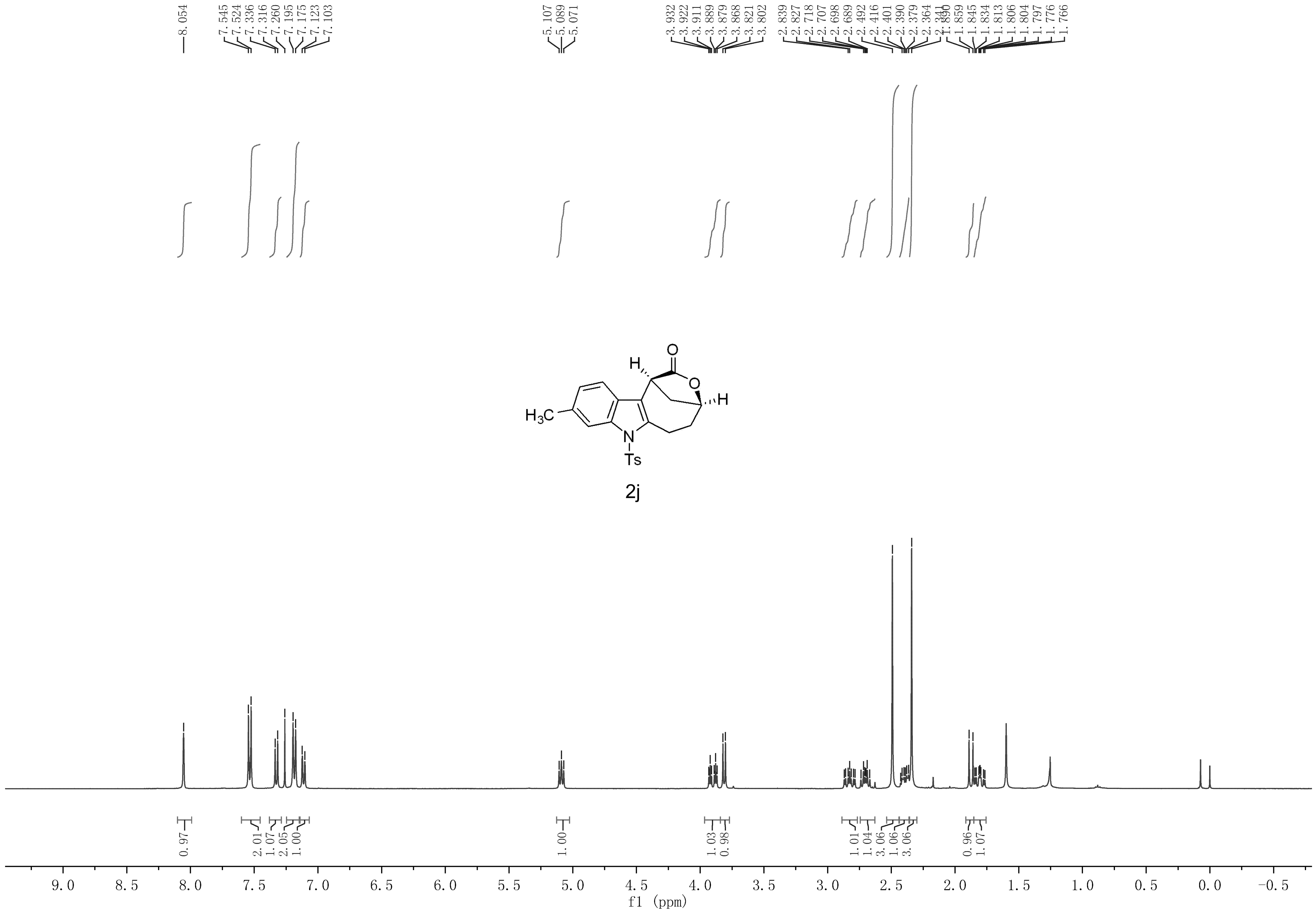
8.054
7.545
7.524
7.336
7.316
7.260
7.195
7.175
7.123
7.103
5.107
5.089
5.071
3.932
3.922
3.911
3.889
3.879
3.868
3.821
3.802
2.839
2.827
2.718
2.707
2.698
2.689
2.492
2.416
2.401
2.390
2.379
2.364
2.341
1.890
1.859
1.845
1.834
1.813
1.806
1.804
1.797
1.776
1.766
H
O
O
H
H_3C
N
Ts
2j
0.97
2.01
1.07
2.05
1.00
1.00
1.03
0.98
1.01
1.04
3.06
1.06
3.06
0.96
1.07
9.0
8.5
8.0
7.5
7.0
6.5
6.0
5.5
5.0
4.5
4.0
3.5
3.0
2.5
2.0
1.5
1.0
0.5
0.0
-0.5
f1 (ppm)

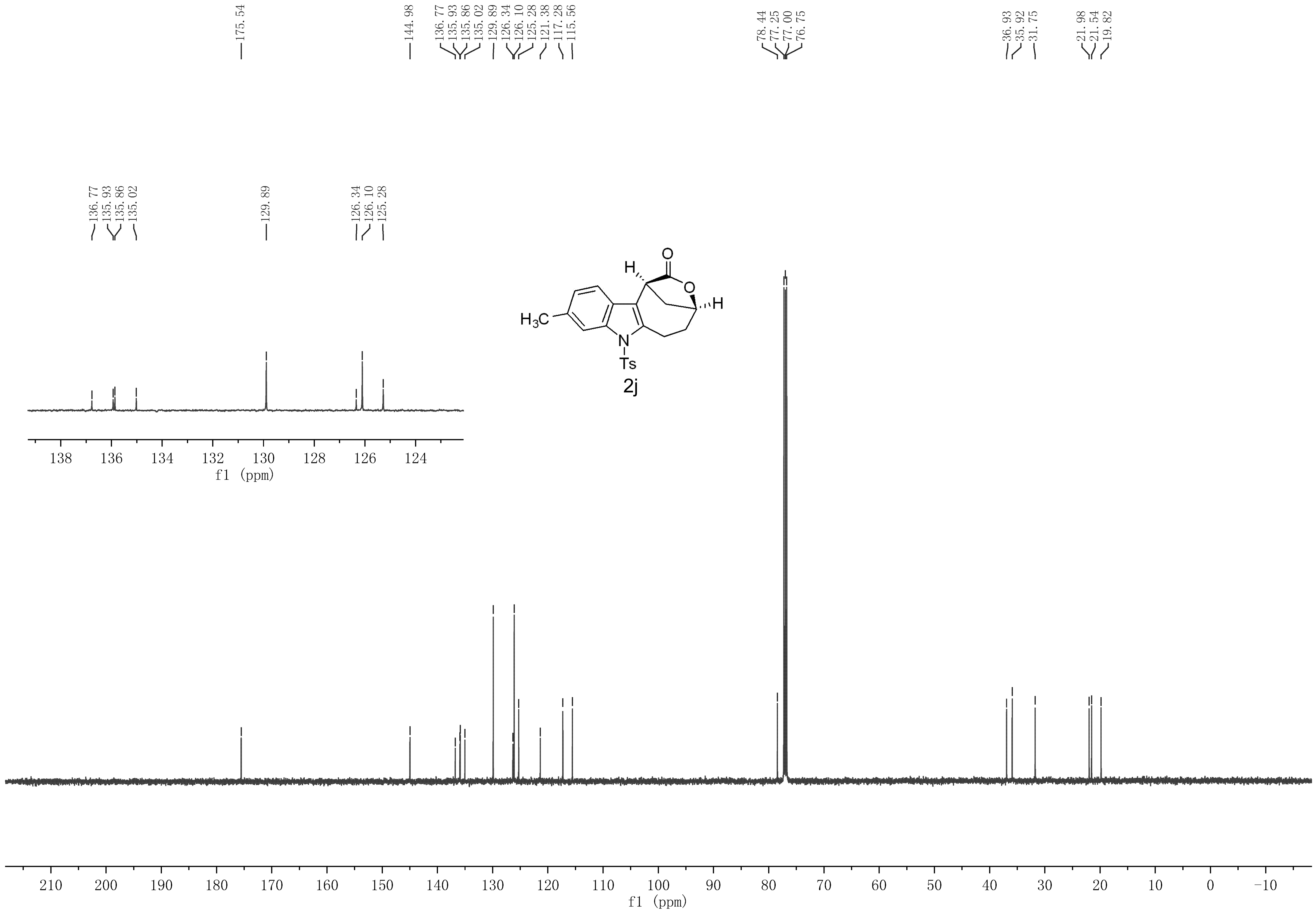
175.54
144.98
136.77
135.93
135.86
135.02
129.89
126.34
126.10
125.28
121.38
117.28
115.56
78.44
77.25
77.00
76.75
36.93
35.92
31.75
21.98
21.54
19.82
136.77
135.93
135.86
135.02
129.89
126.34
126.10
125.28
138 136 134 132 130 128 126 124
f1 (ppm)
O
H
O
H
H3C
N
Ts
2j
210 200 190 180 170 160 150 140 130 120 110 100 90 80 70 60 50 40 30 20 10 0 -10
f1 (ppm)

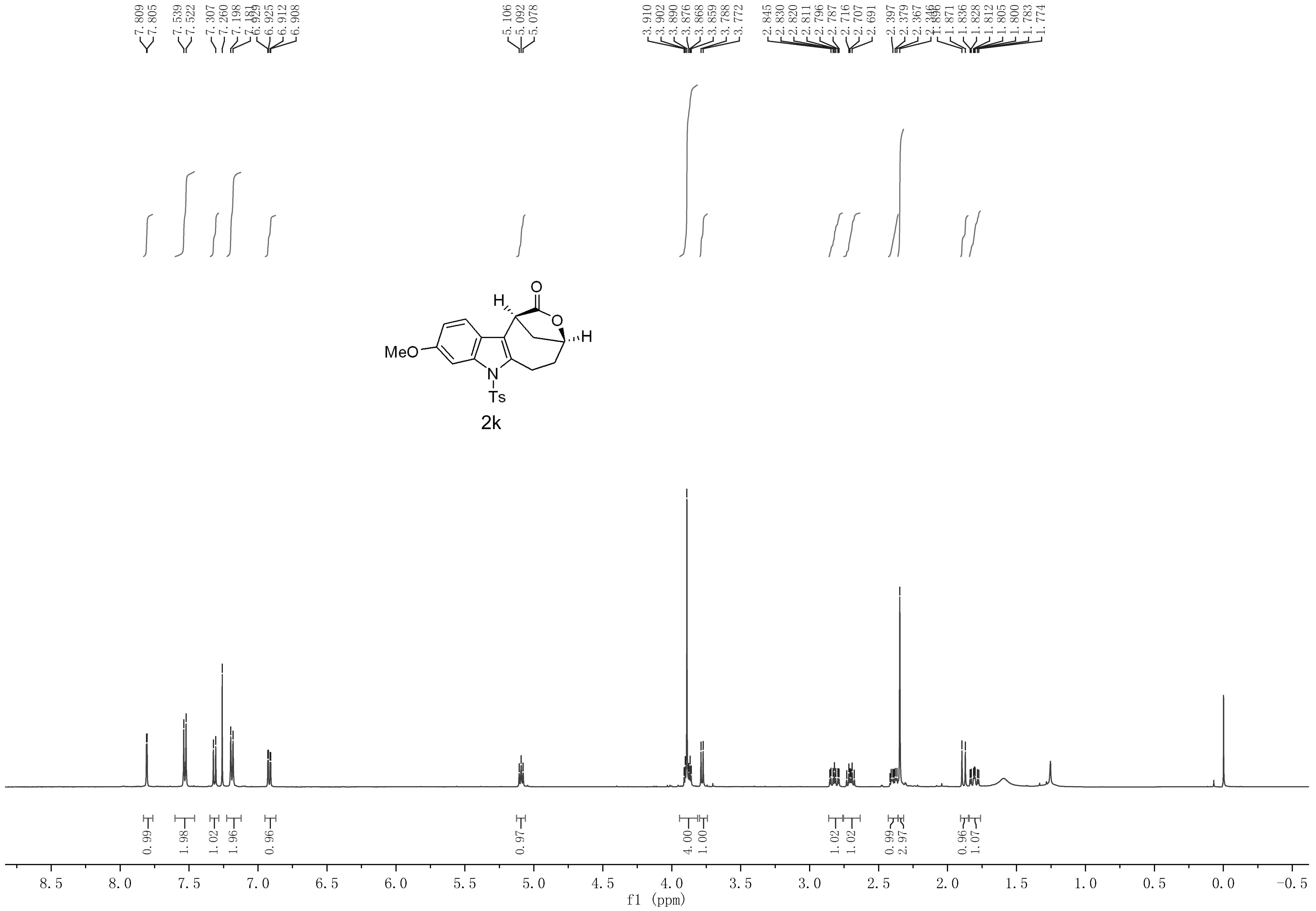

7.809
7.805
7.539
7.522
7.307
7.260
7.198
7.181
6.929
6.925
6.912
6.908
5.106
5.092
5.078
3.910
3.902
3.890
3.876
3.868
3.859
3.788
3.772
2.845
2.830
2.820
2.811
2.796
2.787
2.716
2.707
2.691
2.397
2.379
2.367
2.346
1.896
1.871
1.836
1.828
1.812
1.805
1.800
1.783
1.774
MeO
N
Ts
O
O
H
H
2k
0.99
1.98
1.02
1.96
0.96
0.97
4.00
1.00
1.02
1.02
0.99
2.97
0.96
1.07
8.5
8.0
7.5
7.0
6.5
6.0
5.5
5.0
4.5
4.0
3.5
3.0
2.5
2.0
1.5
1.0
0.5
0.0
-0.5
f1 (ppm)

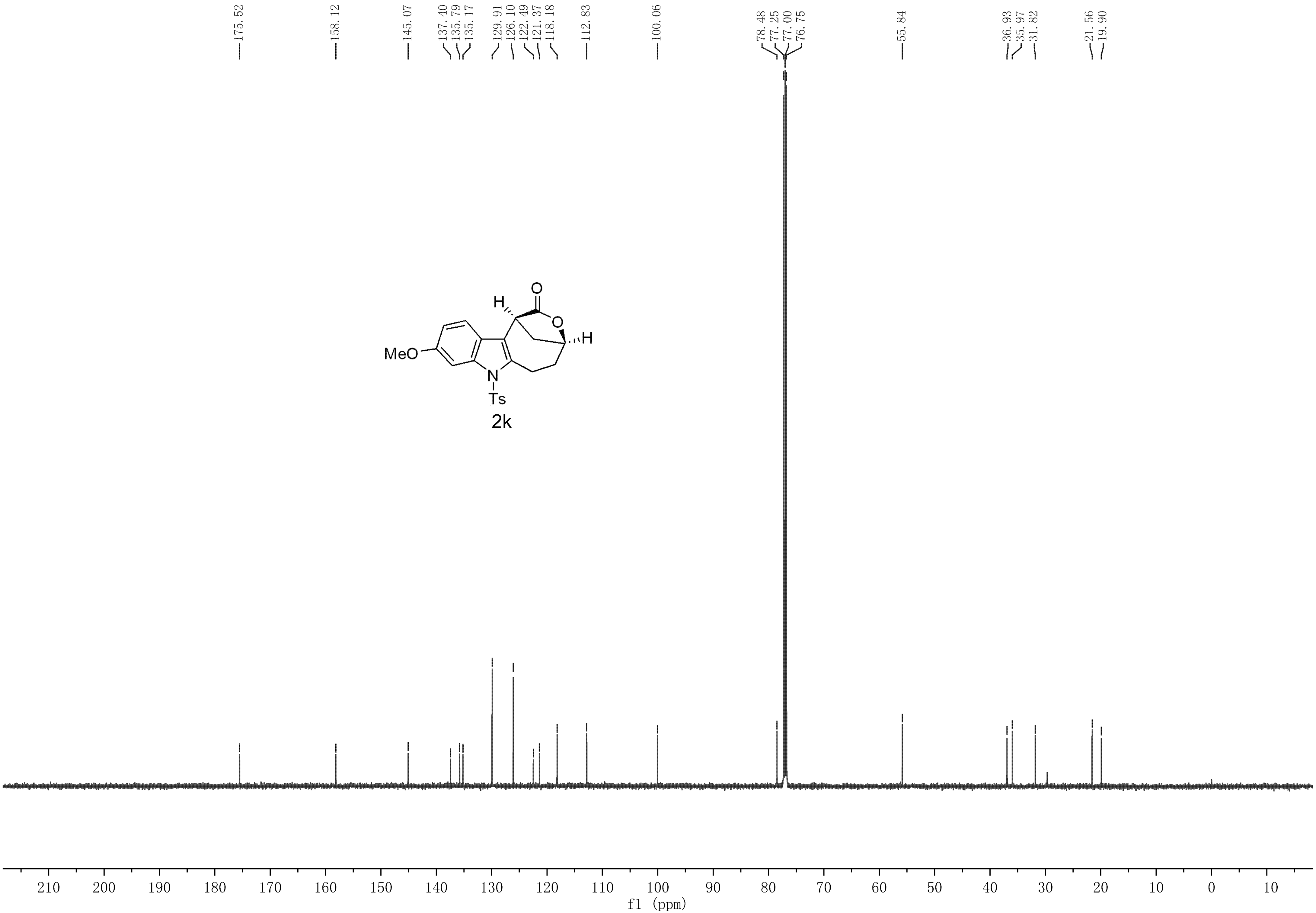
175.52
158.12
145.07
137.40
135.79
135.17
129.91
126.10
122.49
121.37
118.18
112.83
100.06
78.48
77.25
77.00
76.75
55.84
36.93
35.97
31.82
21.56
19.90
MeO
N
Ts
H
O
O
H
2k
210
200
190
180
170
160
150
140
130
120
110
100
90
80
70
60
50
40
30
20
10
0
−10
f1 (ppm)

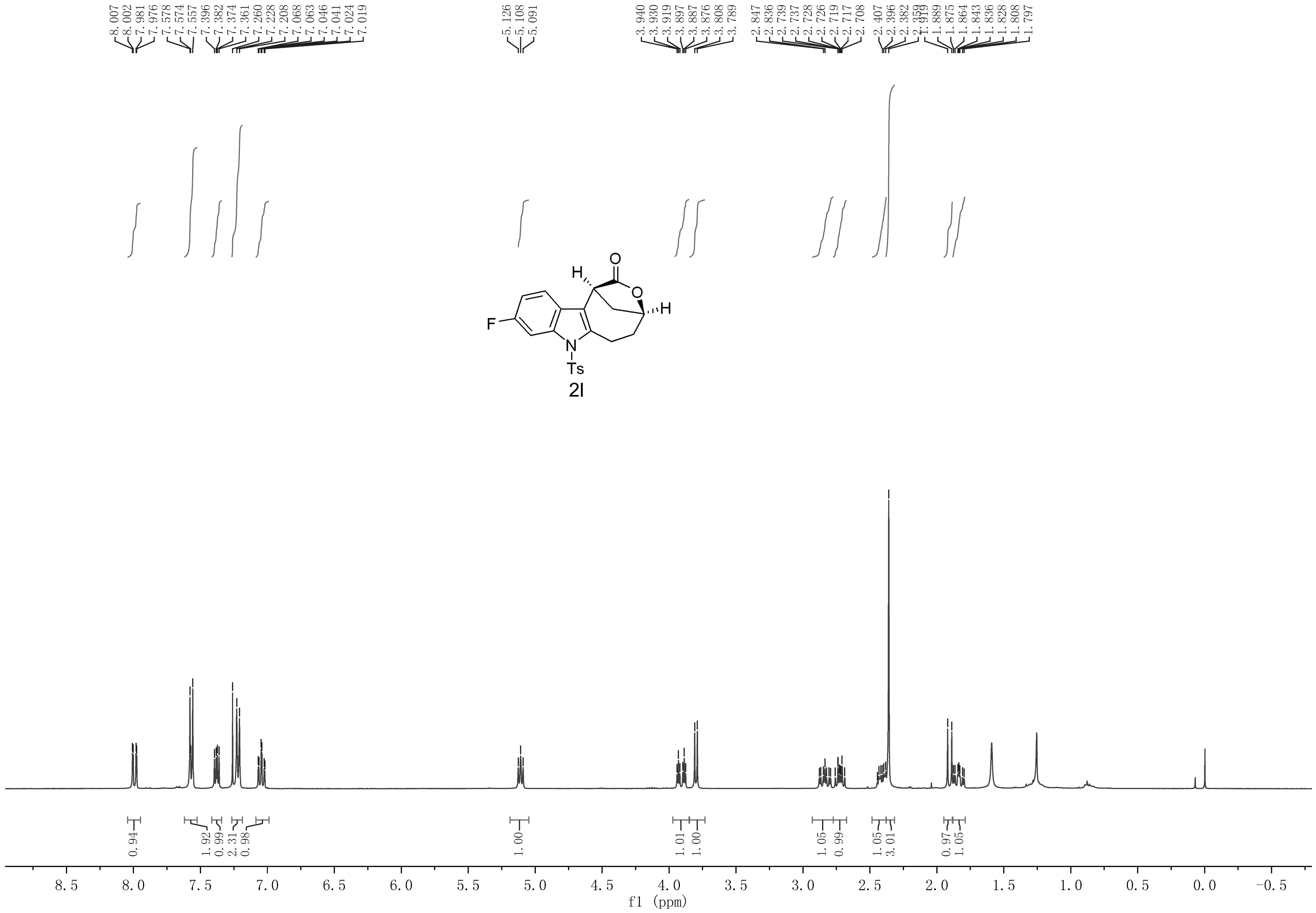

8.007
8.002
7.981
7.976
7.578
7.574
7.557
7.396
7.382
7.374
7.361
7.260
7.228
7.208
7.068
7.063
7.046
7.041
7.024
7.019
5.126
5.108
5.091
3.940
3.930
3.919
3.897
3.887
3.876
3.808
3.789
2.847
2.836
2.739
2.737
2.728
2.726
2.719
2.717
2.708
2.407
2.396
2.382
1.889
1.875
1.864
1.843
1.836
1.828
1.808
1.797
F
N
Ts
O
H
2l
0.94
1.92
0.99
2.31
0.98
1.00
1.01
1.00
1.05
0.99
1.05
3.01
0.97
1.05
8.5
8.0
7.5
7.0
6.5
6.0
5.5
5.0
4.5
4.0
3.5
3.0
2.5
2.0
1.5
1.0
0.5
0.0
−0.5
f1 (ppm)

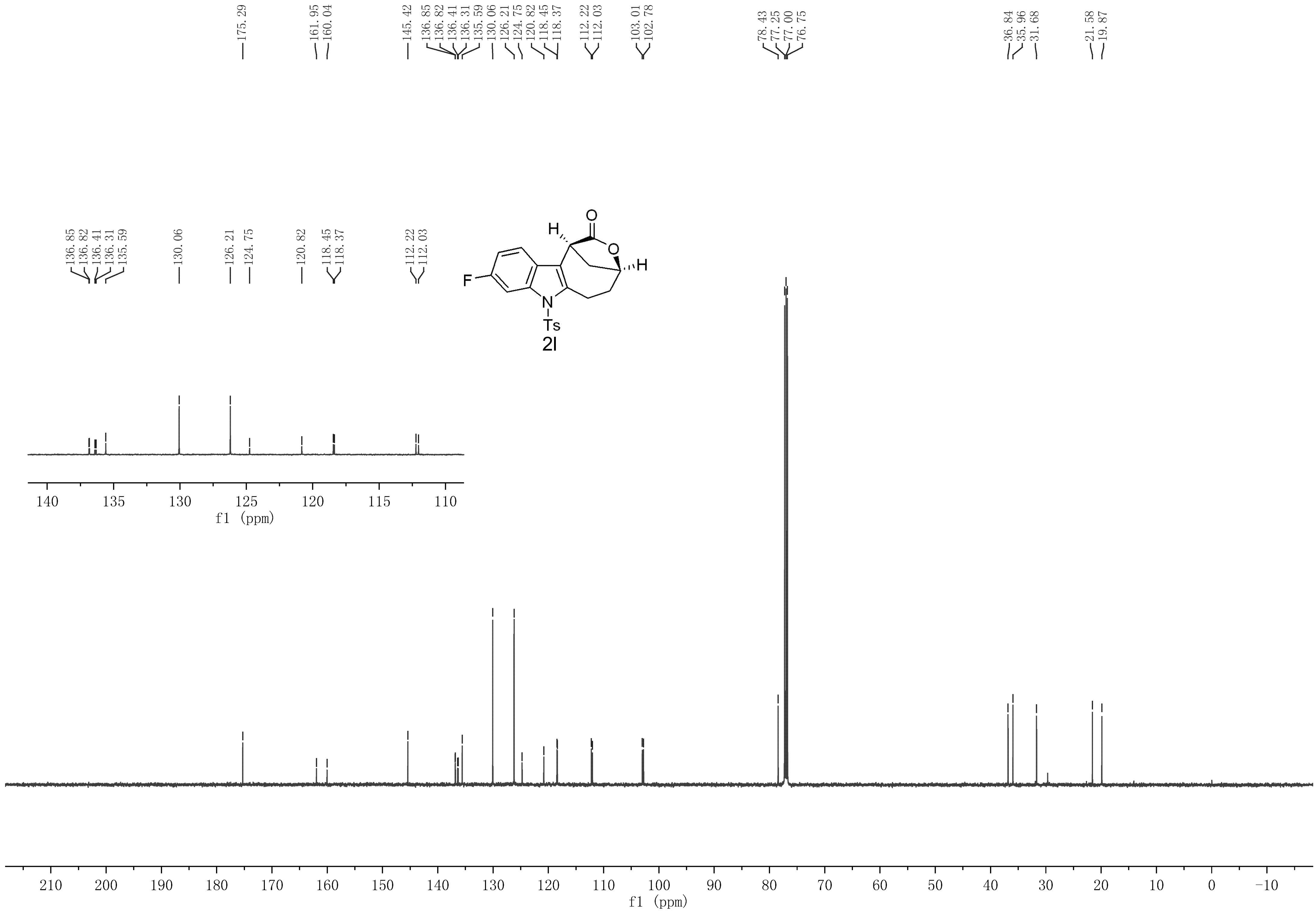
175.29
161.95
160.04
145.42
136.85
136.82
136.41
136.31
135.59
130.06
126.21
124.75
120.82
118.45
118.37
112.22
112.03
103.01
102.78
78.43
77.25
77.00
76.75
36.84
35.96
31.68
21.58
19.87
H
O
O
H
F
N
Ts
2l
140 135 130 125 120 115 110
f1 (ppm)
210 200 190 180 170 160 150 140 130 120 110 100 90 80 70 60 50 40 30 20 10 0 −10
f1 (ppm)

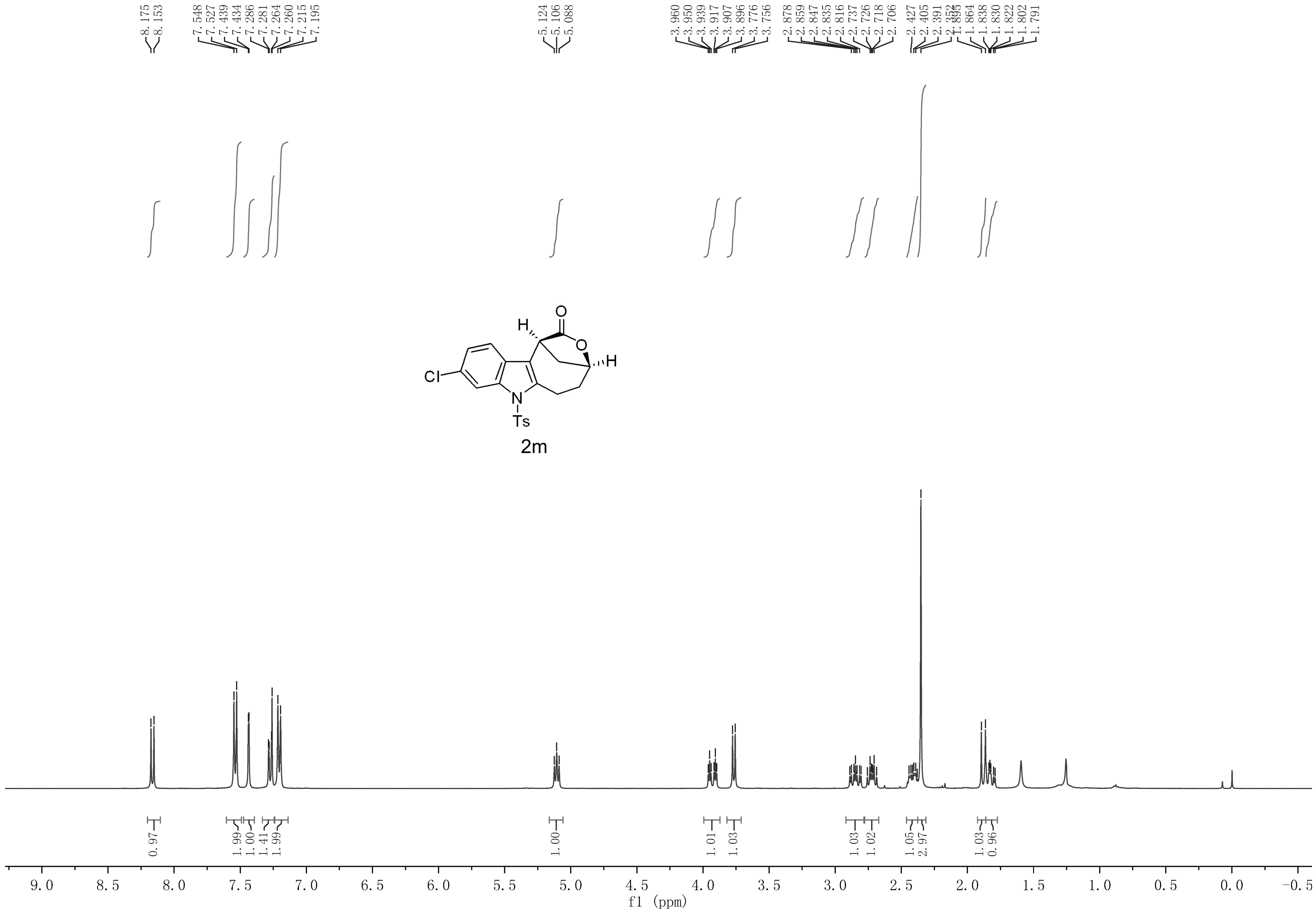
8.175
8.153
7.548
7.527
7.439
7.434
7.286
7.281
7.264
7.260
7.215
7.195
5.124
5.106
5.088
3.960
3.950
3.939
3.917
3.907
3.896
3.776
3.756
2.878
2.859
2.847
2.835
2.816
2.737
2.726
2.718
2.706
2.427
2.405
2.391
2.352
1.895
1.864
1.838
1.830
1.822
1.802
1.791
H
O
O
H
Cl
N
Ts
2m
0.97
1.99
1.00
1.41
1.99
1.00
1.01
1.03
1.03
1.02
1.05
2.97
1.03
0.96
9.0
8.5
8.0
7.5
7.0
6.5
6.0
5.5
5.0
4.5
4.0
3.5
3.0
2.5
2.0
1.5
1.0
0.5
0.0
-0.5
f1 (ppm)

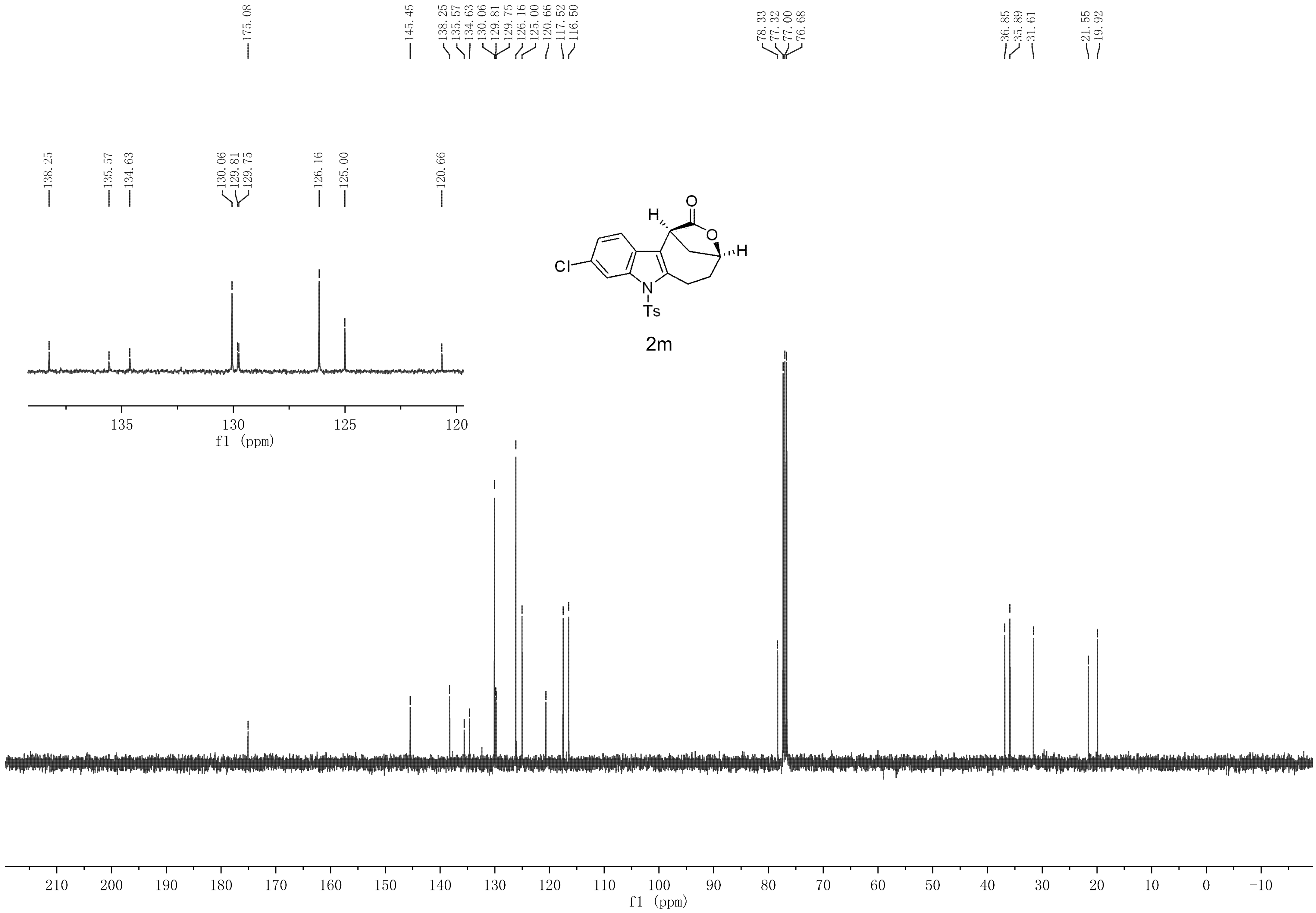
175.08
145.45
138.25
135.57
134.63
130.06
129.81
129.75
126.16
125.00
120.66
117.52
116.50
78.33
77.32
77.00
76.68
36.85
35.89
31.61
21.55
19.92
Cl
N
Ts
H
O
2m
f1 (ppm)

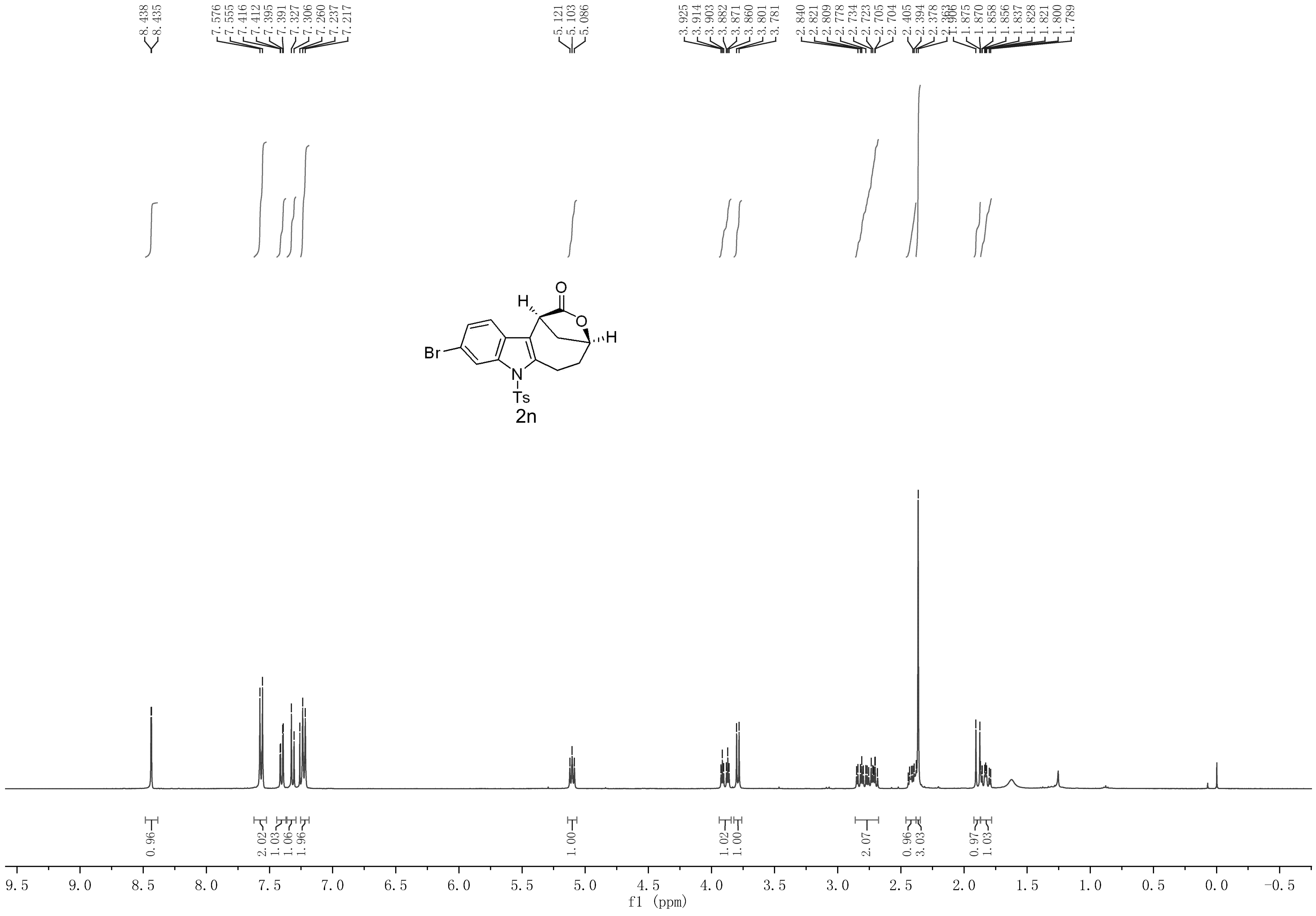

8.438
8.435
7.576
7.555
7.416
7.412
7.395
7.391
7.327
7.306
7.260
7.237
7.217
5.121
5.103
5.086
3.925
3.914
3.903
3.882
3.871
3.860
3.801
3.781
2.840
2.821
2.809
2.778
2.734
2.723
2.705
2.704
2.405
2.394
2.378
2.363
1.906
1.875
1.870
1.858
1.856
1.837
1.828
1.821
1.800
1.789
O
H
O
H
Br
N
Ts
2n
0.96
2.02
1.03
1.06
1.96
1.00
1.02
1.00
2.07
0.96
3.03
0.97
1.03
9.5
9.0
8.5
8.0
7.5
7.0
6.5
6.0
5.5
5.0
4.5
4.0
3.5
3.0
2.5
2.0
1.5
1.0
0.5
0.0
-0.5
f1 (ppm)

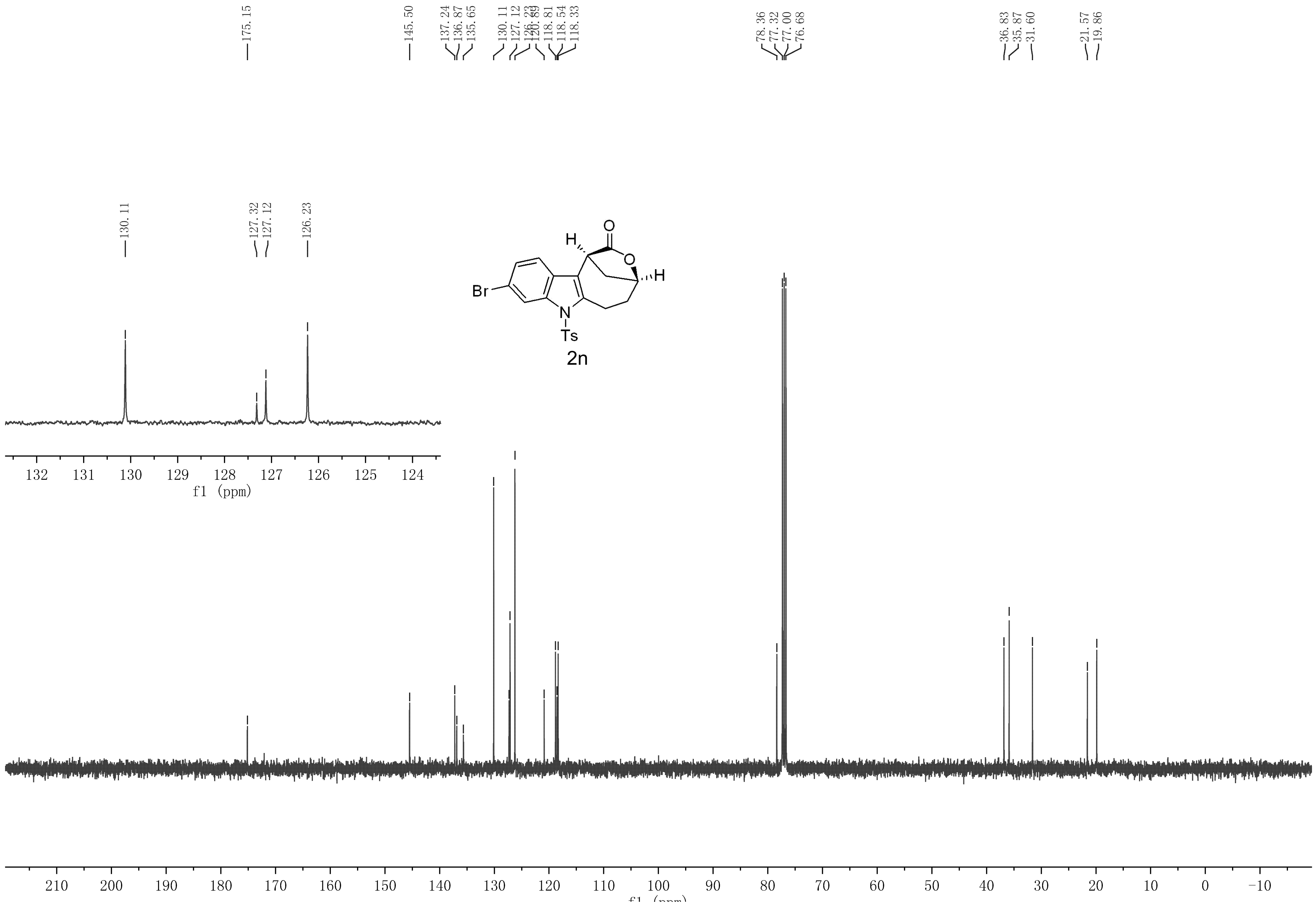

175.15
145.50
137.24
136.87
135.65
130.11
127.12
126.23
120.89
118.81
118.54
118.33
78.36
77.32
77.00
76.68
36.83
35.87
31.60
21.57
19.86
130.11
127.32
127.12
126.23
132 131 130 129 128 127 126 125 124
f1 (ppm)
H
O
O
H
Br
N
Ts
2n
210 200 190 180 170 160 150 140 130 120 110 100 90 80 70 60 50 40 30 20 10 0 −10

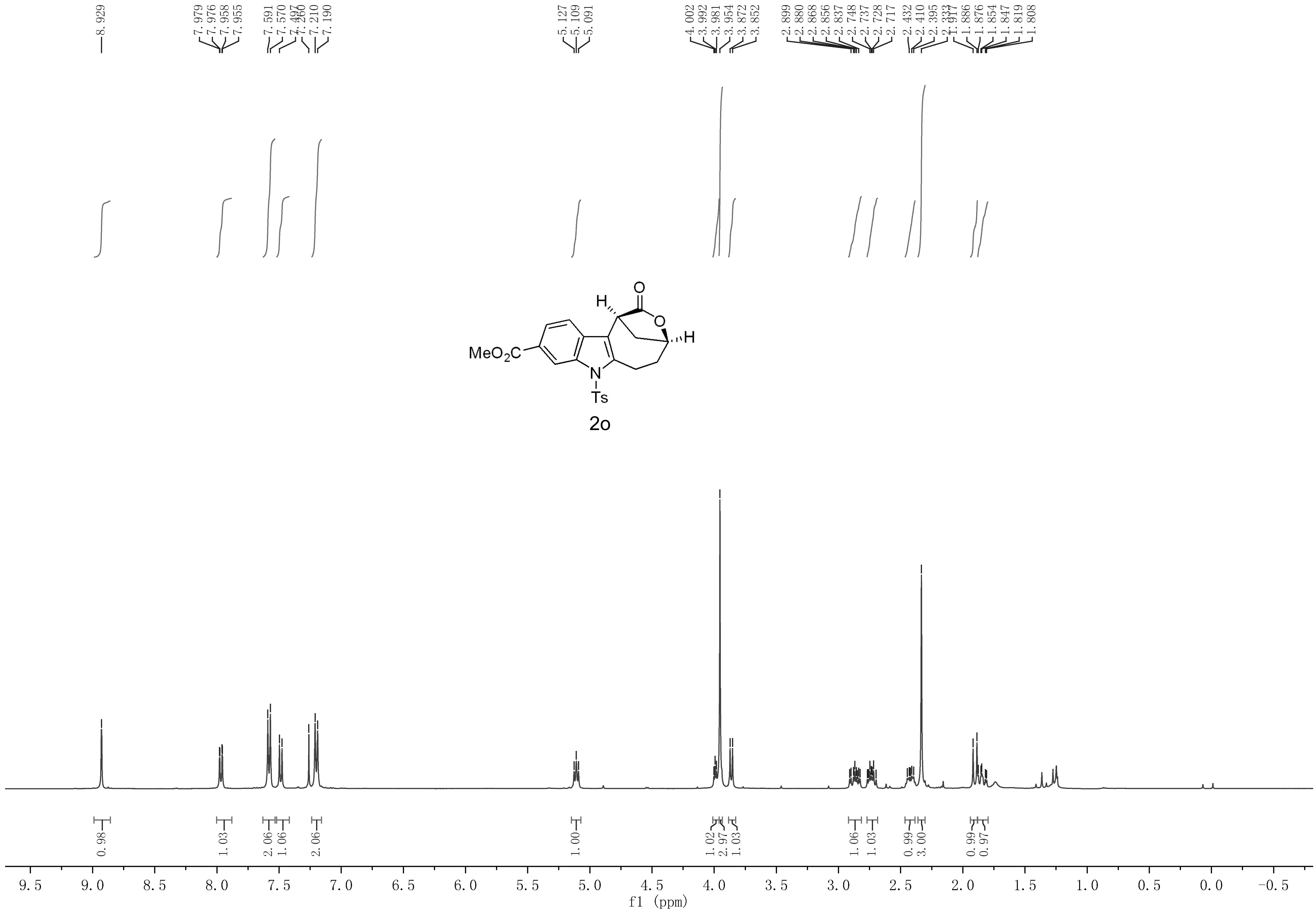
8.929
7.979
7.976
7.958
7.955
7.591
7.570
7.497
7.260
7.210
7.190
5.127
5.109
5.091
4.002
3.992
3.981
3.954
3.872
3.852
2.899
2.880
2.868
2.856
2.837
2.748
2.737
2.728
2.717
2.432
2.410
2.395
2.333
1.917
1.886
1.876
1.854
1.847
1.819
1.808
H
O
O
H
MeO2C
N
Ts
2o
0.98
1.03
2.06
1.06
2.06
1.00
1.02
2.97
1.03
1.06
1.03
0.99
3.00
0.99
0.97
9.5 9.0 8.5 8.0 7.5 7.0 6.5 6.0 5.5 5.0 4.5 4.0 3.5 3.0 2.5 2.0 1.5 1.0 0.5 0.0 -0.5
f1 (ppm)

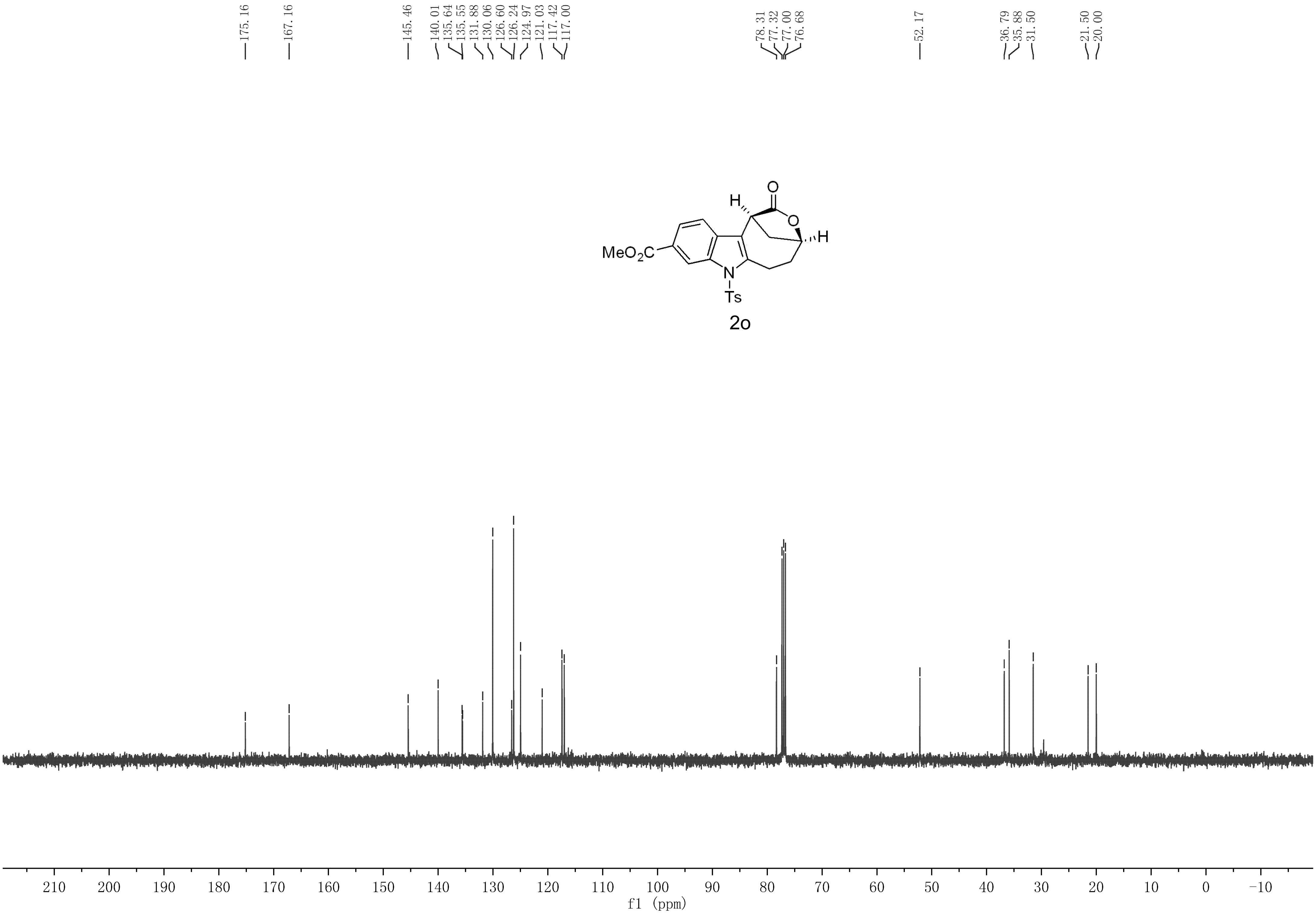
175.16
167.16
145.46
140.01
135.64
135.55
131.88
130.06
126.60
126.24
124.97
121.03
117.42
117.00
78.31
77.32
77.00
76.68
52.17
36.79
35.88
31.50
21.50
20.00
MeO2C
N
Ts
H
O
O
H
210 200 190 180 170 160 150 140 130 120 110 100 90 80 70 60 50 40 30 20 10 0 −10
f1 (ppm)

2o

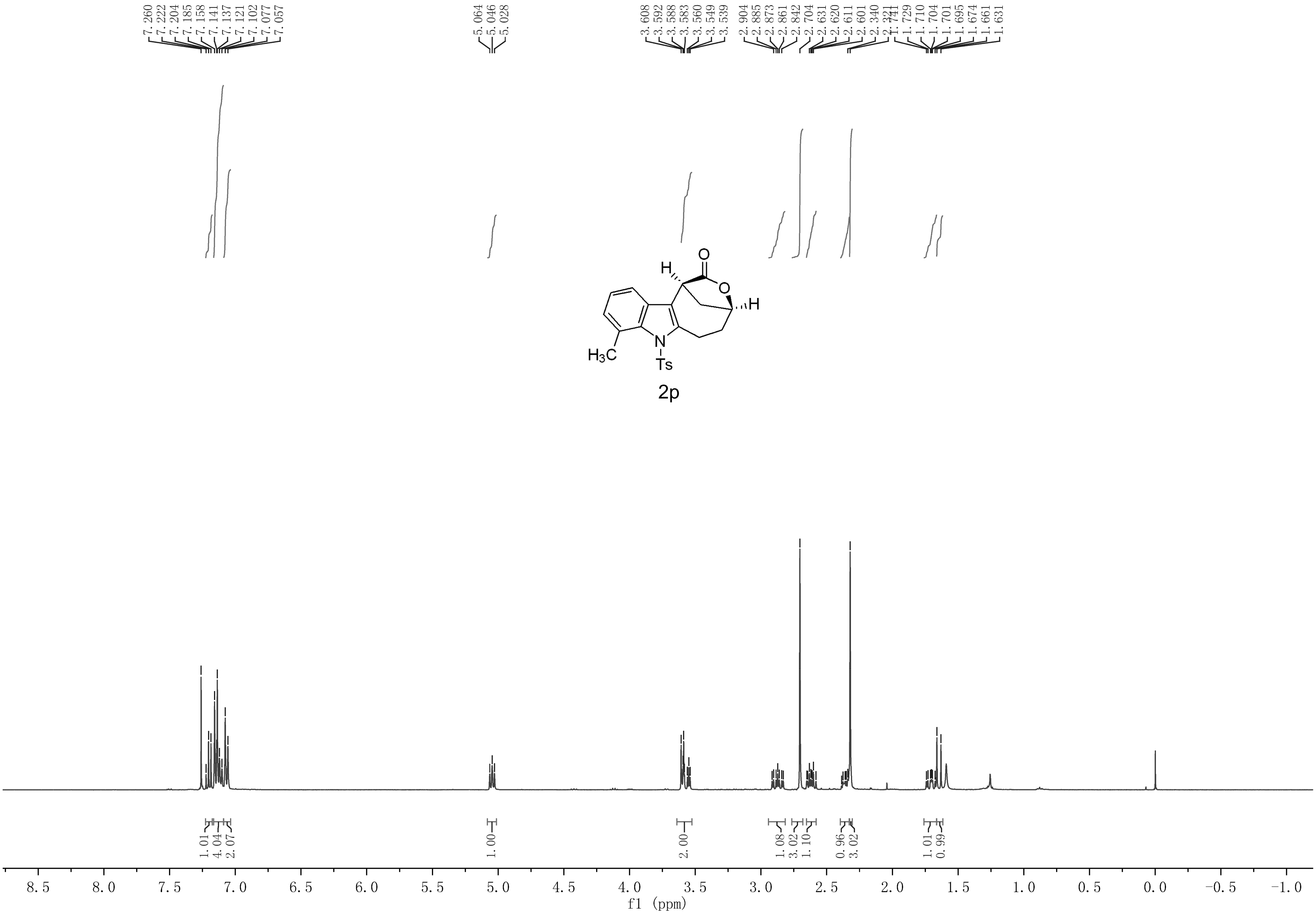
7.260
7.222
7.204
7.185
7.158
7.141
7.137
7.121
7.102
7.077
7.057
5.064
5.046
5.028
3.608
3.592
3.588
3.583
3.560
3.549
3.539
2.904
2.885
2.873
2.861
2.842
2.704
2.631
2.620
2.611
2.601
2.340
2.321
1.741
1.729
1.710
1.704
1.701
1.695
1.674
1.661
1.631
O
H
O
H
H_3C
N
Ts
2p
1.01
4.04
2.07
1.00
2.00
1.08
3.02
1.10
0.96
3.02
1.01
0.99
8.5
8.0
7.5
7.0
6.5
6.0
5.5
5.0
4.5
4.0
3.5
3.0
2.5
2.0
1.5
1.0
0.5
0.0
−0.5
−1.0
f1 (ppm)

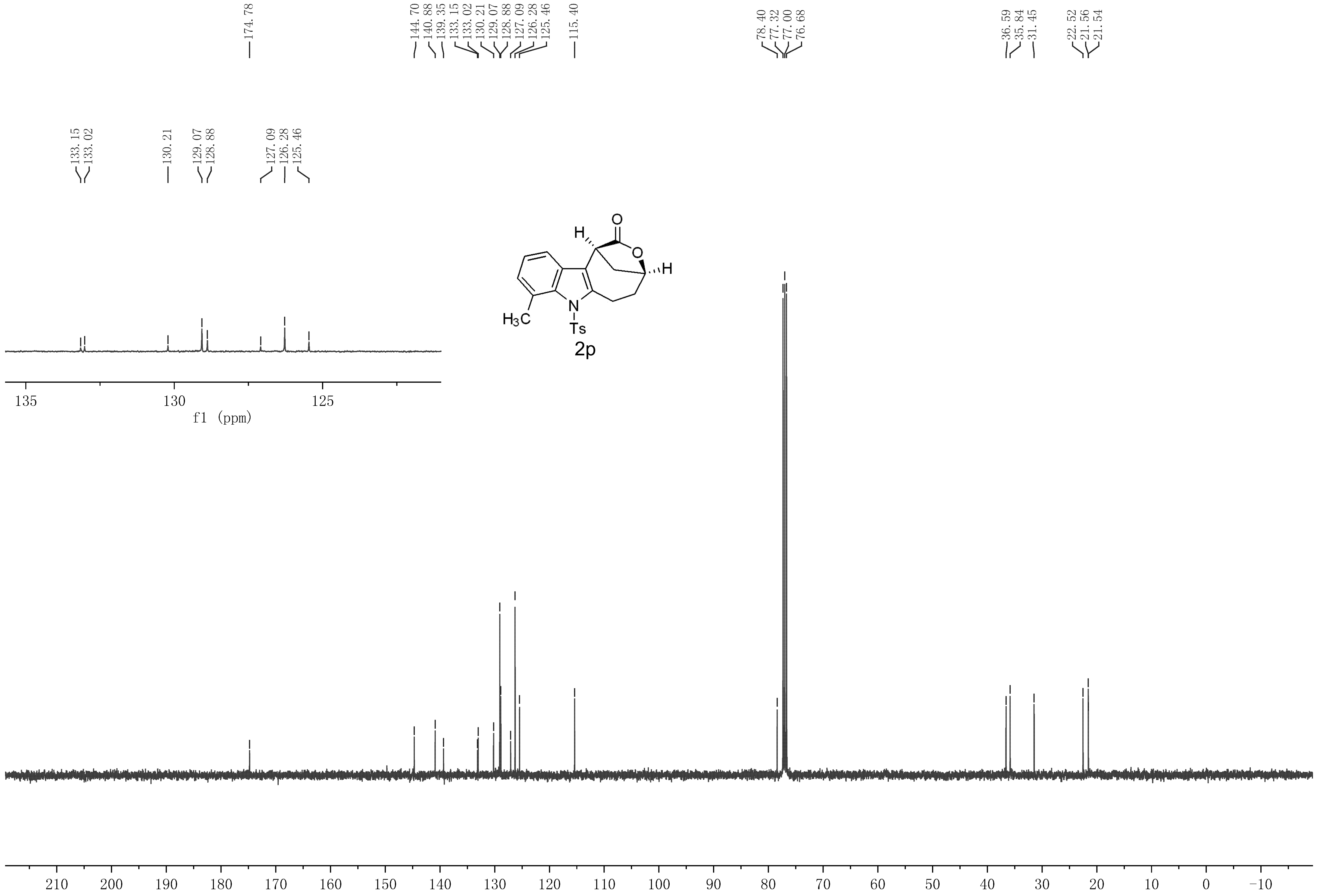
174.78
144.70
140.88
139.35
133.15
133.02
130.21
129.07
128.88
127.09
126.28
125.46
115.40
78.40
77.32
77.00
76.68
36.59
35.84
31.45
22.52
21.56
21.54
133.15
133.02
130.21
129.07
128.88
127.09
126.28
125.46
135
130
125
f1 (ppm)
H
O
O
H
H3C
N
Ts
2p
210
200
190
180
170
160
150
140
130
120
110
100
90
80
70
60
50
40
30
20
10
0
−10

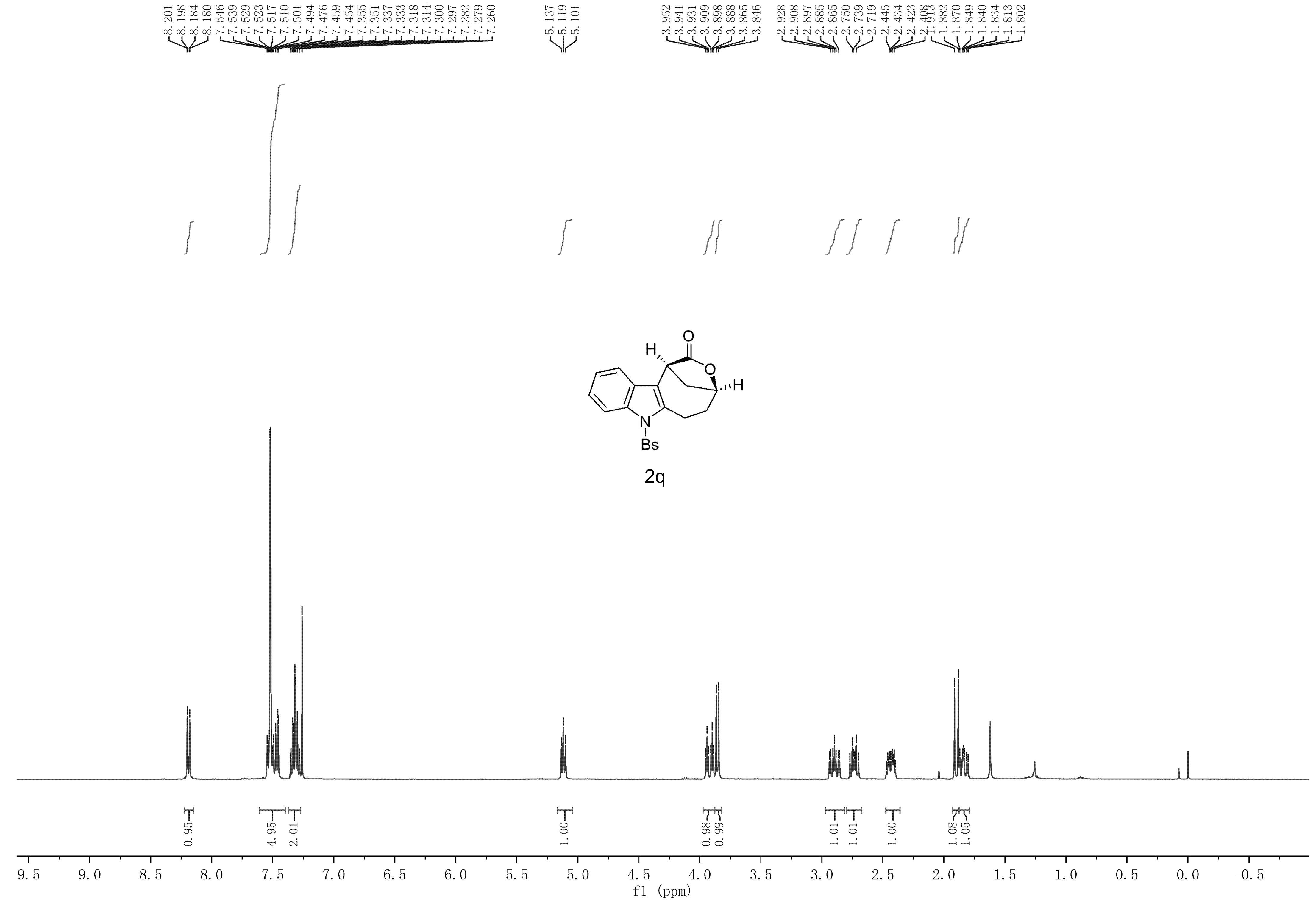
8.201
8.198
8.184
8.180
7.546
7.539
7.529
7.523
7.517
7.510
7.501
7.494
7.476
7.459
7.454
7.355
7.351
7.337
7.333
7.318
7.314
7.300
7.297
7.282
7.279
7.260
5.137
5.119
5.101
3.952
3.941
3.931
3.909
3.898
3.888
3.865
3.846
2.928
2.908
2.897
2.885
2.865
2.750
2.739
2.719
2.445
2.434
2.423
1.913
1.882
1.870
1.849
1.840
1.834
1.813
1.802
O
H
H
N
Bs
2q
0.95
4.95
2.01
1.00
0.98
0.99
1.01
1.01
1.00
1.08
1.05
9.5
9.0
8.5
8.0
7.5
7.0
6.5
6.0
5.5
5.0
4.5
4.0
3.5
3.0
2.5
2.0
1.5
1.0
0.5
0.0
-0.5
f1 (ppm)

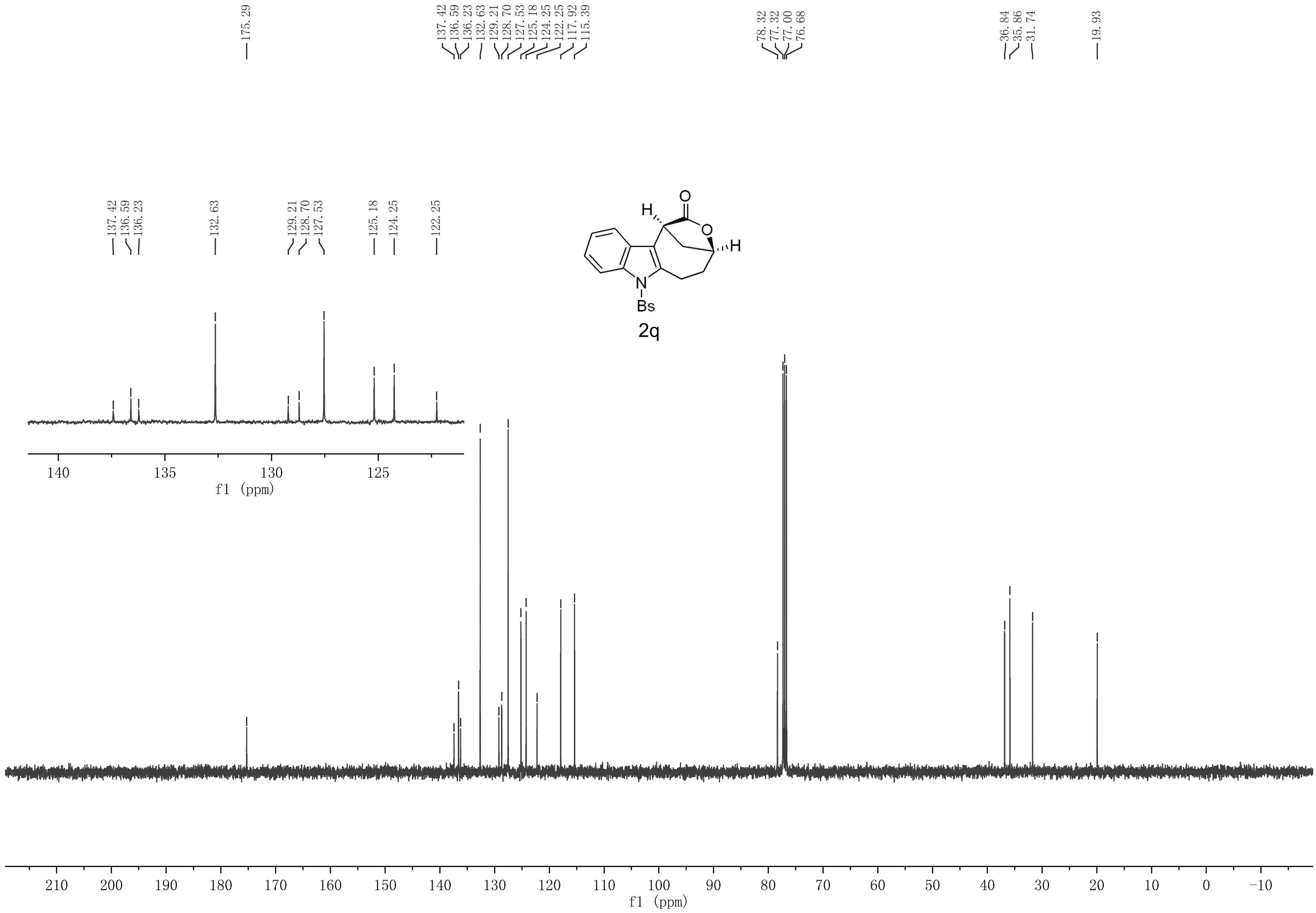
175.29
137.42
136.59
136.23
132.63
129.21
128.70
127.53
125.18
124.25
122.25
117.92
115.39
78.32
77.32
77.00
76.68
36.84
35.86
31.74
19.93
2q
f1 (ppm)

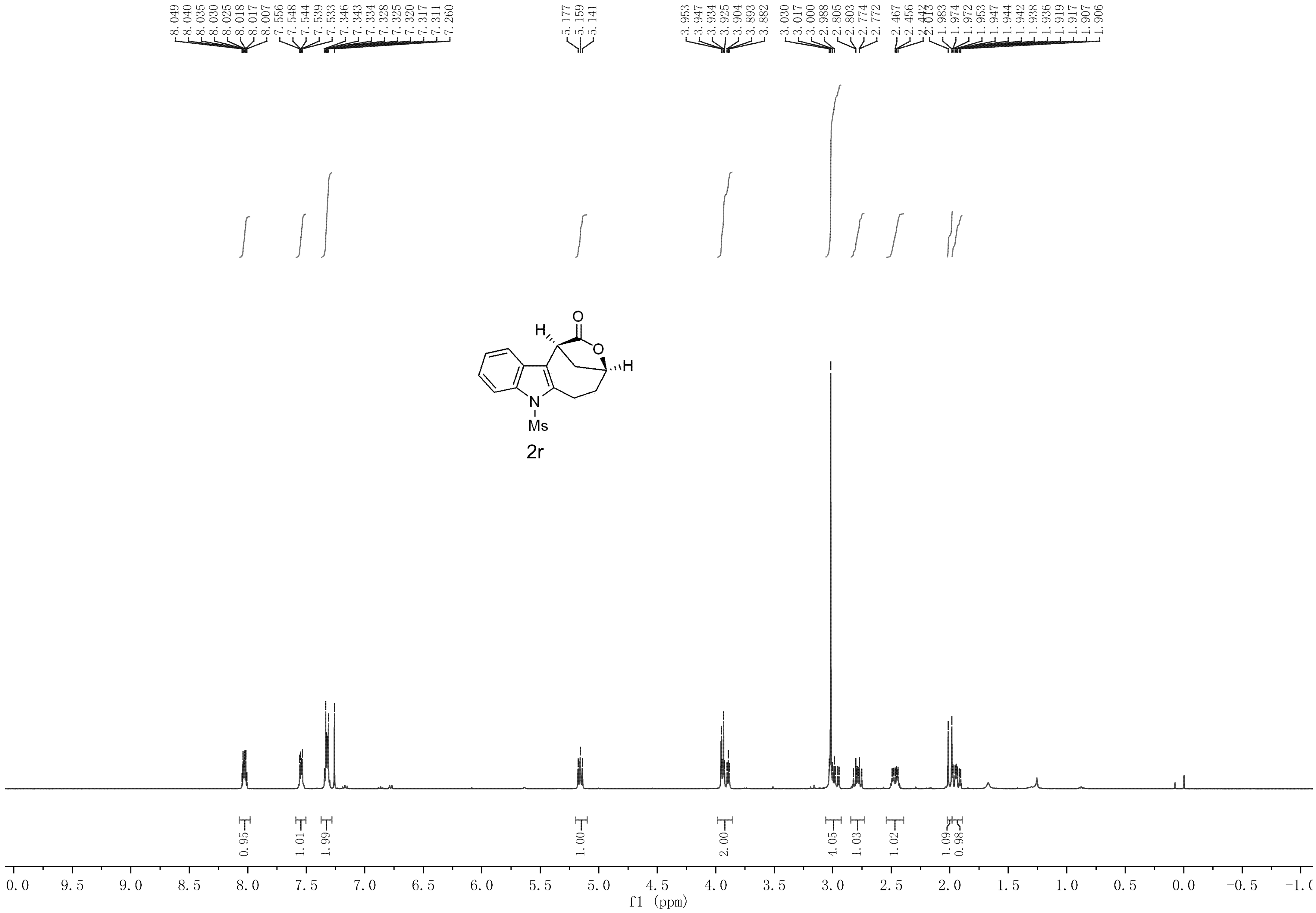
8.049
8.040
8.035
8.030
8.025
8.018
8.017
8.007
7.556
7.548
7.544
7.539
7.533
7.346
7.343
7.334
7.328
7.325
7.320
7.317
7.311
7.260
5.177
5.159
5.141
3.953
3.947
3.934
3.925
3.904
3.893
3.882
3.030
3.017
3.000
2.988
2.805
2.803
2.774
2.772
2.467
2.456
1.983
1.974
1.972
1.953
1.947
1.944
1.942
1.938
1.936
1.919
1.917
1.907
1.906
O
H
N
Ms
2r
0.95
1.01
1.99
1.00
2.00
4.05
1.03
1.02
1.09
0.98
9.5
9.0
8.5
8.0
7.5
7.0
6.5
6.0
5.5
5.0
4.5
4.0
3.5
3.0
2.5
2.0
1.5
1.0
0.5
0.0
-0.5
f1 (ppm)

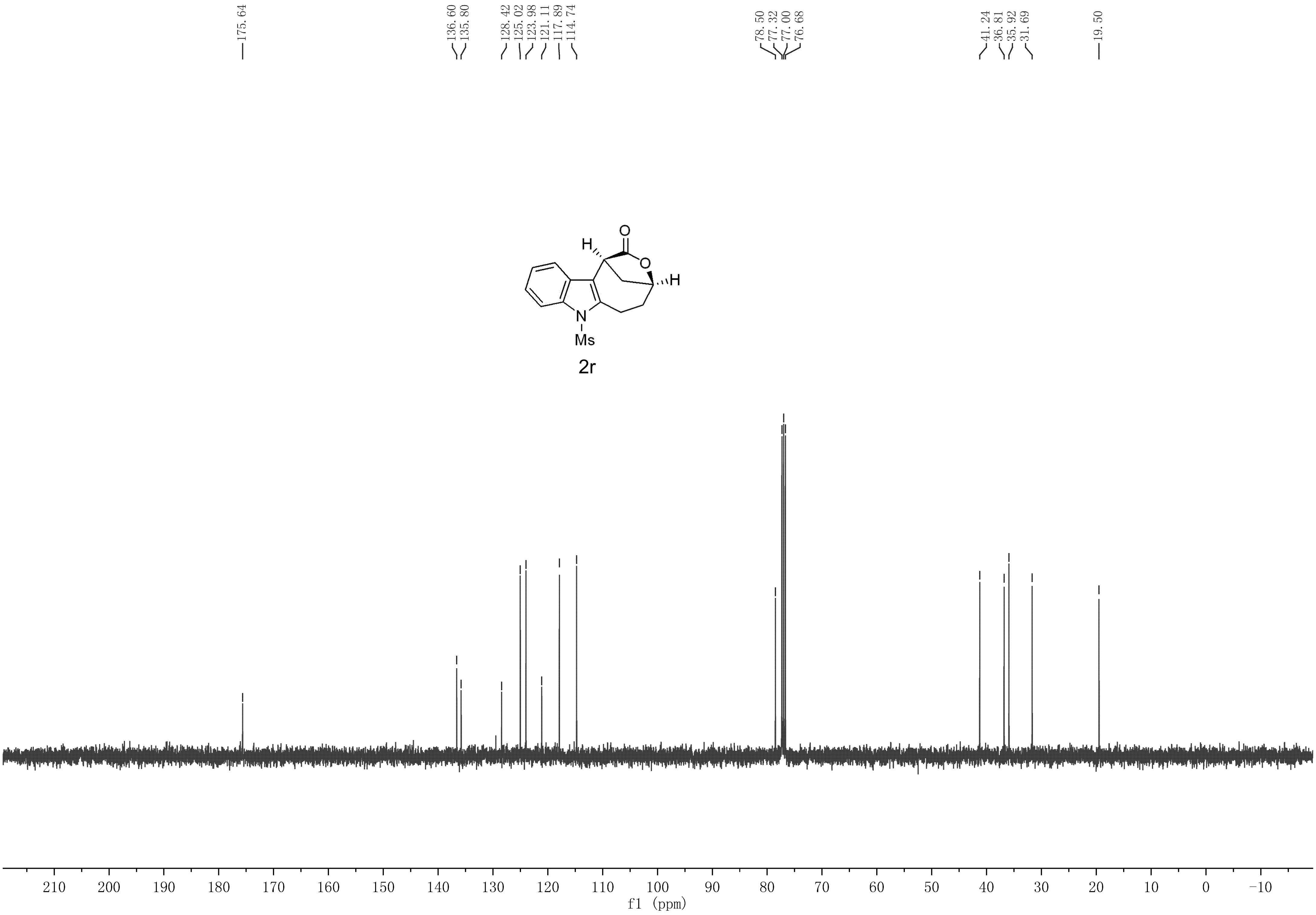
175.64
136.60
135.80
128.42
125.02
123.98
121.11
117.89
114.74
78.50
77.32
77.00
76.68
41.24
36.81
35.92
31.69
19.50
H
O
O
H
N
Ms
2r
210 200 190 180 170 160 150 140 130 120 110 100 90 80 70 60 50 40 30 20 10 0 −10
f1 (ppm)

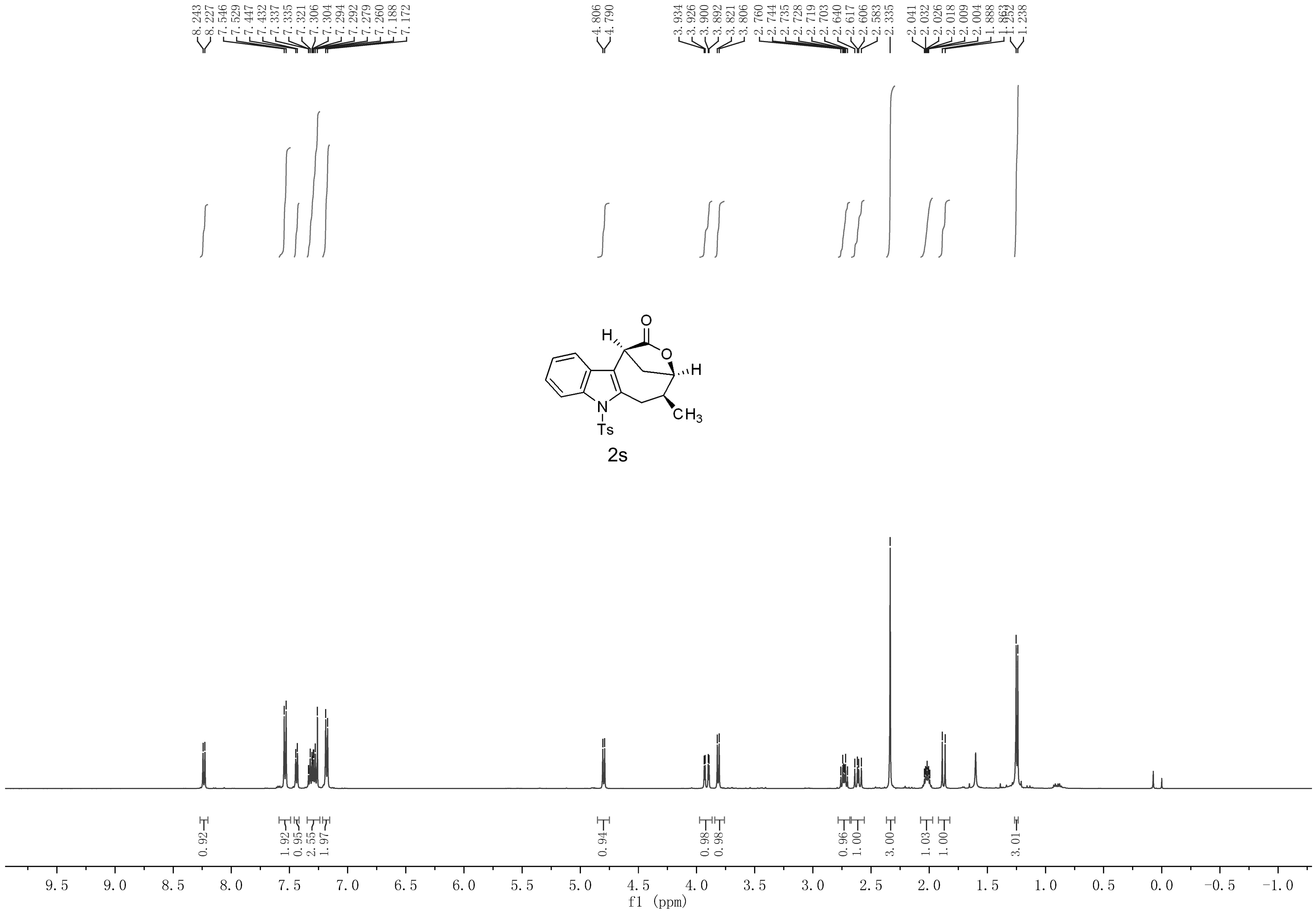
8.243
8.227
7.546
7.529
7.447
7.432
7.337
7.335
7.321
7.306
7.304
7.294
7.292
7.279
7.260
7.188
7.172
4.806
4.790
3.934
3.926
3.900
3.892
3.821
3.806
2.760
2.744
2.735
2.728
2.719
2.703
2.640
2.617
2.606
2.583
2.335
2.041
2.032
2.026
2.018
2.009
2.004
1.888
1.862
1.252
1.238
H
O
H
N
Ts
CH3
2s
0.92
1.92
0.95
2.55
1.97
0.94
0.98
0.98
0.96
1.00
3.00
1.03
1.00
3.01
9.5 9.0 8.5 8.0 7.5 7.0 6.5 6.0 5.5 5.0 4.5 4.0 3.5 3.0 2.5 2.0 1.5 1.0 0.5 0.0 -0.5 -1.0
f1 (ppm)

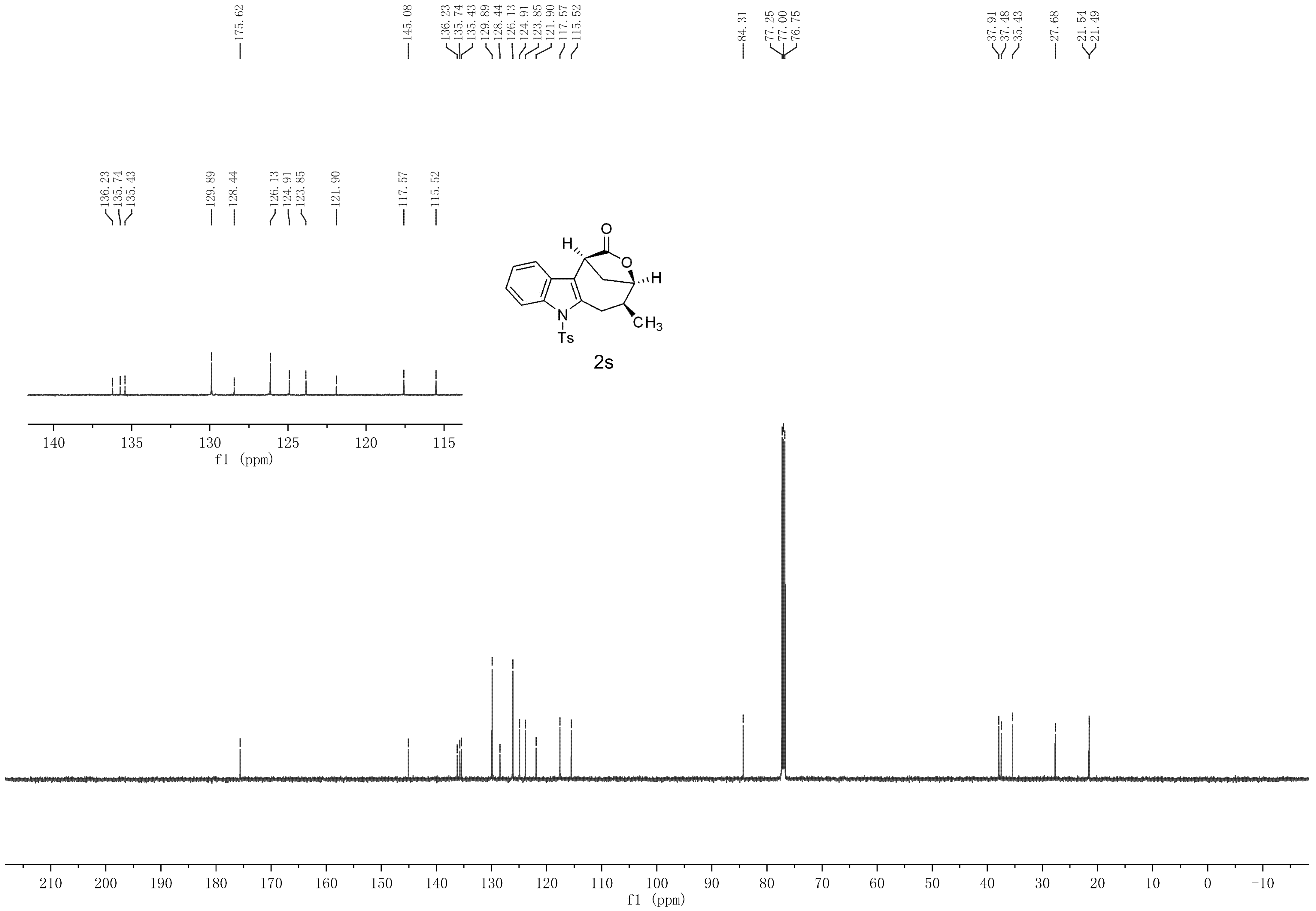

175.62
145.08
136.23
135.74
135.43
129.89
128.44
126.13
124.91
123.85
121.90
117.57
115.52
84.31
77.25
77.00
76.75
37.91
37.48
35.43
27.68
21.54
21.49
2s
f1 (ppm)

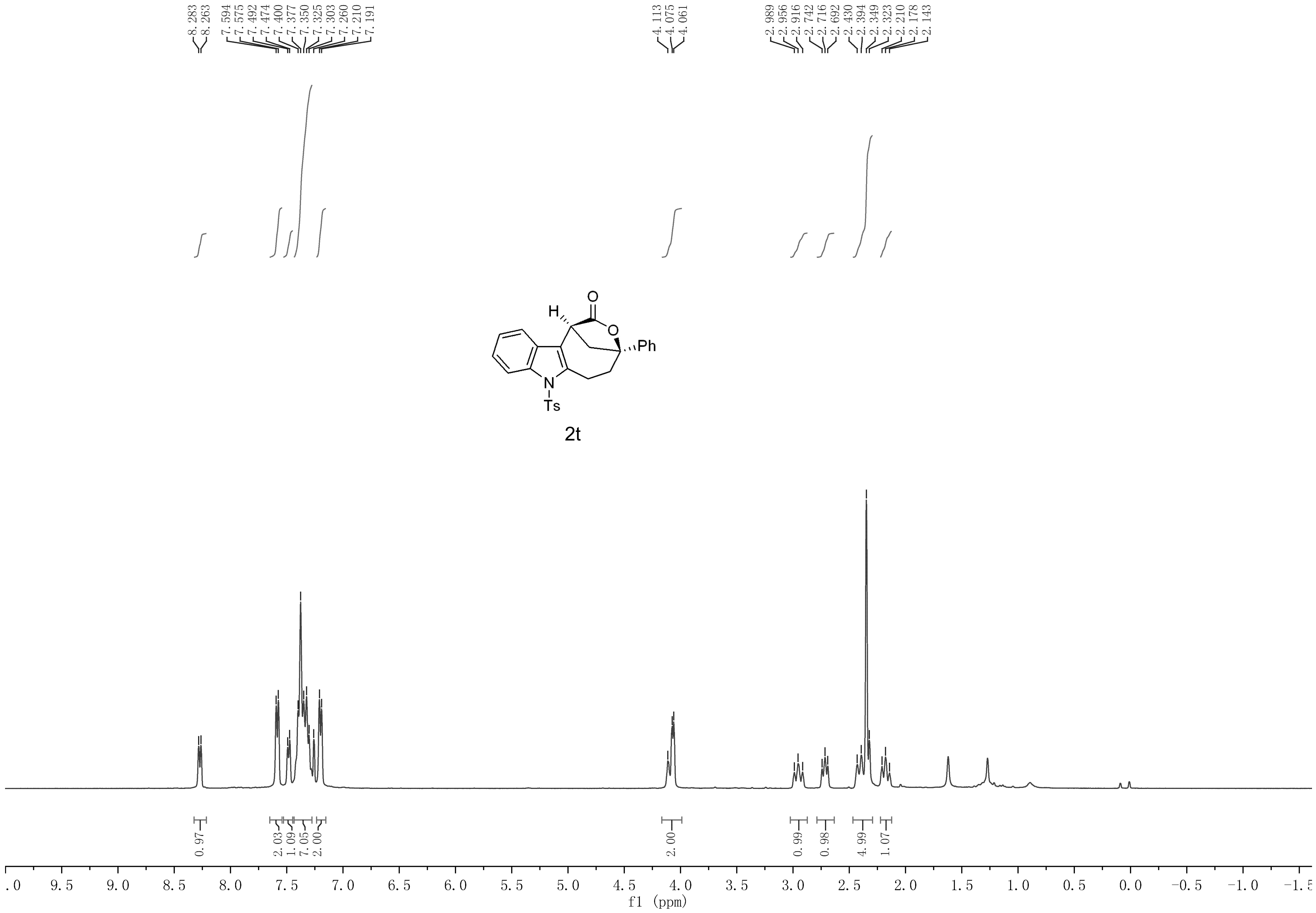

8.283
8.263
7.594
7.575
7.492
7.474
7.400
7.377
7.350
7.325
7.303
7.260
7.210
7.191
4.113
4.075
4.061
2.989
2.956
2.916
2.742
2.716
2.692
2.430
2.394
2.349
2.323
2.210
2.178
2.143
H
O
O
Ph
N
Ts
2t
0.97
2.03
1.09
7.05
2.00
2.00
0.99
0.98
4.99
1.07
.0 9.5 9.0 8.5 8.0 7.5 7.0 6.5 6.0 5.5 5.0 4.5 4.0 3.5 3.0 2.5 2.0 1.5 1.0 0.5 0.0 -0.5 -1.0
f1 (ppm)

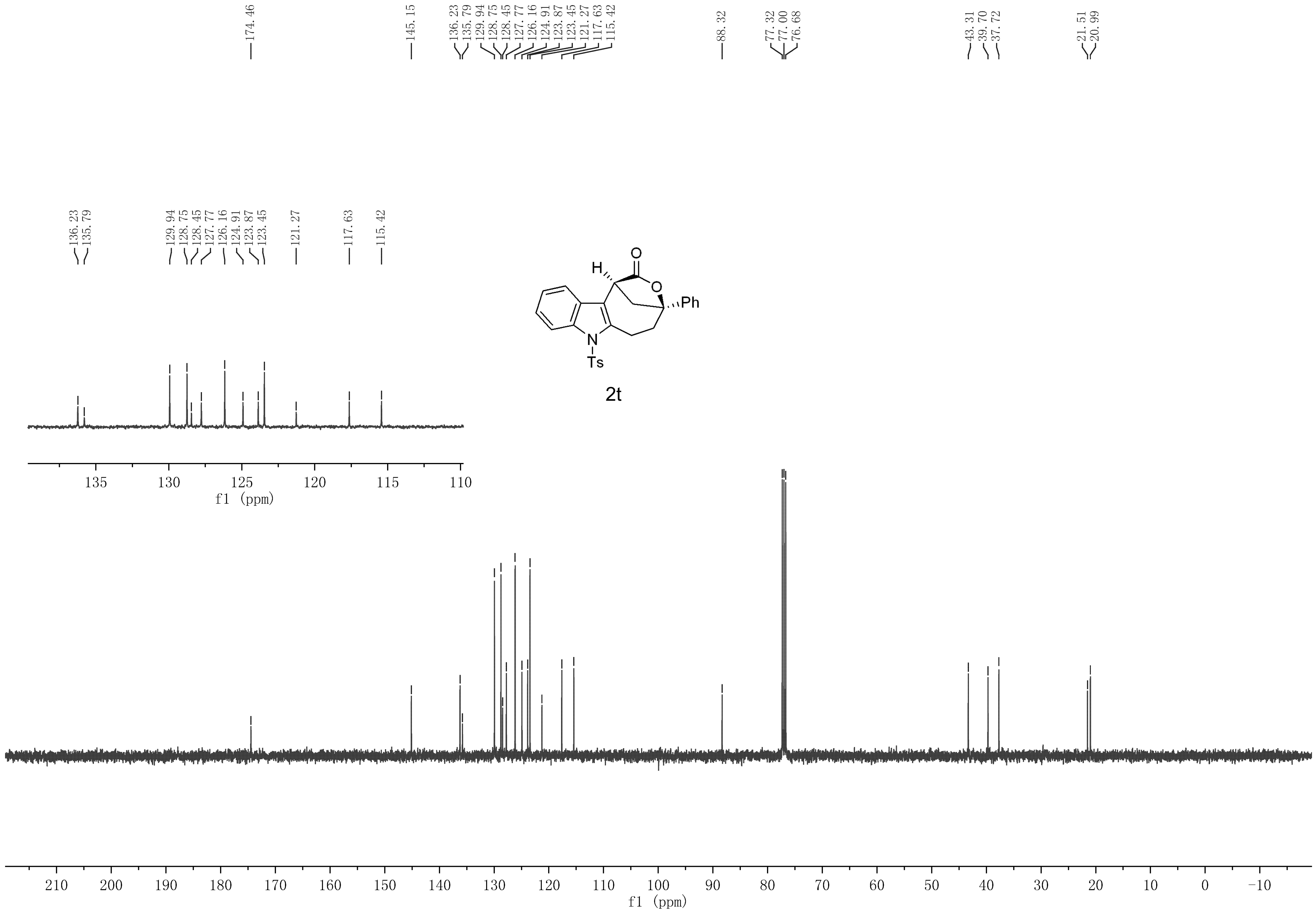

174.46
145.15
136.23
135.79
129.94
128.75
128.45
127.77
126.16
124.91
123.87
123.45
121.27
117.63
115.42
88.32
77.32
77.00
76.68
43.31
39.70
37.72
21.51
20.99
O
H
O
Ph
N
Ts
2t
135
130
125
120
115
110
f1 (ppm)
210
200
190
180
170
160
150
140
130
120
110
100
90
80
70
60
50
40
30
20
10
0
−10
f1 (ppm)

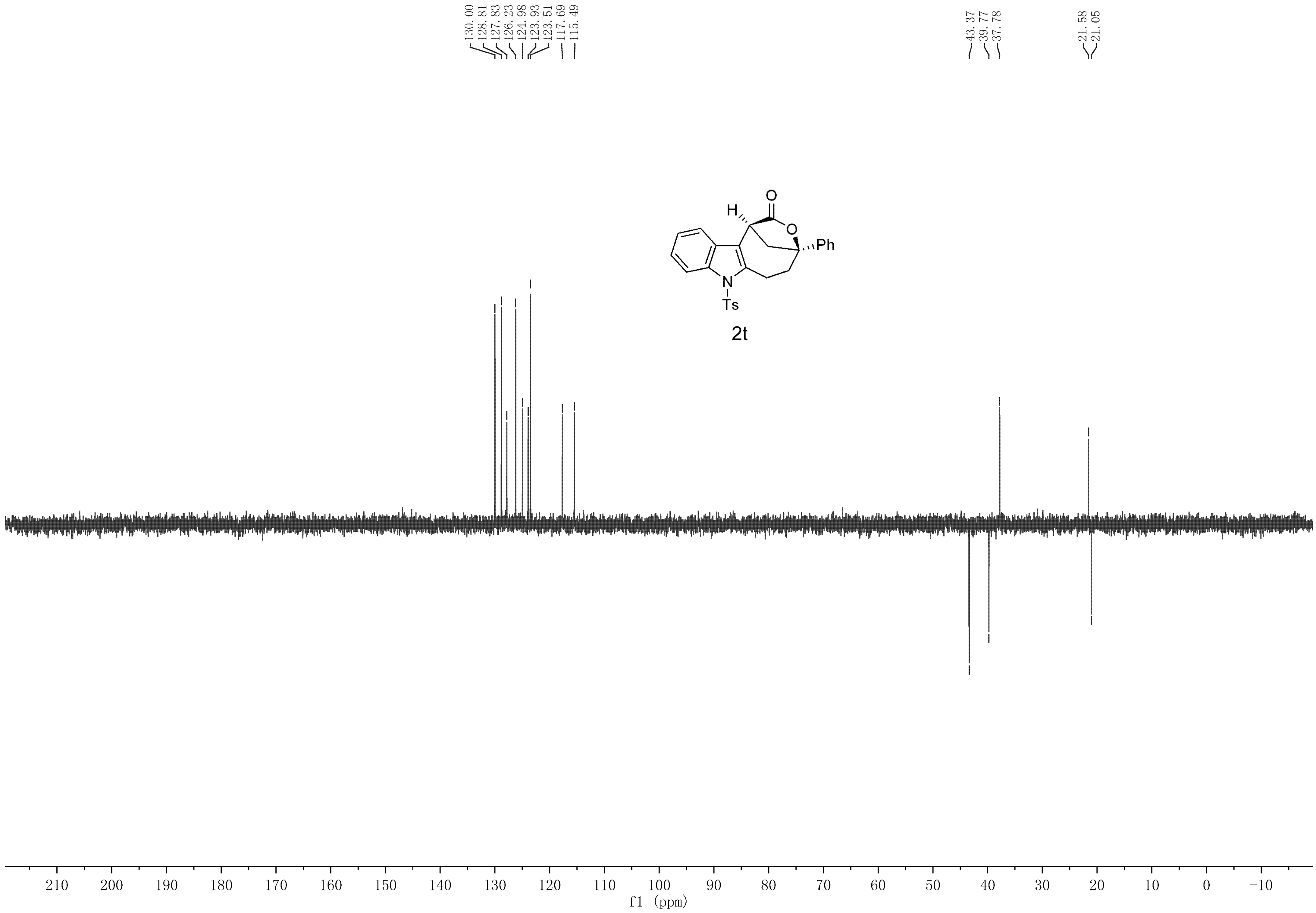
130.00
128.81
127.83
126.23
124.98
123.93
123.51
117.69
115.49
43.37
39.77
37.78
21.58
21.05
H
O
O
Ph
N
Ts
2t
210 200 190 180 170 160 150 140 130 120 110 100 90 80 70 60 50 40 30 20 10 0 −10
f1 (ppm)

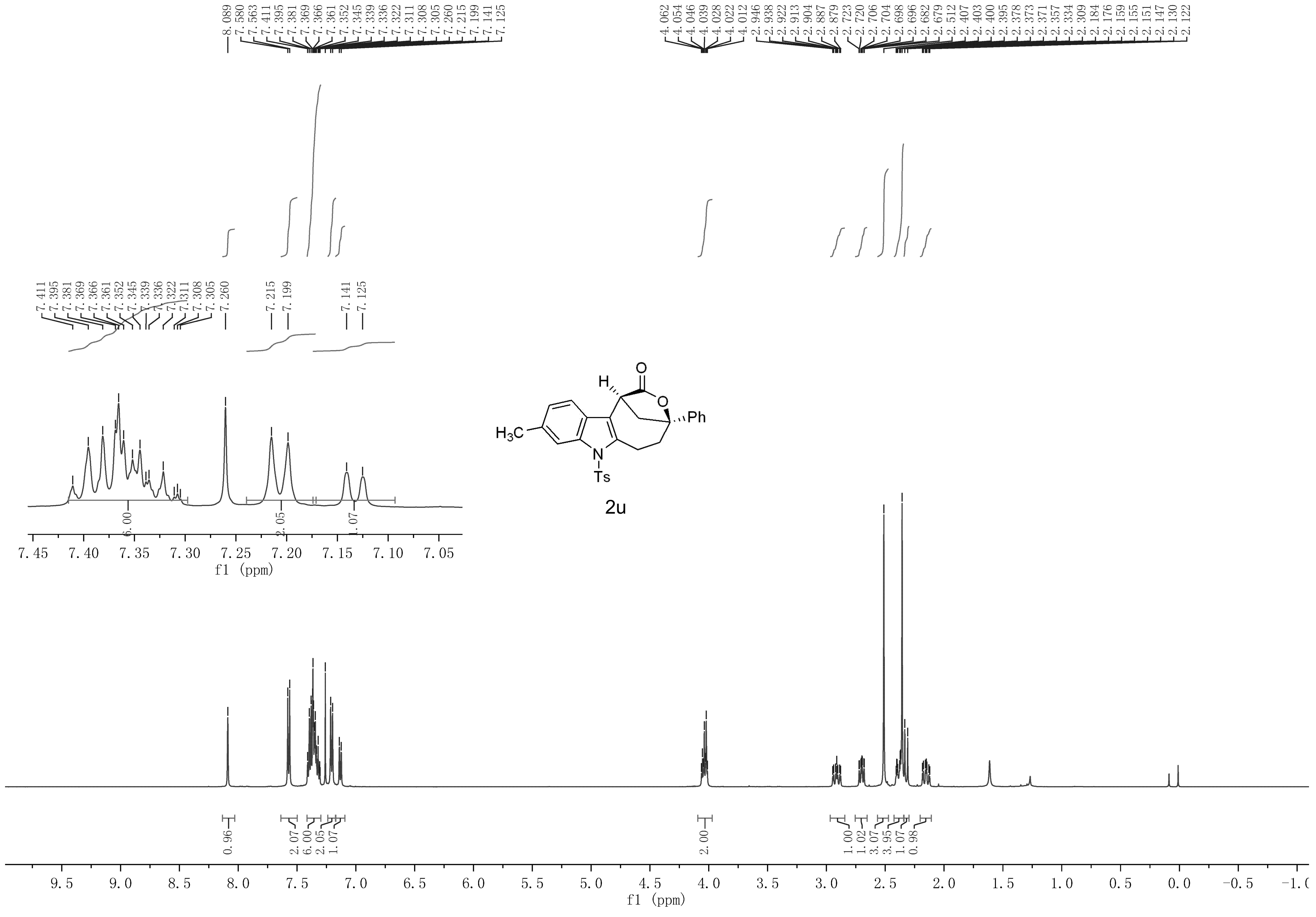
8.089 7.580 7.563 7.411 7.395 7.381 7.369 7.366 7.361 7.352 7.345 7.339 7.336 7.322 7.311 7.308 7.305 7.260 7.215 7.199 7.141 7.125
4.062 4.054 4.046 4.039 4.028 4.022 4.012 2.946 2.938 2.922 2.913 2.904 2.887 2.879 2.723 2.720 2.706 2.704 2.698 2.696 2.682 2.679 2.512 2.407 2.403 2.400 2.395 2.378 2.373 2.371 2.357 2.334 2.309 2.184 2.176 2.159 2.155 2.151 2.147 2.130 2.122
7.411 7.395 7.381 7.369 7.366 7.361 7.352 7.345 7.339 7.336 7.322 7.311 7.308 7.305 7.260 7.215 7.199 7.141 7.125
6.00 2.05 1.07
7.45 7.40 7.35 7.30 7.25 7.20 7.15 7.10 7.05
f1 (ppm)
H
O
O
Ph
H_3C
N
Ts
2u
0.96 2.07 6.00 2.05 1.07 2.00 1.00 1.02 3.07 3.95 1.07 0.98
9.5 9.0 8.5 8.0 7.5 7.0 6.5 6.0 5.5 5.0 4.5 4.0 3.5 3.0 2.5 2.0 1.5 1.0 0.5 0.0 −0.5 −1.0
f1 (ppm)

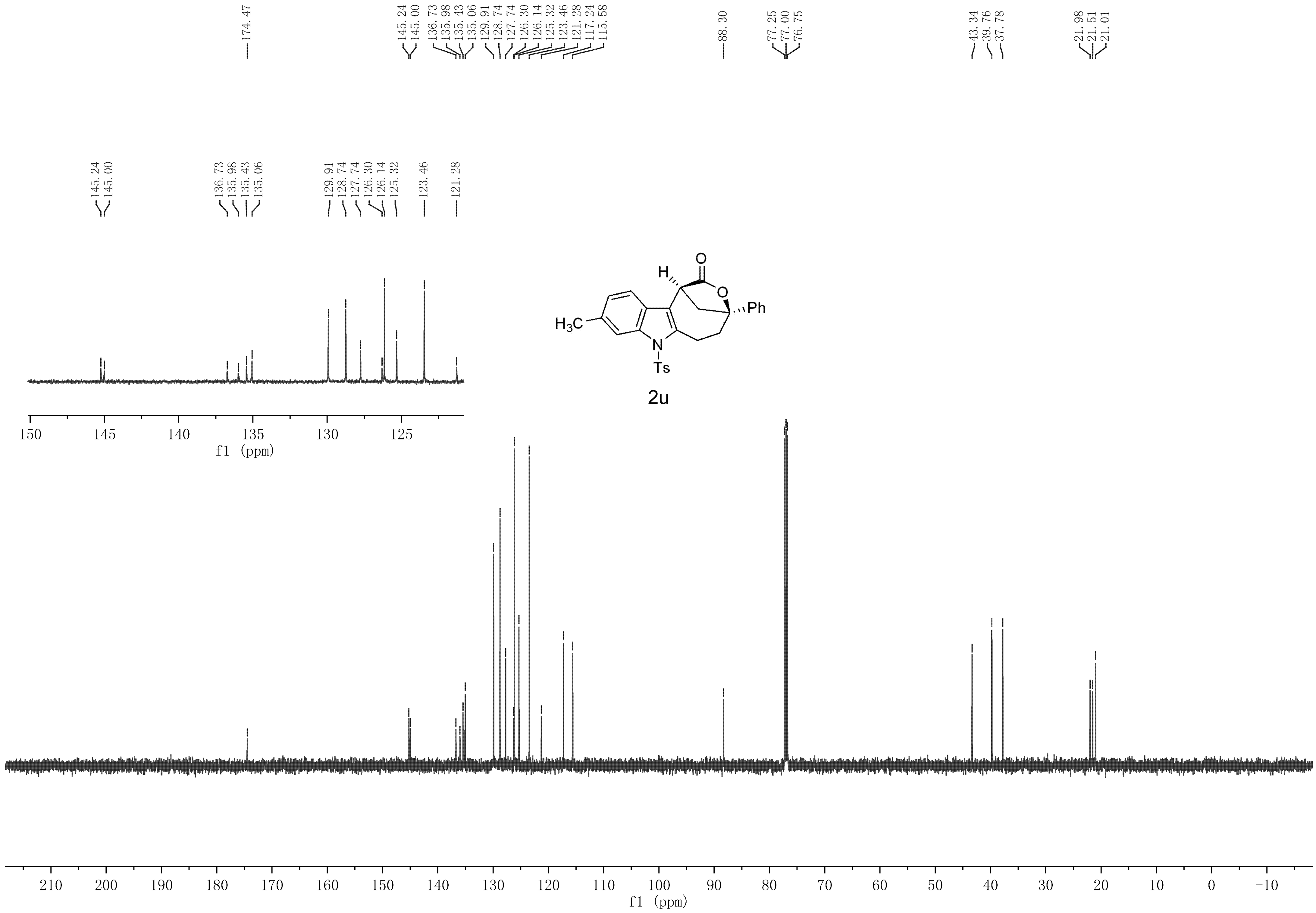
174.47
145.24
145.00
136.73
135.98
135.43
135.06
129.91
128.74
127.74
126.30
126.14
125.32
123.46
121.28
117.24
115.58
88.30
77.25
77.00
76.75
43.34
39.76
37.78
21.98
21.51
21.01
H
O
O
Ph
H3C
N
Ts
2u
150 145 140 135 130 125
f1 (ppm)
210 200 190 180 170 160 150 140 130 120 110 100 90 80 70 60 50 40 30 20 10 0 -10
f1 (ppm)

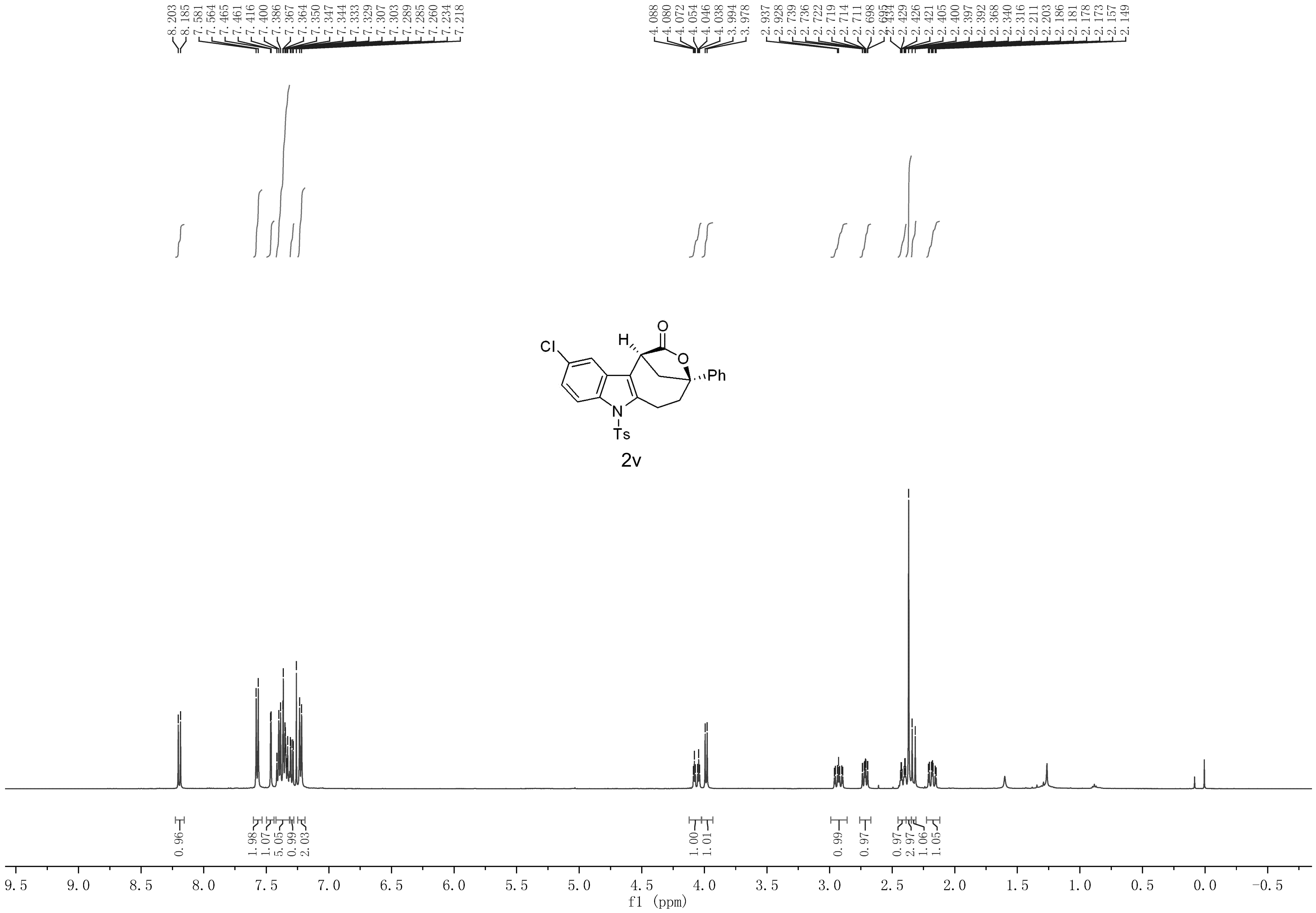
8.203 8.185 7.581 7.564 7.465 7.461 7.416 7.400 7.386 7.367 7.364 7.350 7.347 7.344 7.333 7.329 7.307 7.303 7.289 7.285 7.260 7.234 7.218
4.088 4.080 4.072 4.054 4.046 4.038 3.994 3.978
2.937 2.928 2.739 2.736 2.722 2.719 2.714 2.711 2.698 2.695 2.434 2.429 2.426 2.421 2.405 2.400 2.397 2.392 2.368 2.340 2.316 2.211 2.203 2.186 2.181 2.178 2.173 2.157 2.149
Cl
H
O
O
Ph
N
Ts
2v
0.96 1.98 1.07 5.05 0.99 2.03 1.00 1.01 0.99 0.97 0.97 2.97 1.06 1.05
9.5 9.0 8.5 8.0 7.5 7.0 6.5 6.0 5.5 5.0 4.5 4.0 3.5 3.0 2.5 2.0 1.5 1.0 0.5 0.0 -0.5
f1 (ppm)

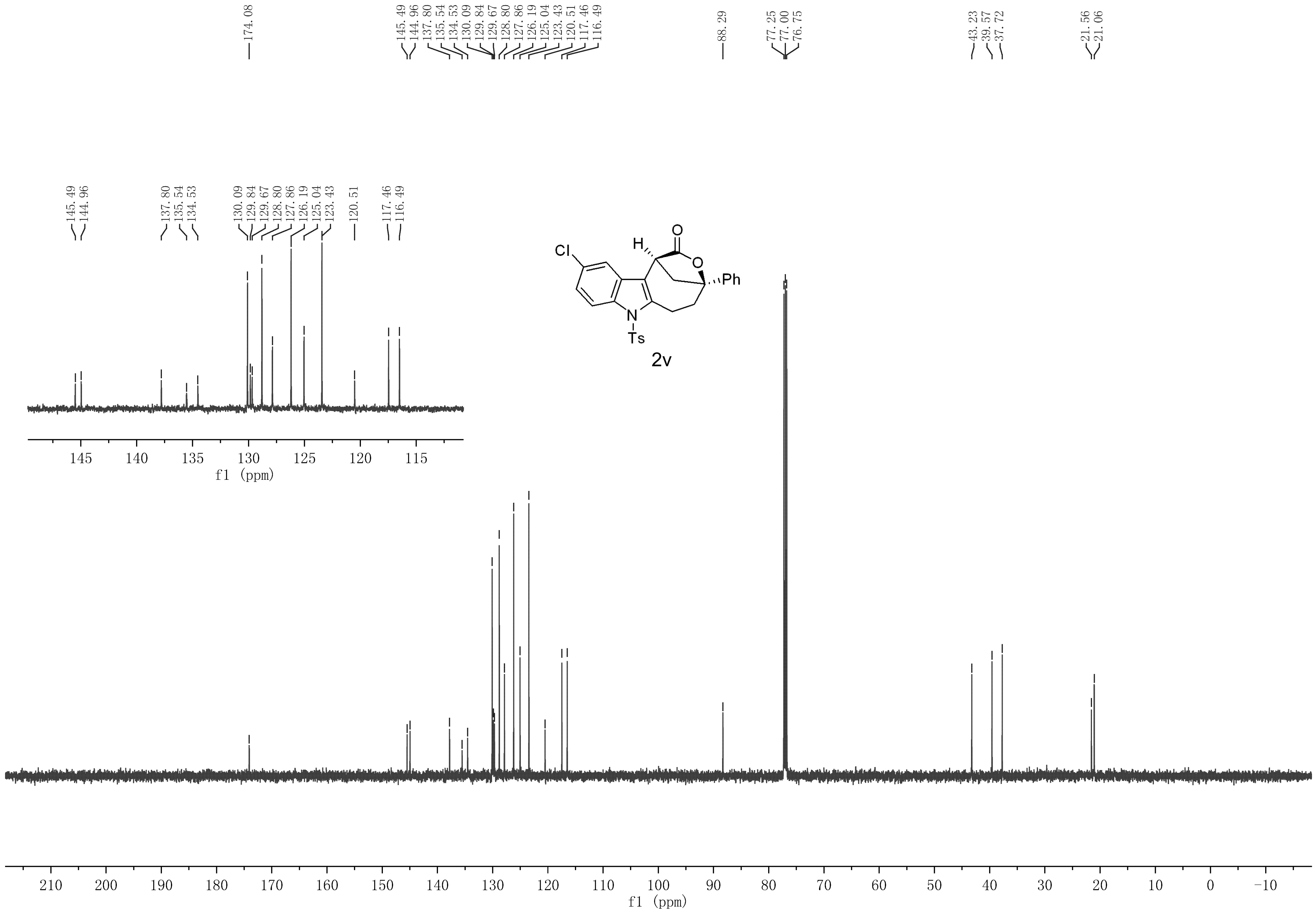
174.08
145.49
144.96
137.80
135.54
134.53
130.09
129.84
129.67
128.80
127.86
126.19
125.04
123.43
120.51
117.46
116.49
88.29
77.25
77.00
76.75
43.23
39.57
37.72
21.56
21.06
Cl
H
O
O
Ph
N
Ts
2v
f1 (ppm)
f1 (ppm)

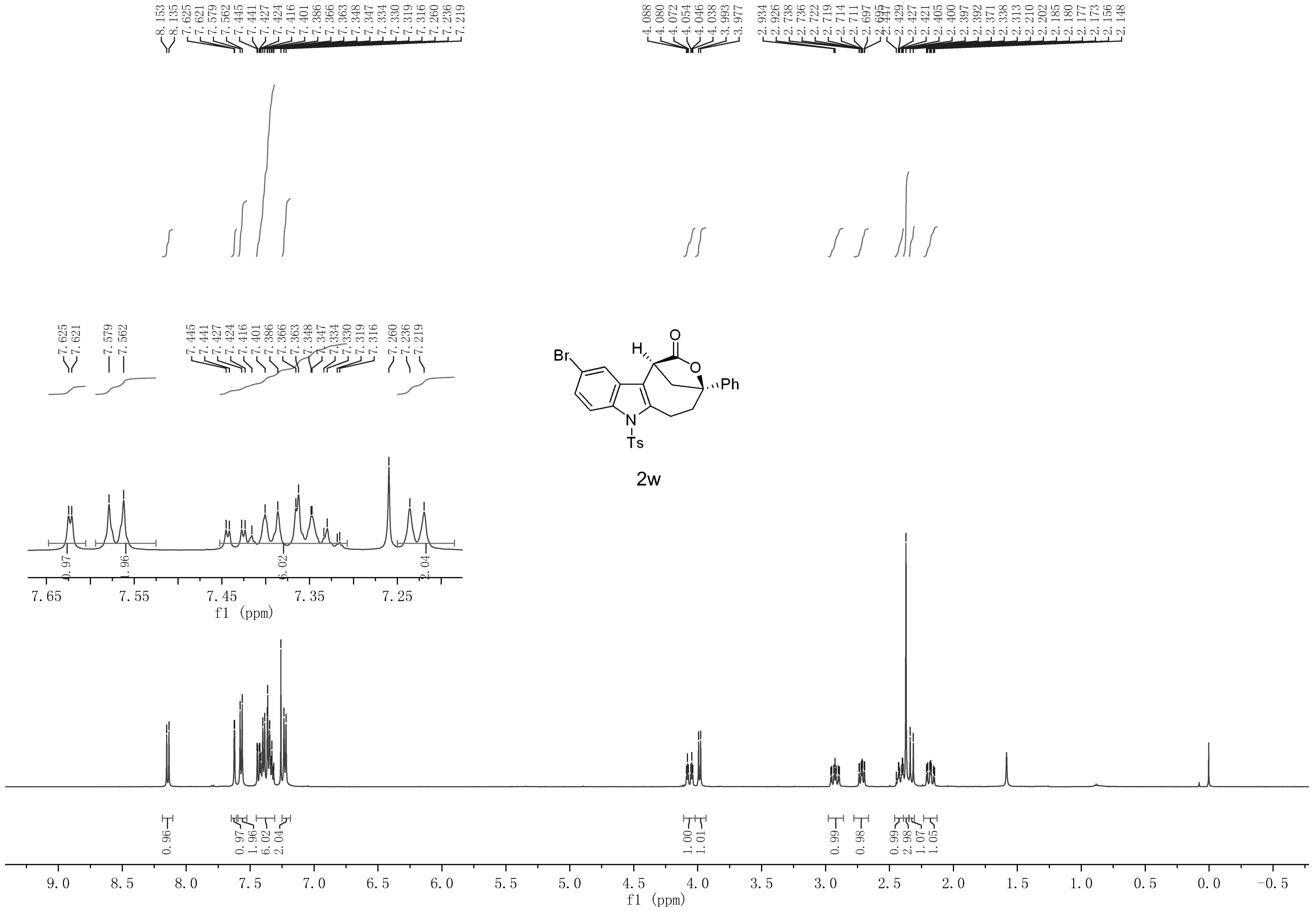

8.153 8.135 7.625 7.621 7.579 7.562 7.445 7.441 7.427 7.424 7.416 7.401 7.386 7.366 7.363 7.348 7.347 7.334 7.330 7.319 7.316 7.260 7.236 7.219
4.088 4.080 4.072 4.054 4.046 4.038 3.993 3.977
2.934 2.926 2.738 2.736 2.722 2.719 2.714 2.711 2.697 2.695 2.447 2.429 2.427 2.421 2.405 2.400 2.397 2.392 2.371 2.338 2.313 2.210 2.202 2.185 2.180 2.177 2.173 2.156 2.148
Br
H
O
O
Ph
N
Ts
2w
0.97 1.96 6.02 2.04
7.65 7.55 7.45 7.35 7.25
f1 (ppm)
0.96 0.97 1.96 6.02 2.04 1.00 1.01 0.99 0.98 0.99 2.98 1.07 1.05
9.0 8.5 8.0 7.5 7.0 6.5 6.0 5.5 5.0 4.5 4.0 3.5 3.0 2.5 2.0 1.5 1.0 0.5 0.0 −0.5
f1 (ppm)

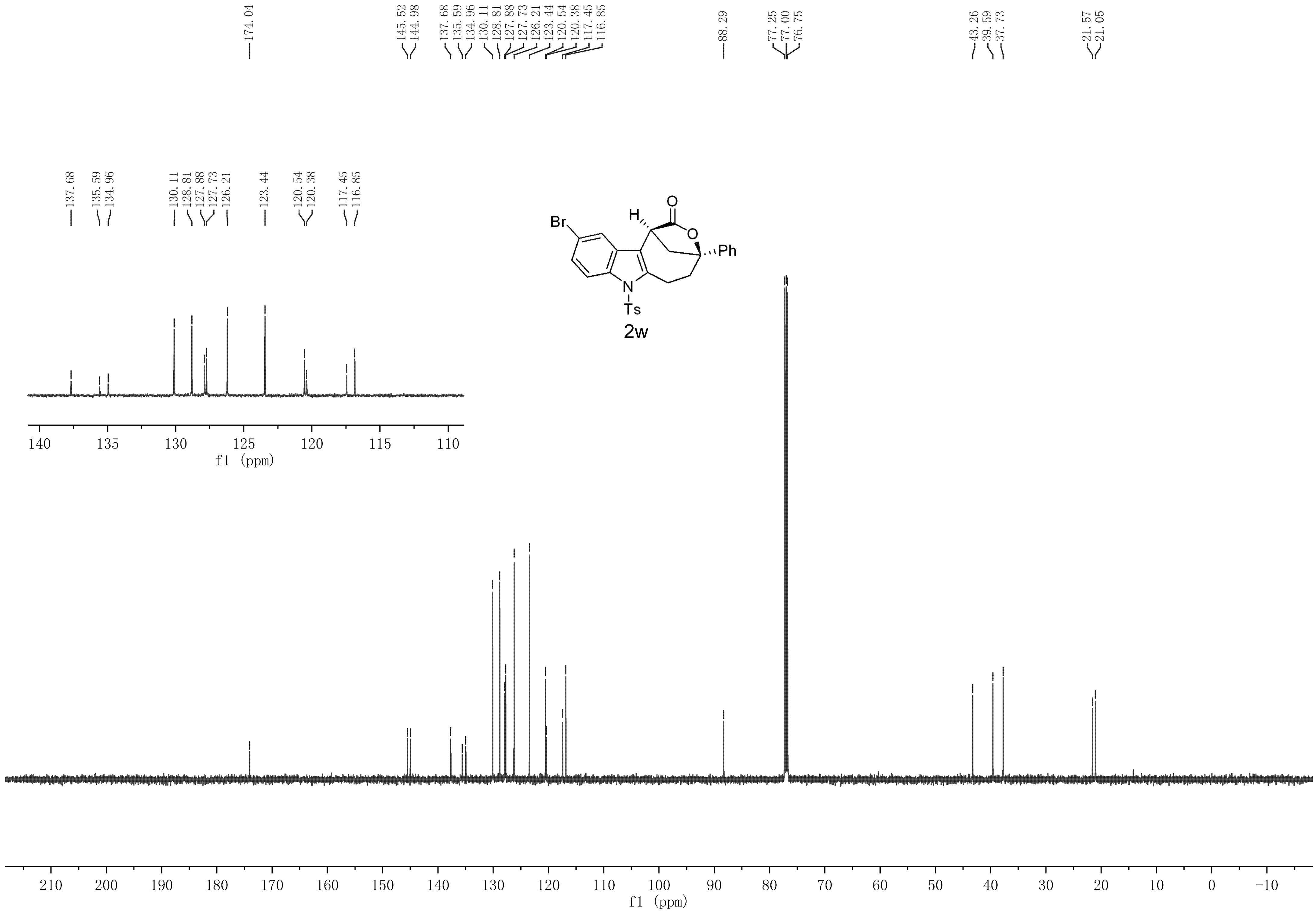
174.04
145.52
144.98
137.68
135.59
134.96
130.11
128.81
127.88
127.73
126.21
123.44
120.54
120.38
117.45
116.85
88.29
77.25
77.00
76.75
43.26
39.59
37.73
21.57
21.05
Br
H
O
O
Ph
N
Ts
2w
140 135 130 125 120 115 110
f1 (ppm)
210 200 190 180 170 160 150 140 130 120 110 100 90 80 70 60 50 40 30 20 10 0 −10
f1 (ppm)

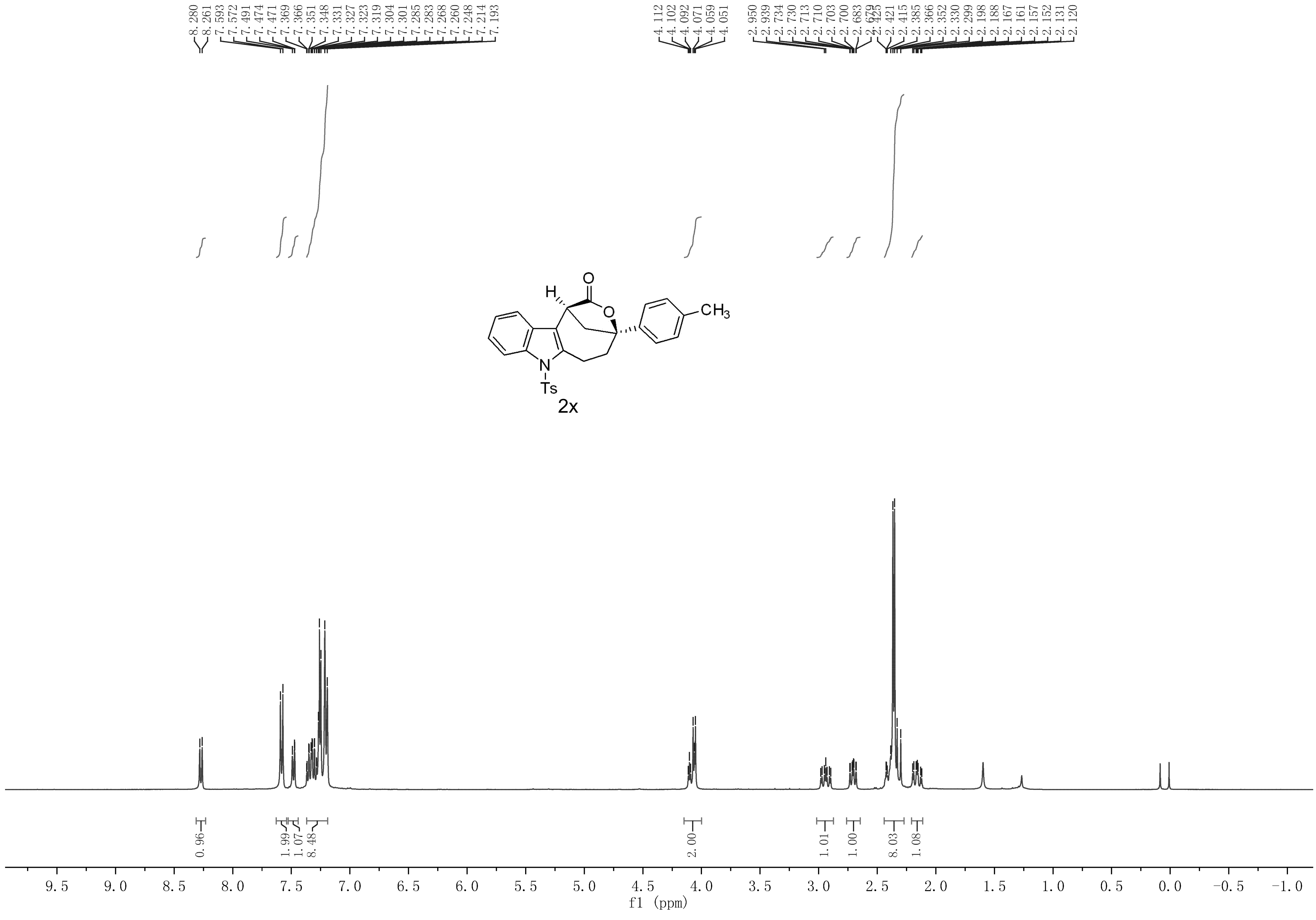
8.280
8.261
7.593
7.572
7.491
7.474
7.471
7.369
7.366
7.351
7.348
7.331
7.327
7.323
7.319
7.304
7.301
7.285
7.283
7.268
7.260
7.248
7.214
7.193
4.112
4.102
4.092
4.071
4.059
4.051
2.950
2.939
2.734
2.730
2.713
2.710
2.703
2.700
2.683
2.679
2.425
2.421
2.415
2.385
2.366
2.352
2.330
2.299
2.198
2.188
2.167
2.161
2.157
2.152
2.131
2.120
H
O
O
CH3
N
Ts
2x
0.96
1.99
1.07
8.48
2.00
1.01
1.00
8.03
1.08
9.5
9.0
8.5
8.0
7.5
7.0
6.5
6.0
5.5
5.0
4.5
4.0
3.5
3.0
2.5
2.0
1.5
1.0
0.5
0.0
-0.5
-1.0
f1 (ppm)

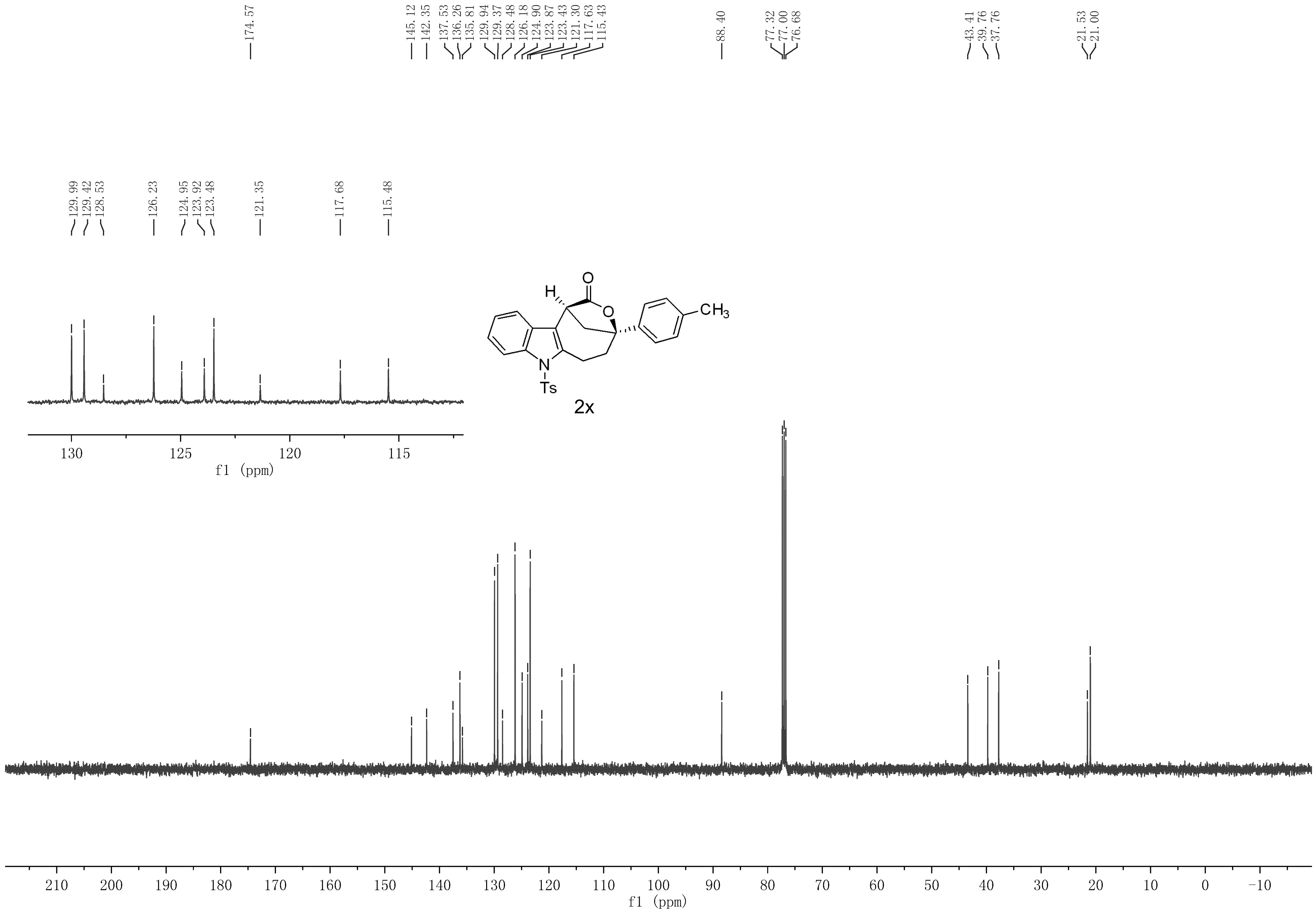
174.57
145.12
142.35
137.53
136.26
135.81
129.94
129.37
128.48
126.18
124.90
123.87
123.43
121.30
117.63
115.43
88.40
77.32
77.00
76.68
43.41
39.76
37.76
21.53
21.00
129.99
129.42
128.53
126.23
124.95
123.92
123.48
121.35
117.68
115.48
130
125
120
115
f1 (ppm)
H
O
O
CH3
N
Ts
2x
210
200
190
180
170
160
150
140
130
120
110
100
90
80
70
60
50
40
30
20
10
0
-10
f1 (ppm)

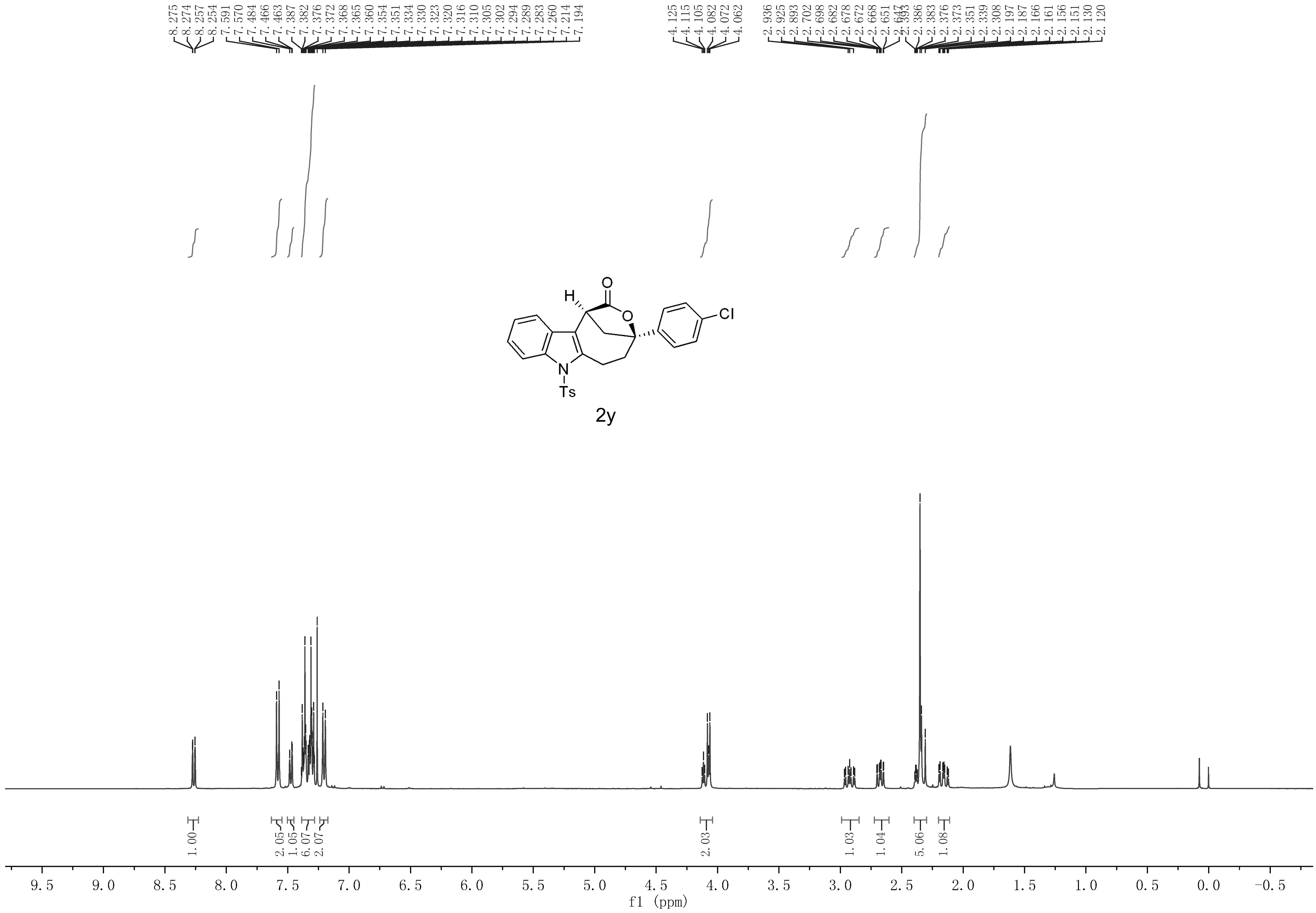

8.275
8.274
8.257
8.254
7.591
7.570
7.484
7.466
7.463
7.387
7.382
7.376
7.372
7.368
7.365
7.360
7.354
7.351
7.334
7.330
7.323
7.320
7.316
7.310
7.305
7.302
7.294
7.289
7.283
7.260
7.214
7.194
4.125
4.115
4.105
4.082
4.072
4.062
2.936
2.925
2.893
2.702
2.698
2.682
2.678
2.672
2.668
2.651
2.386
2.383
2.376
2.373
2.351
2.339
2.308
2.197
2.187
2.166
2.161
2.156
2.151
2.130
2.120
H
O
O
Cl
N
Ts
2y
1.00
2.05
1.05
6.07
2.07
2.03
1.03
1.04
5.06
1.08
9.5
9.0
8.5
8.0
7.5
7.0
6.5
6.0
5.5
5.0
4.5
4.0
3.5
3.0
2.5
2.0
1.5
1.0
0.5
0.0
-0.5
f1 (ppm)

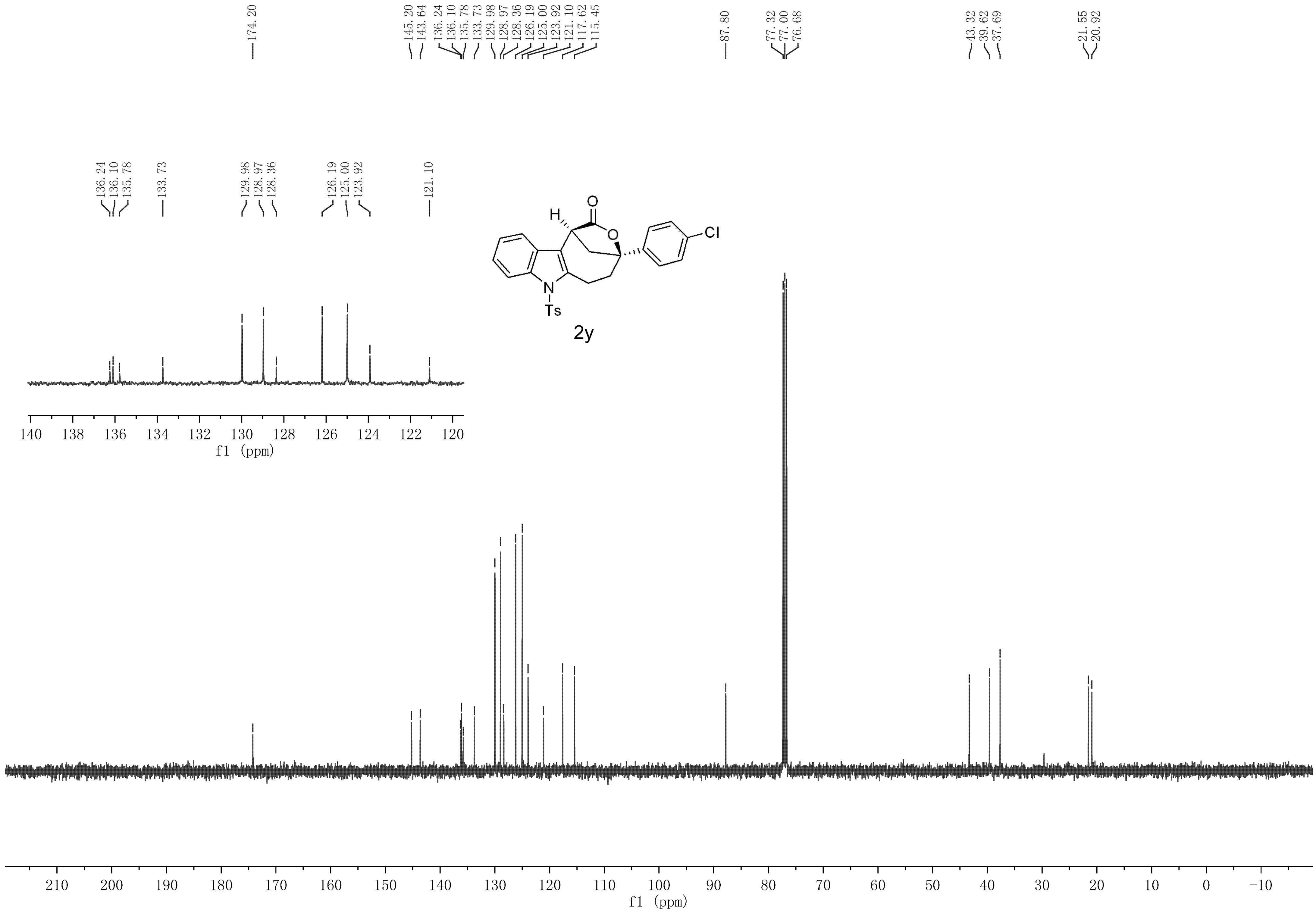
174.20
145.20
143.64
136.24
136.10
135.78
133.73
129.98
128.97
128.36
126.19
125.00
123.92
121.10
117.62
115.45
87.80
77.32
77.00
76.68
43.32
39.62
37.69
21.55
20.92
H
O
O
Cl
N
Ts
2y
140 138 136 134 132 130 128 126 124 122 120
f1 (ppm)
210 200 190 180 170 160 150 140 130 120 110 100 90 80 70 60 50 40 30 20 10 0 −10
f1 (ppm)

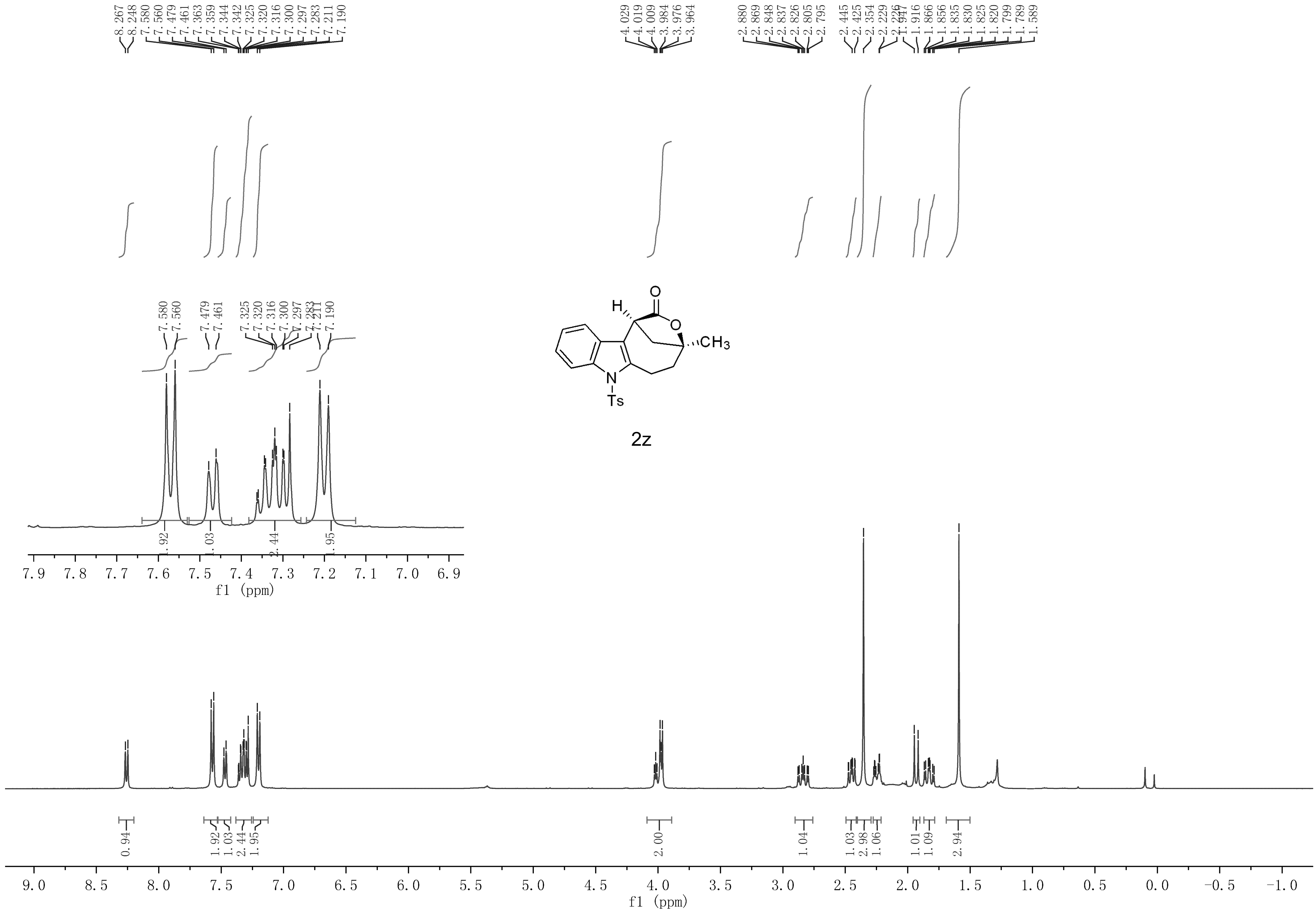
8.267
8.248
7.580
7.560
7.479
7.461
7.363
7.359
7.344
7.342
7.325
7.320
7.316
7.300
7.297
7.283
7.211
7.190
4.029
4.019
4.009
3.984
3.976
3.964
2.880
2.869
2.848
2.837
2.826
2.805
2.795
2.445
2.425
2.354
2.229
1.916
1.866
1.856
1.835
1.830
1.825
1.820
1.799
1.789
1.589
H
O
O
CH3
N
Ts
2z
0.94
1.92
1.03
2.44
1.95
2.00
1.04
1.03
2.98
1.06
1.01
1.09
2.94
f1 (ppm)

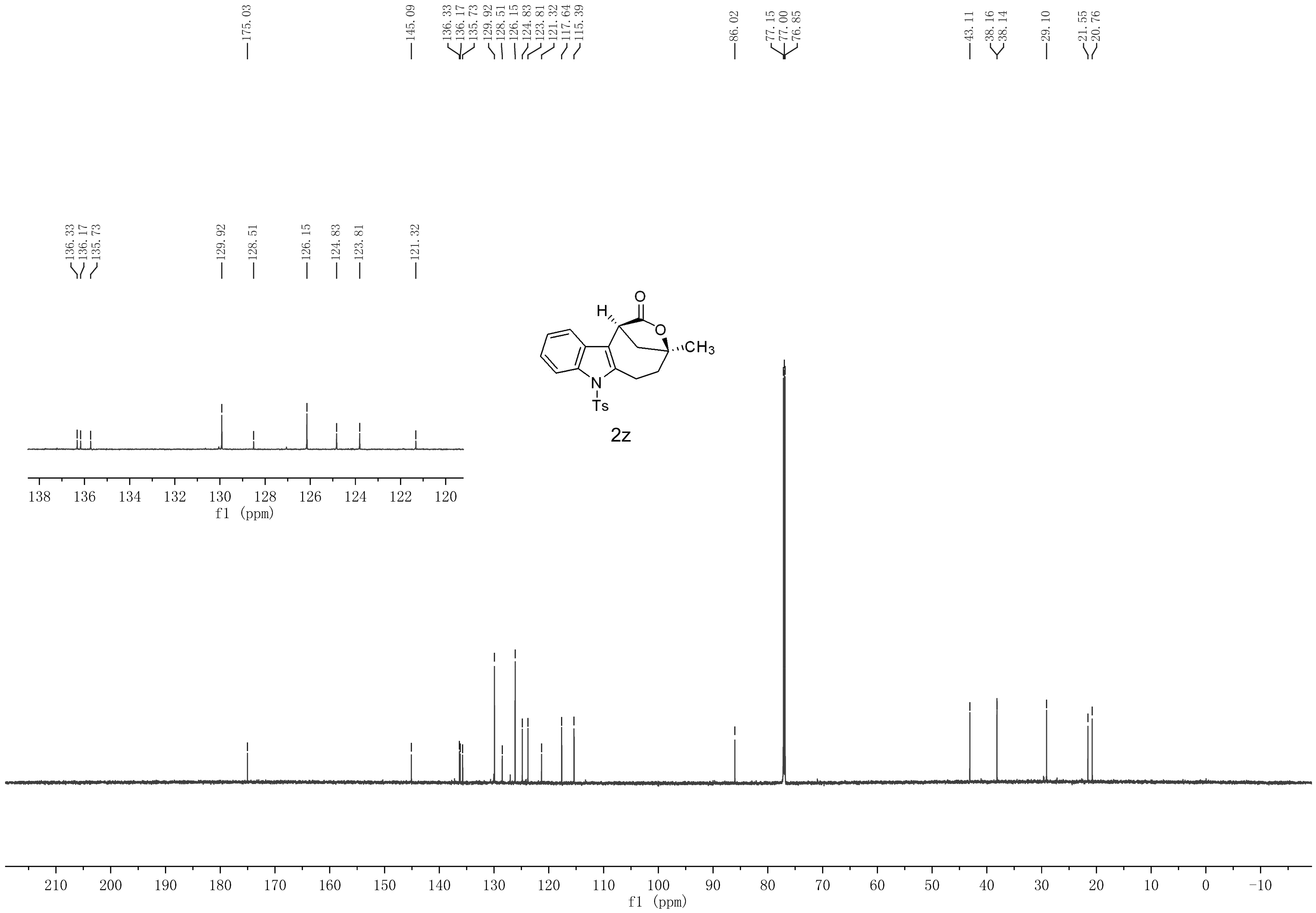
175.03
145.09
136.33
136.17
135.73
129.92
128.51
126.15
124.83
123.81
121.32
117.64
115.39
86.02
77.15
77.00
76.85
43.11
38.16
38.14
29.10
21.55
20.76
H
O
O
CH3
N
Ts
2z
f1 (ppm)

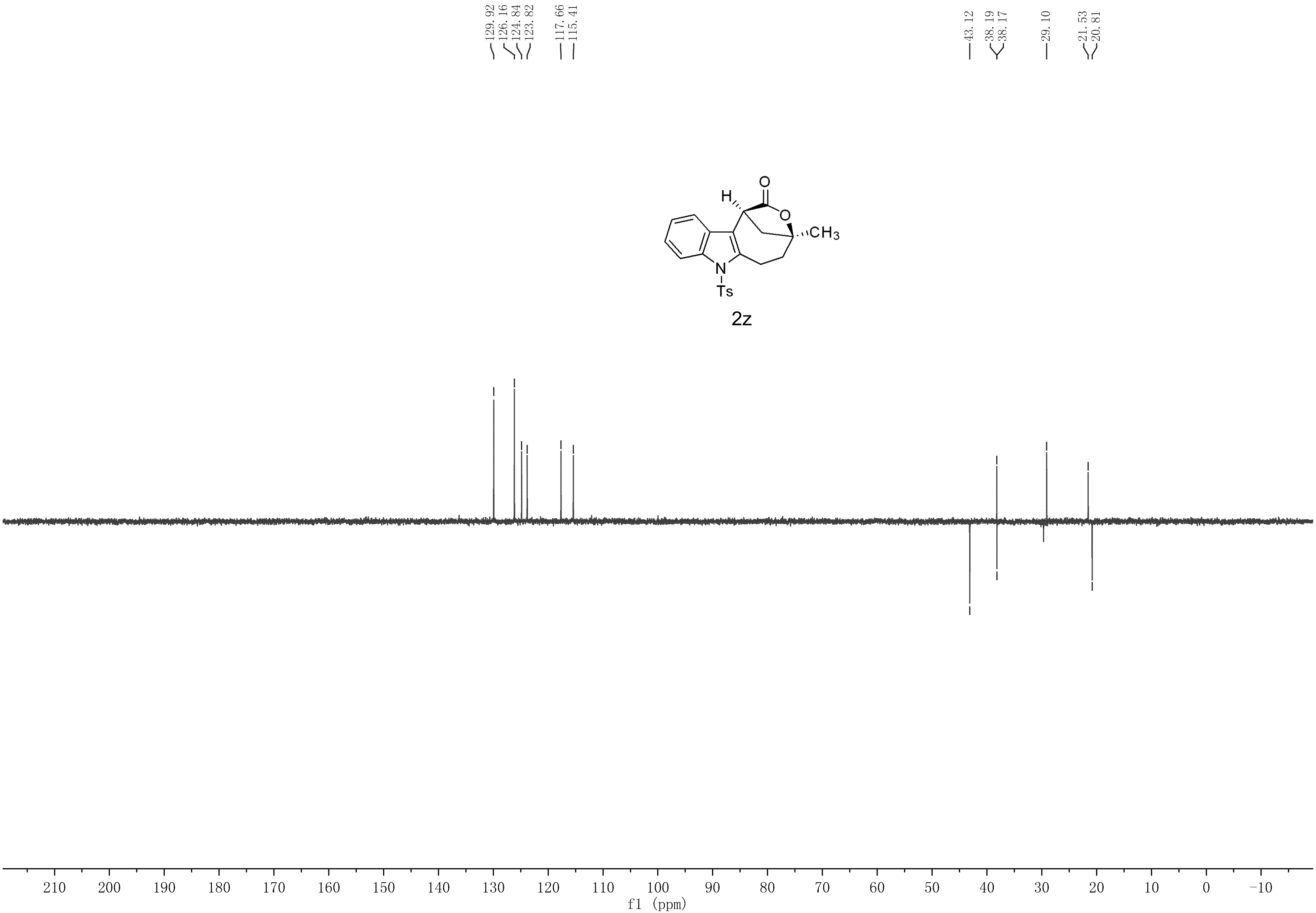

129.92
126.16
124.84
123.82
117.66
115.41
43.12
38.19
38.17
29.10
21.53
20.81
O
H
O
CH3
N
Ts
2z
210 200 190 180 170 160 150 140 130 120 110 100 90 80 70 60 50 40 30 20 10 0 −10
f1 (ppm)

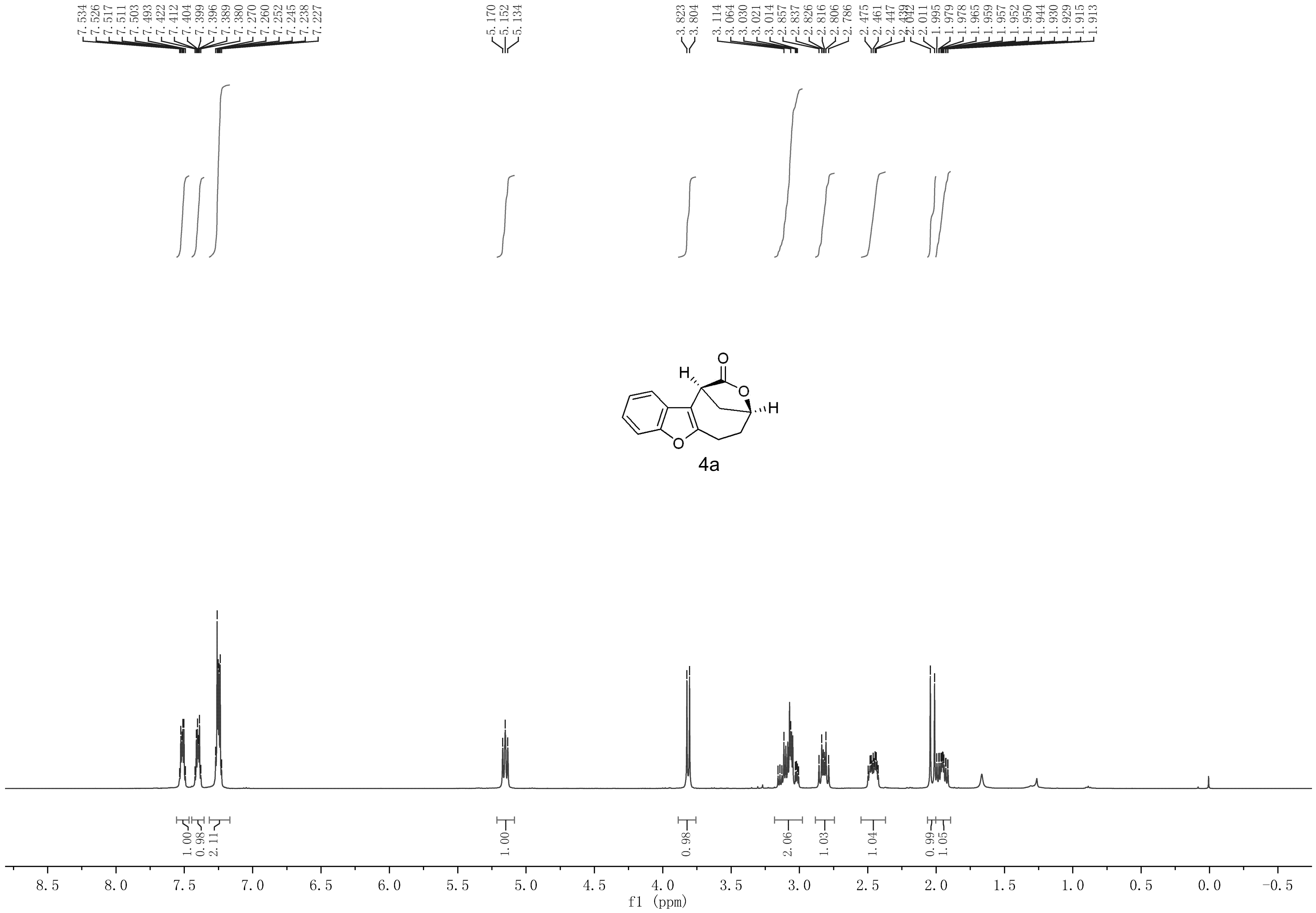

7.534
7.526
7.517
7.511
7.503
7.493
7.422
7.412
7.404
7.399
7.396
7.389
7.380
7.270
7.260
7.252
7.245
7.238
7.227
5.170
5.152
5.134
3.823
3.804
3.114
3.064
3.030
3.021
3.014
2.857
2.837
2.826
2.816
2.806
2.786
2.475
2.461
2.447
2.011
1.995
1.979
1.978
1.965
1.959
1.957
1.952
1.950
1.944
1.930
1.929
1.915
1.913
H
O
O
H
O
4a
1.00
0.98
2.11
1.00
0.98
2.06
1.03
1.04
0.99
1.05
8.5
8.0
7.5
7.0
6.5
6.0
5.5
5.0
4.5
4.0
3.5
3.0
2.5
2.0
1.5
1.0
0.5
0.0
-0.5
f1 (ppm)

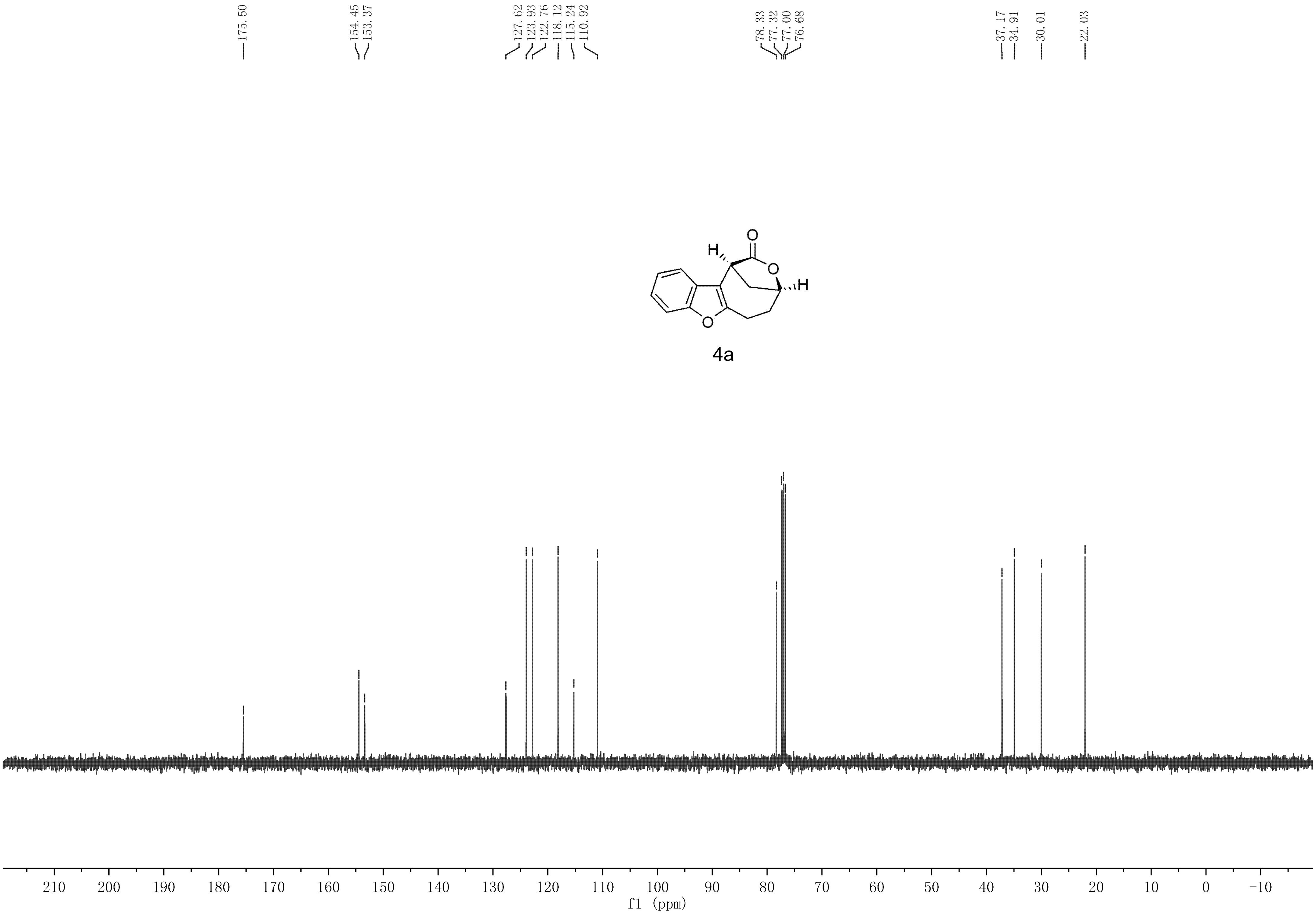
175.50
154.45
153.37
127.62
123.93
122.76
118.12
115.24
110.92
78.33
77.32
77.00
76.68
37.17
34.91
30.01
22.03
H
O
O
H
O
4a
210 200 190 180 170 160 150 140 130 120 110 100 90 80 70 60 50 40 30 20 10 0 −10
f1 (ppm)

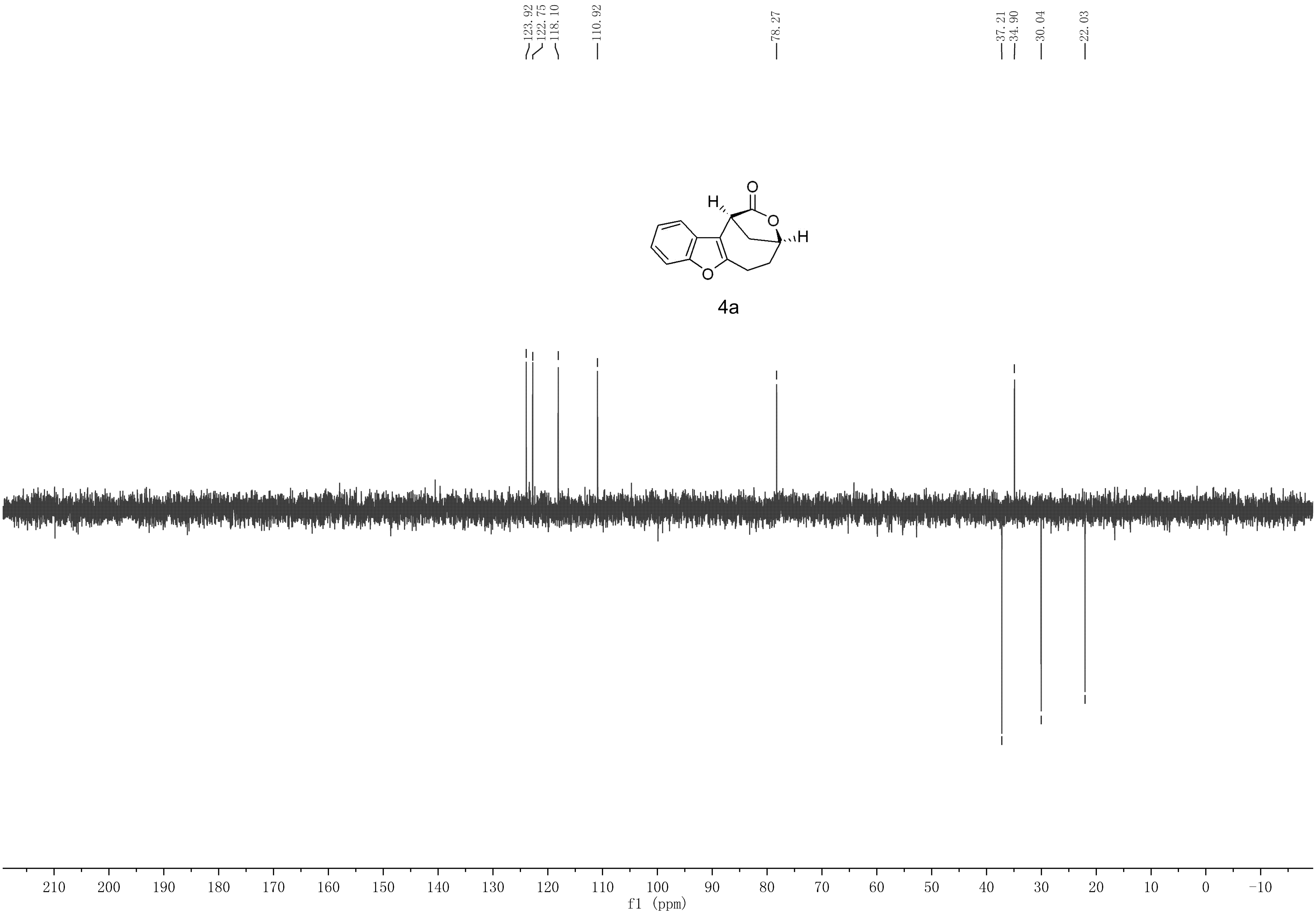
123.92
122.75
118.10
110.92
78.27
37.21
34.90
30.04
22.03
O
O
H
H
O
4a
210
200
190
180
170
160
150
140
130
120
110
100
90
80
70
60
50
40
30
20
10
0
−10
f1 (ppm)

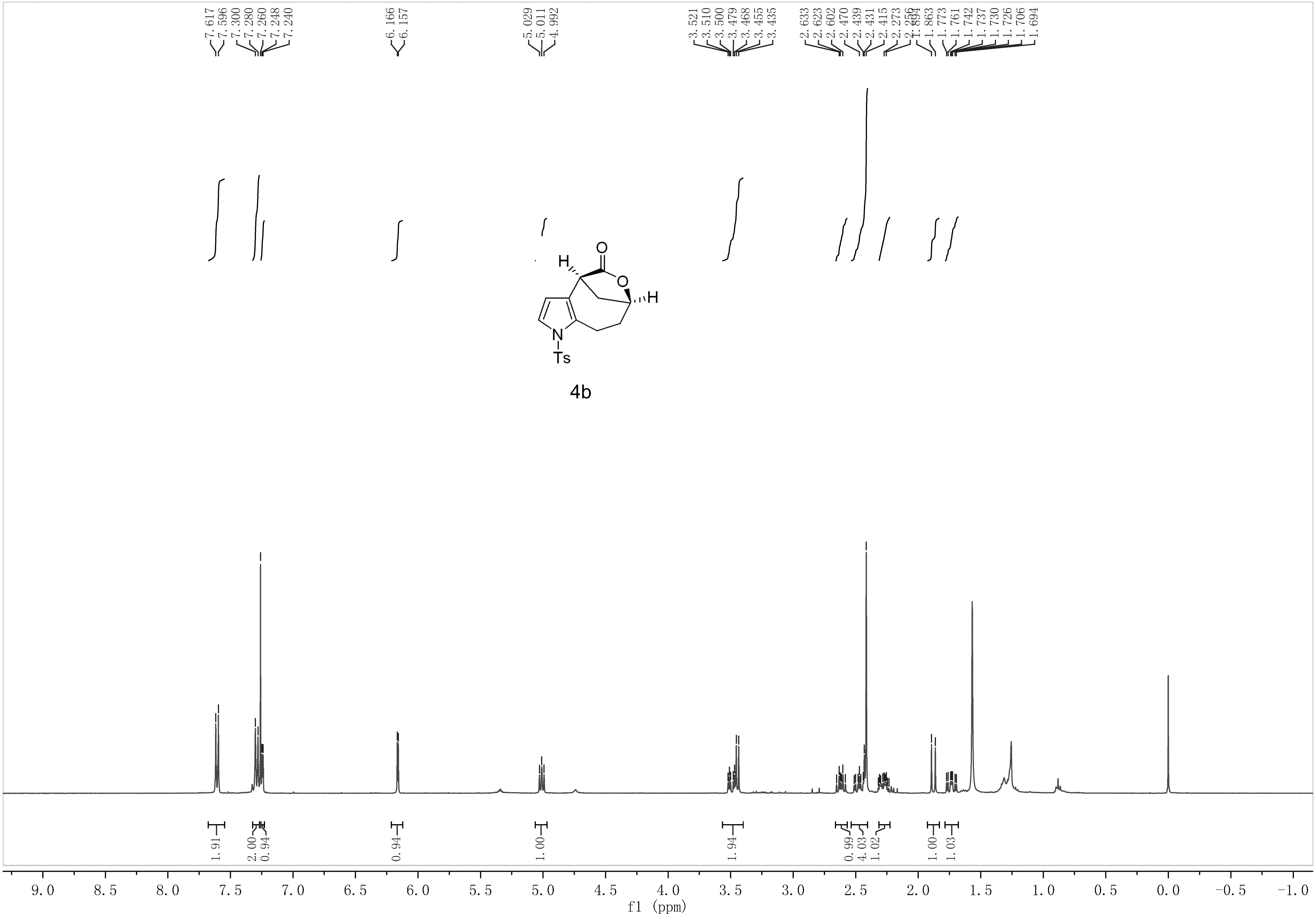
7.617
7.596
7.300
7.280
7.260
7.248
7.240
6.166
6.157
5.029
5.011
4.992
3.521
3.510
3.500
3.479
3.468
3.455
3.435
2.633
2.623
2.602
2.470
2.439
2.431
2.415
2.273
2.256
1.894
1.863
1.773
1.761
1.742
1.737
1.730
1.726
1.706
1.694
O
O
H
H
N
Ts
4b
1.91
2.00
0.94
0.94
1.00
1.94
0.99
4.03
1.02
1.00
1.03
9.0 8.5 8.0 7.5 7.0 6.5 6.0 5.5 5.0 4.5 4.0 3.5 3.0 2.5 2.0 1.5 1.0 0.5 0.0 -0.5 -1.0
f1 (ppm)

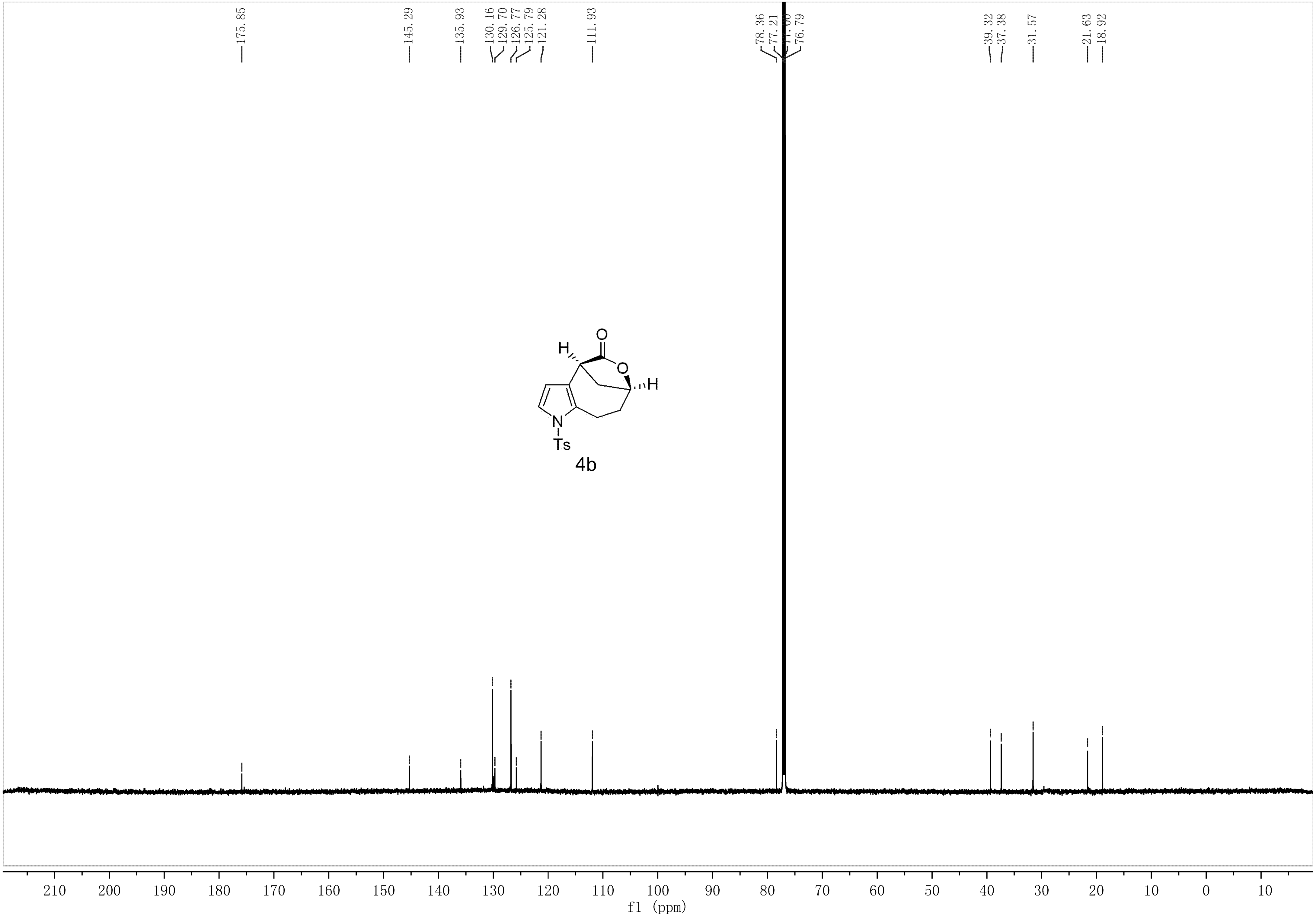
175.85
145.29
135.93
130.16
129.70
126.77
125.79
121.28
111.93
78.36
77.21
76.79
39.32
37.38
31.57
21.63
18.92
O
H
H
N
Ts
4b
210 200 190 180 170 160 150 140 130 120 110 100 90 80 70 60 50 40 30 20 10 0 −10
f1 (ppm)

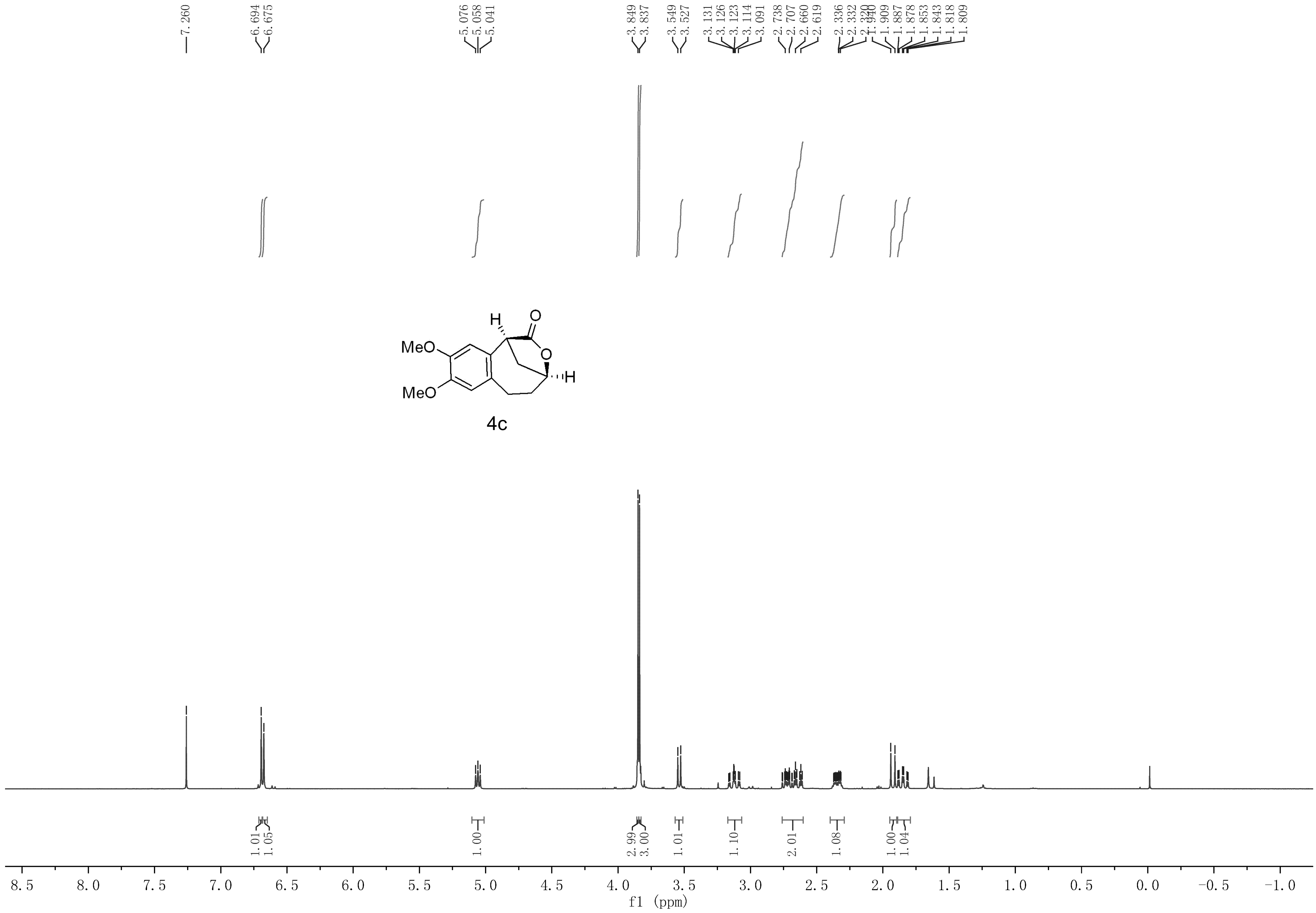

7.260
6.694
6.675
5.076
5.058
5.041
3.849
3.837
3.549
3.527
3.131
3.126
3.123
3.114
3.091
2.738
2.707
2.660
2.619
2.336
2.332
1.909
1.887
1.878
1.853
1.843
1.818
1.809
H
O
MeO
O
H
MeO
4c
1.01
1.05
1.00
2.99
3.00
1.01
1.10
2.01
1.08
1.00
1.04
8.5 8.0 7.5 7.0 6.5 6.0 5.5 5.0 4.5 4.0 3.5 3.0 2.5 2.0 1.5 1.0 0.5 0.0 −0.5 −1.0
f1 (ppm)

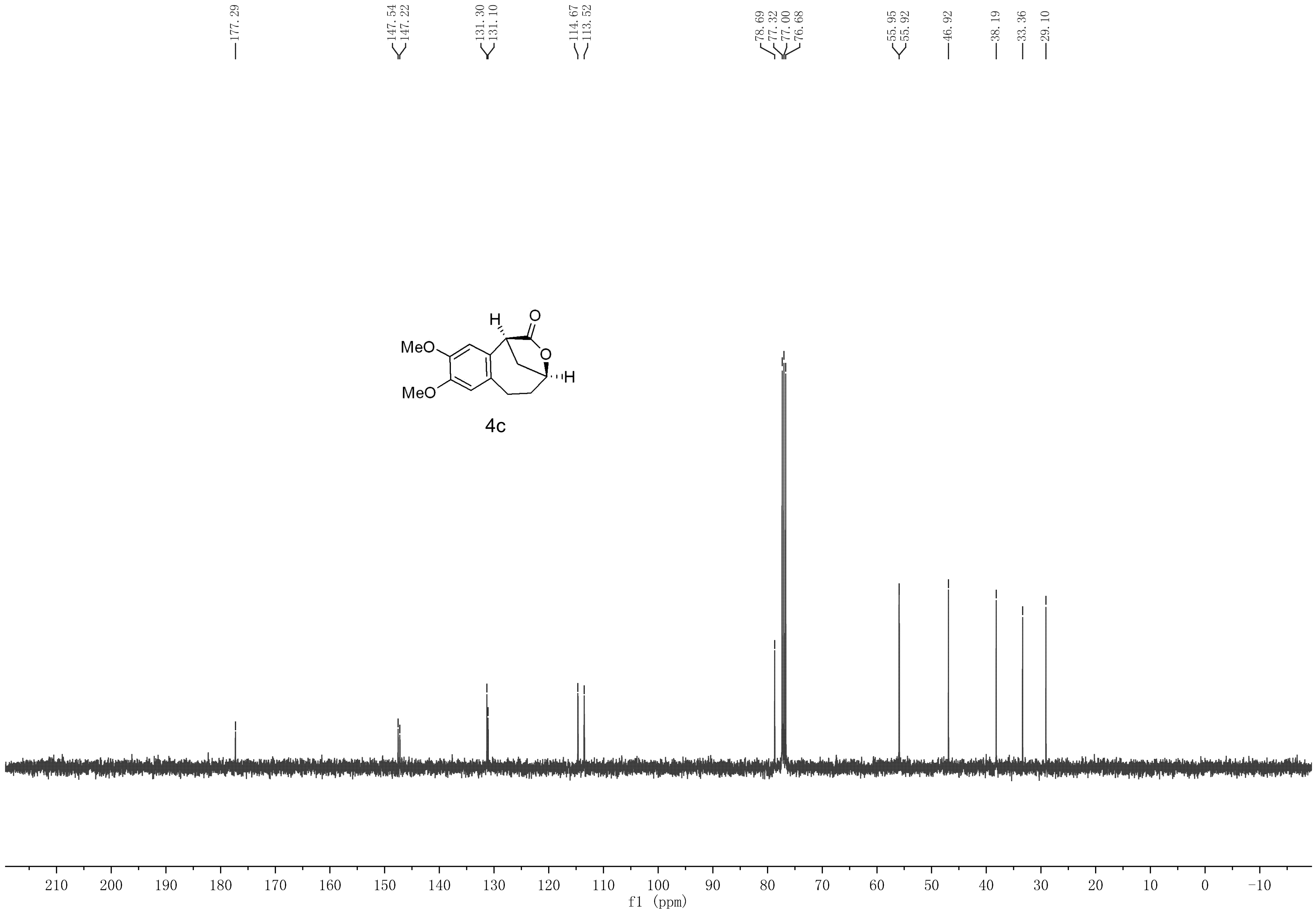
177.29
147.54
147.22
131.30
131.10
114.67
113.52
78.69
77.32
77.00
76.68
55.95
55.92
46.92
38.19
33.36
29.10
MeO
MeO
H
O
O
H
4c
210 200 190 180 170 160 150 140 130 120 110 100 90 80 70 60 50 40 30 20 10 0 −10
f1 (ppm)

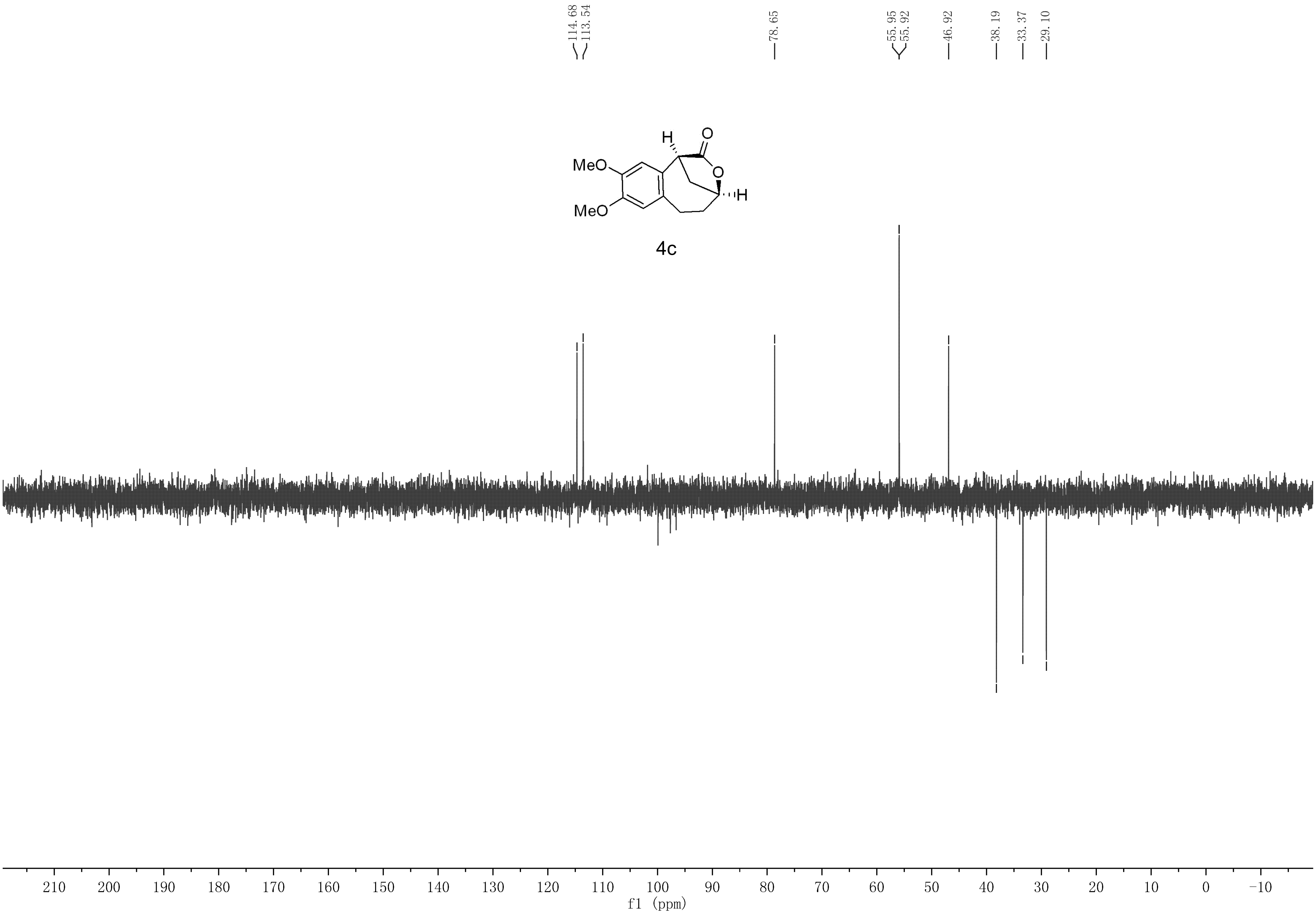
114.68
113.54
78.65
55.95
55.92
46.92
38.19
33.37
29.10
MeO
MeO
H
H
O
O
4c
210 200 190 180 170 160 150 140 130 120 110 100 90 80 70 60 50 40 30 20 10 0 −10
f1 (ppm)

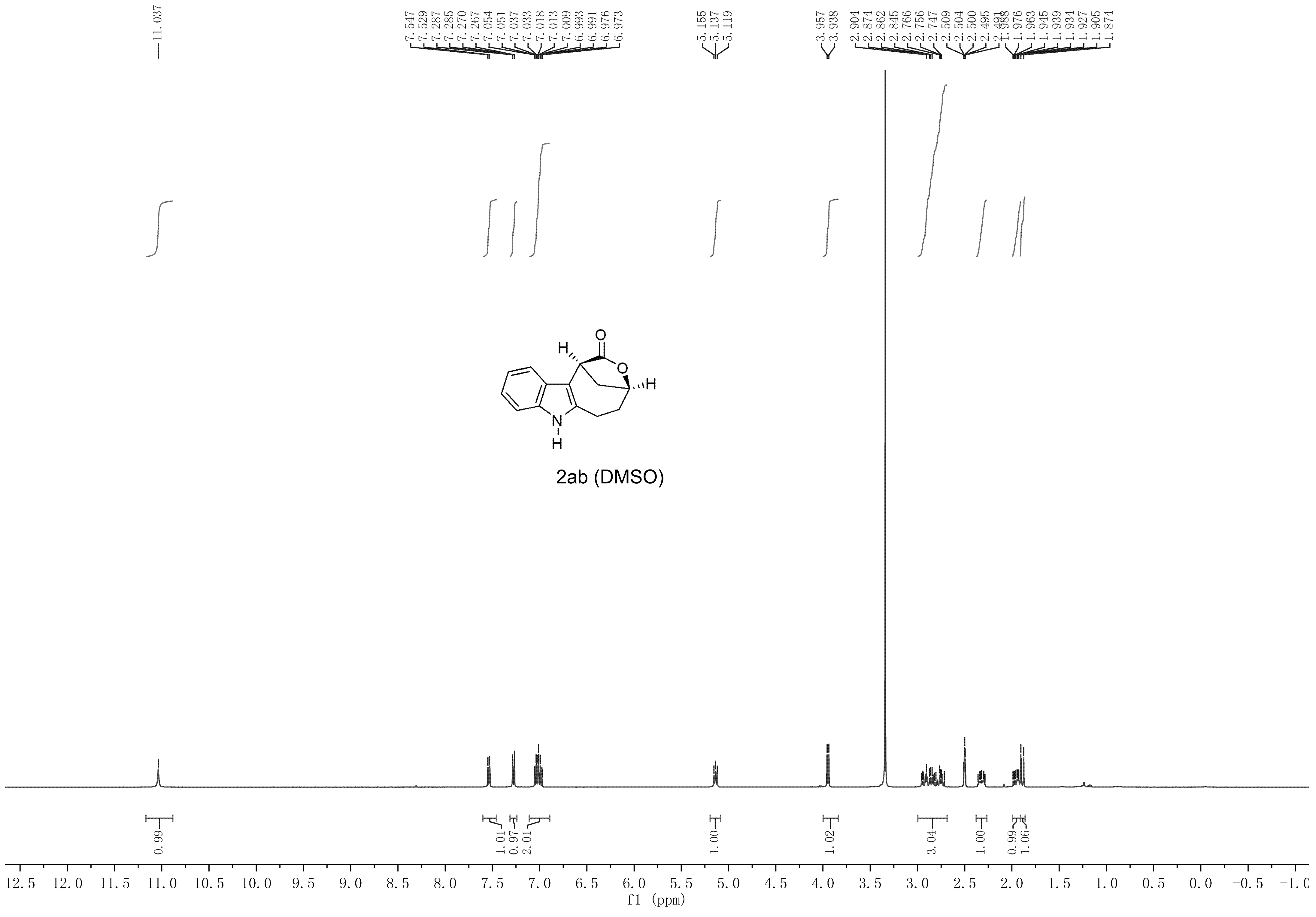
11.037
7.547
7.529
7.287
7.285
7.270
7.267
7.054
7.051
7.037
7.033
7.018
7.013
7.009
6.993
6.991
6.976
6.973
5.155
5.137
5.119
3.957
3.938
2.904
2.874
2.862
2.845
2.766
2.756
2.747
2.509
2.504
2.500
2.495
2.491
1.988
1.976
1.963
1.945
1.939
1.934
1.927
1.905
1.874
2ab (DMSO)
0.99
1.01
0.97
2.01
1.00
1.02
3.04
1.00
0.99
1.06
12.5 12.0 11.5 11.0 10.5 10.0 9.5 9.0 8.5 8.0 7.5 7.0 6.5 6.0 5.5 5.0 4.5 4.0 3.5 3.0 2.5 2.0 1.5 1.0 0.5 0.0 -0.5 -1.0
f1 (ppm)

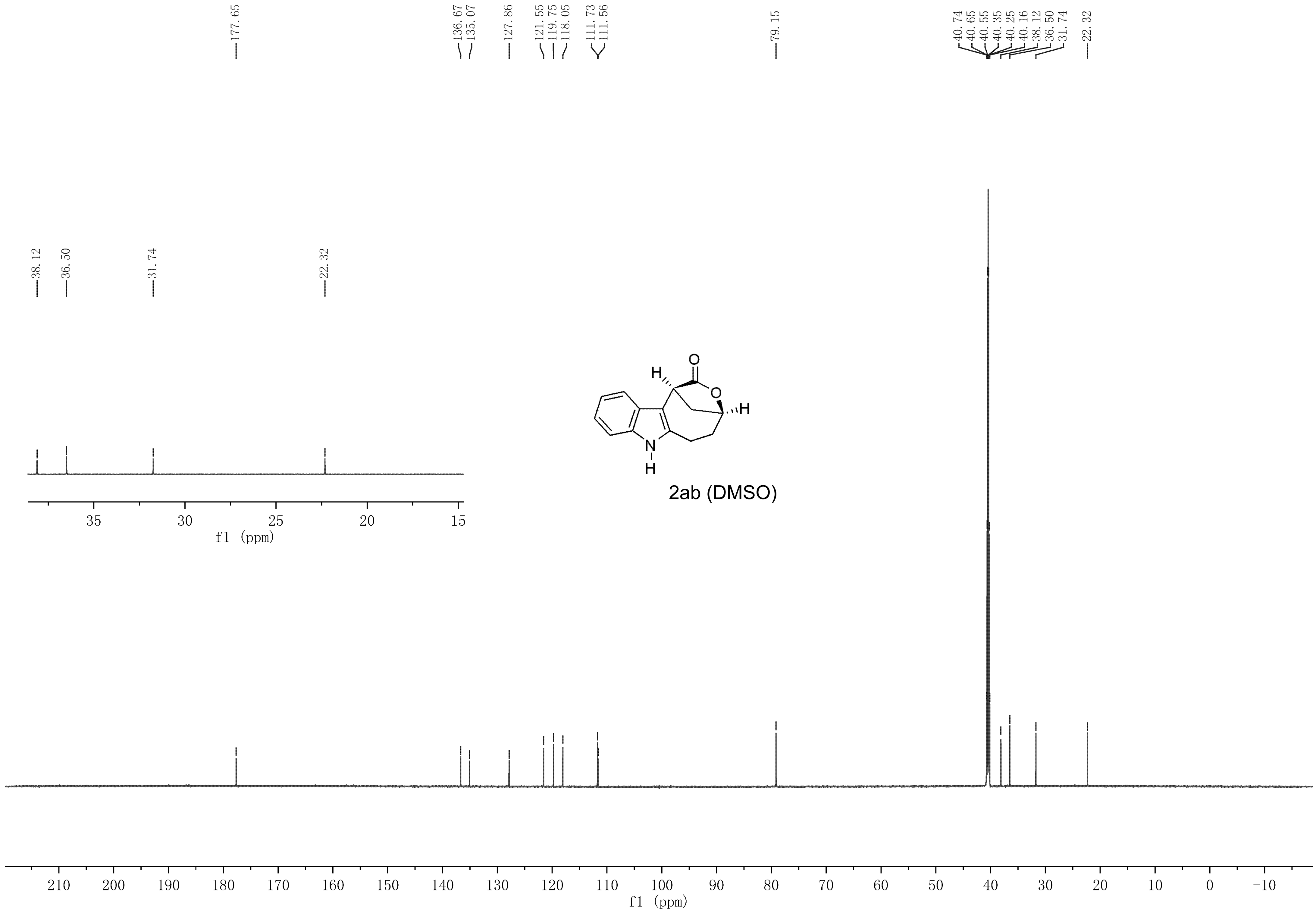
177.65
136.67
135.07
127.86
121.55
119.75
118.05
111.73
111.56
79.15
40.74
40.65
40.55
40.35
40.25
40.16
38.12
36.50
31.74
22.32
38.12
36.50
31.74
22.32
35
30
25
20
15
f1 (ppm)
H
O
O
H
N
H
2ab (DMSO)
210
200
190
180
170
160
150
140
130
120
110
100
90
80
70
60
50
40
30
20
10
0
-10
f1 (ppm)

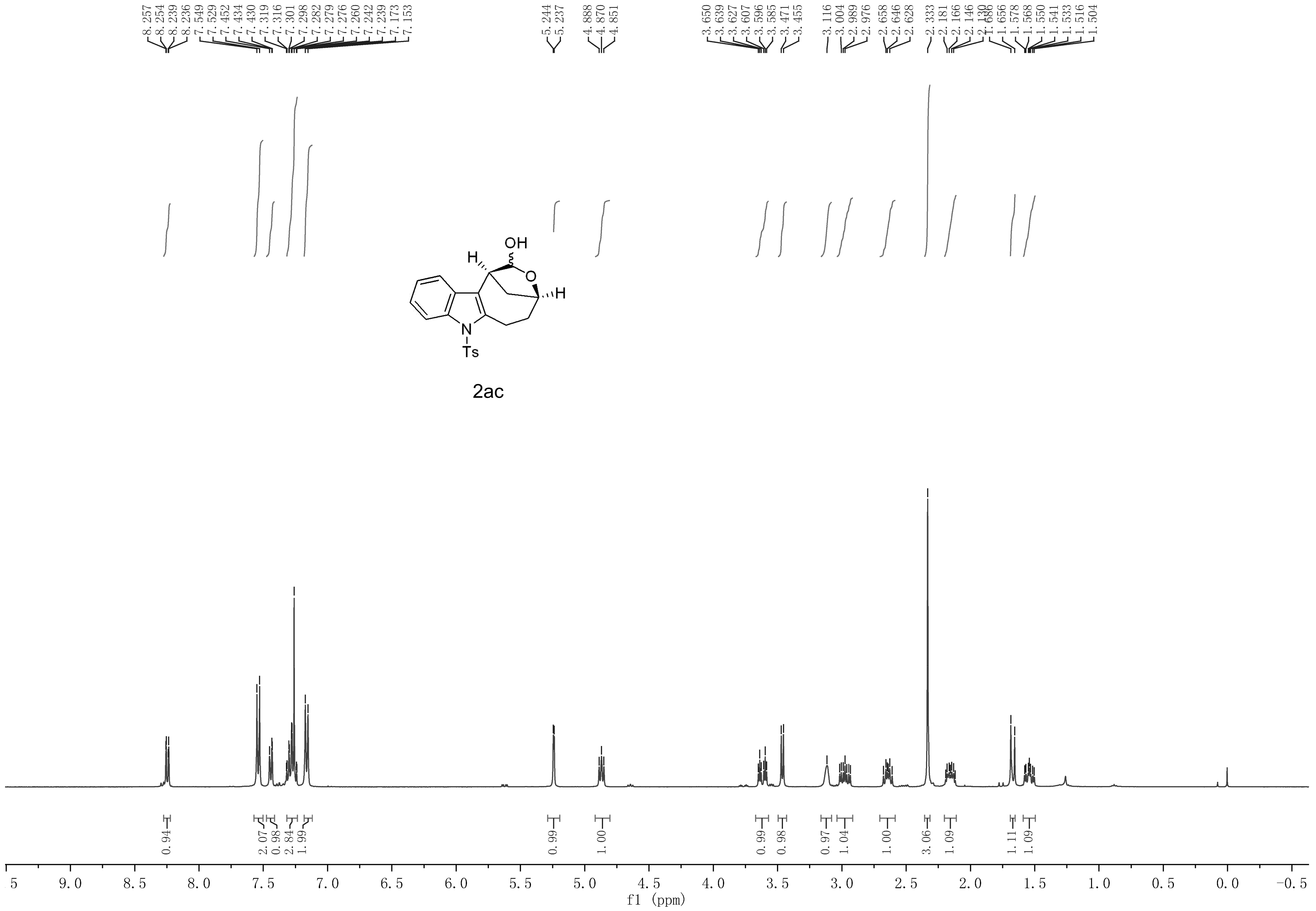
8.257
8.254
8.239
8.236
7.549
7.529
7.452
7.434
7.430
7.319
7.316
7.301
7.298
7.282
7.279
7.276
7.260
7.242
7.239
7.173
7.153
5.244
5.237
4.888
4.870
4.851
3.650
3.639
3.627
3.607
3.596
3.585
3.471
3.455
3.116
3.004
2.989
2.976
2.658
2.646
2.628
2.333
2.181
2.166
2.146
2.130
1.686
1.656
1.578
1.568
1.550
1.541
1.533
1.516
1.504
OH
H
O
H
N
Ts
2ac
0.94
2.07
0.98
2.84
1.99
0.99
1.00
0.99
0.98
0.97
1.04
1.00
3.06
1.09
1.11
1.09
9.0
8.5
8.0
7.5
7.0
6.5
6.0
5.5
5.0
4.5
4.0
3.5
3.0
2.5
2.0
1.5
1.0
0.5
0.0
-0.5
f1 (ppm)

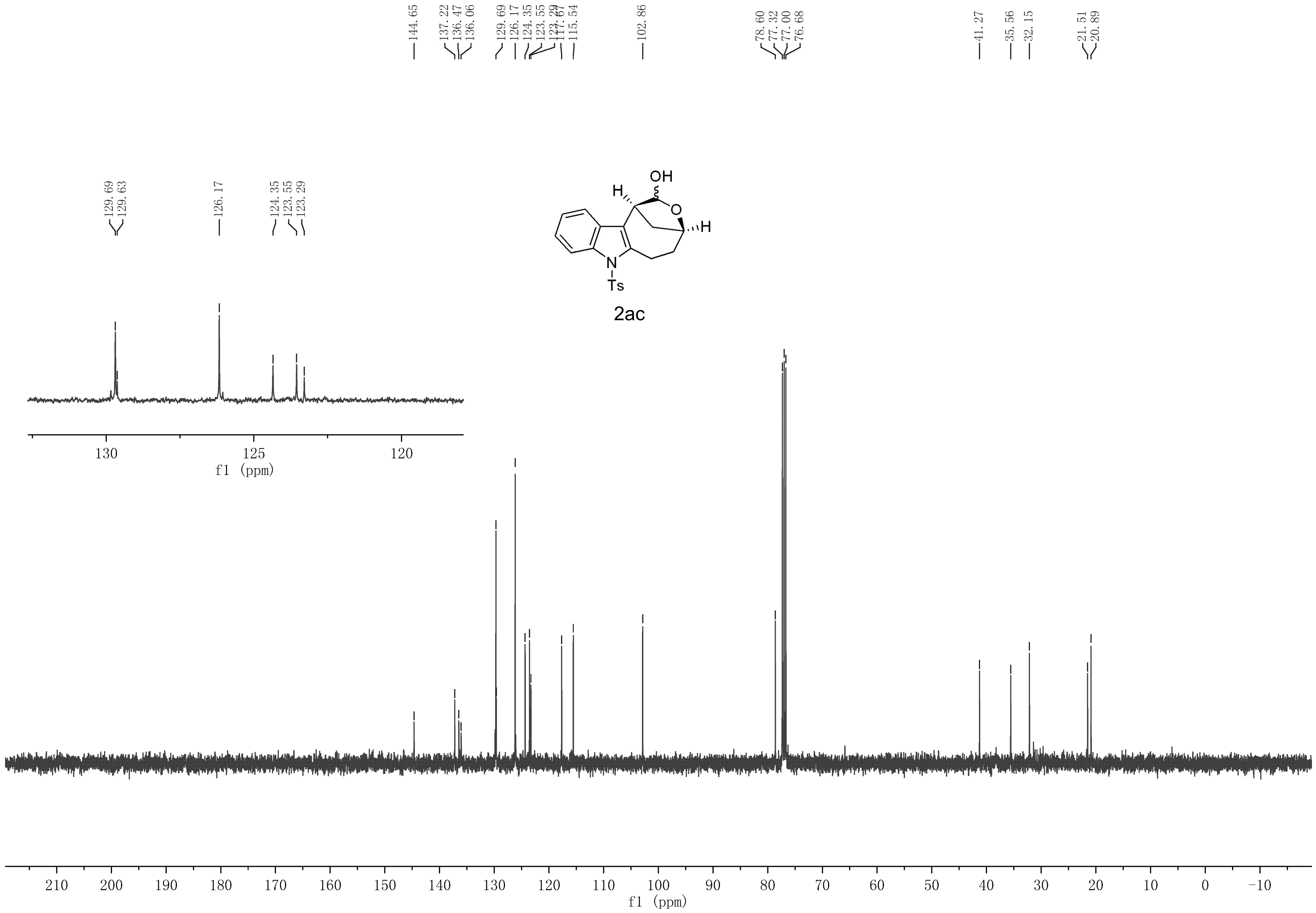

144.65
137.22
136.47
136.06
129.69
126.17
124.35
123.55
123.29
117.67
115.54
102.86
78.60
77.32
77.00
76.68
41.27
35.56
32.15
21.51
20.89
129.69
129.63
126.17
124.35
123.55
123.29
OH
H
O
H
N
Ts
2ac
130
125
120
f1 (ppm)
210
200
190
180
170
160
150
140
130
120
110
100
90
80
70
60
50
40
30
20
10
0
−10
f1 (ppm)

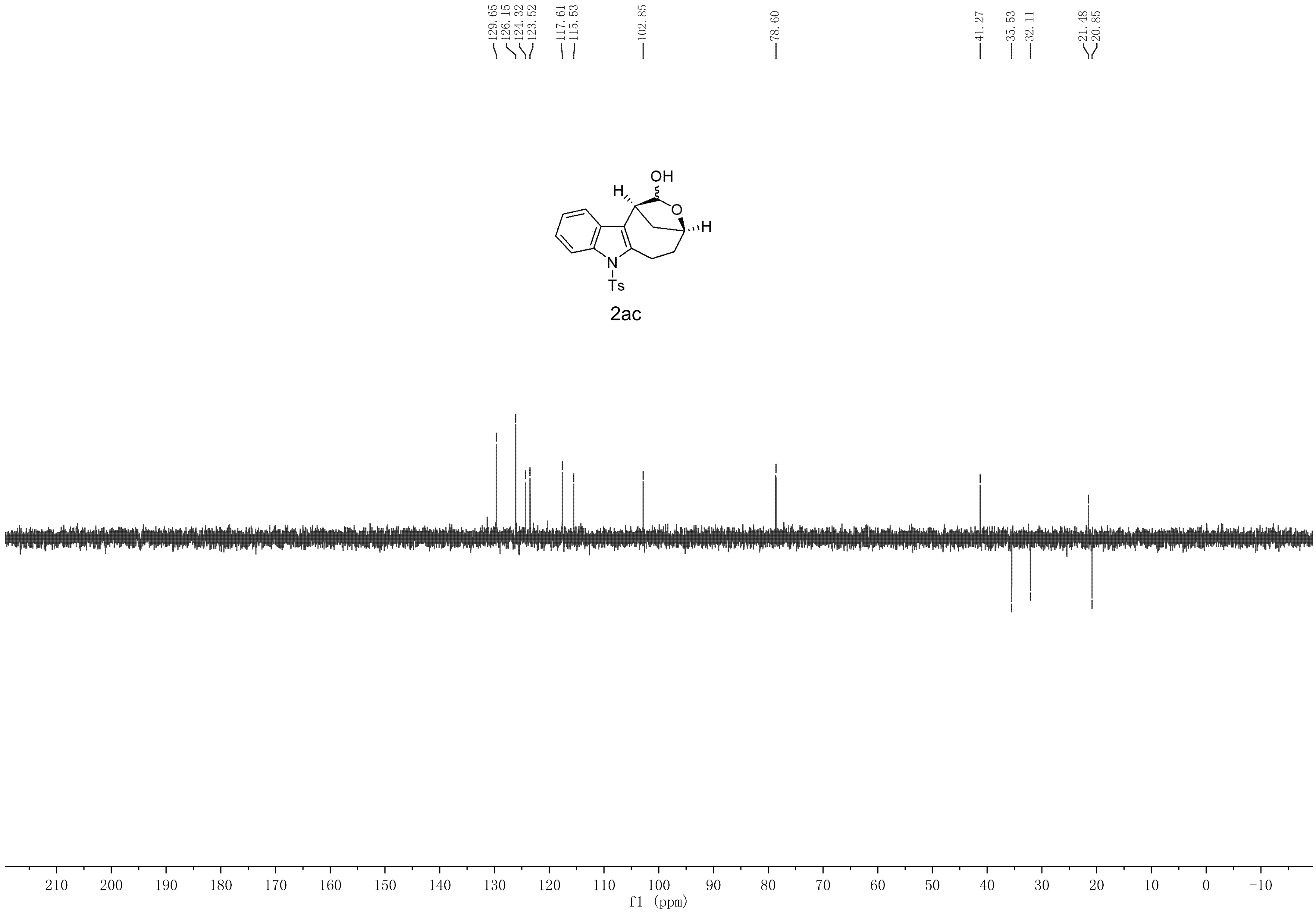
129.65
126.15
124.32
123.52
117.61
115.53
102.85
78.60
41.27
35.53
32.11
21.48
20.85
OH
H
O
H
N
Ts
2ac
210 200 190 180 170 160 150 140 130 120 110 100 90 80 70 60 50 40 30 20 10 0 −10
f1 (ppm)

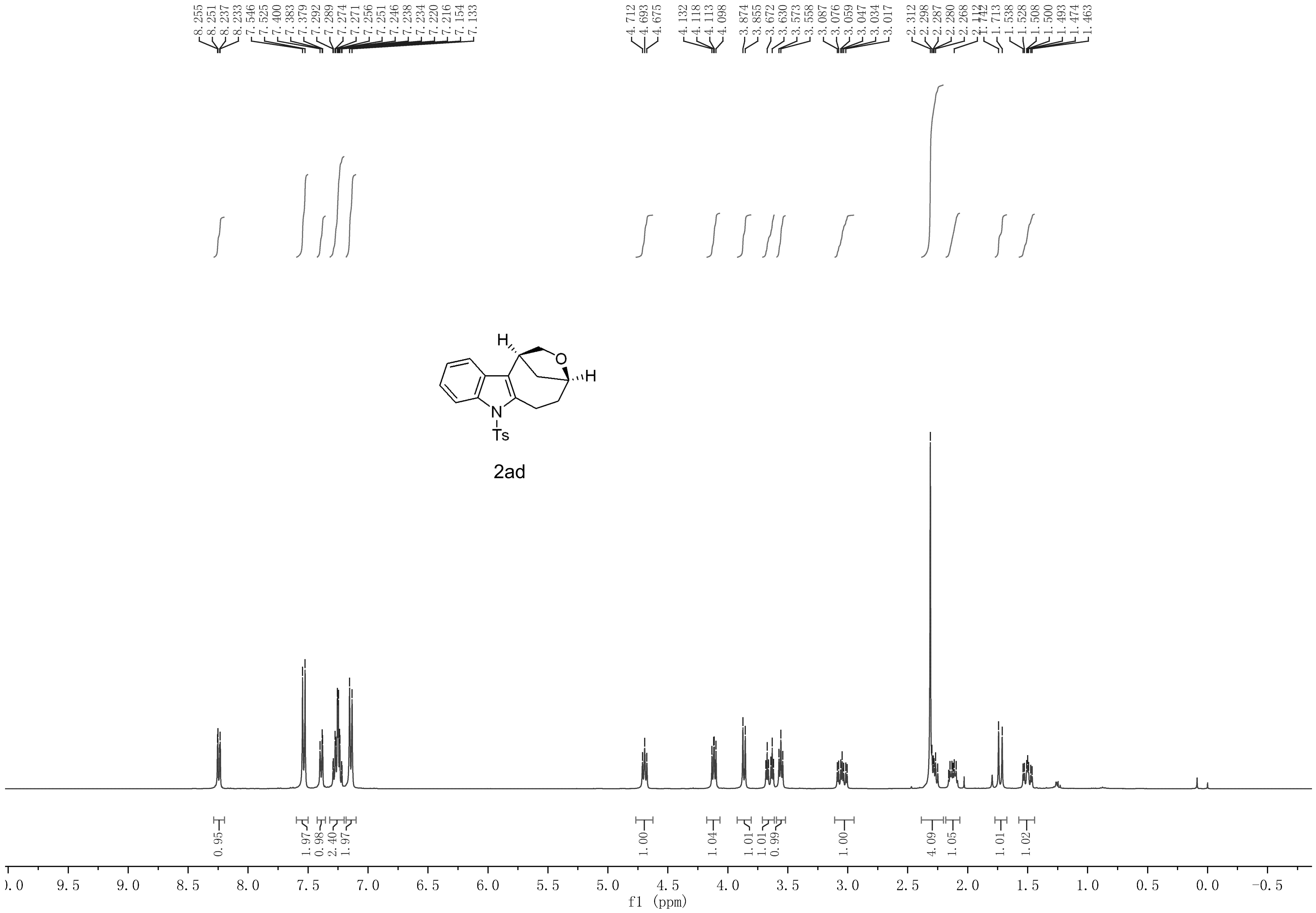
8.255
8.251
8.237
8.233
7.546
7.525
7.400
7.383
7.379
7.292
7.289
7.274
7.271
7.256
7.251
7.246
7.238
7.234
7.220
7.216
7.154
7.133
4.712
4.693
4.675
4.132
4.118
4.113
4.098
3.874
3.855
3.672
3.630
3.573
3.558
3.087
3.076
3.059
3.047
3.034
3.017
2.312
2.298
2.287
2.280
2.268
2.112
1.742
1.713
1.538
1.528
1.508
1.500
1.493
1.474
1.463
H
O
H
N
Ts
2ad
0.95
1.97
0.98
2.40
1.97
1.00
1.04
1.01
1.01
0.99
1.00
4.09
1.05
1.01
1.02
9.5
9.0
8.5
8.0
7.5
7.0
6.5
6.0
5.5
5.0
4.5
4.0
3.5
3.0
2.5
2.0
1.5
1.0
0.5
0.0
-0.5
f1 (ppm)

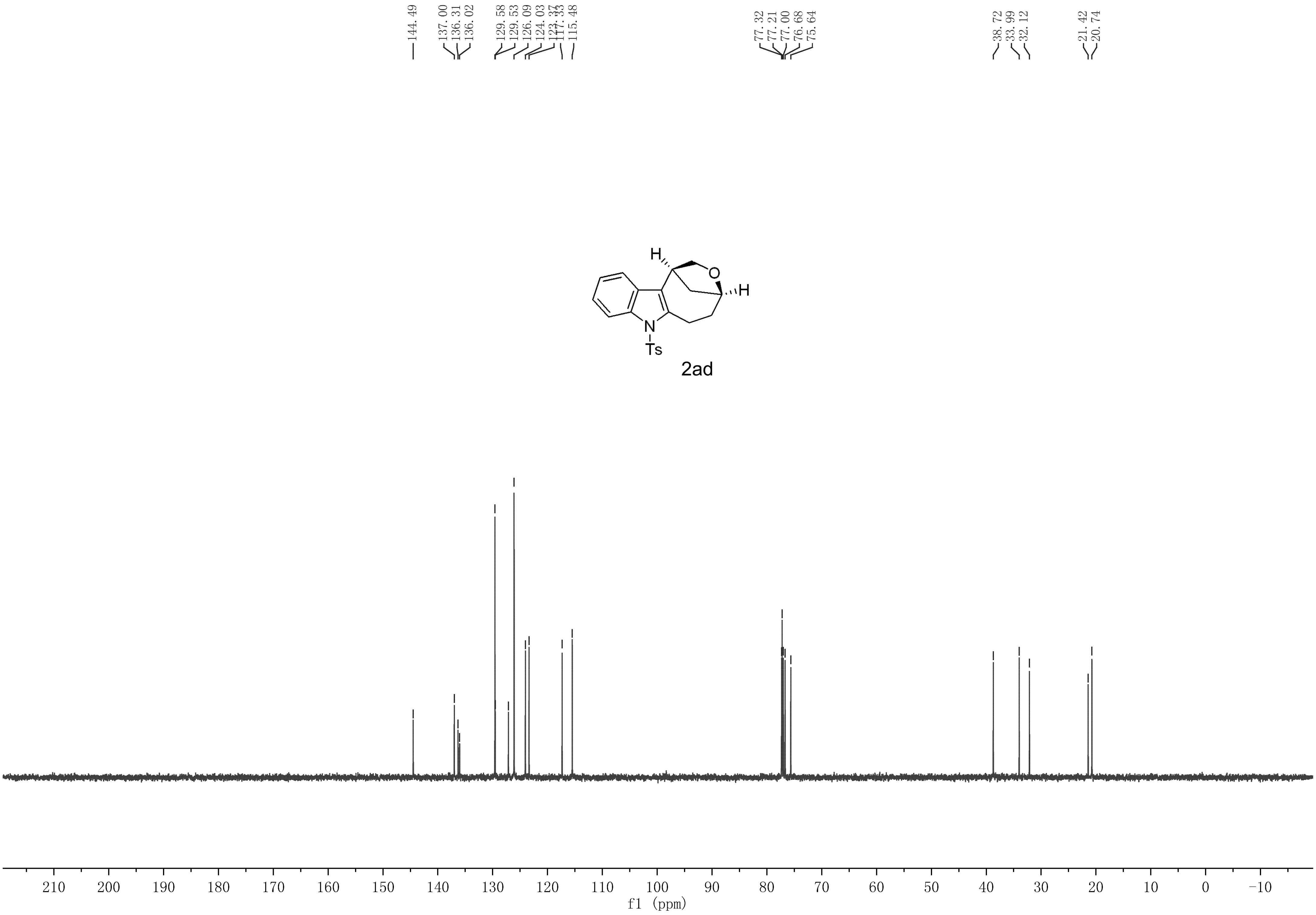
144.49
137.00
136.31
136.02
129.58
129.53
126.09
124.03
123.37
117.33
115.48
77.32
77.21
77.00
76.68
75.64
38.72
33.99
32.12
21.42
20.74
H
O
H
N
Ts
2ad
210 200 190 180 170 160 150 140 130 120 110 100 90 80 70 60 50 40 30 20 10 0 −10
f1 (ppm)

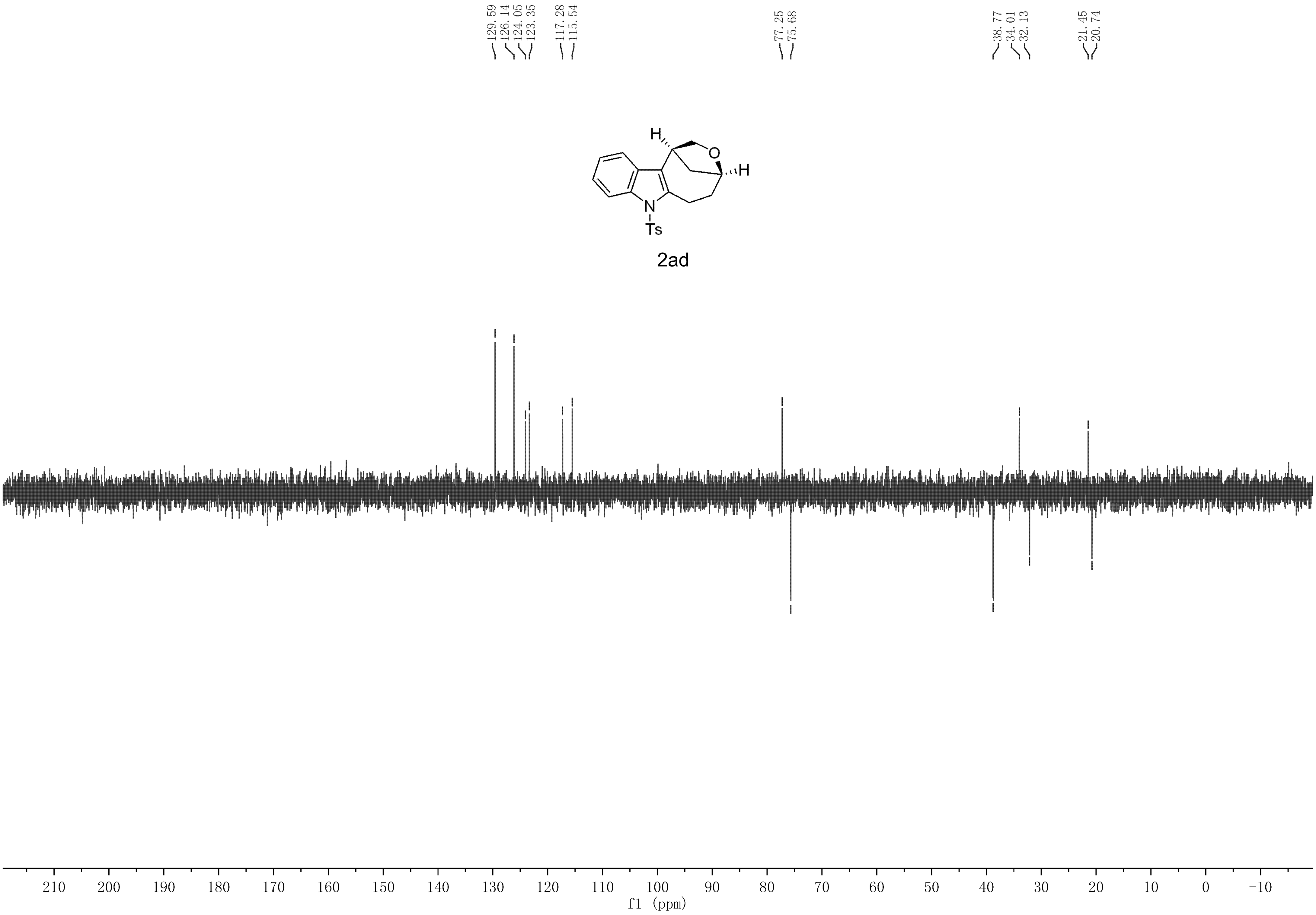
129.59
126.14
124.05
123.35
117.28
115.54
77.25
75.68
38.77
34.01
32.13
21.45
20.74
H
O
H
N
Ts
2ad
210 200 190 180 170 160 150 140 130 120 110 100 90 80 70 60 50 40 30 20 10 0 −10
f1 (ppm)

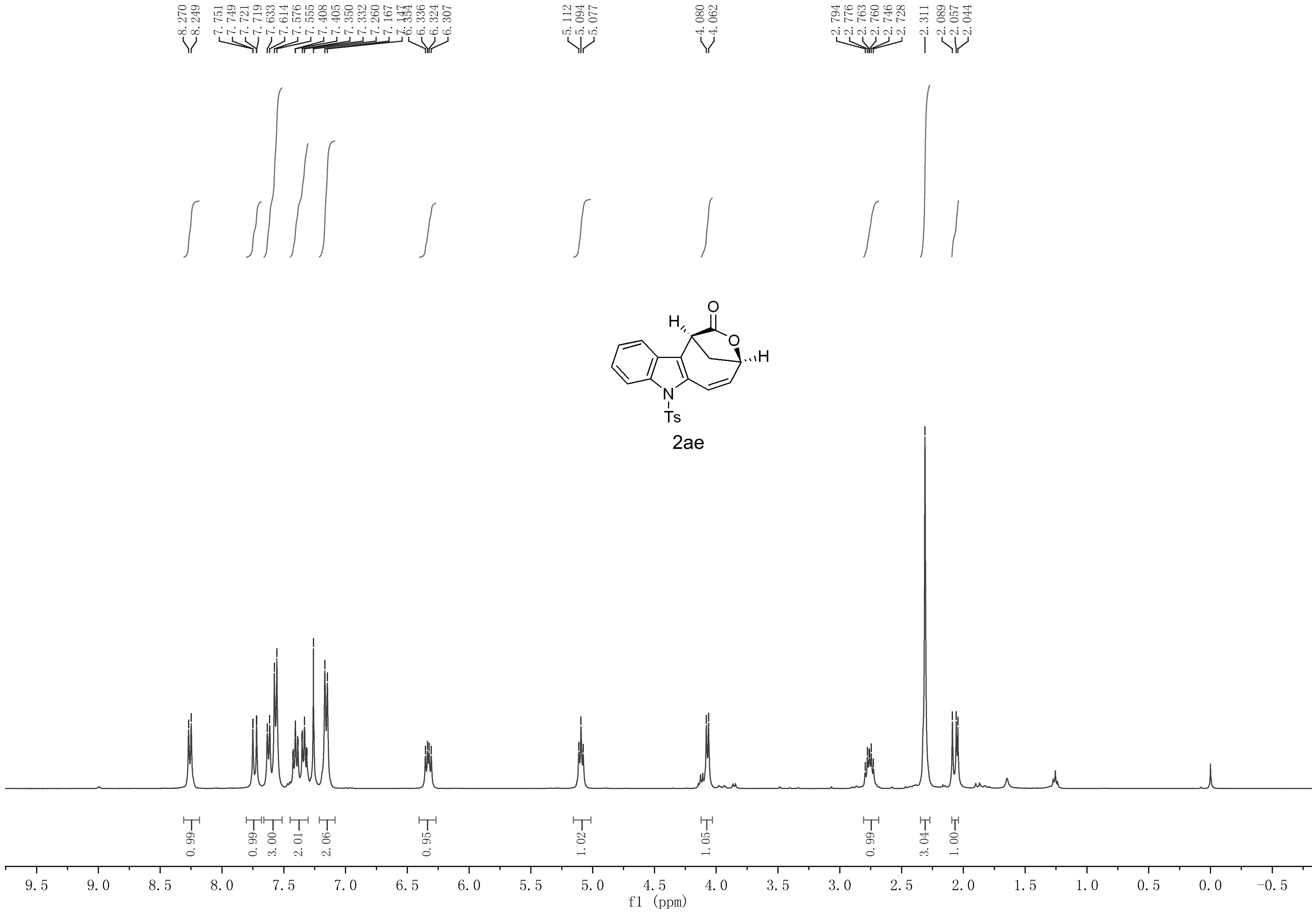
8.270
8.249
7.751
7.749
7.721
7.719
7.633
7.614
7.576
7.555
7.408
7.405
7.350
7.332
7.260
7.167
7.147
6.354
6.336
6.324
6.307
5.112
5.094
5.077
4.080
4.062
2.794
2.776
2.763
2.760
2.746
2.728
2.311
2.089
2.057
2.044
H
O
O
H
N
Ts
2ae
0.99
0.99
3.00
2.01
2.06
0.95
1.02
1.05
0.99
3.04
1.00
9.5
9.0
8.5
8.0
7.5
7.0
6.5
6.0
5.5
5.0
4.5
4.0
3.5
3.0
2.5
2.0
1.5
1.0
0.5
0.0
−0.5
f1 (ppm)

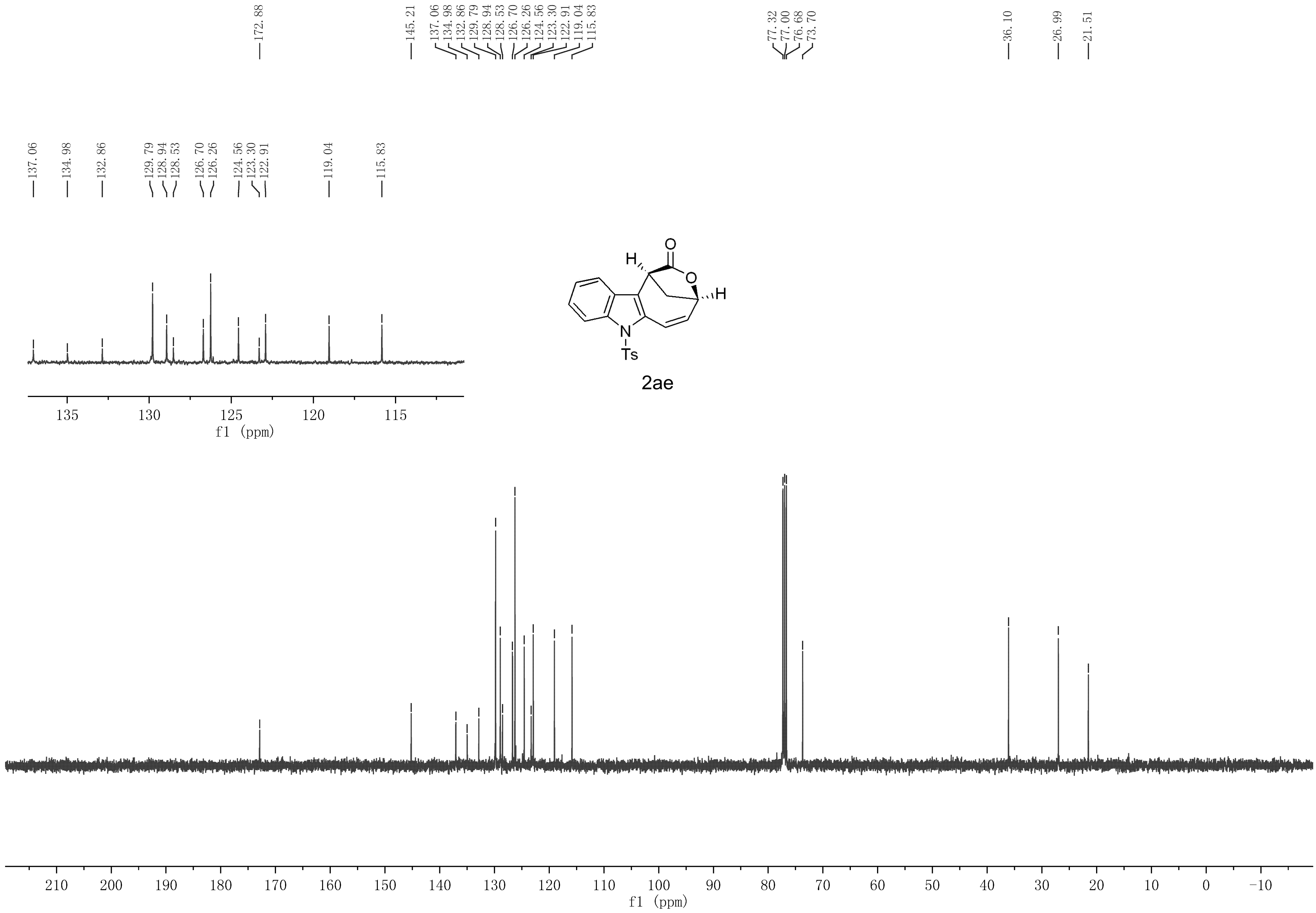

172.88
145.21
137.06
134.98
132.86
129.79
128.94
128.53
126.70
126.26
124.56
123.30
122.91
119.04
115.83
77.32
77.00
76.68
73.70
36.10
26.99
21.51
O
H
O
H
N
Ts
2ae
135
130
125
120
115
f1 (ppm)
210
200
190
180
170
160
150
140
130
120
110
100
90
80
70
60
50
40
30
20
10
0
−10
f1 (ppm)

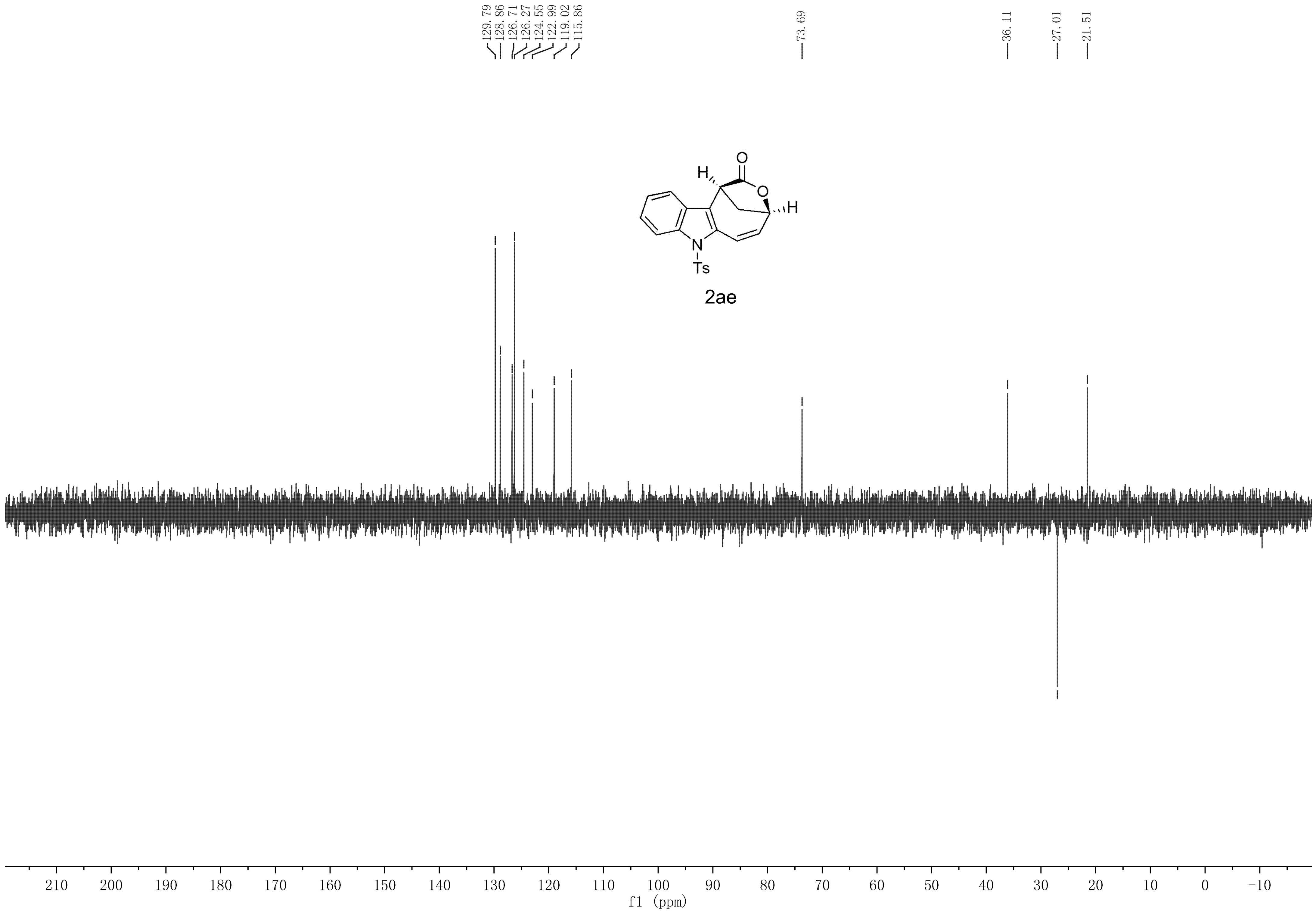

129.79
128.86
126.71
126.27
124.55
122.99
119.02
115.86
73.69
36.11
27.01
21.51
H
O
O
H
N
Ts
2ae
210 200 190 180 170 160 150 140 130 120 110 100 90 80 70 60 50 40 30 20 10 0 −10
f1 (ppm)

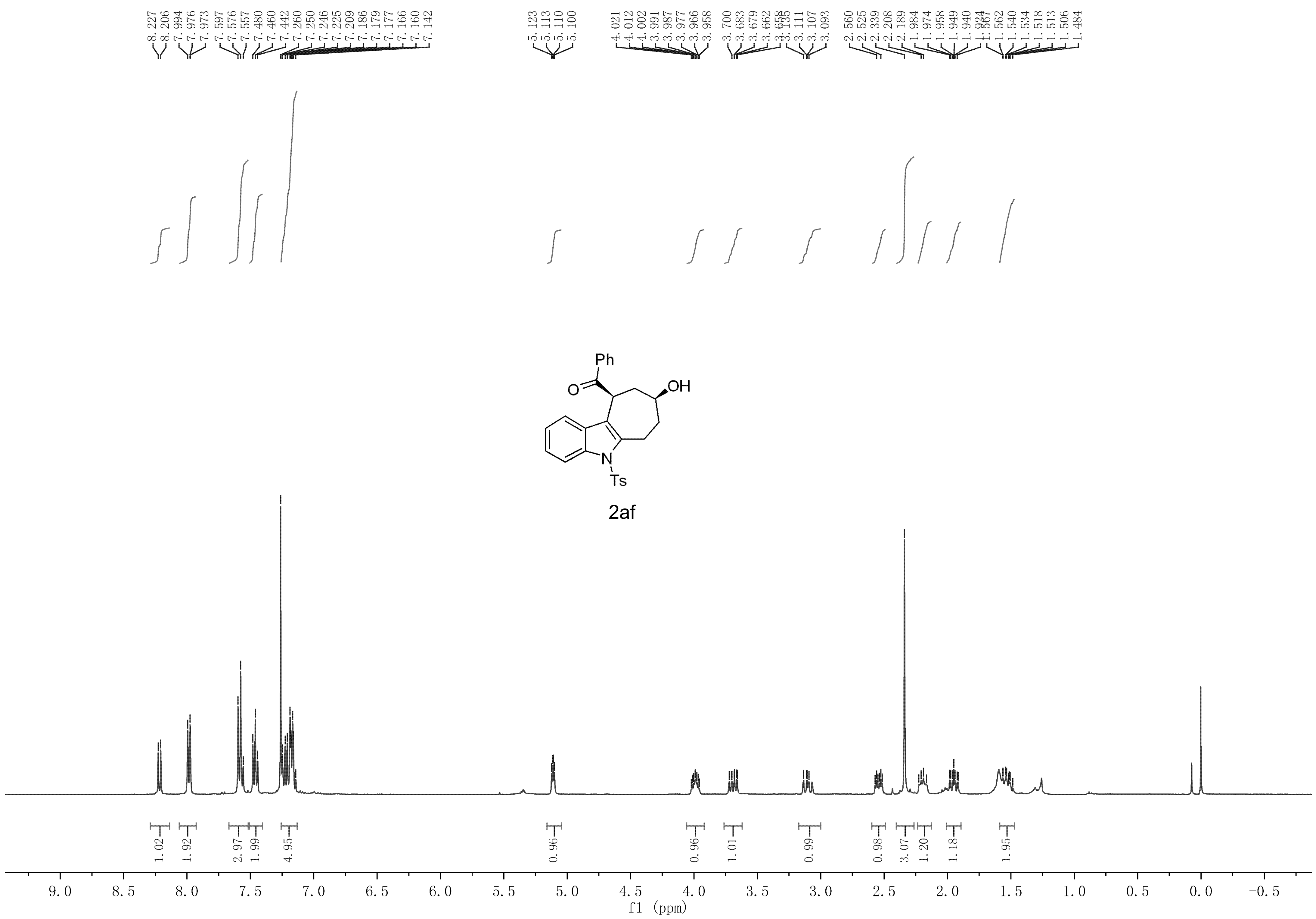
8.227
8.206
7.994
7.976
7.973
7.597
7.576
7.557
7.480
7.460
7.442
7.260
7.250
7.246
7.225
7.209
7.186
7.179
7.177
7.166
7.160
7.142
5.123
5.113
5.110
5.100
4.021
4.012
4.002
3.991
3.987
3.977
3.966
3.958
3.700
3.683
3.679
3.662
3.135
3.111
3.107
3.093
2.560
2.525
2.339
2.208
2.189
1.984
1.974
1.958
1.949
1.940
1.562
1.540
1.534
1.518
1.513
1.506
1.484
Ph
O
OH
N
Ts
2af
1.02
1.92
2.97
1.99
4.95
0.96
0.96
1.01
0.99
0.98
3.07
1.20
1.18
1.95
9.0
8.5
8.0
7.5
7.0
6.5
6.0
5.5
5.0
4.5
4.0
3.5
3.0
2.5
2.0
1.5
1.0
0.5
0.0
-0.5
f1 (ppm)

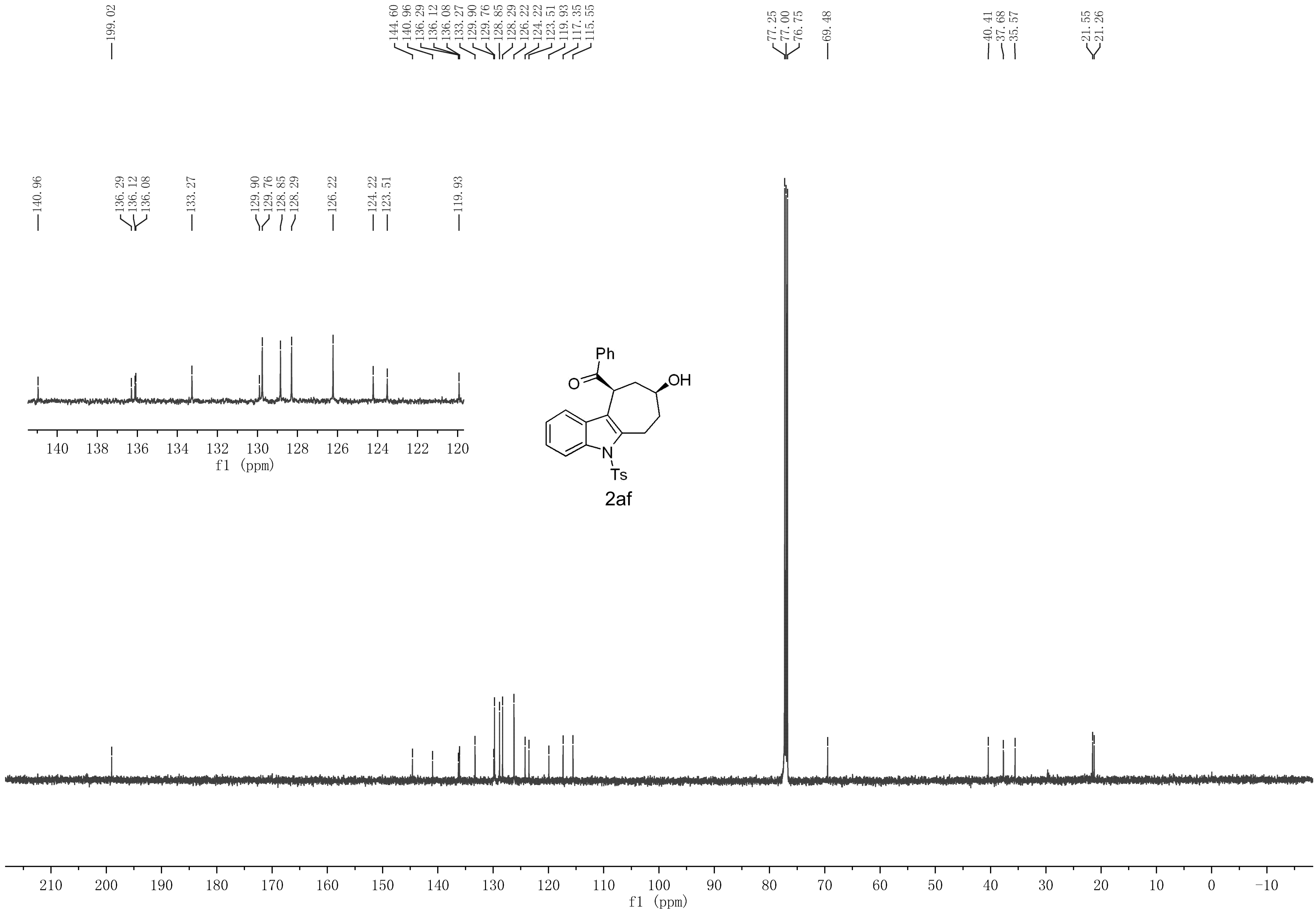
199.02
144.60
140.96
136.29
136.12
136.08
133.27
129.90
129.76
128.85
128.29
126.22
124.22
123.51
119.93
117.35
115.55
77.25
77.00
76.75
69.48
40.41
37.68
35.57
21.55
21.26
Ph
O
OH
N
Ts
2af
f1 (ppm)

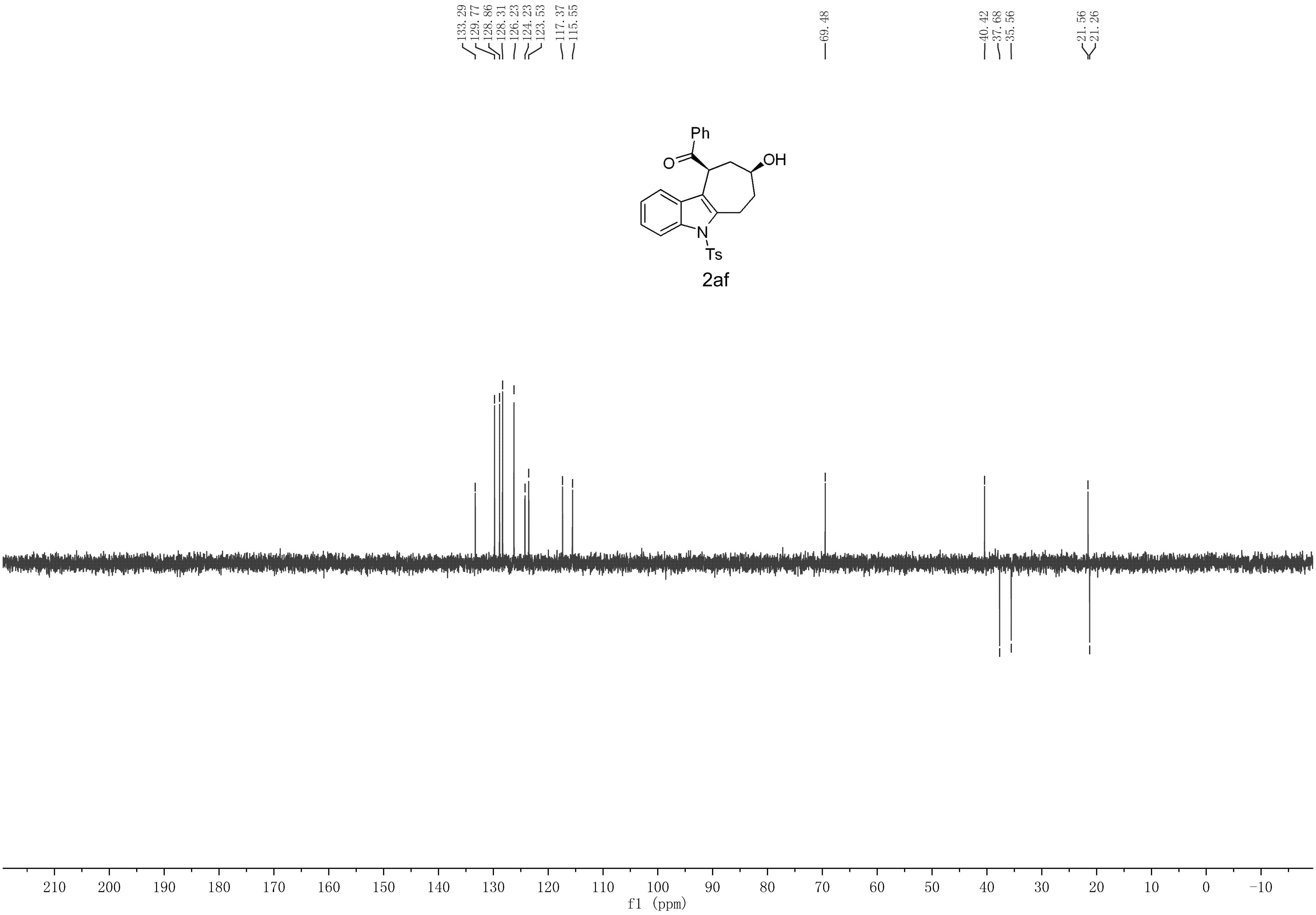
133.29
129.77
128.86
128.31
126.23
124.23
123.53
117.37
115.55
69.48
40.42
37.68
35.56
21.56
21.26
210
200
190
180
170
160
150
140
130
120
110
100
90
80
70
60
50
40
30
20
10
0
−10
f1 (ppm)
Ph
O
OH
N
Ts

2af

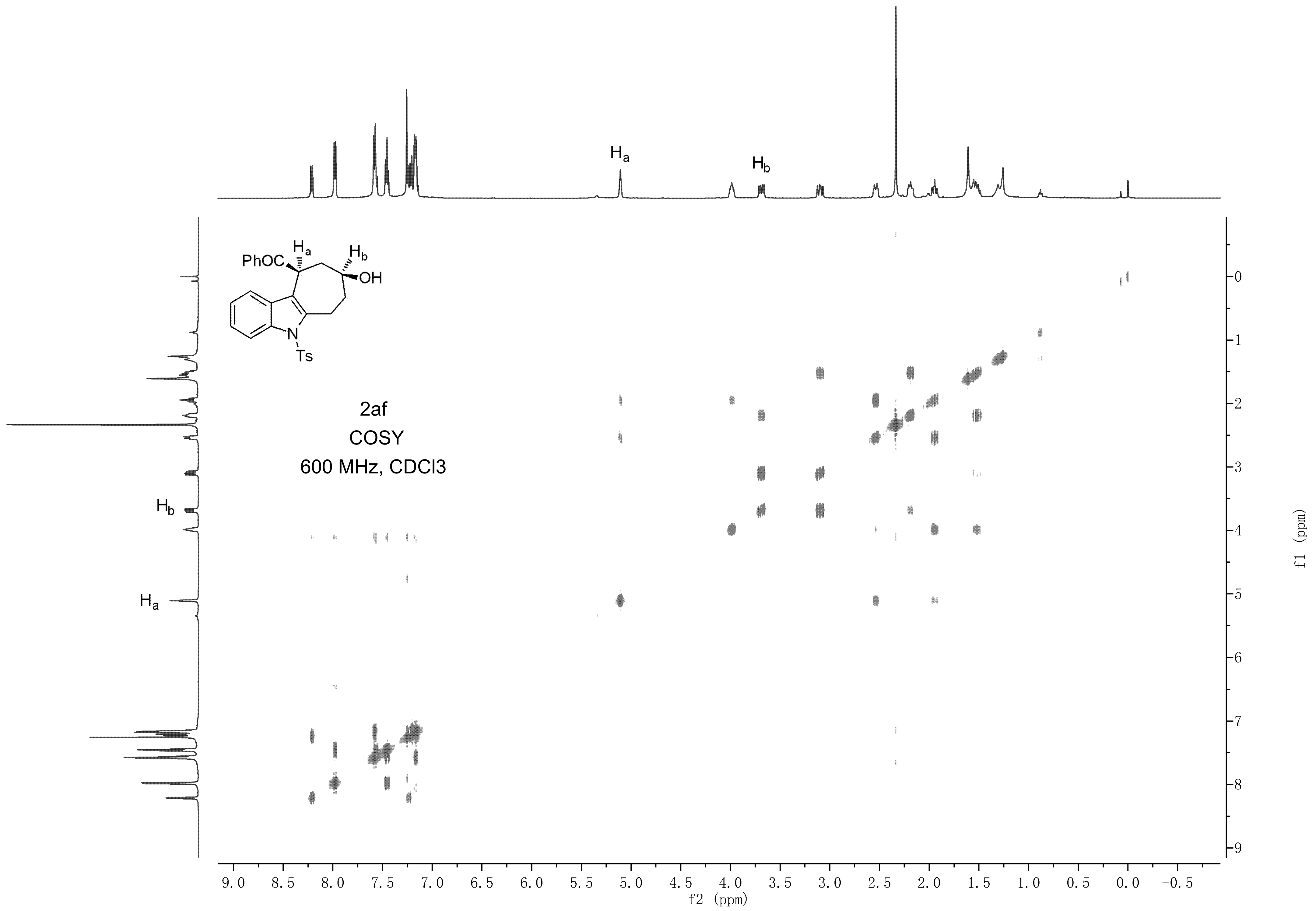

Ha
Hb
PhOC
OH
N
Ts
2af
COSY
600 MHz, CDCl3
f2 (ppm)
f1 (ppm)
9.0 8.5 8.0 7.5 7.0 6.5 6.0 5.5 5.0 4.5 4.0 3.5 3.0 2.5 2.0 1.5 1.0 0.5 0.0 −0.5
0 1 2 3 4 5 6 7 8 9

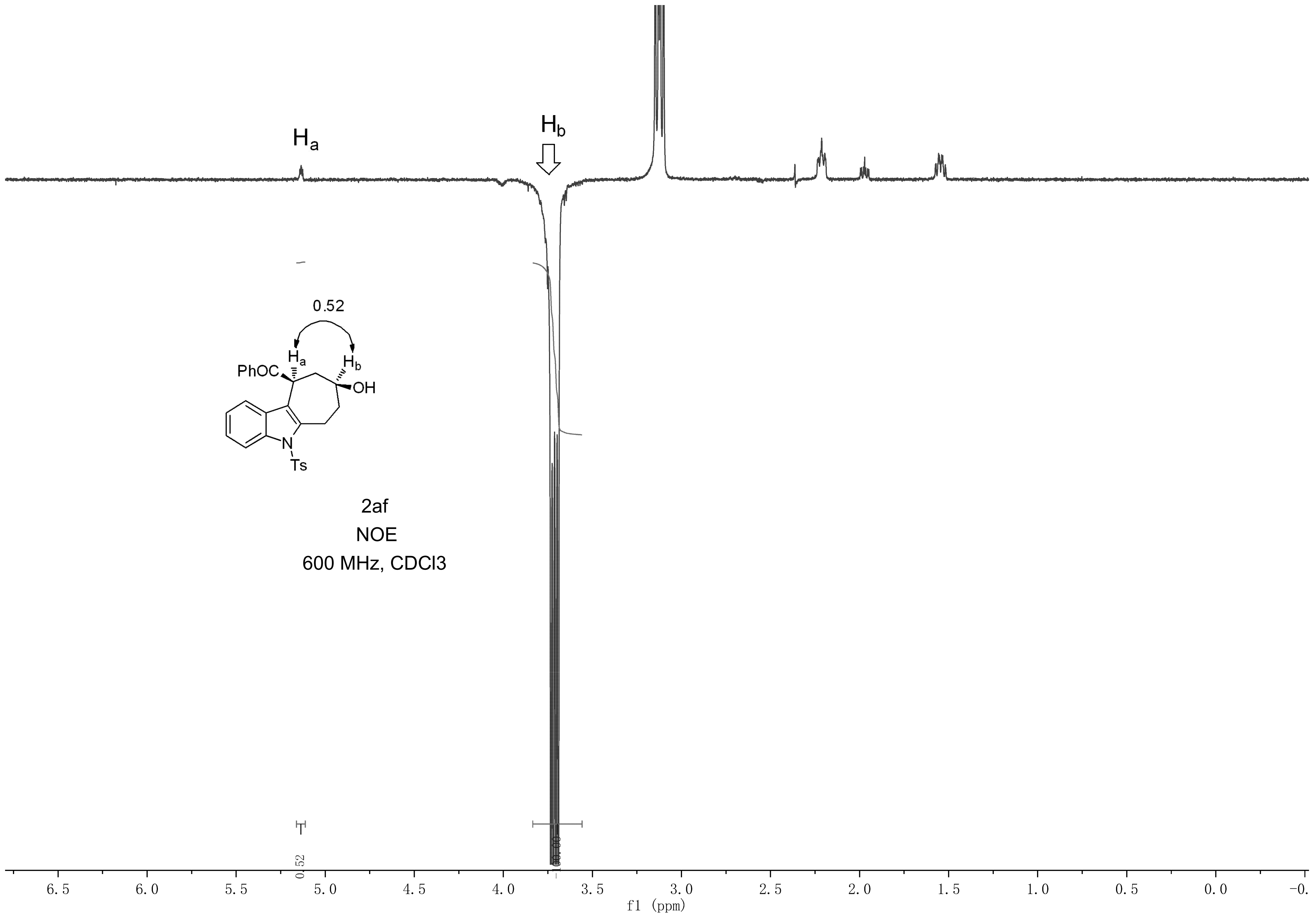
H
a
H
b
0.52
PhOC
OH
N
Ts
2af
NOE
600 MHz, CDCl3
0.52
100.00
6.5
6.0
5.5
5.0
4.5
4.0
3.5
3.0
2.5
2.0
1.5
1.0
0.5
0.0
-0.
f1 (ppm)

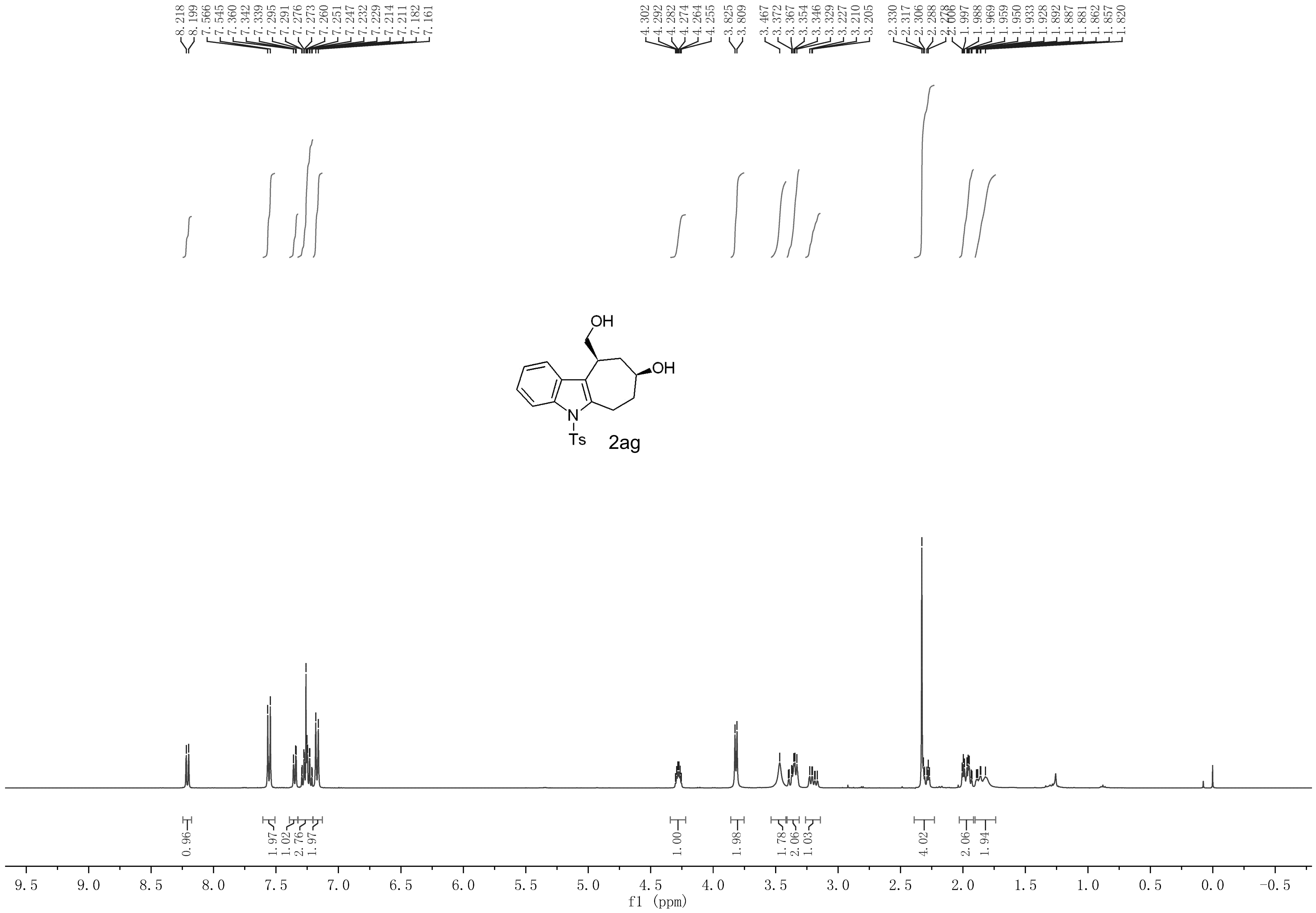
8.218
8.199
7.566
7.545
7.360
7.342
7.339
7.295
7.291
7.276
7.273
7.260
7.251
7.247
7.232
7.229
7.214
7.211
7.182
7.161
4.302
4.292
4.282
4.274
4.264
4.255
3.825
3.809
3.467
3.372
3.367
3.354
3.346
3.329
3.227
3.210
3.205
2.330
2.317
2.306
2.288
1.997
1.988
1.969
1.959
1.950
1.933
1.928
1.892
1.887
1.881
1.862
1.857
1.820
OH
OH
N
Ts
2ag
0.96
1.97
1.02
2.76
1.97
1.00
1.98
1.78
2.06
1.03
4.02
2.06
1.94
9.5
9.0
8.5
8.0
7.5
7.0
6.5
6.0
5.5
5.0
4.5
4.0
3.5
3.0
2.5
2.0
1.5
1.0
0.5
0.0
-0.5
f1 (ppm)

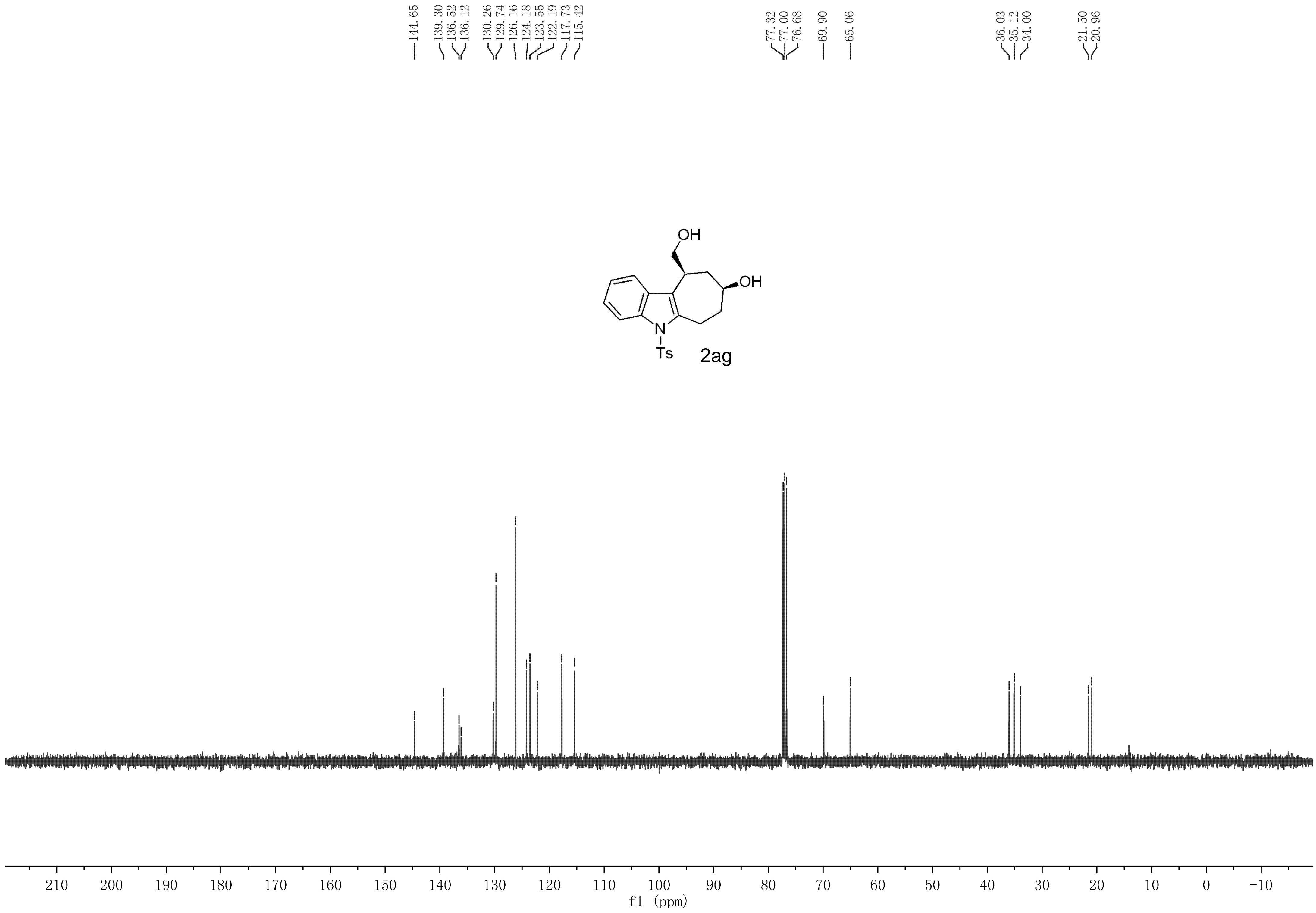
144.65
139.30
136.52
136.12
130.26
129.74
126.16
124.18
123.55
122.19
117.73
115.42
77.32
77.00
76.68
69.90
65.06
36.03
35.12
34.00
21.50
20.96
OH
OH
N
Ts
2ag
210 200 190 180 170 160 150 140 130 120 110 100 90 80 70 60 50 40 30 20 10 0 −10
f1 (ppm)

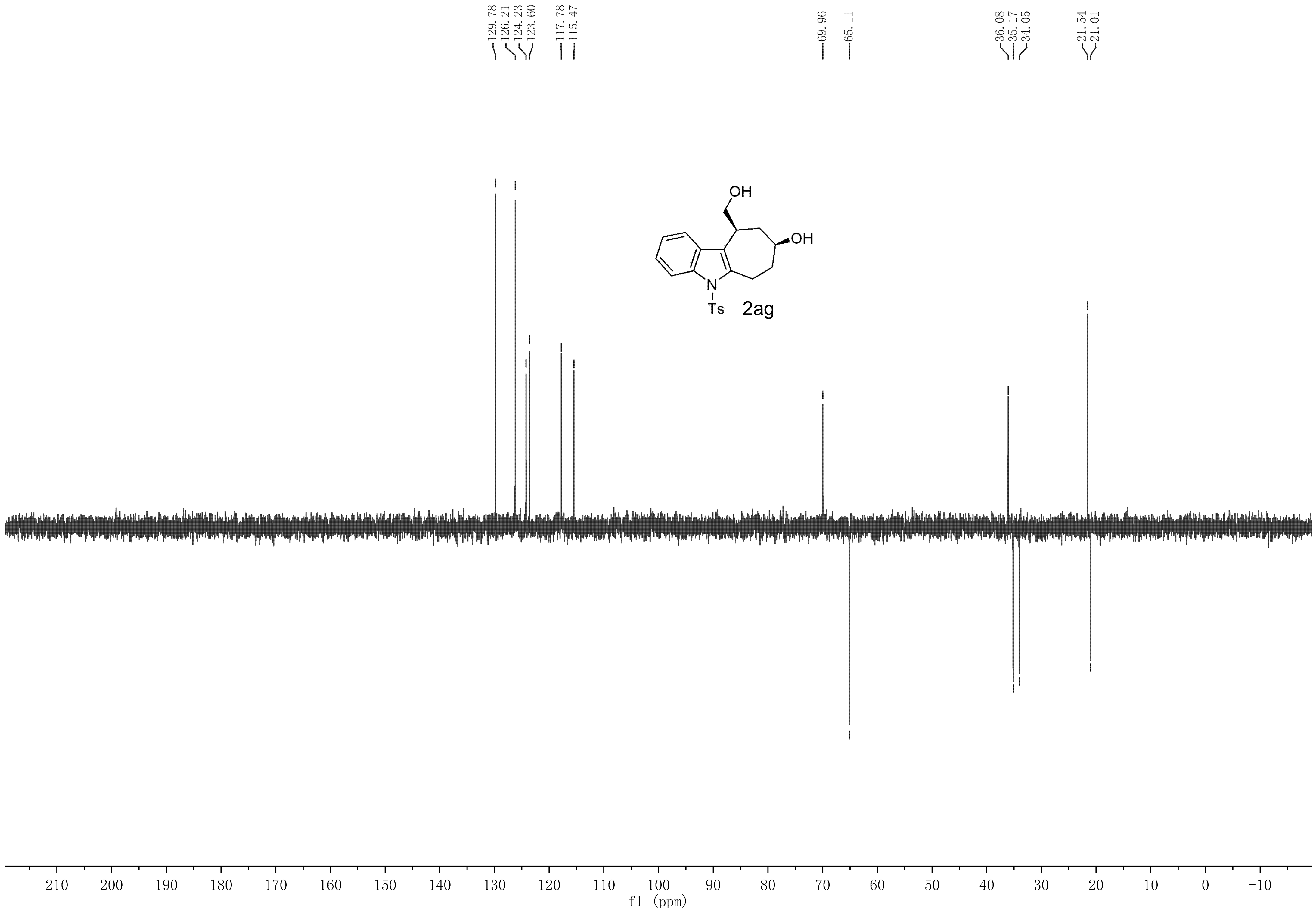
129.78
126.21
124.23
123.60
117.78
115.47
69.96
65.11
36.08
35.17
34.05
21.54
21.01
OH
OH
N
Ts
2ag
210
200
190
180
170
160
150
140
130
120
110
100
90
80
70
60
50
40
30
20
10
0
−10
f1 (ppm)

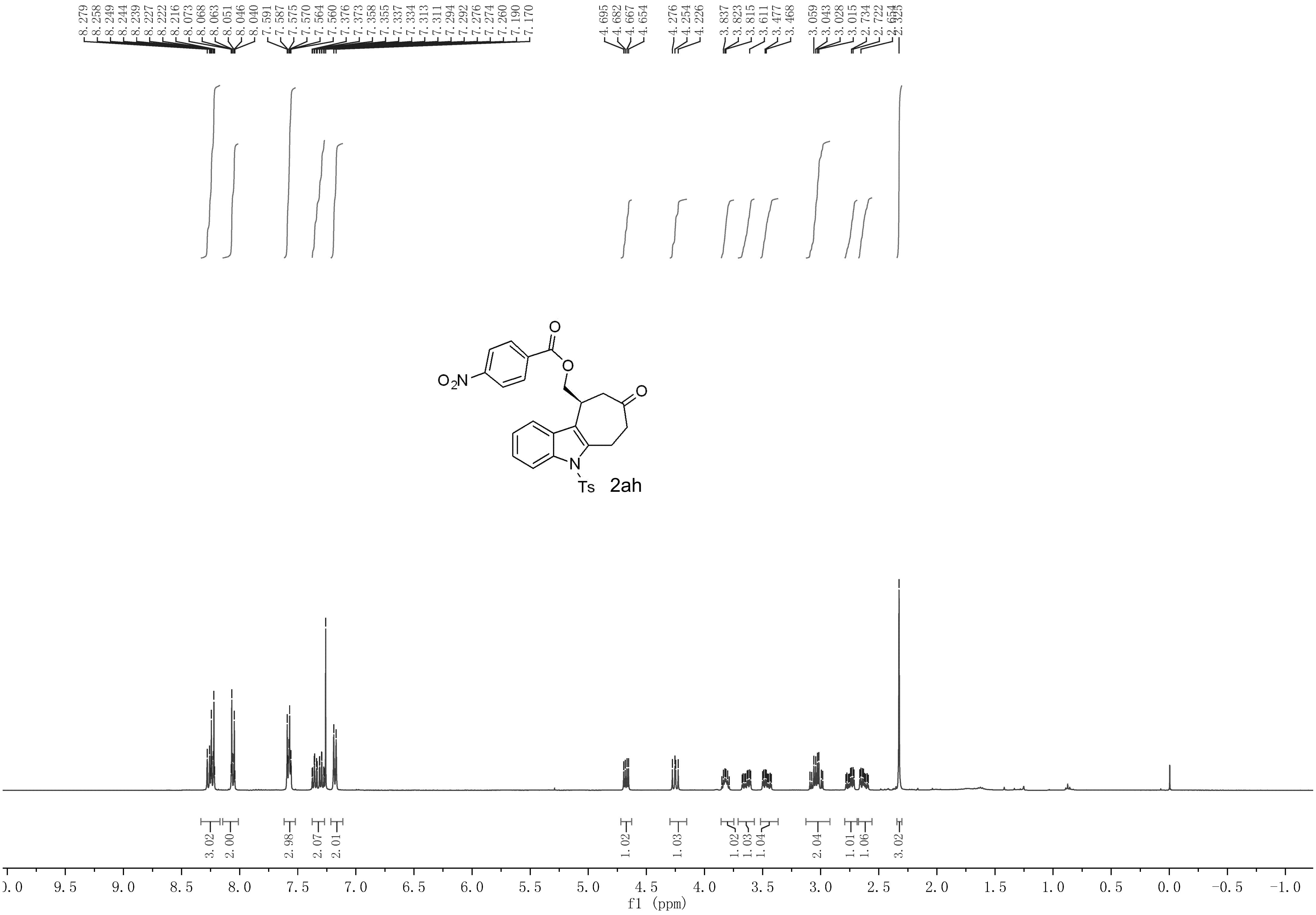
8.279 8.258 8.249 8.244 8.239 8.227 8.222 8.216 8.073 8.068 8.063 8.051 8.046 8.040 7.591 7.587 7.575 7.570 7.564 7.560 7.376 7.373 7.358 7.355 7.337 7.334 7.313 7.311 7.294 7.292 7.276 7.274 7.260 7.190 7.170
4.695 4.682 4.667 4.654
4.276 4.254 4.226
3.837 3.823 3.815 3.611 3.477 3.468
3.059 3.043 3.028 3.015 2.734 2.722
2.325
O
O
O2N
O
N
Ts
2ah
3.02 2.00 2.98 2.07 2.01 1.02 1.03 1.02 1.03 1.04 2.04 1.01 1.06 3.02
9.5 9.0 8.5 8.0 7.5 7.0 6.5 6.0 5.5 5.0 4.5 4.0 3.5 3.0 2.5 2.0 1.5 1.0 0.5 0.0 -0.5 -1.0
f1 (ppm)

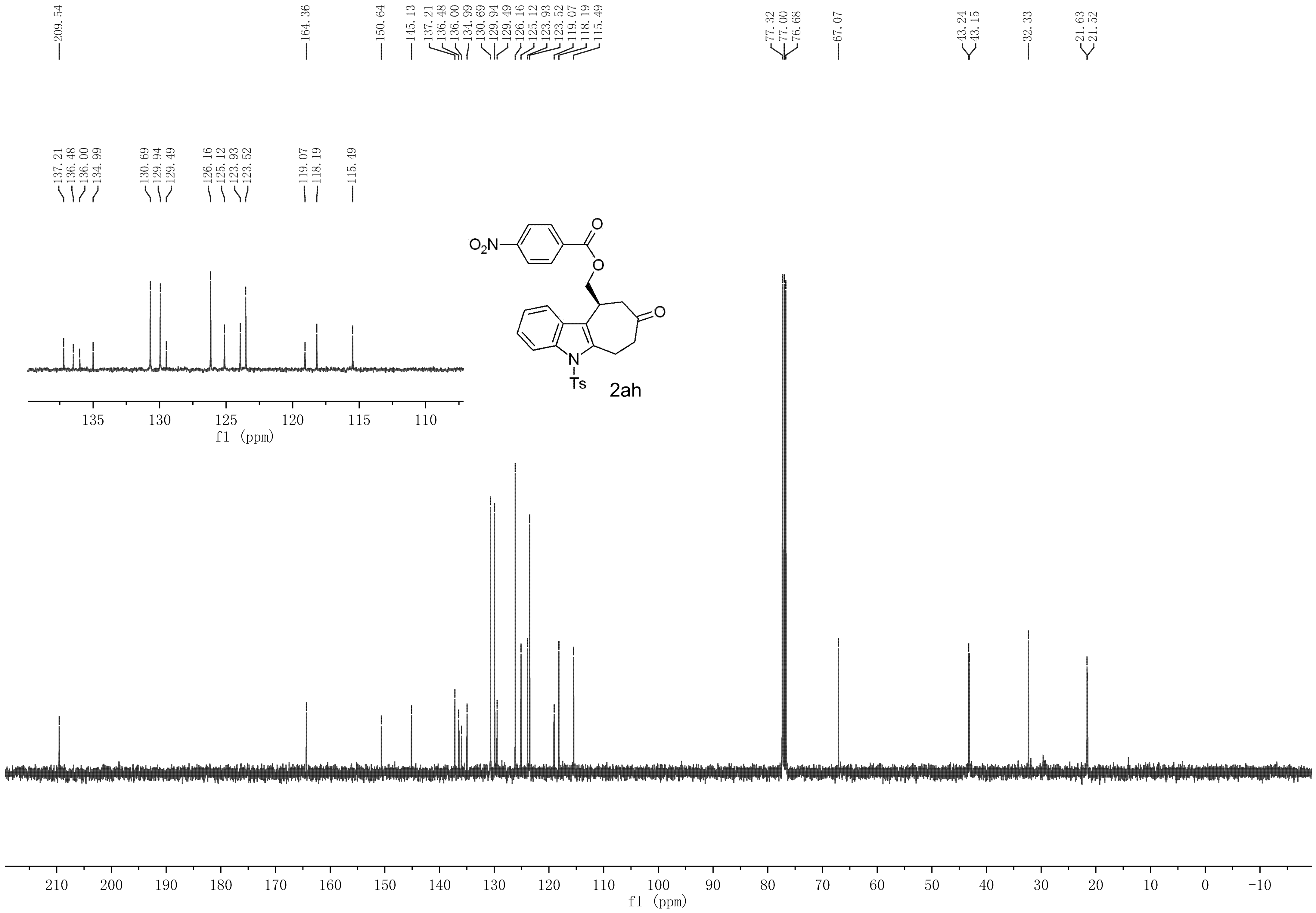
209.54
164.36
150.64
145.13
137.21
136.48
136.00
134.99
130.69
129.94
129.49
126.16
125.12
123.93
123.52
119.07
118.19
115.49
77.32
77.00
76.68
67.07
43.24
43.15
32.33
21.63
21.52
O2N
O
O
O
N
Ts
2ah
f1 (ppm)
f1 (ppm)

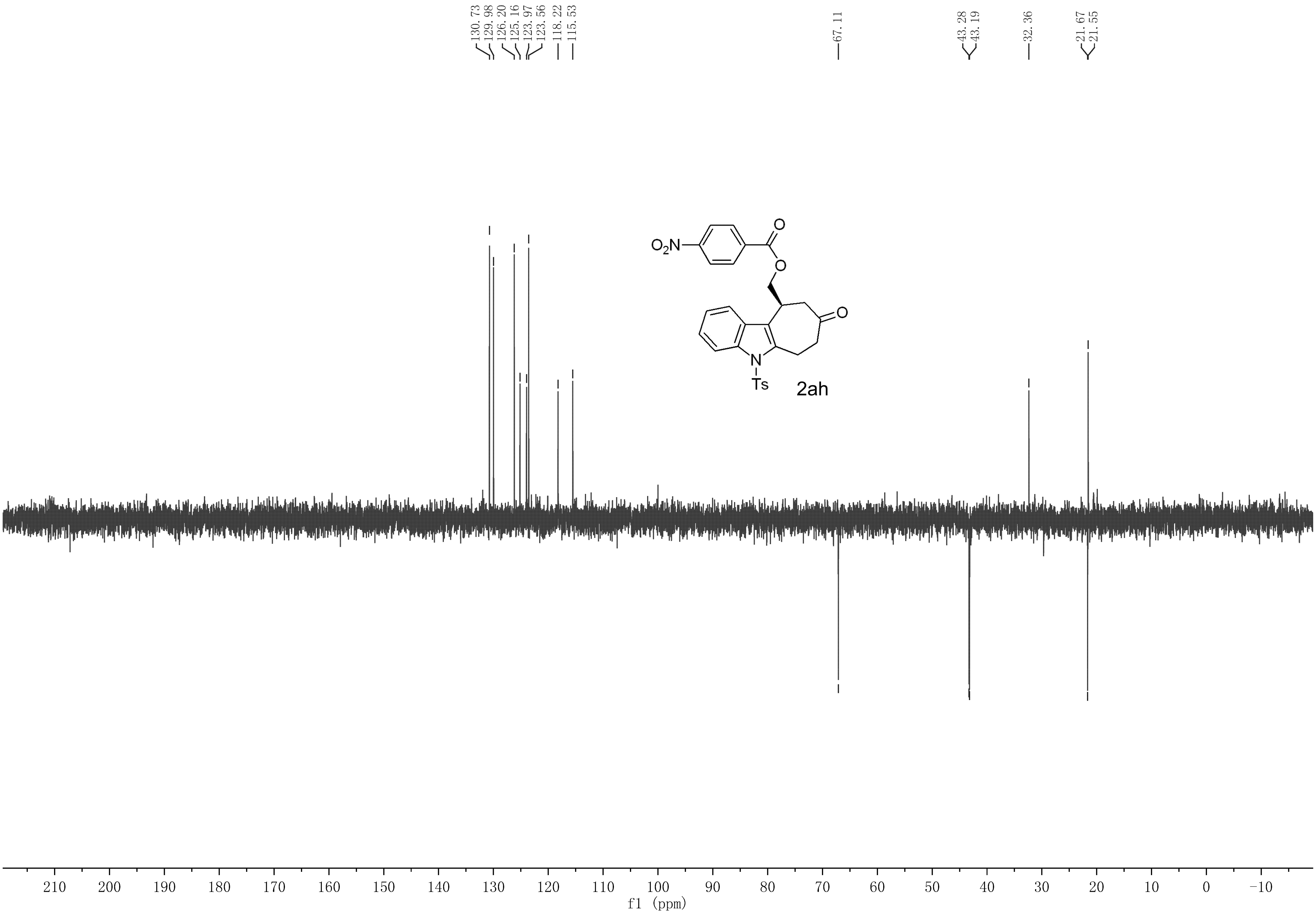
130.73
129.98
126.20
125.16
123.97
123.56
118.22
115.53
67.11
43.28
43.19
32.36
21.67
21.55
O2N
O
O
O
N
Ts
2ah
210 200 190 180 170 160 150 140 130 120 110 100 90 80 70 60 50 40 30 20 10 0 −10
f1 (ppm)

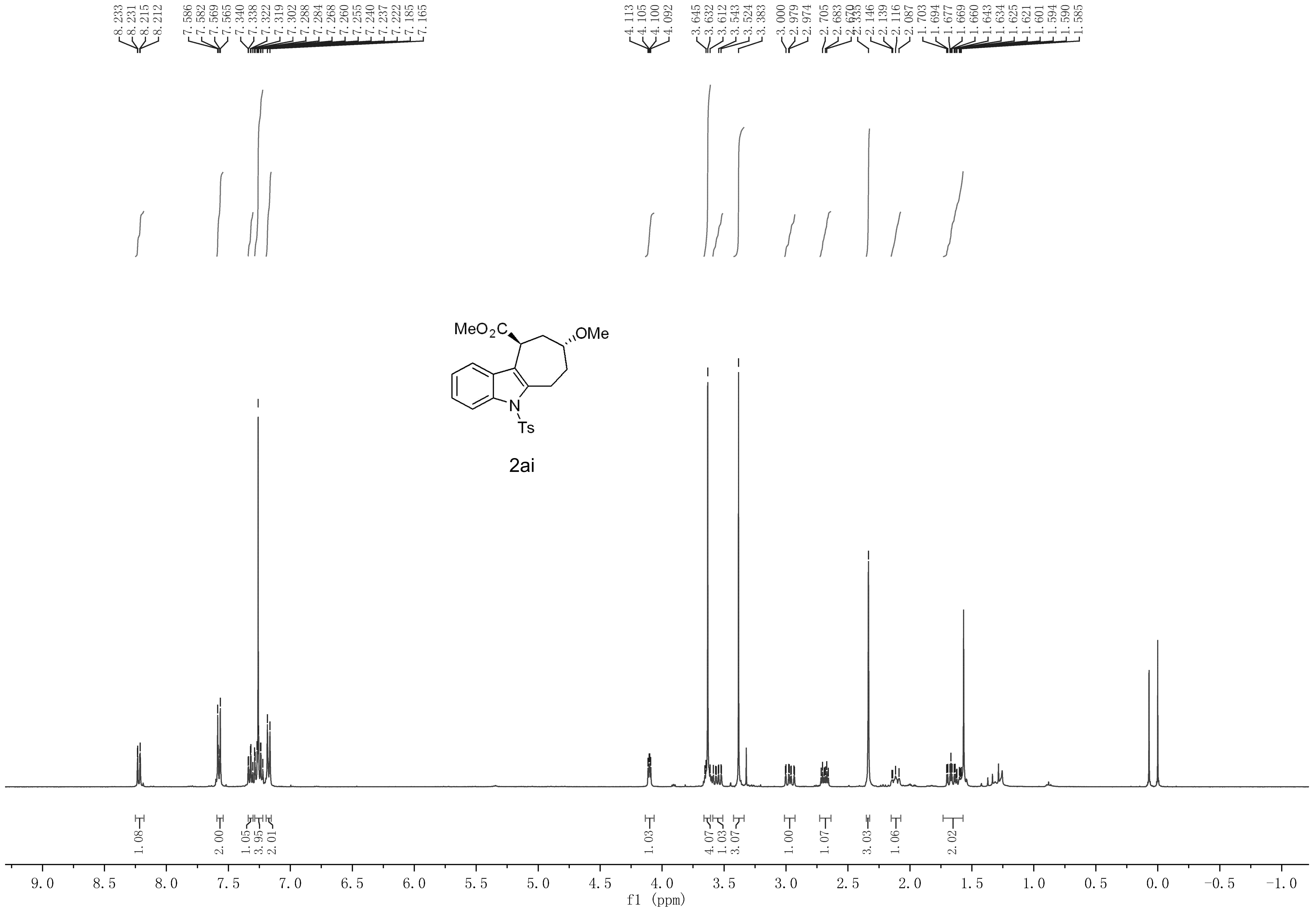
8.233
8.231
8.215
8.212
7.586
7.582
7.569
7.565
7.340
7.338
7.322
7.319
7.302
7.288
7.284
7.268
7.260
7.255
7.240
7.237
7.222
7.185
7.165
4.113
4.105
4.100
4.092
3.645
3.632
3.612
3.543
3.524
3.383
3.000
2.979
2.974
2.705
2.683
2.670
2.335
2.146
2.139
2.116
2.087
1.703
1.694
1.677
1.669
1.660
1.643
1.634
1.625
1.621
1.601
1.594
1.590
1.585
MeO2C
OMe
N
Ts
2ai
1.08
2.00
1.05
3.95
2.01
1.03
4.07
1.03
3.07
1.00
1.07
3.03
1.06
2.02
9.0
8.5
8.0
7.5
7.0
6.5
6.0
5.5
5.0
4.5
4.0
3.5
3.0
2.5
2.0
1.5
1.0
0.5
0.0
-0.5
-1.0
f1 (ppm)

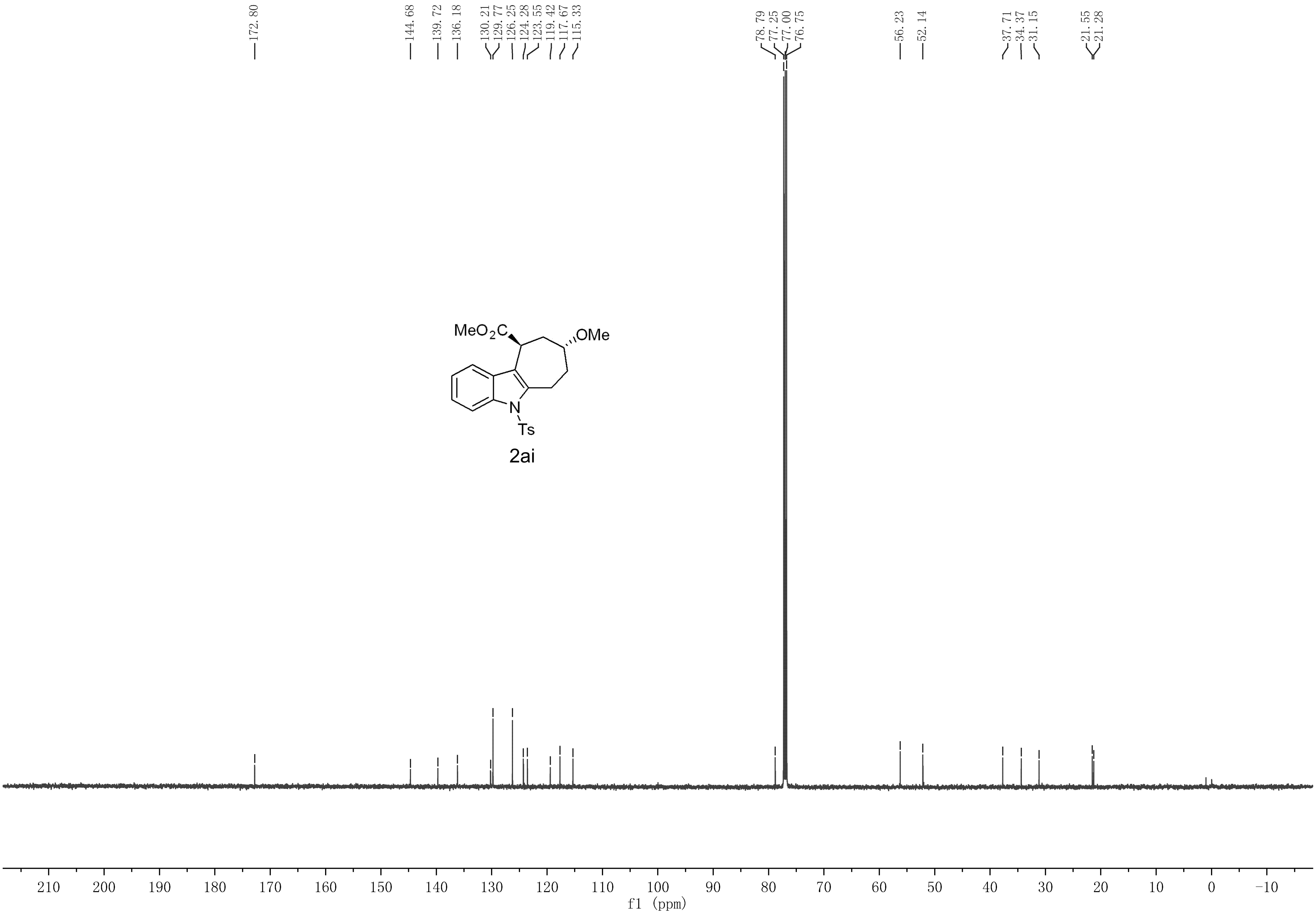
172.80
144.68
139.72
136.18
130.21
129.77
126.25
124.28
123.55
119.42
117.67
115.33
78.79
77.25
77.00
76.75
56.23
52.14
37.71
34.37
31.15
21.55
21.28
MeO2C
OMe
N
Ts
2ai
210 200 190 180 170 160 150 140 130 120 110 100 90 80 70 60 50 40 30 20 10 0 −10
f1 (ppm)

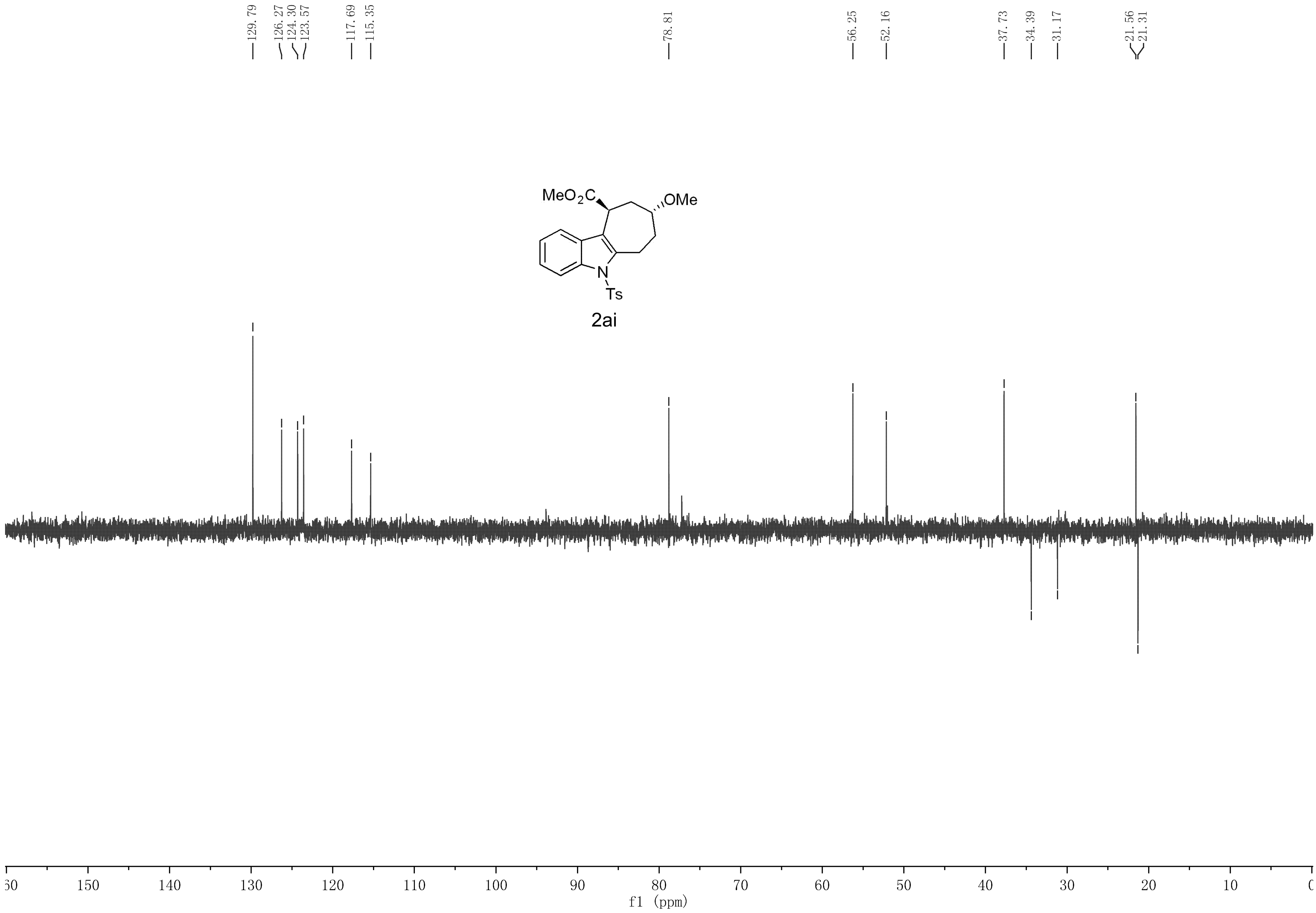
129.79
126.27
124.30
123.57
117.69
115.35
78.81
56.25
52.16
37.73
34.39
31.17
21.56
21.31
MeO2C
OMe
N
Ts
2ai
150
140
130
120
110
100
90
80
70
60
50
40
30
20
10
f1 (ppm)

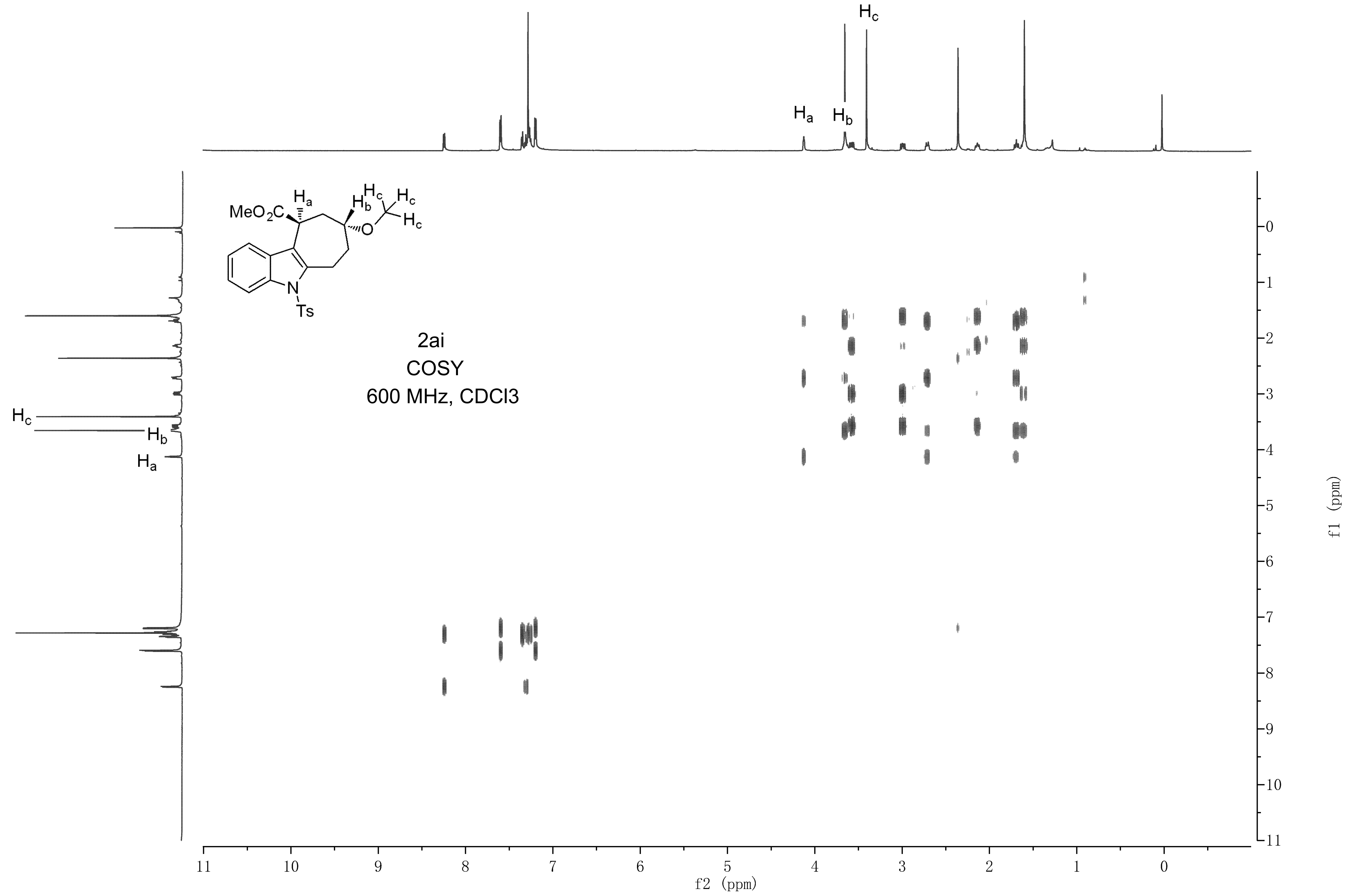

MeO2C
Ha
Hb
Hc
Hc
Hc
O
N
Ts
2ai
COSY
600 MHz, CDCl3
Ha
Hb
Hc
f2 (ppm)
f1 (ppm)

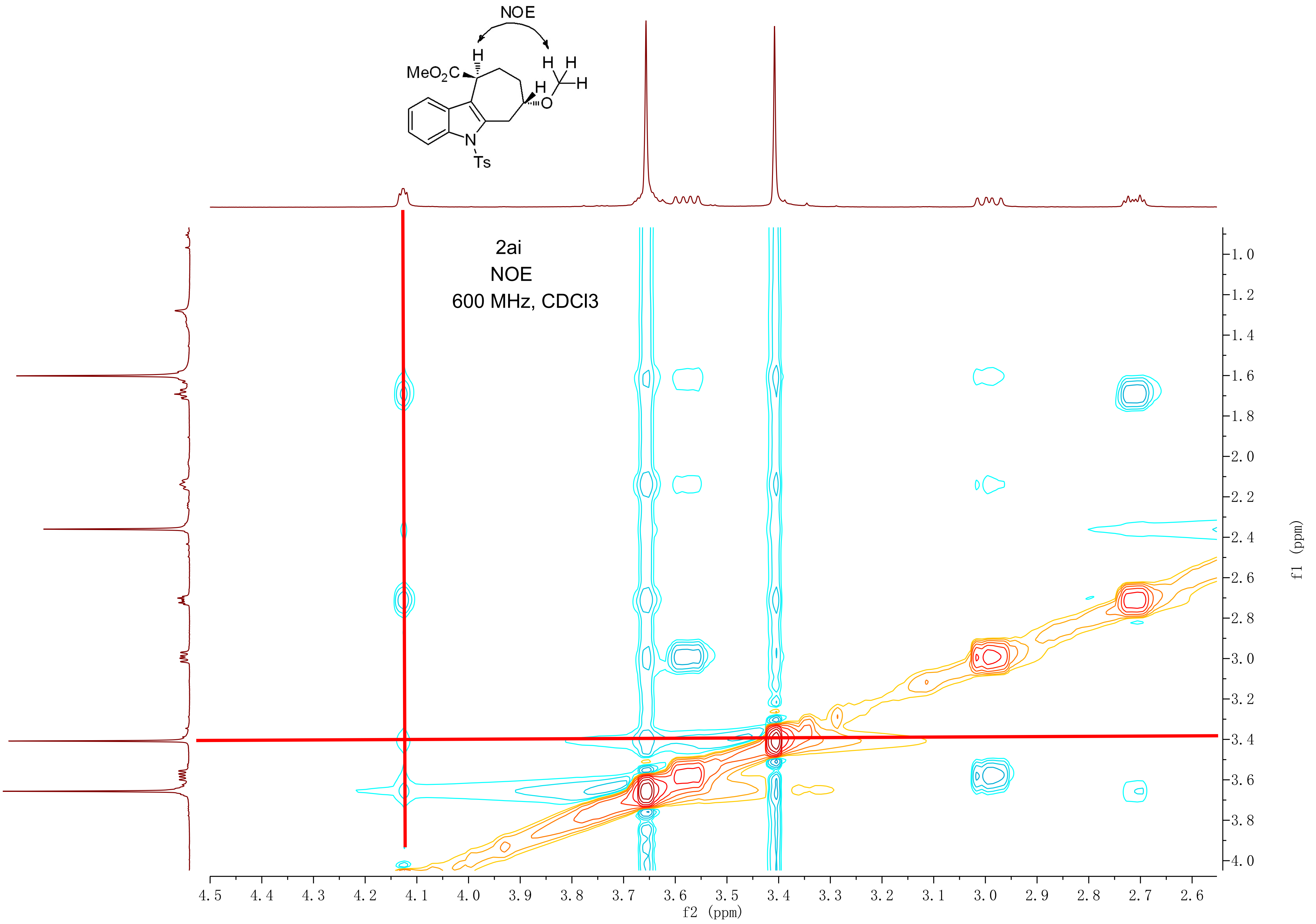
NOE
MeO2C
H
H
H
H
H
O
N
Ts
2ai
NOE
600 MHz, CDCl3
f2 (ppm)
f1 (ppm)

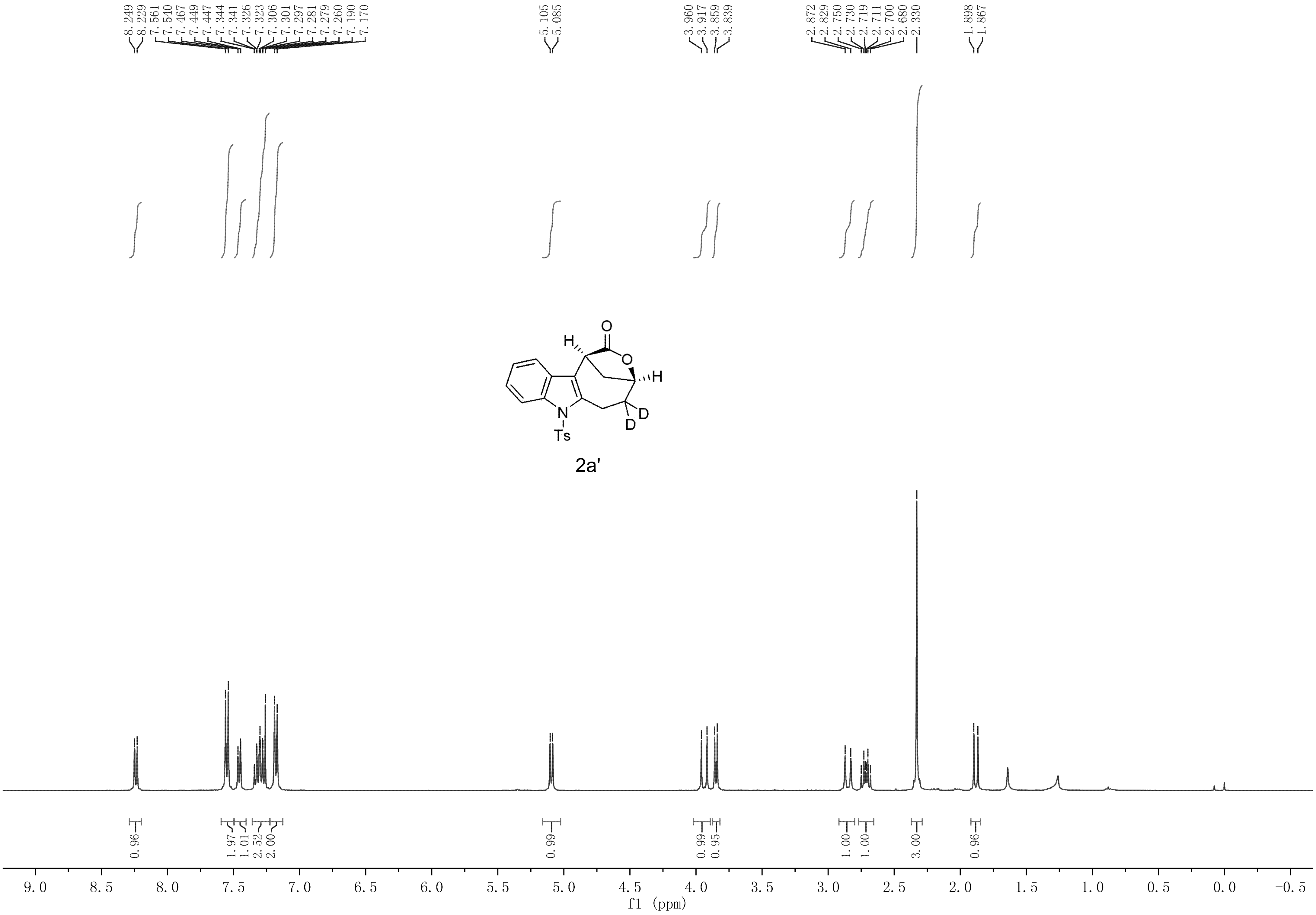
8.249
8.229
7.561
7.540
7.467
7.449
7.447
7.344
7.341
7.326
7.323
7.306
7.301
7.297
7.281
7.279
7.260
7.190
7.170
5.105
5.085
3.960
3.917
3.859
3.839
2.872
2.829
2.750
2.730
2.719
2.711
2.700
2.680
2.330
1.898
1.867
H
O
O
H
N
Ts
D
D
2a'
0.96
1.97
1.01
2.52
2.00
0.99
0.99
0.95
1.00
1.00
3.00
0.96
9.0
8.5
8.0
7.5
7.0
6.5
6.0
5.5
5.0
4.5
4.0
3.5
3.0
2.5
2.0
1.5
1.0
0.5
0.0
-0.5
f1 (ppm)

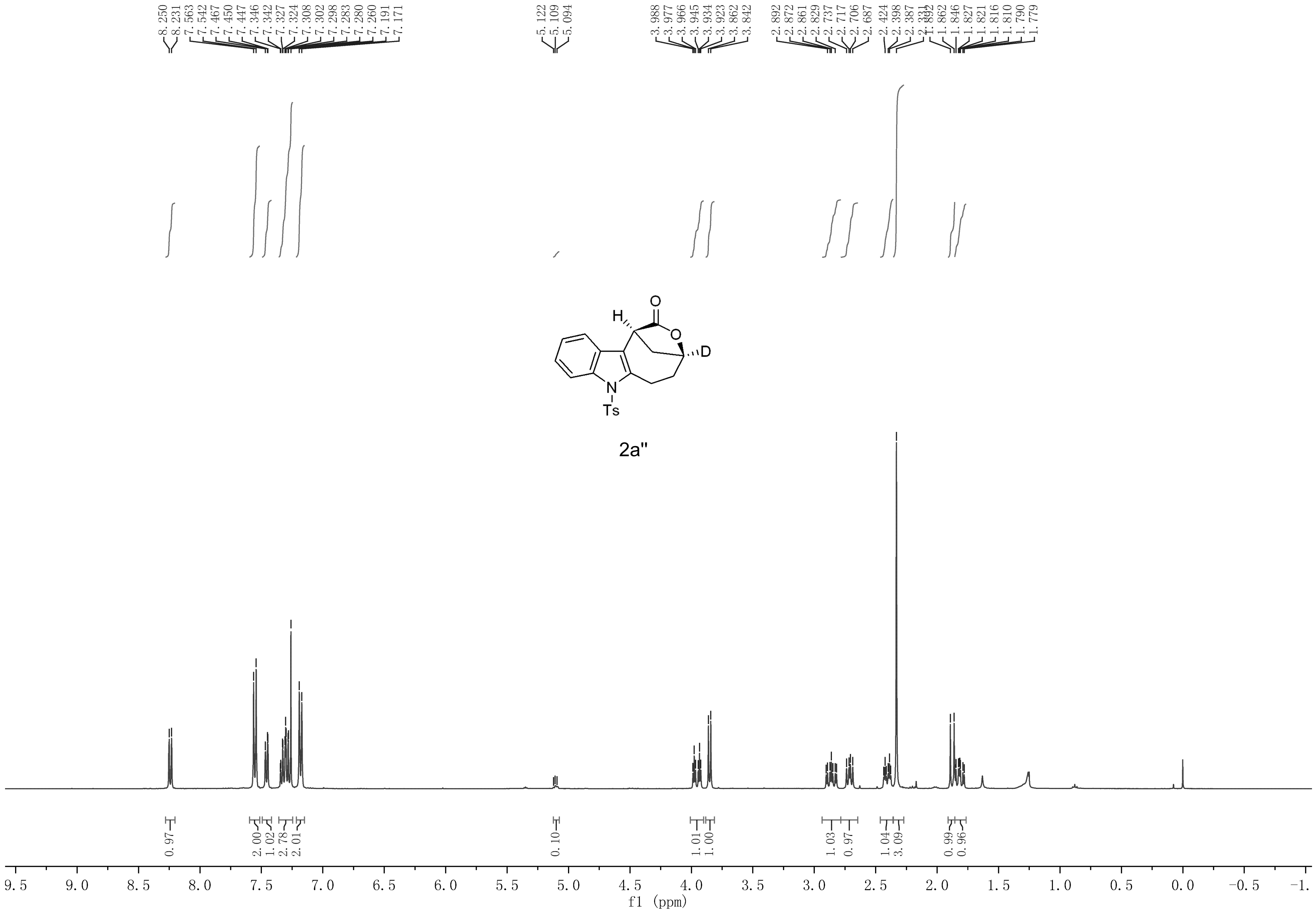
8.250
8.231
7.563
7.542
7.467
7.450
7.447
7.346
7.342
7.327
7.324
7.308
7.302
7.298
7.283
7.280
7.260
7.191
7.171
5.122
5.109
5.094
3.988
3.977
3.966
3.945
3.934
3.923
3.862
3.842
2.892
2.872
2.861
2.829
2.737
2.717
2.706
2.687
2.424
2.398
2.387
1.862
1.846
1.827
1.821
1.816
1.810
1.790
1.779
H
O
O
D
N
Ts
0.97
2.00
1.02
2.78
2.01
0.10
1.01
1.00
1.03
0.97
1.04
3.09
0.99
0.96
9.5
9.0
8.5
8.0
7.5
7.0
6.5
6.0
5.5
5.0
4.5
4.0
3.5
3.0
2.5
2.0
1.5
1.0
0.5
0.0
-0.5
-1.
f1 (ppm)

2a''

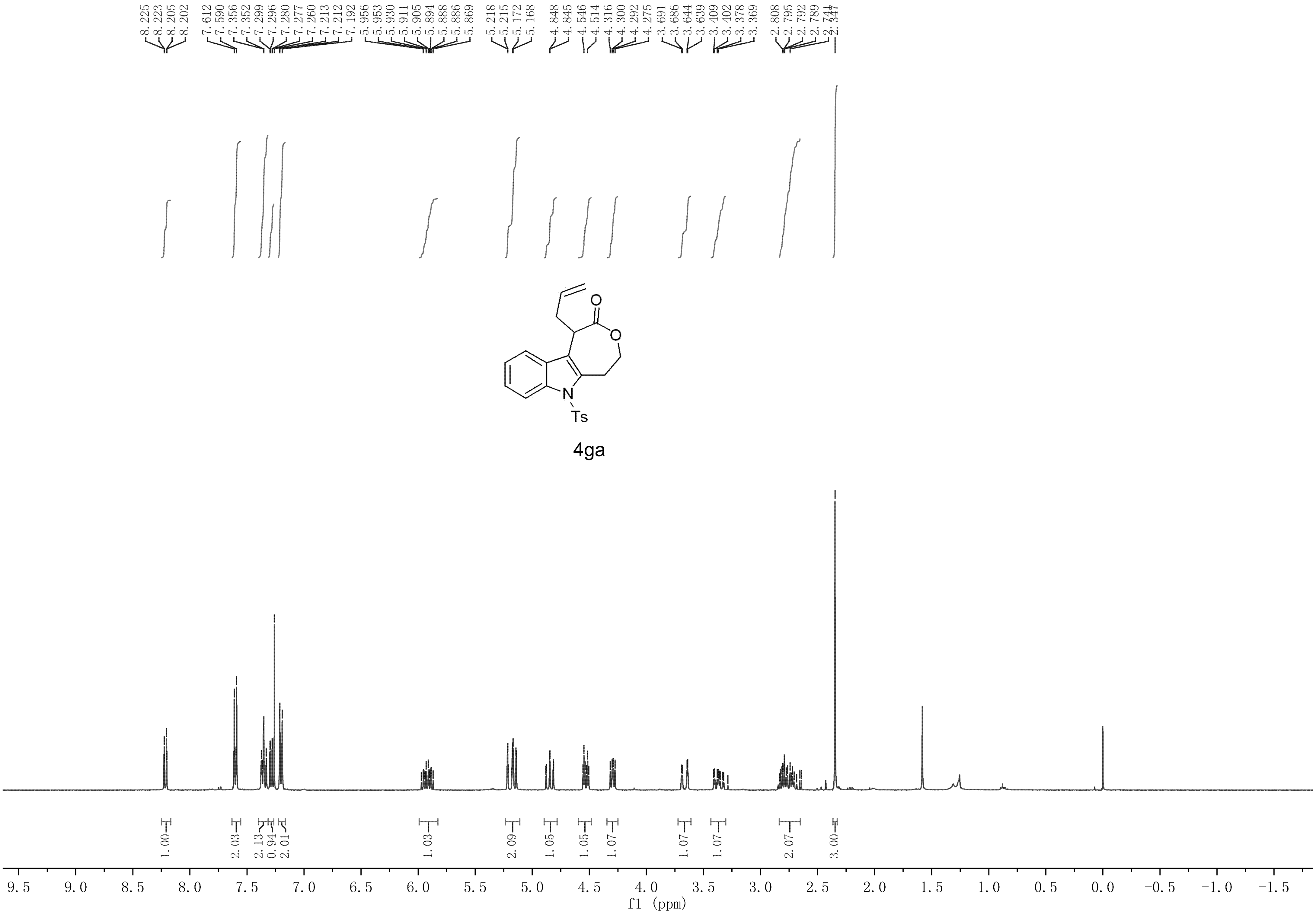

8.225
8.223
8.205
8.202
7.612
7.590
7.356
7.352
7.299
7.296
7.280
7.277
7.260
7.213
7.212
7.192
5.956
5.953
5.930
5.911
5.905
5.894
5.888
5.886
5.869
5.218
5.215
5.172
5.168
4.848
4.845
4.546
4.514
4.316
4.300
4.292
4.275
3.691
3.686
3.644
3.639
3.409
3.402
3.378
3.369
2.808
2.795
2.792
2.789
2.741
2.347
O
O
N
Ts
4ga
1.00
2.03
2.13
0.94
2.01
1.03
2.09
1.05
1.05
1.07
1.07
1.07
2.07
3.00
9.5
9.0
8.5
8.0
7.5
7.0
6.5
6.0
5.5
5.0
4.5
4.0
3.5
3.0
2.5
2.0
1.5
1.0
0.5
0.0
-0.5
-1.0
-1.5
f1 (ppm)

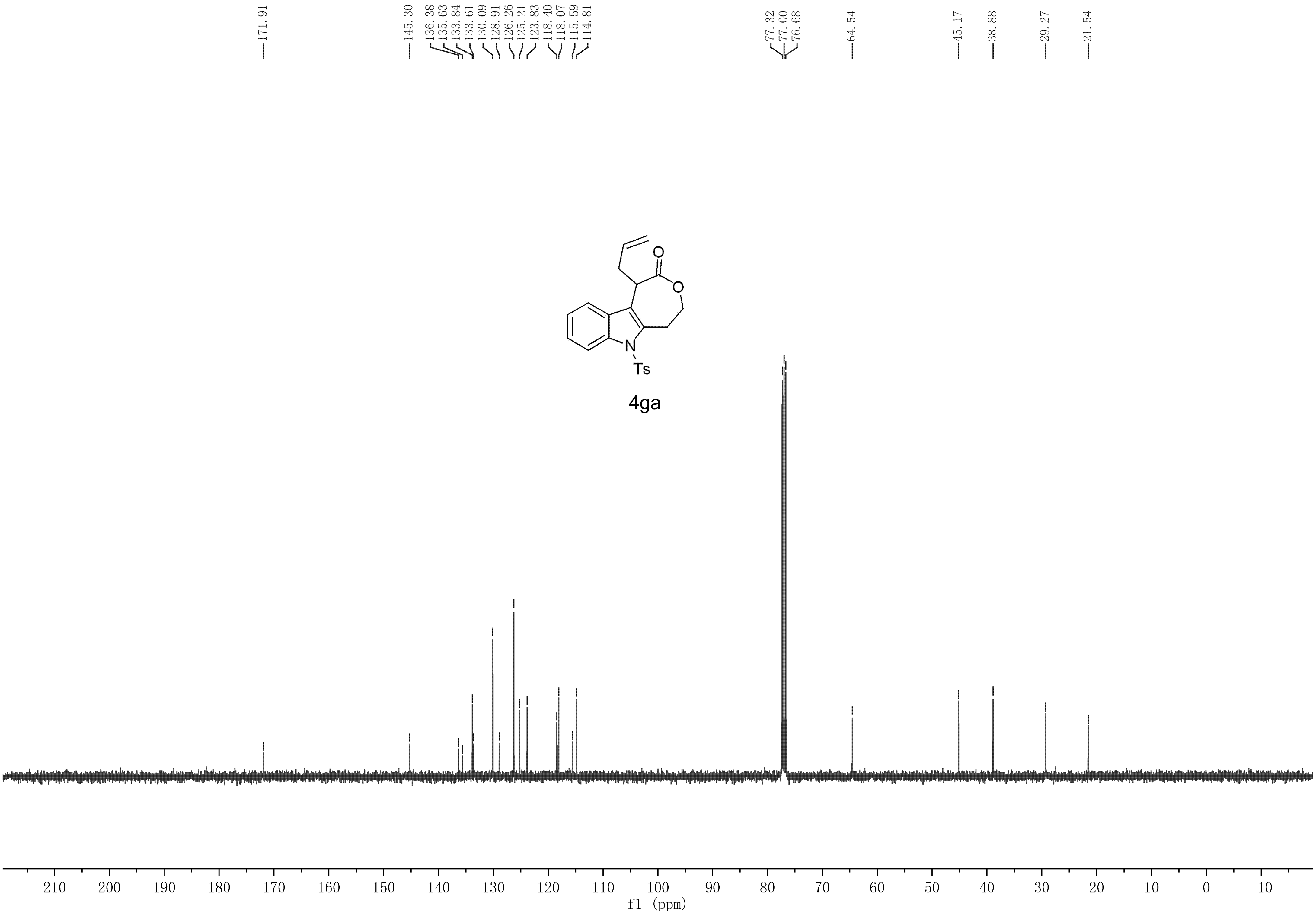

4ga
171.91
145.30
136.38
135.63
133.84
133.61
130.09
128.91
126.26
125.21
123.83
118.40
118.07
115.59
114.81
77.32
77.00
76.68
64.54
45.17
38.88
29.27
21.54
210
200
190
180
170
160
150
140
130
120
110
100
90
80
70
60
50
40
30
20
10
0
−10
f1 (ppm)

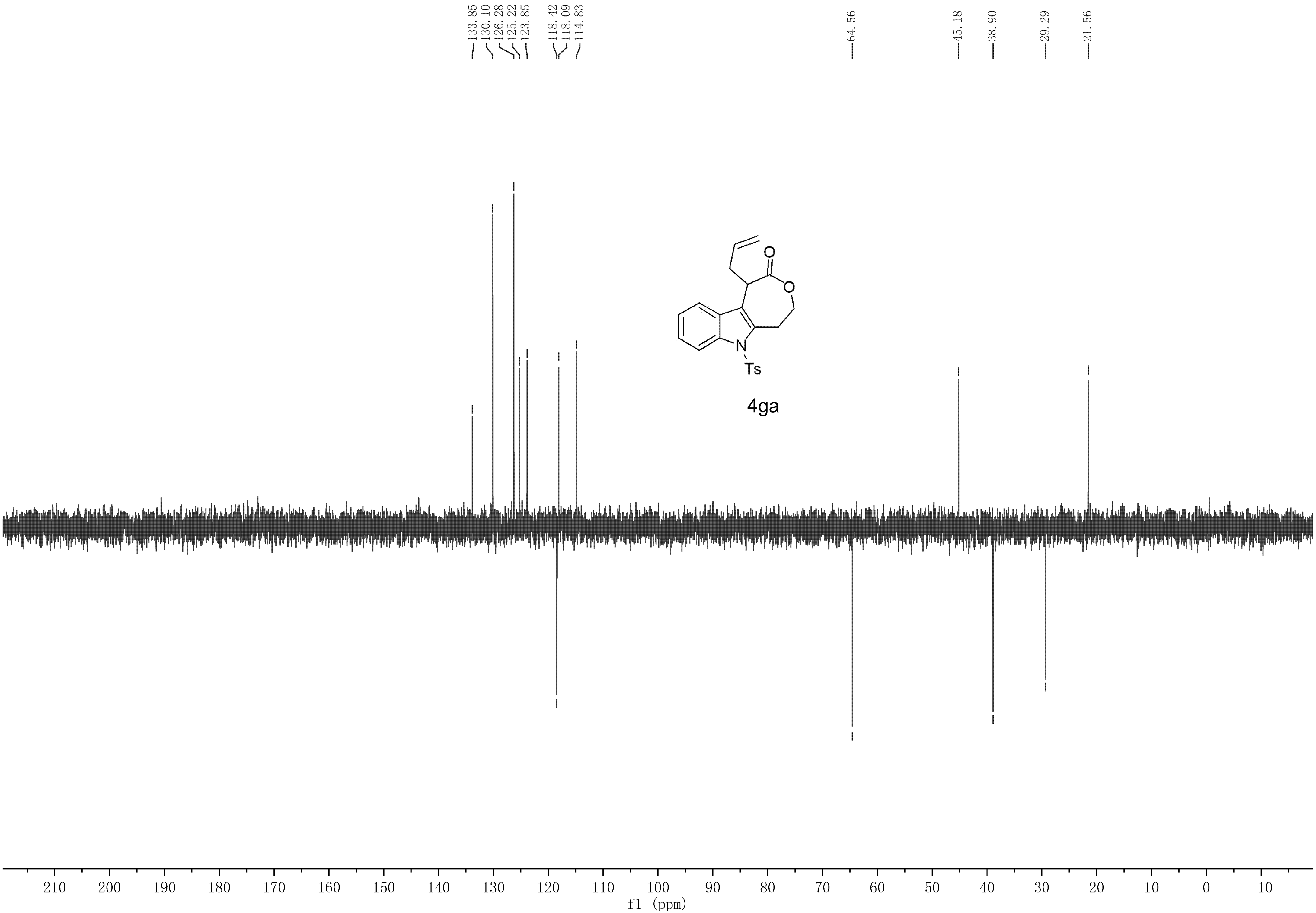
133.85
130.10
126.28
125.22
123.85
118.42
118.09
114.83
64.56
45.18
38.90
29.29
21.56
O
O
N
Ts
4ga
210
200
190
180
170
160
150
140
130
120
110
100
90
80
70
60
50
40
30
20
10
0
−10
f1 (ppm)

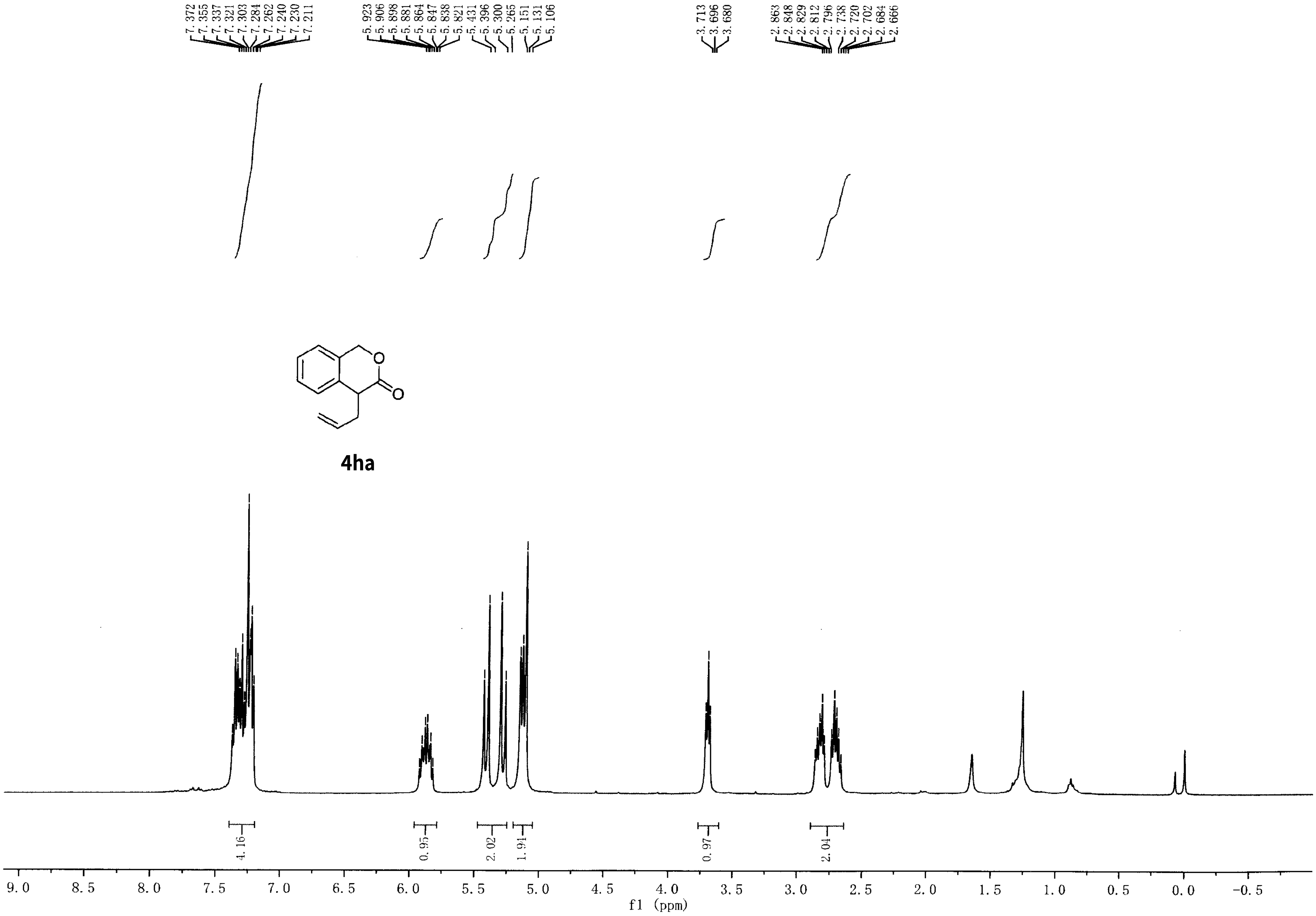

7.372
7.355
7.337
7.321
7.303
7.284
7.262
7.240
7.230
7.211
5.923
5.906
5.898
5.881
5.864
5.847
5.838
5.821
5.431
5.396
5.300
5.265
5.151
5.131
5.106
3.713
3.696
3.680
2.863
2.848
2.829
2.812
2.796
2.720
2.702
2.684
2.666
4ha
4.16
0.95
2.02
1.91
0.97
2.04
9.0
8.5
8.0
7.5
7.0
6.5
6.0
5.5
5.0
4.5
4.0
3.5
3.0
2.5
2.0
1.5
1.0
0.5
0.0
-0.5
f1 (ppm)

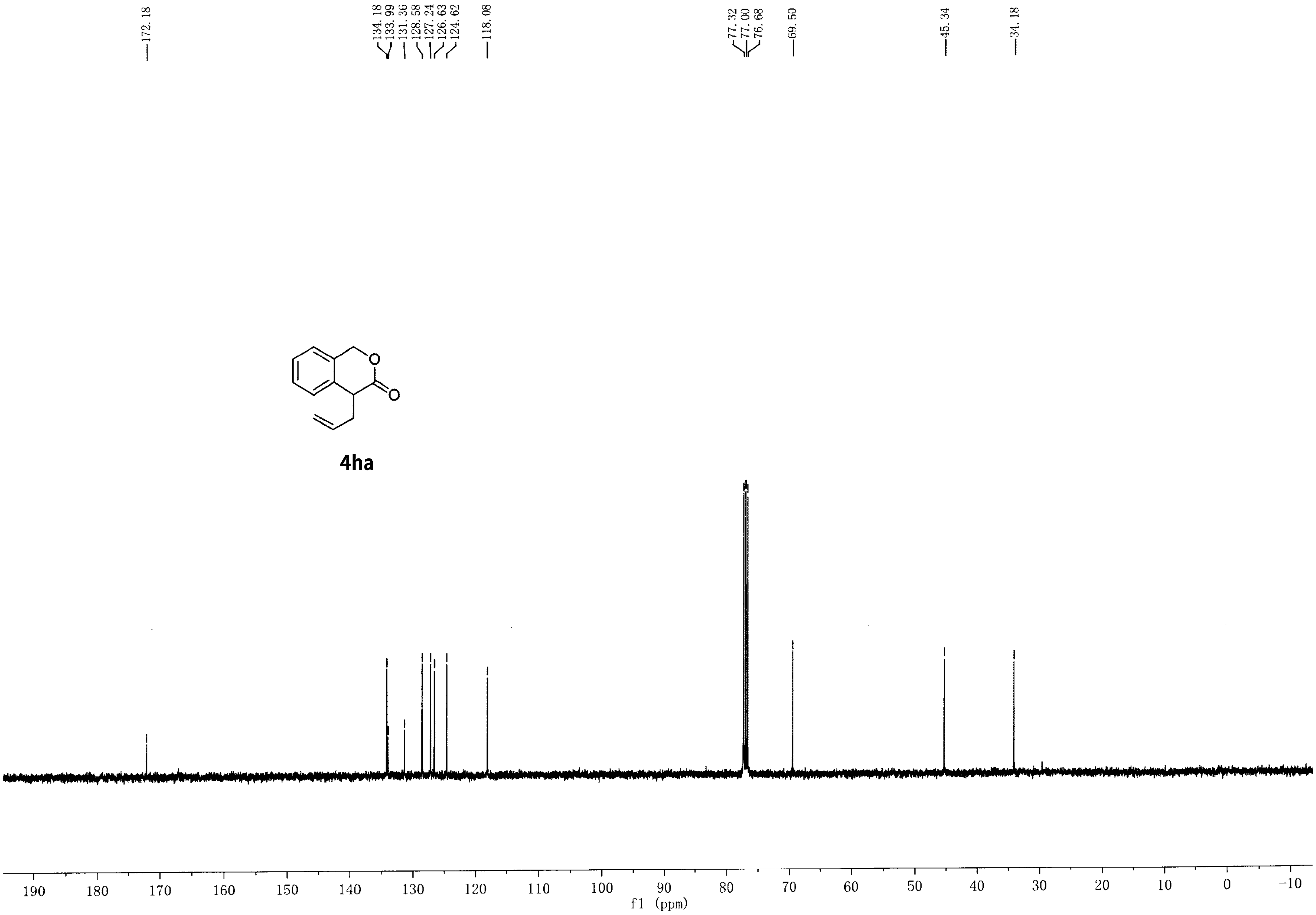

172.18
134.18
133.99
131.36
128.58
127.24
126.63
124.62
118.08
77.32
77.00
76.68
69.50
45.34
34.18
190 180 170 160 150 140 130 120 110 100 90 80 70 60 50 40 30 20 10 0 −10
f1 (ppm)

4ha
